# Supplementary material for: Selenide-catalyzed enantioselective synthesis of trifluoromethylthiolated tetrahydronaphthalenes by merging desymmetrization and trifluoromethylthiolation
Source: Nat Commun. 2018 Feb 6;9:527. doi: 10.1038/s41467-018-02955-0 (PMC5802806; doi:10.1038/s41467-018-02955-0)
Supplement: Supplementary file 1 — Supplementary Information [file 41467_2018_2955_MOESM1_ESM.pdf]

Selenide-Catalyzed Enantioselective Synthesis of Trifluoromethylthiolated Tetrahydronaphthalenes by Merging Desymmetrization and Trifluoromethylthiolation

Luo *et al.*

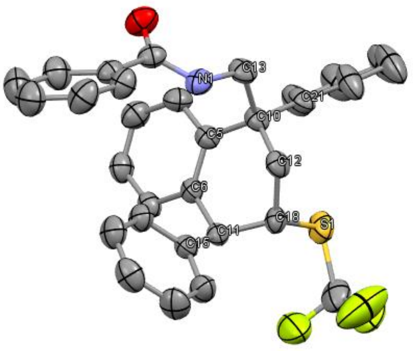

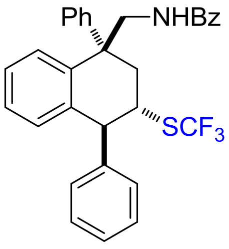

2a
CCDC 1523336

Supplementary Figure 1. X-ray of **2a**

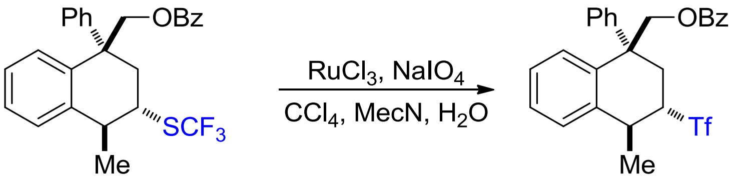

2s **2s'**
CCDC 1577179

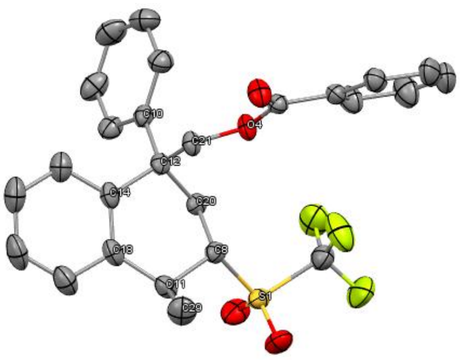

Supplementary Figure 2. X-ray of **2s'**

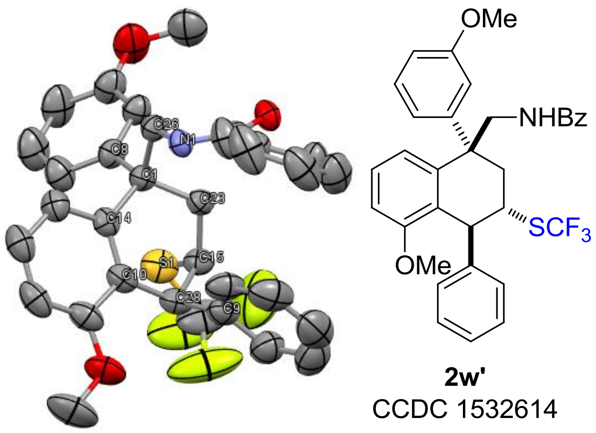

2w'
CCDC 1532614

Supplementary Figure 3. X-ray of regioisomer of **2w** (**2w'**)

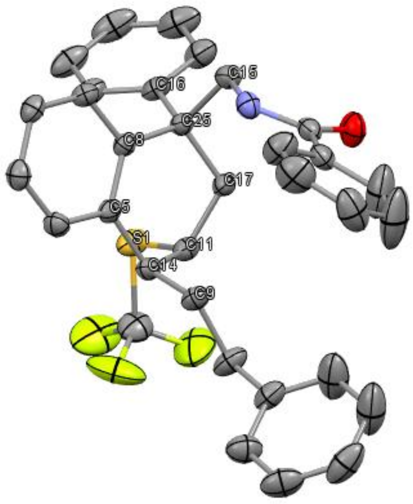

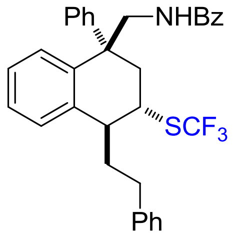

2j
CCDC 1533403

Supplementary Figure 4. X-ray of **2j**

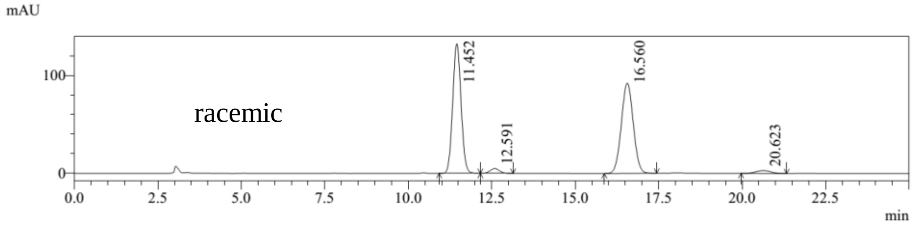

Peak Table

PDA Ch1 222nm

Peak#	Ret. Time	Area	Height	Area%	Height%
1	11.452	2314504	132598	48.177	56.957
2	12.591	99964	4965	2.081	2.133
3	16.560	2296285	92302	47.797	39.648
4	20.623	93448	2939	1.945	1.262
Total		4804202	232803	100.000	100.000

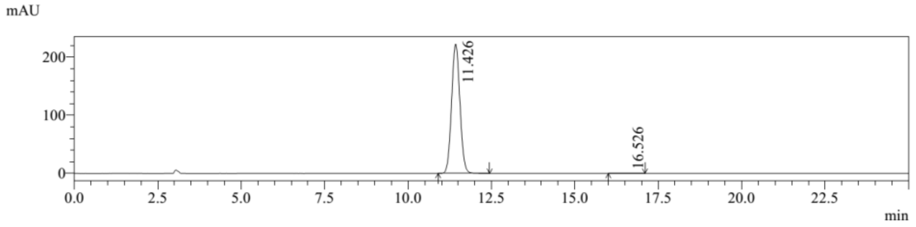

Peak Table

PDA Ch1 222nm

Peak#	Ret. Time	Area	Height	Area%	Height%
1	11.426	3888230	222829	99.942	99.957
2	16.526	2257	97	0.058	0.043
Total		3890486	222926	100.000	100.000

Supplementary Figure 5. HPLC for recovered crystal of **2j** (99.9% ee)

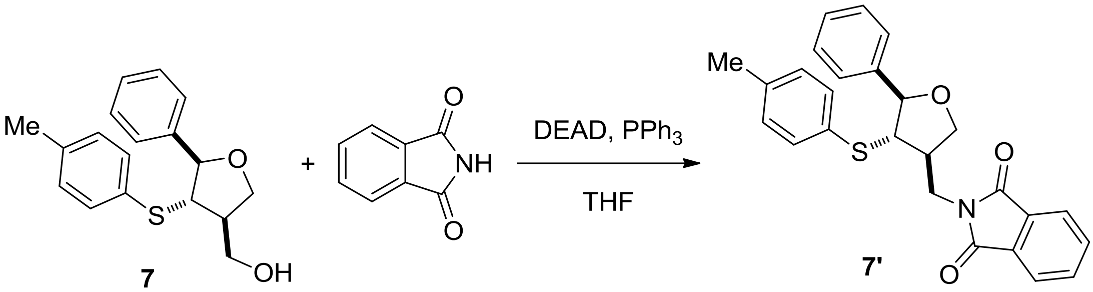

CCDC 1540104

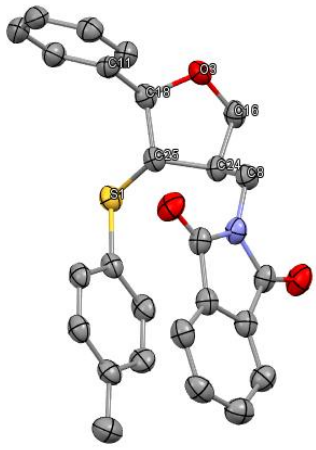

Supplementary Figure 6. X-ray of **7’**

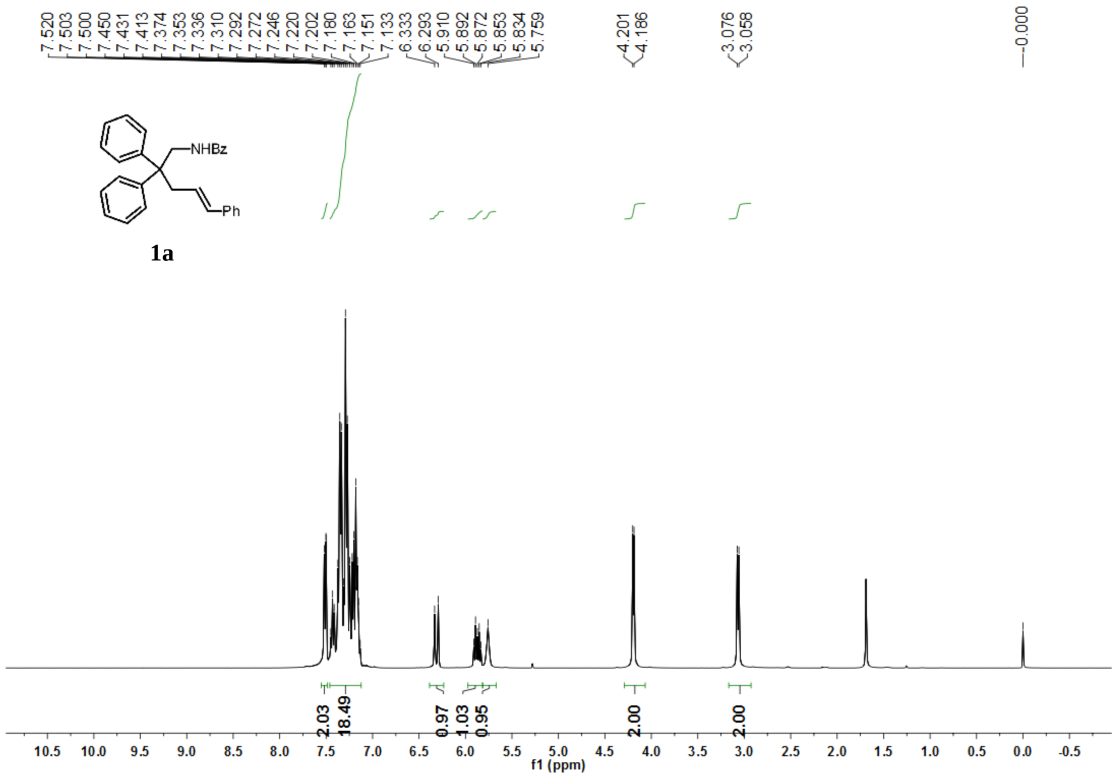

Supplementary Figure 7. ^{1}H NMR spectrum of compound **1a** in $CDCl_3$

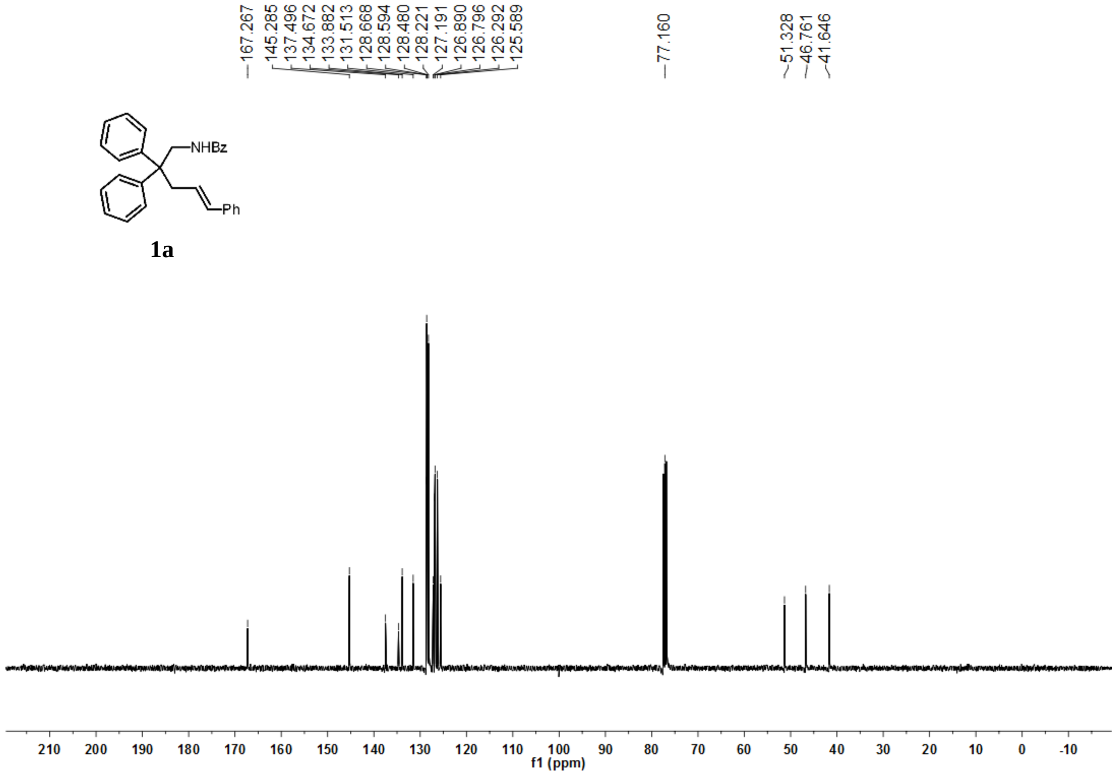

Supplementary Figure 8. ^{13}C NMR spectrum of compound **1a** in $CDCl_3$

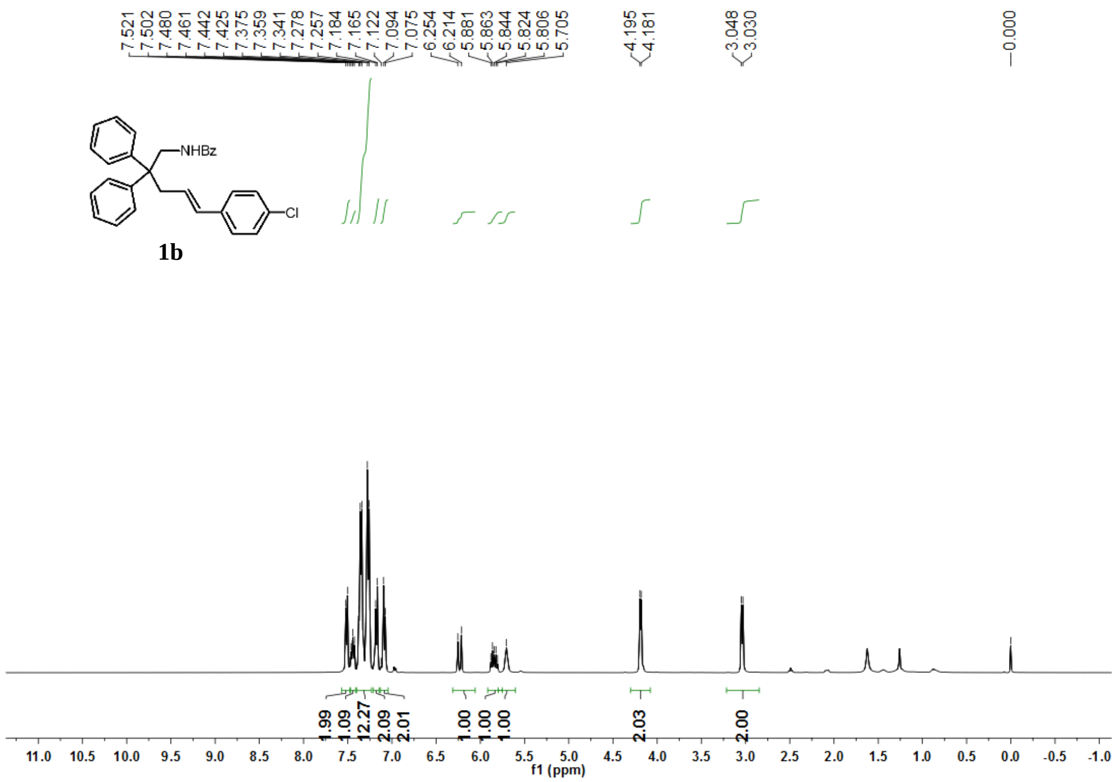

Supplementary Figure 9. ^{1}H NMR spectrum of compound **1b** in $CDCl_3$

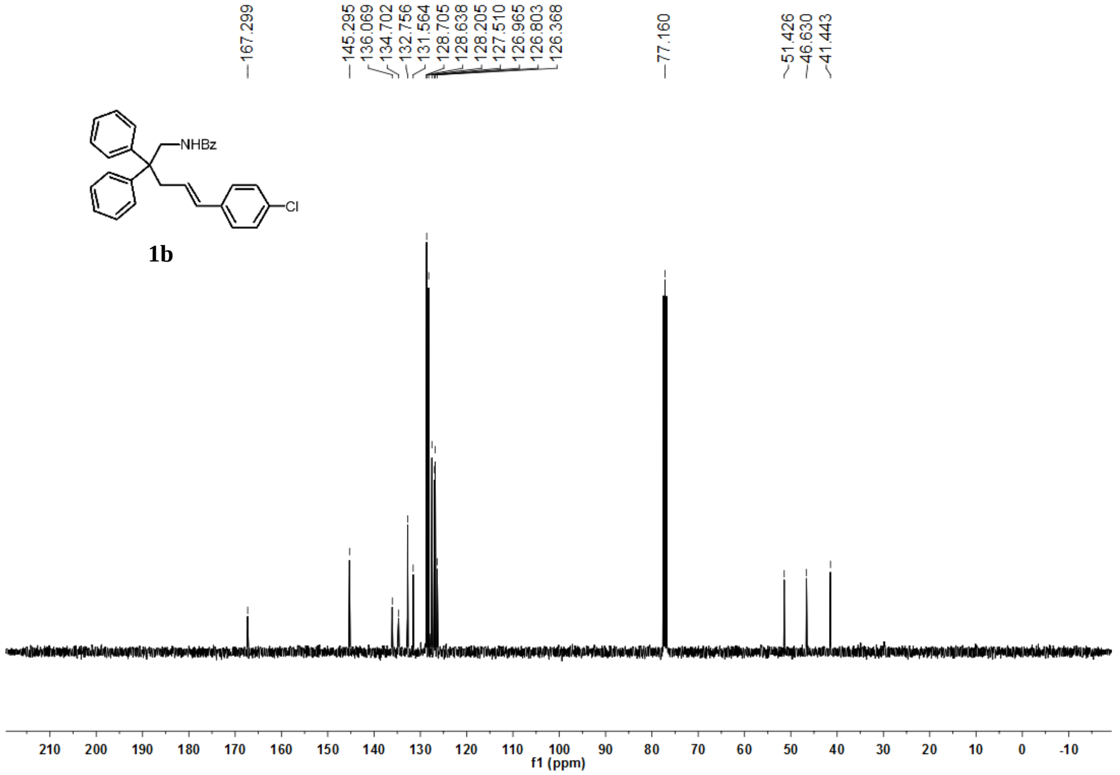

Supplementary Figure 10. ^{13}C NMR spectrum of compound **1b** in $CDCl_3$

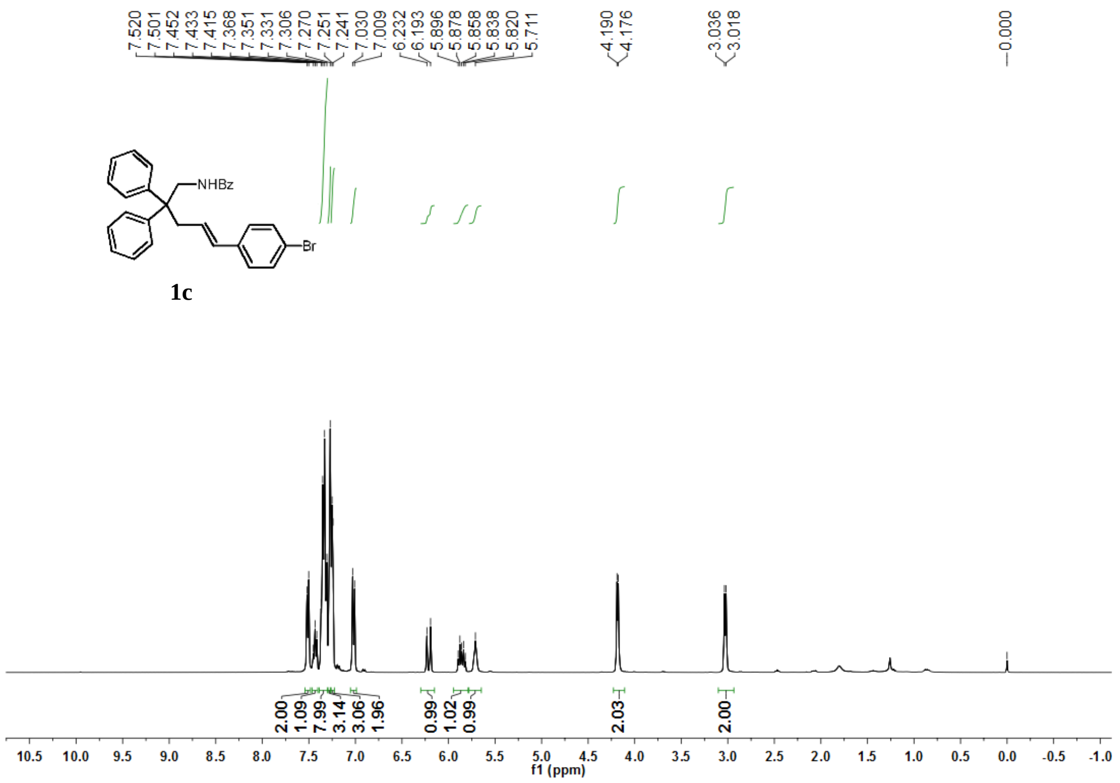

Supplementary Figure 11. ^{1}H NMR spectrum of compound **1c** in $CDCl_3$

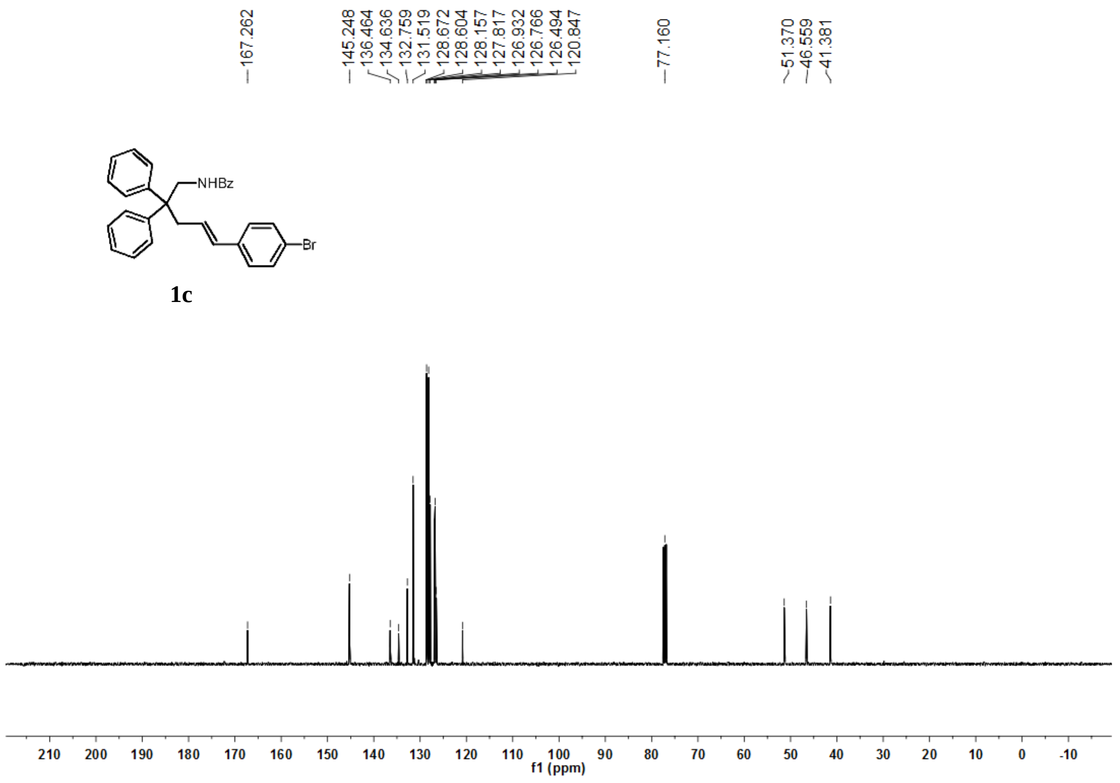

Supplementary Figure 12. ^{13}C NMR spectrum of compound **1c** in $CDCl_3$

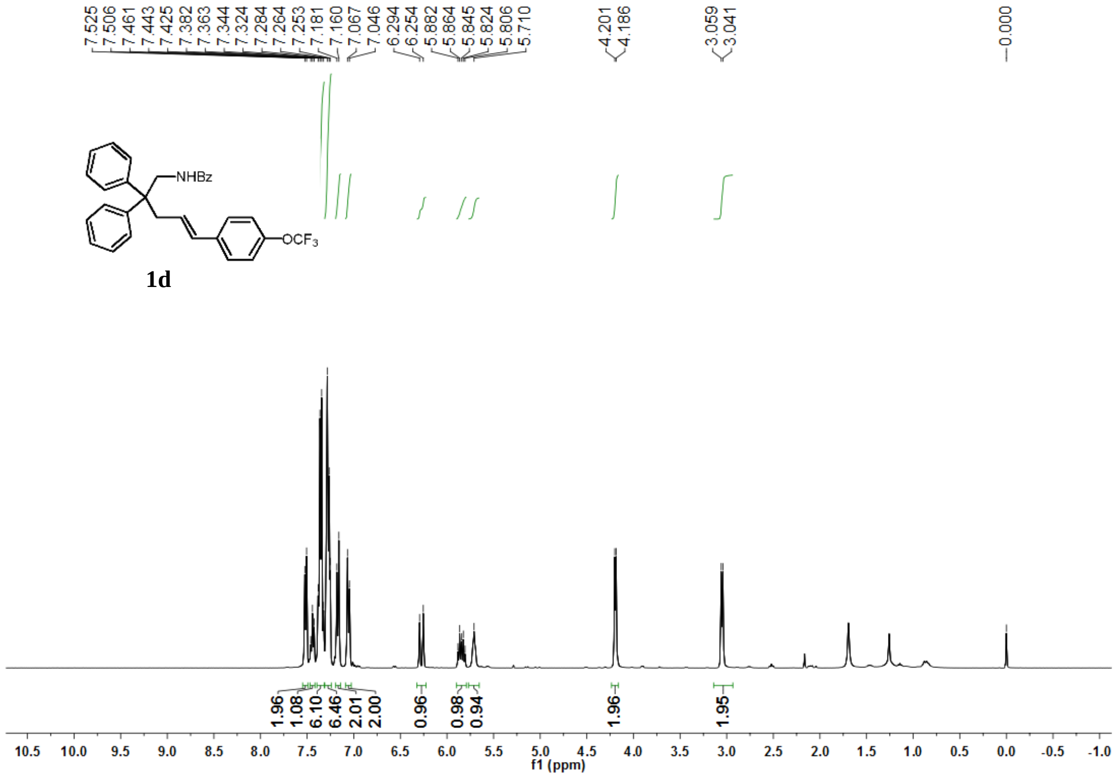

Supplementary Figure 13. ^{1}H NMR spectrum of compound **1d** in $CDCl_3$

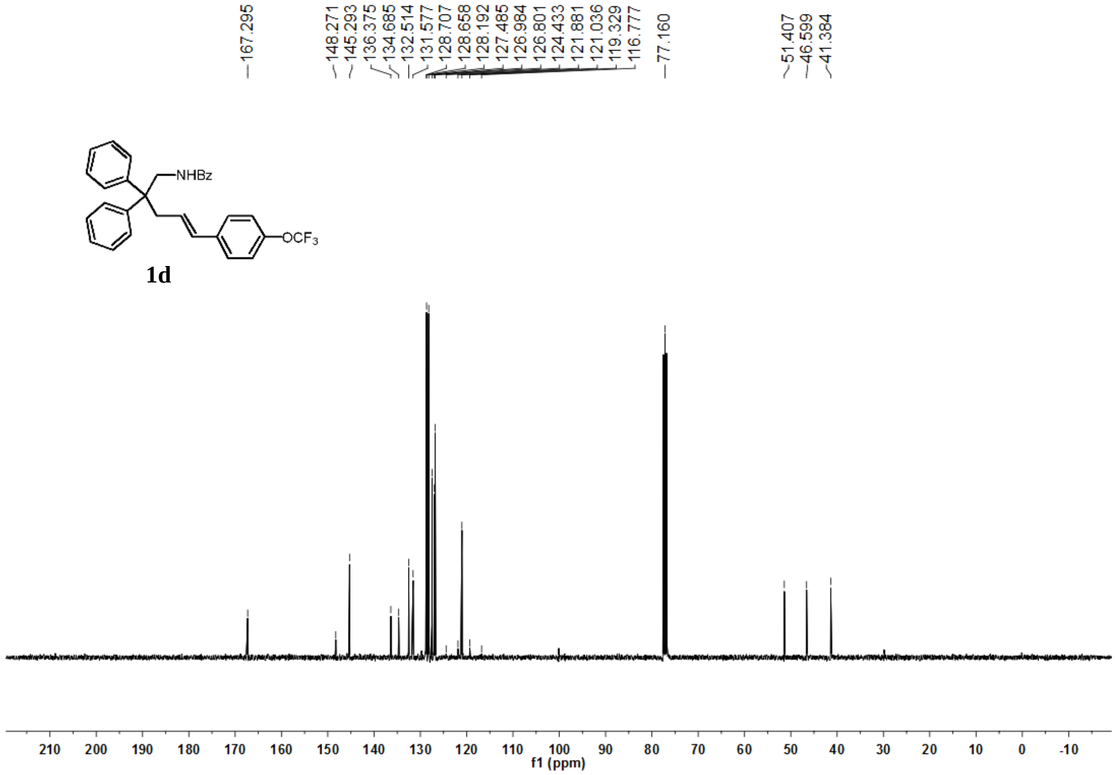

Supplementary Figure 14. ^{13}C NMR spectrum of compound **1d** in $CDCl_3$

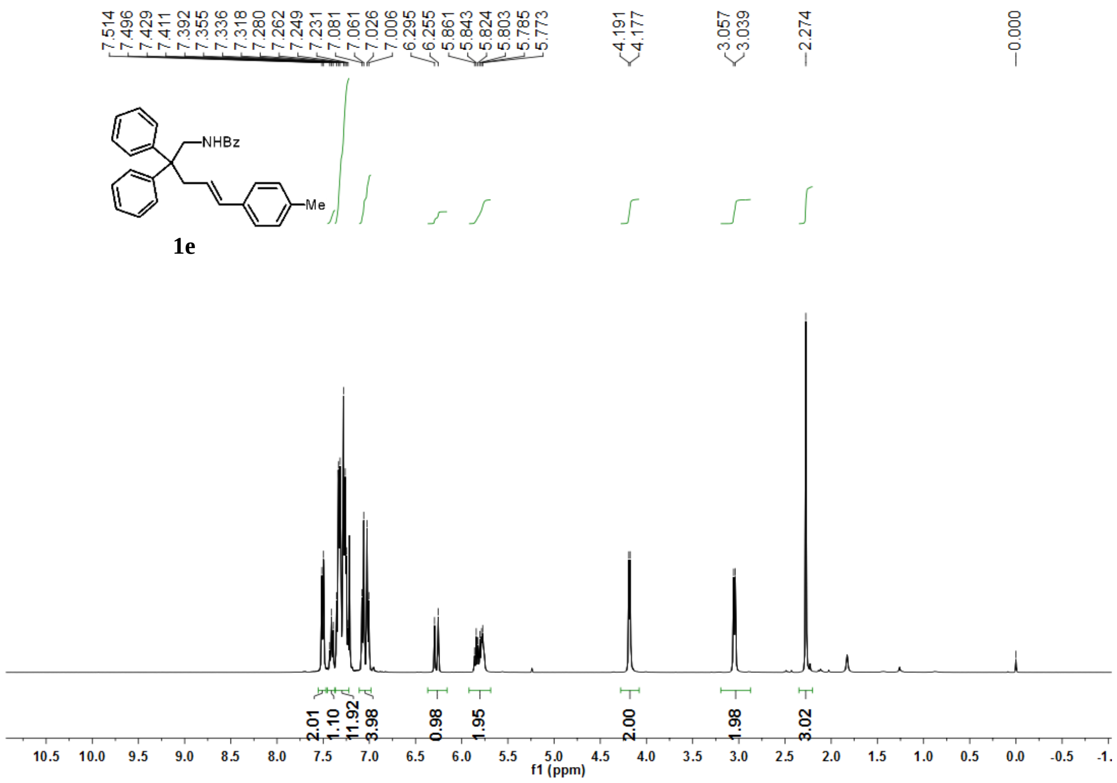

Supplementary Figure 15. ^{1}H NMR spectrum of compound **1e** in $CDCl_3$

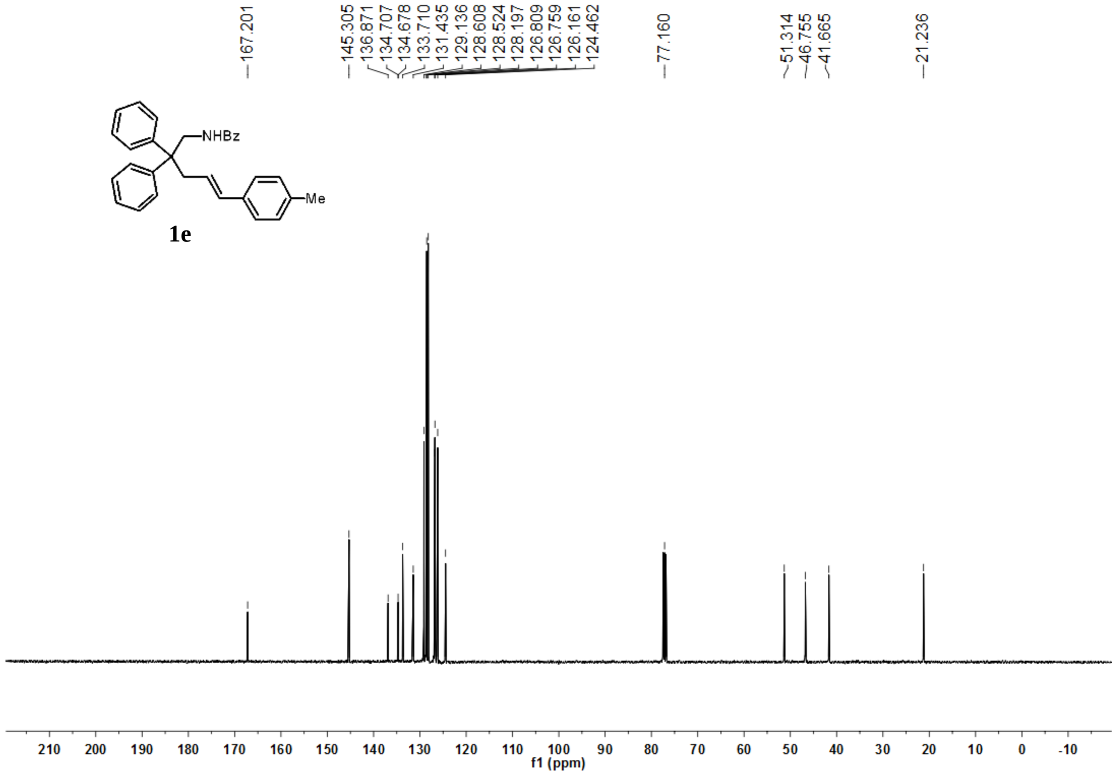

Supplementary Figure 16. ^{13}C NMR spectrum of compound **1e** in $CDCl_3$

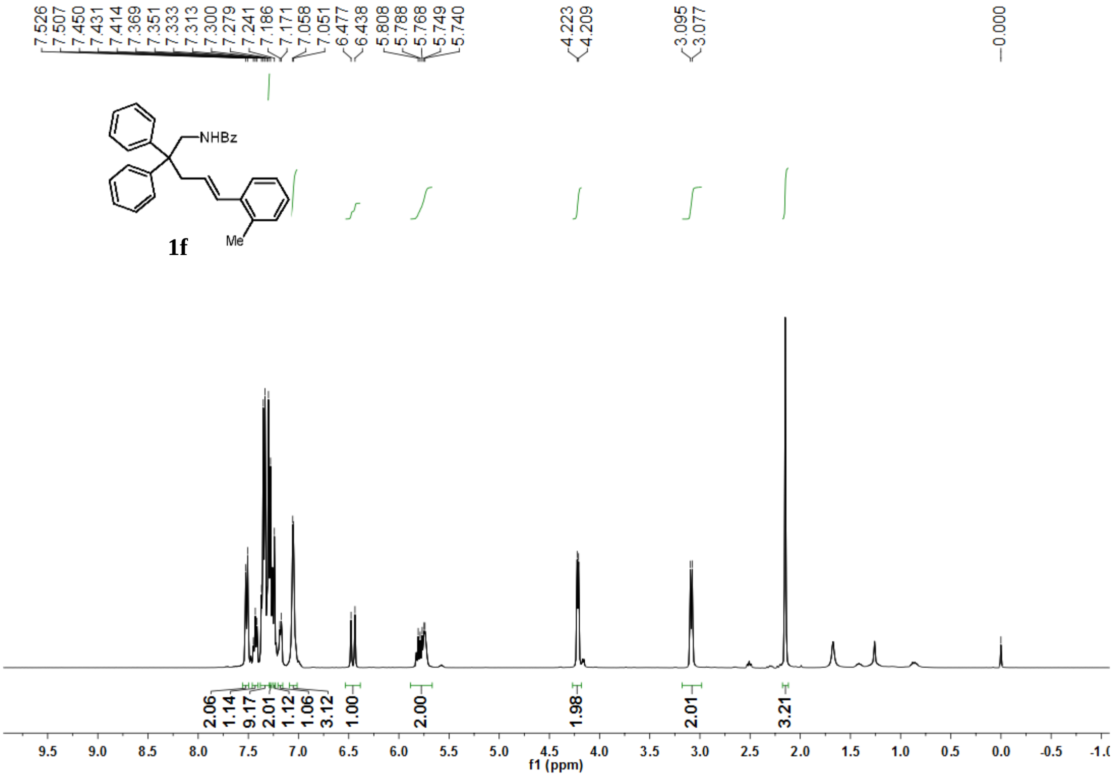

Supplementary Figure 17. ^{1}H NMR spectrum of compound **1f** in $CDCl_3$

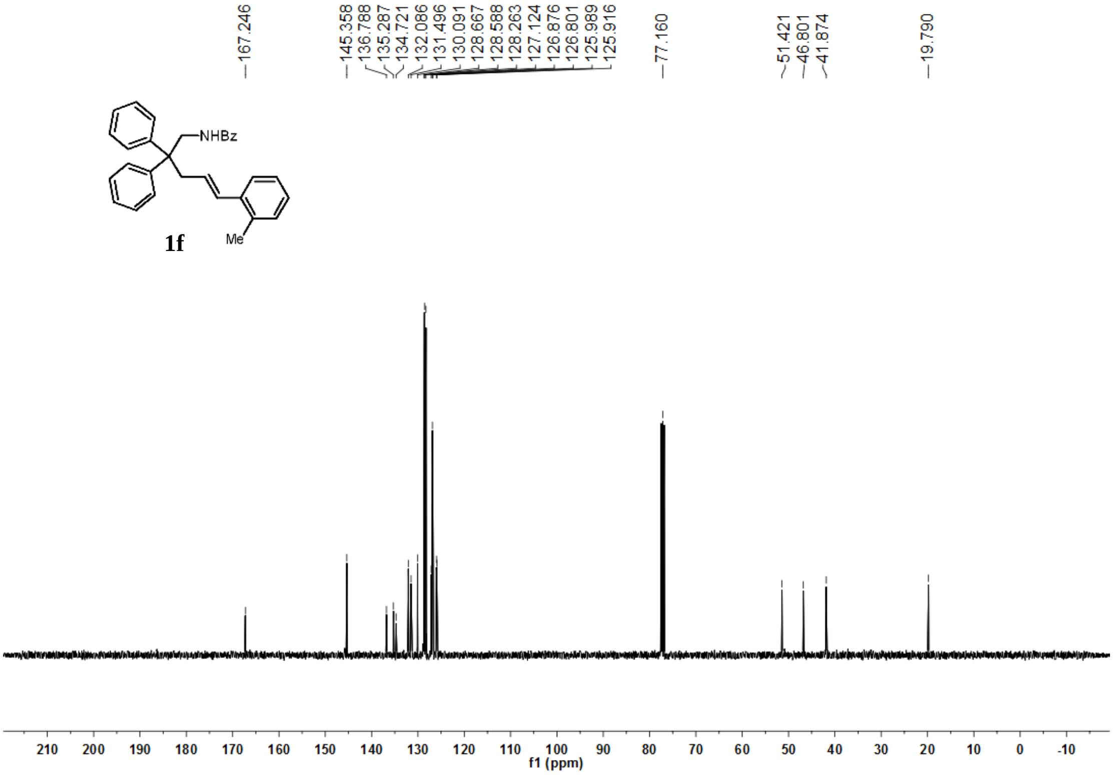

Supplementary Figure 18. ^{13}C NMR spectrum of compound **1f** in $CDCl_3$

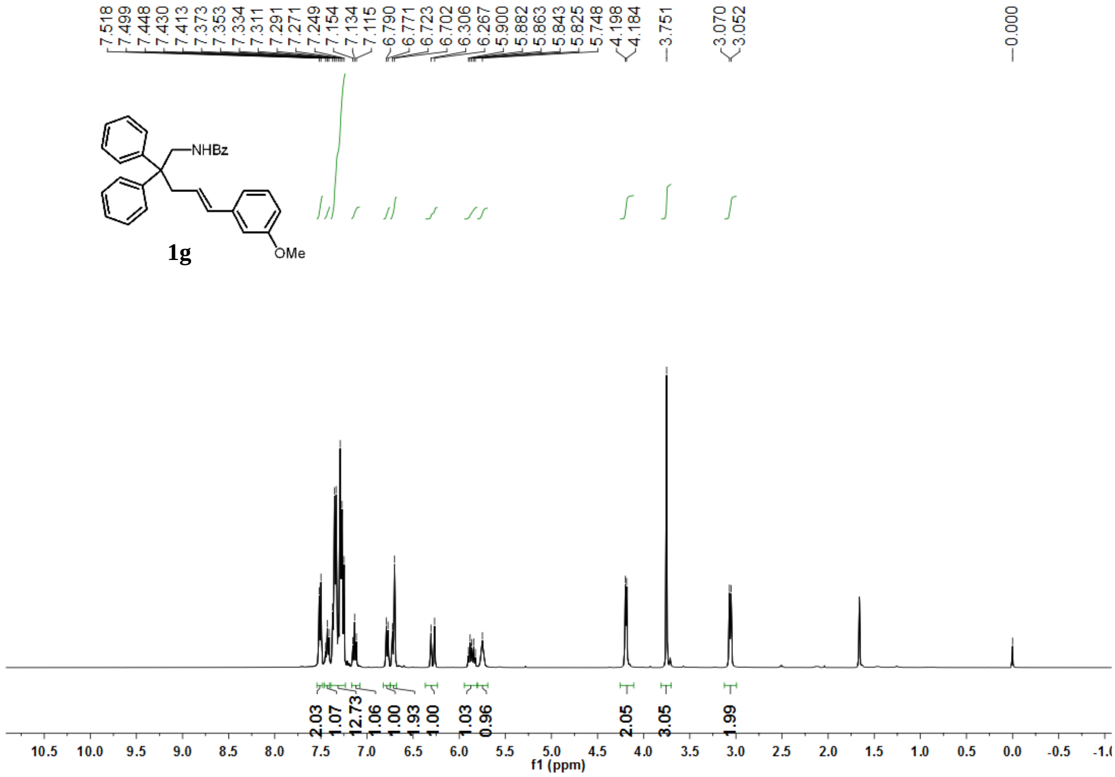

Supplementary Figure 19. ^{1}H NMR spectrum of compound **1g** in $CDCl_3$

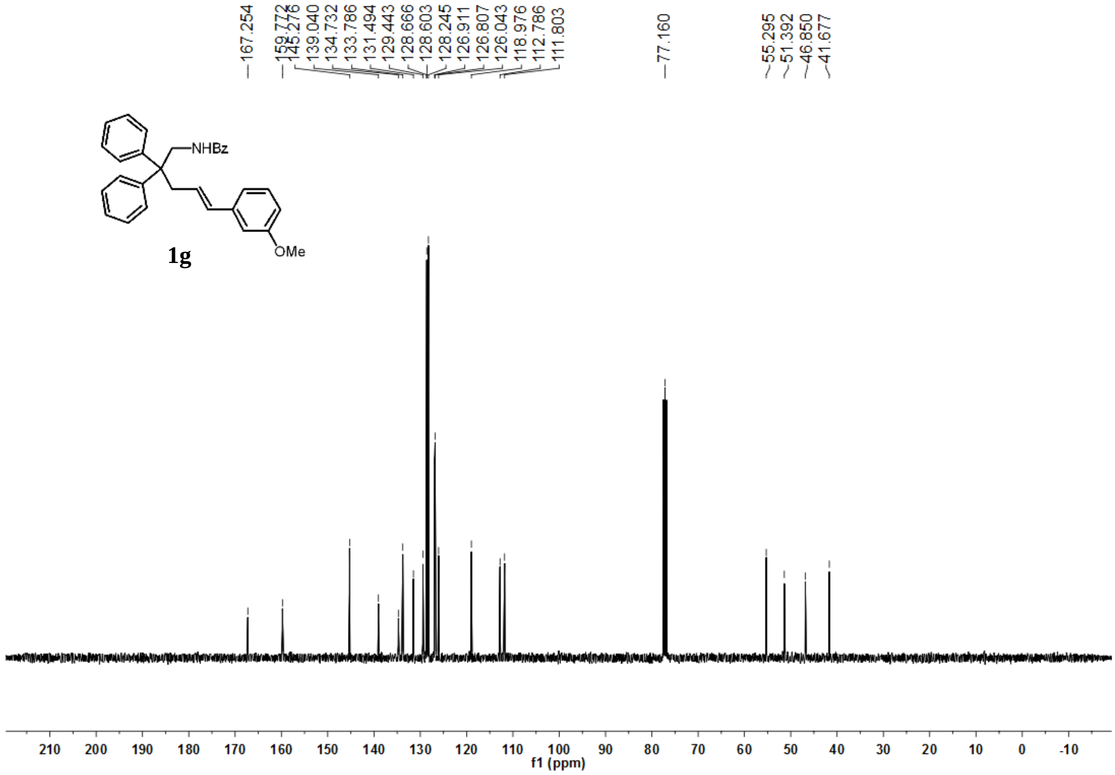

Supplementary Figure 20. ^{13}C NMR spectrum of compound **1g** in $CDCl_3$

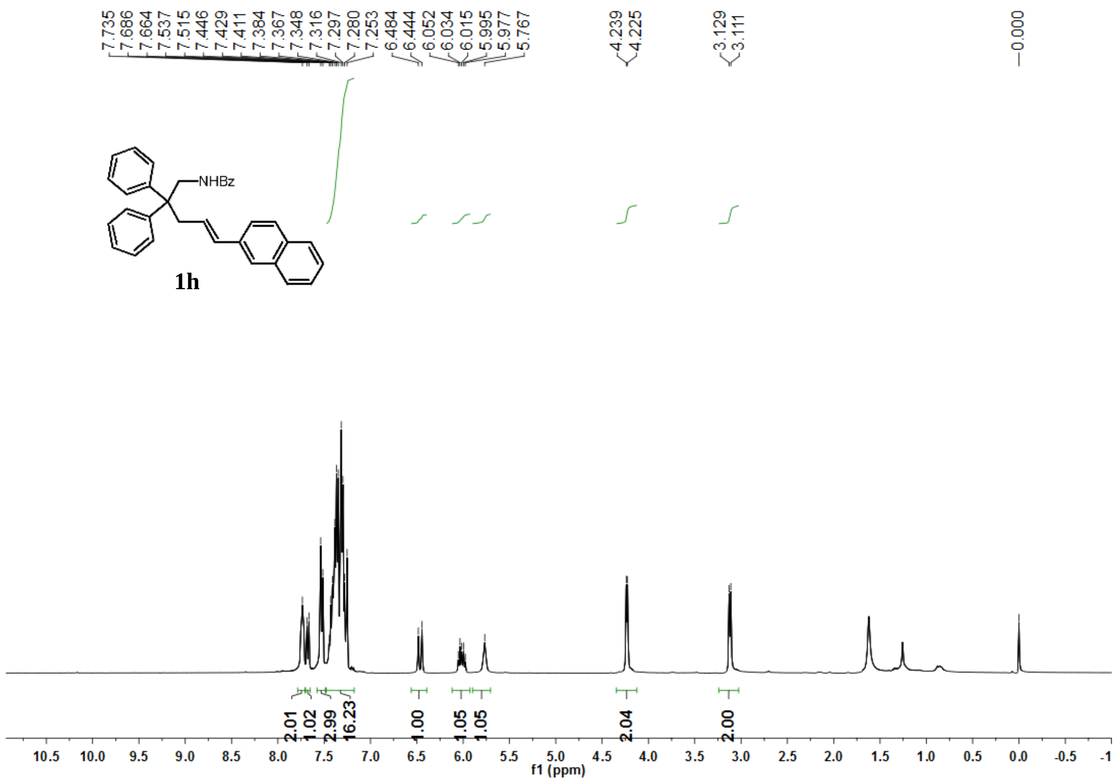

Supplementary Figure 21. ^{1}H NMR spectrum of compound **1h** in $CDCl_3$

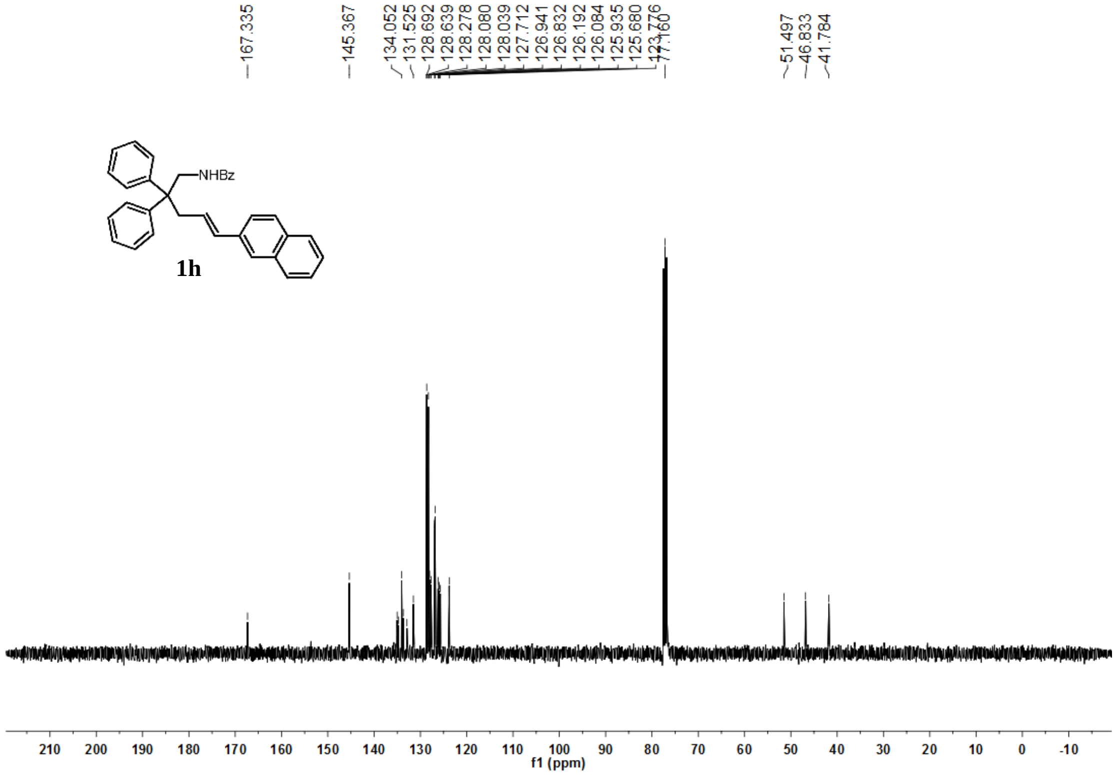

Supplementary Figure 22. ^{13}C NMR spectrum of compound **1h** in $CDCl_3$

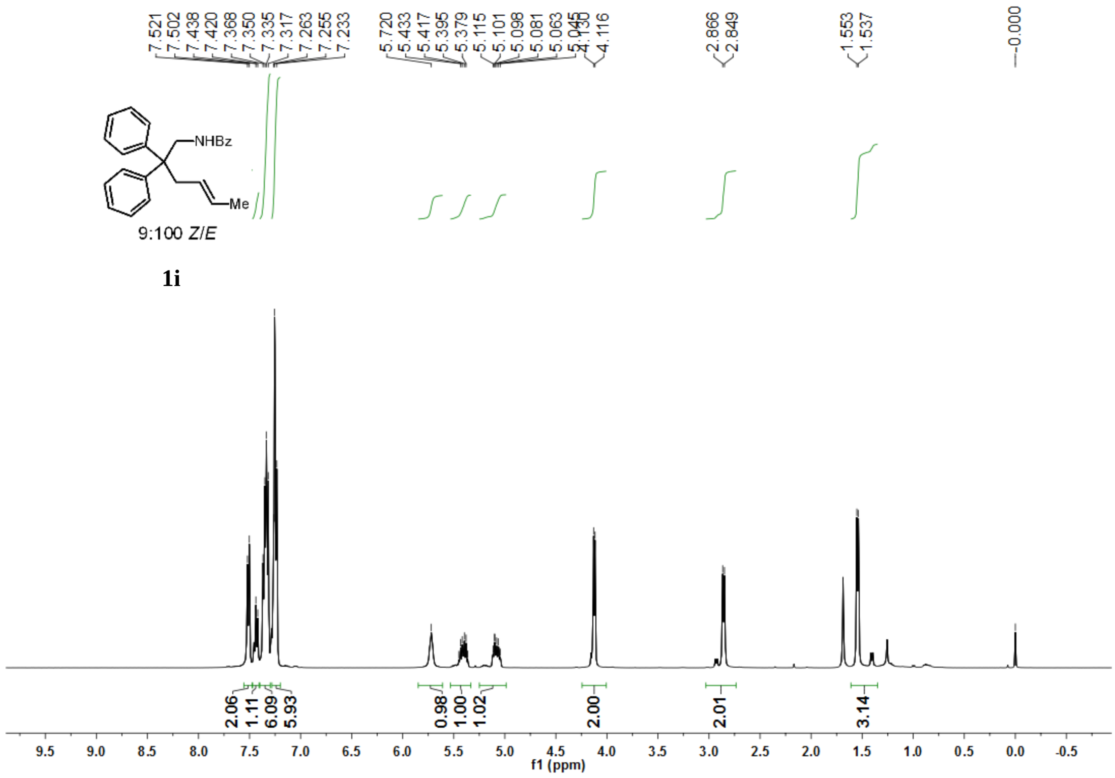

Supplementary Figure 23. ^{1}H NMR spectrum of compound **1i** in $CDCl_3$

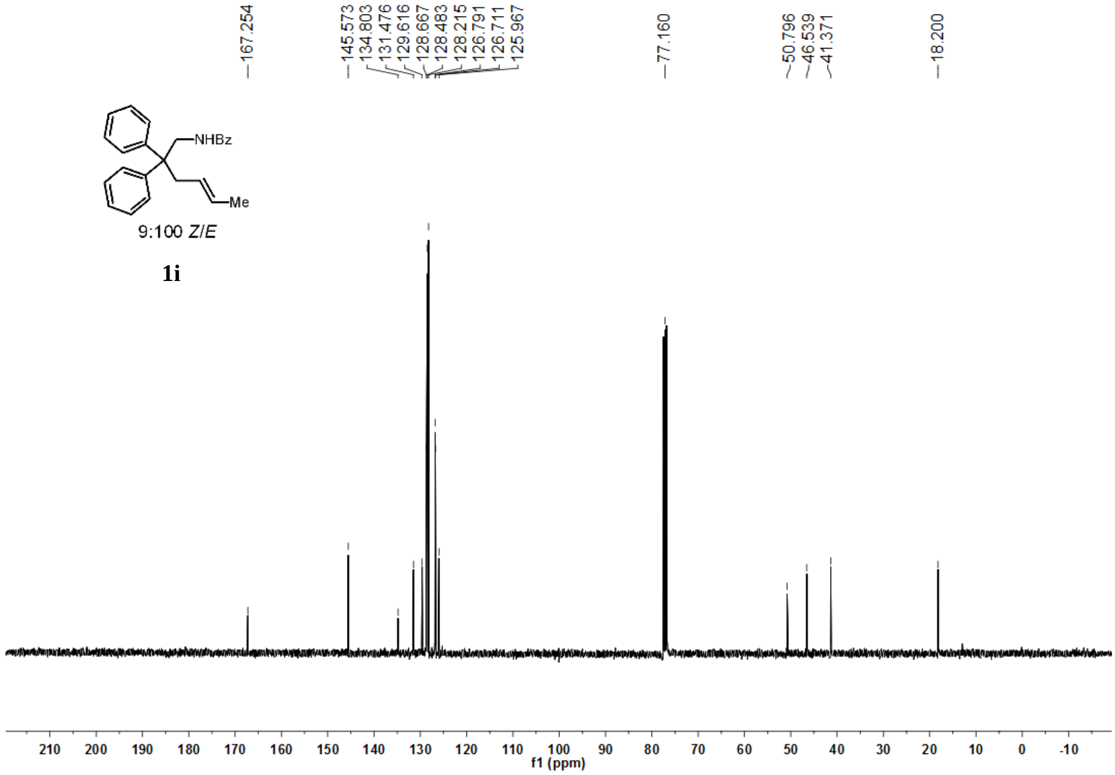

Supplementary Figure 24. ^{13}C NMR spectrum of compound **1i** in $CDCl_3$

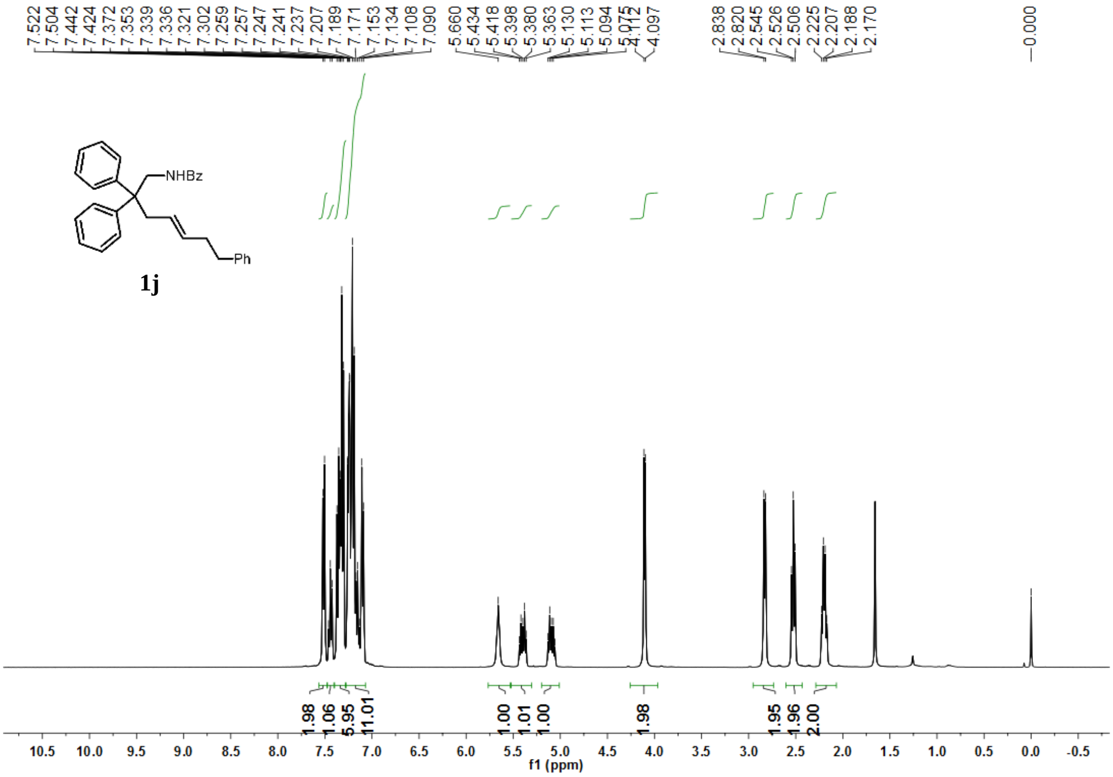

Supplementary Figure 25. ^{1}H NMR spectrum of compound **1j** in $CDCl_3$

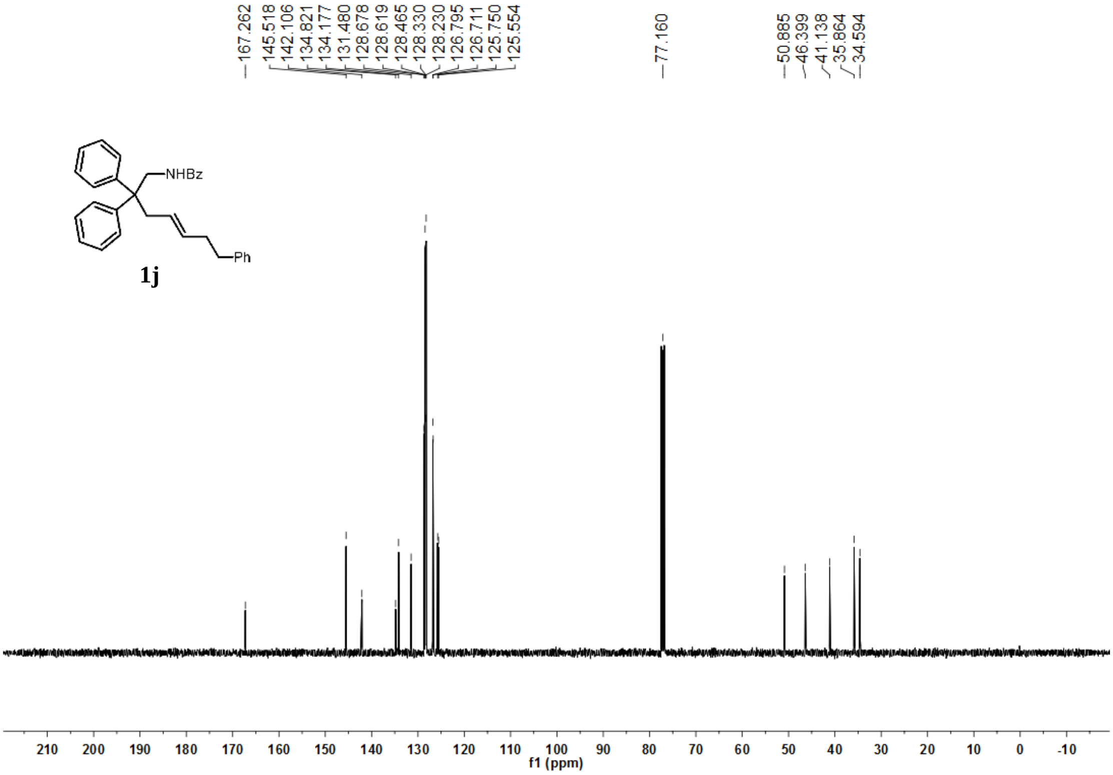

Supplementary Figure 26. ^{13}C NMR spectrum of compound **1j** in $CDCl_3$

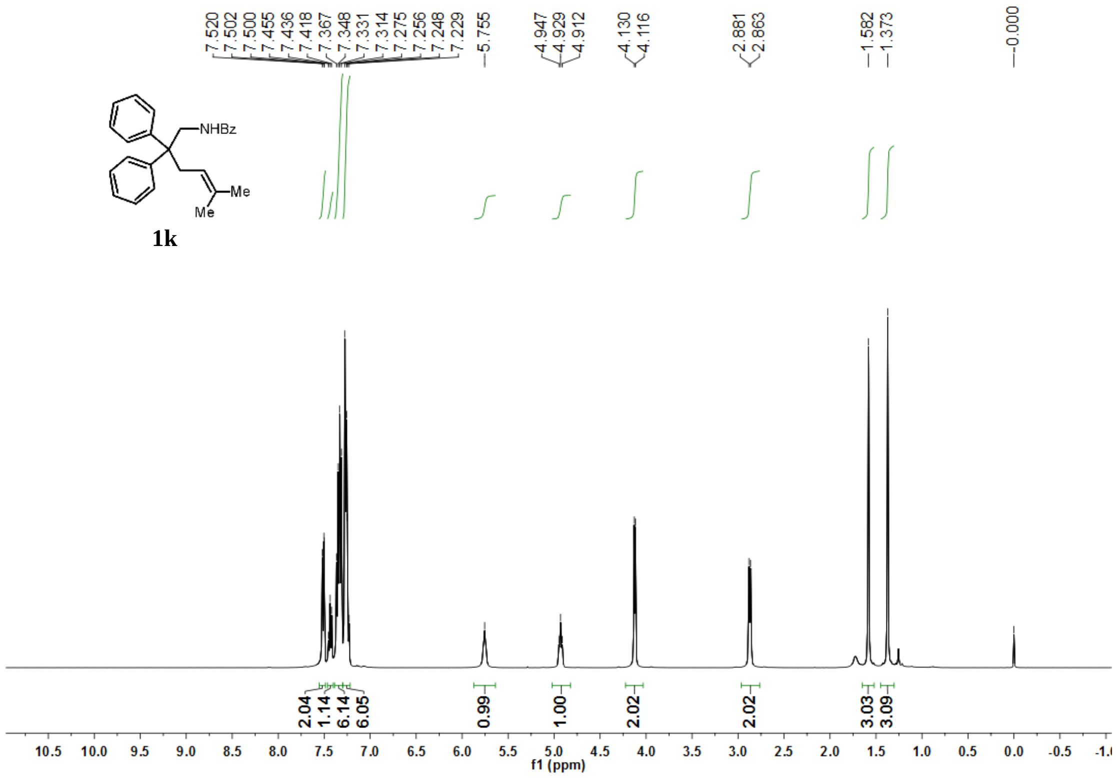

Supplementary Figure 27. ^{1}H NMR spectrum of compound **1k** in $CDCl_3$

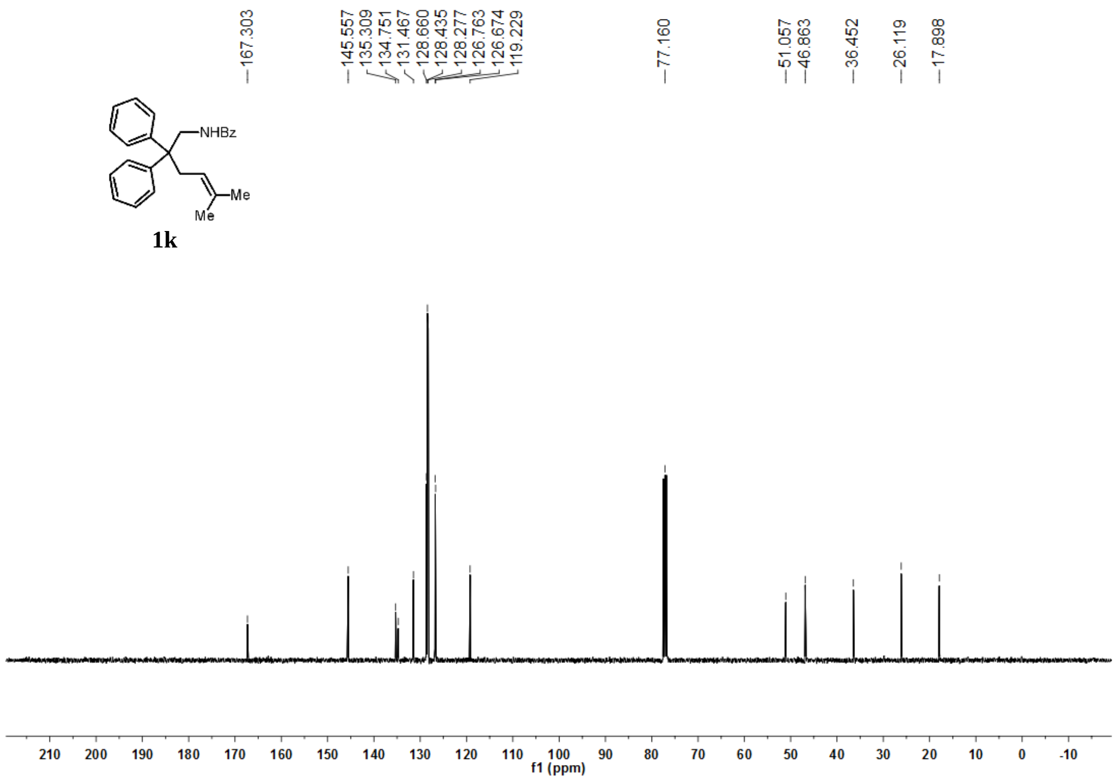

Supplementary Figure 28. ^{13}C NMR spectrum of compound **1k** in $CDCl_3$

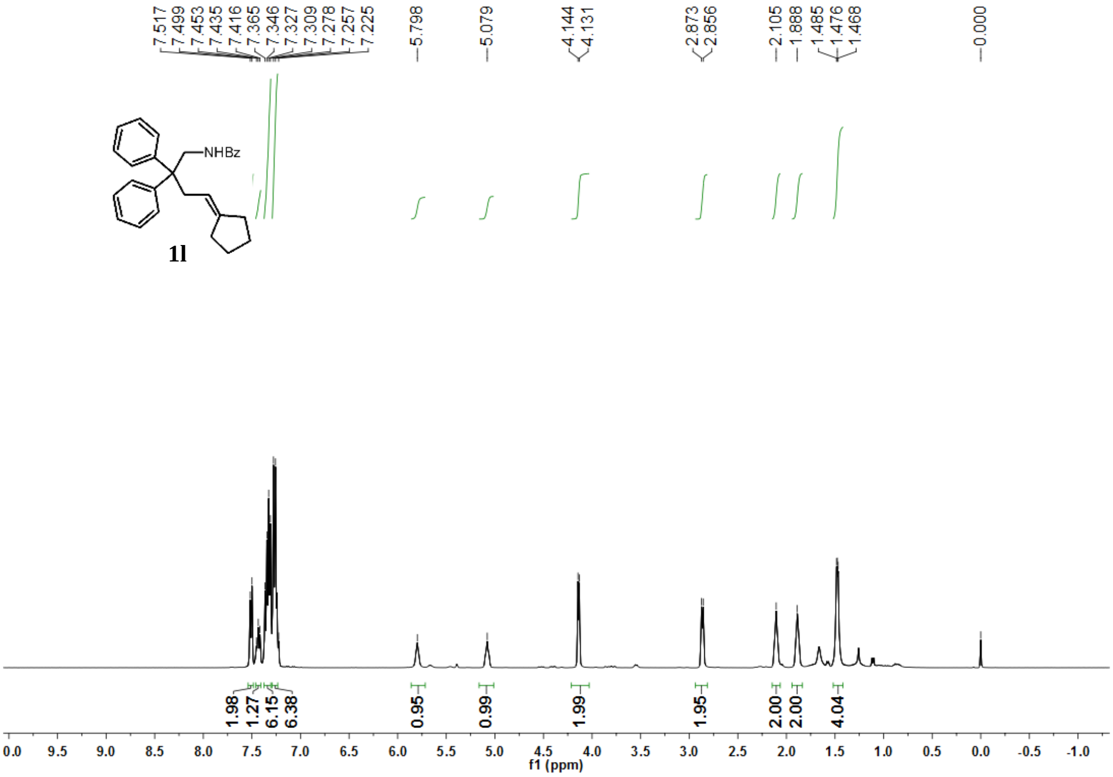

Supplementary Figure 29. ^{1}H NMR spectrum of compound **1l** in $CDCl_3$

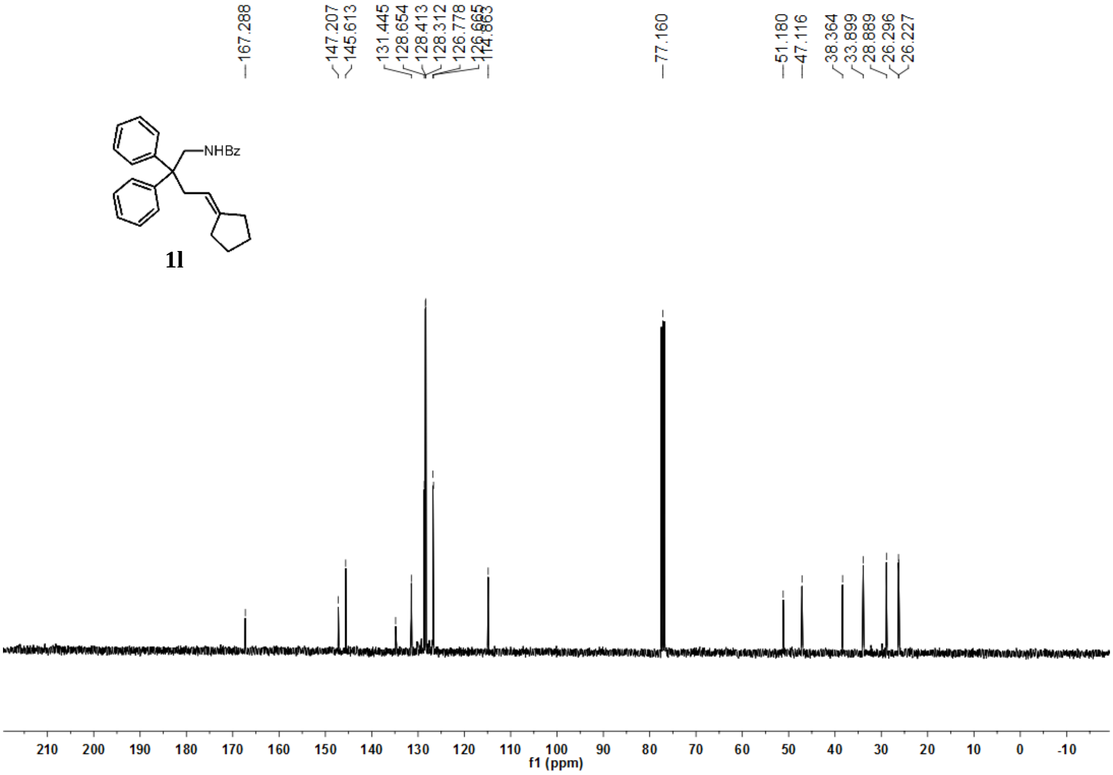

Supplementary Figure 30. ^{13}C NMR spectrum of compound **1l** in $CDCl_3$

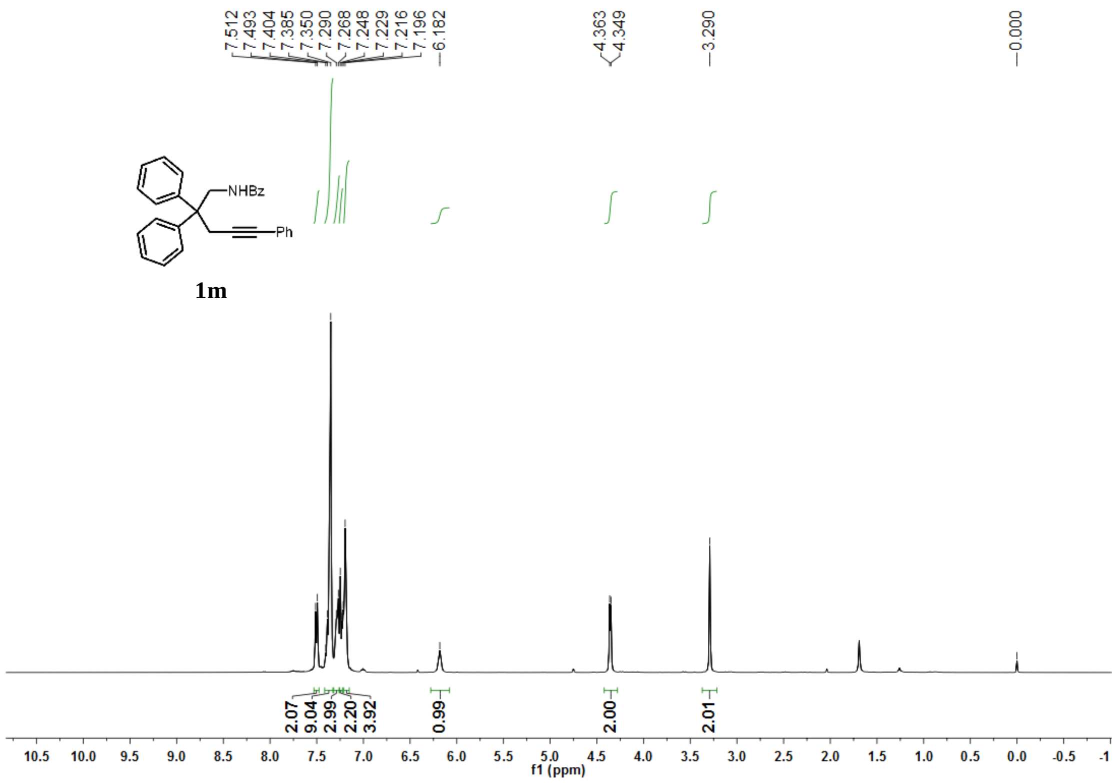

Supplementary Figure 31. ^{1}H NMR spectrum of compound **1m** in $CDCl_3$

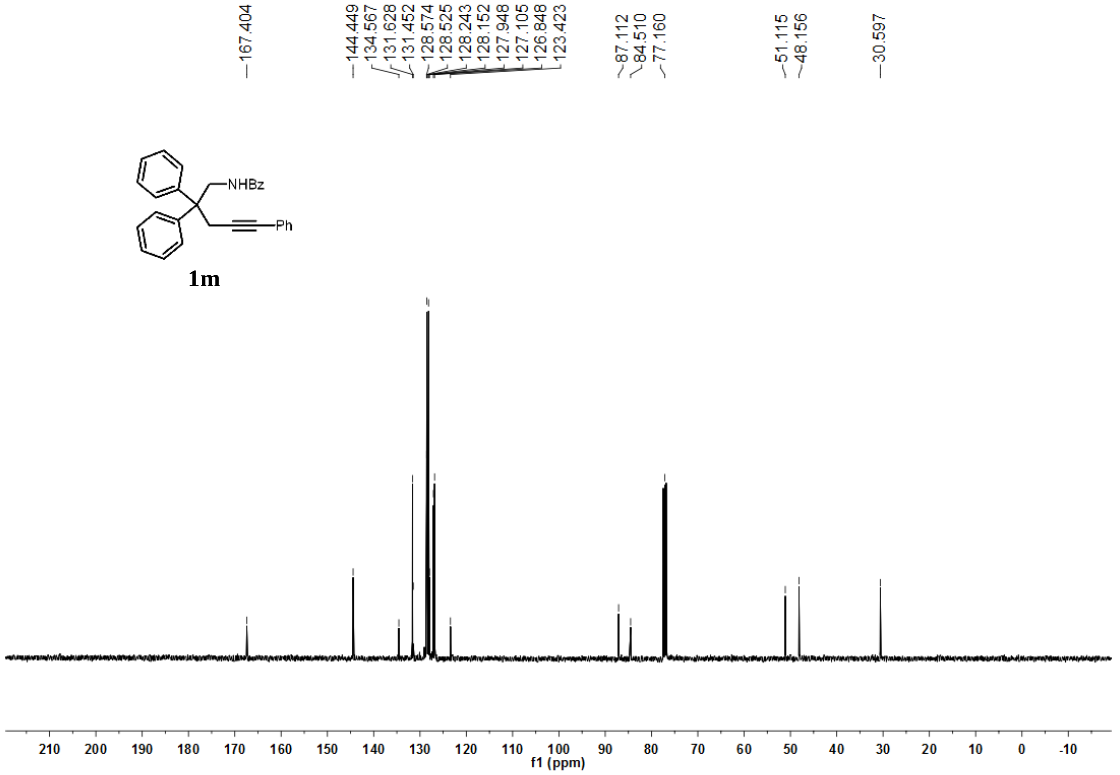

Supplementary Figure 32. ^{13}C NMR spectrum of compound **1m** in $CDCl_3$

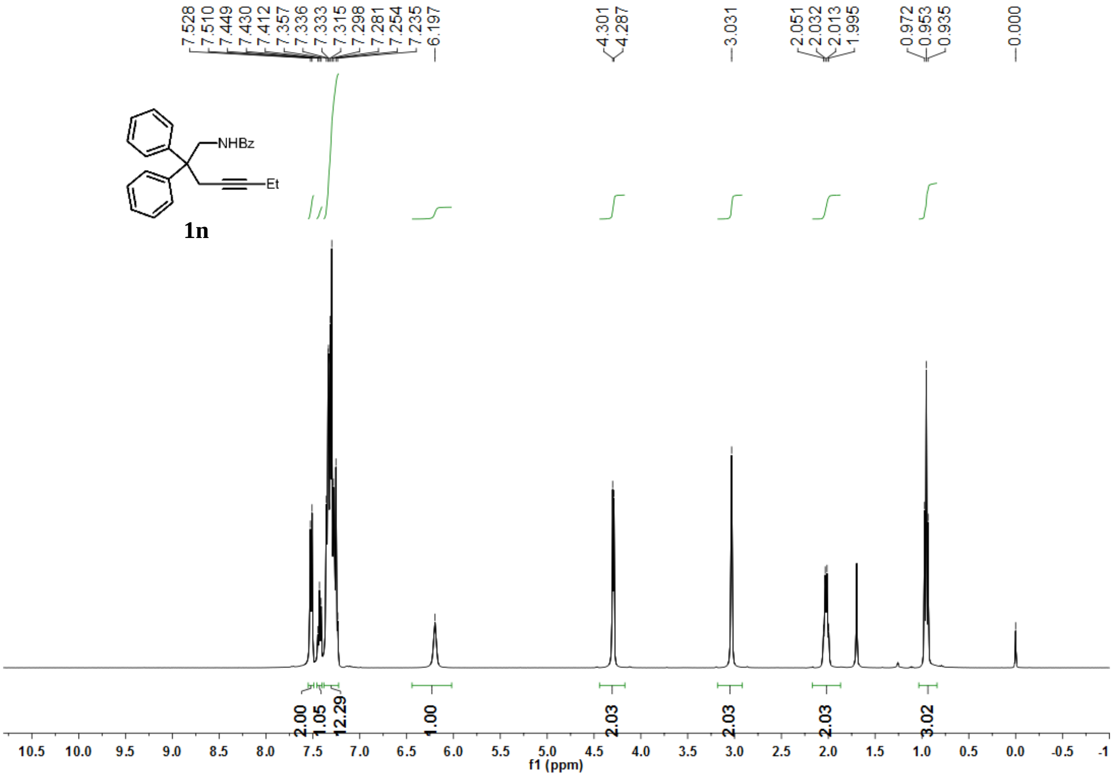

Supplementary Figure 33. ^{1}H NMR spectrum of compound **1n** in $CDCl_3$

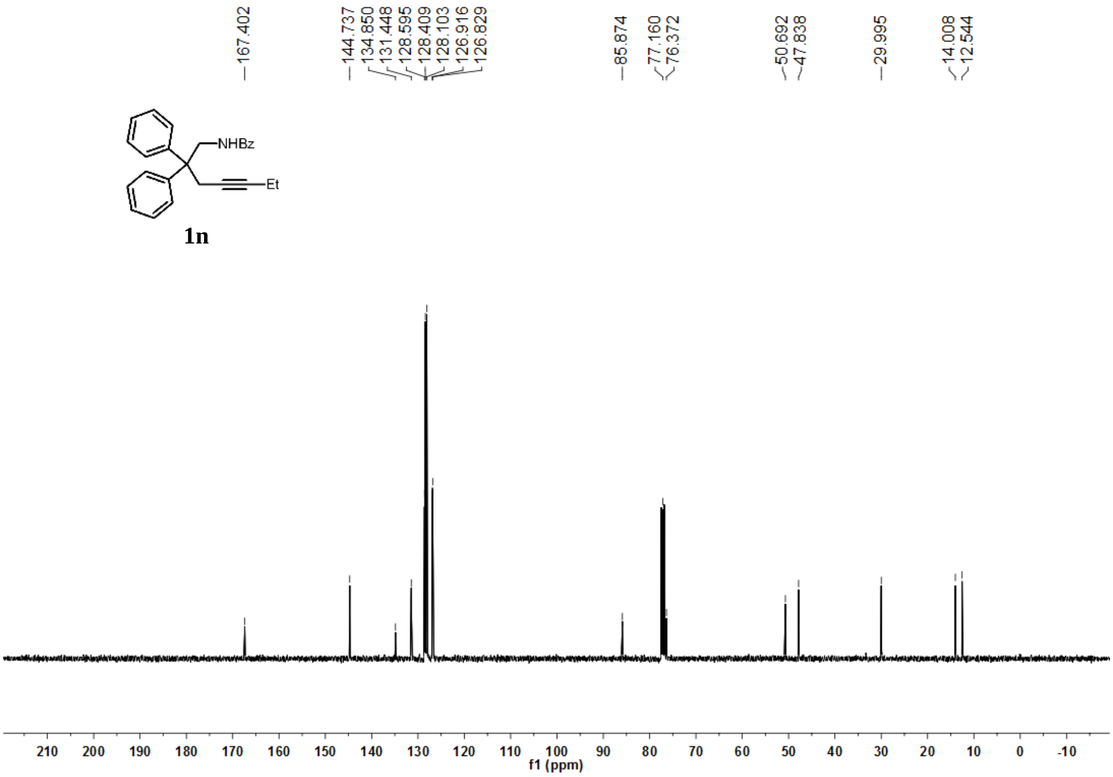

Supplementary Figure 34. ^{13}C NMR spectrum of compound **1n** in $CDCl_3$

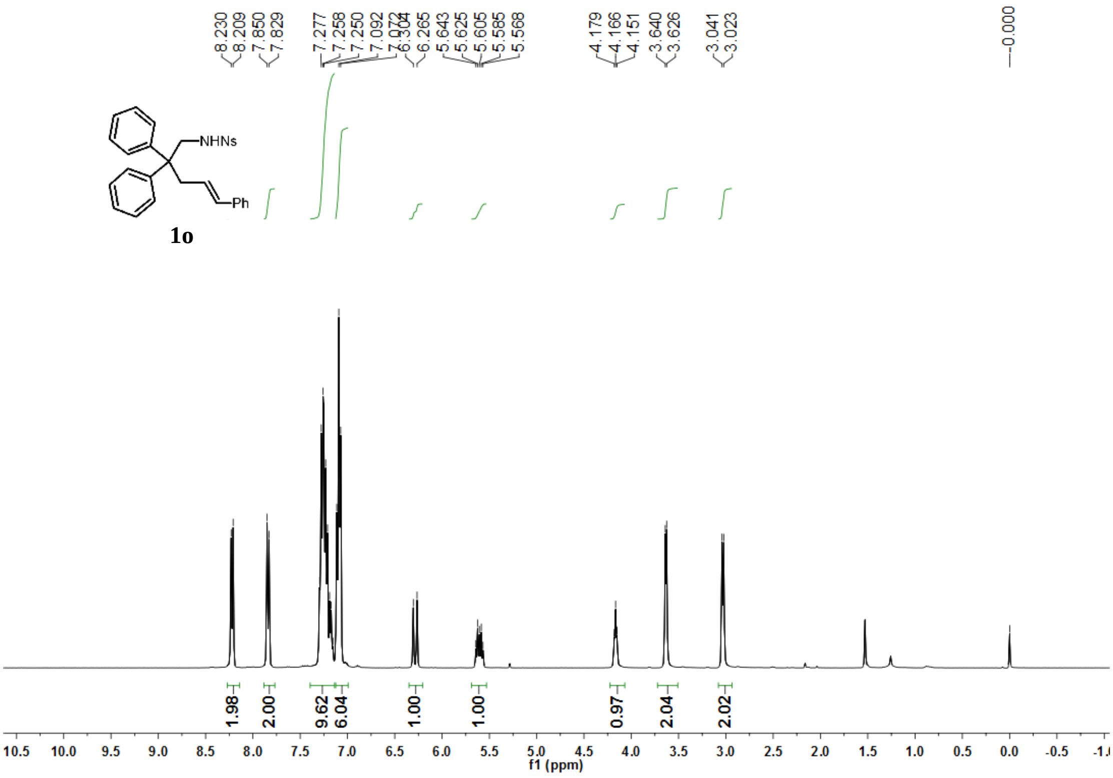

Supplementary Figure 35. ^{1}H NMR spectrum of compound **1o** in $CDCl_3$

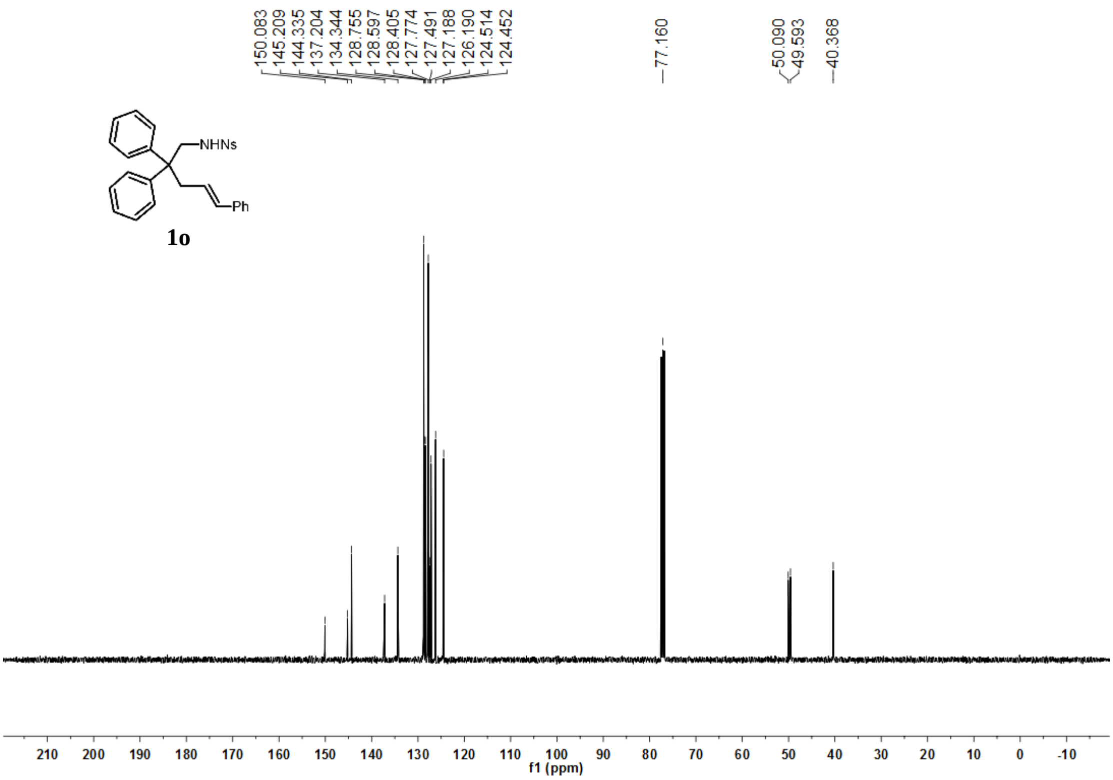

Supplementary Figure 36. ^{13}C NMR spectrum of compound **1o** in $CDCl_3$

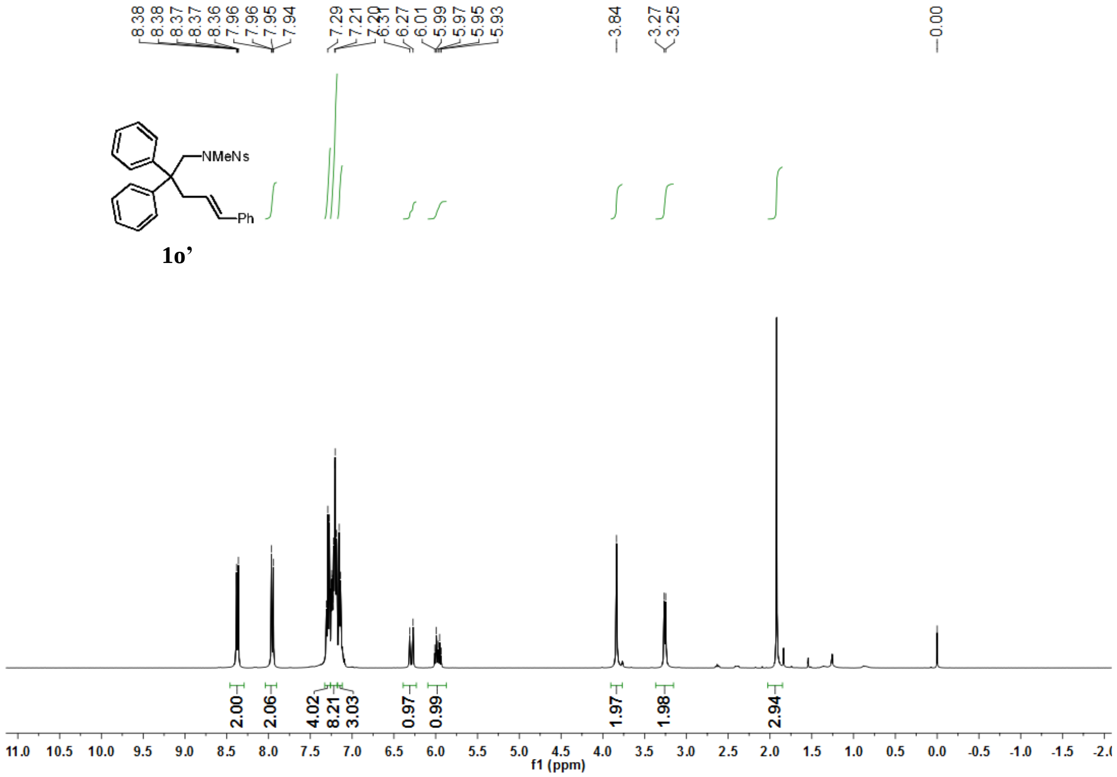

Supplementary Figure 37. ^{1}H NMR spectrum of compound **1o'** in $CDCl_3$

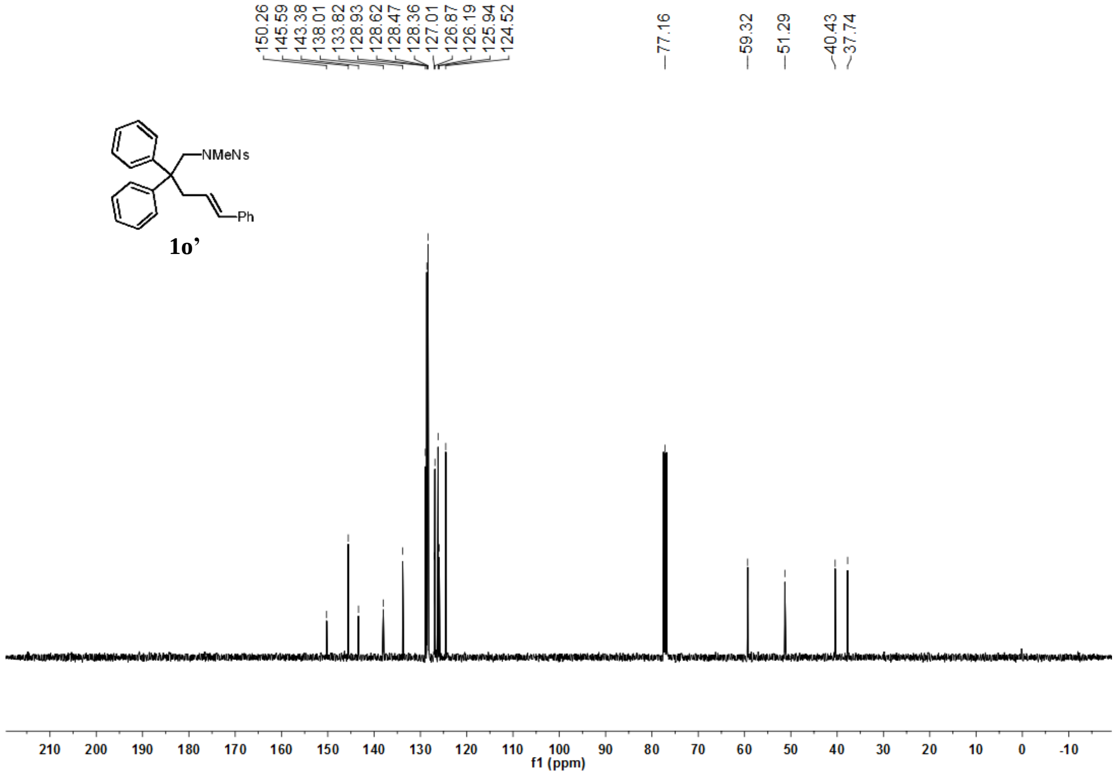

Supplementary Figure 38. ^{13}C NMR spectrum of compound **1o'** in $CDCl_3$

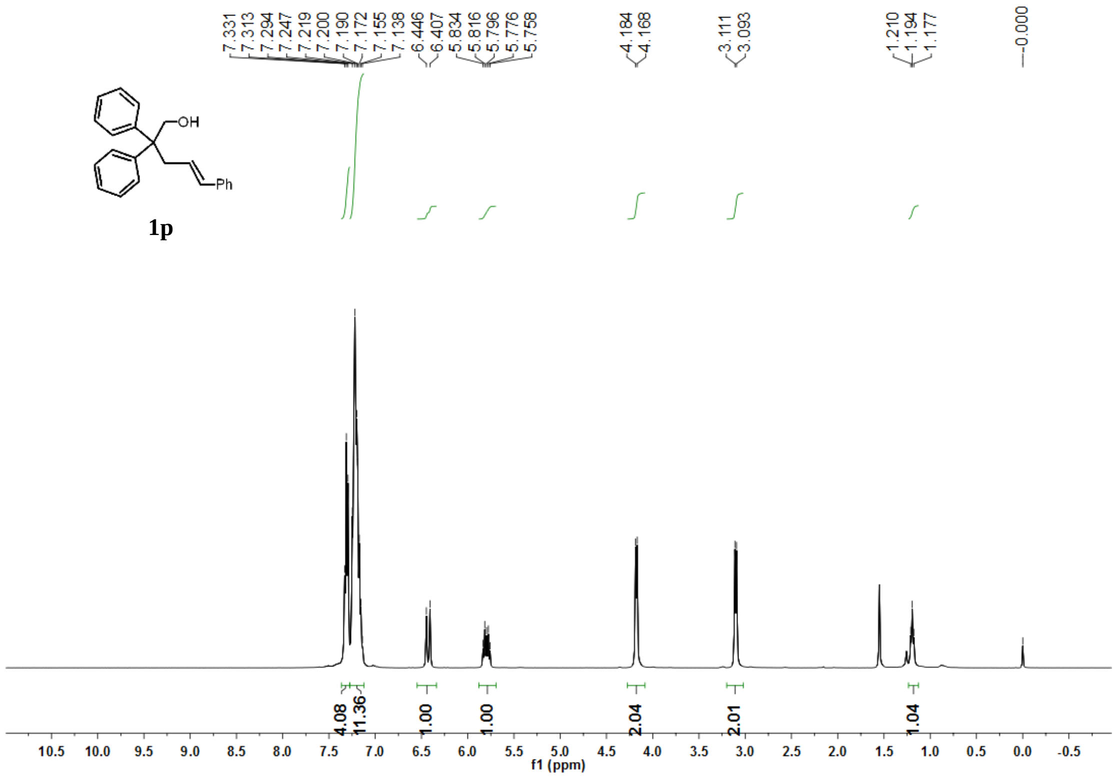

Supplementary Figure 39. ^{1}H NMR spectrum of compound **1p** in $CDCl_3$

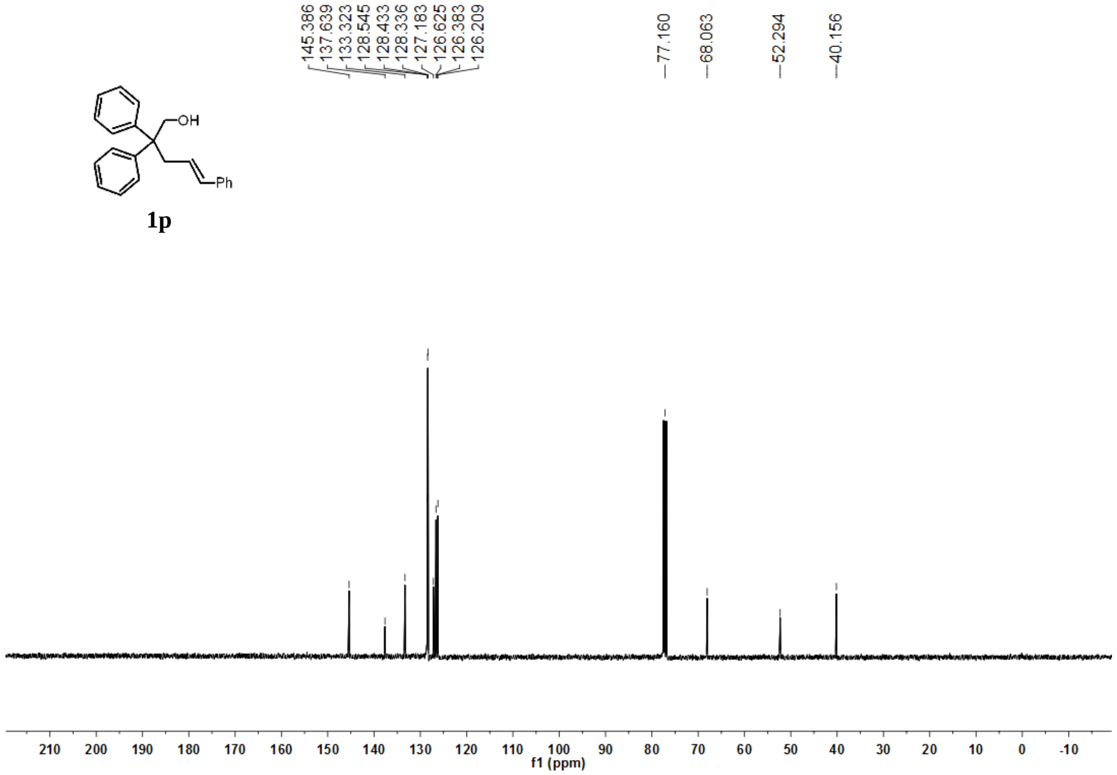

Supplementary Figure 40. ^{13}C NMR spectrum of compound **1p** in $CDCl_3$

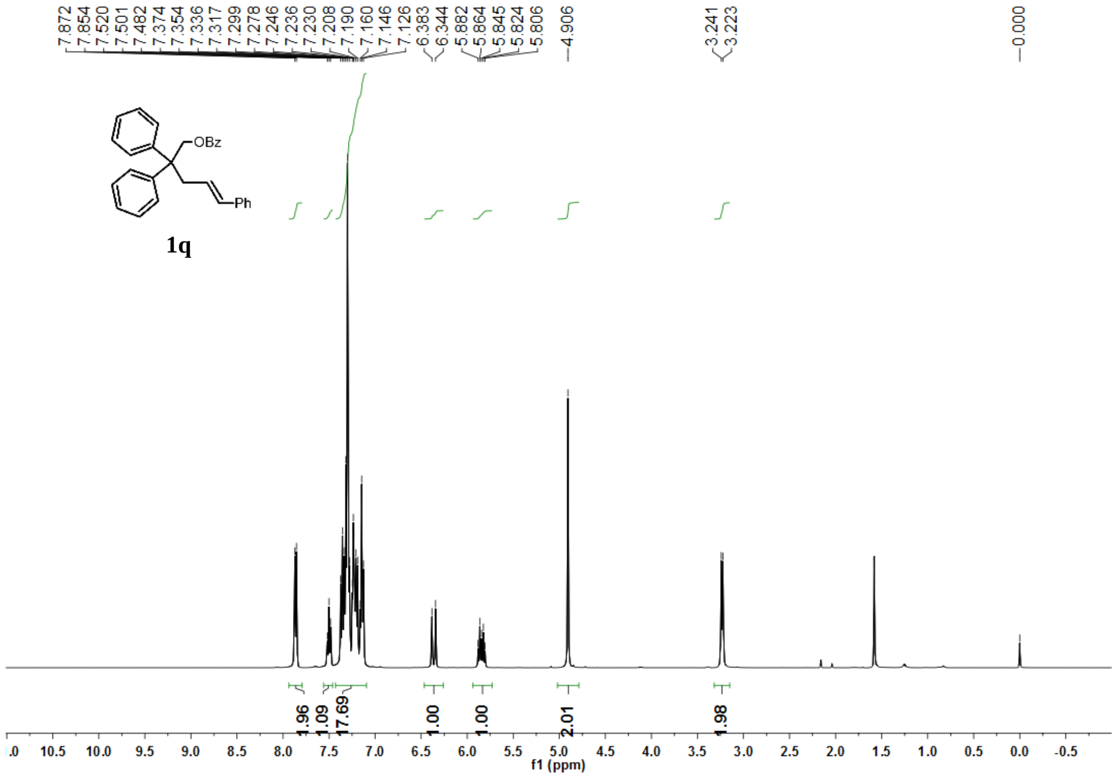

Supplementary Figure 41. ^{1}H NMR spectrum of compound **1q** in $CDCl_3$

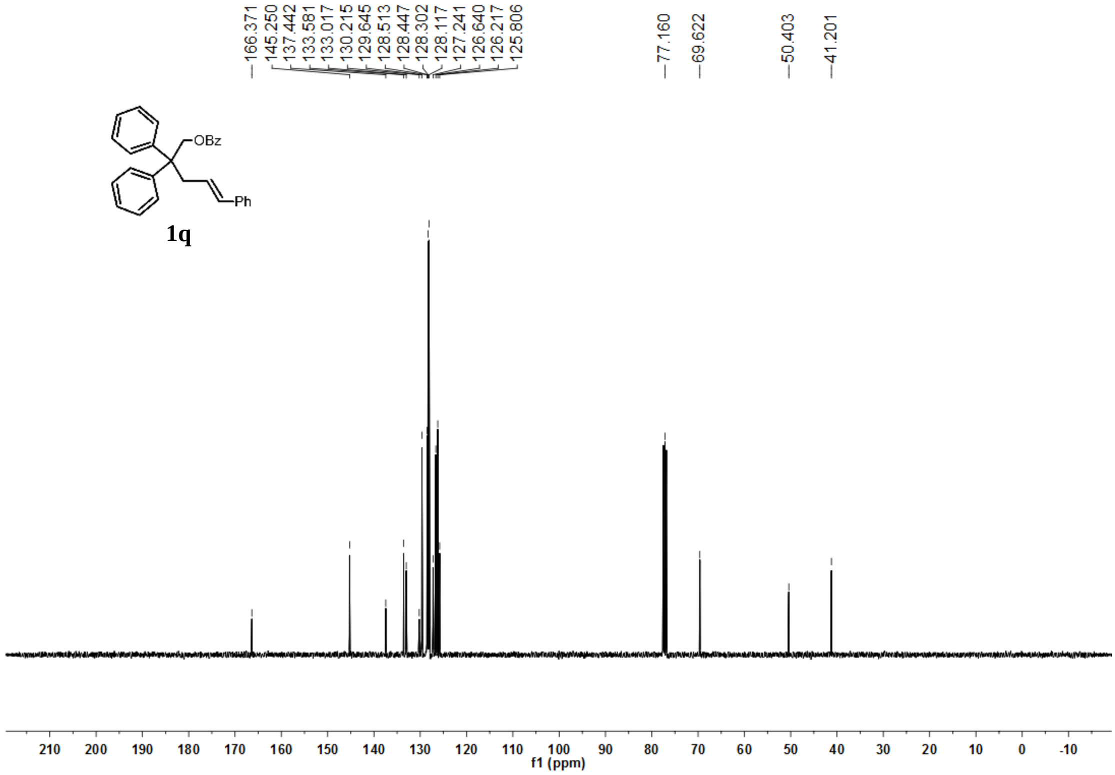

Supplementary Figure 42. ^{13}C NMR spectrum of compound **1q** in $CDCl_3$

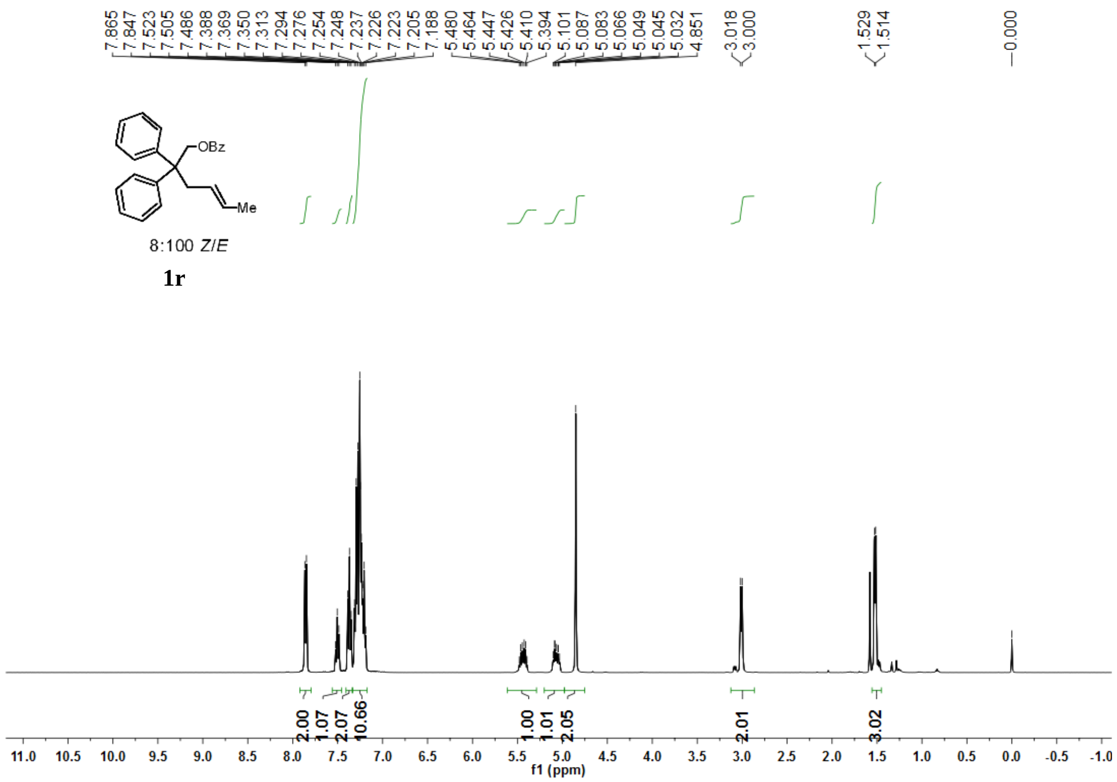

Supplementary Figure 43. ^{1}H NMR spectrum of compound **1r** in $CDCl_3$

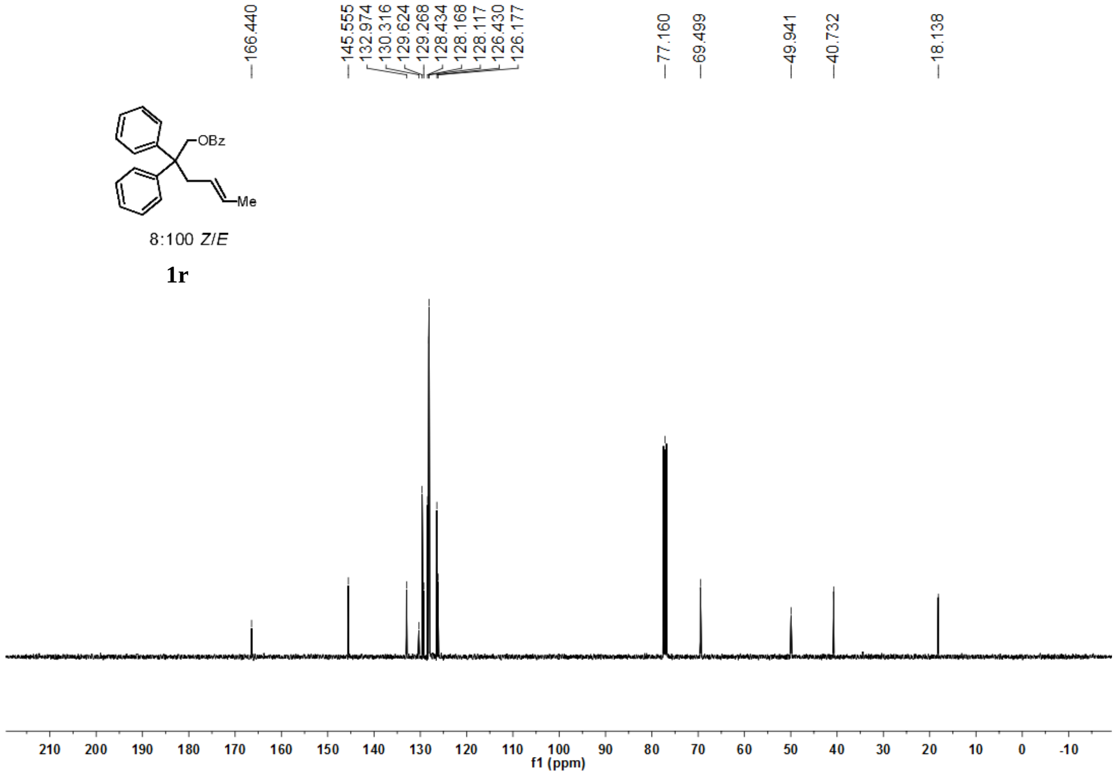

Supplementary Figure 44. ^{13}C NMR spectrum of compound **1r** in $CDCl_3$

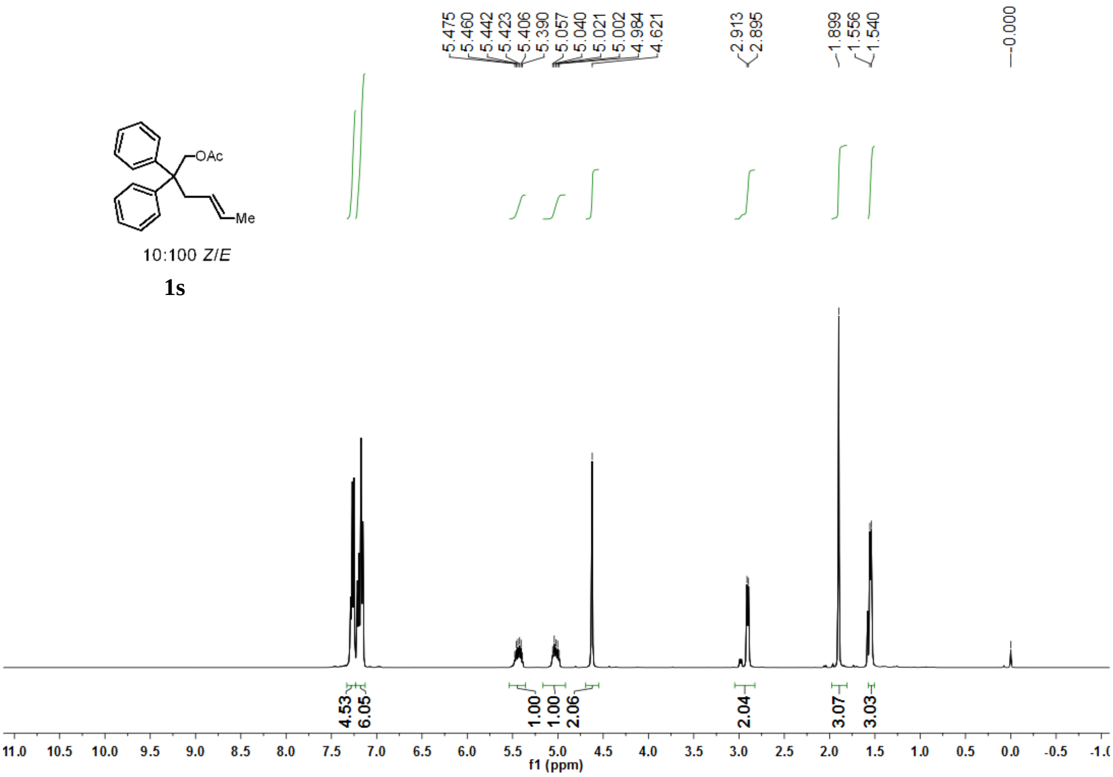

Supplementary Figure 45. ^{1}H NMR spectrum of compound **1s** in $CDCl_3$

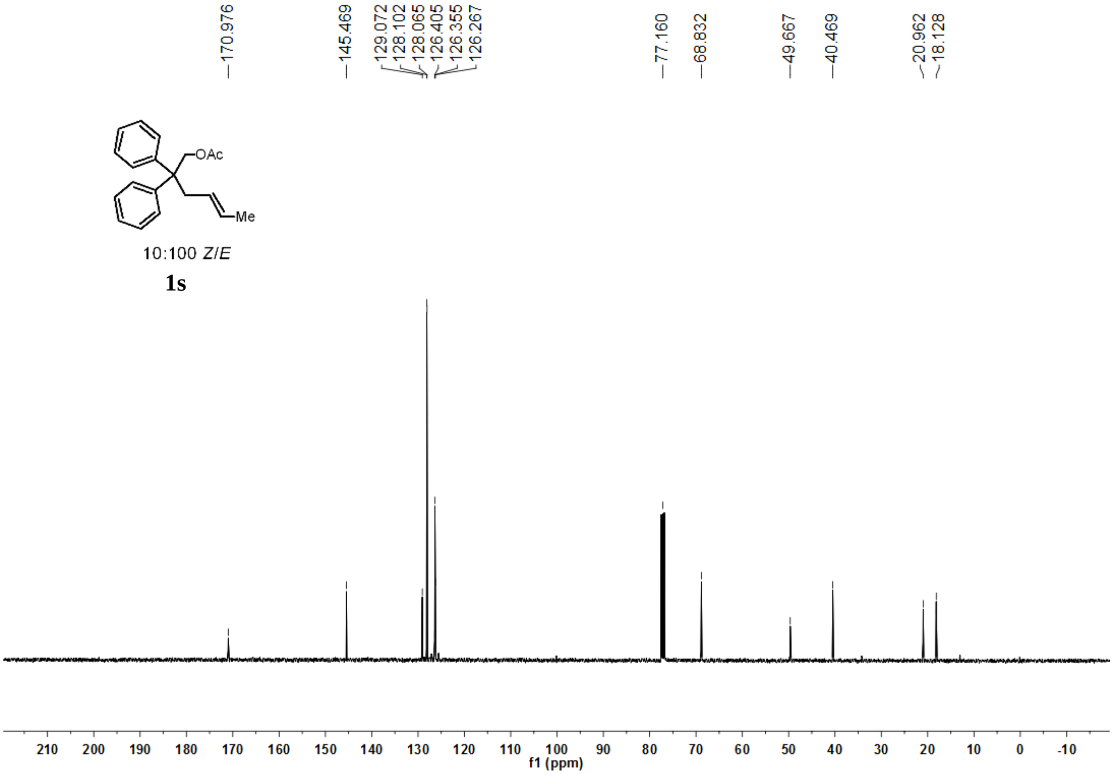

Supplementary Figure 46. ^{13}C NMR spectrum of compound **1s** in $CDCl_3$

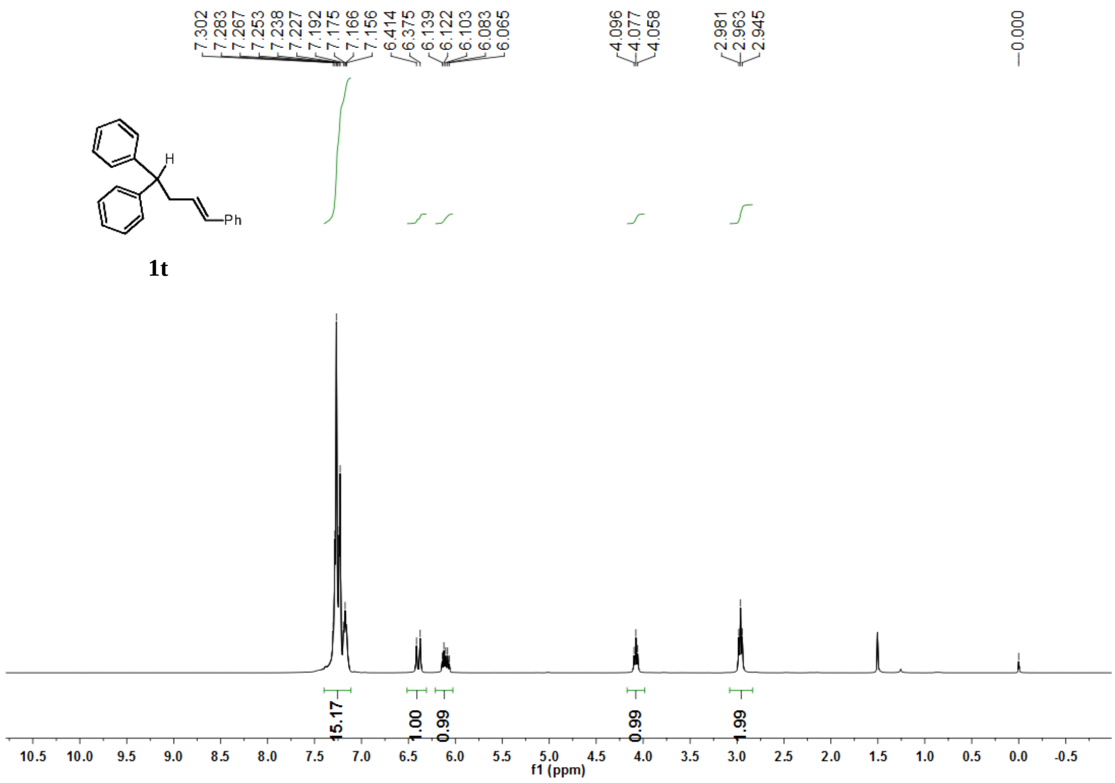

Supplementary Figure 47. ^{1}H NMR spectra of compound **1t** in $CDCl_3$

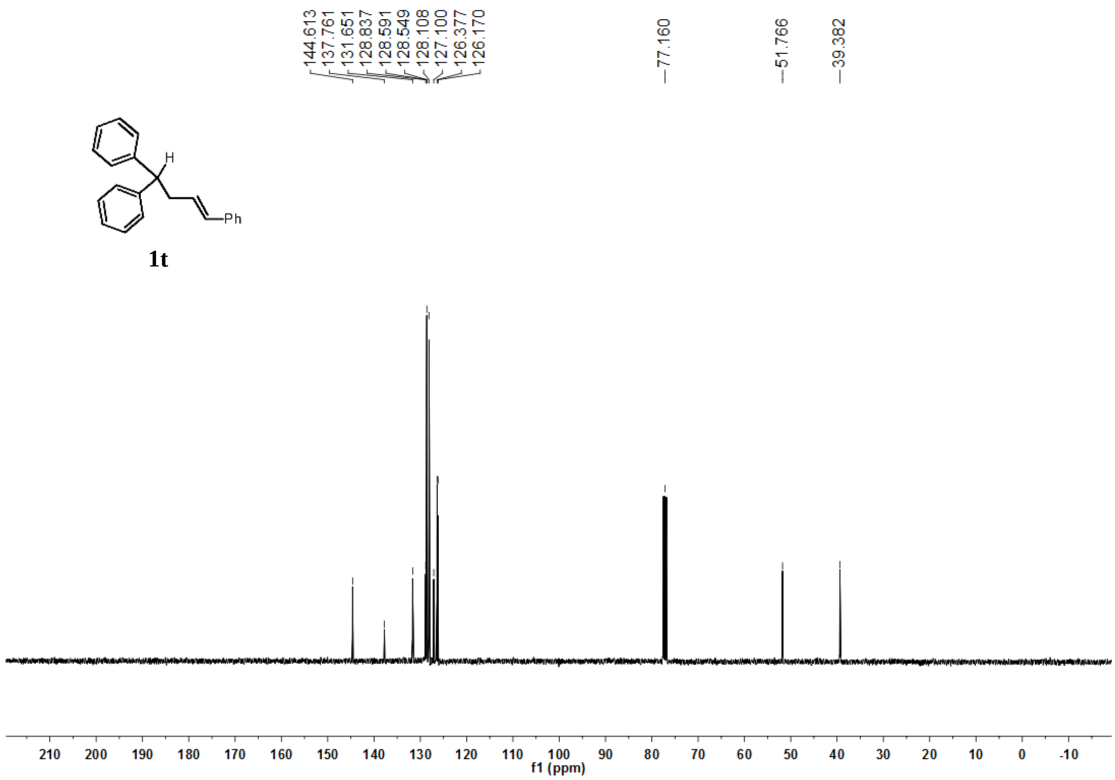

Supplementary Figure 48. ^{13}C NMR spectra of compound **1t** in $CDCl_3$

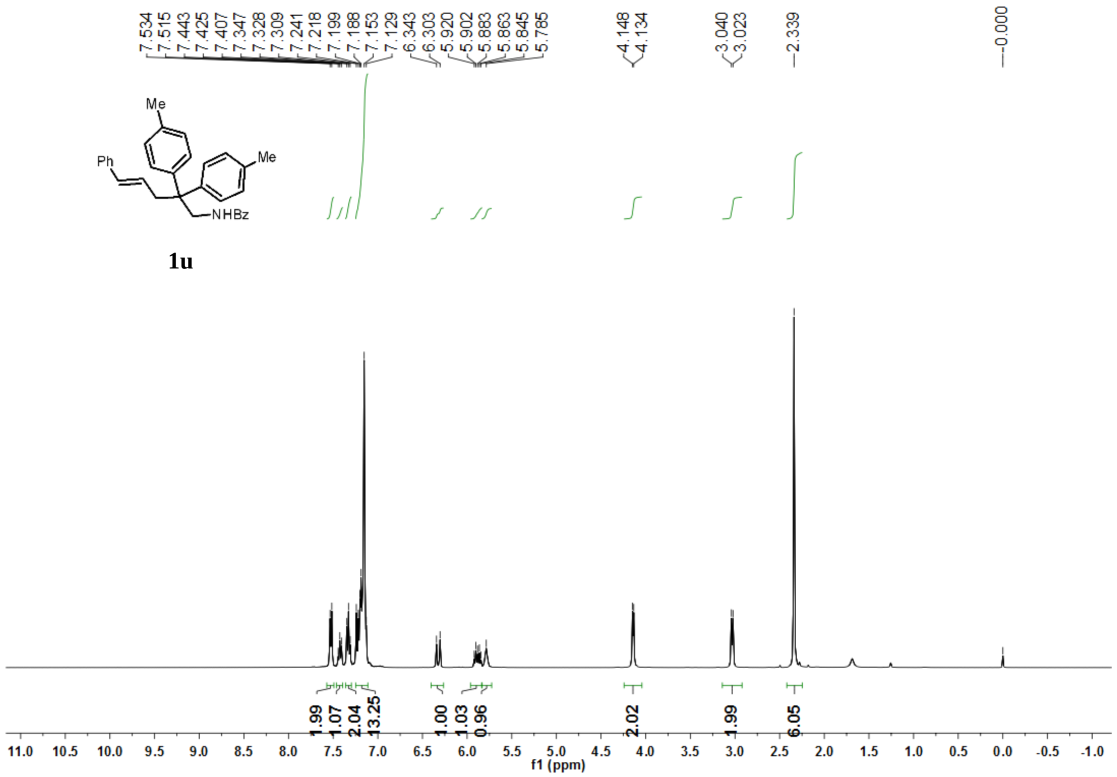

Supplementary Figure 49. ^{1}H NMR spectrum of compound **1u** in $CDCl_3$

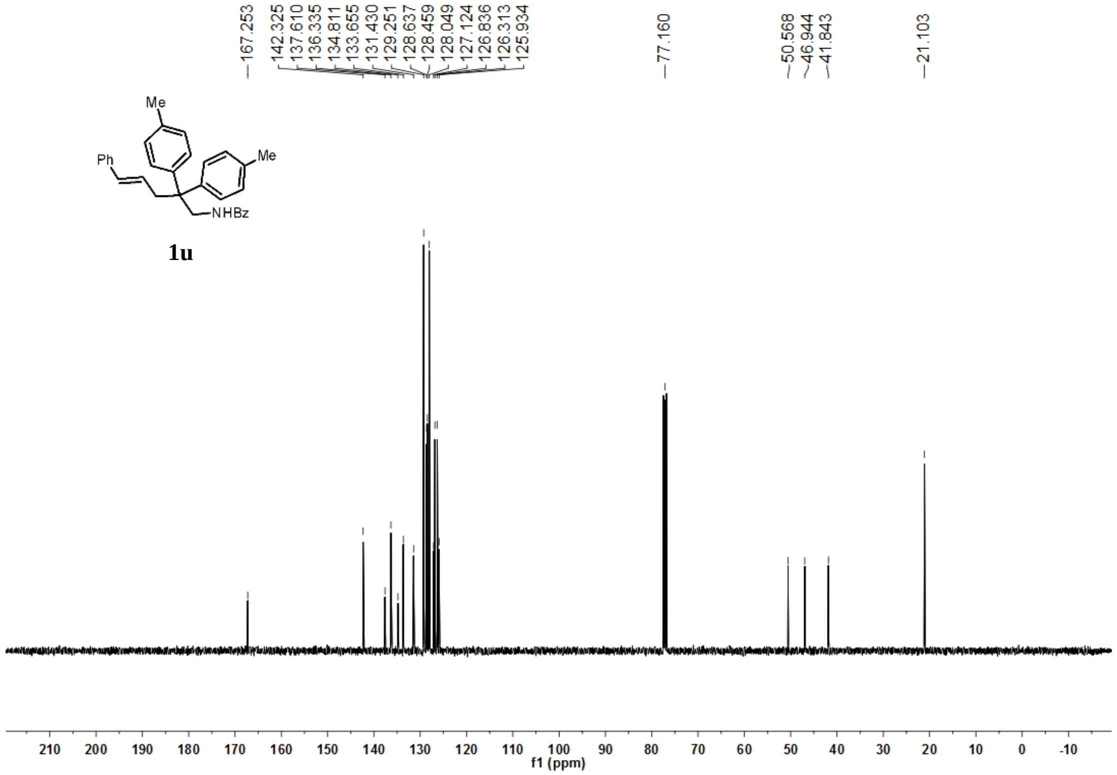

Supplementary Figure 50. ^{13}C NMR spectrum of compound **1u** in $CDCl_3$

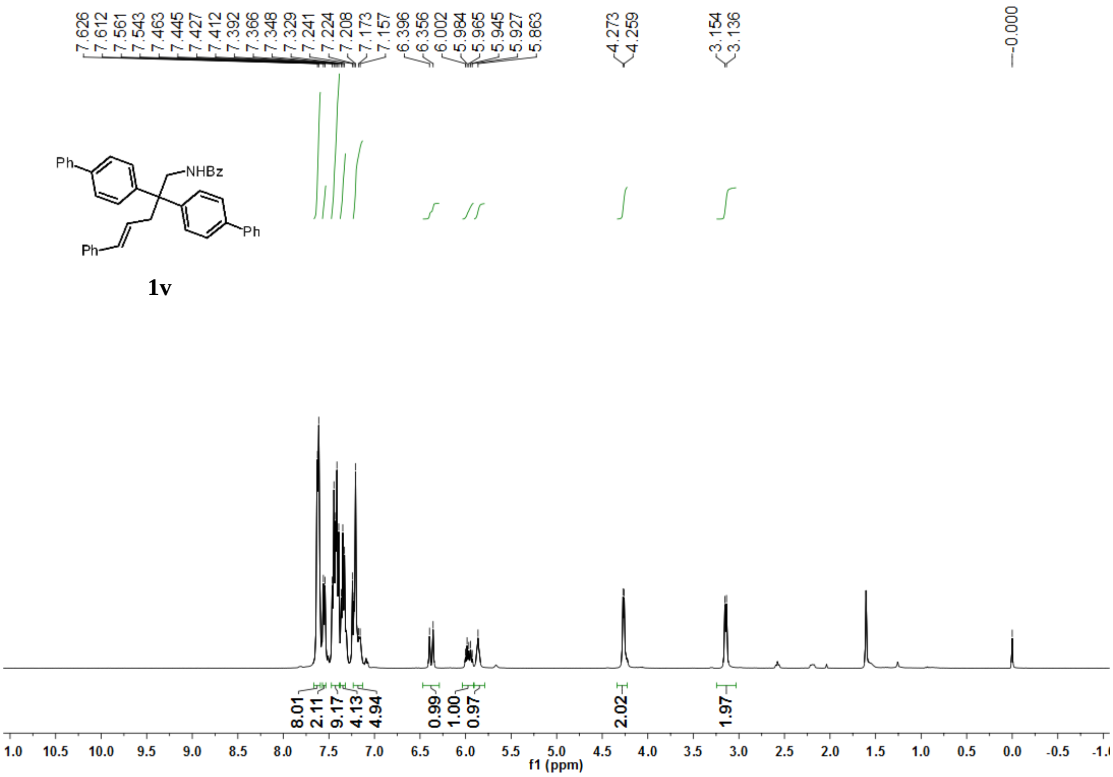

Supplementary Figure 51. ^{1}H NMR spectrum of compound **1v** in $CDCl_3$

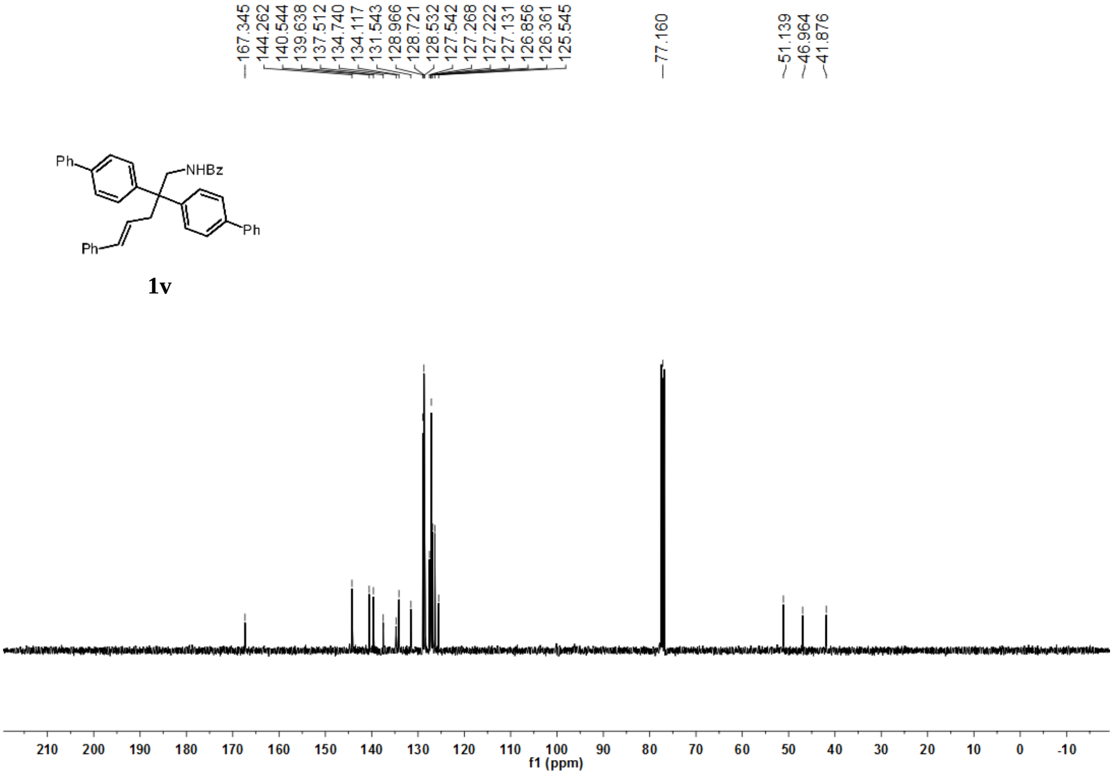

Supplementary Figure 52. ^{13}C NMR spectrum of compound **1v** in $CDCl_3$

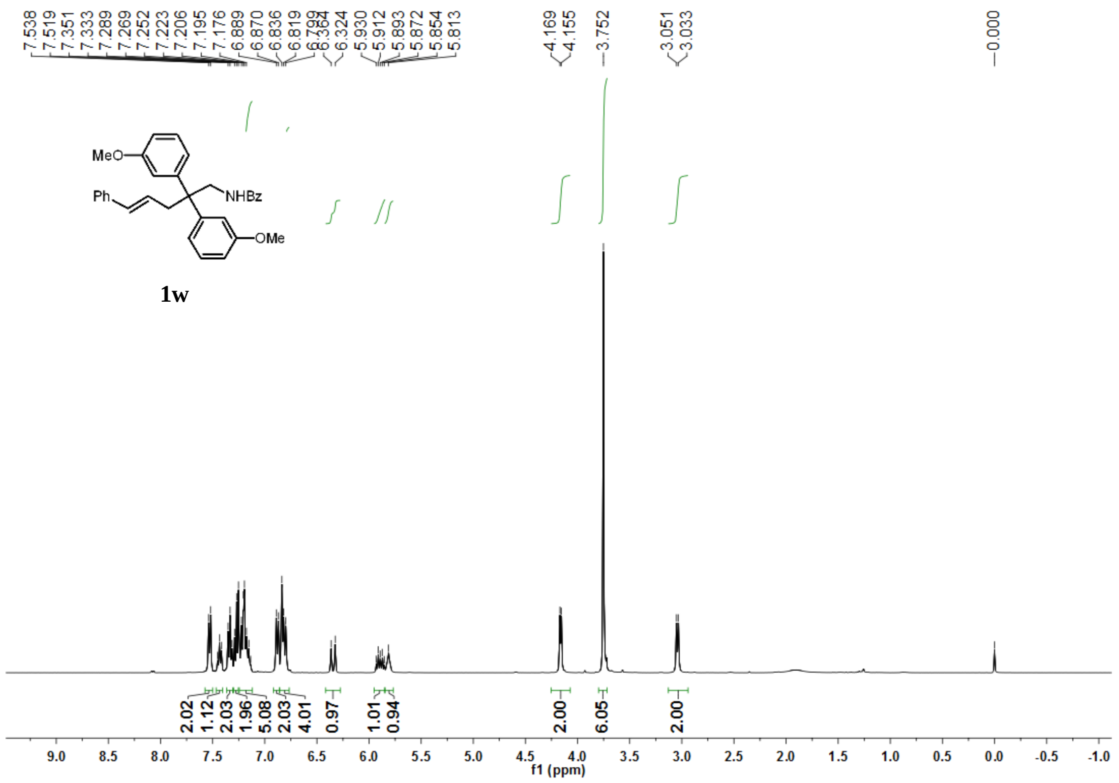

Supplementary Figure 53. ^{1}H NMR spectrum of compound **1w** in $CDCl_3$

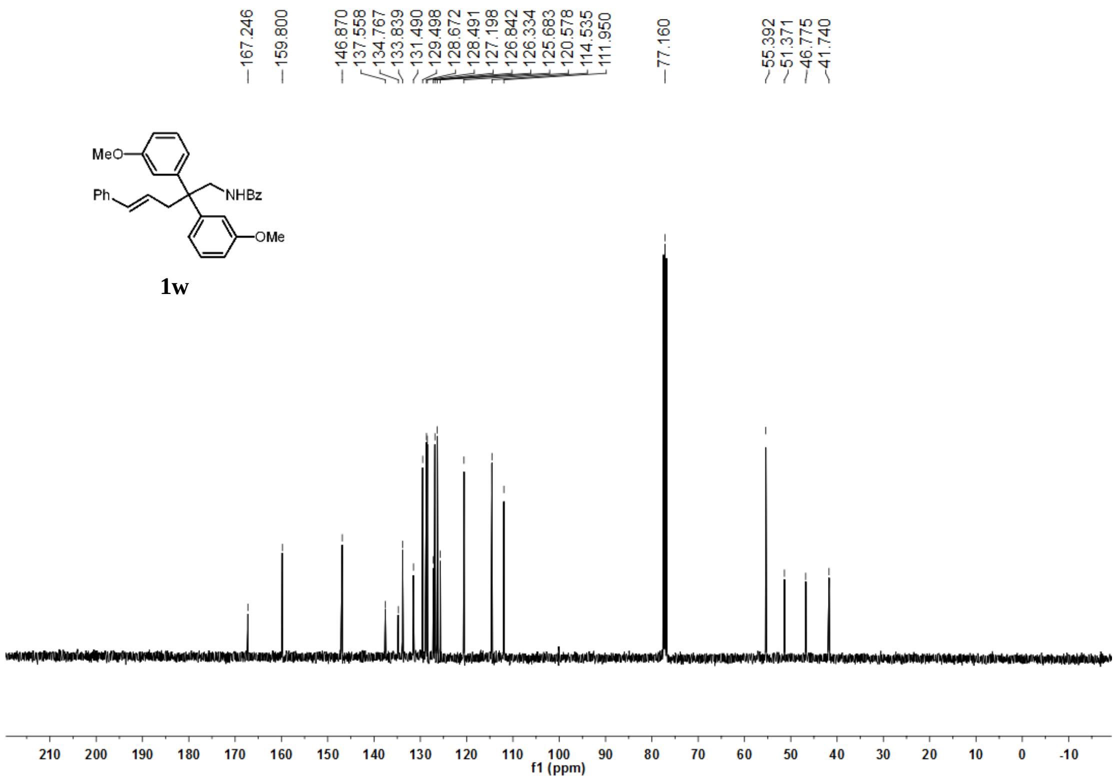

Supplementary Figure 54. ^{13}C NMR spectrum of compound **1w** in $CDCl_3$

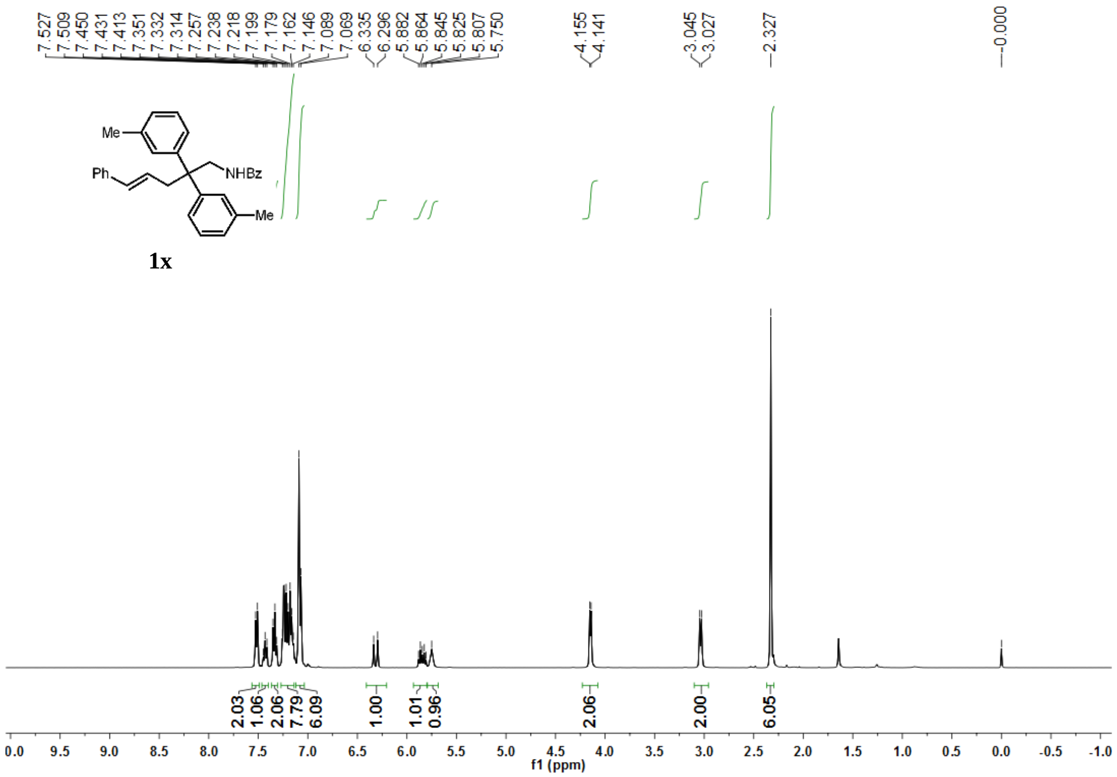

Supplementary Figure 55. ^{1}H NMR spectrum of compound **1x** in $CDCl_3$

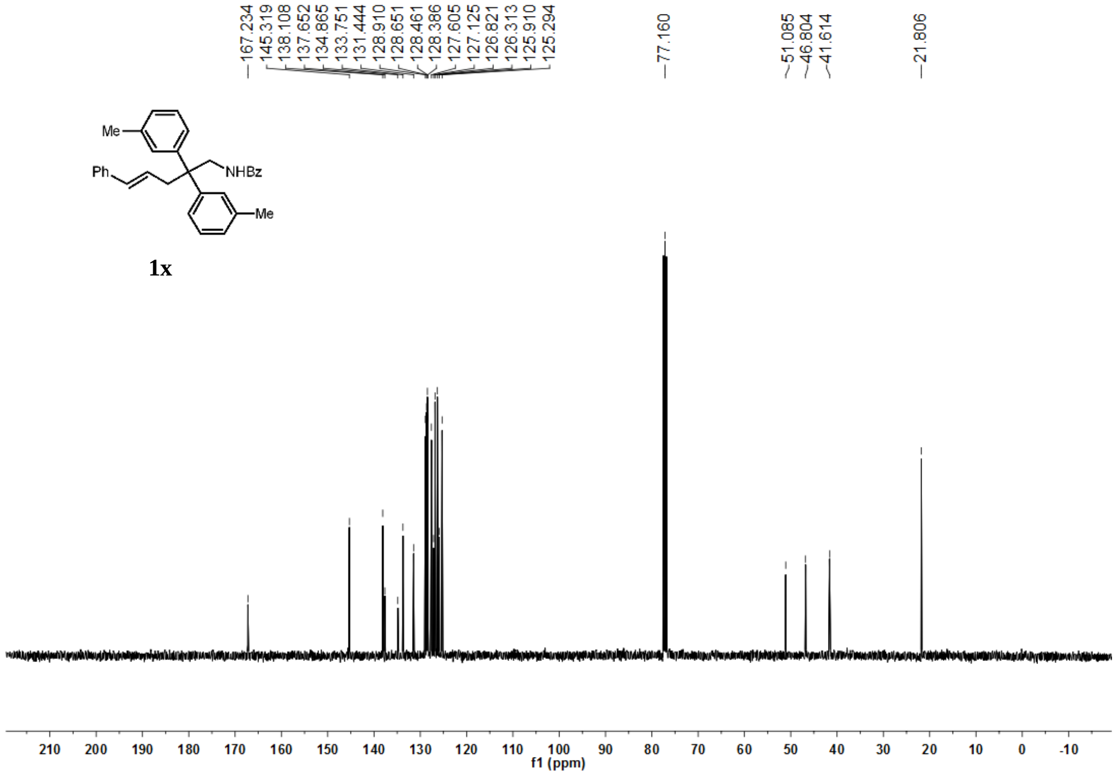

Supplementary Figure 56. ^{13}C NMR spectrum of compound **1x** in $CDCl_3$

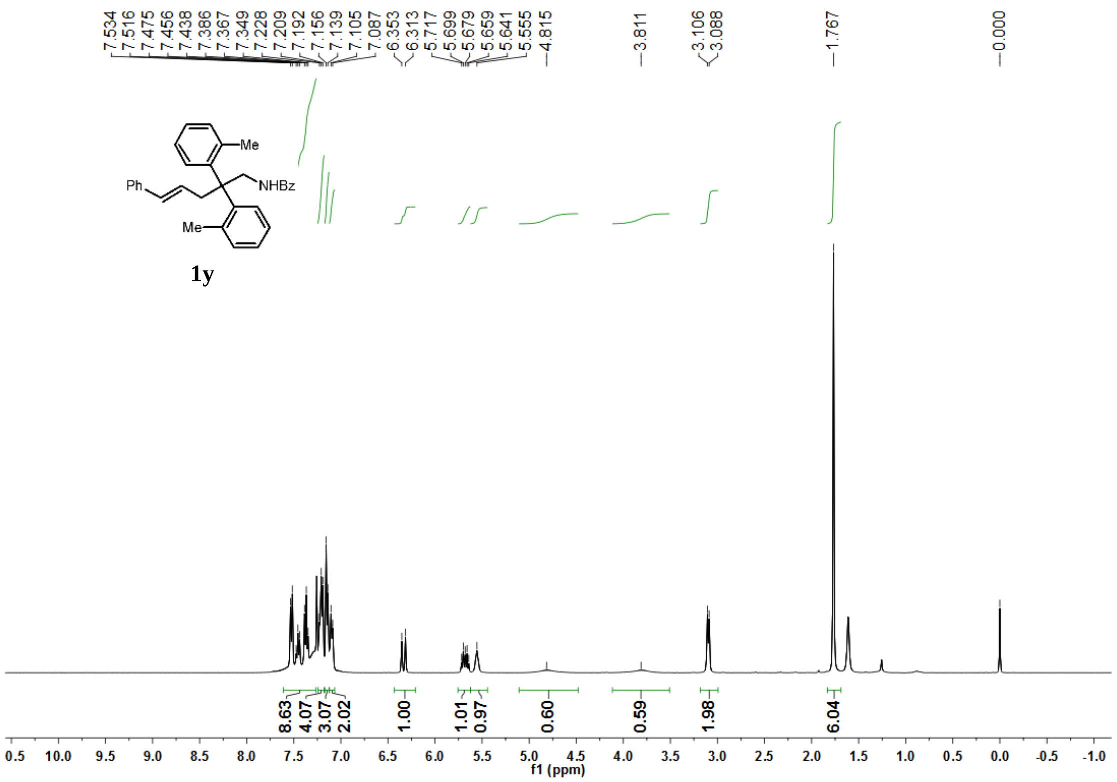

Supplementary Figure 57. ^{1}H NMR spectrum of compound **1y** in $CDCl_3$

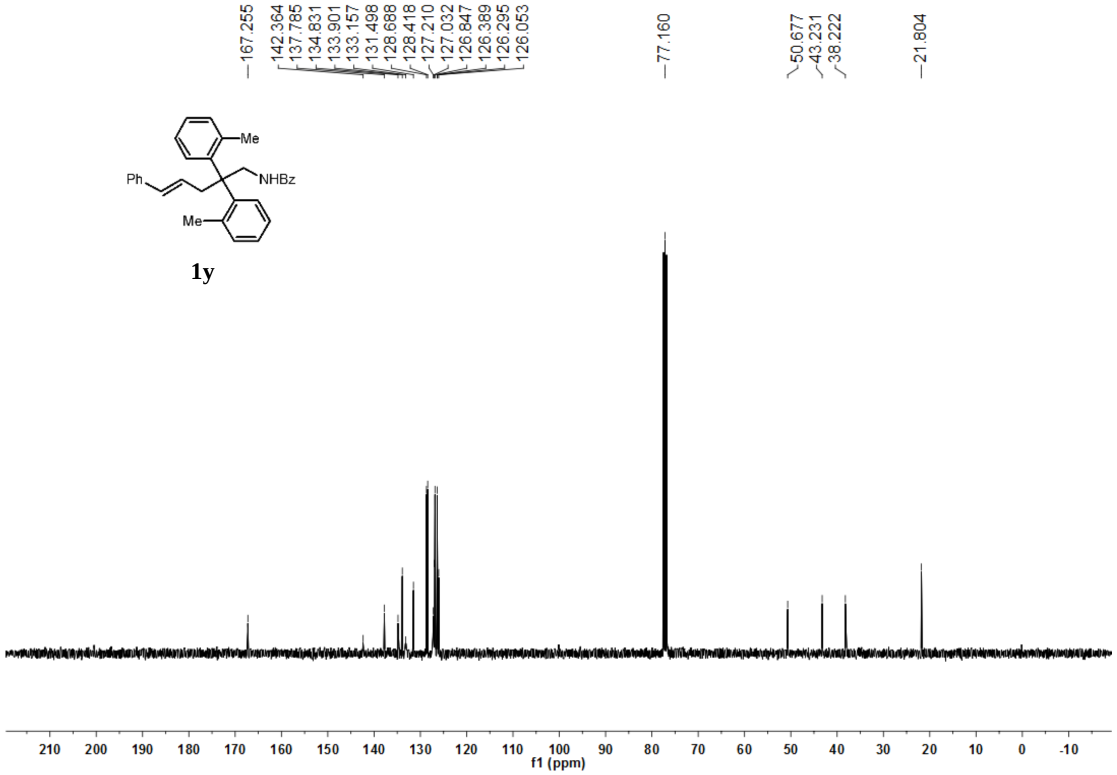

Supplementary Figure 58. ^{13}C NMR spectrum of compound **1y** in $CDCl_3$

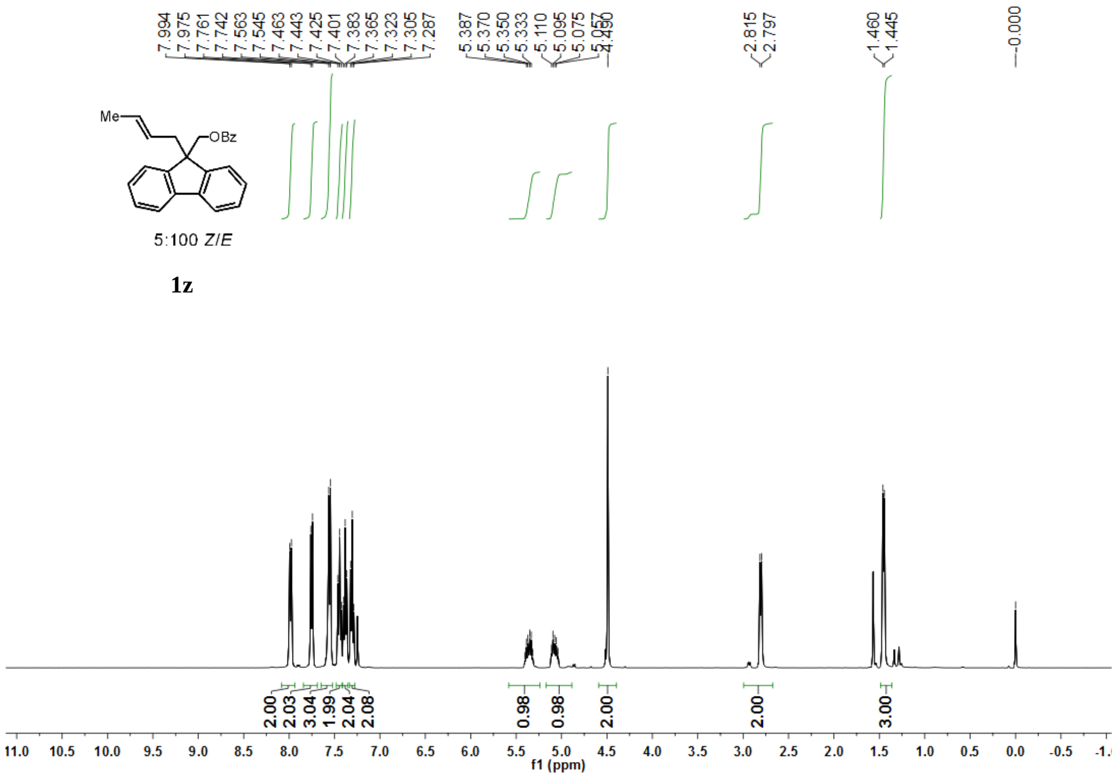

Supplementary Figure 59. ^{1}H NMR spectrum of compound **1z** in $CDCl_3$

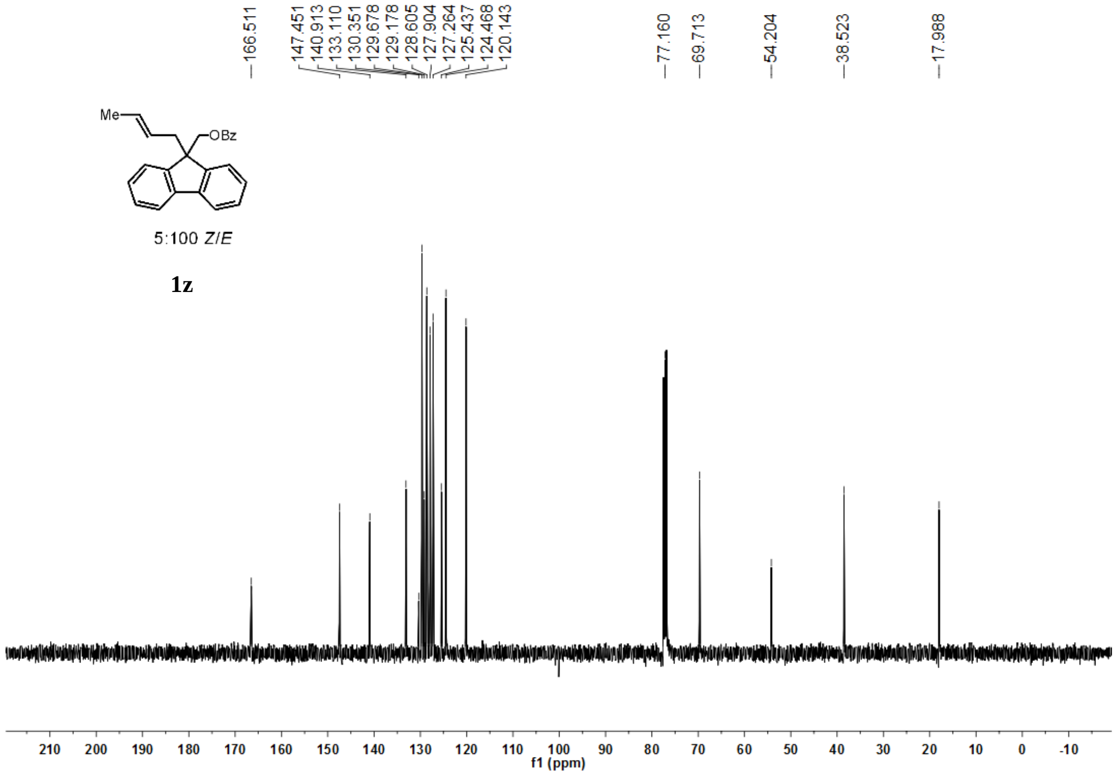

Supplementary Figure 60. ^{13}C NMR spectrum of compound **1z** in $CDCl_3$

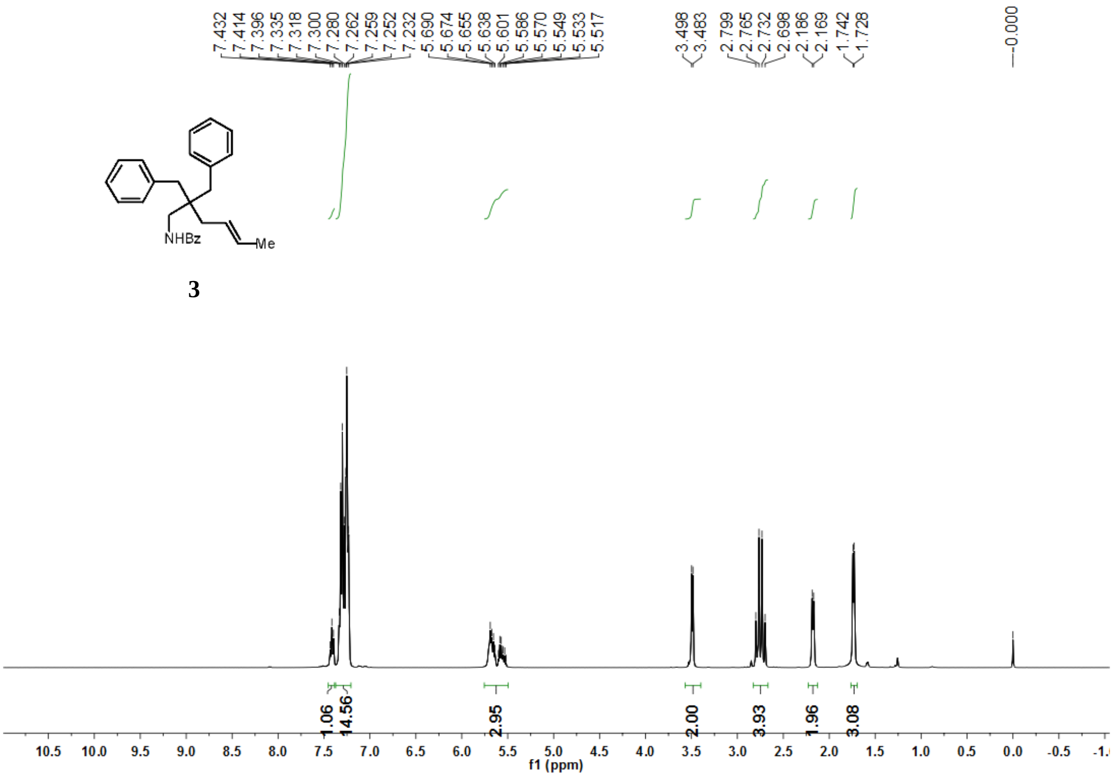

Supplementary Figure 61. ^{1}H NMR spectrum of compound **3** in $CDCl_3$

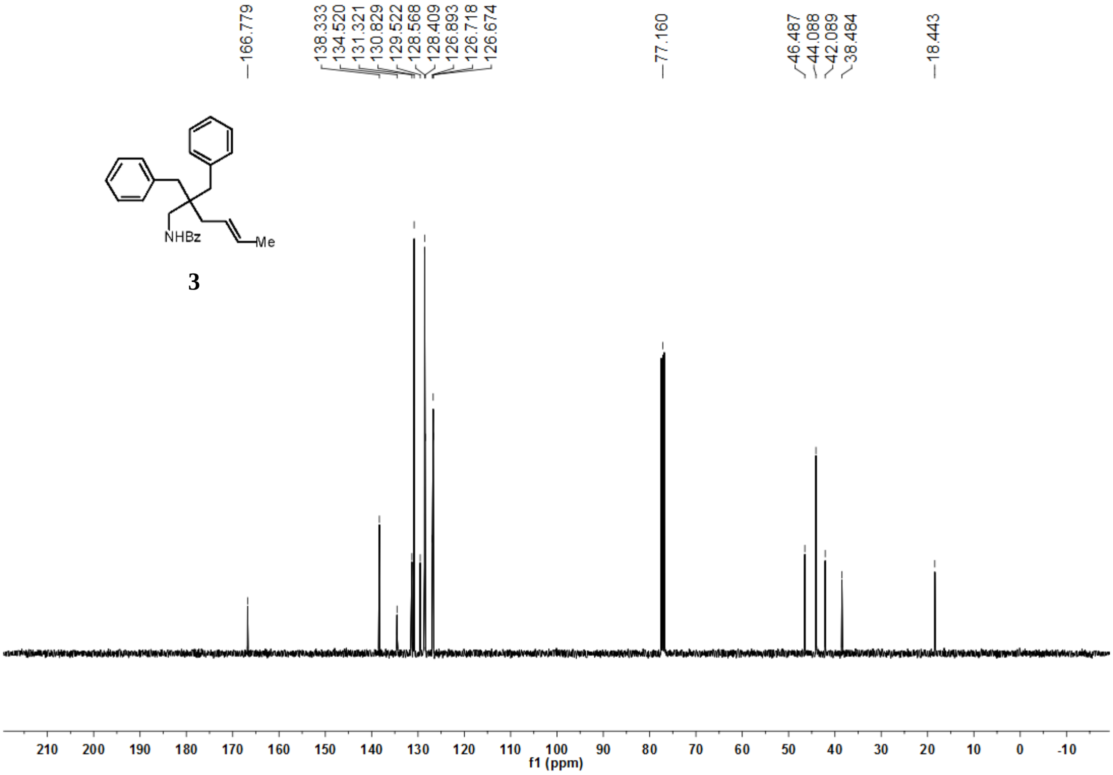

Supplementary Figure 62. ^{13}C NMR spectrum of compound **3**in $CDCl_3$

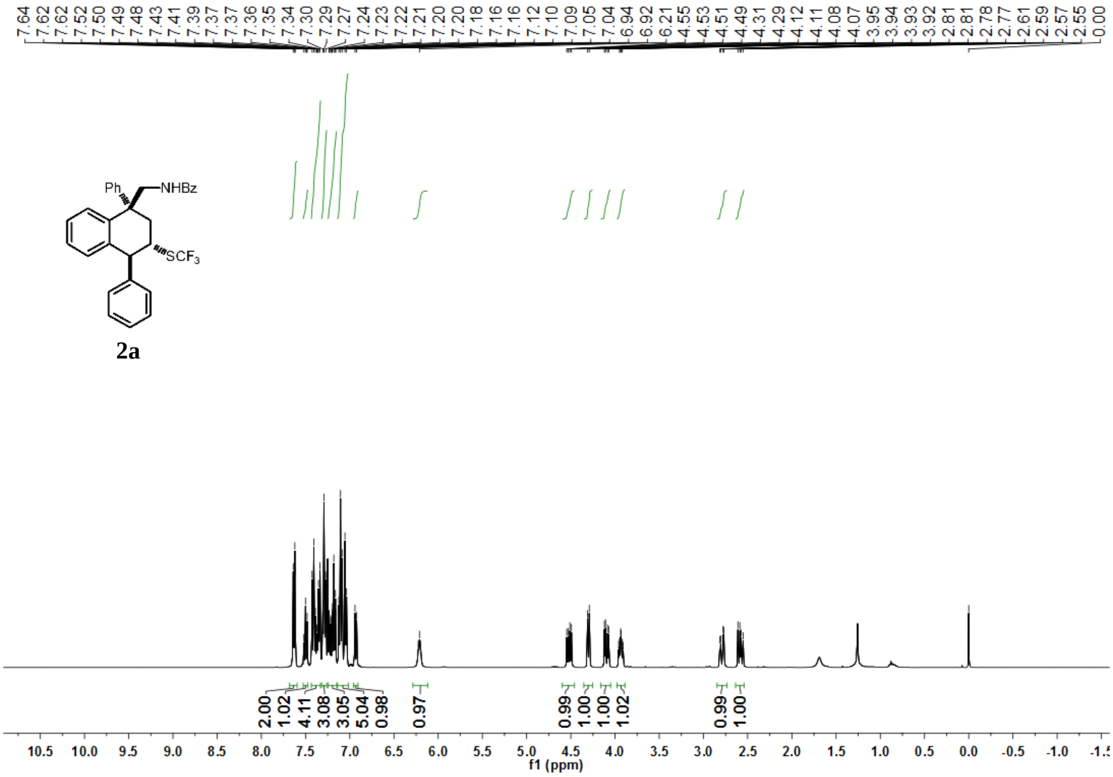

Supplementary Figure 63. ^{1}H NMR spectrum of compound **2a** in $CDCl_3$

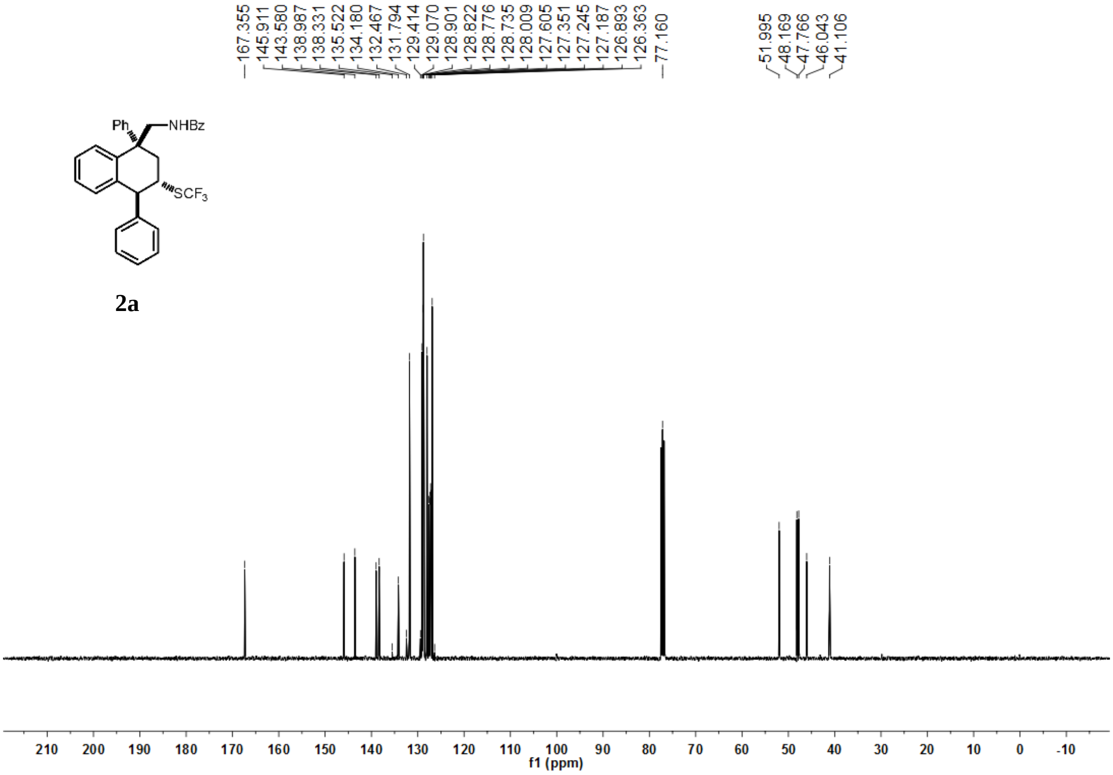

Supplementary Figure 64. ^{13}C NMR spectrum of compound **2a** in $CDCl_3$

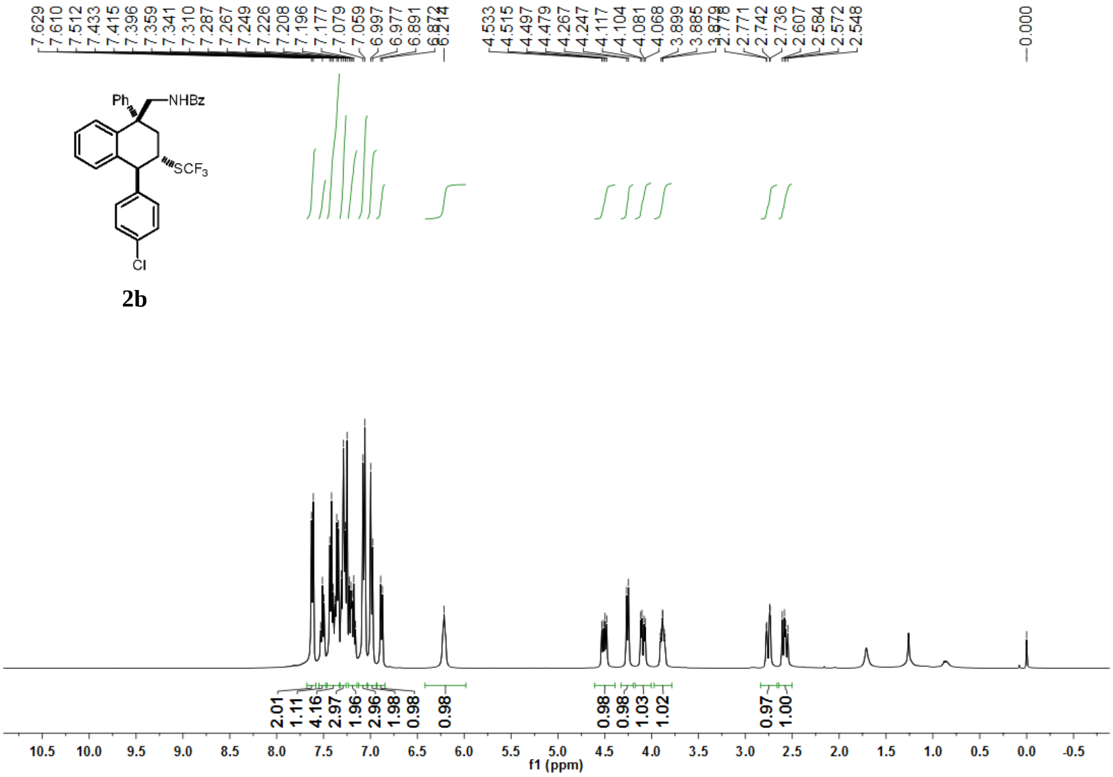

Supplementary Figure 65. ^{1}H NMR spectrum of compound **2b** in $CDCl_3$

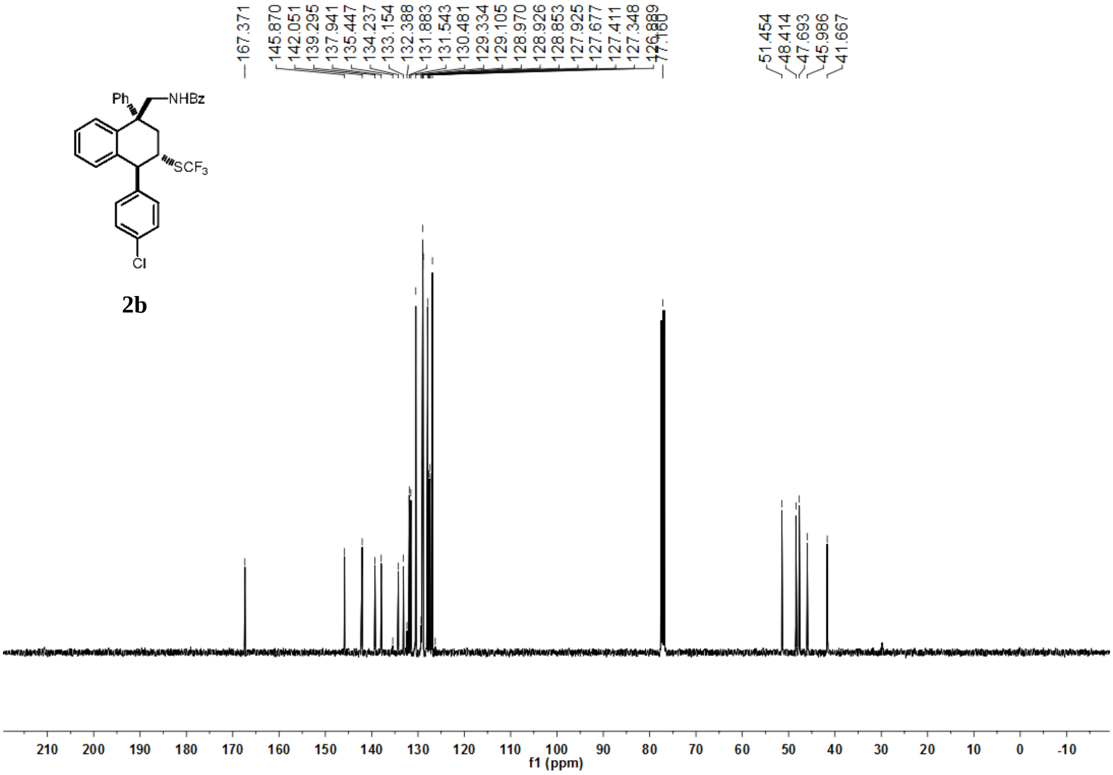

Supplementary Figure 66. ^{13}C NMR spectrum of compound **2b** in $CDCl_3$

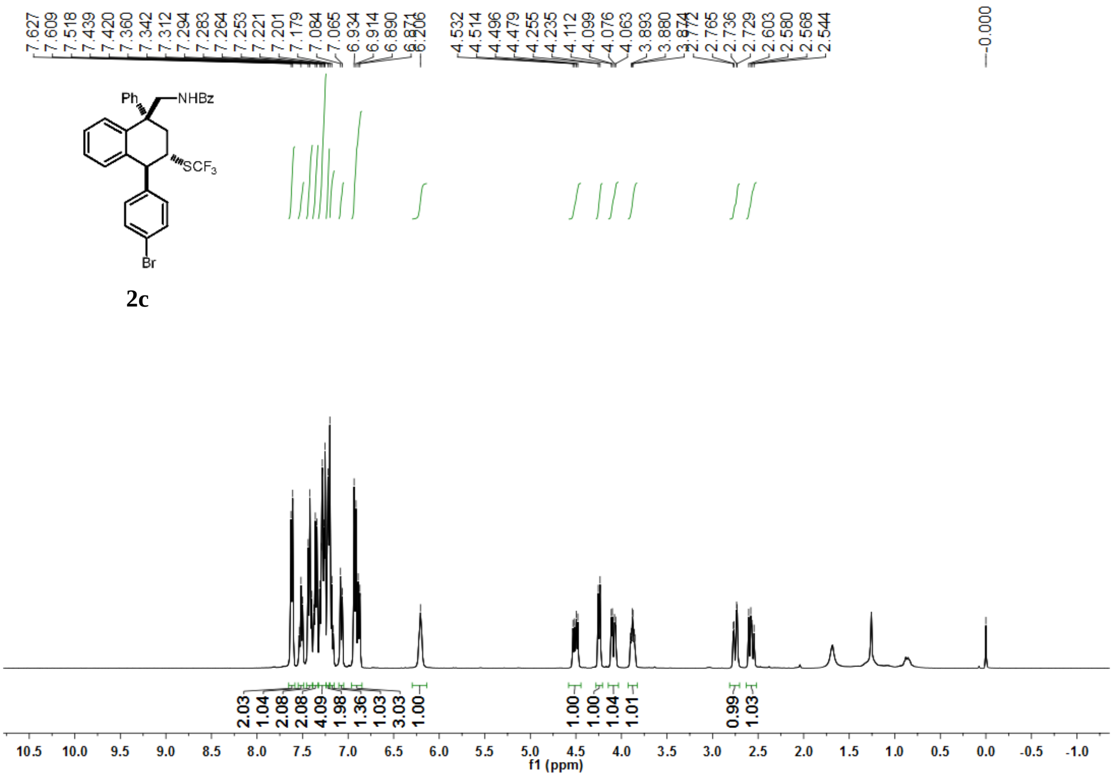

Supplementary Figure 67. ^{1}H NMR spectrum of compound **2c** in $CDCl_3$

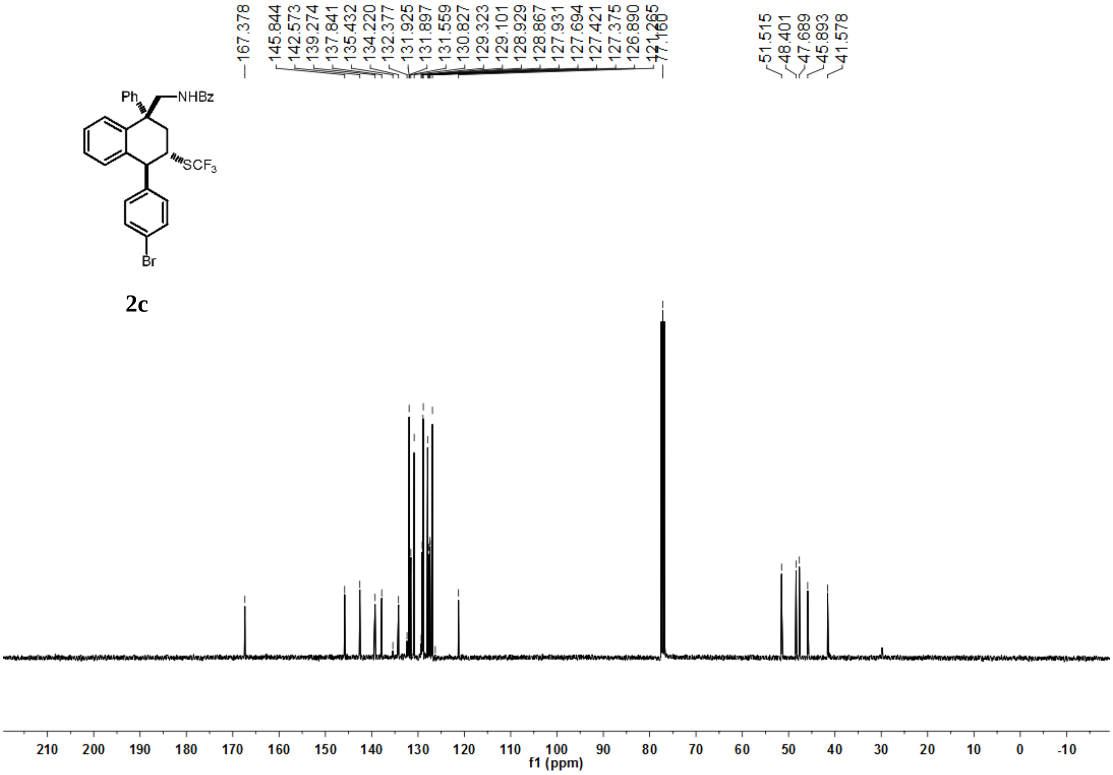

Supplementary Figure 68. ^{13}C NMR spectrum of compound **2c** in $CDCl_3$

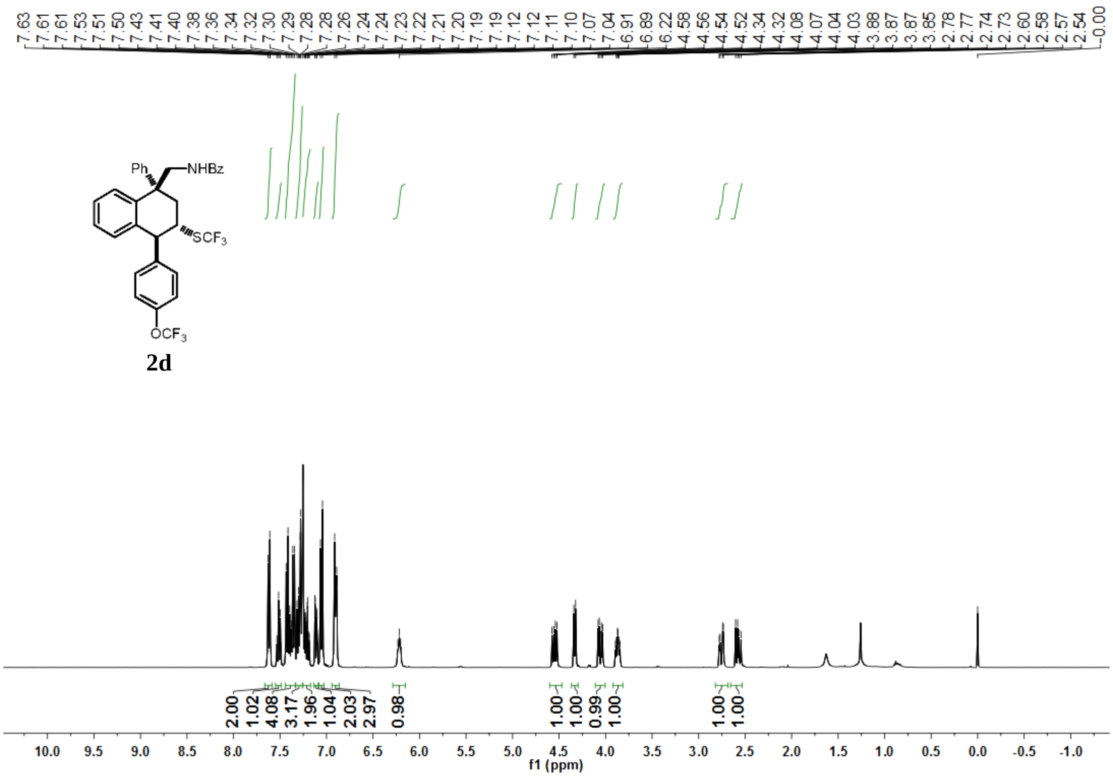

Supplementary Figure 69. ^{1}H NMR spectrum of compound **2d** in $CDCl_3$

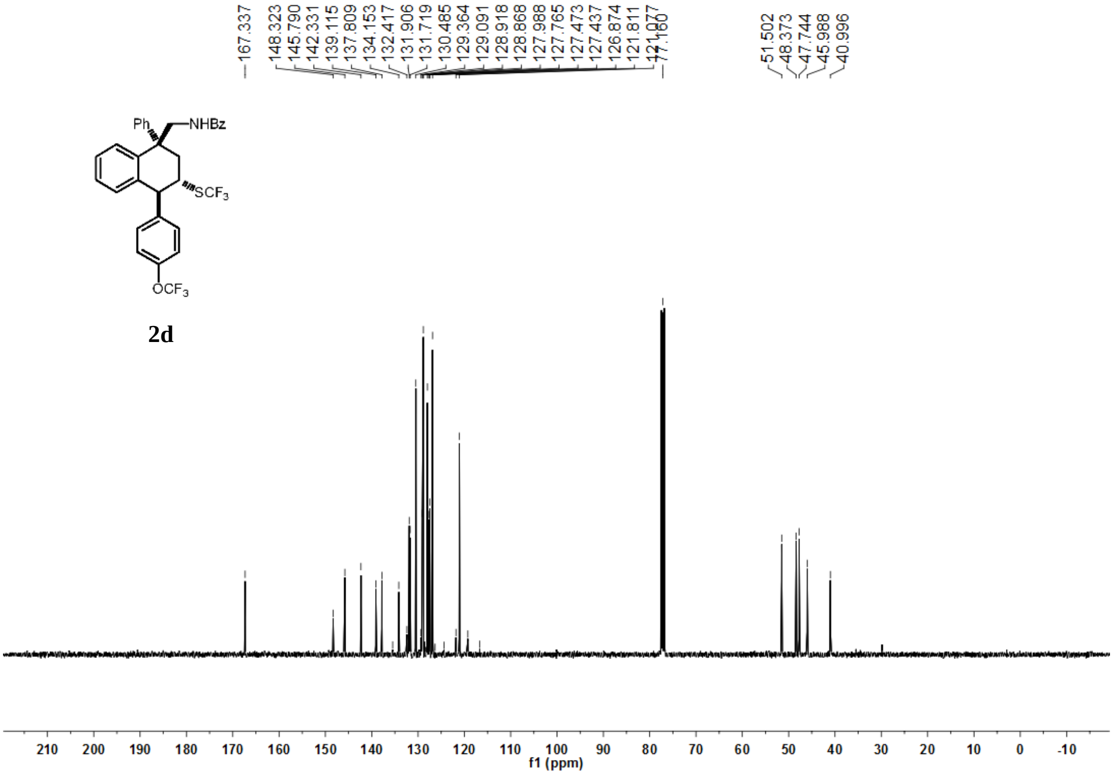

Supplementary Figure 70. ^{13}C NMR spectrum of compound **2d** in $CDCl_3$

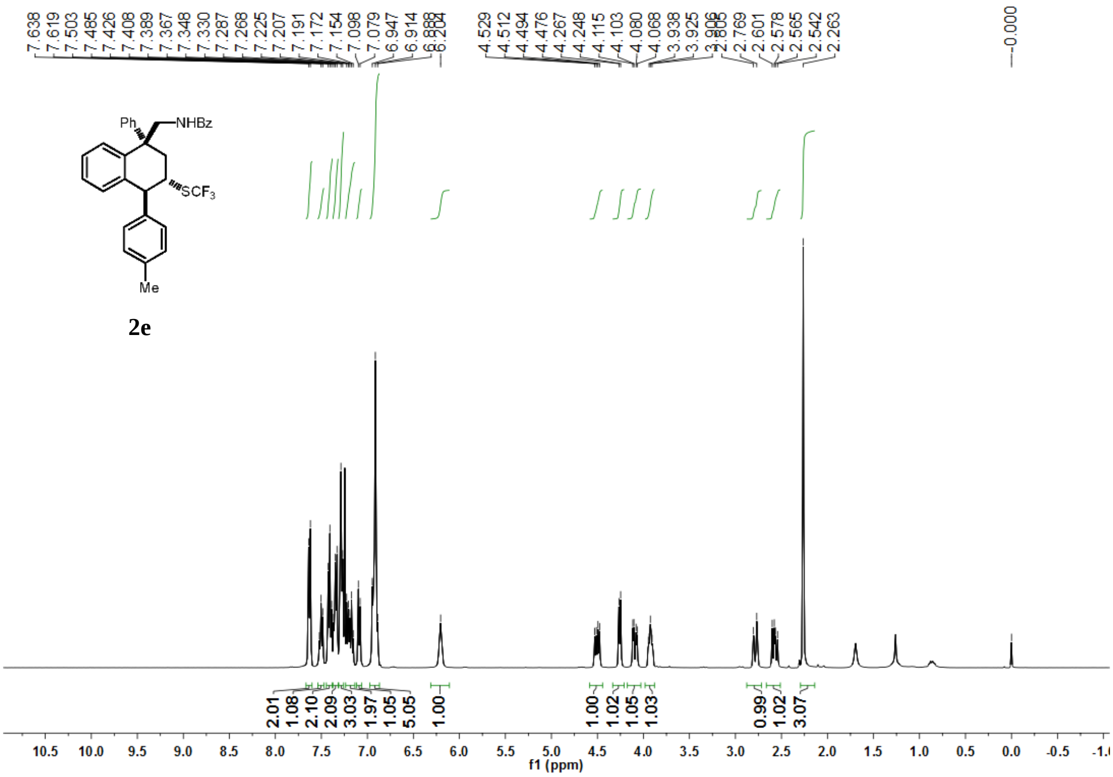

Supplementary Figure 71. ^{1}H NMR spectrum of compound **2e** in $CDCl_3$

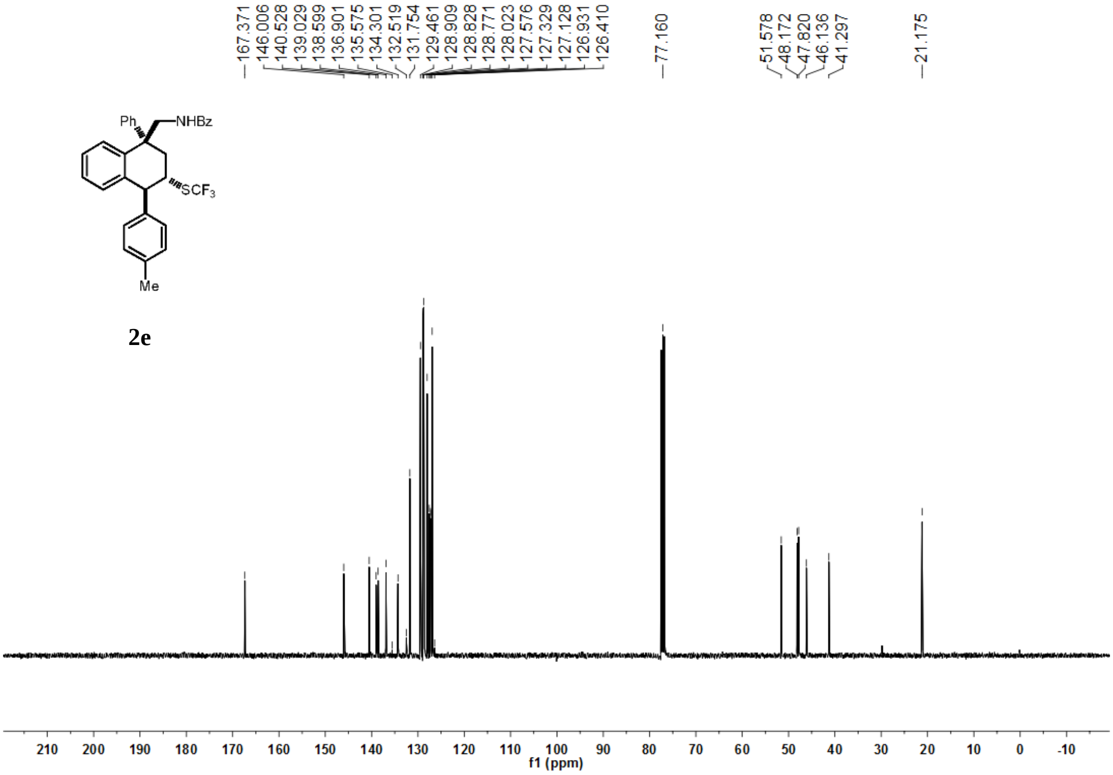

Supplementary Figure 72. ^{13}C NMR spectrum of compound **2e** in $CDCl_3$

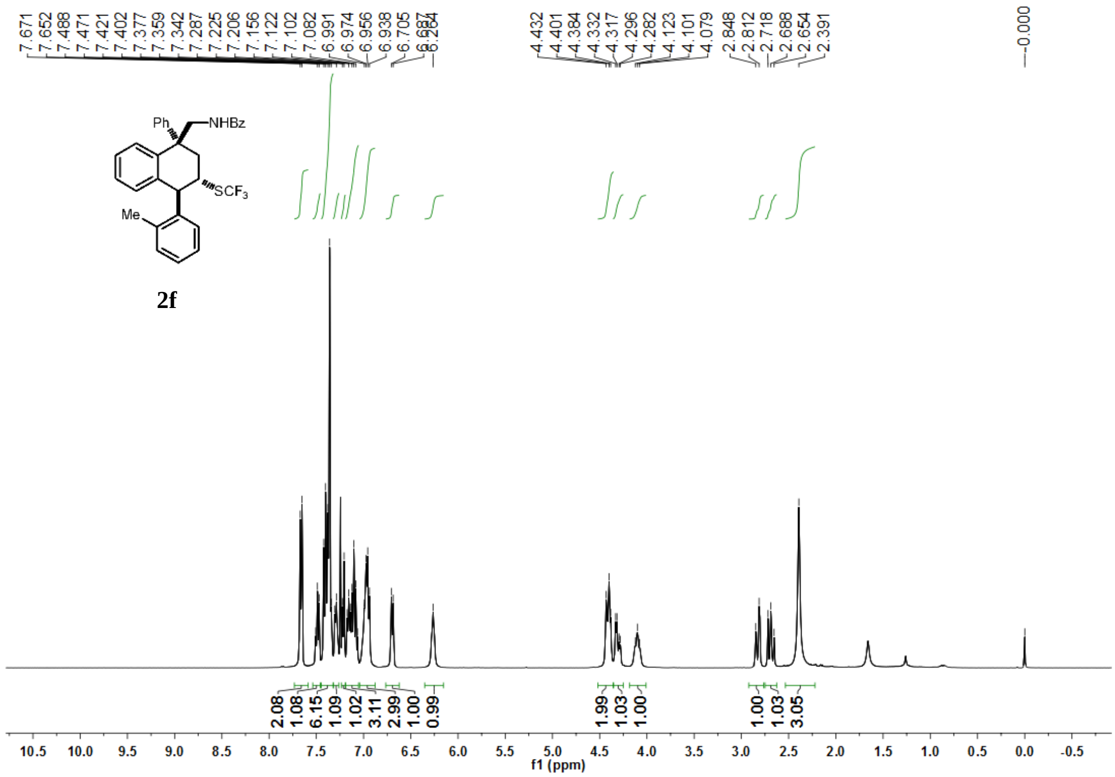

Supplementary Figure 73. ^{1}H NMR spectrum of compound **2f** in $CDCl_3$

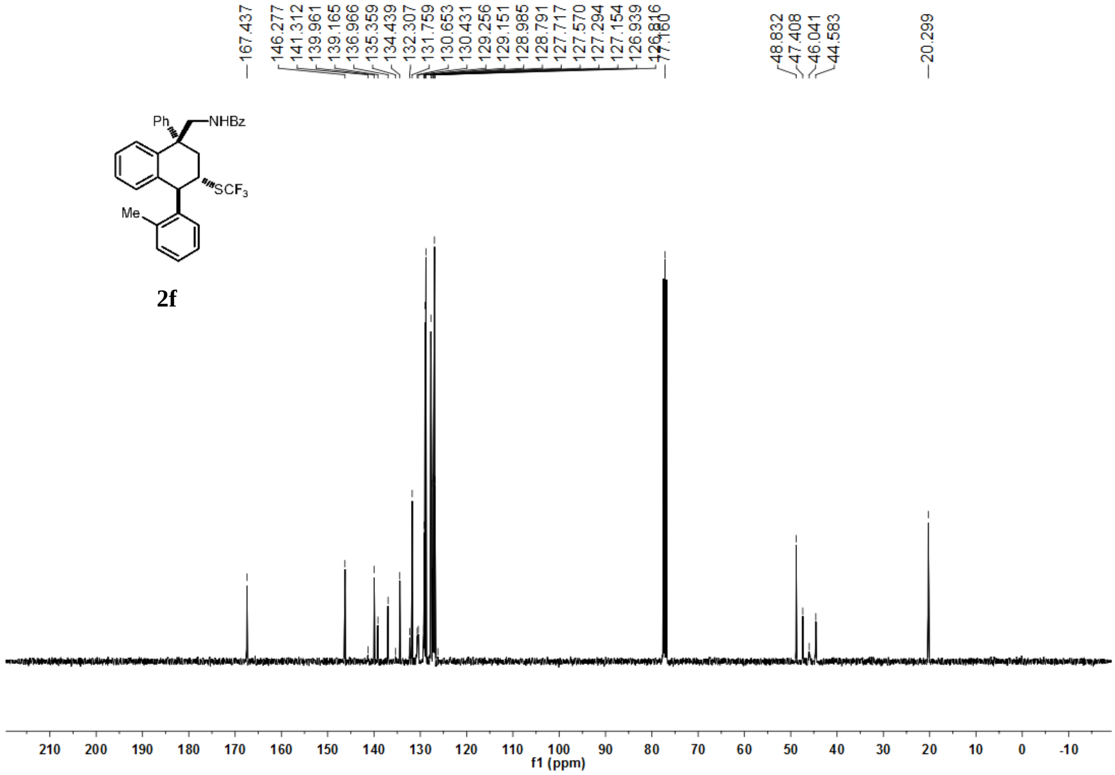

Supplementary Figure 74. ^{13}C NMR spectrum of compound **2f** in $CDCl_3$

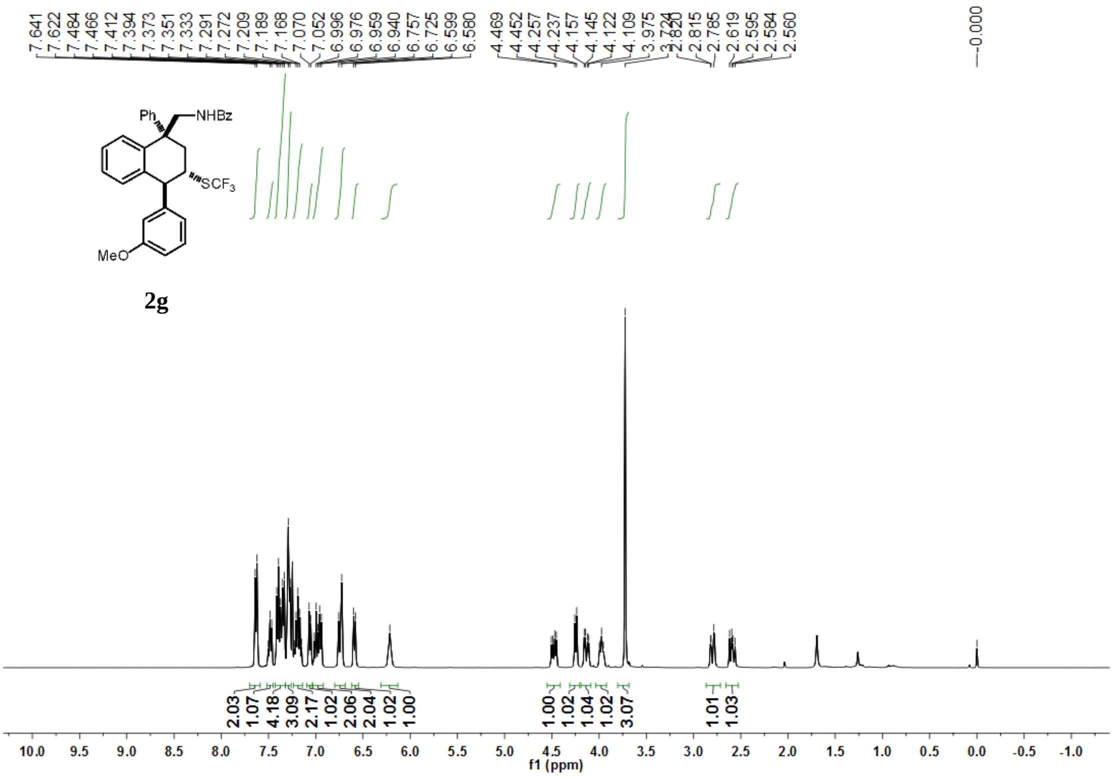

Supplementary Figure 75. ^{1}H NMR spectrum of compound **2g** in $CDCl_3$

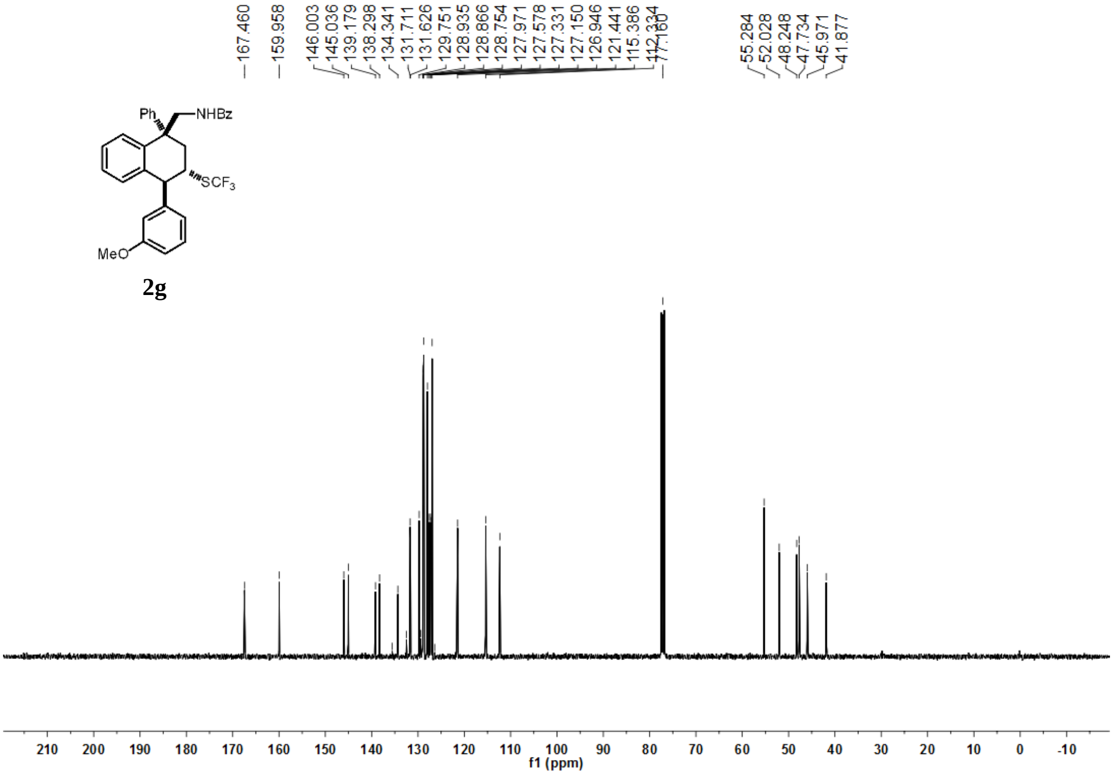

Supplementary Figure 76. ^{13}C NMR spectrum of compound **2g** in $CDCl_3$

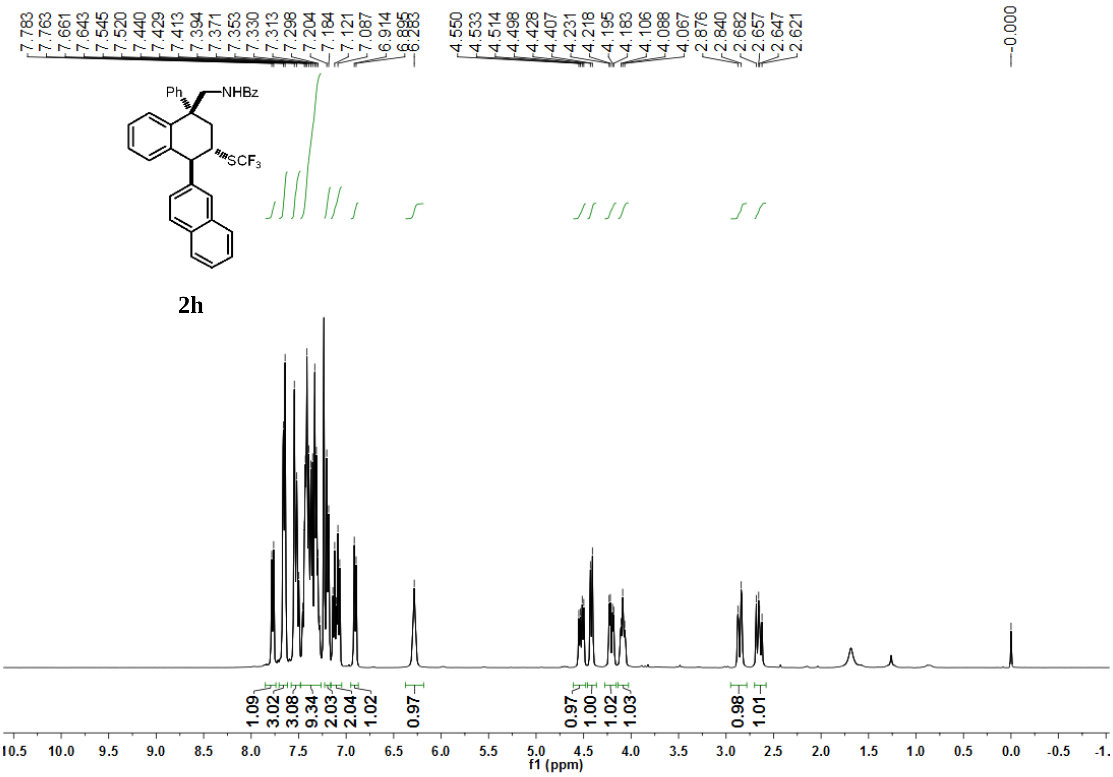

Supplementary Figure 77. ^{1}H NMR spectrum of compound **2h** in $CDCl_3$

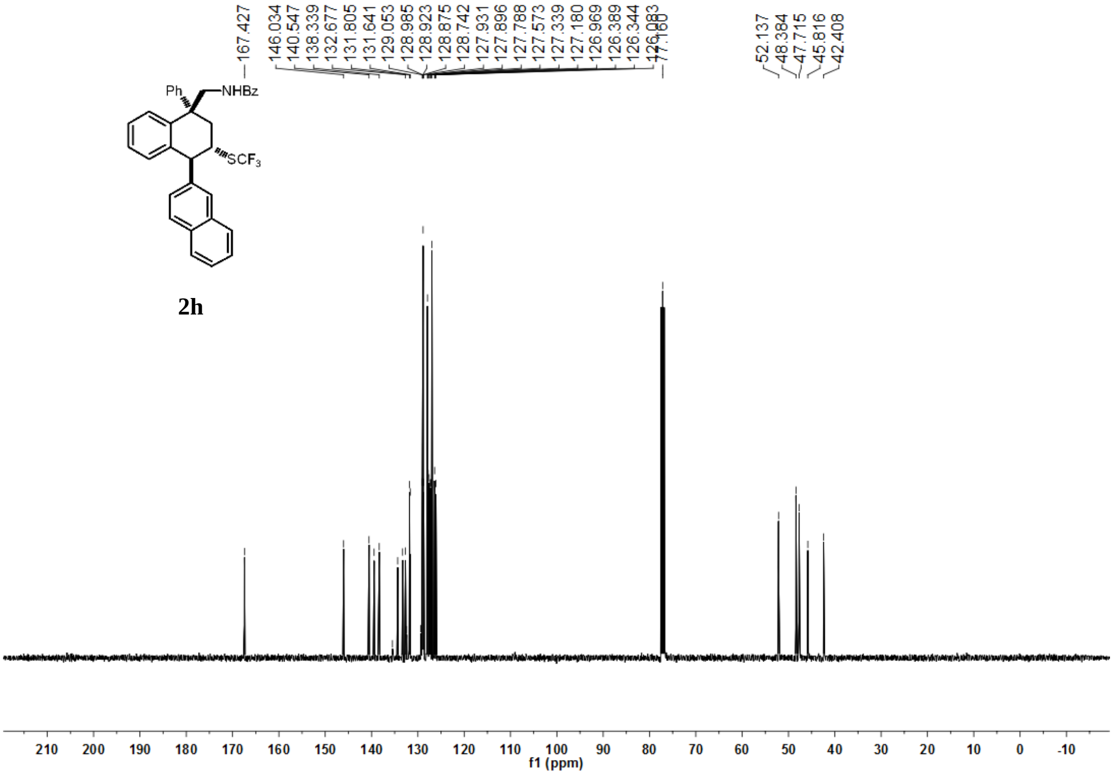

Supplementary Figure 78. ^{13}C NMR spectrum of compound **2h** in $CDCl_3$

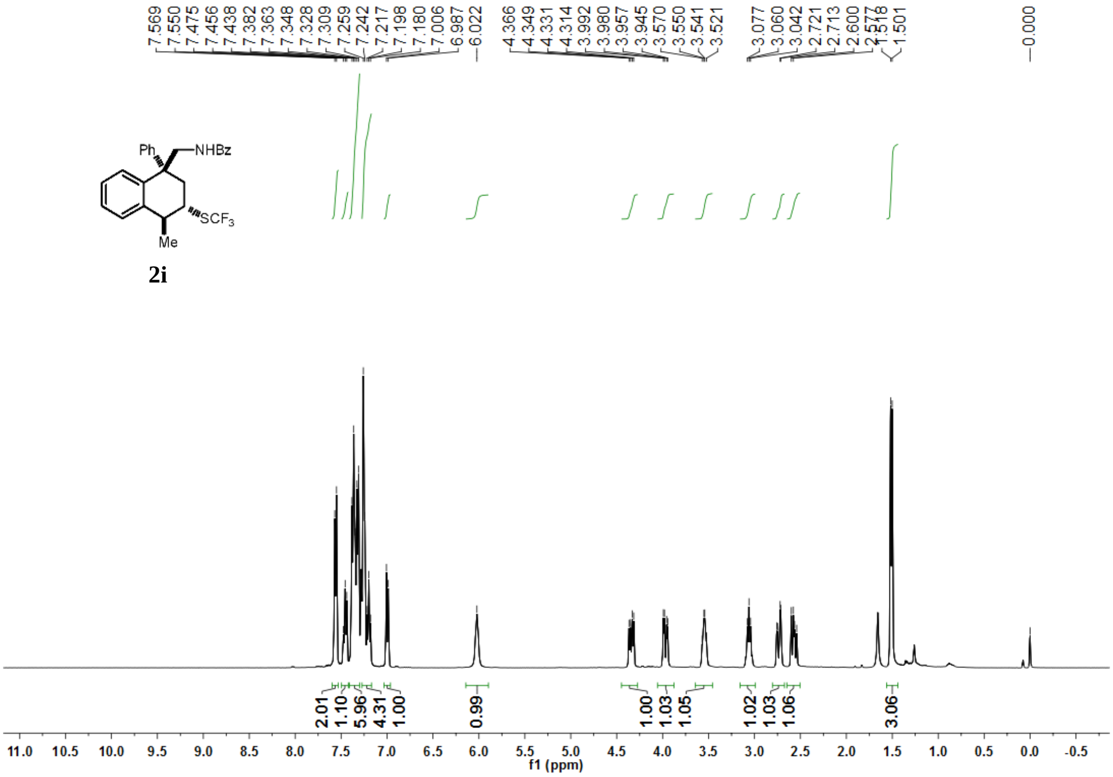

Supplementary Figure 79. ^{1}H NMR spectrum of compound **2i** in $CDCl_3$

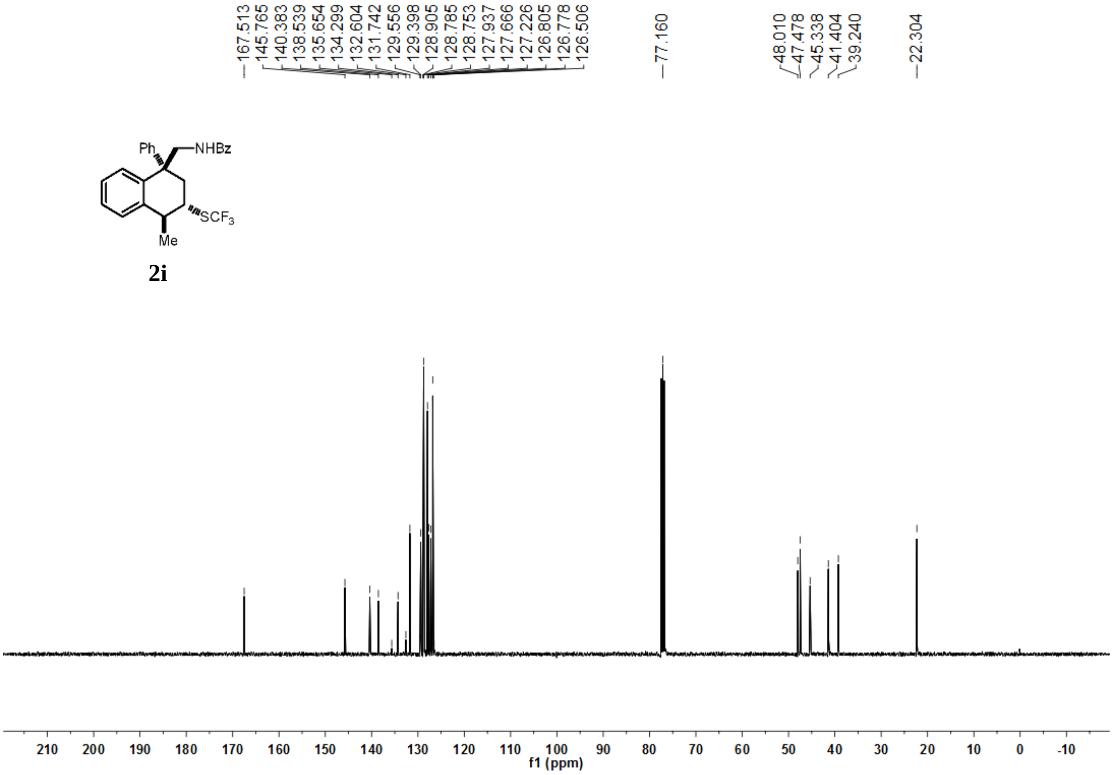

Supplementary Figure 80. ^{13}C NMR spectrum of compound **2i** in $CDCl_3$

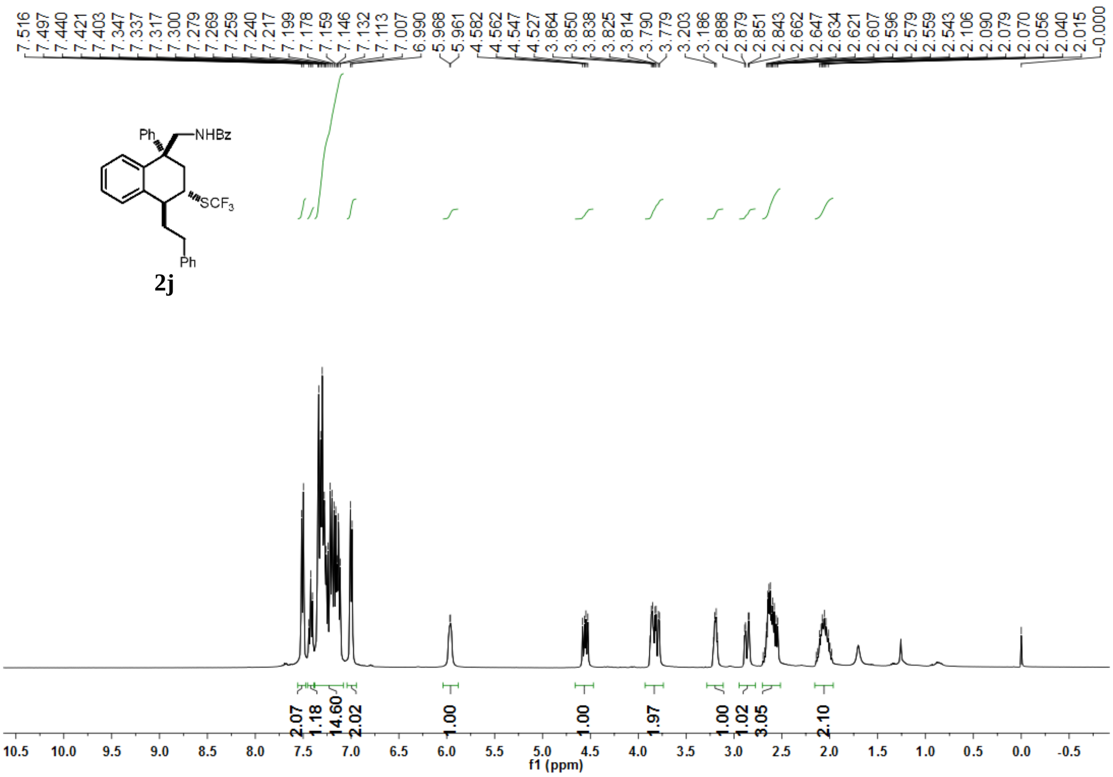

Supplementary Figure 81. ^{1}H NMR spectrum of compound **2j** in $CDCl_3$

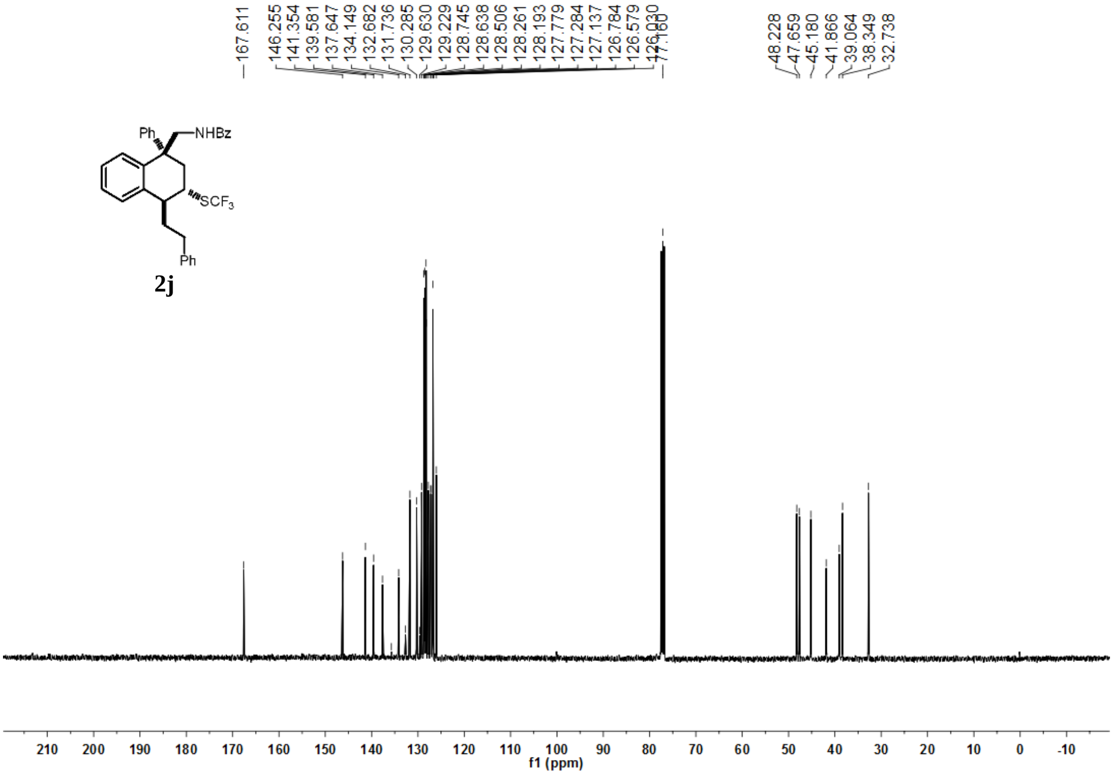

Supplementary Figure 82. ^{13}C NMR spectrum of compound **2j** in $CDCl_3$

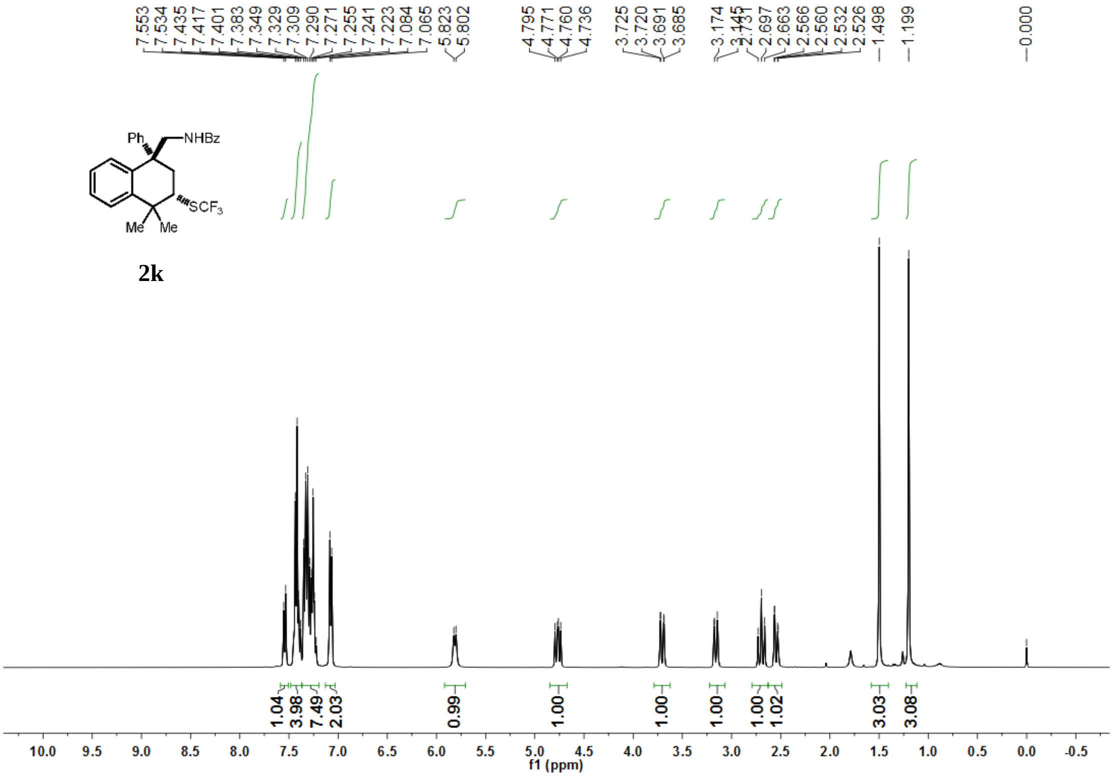

Supplementary Figure 83. ^{1}H NMR spectrum of compound **2k** in $CDCl_3$

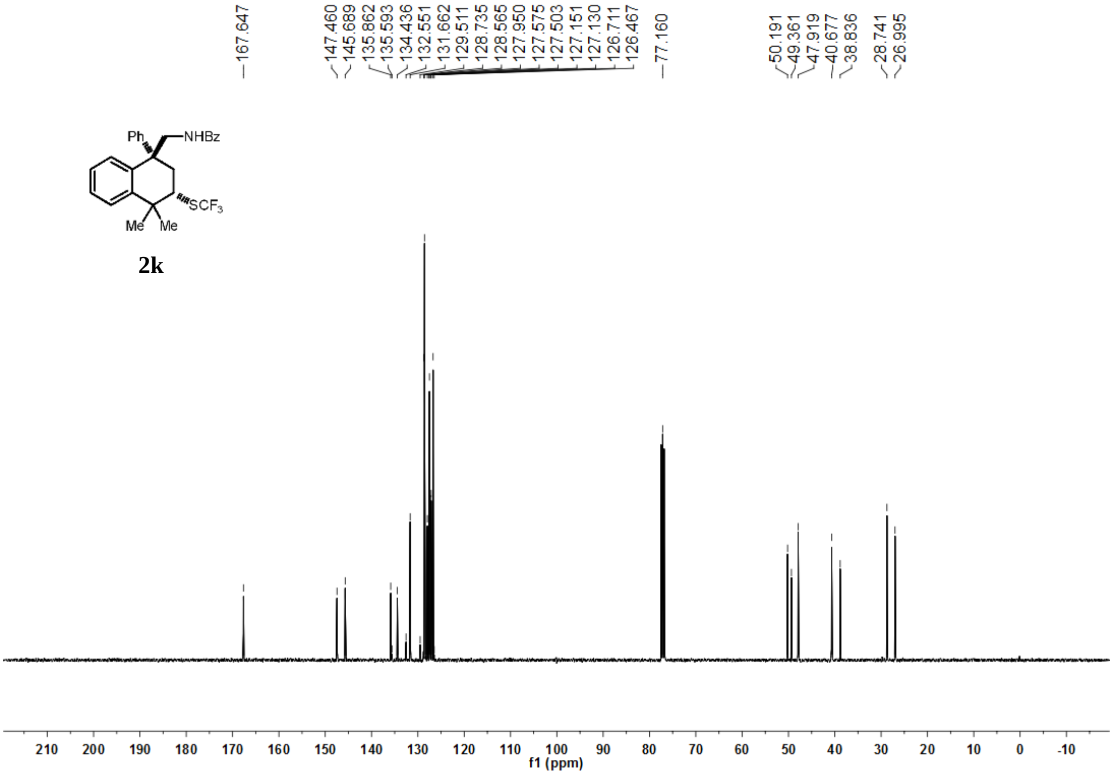

Supplementary Figure 84. ^{13}C NMR spectrum of compound **2k** in $CDCl_3$

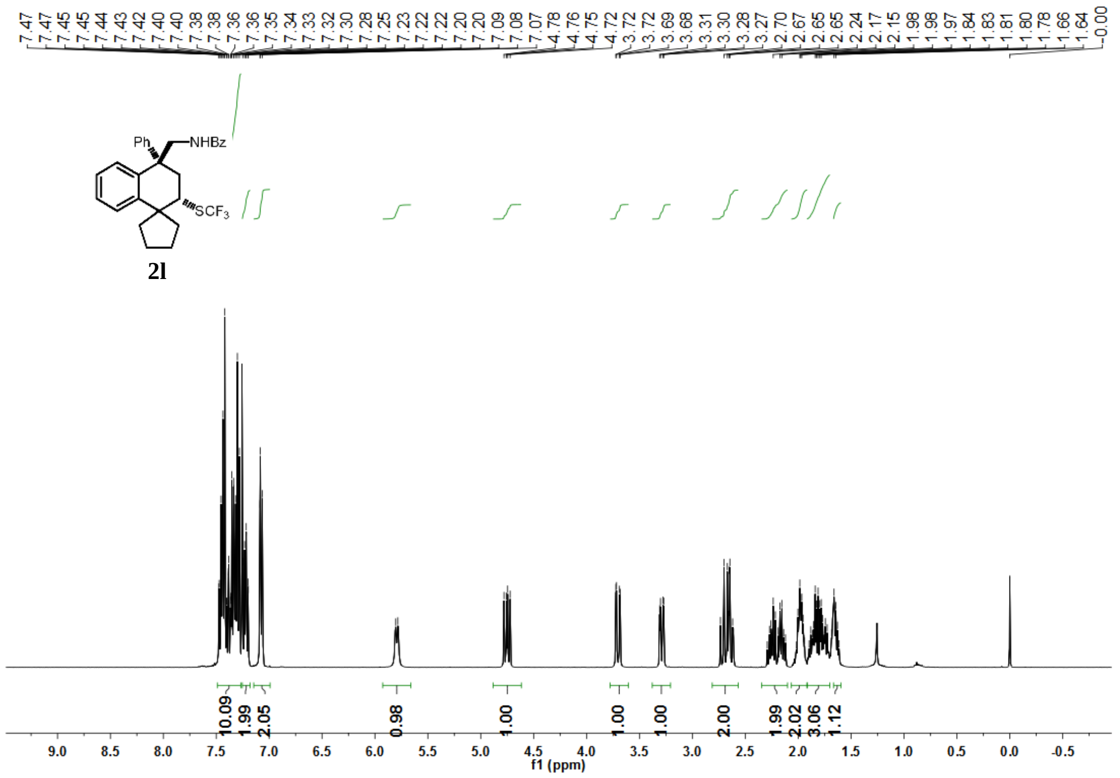

Supplementary Figure 85. ^{1}H NMR spectrum of compound **2l** in $CDCl_3$

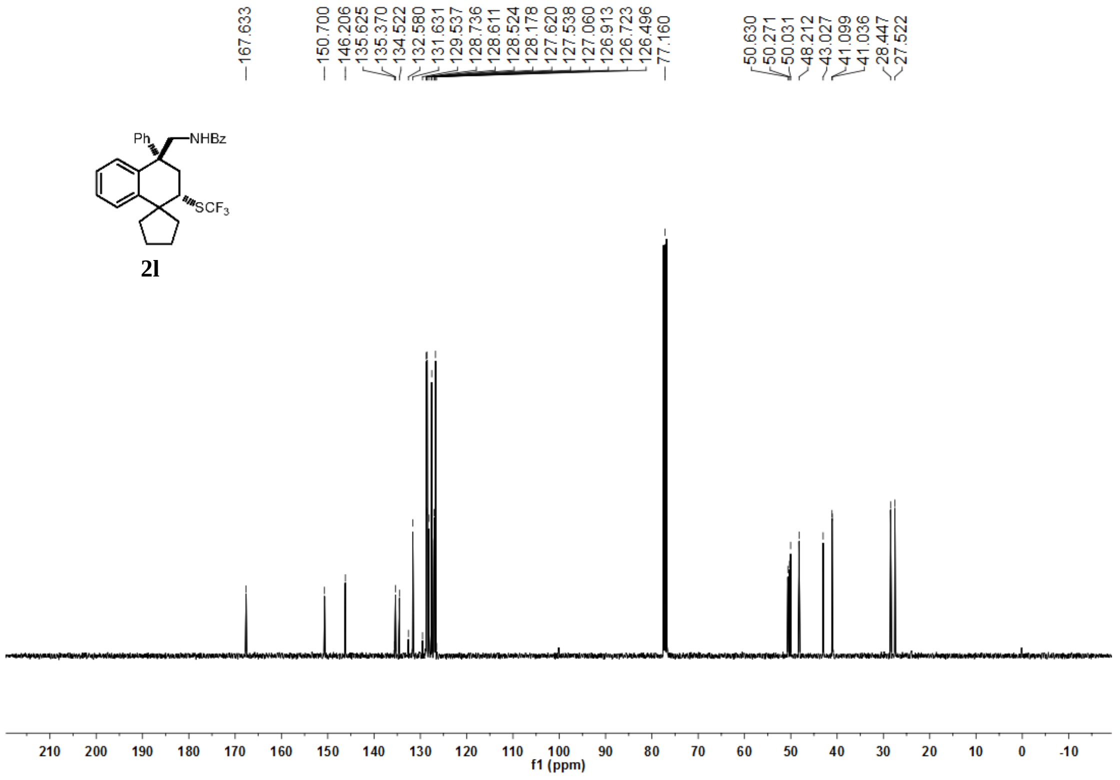

Supplementary Figure 86. ^{13}C NMR spectrum of compound **2l** in $CDCl_3$

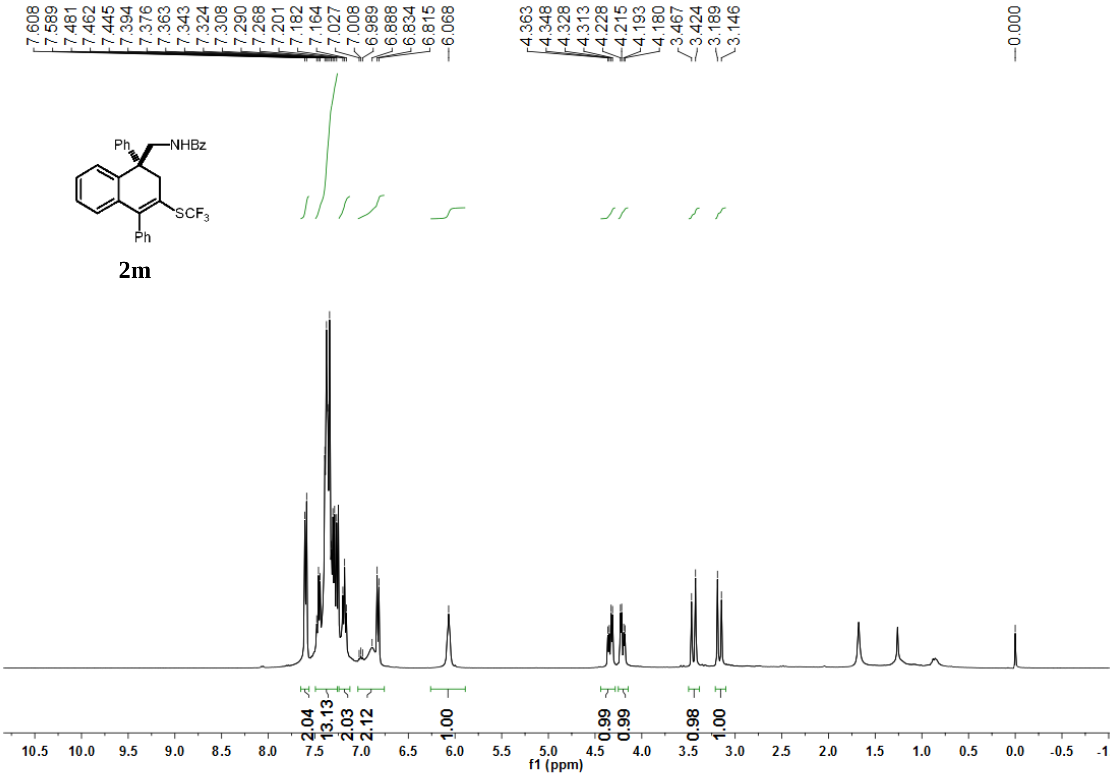

Supplementary Figure 87. ^{1}H NMR spectrum of compound **2m** in $CDCl_3$

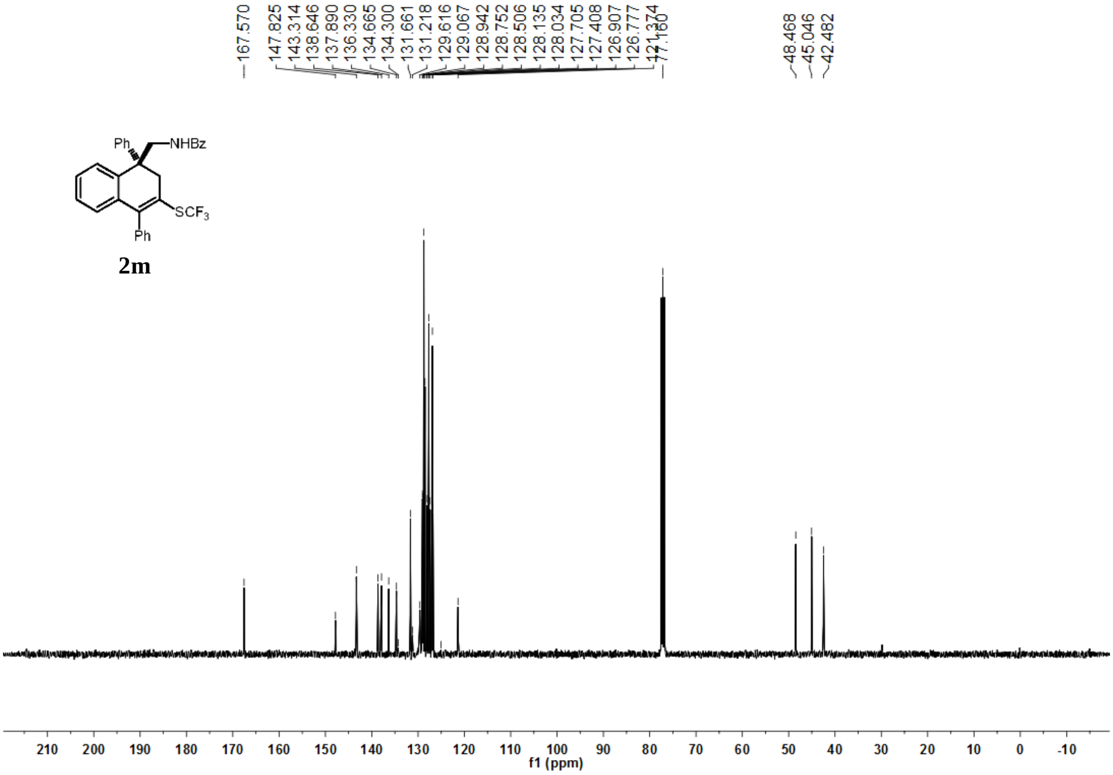

Supplementary Figure 88. ^{13}C NMR spectrum of compound **2m** in $CDCl_3$

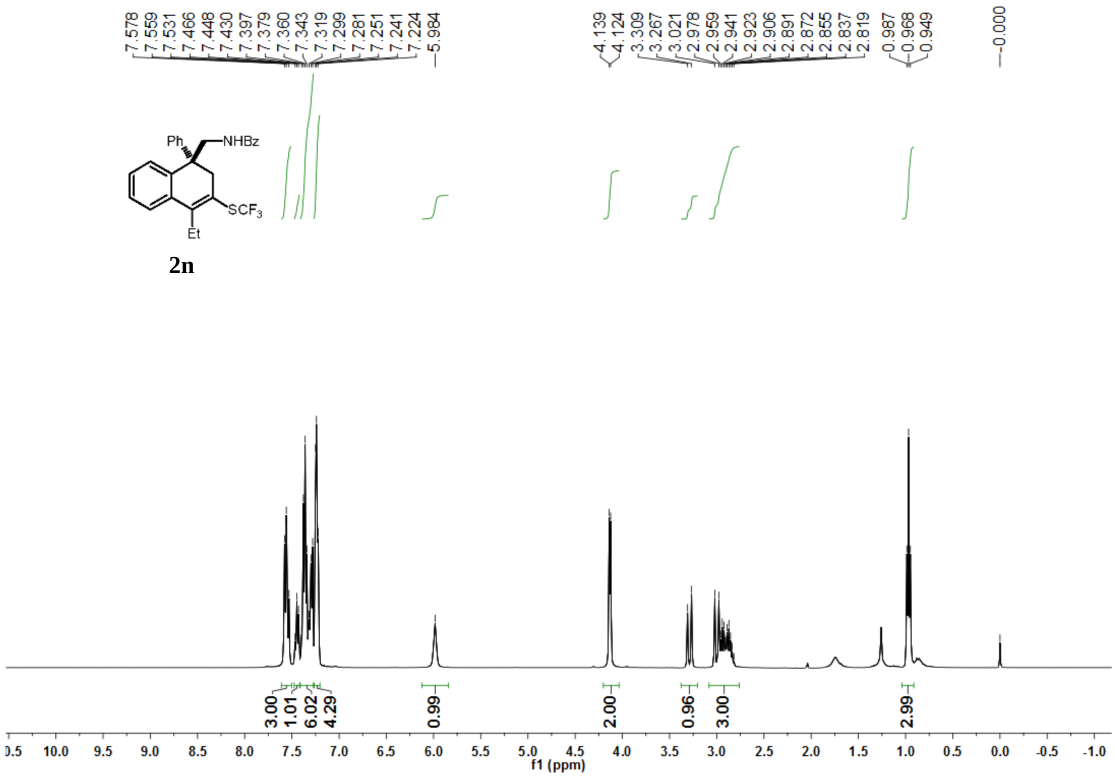

Supplementary Figure 89. ^{1}H NMR spectrum of compound **2n** in $CDCl_3$

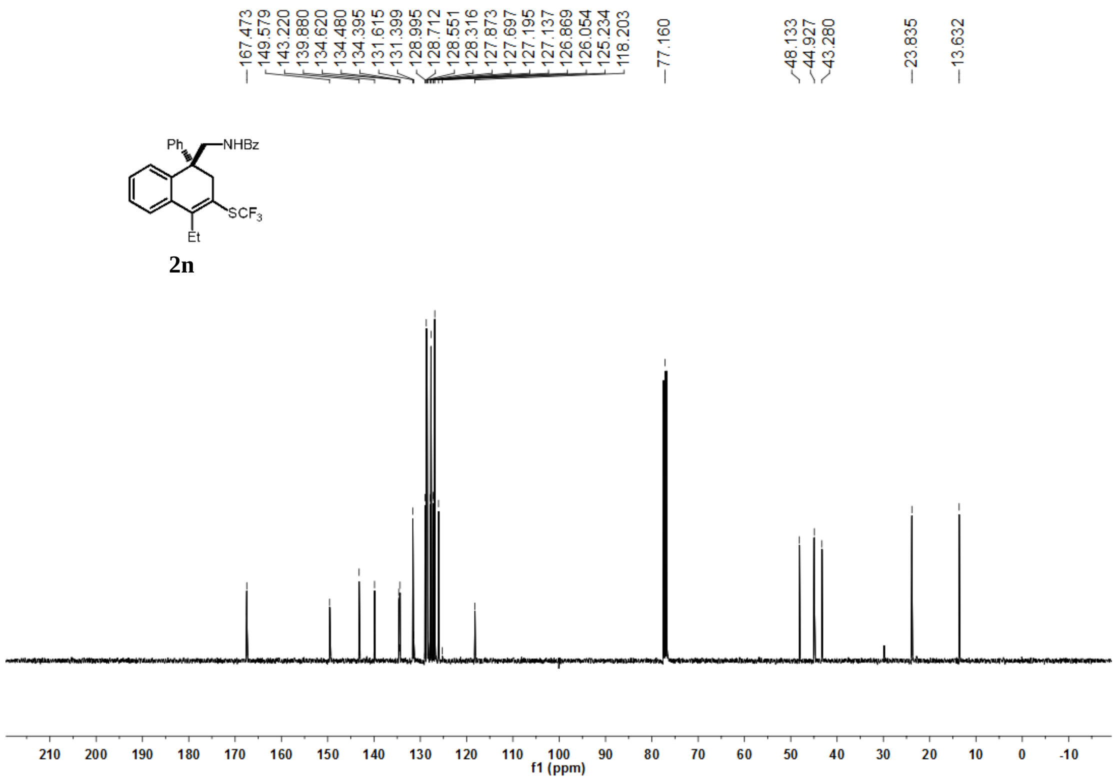

Supplementary Figure 90. ^{13}C NMR spectrum of compound **2n** in $CDCl_3$

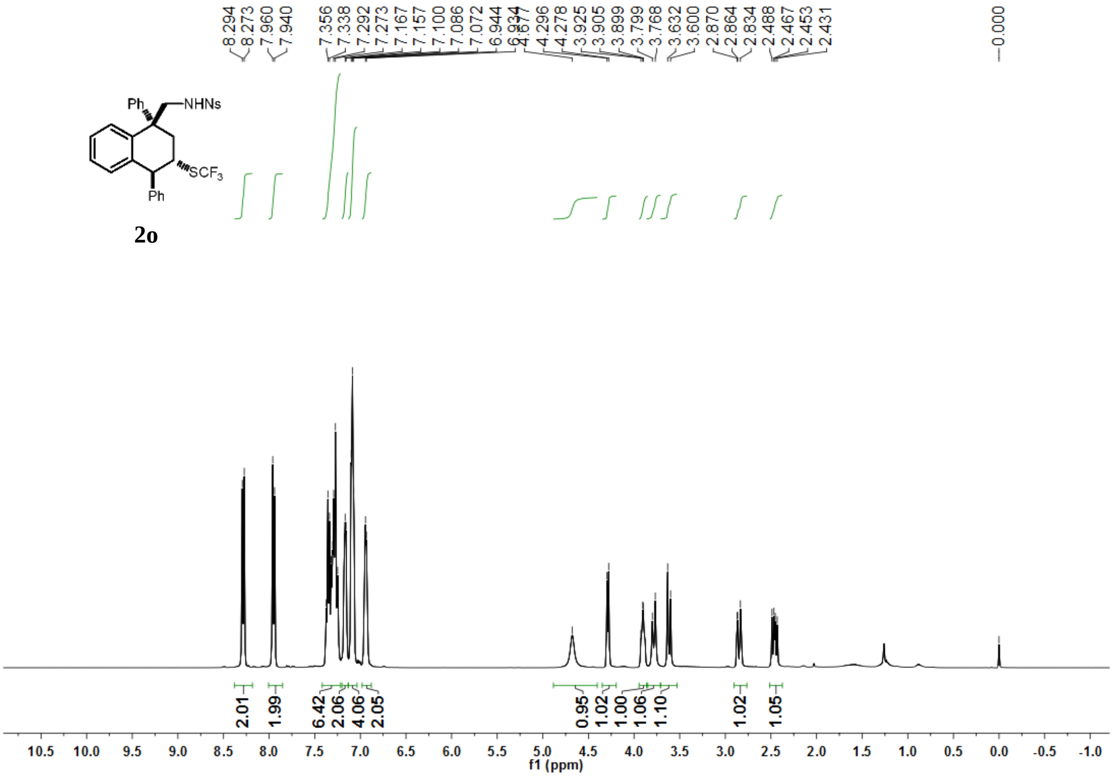

Supplementary Figure 91. ^{1}H NMR spectrum of compound **2o** in $CDCl_3$

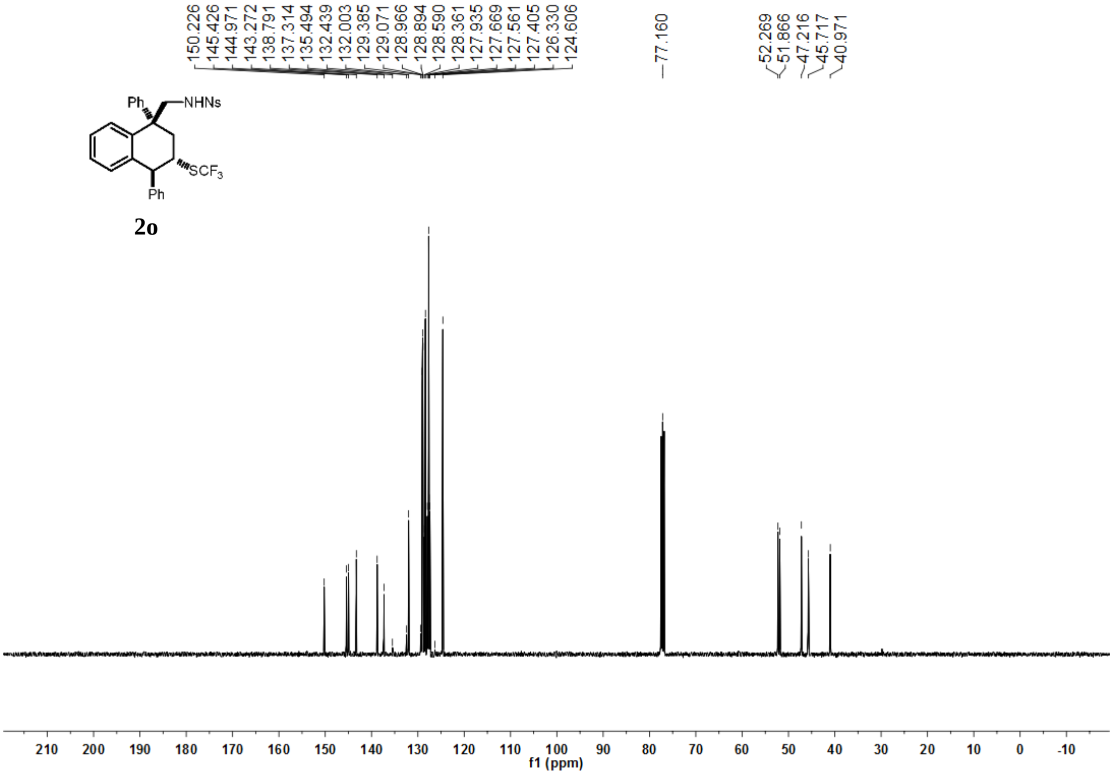

Supplementary Figure 92. ^{13}C NMR spectrum of compound **2o** in $CDCl_3$

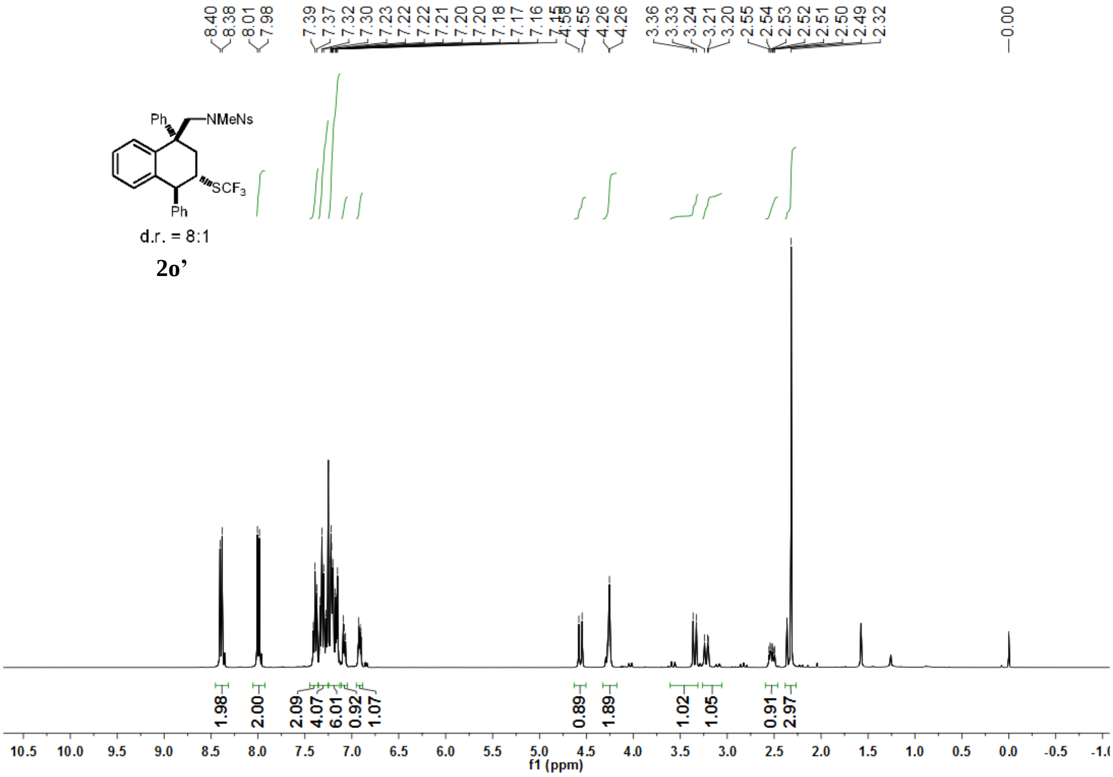

Supplementary Figure 93. ^{1}H NMR spectrum of compound **2o'** in $CDCl_3$

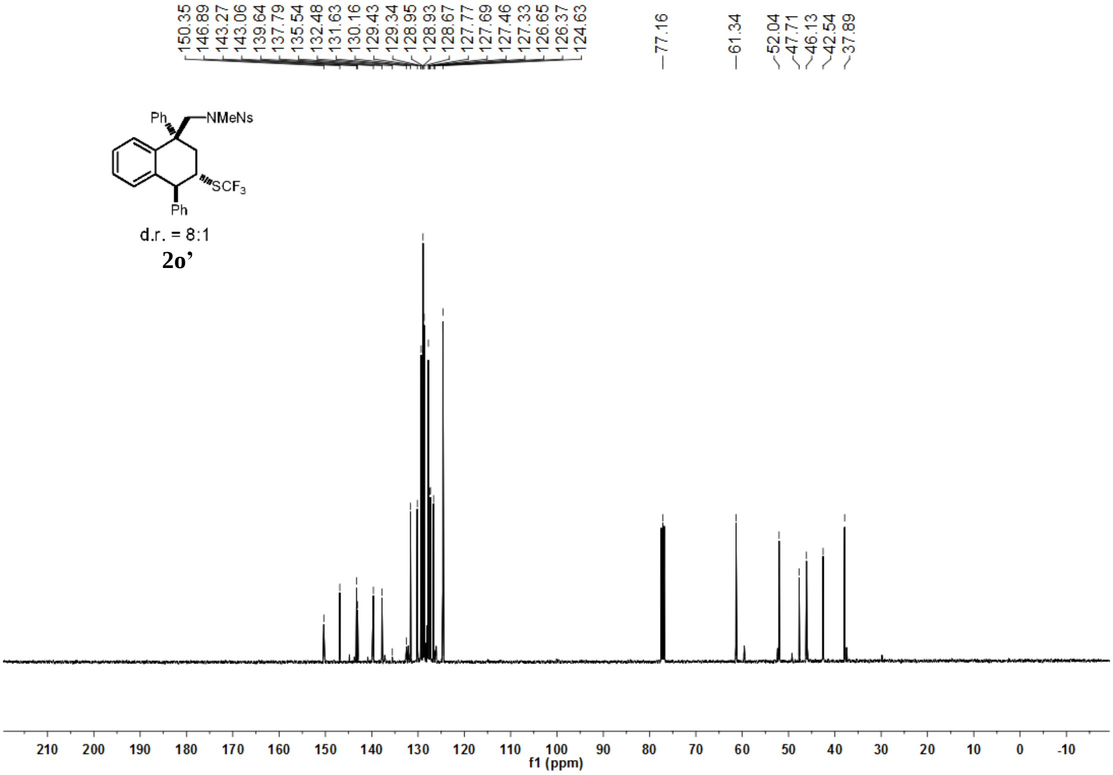

Supplementary Figure 94. ^{13}C NMR spectrum of compound **2o'** in $CDCl_3$

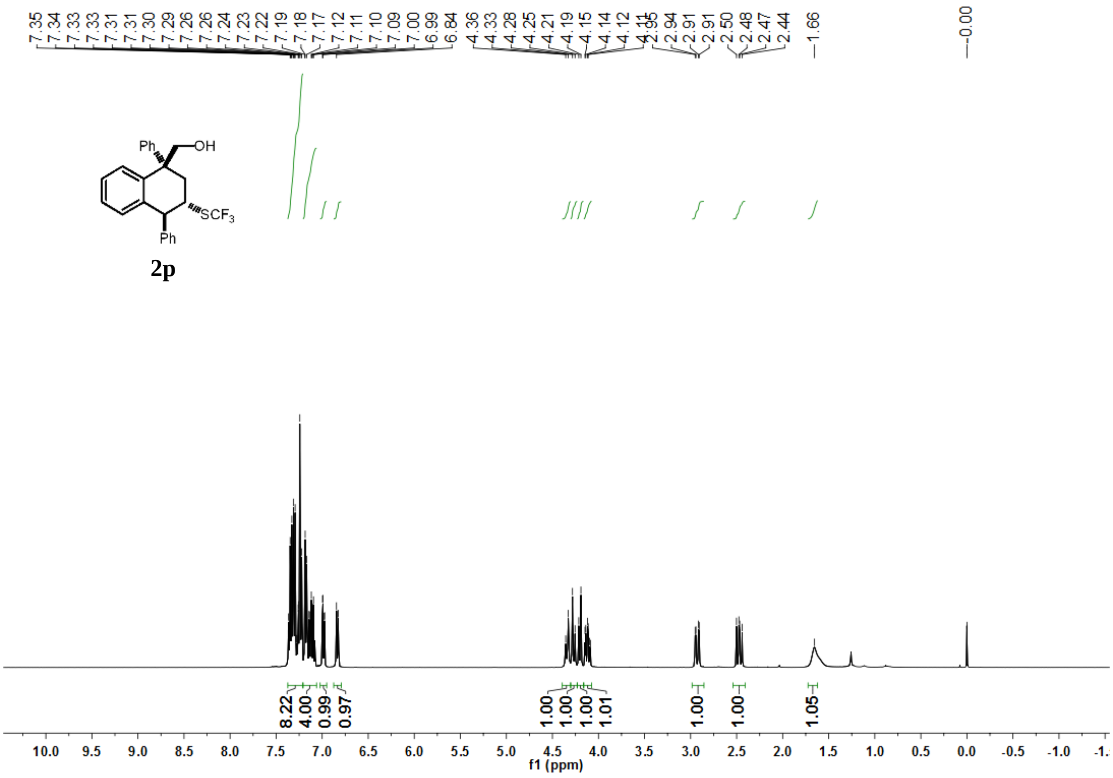

Supplementary Figure 95. ^{1}H NMR spectrum of compound **2p** in $CDCl_3$

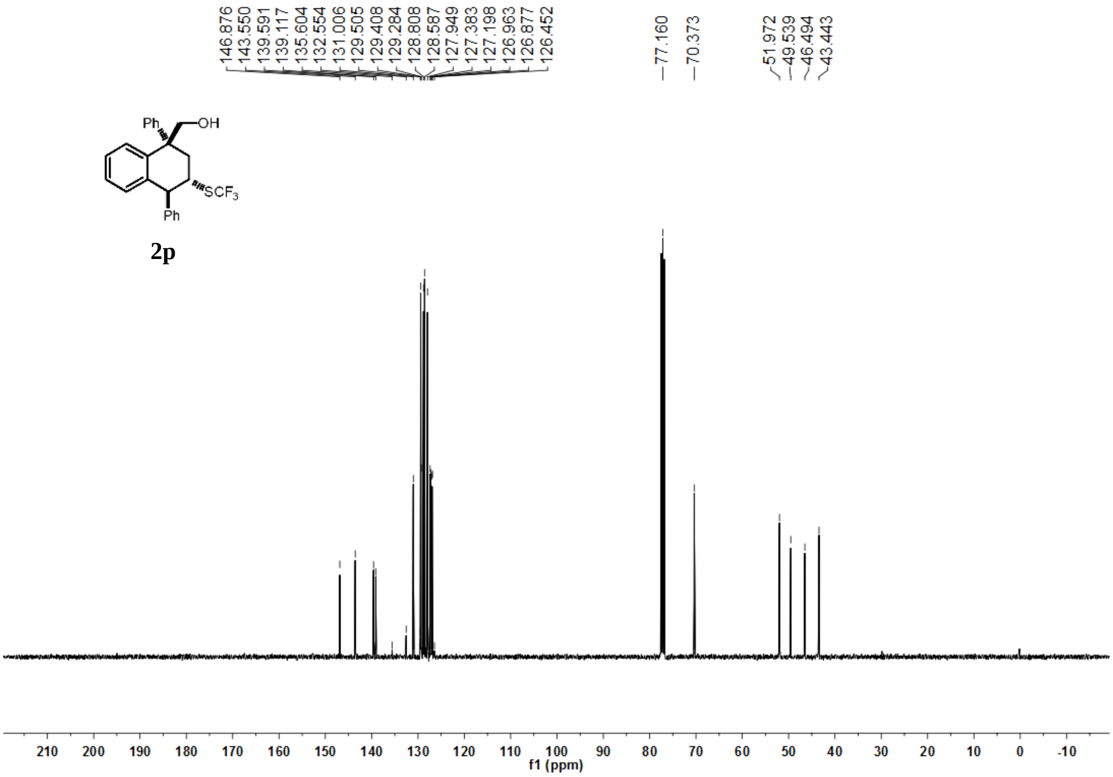

Supplementary Figure 96. ^{13}C NMR spectrum of compound **2p** in $CDCl_3$

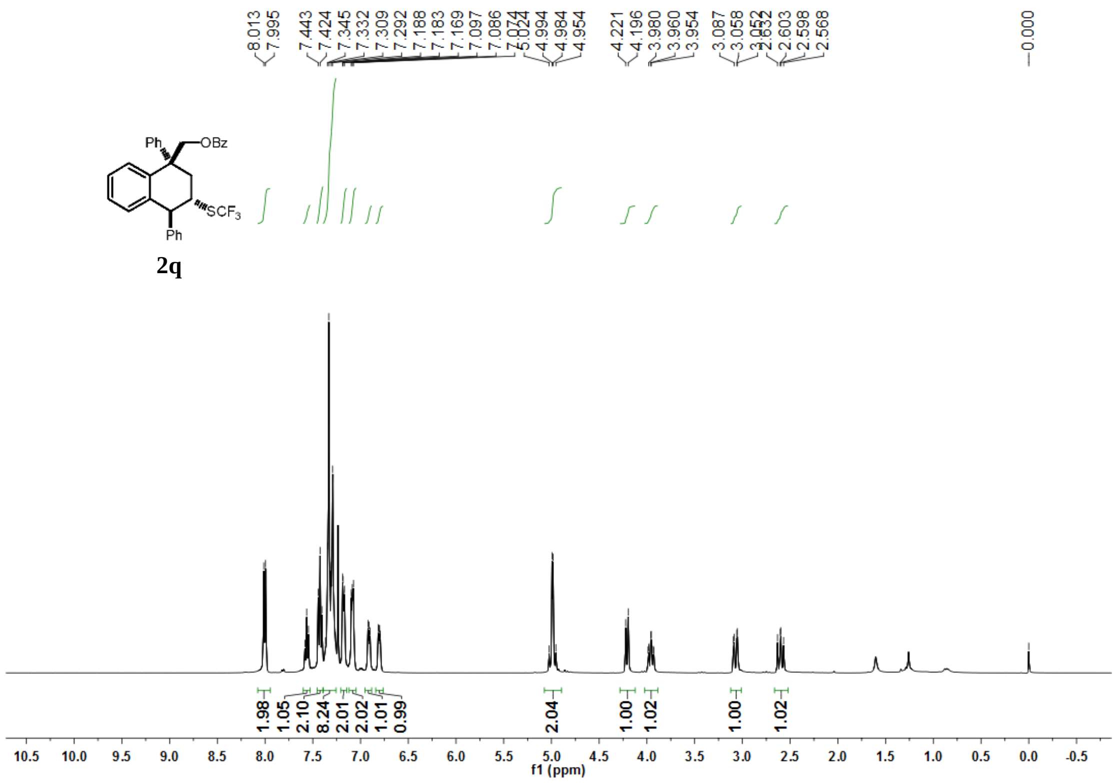

Supplementary Figure 97. ^{1}H NMR spectrum of compound **2q** in $CDCl_3$

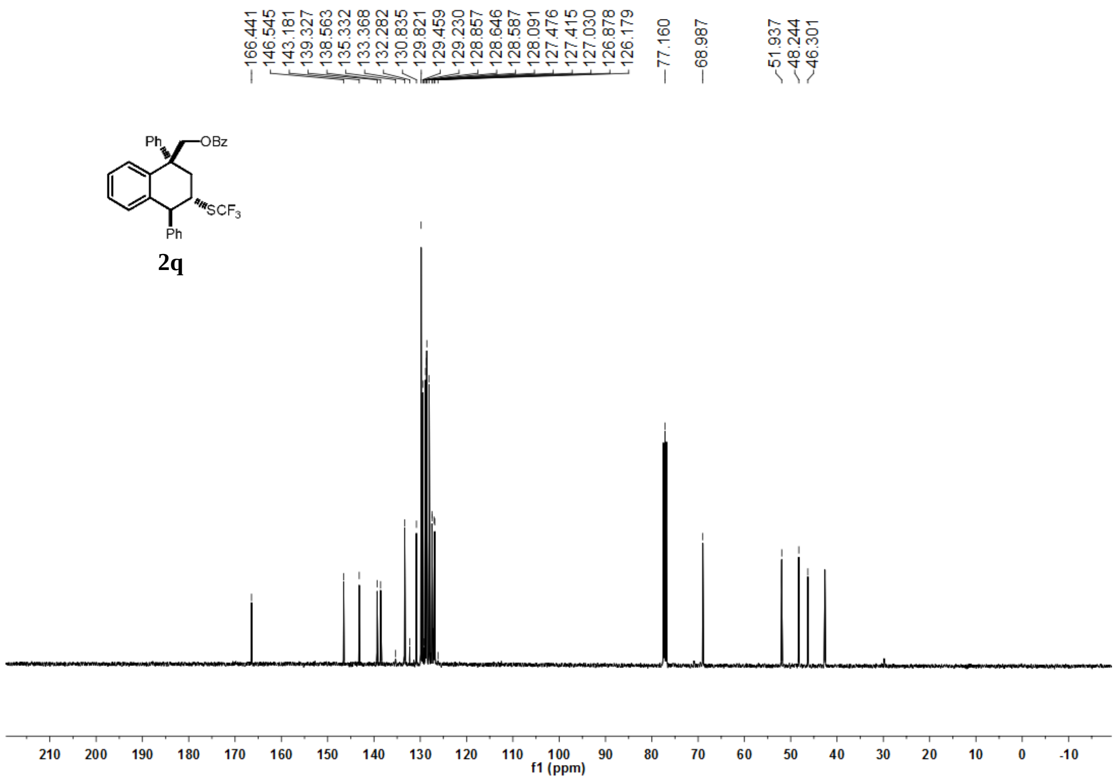

Supplementary Figure 98. ^{13}C NMR spectrum of compound **2q** in $CDCl_3$

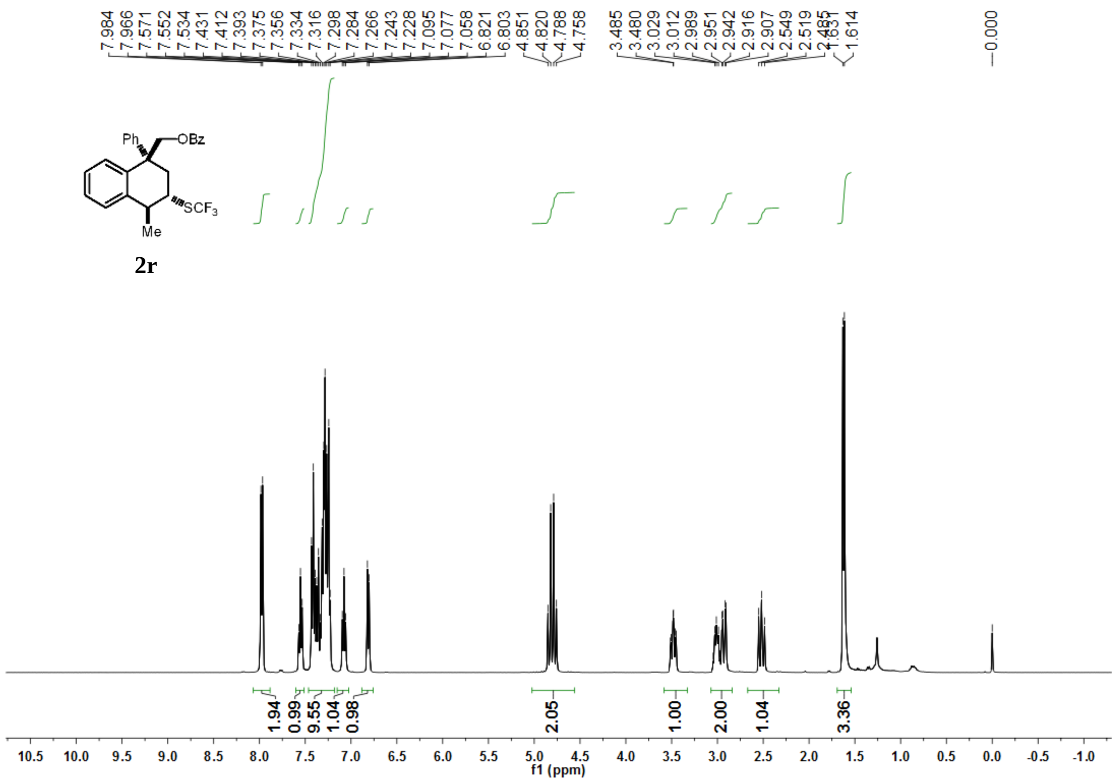

Supplementary Figure 99. ^{1}H NMR spectrum of compound **2r** in $CDCl_3$

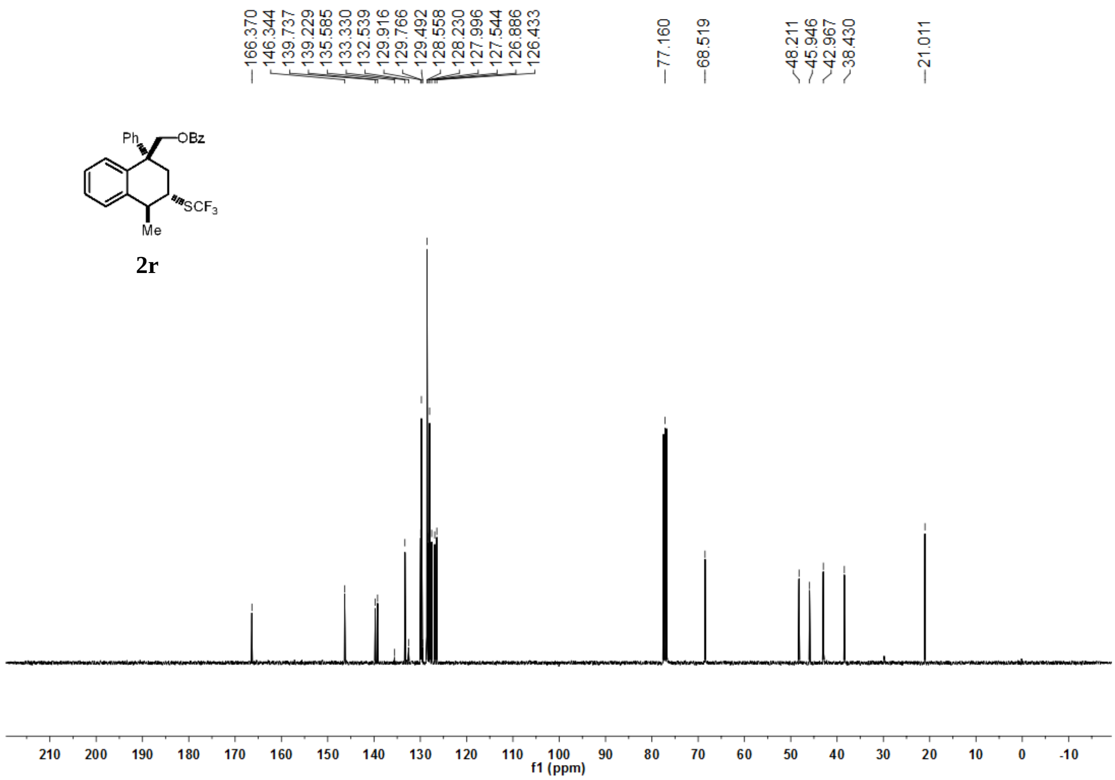

Supplementary Figure 100. ^{13}C NMR spectrum of compound **2r** in $CDCl_3$

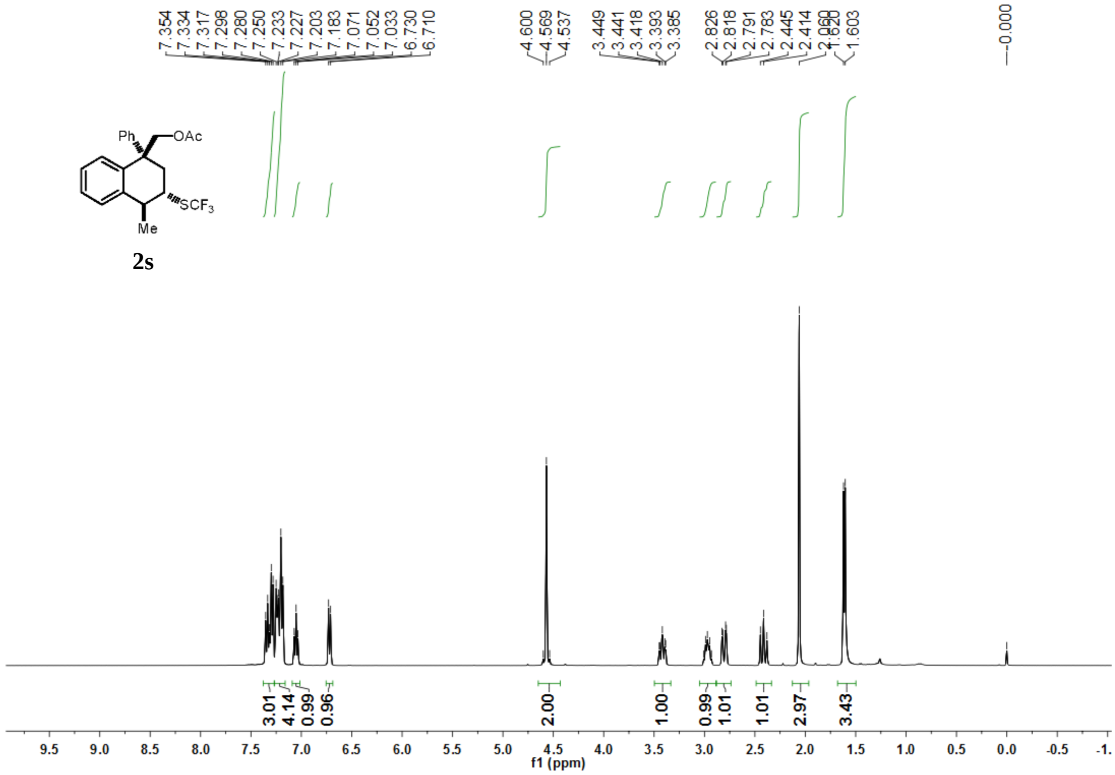

Supplementary Figure 101. ^{1}H NMR spectrum of compound **2s** in $CDCl_3$

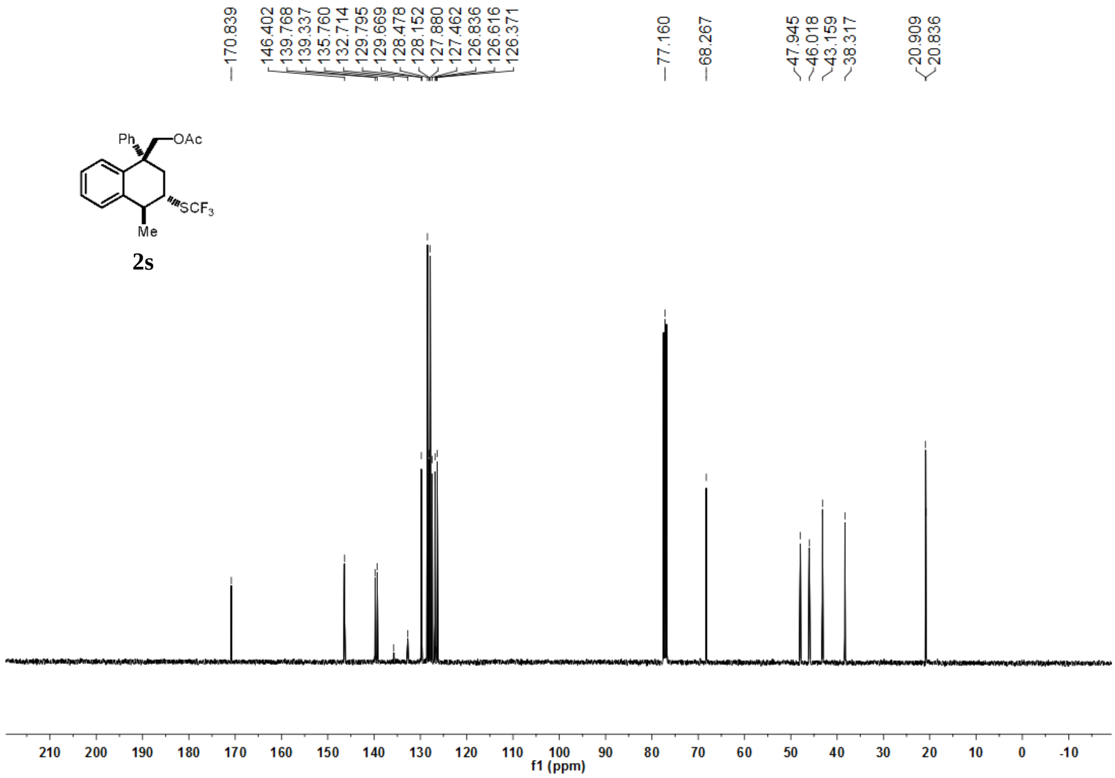

Supplementary Figure 102. ^{13}C NMR spectrum of compound **2s** in $CDCl_3$

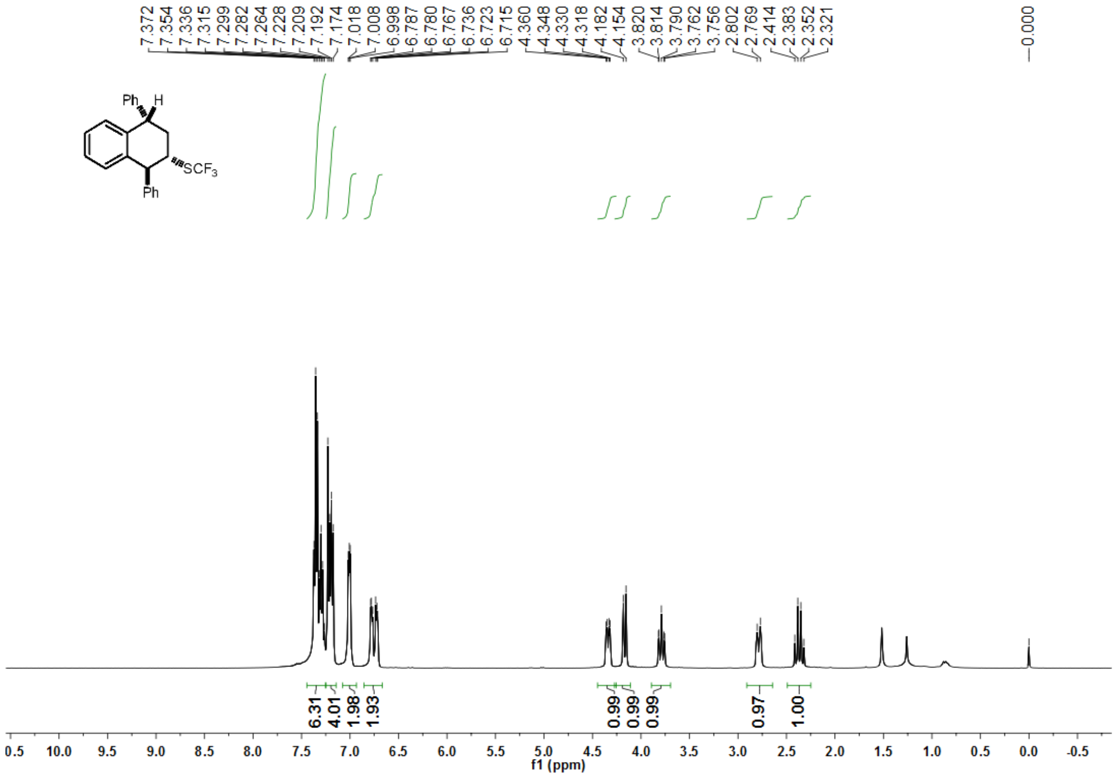

Supplementary Figure 103. ^{1}H NMR spectra of compound **2t** in $CDCl_3$

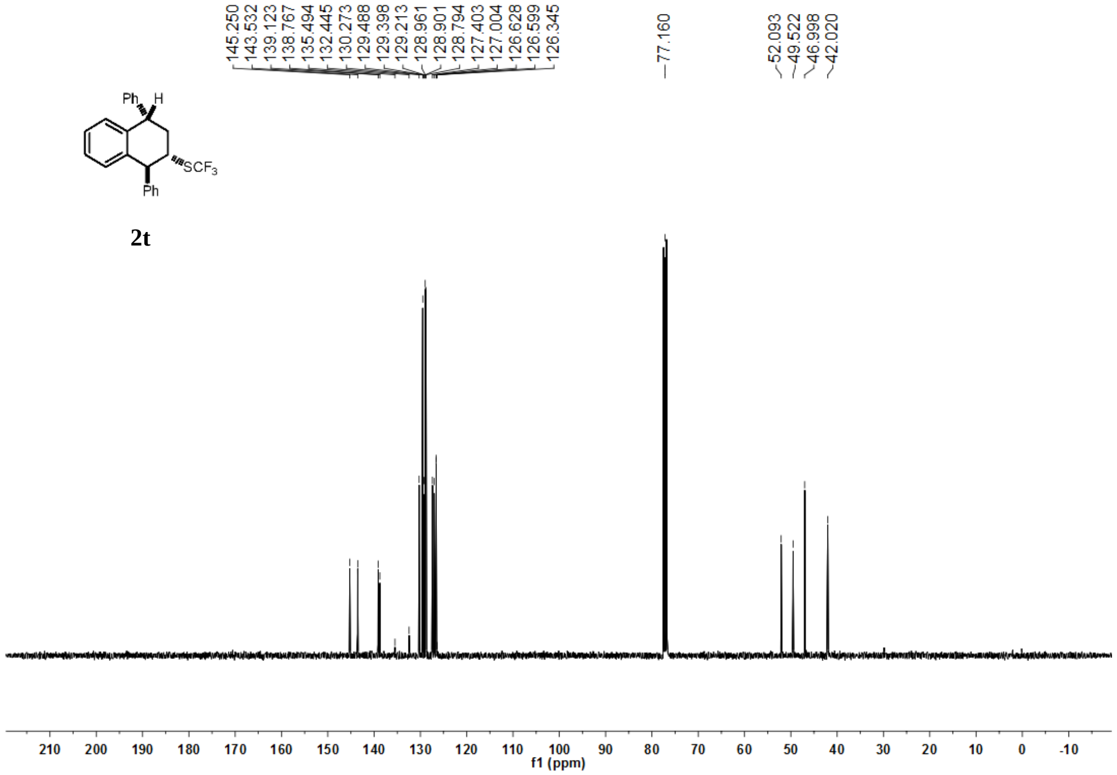

Supplementary Figure 104. ^{13}C NMR spectra of compound **2t** in $CDCl_3$

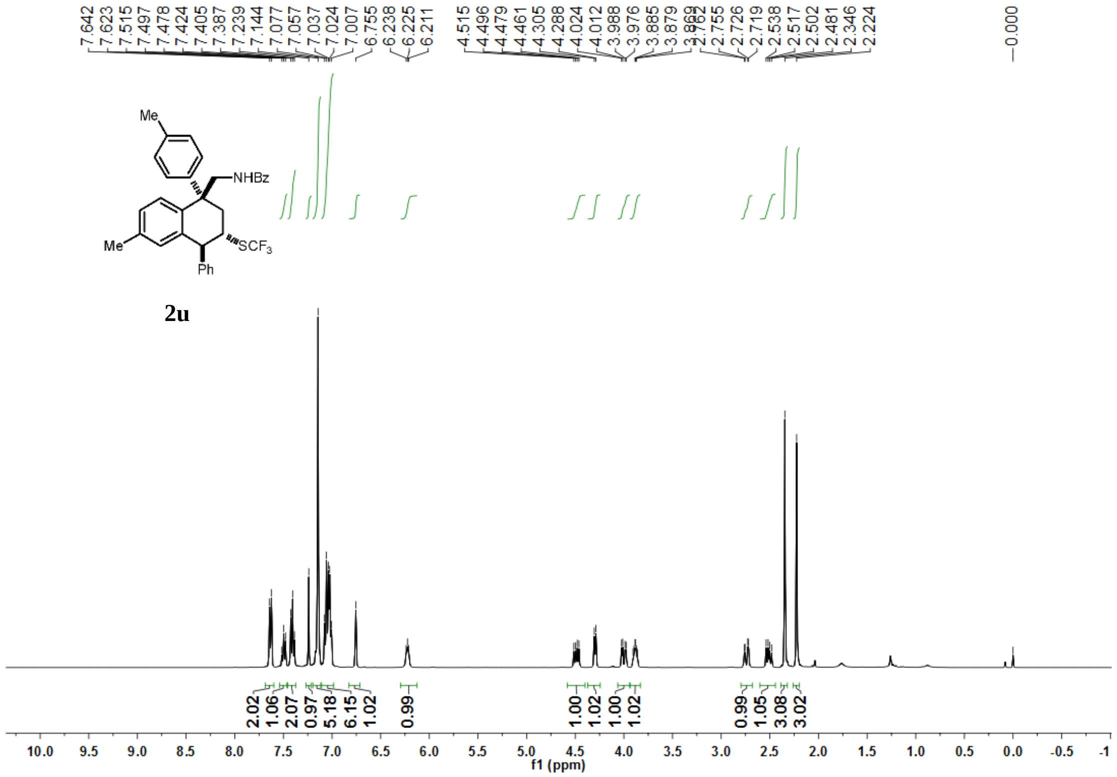

Supplementary Figure 105. ^{1}H NMR spectrum of compound **2u** in $CDCl_3$

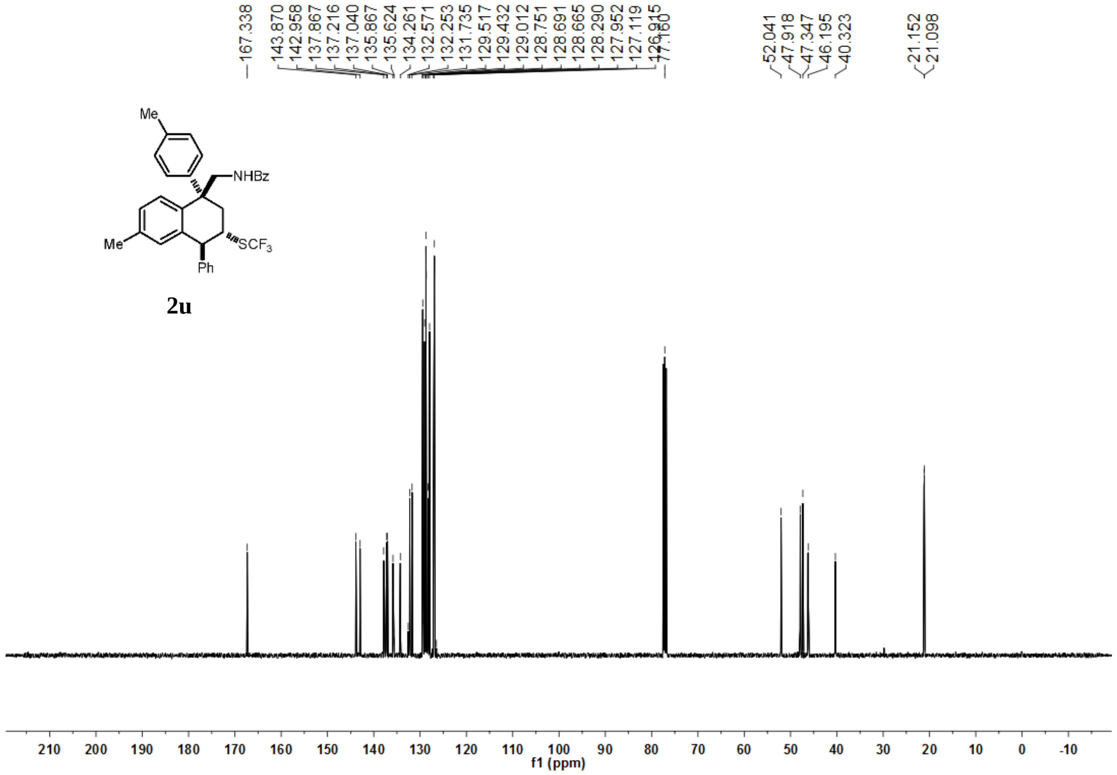

Supplementary Figure 106. ^{13}C NMR spectrum of compound **2u** in $CDCl_3$

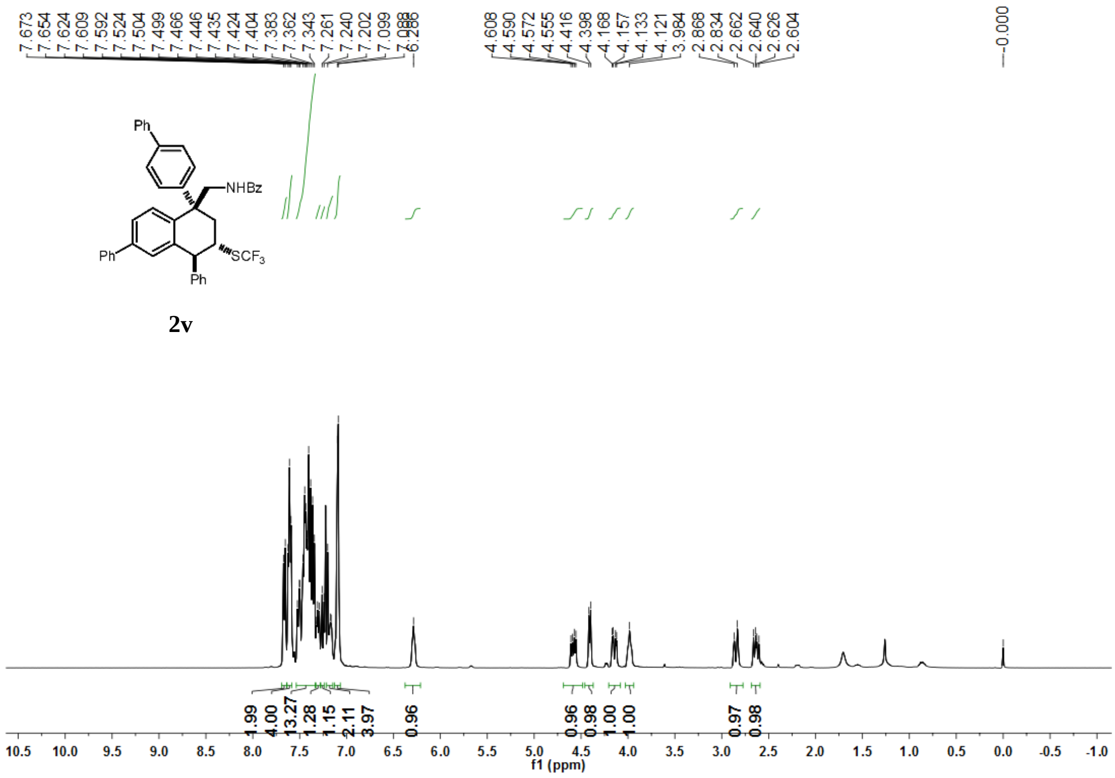

Supplementary Figure 107. ^{1}H NMR spectrum of compound **2v** in $CDCl_3$

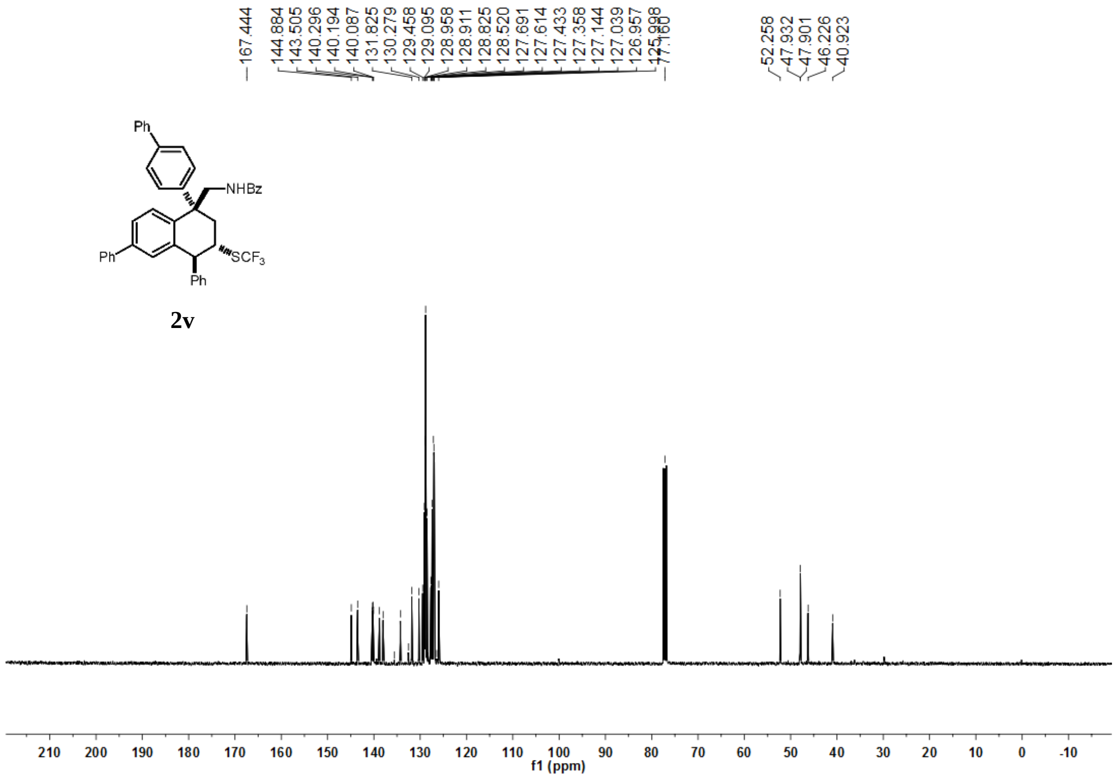

Supplementary Figure 108. ^{13}C NMR spectrum of compound **2v** in $CDCl_3$

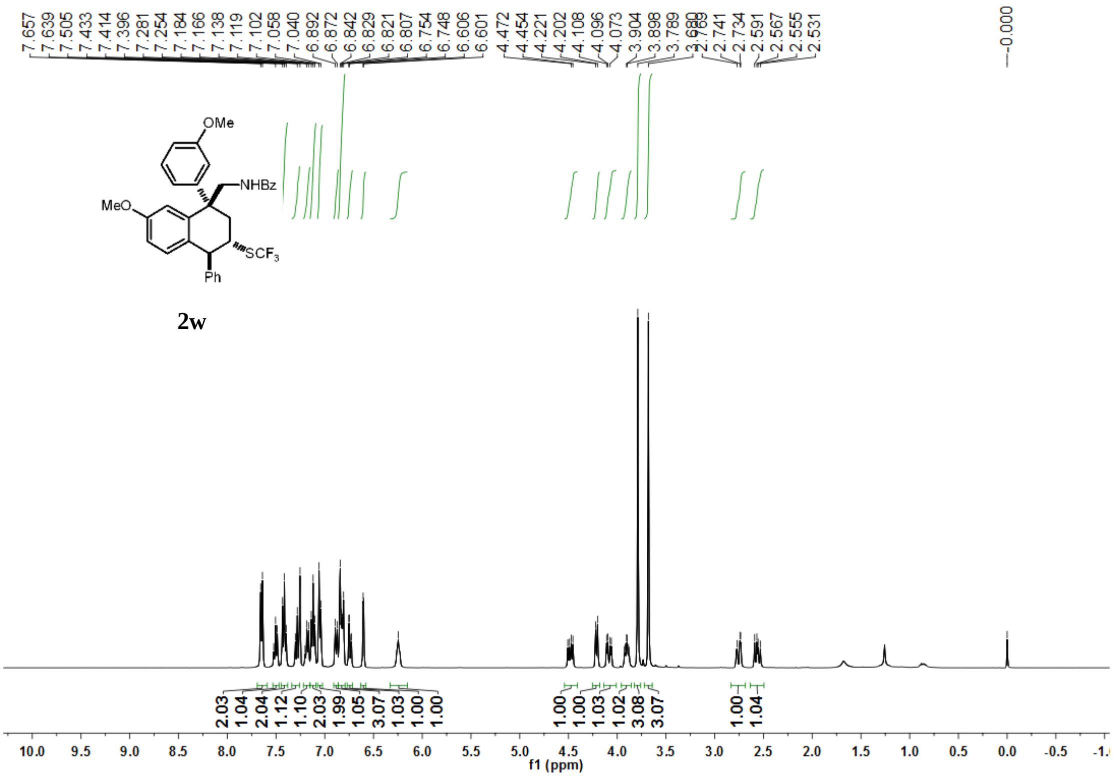

Supplementary Figure 109. ^{1}H NMR spectrum of compound **2w** in $CDCl_3$

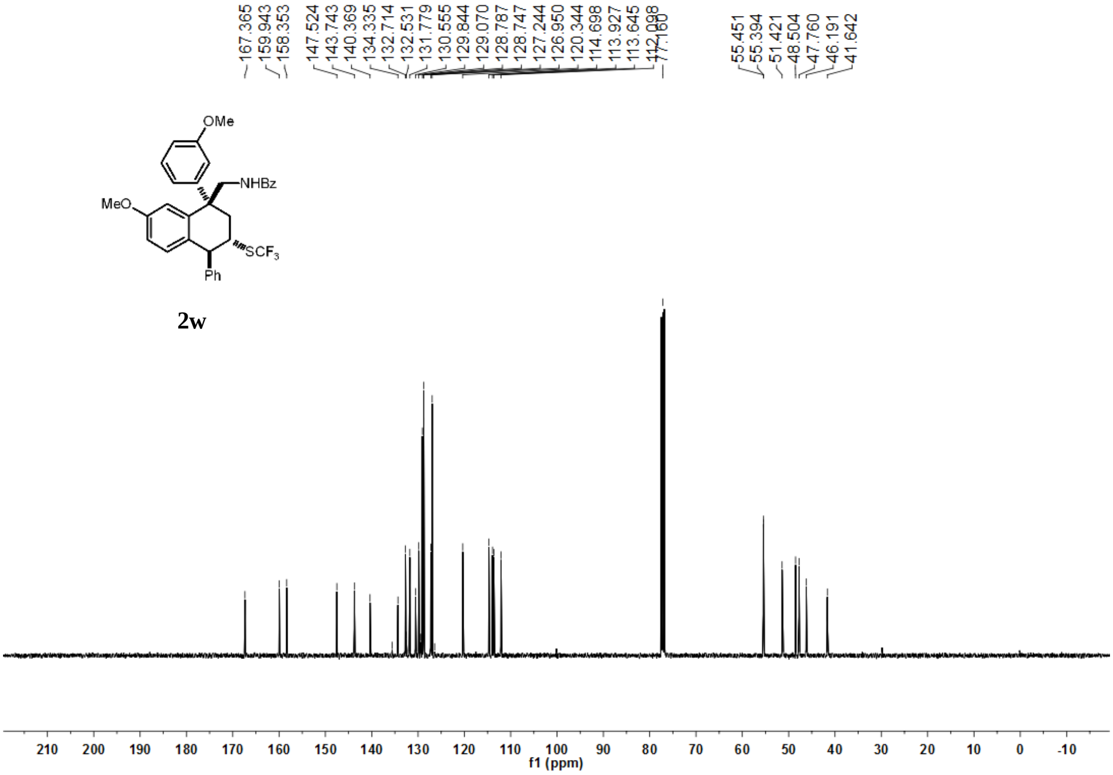

Supplementary Figure 110. ^{13}C NMR spectrum of compound **2w** in $CDCl_3$

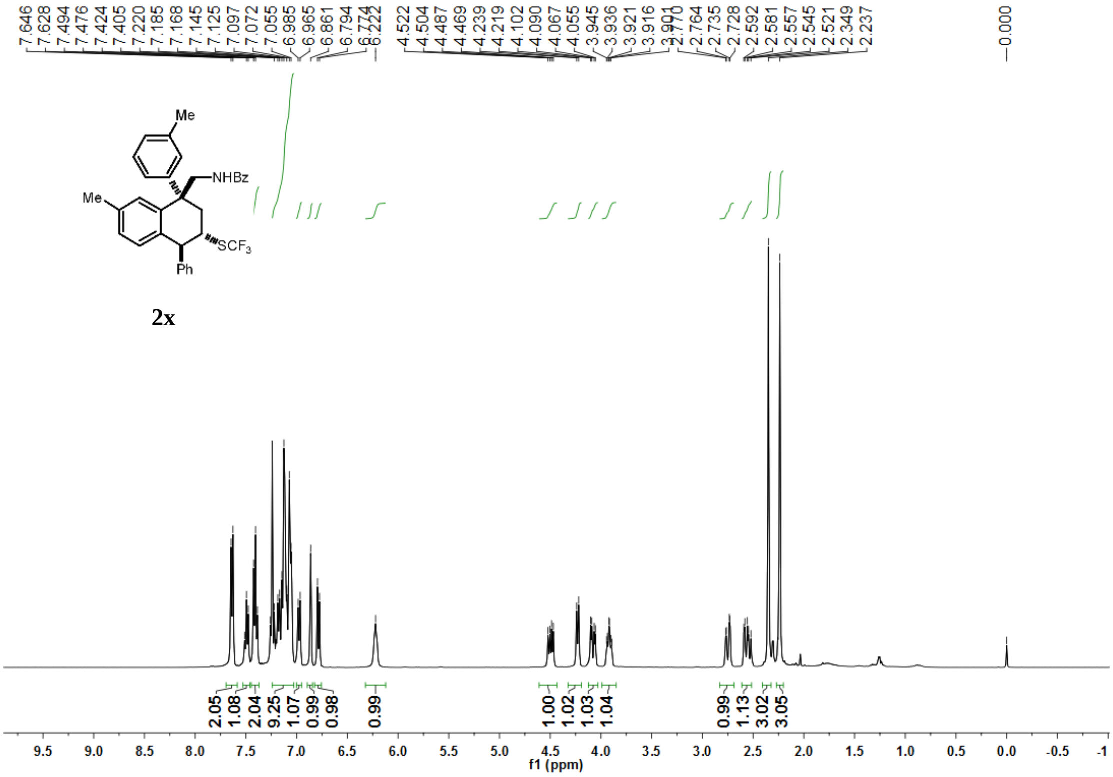

Supplementary Figure 111. ^{1}H NMR spectrum of compound **2x** in $CDCl_3$

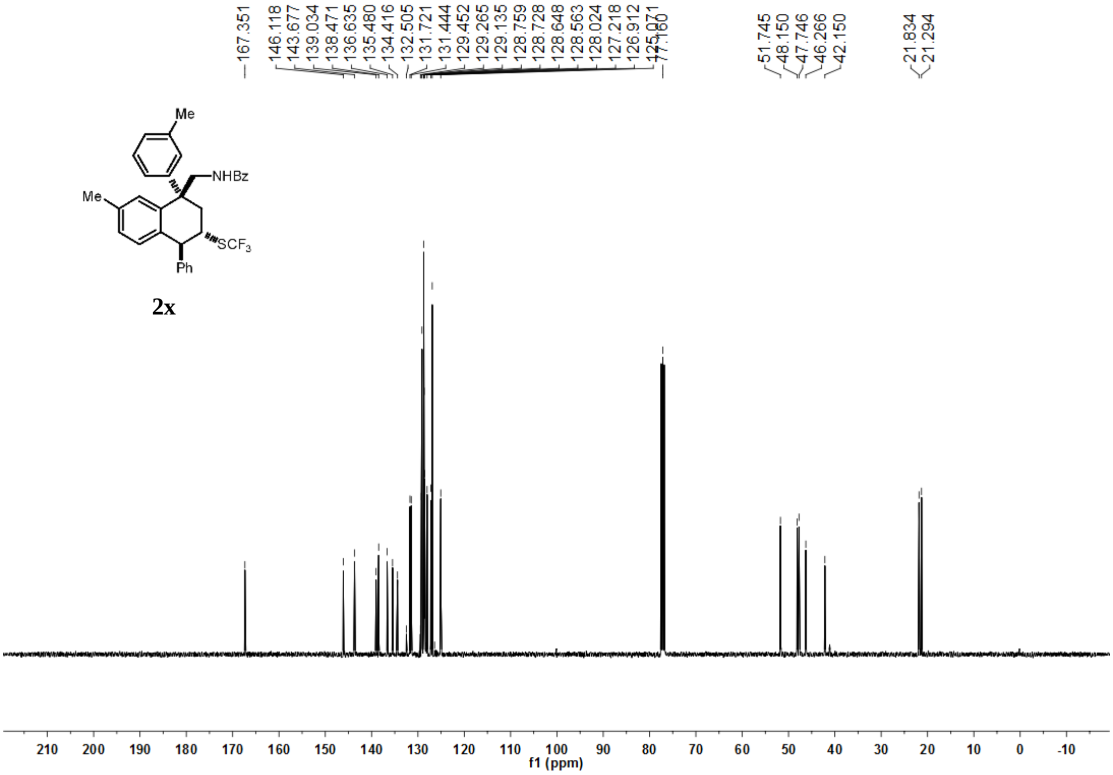

Supplementary Figure 112. ^{13}C NMR spectrum of compound **2x** in $CDCl_3$

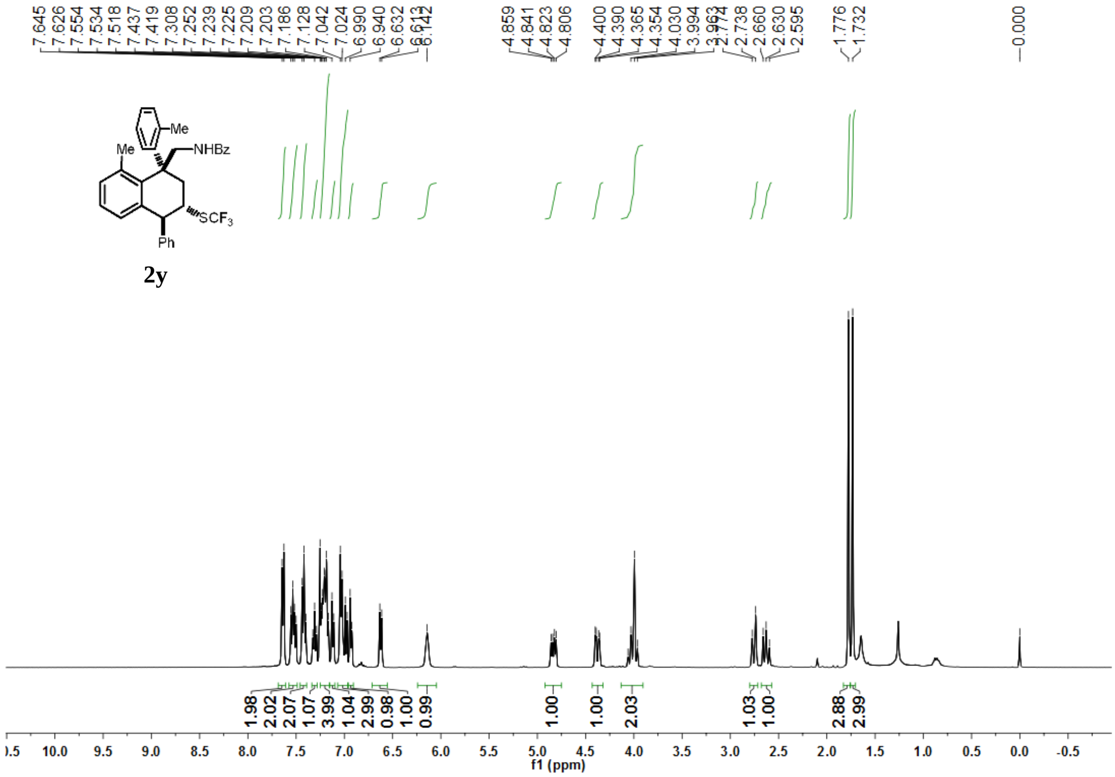

Supplementary Figure 113. ^{1}H NMR spectrum of compound **2y** in $CDCl_3$

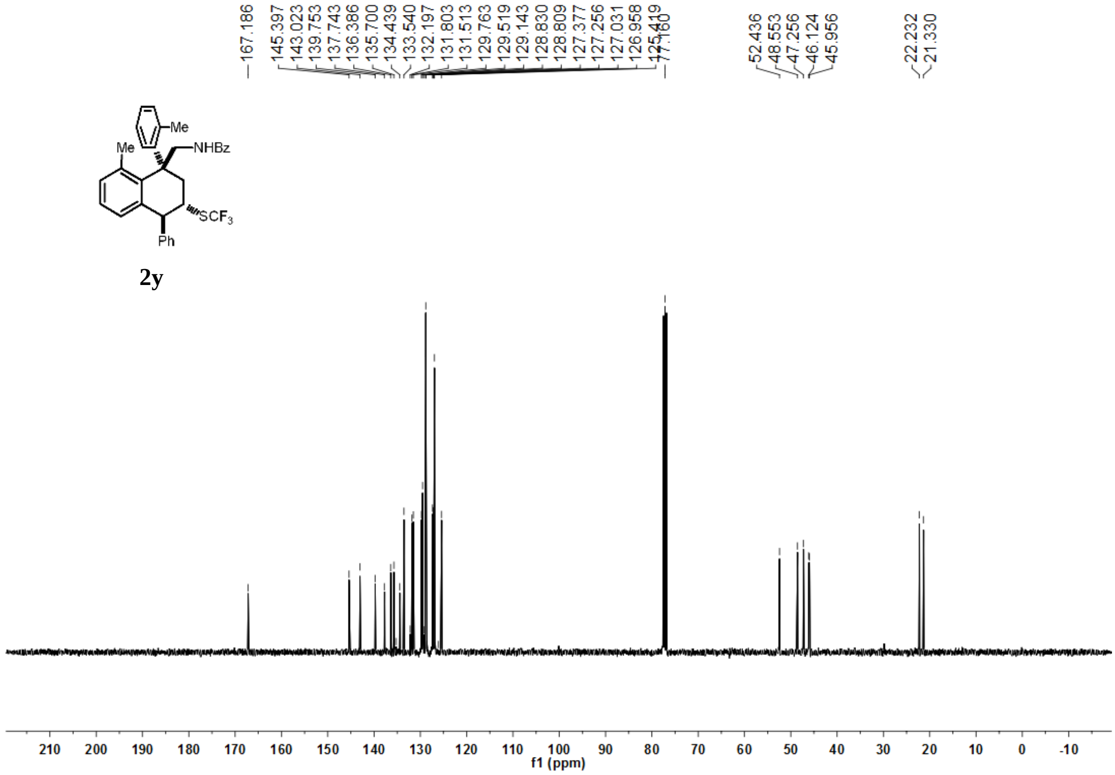

Supplementary Figure 114. ^{13}C NMR spectrum of compound **2y** in $CDCl_3$

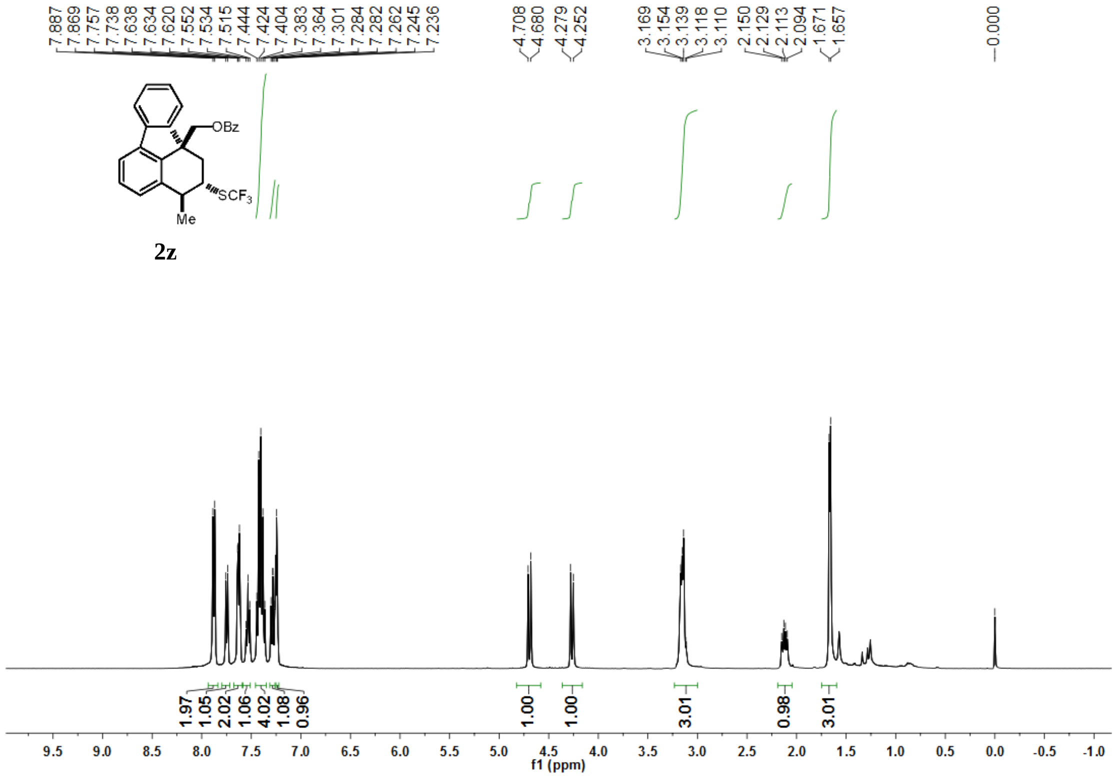

Supplementary Figure 115. ^{1}H NMR spectrum of compound **2z** in $CDCl_3$

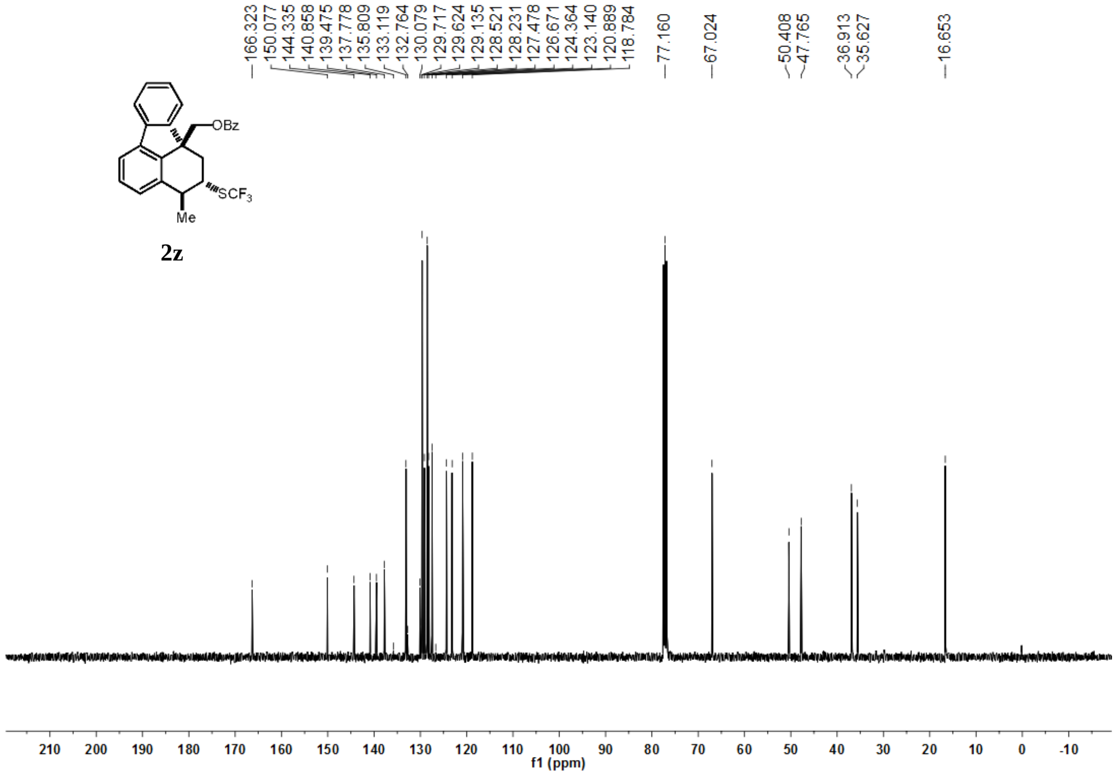

Supplementary Figure 116. ^{13}C NMR spectrum of compound **2z** in $CDCl_3$

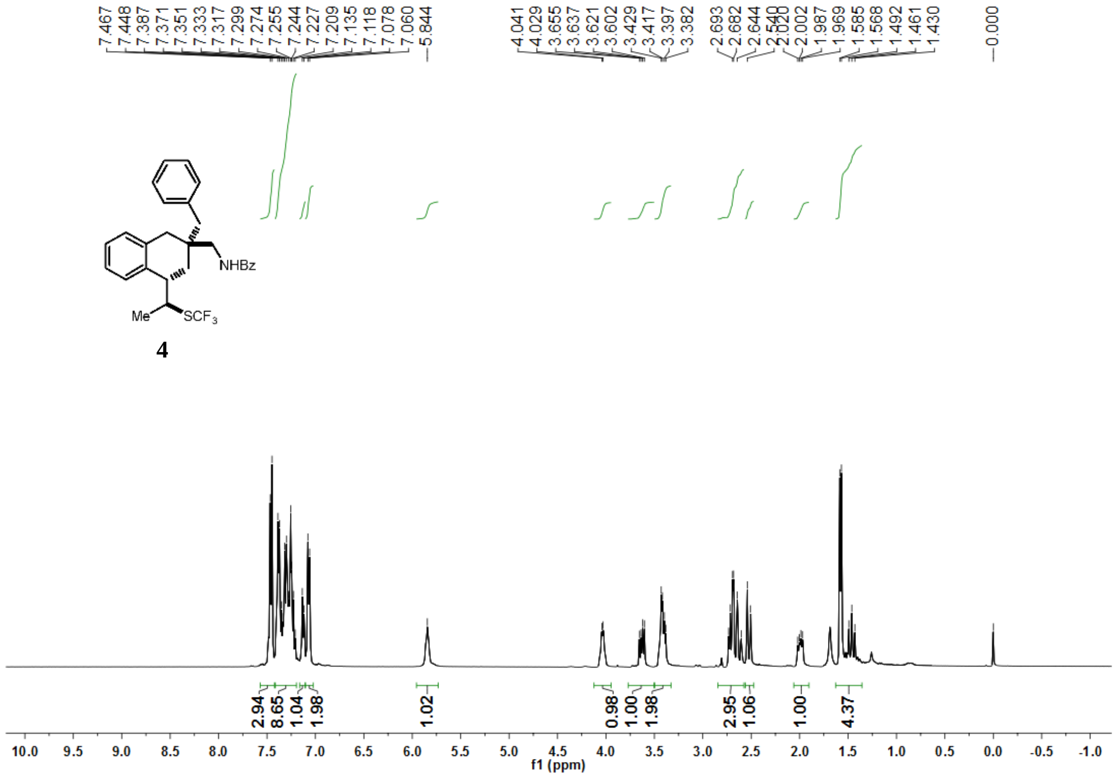

Supplementary Figure 117. ^{1}H NMR spectrum of compound **4** in $CDCl_3$

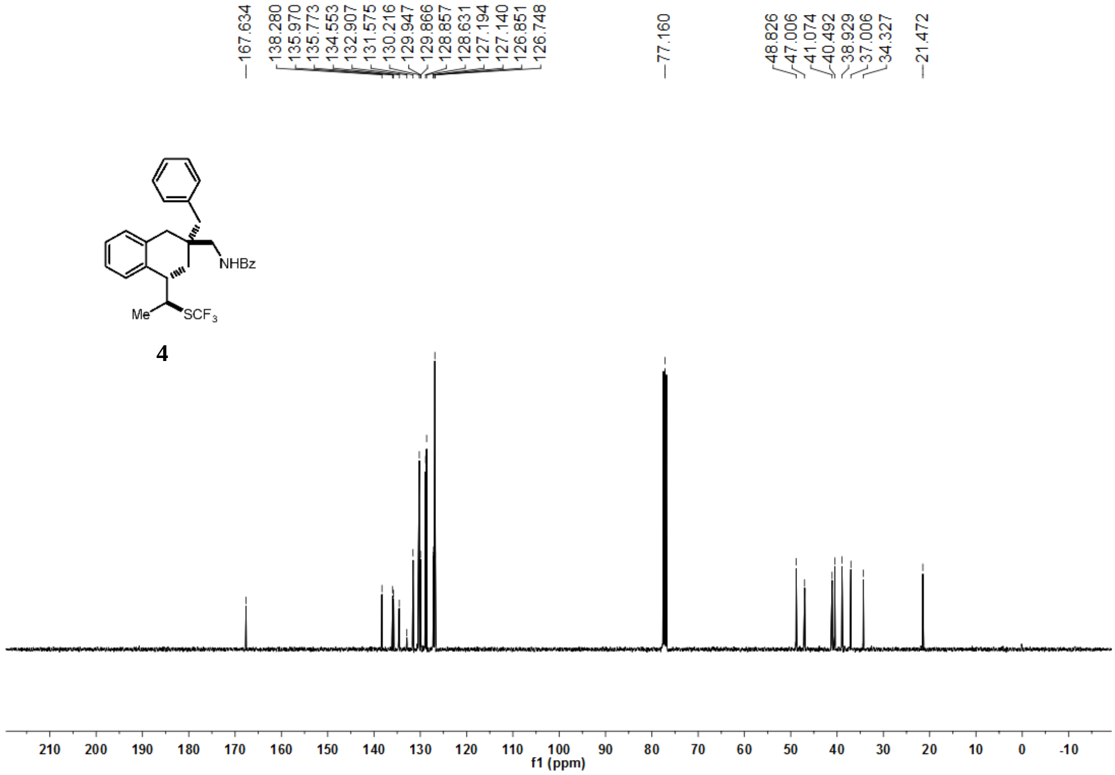

Supplementary Figure 118. ^{13}C NMR spectrum of compound **4** in $CDCl_3$

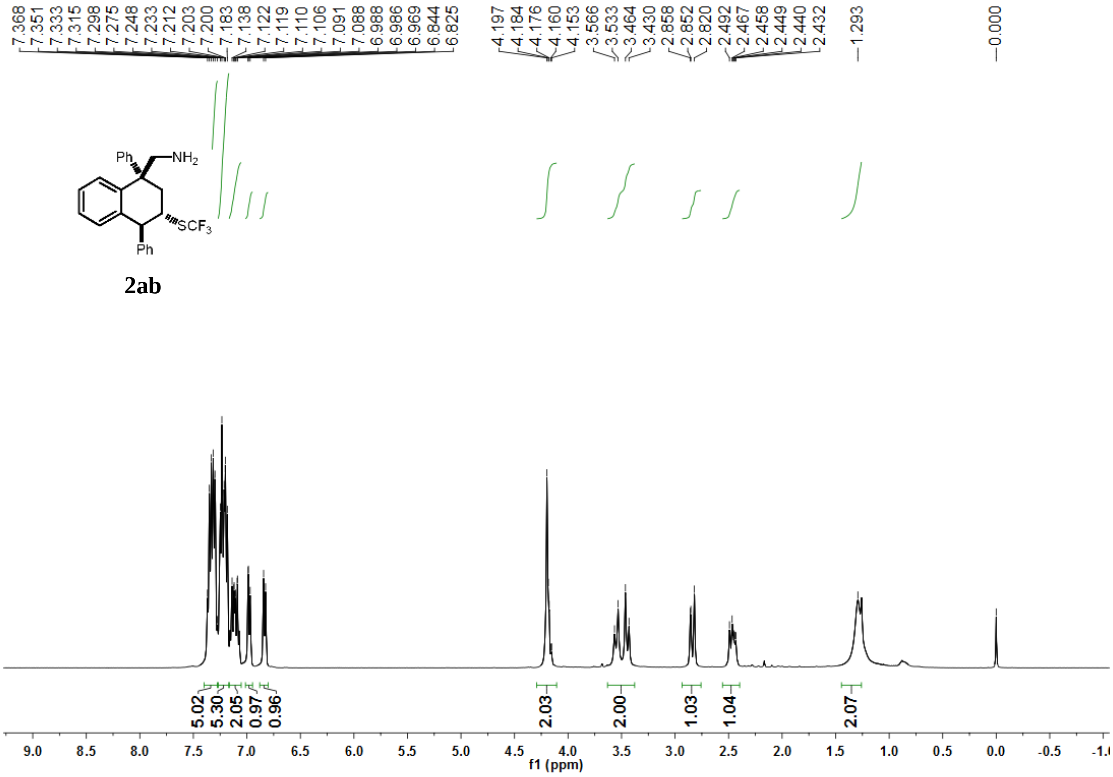

Supplementary Figure 119. ^{1}H NMR spectrum of compound **2ab** in $CDCl_3$

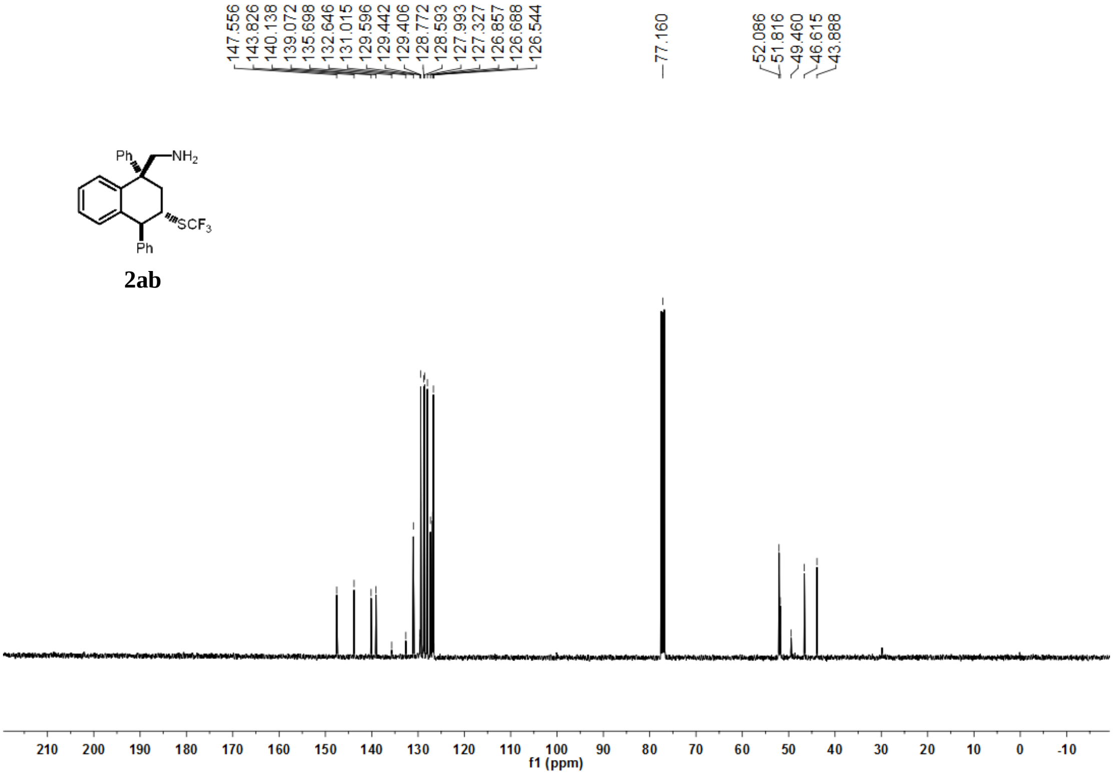

Supplementary Figure 120. ^{13}C NMR spectrum of compound **2ab** in $CDCl_3$

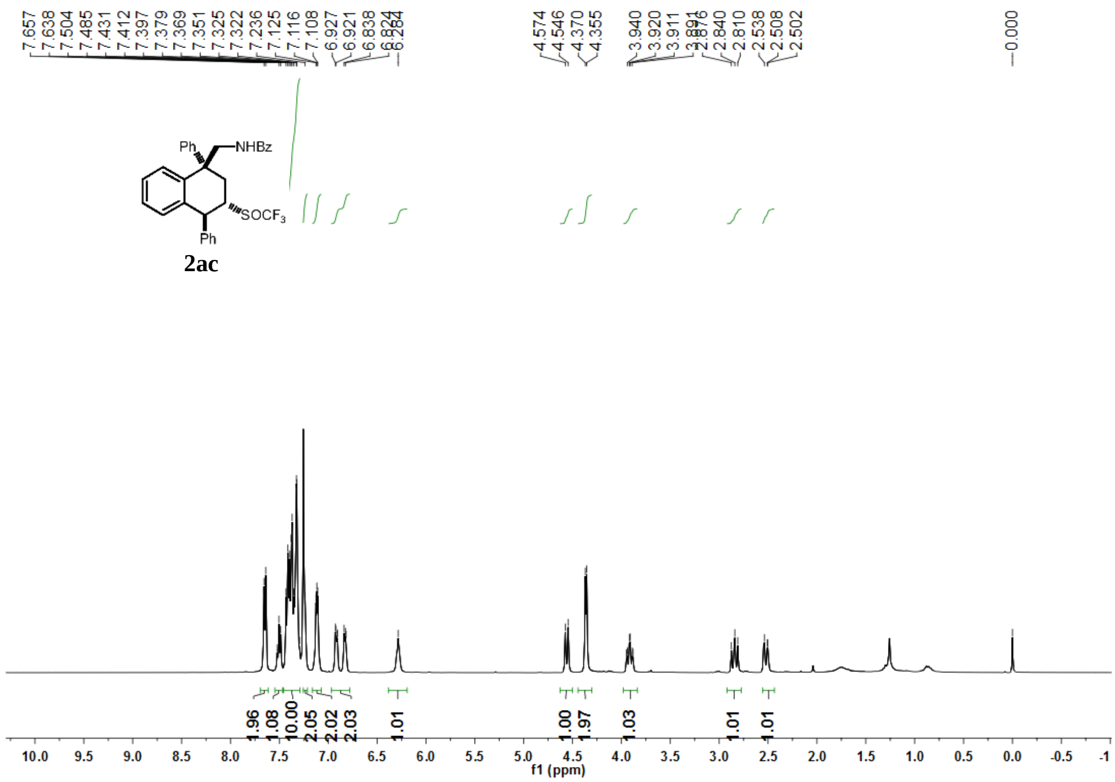

Supplementary Figure 121. ^{1}H NMR spectrum of compound **2ac** in $CDCl_3$

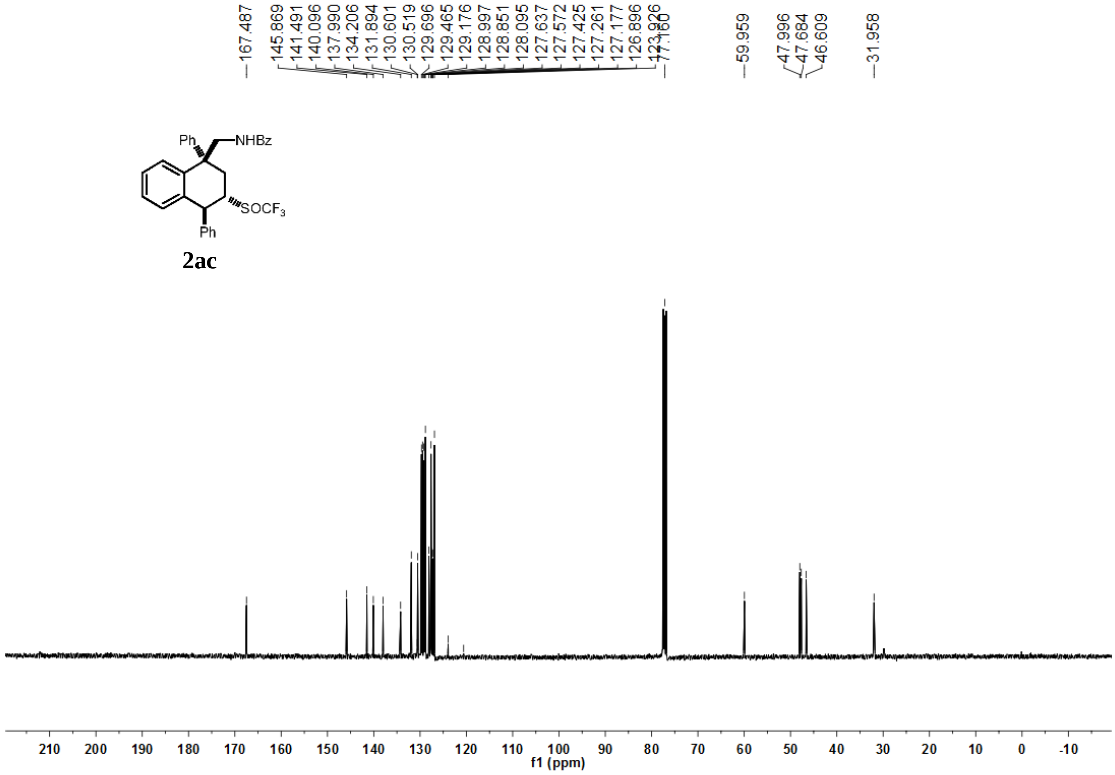

Supplementary Figure 122. ^{13}C NMR spectrum of compound **2ac** in $CDCl_3$

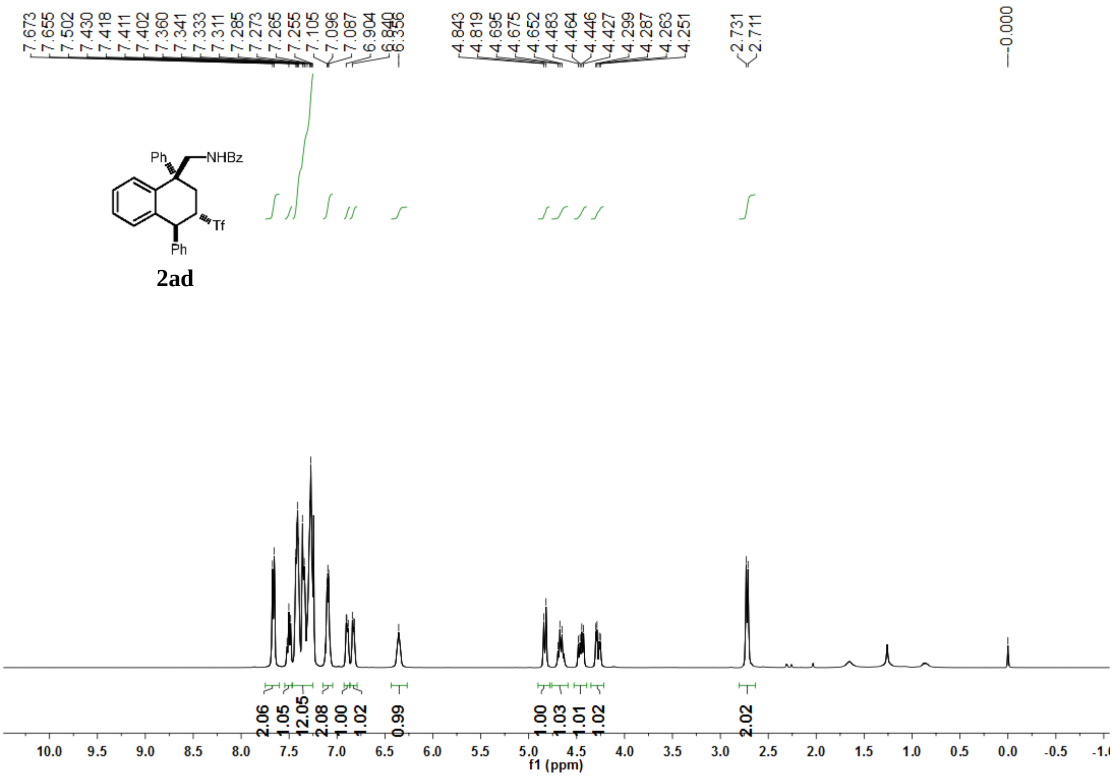

Supplementary Figure 123. ^{1}H NMR spectrum of compound **2ad** in $CDCl_3$

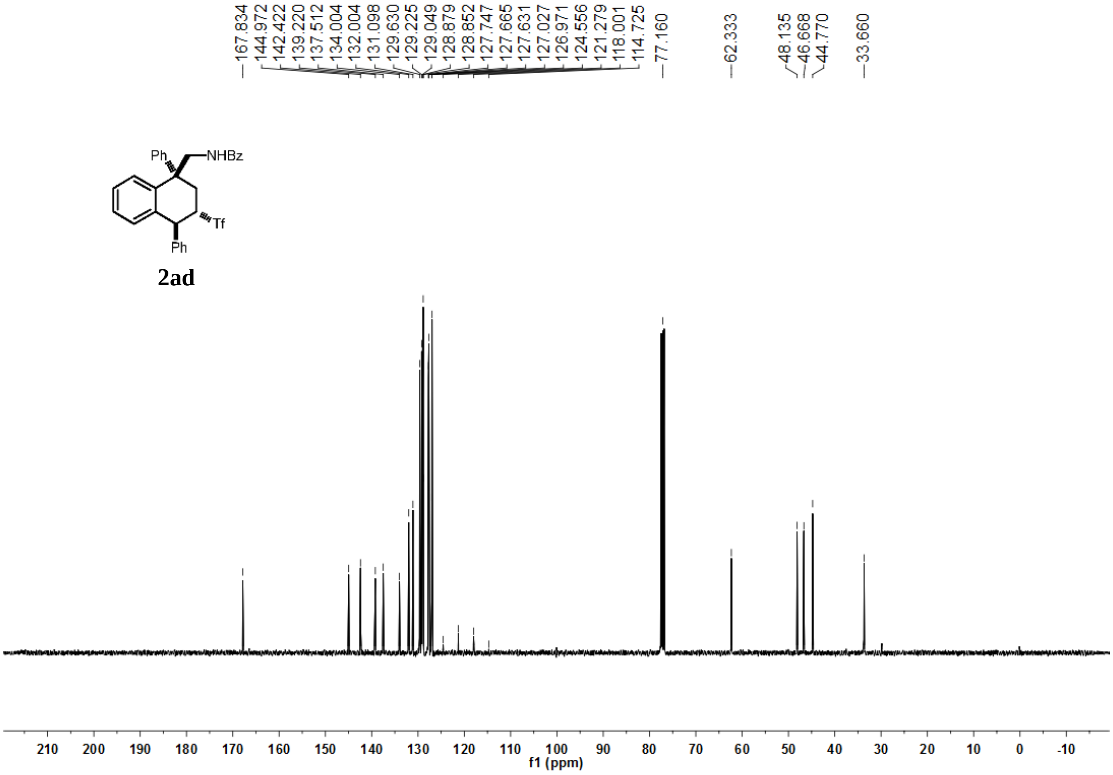

Supplementary Figure 124. ^{13}C NMR spectrum of compound **2ad** in $CDCl_3$

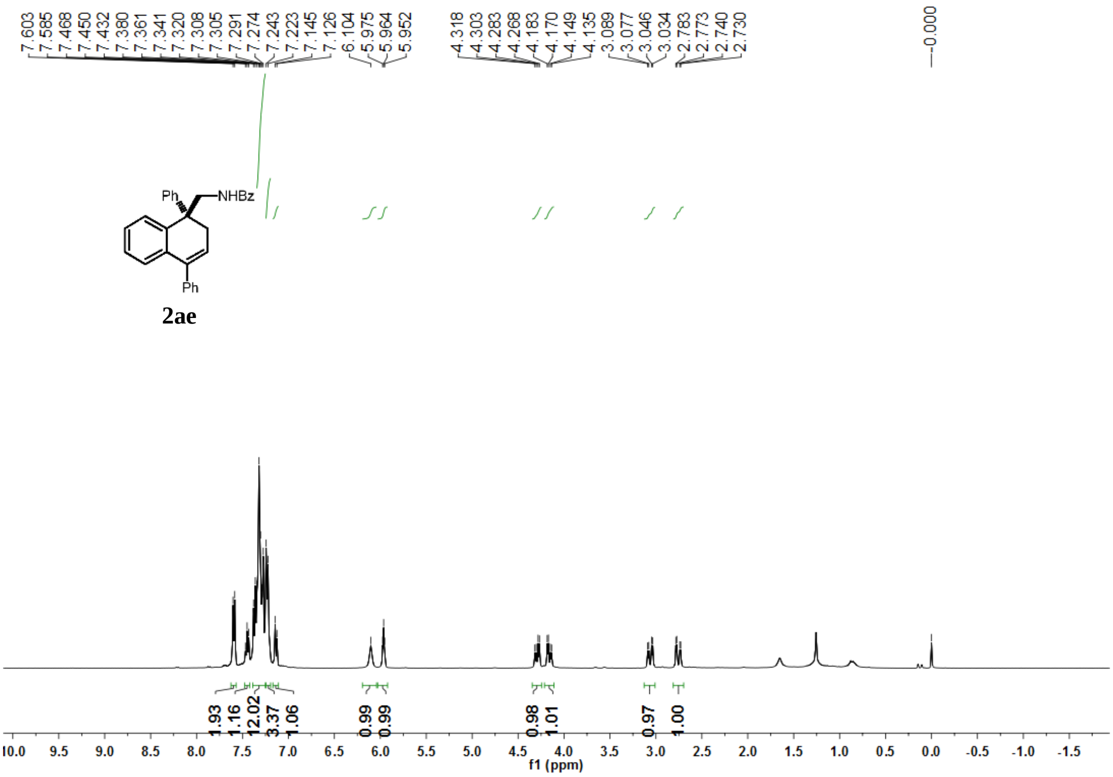

Supplementary Figure 125. ^{1}H NMR spectrum of compound **2ae** in $CDCl_3$

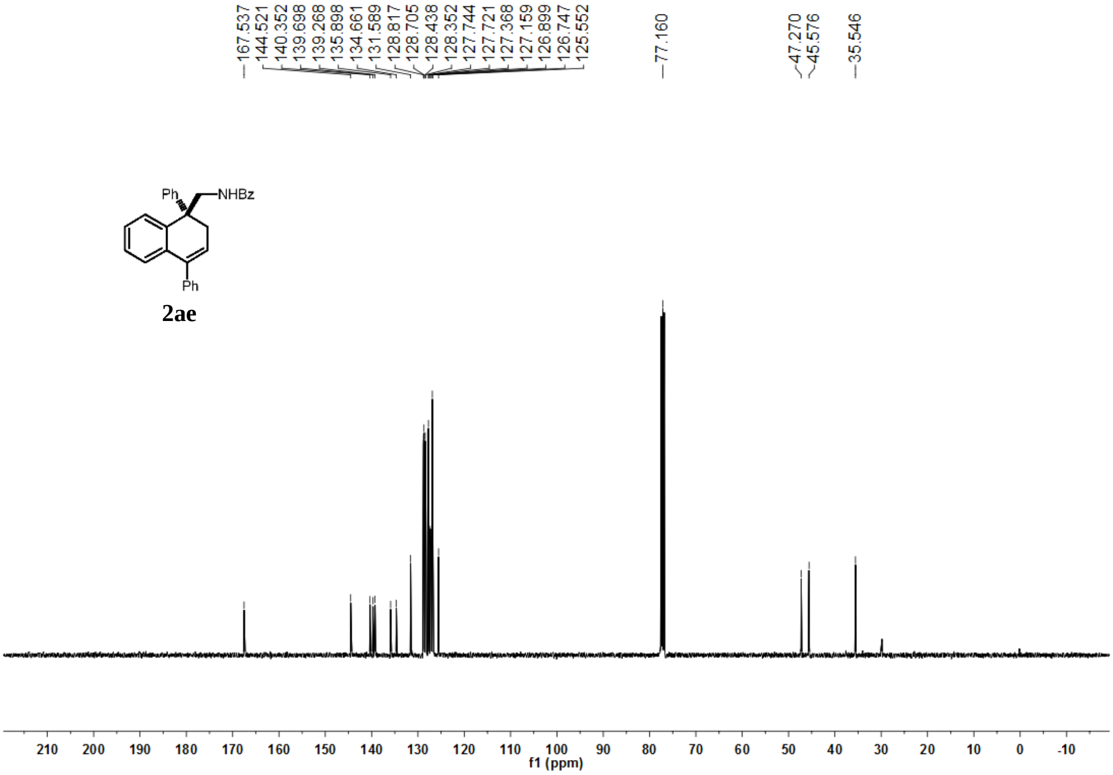

Supplementary Figure 126. ^{13}C NMR spectrum of compound **2ae** in $CDCl_3$

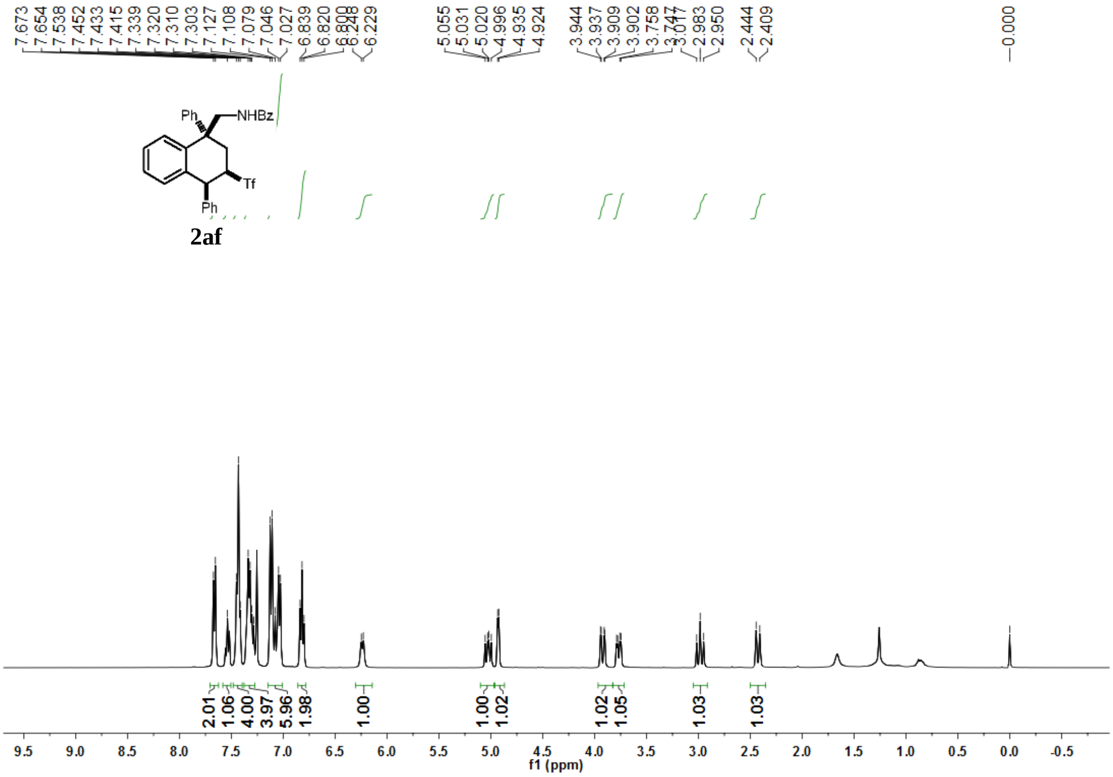

Supplementary Figure 127. ^{1}H NMR spectrum of compound **2af** in $CDCl_3$

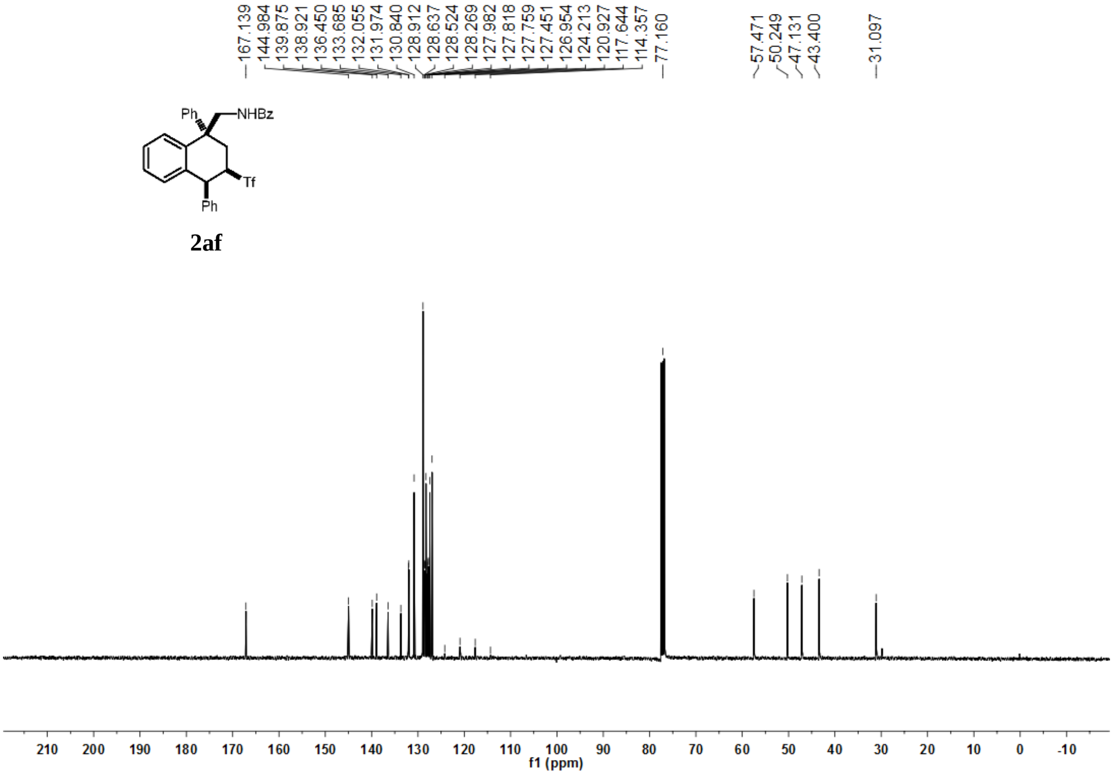

Supplementary Figure 128. ^{13}C NMR spectrum of compound **2af** in $CDCl_3$

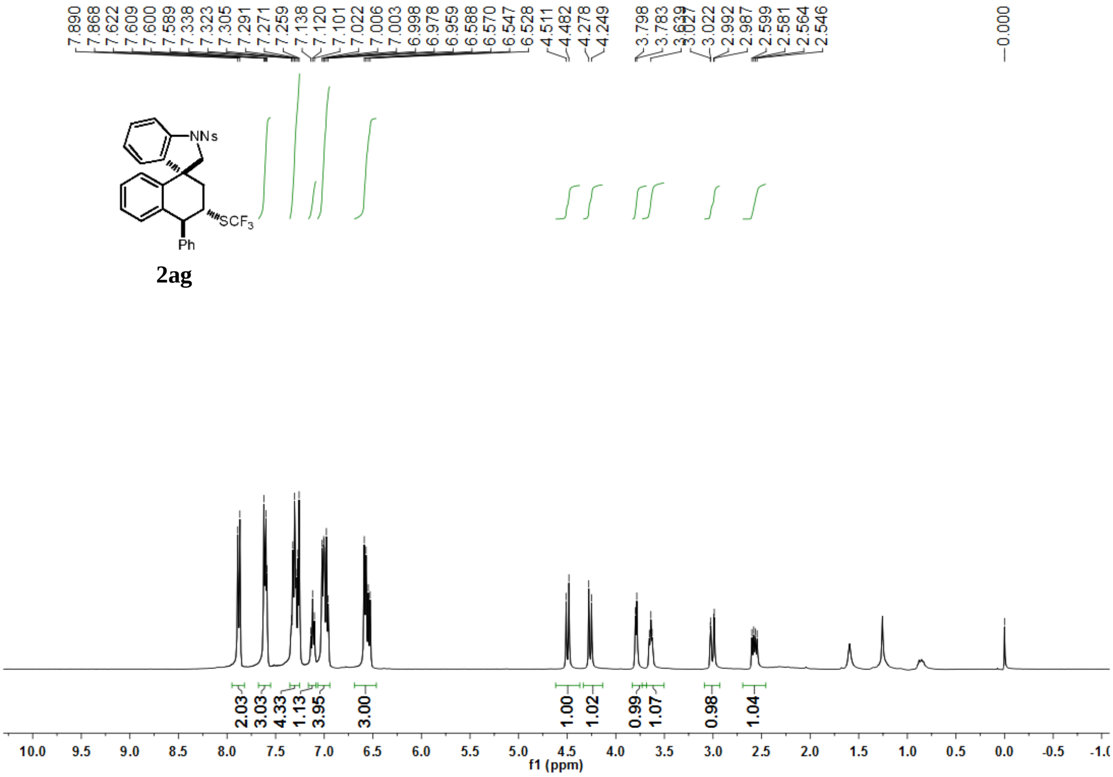

Supplementary Figure 129. ^{1}H NMR spectrum of compound **2ag** in $CDCl_3$

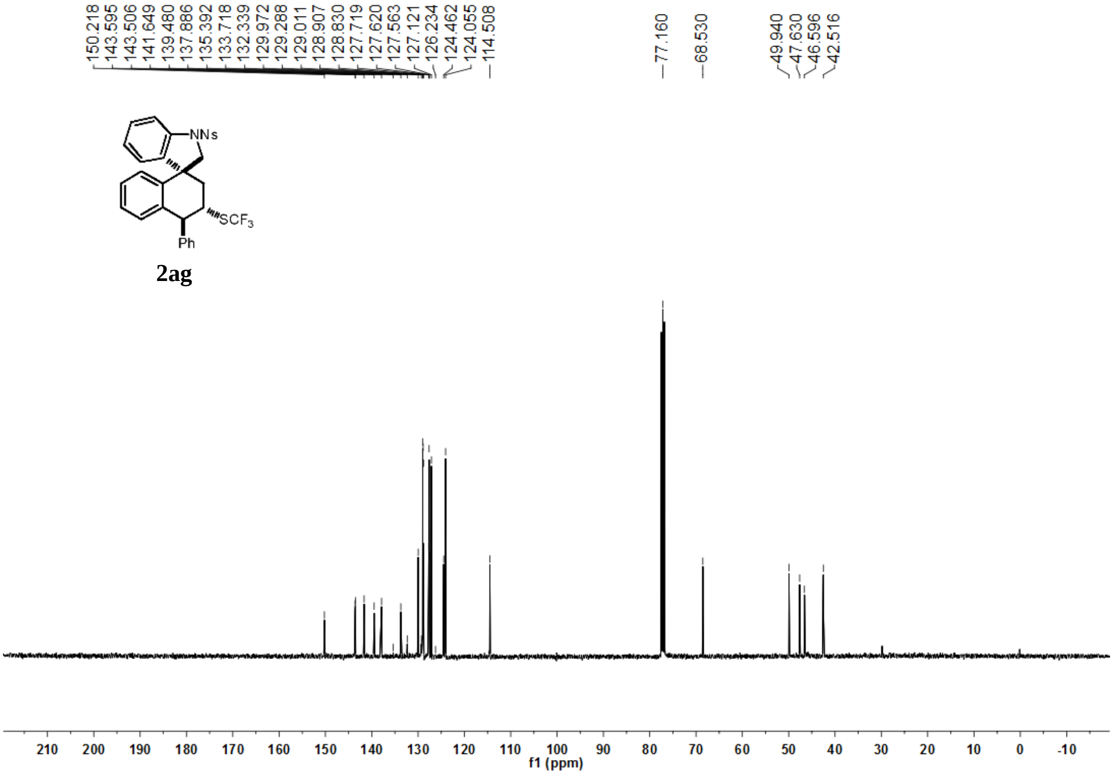

Supplementary Figure 130. ^{13}C NMR spectrum of compound **2ag** in $CDCl_3$

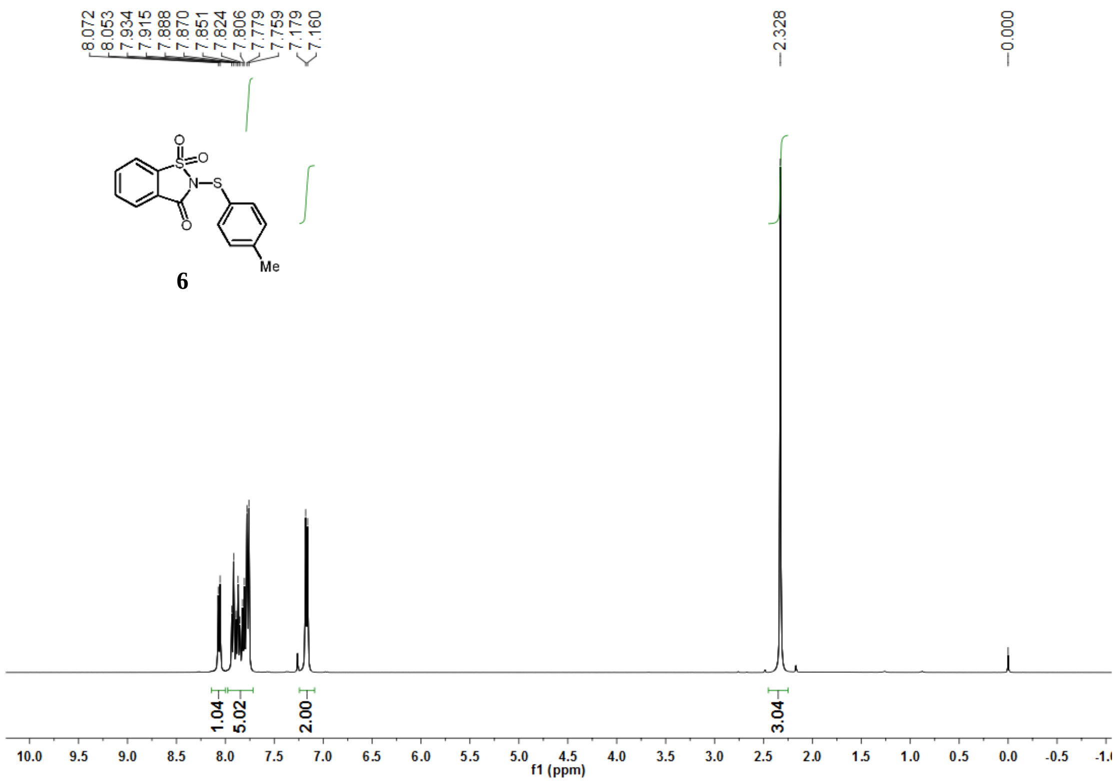

Supplementary Figure 131. ^{1}H NMR spectrum of compound **6** in $CDCl_3$

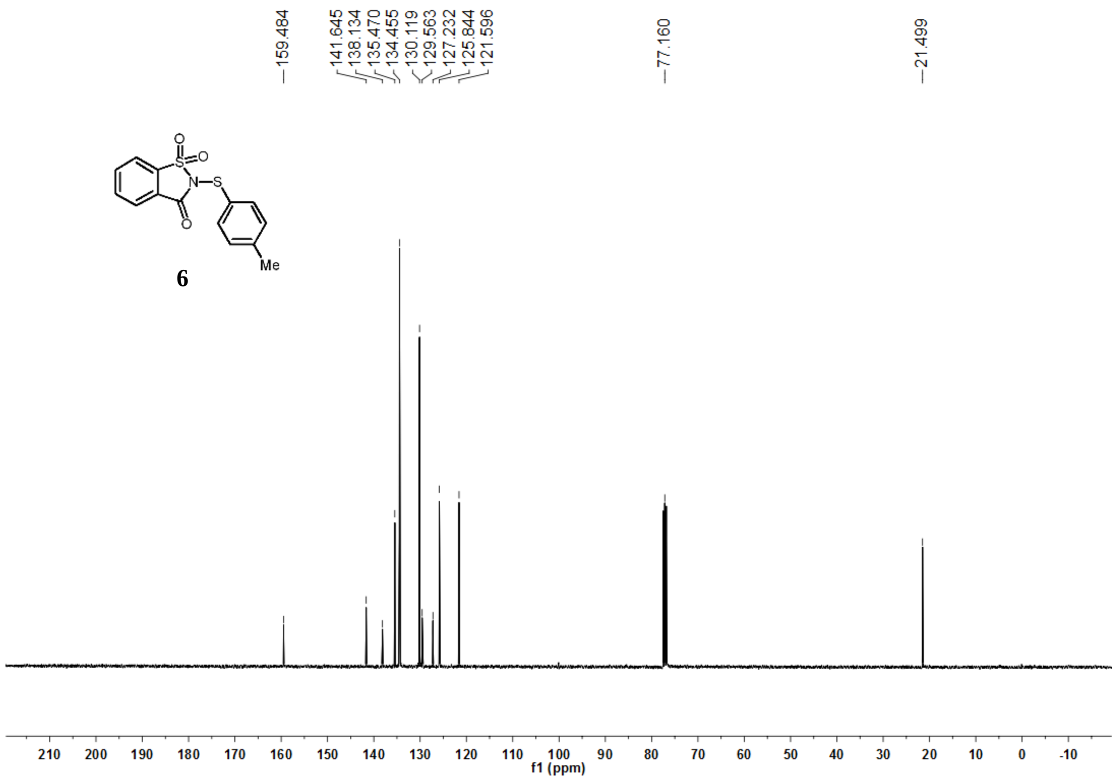

Supplementary Figure 132. ^{13}C NMR spectrum of compound **6** in $CDCl_3$

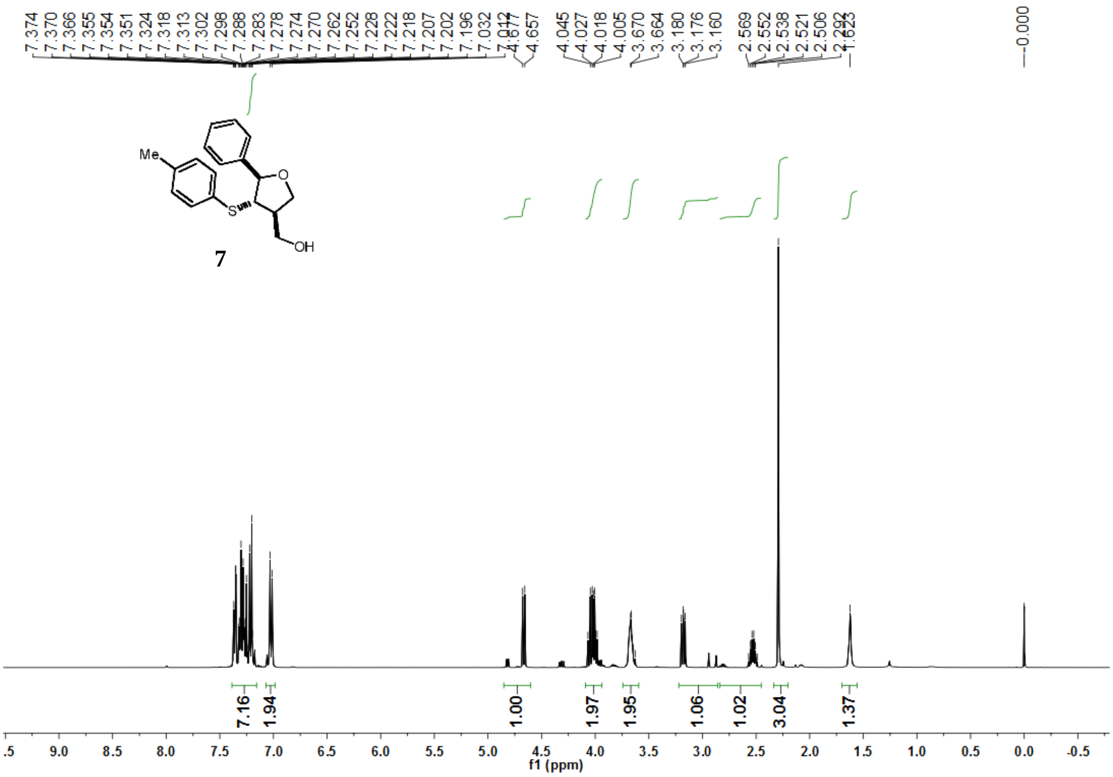

Supplementary Figure 133. ^{1}H NMR spectrum of compound **7** in $CDCl_3$

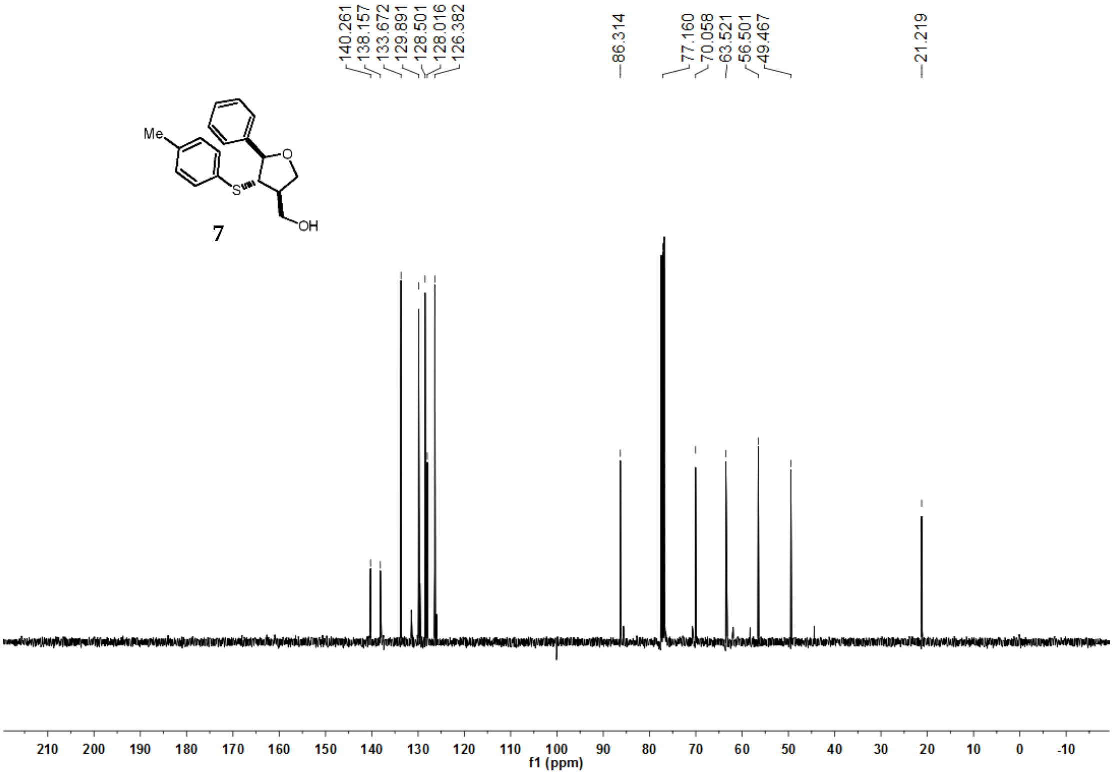

Supplementary Figure 134. ^{13}C NMR spectrum of compound **7** in $CDCl_3$

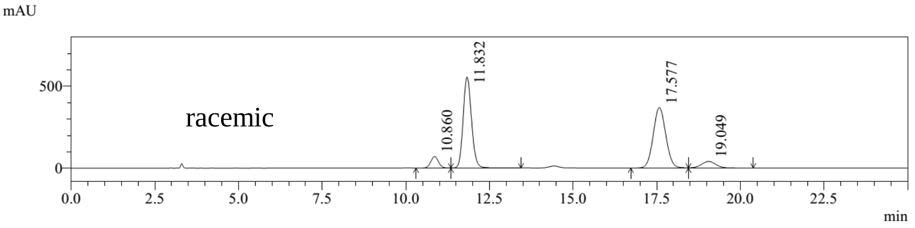

Peak Table

PDA Ch1 222nm

Peak#	Ret. Time	Area	Height	Area%	Height%
1	10.860	1152666	69084	5.323	6.807
2	11.832	9687387	542955	44.735	53.498
3	17.577	9625359	363958	44.448	35.861
4	19.049	1189751	38916	5.494	3.834
Total		21655164	1014913	100.000	100.000

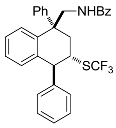

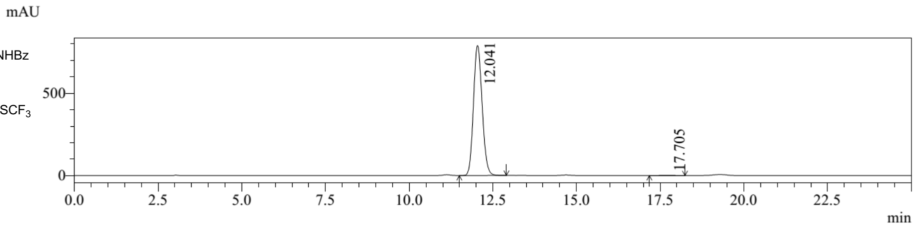

Peak Table

PDA Ch1 222nm

Peak#	Ret. Time	Area	Height	Area%	Height%
1	12.041	14262156	790594	99.632	99.741
2	17.705	52686	2056	0.368	0.259
Total		14314843	792650	100.000	100.000

Supplementary Figure 135. HPLC traces for product **2a**

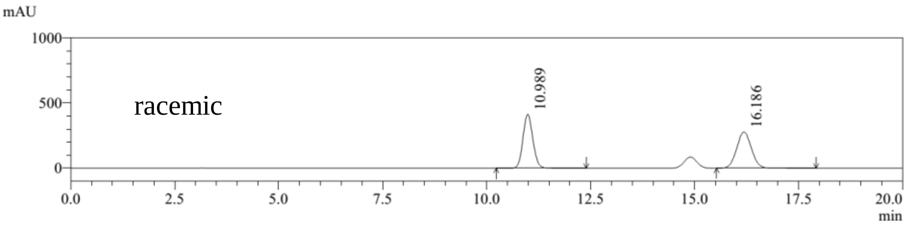

Peak Table

PDA Ch1 222nm

Peak#	Ret. Time	Area	Height	Area%	Height%
1	10.989	6877014	389057	49.971	58.652
2	16.186	6885062	274277	50.029	41.348
Total		13762076	663334	100.000	100.000

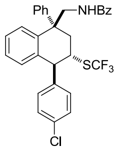

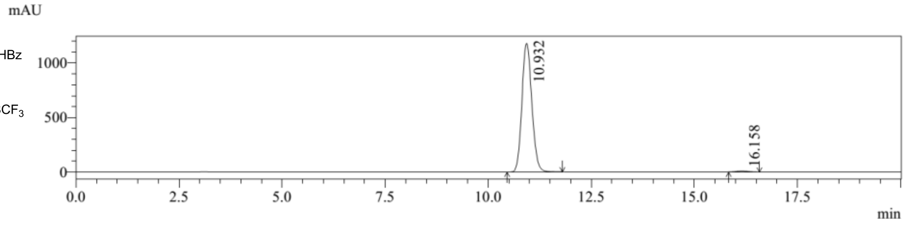

Peak Table

PDA Ch1 222nm

Peak#	Ret. Time	Area	Height	Area%	Height%
1	10.932	19845267	1147816	99.005	99.225
2	16.158	199409	8967	0.995	0.775
Total		20044676	1156783	100.000	100.000

Supplementary Figure 136. HPLC traces for product **2b**

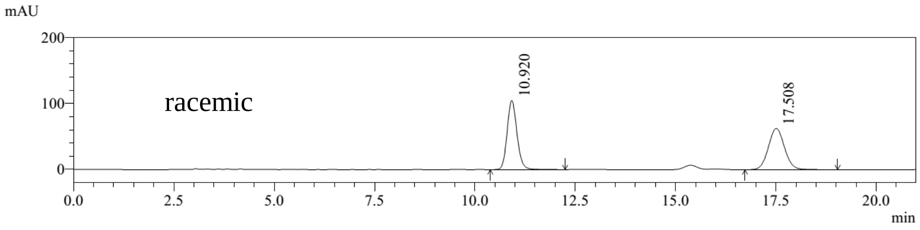

Peak Table

PDA Ch1 240nm

Peak#	Ret. Time	Area	Height	Area%	Height%
1	10.920	1793520	99864	50.555	61.684
2	17.508	1754147	62033	49.445	38.316
Total		3547667	161897	100.000	100.000

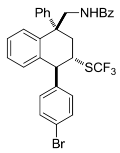

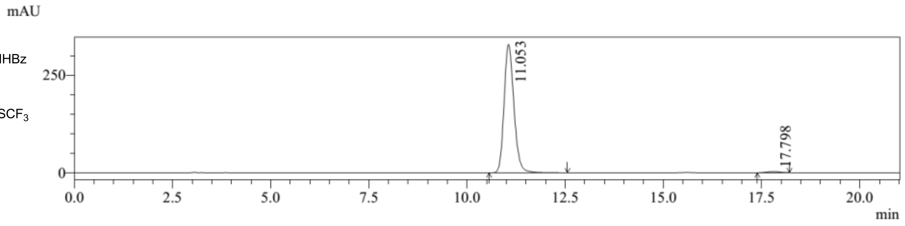

Peak Table

PDA Ch1 240nm

Peak#	Ret. Time	Area	Height	Area%	Height%
1	11.053	5756793	329292	98.800	99.146
2	17.798	69917	2836	1.200	0.854
Total		5826710	332128	100.000	100.000

Supplementary Figure 137. HPLC traces for product **2c**

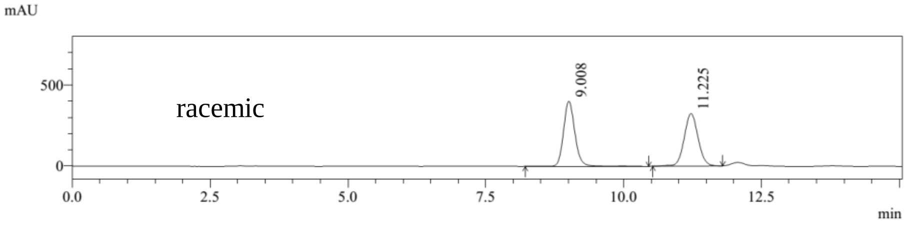

Peak Table

PDA Ch1 222nm

Peak#	Ret. Time	Area	Height	Area%	Height%
1	9.008	5731375	390104	50.233	55.484
2	11.225	5678191	312983	49.767	44.516
Total		11409566	703088	100.000	100.000

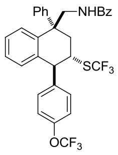

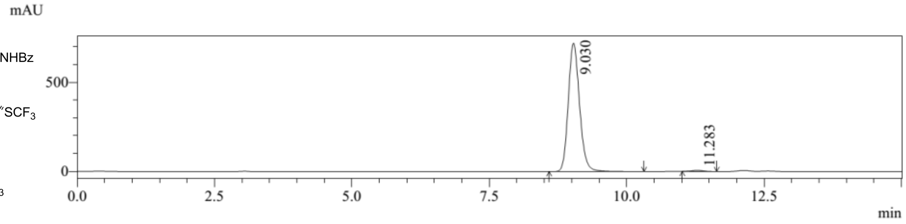

Peak Table

PDA Ch1 222nm

Peak#	Ret. Time	Area	Height	Area%	Height%
1	9.030	10300380	720157	99.195	99.257
2	11.283	83556	5392	0.805	0.743
Total		10383936	725550	100.000	100.000

Supplementary Figure 138. HPLC traces for product **2d**

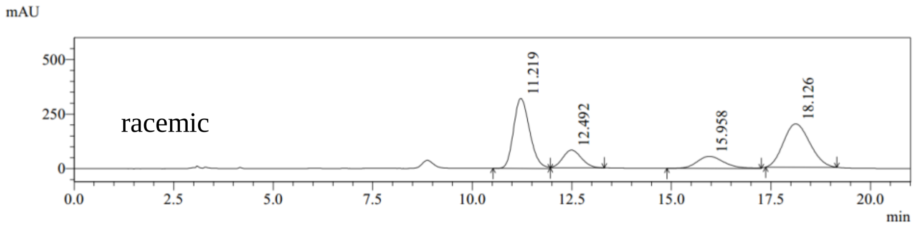

Peak Table

PDA Ch1 209nm

Peak#	Ret. Time	Area	Height	Area%	Height%
1	11.219	9057764	320927	38.477	48.849
2	12.492	2695214	81720	11.449	12.439
3	15.958	2618542	55483	11.123	8.445
4	18.126	9169453	198848	38.951	30.267
Total		23540973	656978	100.000	100.000

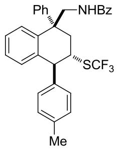

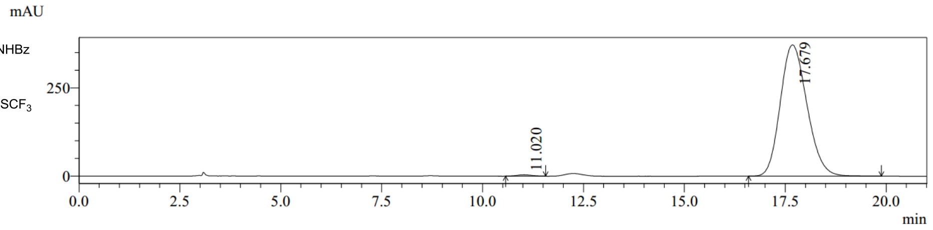

Peak Table

PDA Ch1 209nm

Peak#	Ret. Time	Area	Height	Area%	Height%
1	11.020	100804	3944	0.584	1.049
2	17.679	17157195	371997	99.416	98.951
Total		17257999	375941	100.000	100.000

Supplementary Figure 139. HPLC traces for product **2e**

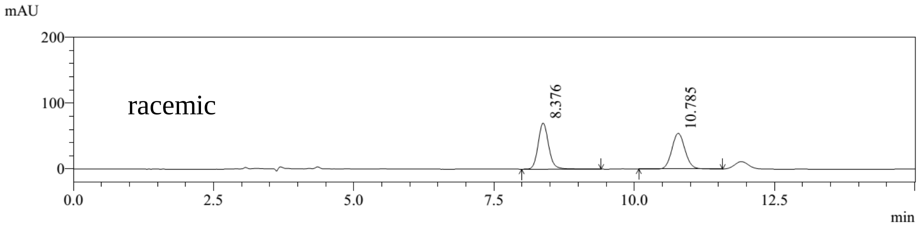

Peak Table

PDA Ch1 240nm

Peak#	Ret. Time	Area	Height	Area%	Height%
1	8.376	911054	63137	50.467	54.398
2	10.785	894191	52928	49.533	45.602
Total		1805245	116064	100.000	100.000

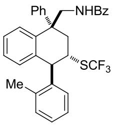

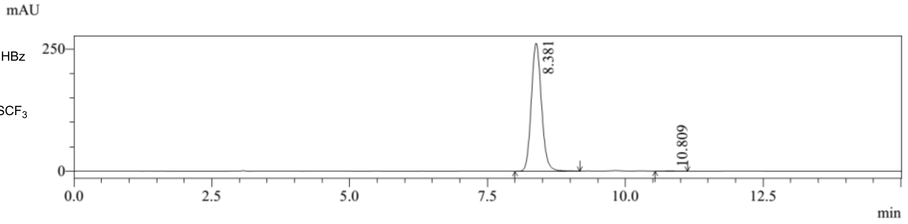

Peak Table

PDA Ch1 240nm

Peak#	Ret. Time	Area	Height	Area%	Height%
1	8.381	3356930	261661	99.746	99.778
2	10.809	8563	582	0.254	0.222
Total		3365494	262244	100.000	100.000

Supplementary Figure 140. HPLC traces for product **2f**

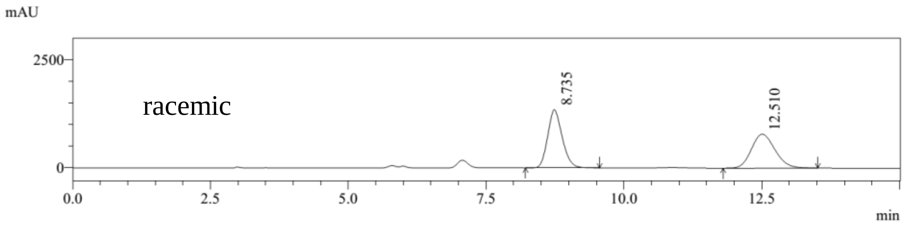

Peak Table

PDA Ch1 206nm

Peak#	Ret. Time	Area	Height	Area%	Height%
1	8.735	24652172	1265492	51.460	61.900
2	12.510	23253130	778936	48.540	38.100
Total		47905302	2044428	100.000	100.000

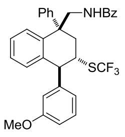

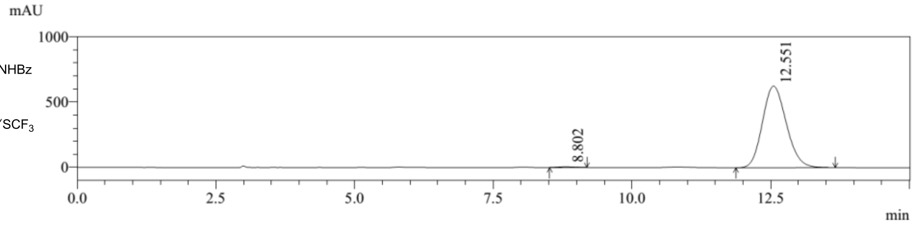

Peak Table

PDA Ch1 206nm

Peak#	Ret. Time	Area	Height	Area%	Height%
1	8.802	121736	5664	0.646	0.920
2	12.551	18708660	609805	99.354	99.080
Total		18830396	615469	100.000	100.000

Supplementary Figure 141. HPLC traces for product **2g**

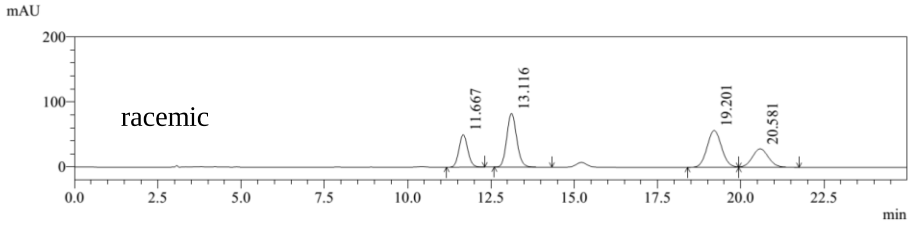

Peak Table

PDA Ch1 245nm

Peak#	Ret. Time	Area	Height	Area%	Height%
1	11.667	922736	49777	17.322	22.902
2	13.116	1715420	82494	32.203	37.956
3	19.201	1721958	56614	32.326	26.049
4	20.581	966753	28457	18.149	13.093
Total		5326868	217342	100.000	100.000

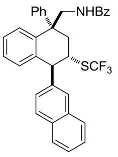

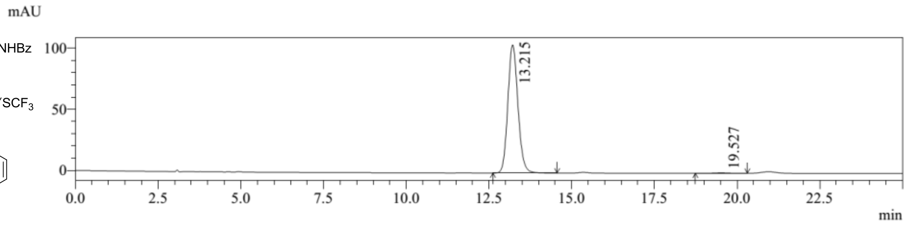

Peak Table

PDA Ch1 245nm

Peak#	Ret. Time	Area	Height	Area%	Height%
1	13.215	2223312	101023	99.500	99.634
2	19.527	11165	371	0.500	0.366
Total		2234477	101394	100.000	100.000

Supplementary Figure 142. HPLC traces for product **2h**

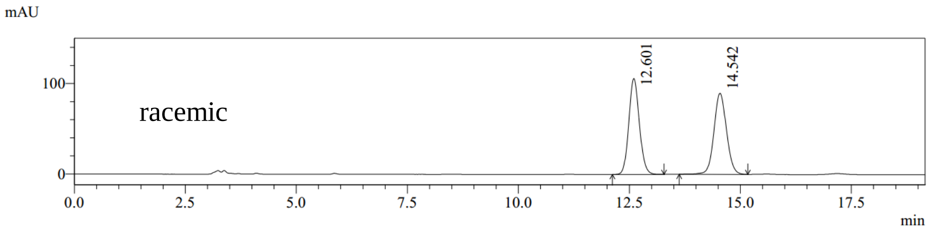

Peak Table

PDA Ch1 222nm

Peak#	Ret. Time	Area	Height	Area%	Height%
1	12.601	1758091	106062	49.838	54.189
2	14.542	1769522	89666	50.162	45.811
Total		3527613	195728	100.000	100.000

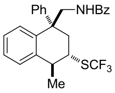

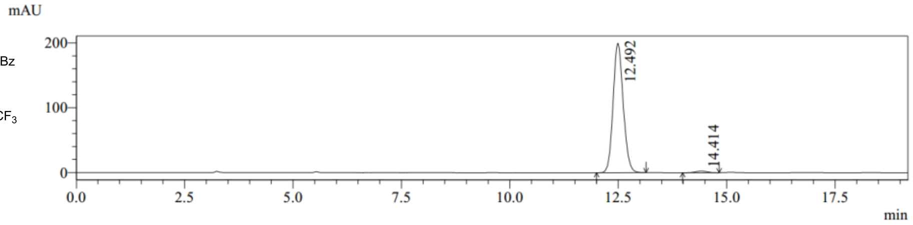

Peak Table

PDA Ch1 222nm

Peak#	Ret. Time	Area	Height	Area%	Height%
1	12.492	3264190	199684	98.696	98.861
2	14.414	43133	2301	1.304	1.139
Total		3307323	201985	100.000	100.000

Supplementary Figure 143. HPLC traces for product **2i**

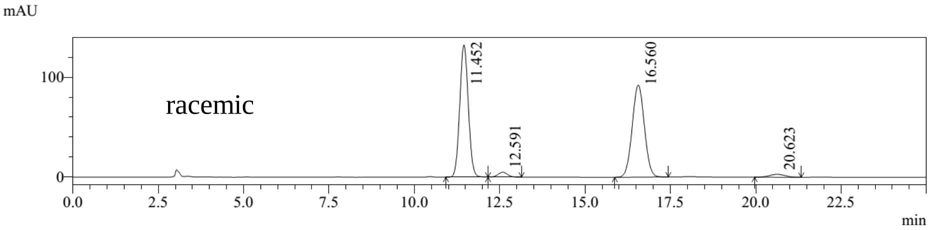

Peak Table

PDA Ch1 222nm

Peak#	Ret. Time	Area	Height	Area%	Height%
1	11.452	2314504	132598	48.177	56.957
2	12.591	99964	4965	2.081	2.133
3	16.560	2296285	92302	47.797	39.648
4	20.623	93448	2939	1.945	1.262
Total		4804202	232803	100.000	100.000

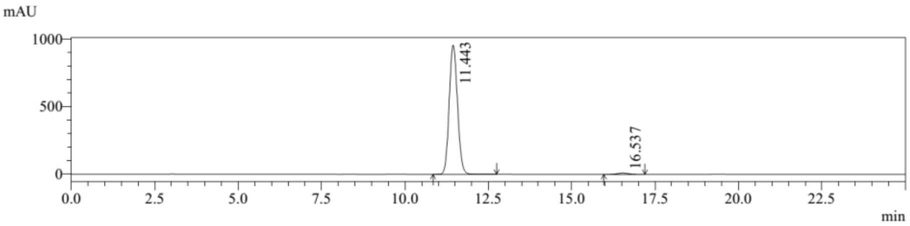

Peak Table

PDA Ch1 222nm

Peak#	Ret. Time	Area	Height	Area%	Height%
1	11.443	16539070	956663	98.620	99.025
2	16.537	231513	9417	1.380	0.975
Total		16770583	966081	100.000	100.000

Supplementary Figure 144. HPLC traces for product **2j**

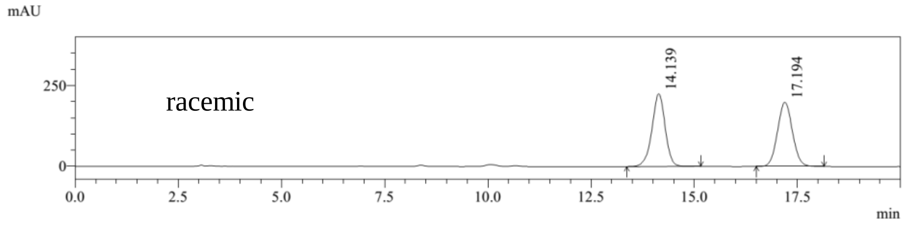

Peak Table

PDA Ch1 222nm

Peak#	Ret. Time	Area	Height	Area%	Height%
1	14.139	5002598	220394	50.052	53.054
2	17.194	4992219	195022	49.948	46.946
Total		9994817	415416	100.000	100.000

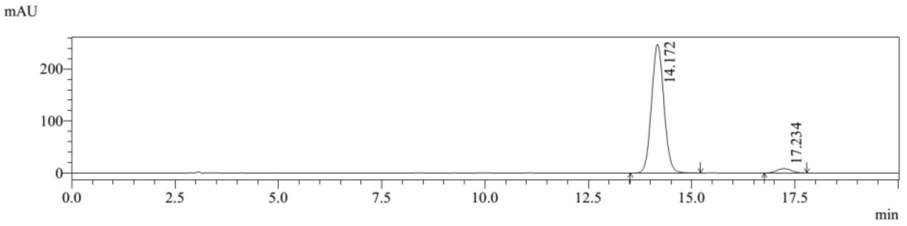

Peak Table

PDA Ch1 222nm

Peak#	Ret. Time	Area	Height	Area%	Height%
1	14.172	5163626	247479	96.146	96.686
2	17.234	206957	8484	3.854	3.314
Total		5370583	255963	100.000	100.000

Supplementary Figure 145. HPLC traces for product **2k**

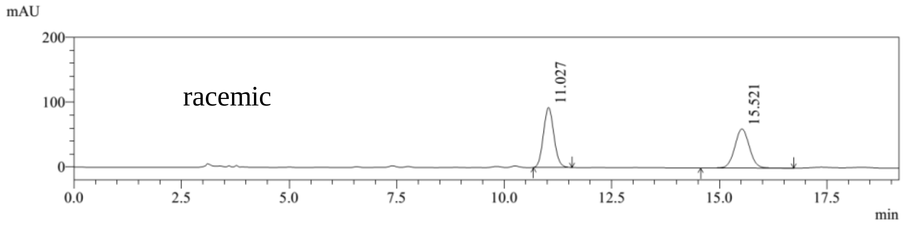

Peak Table

PDA Ch1 222nm

Peak#	Ret. Time	Area	Height	Area%	Height%
1	11.027	1572594	90589	52.437	60.473
2	15.521	1426433	59210	47.563	39.527
Total		2999027	149799	100.000	100.000

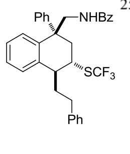

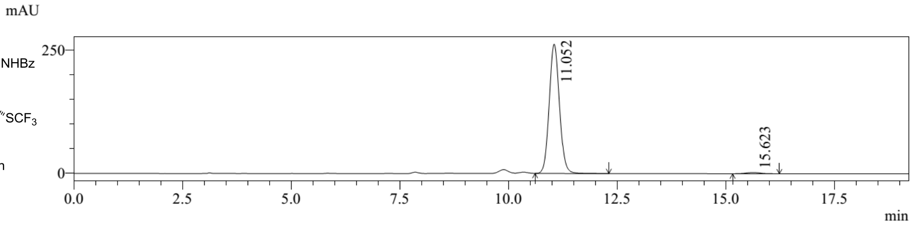

Peak Table

PDA Ch1 222nm

Peak#	Ret. Time	Area	Height	Area%	Height%
1	11.052	4329121	262707	98.578	98.987
2	15.623	62439	2689	1.422	1.013
Total		4391560	265396	100.000	100.000

Supplementary Figure 146. HPLC traces for product **2l**

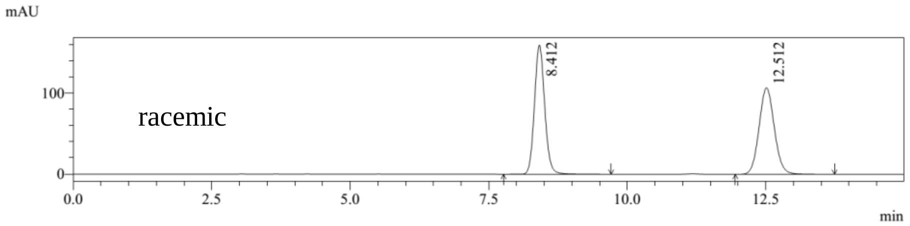

Peak Table

PDA Ch1 290nm

Peak#	Ret. Time	Area	Height	Area%	Height%
1	8.412	2072388	154550	50.195	59.427
2	12.512	2056275	105518	49.805	40.573
Total		4128663	260068	100.000	100.000

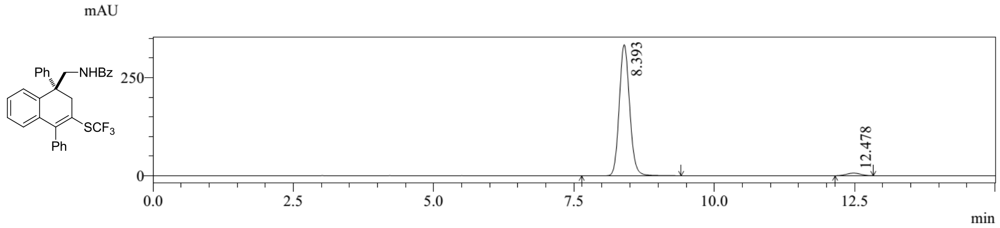

Peak Table

PDA Ch1 290nm

Peak#	Ret. Time	Area	Height	Area%	Height%
1	8.393	4303844	334512	97.260	98.030
2	12.478	121254	6723	2.740	1.970
Total		4425097	341235	100.000	100.000

Supplementary Figure 147. HPLC traces for product **2m**

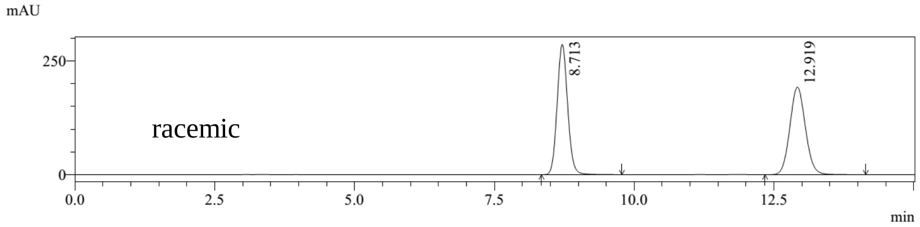

Peak Table

PDA Ch1 280nm

Peak#	Ret. Time	Area	Height	Area%	Height%
1	8.713	3749136	286897	50.064	59.806
2	12.919	3739532	192814	49.936	40.194
Total		7488667	479711	100.000	100.000

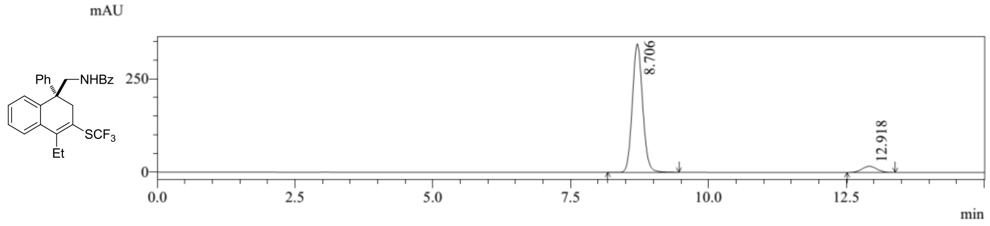

Peak Table

PDA Ch1 280nm

Peak#	Ret. Time	Area	Height	Area%	Height%
1	8.706	4485266	345200	93.474	95.427
2	12.918	313153	16543	6.526	4.573
Total		4798419	361743	100.000	100.000

Supplementary Figure 148. HPLC traces for product **2n**

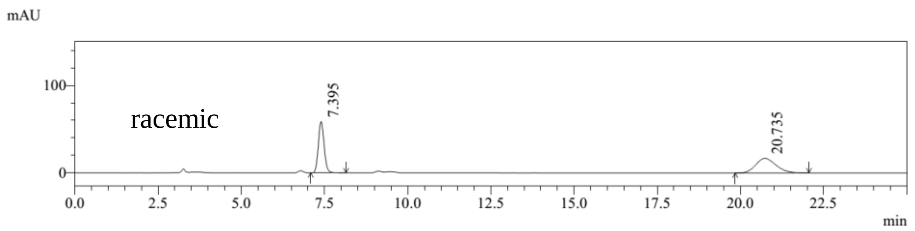

Peak Table

PDA Ch1 262nm

Peak#	Ret. Time	Area	Height	Area%	Height%
1	7.395	716457	58717	50.235	77.956
2	20.735	709750	16604	49.765	22.044
Total		1426208	75321	100.000	100.000

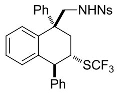

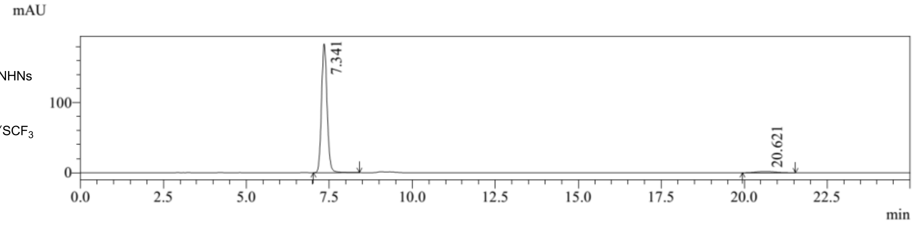

Peak Table

PDA Ch1 262nm

Peak#	Ret. Time	Area	Height	Area%	Height%
1	7.341	2158951	184728	96.985	99.110
2	20.621	67117	1659	3.015	0.890
Total		2226067	186387	100.000	100.000

Supplementary Figure 149. HPLC traces for product **2o**

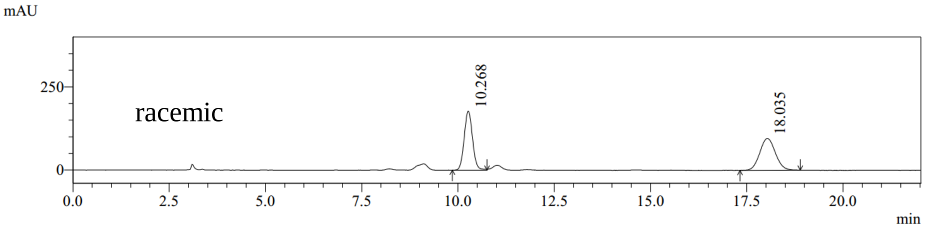

Peak Table

PDA Ch1 207nm

Peak#	Ret. Time	Area	Height	Area%	Height%
1	10.268	2676554	173033	49.921	64.549
2	18.035	2685014	95031	50.079	35.451
Total		5361568	268064	100.000	100.000

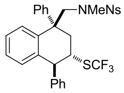

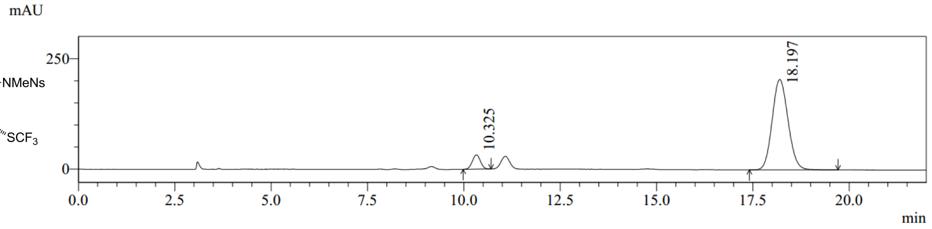

Peak Table

PDA Ch1 207nm

Peak#	Ret. Time	Area	Height	Area%	Height%
1	10.325	474832	31663	7.490	13.442
2	18.197	5864782	203885	92.510	86.558
Total		6339614	235549	100.000	100.000

Supplementary Figure 150. HPLC traces for product **2o’**

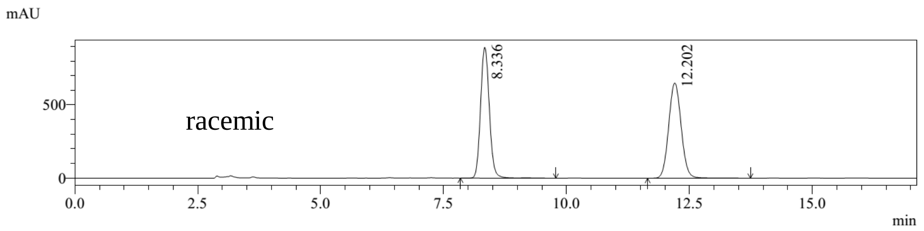

Peak Table

PDA Ch1 207nm

Peak#	Ret. Time	Area	Height	Area%	Height%
1	8.336	11137915	863396	49.170	57.567
2	12.202	11514080	636403	50.830	42.433
Total		22651994	1499798	100.000	100.000

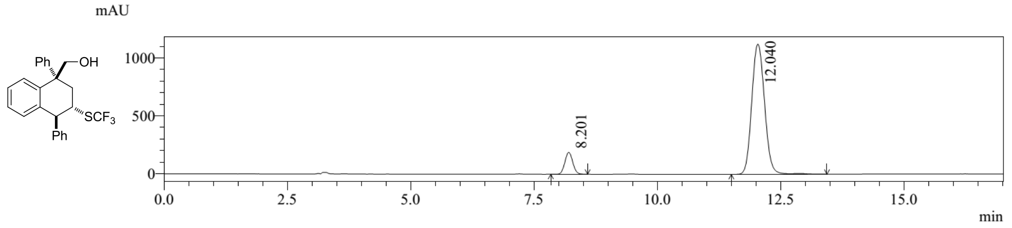

Peak Table

PDA Ch1 207nm

Peak#	Ret. Time	Area	Height	Area%	Height%
1	8.201	2116995	173990	9.419	13.823
2	12.040	20359179	1084709	90.581	86.177
Total		22476175	1258698	100.000	100.000

Supplementary Figure 151. HPLC traces for product **2p**

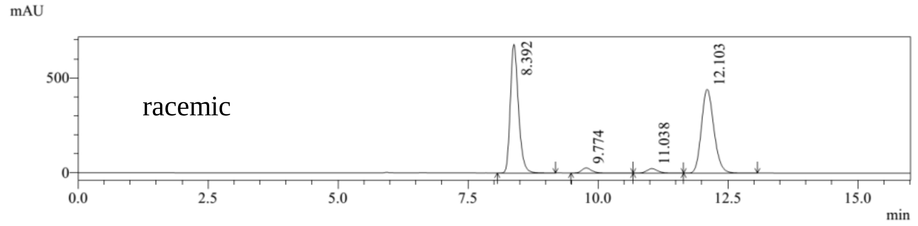

Peak Table

PDA Ch1 222nm

Peak#	Ret. Time	Area	Height	Area%	Height%
1	8.392	7396276	623038	47.723	57.613
2	9.774	350322	24860	2.260	2.299
3	11.038	341397	22093	2.203	2.043
4	12.103	7410238	411424	47.813	38.045
Total		15498233	1081414	100.000	100.000

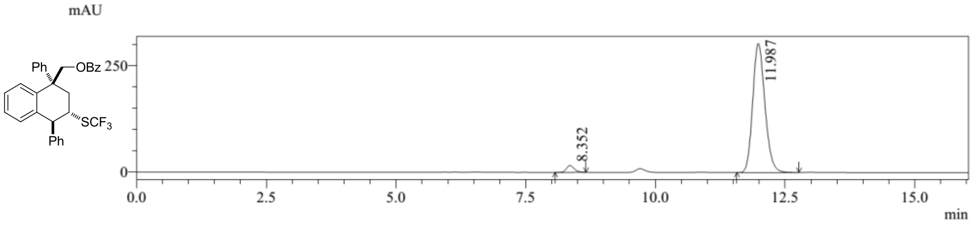

Peak Table

PDA Ch1 222nm

Peak#	Ret. Time	Area	Height	Area%	Height%
1	8.352	164912	14304	3.207	4.592
2	11.987	4977127	297186	96.793	95.408
Total		5142039	311490	100.000	100.000

Supplementary Figure 152. HPLC traces for product **2q**

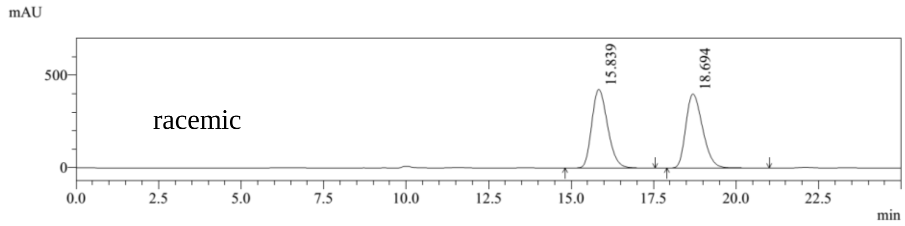

Peak Table

PDA Ch1 222nm

Peak#	Ret. Time	Area	Height	Area%	Height%
1	15.839	14197822	424538	50.020	51.493
2	18.694	14186476	399913	49.980	48.507
Total		28384298	824450	100.000	100.000

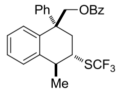

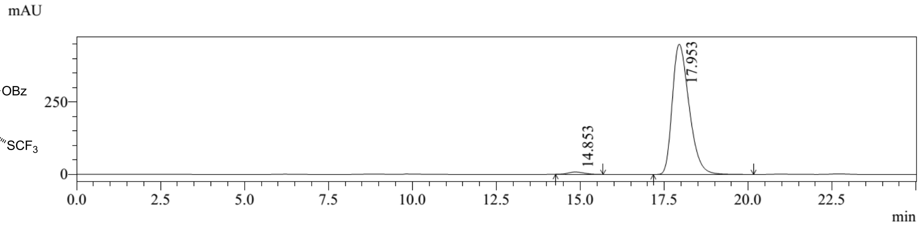

Peak Table

PDA Ch1 222nm

Peak#	Ret. Time	Area	Height	Area%	Height%
1	14.853	274831	8071	1.720	1.773
2	17.953	15706887	447122	98.280	98.227
Total		15981718	455193	100.000	100.000

Supplementary Figure 153. HPLC traces for product **2r**

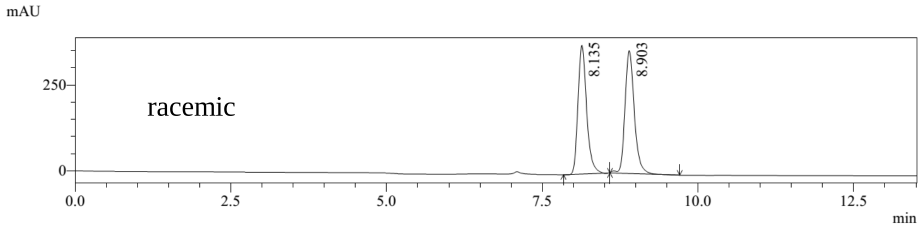

Peak Table

PDA Ch1 222nm

Peak#	Ret. Time	Area	Height	Area%	Height%
1	8.135	3622542	315621	49.236	50.592
2	8.903	3734898	308231	50.764	49.408
Total		7357439	623852	100.000	100.000

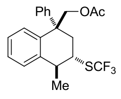

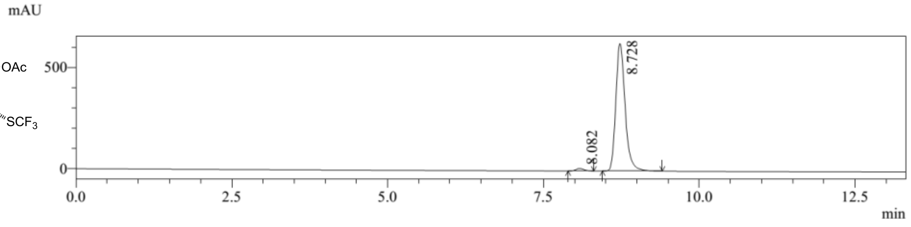

Peak Table

PDA Ch1 222nm

Peak#	Ret. Time	Area	Height	Area%	Height%
1	8.082	113272	12462	1.697	1.944
2	8.728	6563307	628644	98.303	98.056
Total		6676579	641106	100.000	100.000

Supplementary Figure 154. HPLC traces for product **2s**

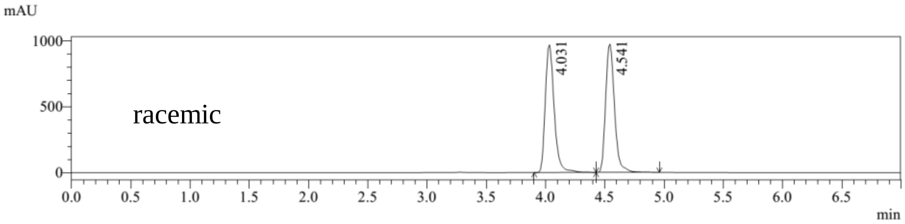

Peak Table

PDA Ch1 222nm

Peak#	Ret. Time	Area	Height	Area%	Height%
1	4.031	5065012	969899	49.908	49.921
2	4.541	5083646	972968	50.092	50.079
Total		10148658	1942868	100.000	100.000

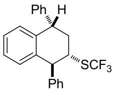

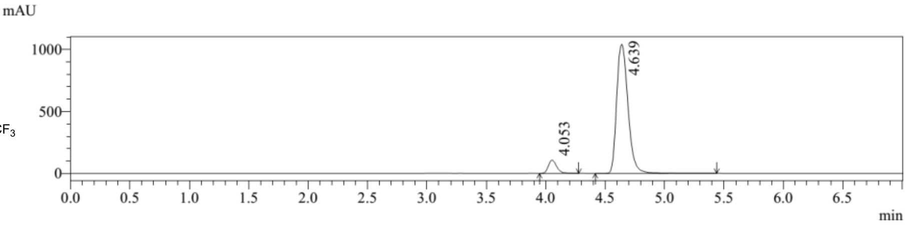

Peak Table

PDA Ch1 222nm

Peak#	Ret. Time	Area	Height	Area%	Height%
1	4.053	533990	108186	7.166	9.386
2	4.639	6917402	1044402	92.834	90.614
Total		7451392	1152588	100.000	100.000

Supplementary Figure 155. HPLC traces for product **2t**

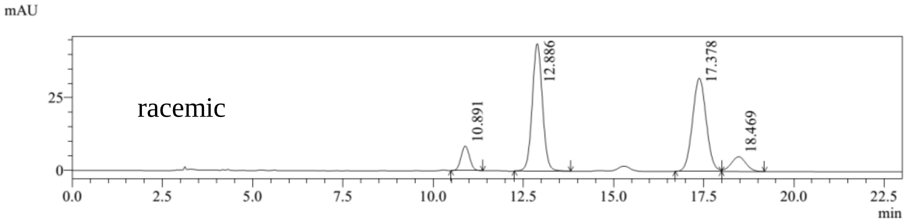

Peak Table

PDA Ch1 238nm

Peak#	Ret. Time	Area	Height	Area%	Height%
1	10.891	140785	8528	7.036	9.509
2	12.886	865406	43977	43.248	49.036
3	17.378	850632	32132	42.510	35.829
4	18.469	144212	5045	7.207	5.626
Total		2001035	89683	100.000	100.000

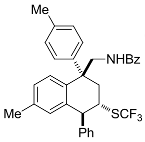

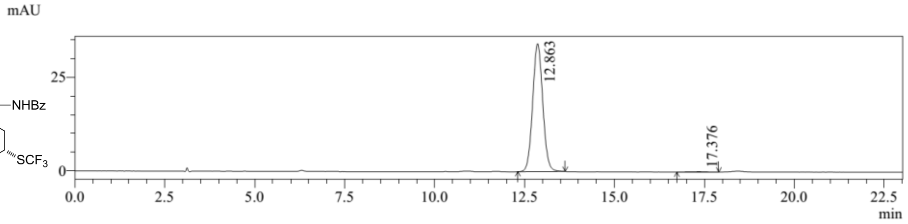

Peak Table

PDA Ch1 238nm

Peak#	Ret. Time	Area	Height	Area%	Height%
1	12.863	667886	34200	99.680	99.756
2	17.376	2141	84	0.320	0.244
Total		670027	34284	100.000	100.000

Supplementary Figure 156. HPLC traces for product **2u**

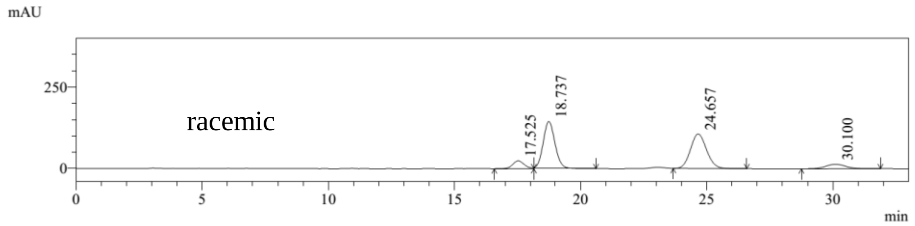

Peak Table

PDA Ch1 230nm

Peak#	Ret. Time	Area	Height	Area%	Height%
1	17.525	787174	24014	7.130	8.383
2	18.737	4776255	143095	43.262	49.955
3	24.657	4704709	105400	42.614	36.795
4	30.100	772061	13941	6.993	4.867
Total		11040200	286450	100.000	100.000

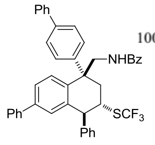

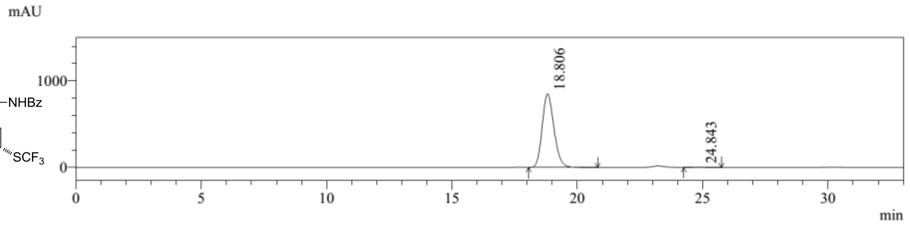

Peak Table

PDA Ch1 230nm

Peak#	Ret. Time	Area	Height	Area%	Height%
1	18.806	27938551	850838	99.616	99.678
2	24.843	107630	2749	0.384	0.322
Total		28046181	853587	100.000	100.000

Supplementary Figure 157. HPLC traces for product **2v**

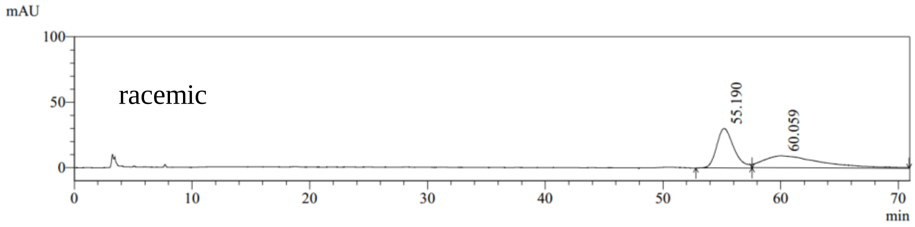

Peak Table

PDA Ch1 220nm

Peak#	Ret. Time	Area	Height	Area%	Height%
1	55.190	3075735	30218	49.354	76.799
2	60.059	3156241	9129	50.646	23.201
Total		6231976	39347	100.000	100.000

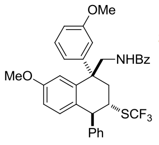

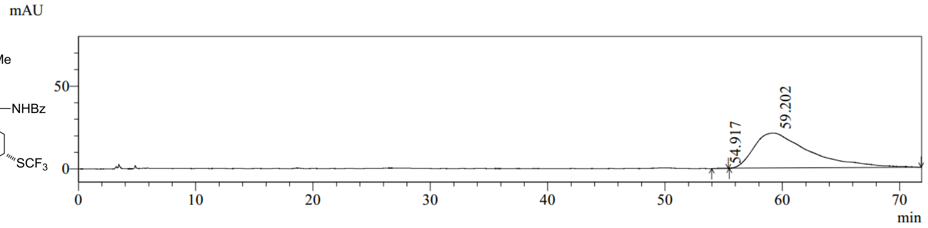

Peak Table

PDA Ch1 220nm

Peak#	Ret. Time	Area	Height	Area%	Height%
1	54.917	15572	263	0.223	1.230
2	59.202	6956121	21075	99.777	98.770
Total		6971693	21338	100.000	100.000

Supplementary Figure 158. HPLC traces for product **2w**

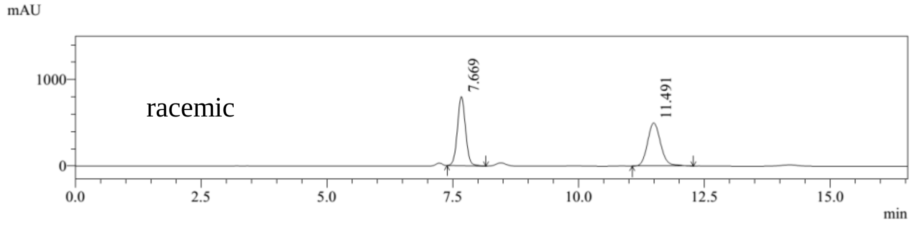

Peak Table

PDA Ch1 222nm

Peak#	Ret. Time	Area	Height	Area%	Height%
1	7.669	8954498	797791	49.602	61.522
2	11.491	9098361	498972	50.398	38.478
Total		18052859	1296763	100.000	100.000

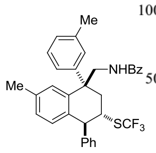

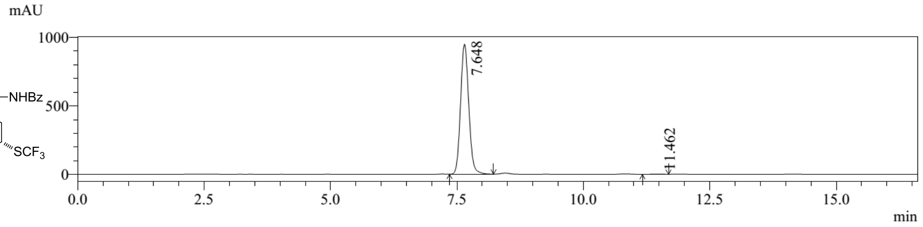

Peak Table

PDA Ch1 222nm

Peak#	Ret. Time	Area	Height	Area%	Height%
1	7.648	10732288	952093	99.812	99.850
2	11.462	20238	1426	0.188	0.150
Total		10752526	953519	100.000	100.000

Supplementary Figure 159. HPLC traces for product **2x**

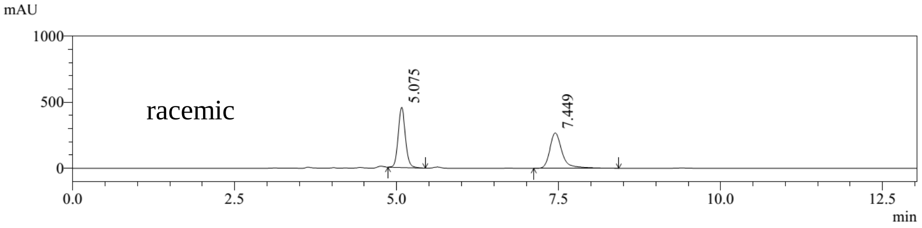

Peak Table

PDA Ch1 222nm

Peak#	Ret. Time	Area	Height	Area%	Height%
1	5.075	3570248	455581	49.976	62.982
2	7.449	3573714	267767	50.024	37.018
Total		7143962	723348	100.000	100.000

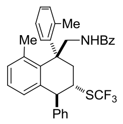

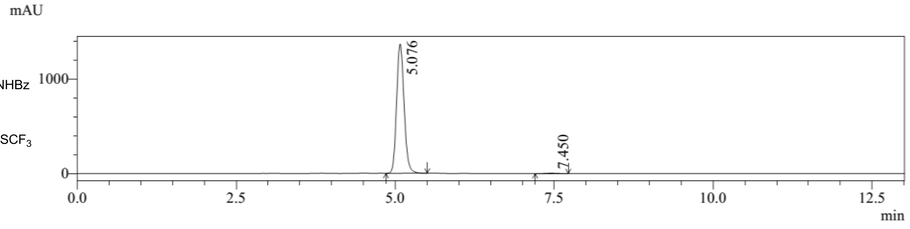

Peak Table

PDA Ch1 222nm

Peak#	Ret. Time	Area	Height	Area%	Height%
1	5.076	11215119	1368933	99.558	99.693
2	7.450	49776	4210	0.442	0.307
Total		11264895	1373142	100.000	100.000

Supplementary Figure 160. HPLC traces for product **2y**

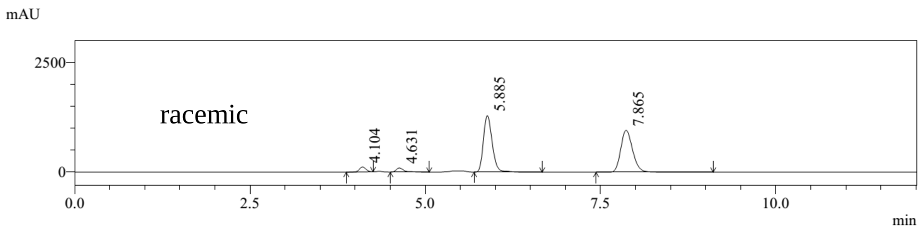

Peak Table

PDA Ch1 268nm

Peak#	Ret. Time	Area	Height	Area%	Height%
1	4.104	783980	113824	3.217	4.621
2	4.631	789648	98180	3.240	3.986
3	5.885	11413140	1291200	46.835	52.415
4	7.865	11382076	960218	46.707	38.979
Total		24368844	2463422	100.000	100.000

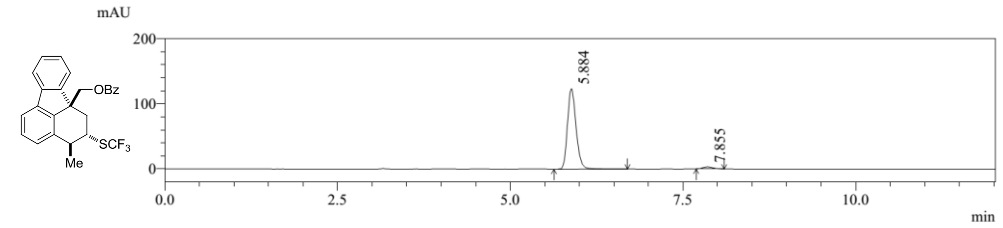

Peak Table

PDA Ch1 268nm

Peak#	Ret. Time	Area	Height	Area%	Height%
1	5.884	1072777	123388	96.844	97.397
2	7.855	34961	3298	3.156	2.603
Total		1107737	126685	100.000	100.000

Supplementary Figure 161. HPLC traces for product **2z**

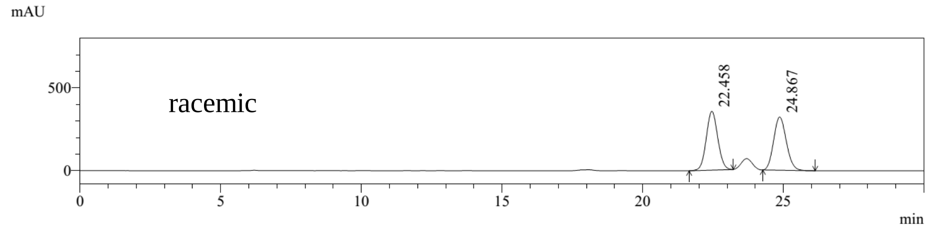

Peak Table

PDA Ch1 222nm

Peak#	Ret. Time	Area	Height	Area%	Height%
1	22.458	10105163	351994	50.070	52.744
2	24.867	10076794	315371	49.930	47.256
Total		20181956	667365	100.000	100.000

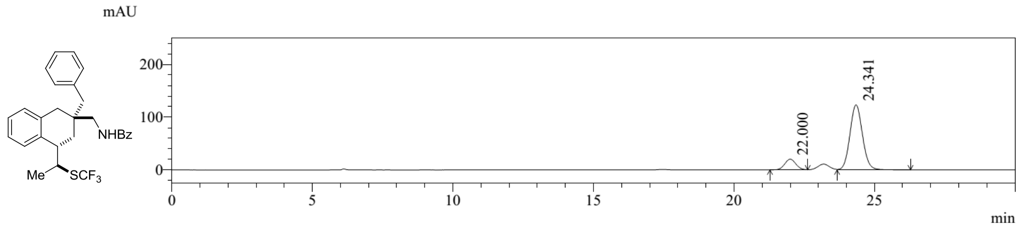

Peak Table

PDA Ch1 222nm

Peak#	Ret. Time	Area	Height	Area%	Height%
1	22.000	566488	20192	12.887	14.296
2	24.341	3829294	121051	87.113	85.704
Total		4395782	141243	100.000	100.000

Supplementary Figure 162. HPLC traces for product **4**

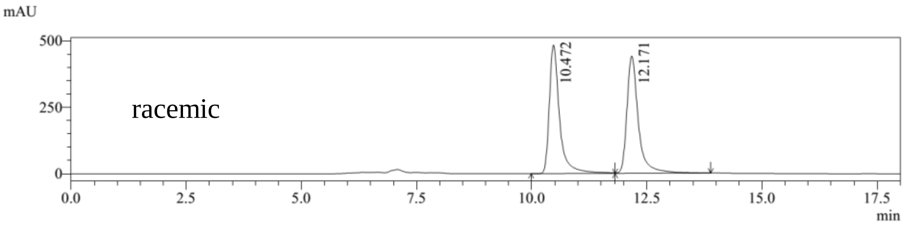

Peak Table

PDA Ch1 222nm

Peak#	Ret. Time	Area	Height	Area%	Height%
1	10.472	7541340	483692	49.751	52.298
2	12.171	7616838	441184	50.249	47.702
Total		15158179	924876	100.000	100.000

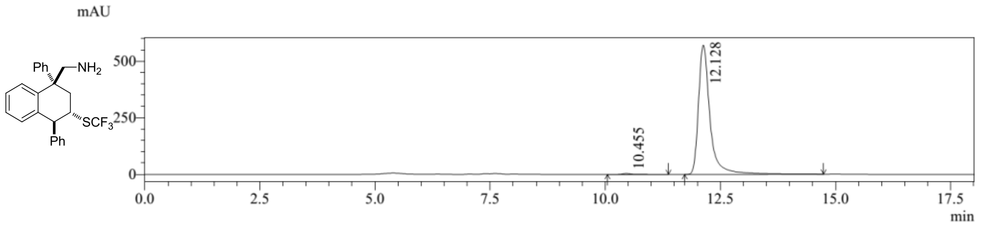

Peak Table

PDA Ch1 222nm

Peak#	Ret. Time	Area	Height	Area%	Height%
1	10.455	69122	3845	0.689	0.668
2	12.128	9958478	572013	99.311	99.332
Total		10027600	575858	100.000	100.000

Supplementary Figure 163. HPLC traces for product **2ab**

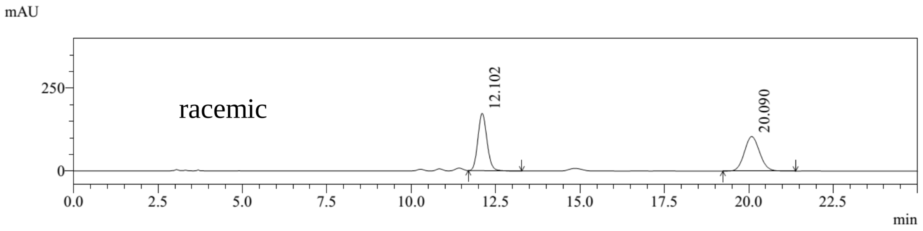

Peak Table

PDA Ch1 222nm

Peak#	Ret. Time	Area	Height	Area%	Height%
1	12.102	3247847	172797	49.666	62.392
2	20.090	3291552	104155	50.334	37.608
Total		6539399	276952	100.000	100.000

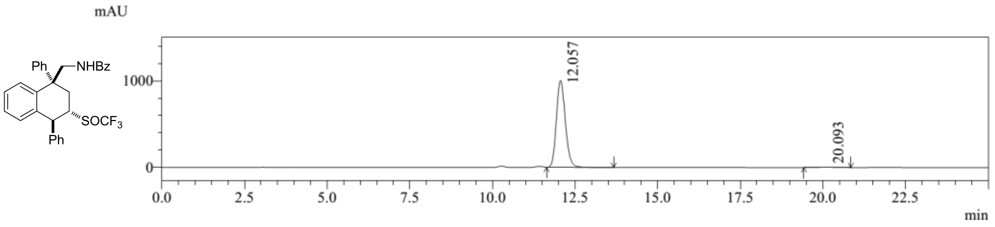

Peak Table

PDA Ch1 222nm

Peak#	Ret. Time	Area	Height	Area%	Height%
1	12.057	18927365	999765	99.342	99.600
2	20.093	125393	4013	0.658	0.400
Total		19052758	1003779	100.000	100.000

Supplementary Figure 164. HPLC traces for product **2ac**

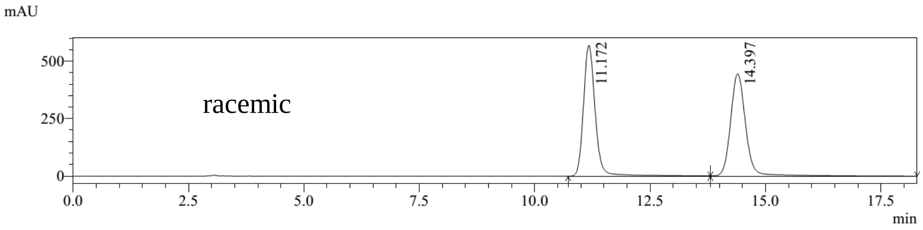

Peak Table

PDA Ch1 222nm

Peak#	Ret. Time	Area	Height	Area%	Height%
1	11.172	10221734	569326	49.905	56.129
2	14.397	10260821	444986	50.095	43.871
Total		20482555	1014312	100.000	100.000

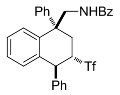

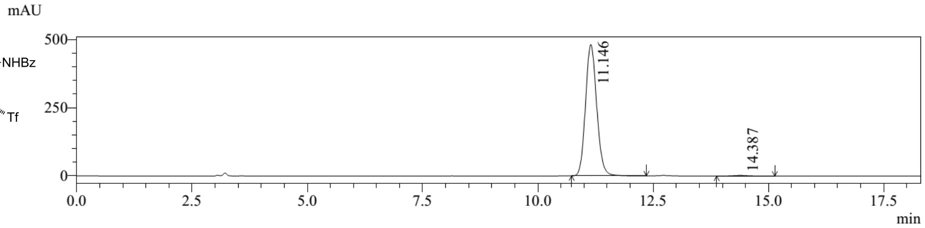

Peak Table

PDA Ch1 222nm

Peak#	Ret. Time	Area	Height	Area%	Height%
1	11.146	8172217	482368	99.389	99.523
2	14.387	50249	2313	0.611	0.477
Total		8222466	484681	100.000	100.000

Supplementary Figure 165. HPLC traces for product **2ad**

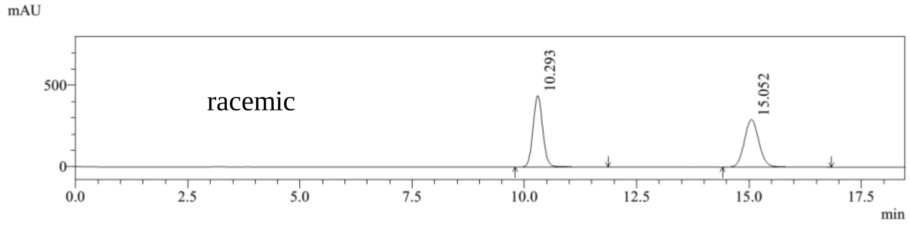

Peak Table

PDA Ch1 240nm

Peak#	Ret. Time	Area	Height	Area%	Height%
1	10.293	6677262	439722	50.272	60.115
2	15.052	6605053	291744	49.728	39.885
Total		13282316	731466	100.000	100.000

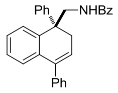

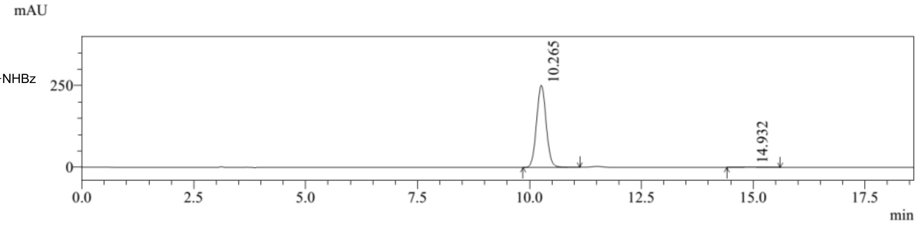

Peak Table

PDA Ch1 240nm

Peak#	Ret. Time	Area	Height	Area%	Height%
1	10.265	3779369	244871	99.522	99.680
2	14.932	18156	785	0.478	0.320
Total		3797525	245656	100.000	100.000

Supplementary Figure 166. HPLC traces for product **2ae**

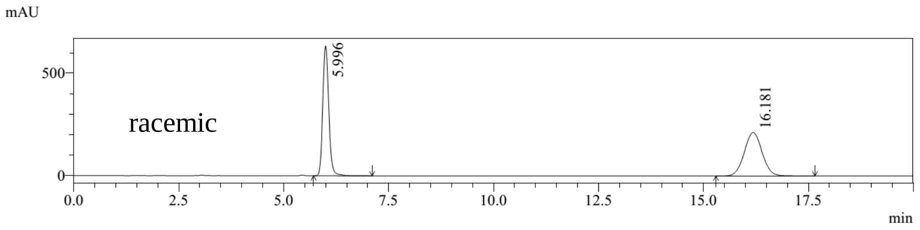

Peak Table

PDA Ch1 222nm

Peak#	Ret. Time	Area	Height	Area%	Height%
1	5.996	6308876	636273	50.084	74.913
2	16.181	6287669	213081	49.916	25.087
Total		12596545	849354	100.000	100.000

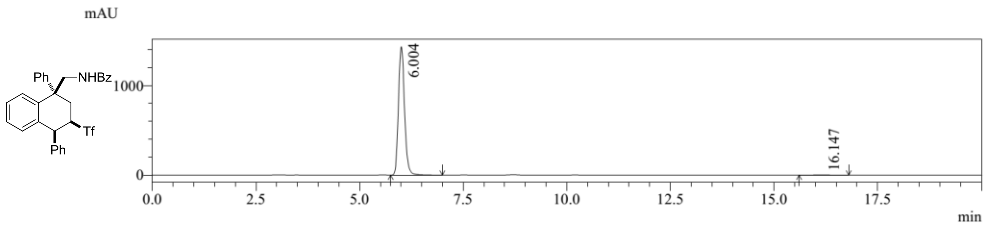

Peak Table

PDA Ch1 222nm

Peak#	Ret. Time	Area	Height	Area%	Height%
1	6.004	14736531	1436658	99.515	99.821
2	16.147	71751	2573	0.485	0.179
Total		14808282	1439230	100.000	100.000

Supplementary Figure 167. HPLC traces for product **2af**

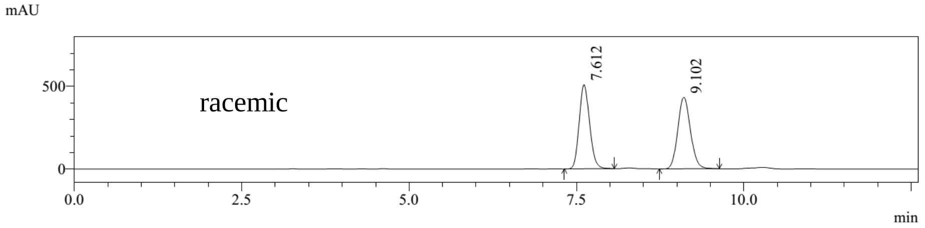

Peak Table

PDA Ch1 244nm

Peak#	Ret. Time	Area	Height	Area%	Height%
1	7.612	5532713	449064	49.243	52.813
2	9.102	5702739	401234	50.757	47.187
Total		11235452	850298	100.000	100.000

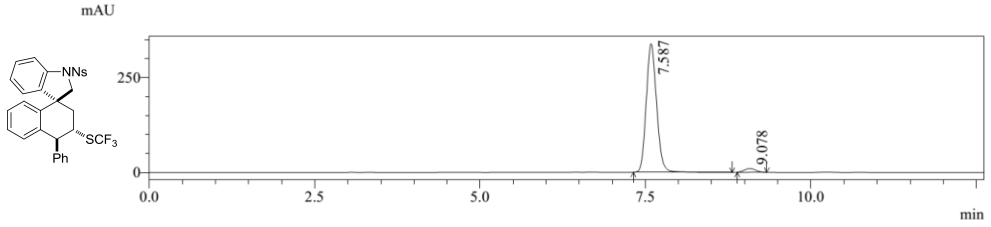

Peak Table

PDA Ch1 244nm

Peak#	Ret. Time	Area	Height	Area%	Height%
1	7.587	3716054	324109	96.900	97.139
2	9.078	118881	9544	3.100	2.861
Total		3834934	333653	100.000	100.000

Supplementary Figure 168. HPLC traces for product **2ag**

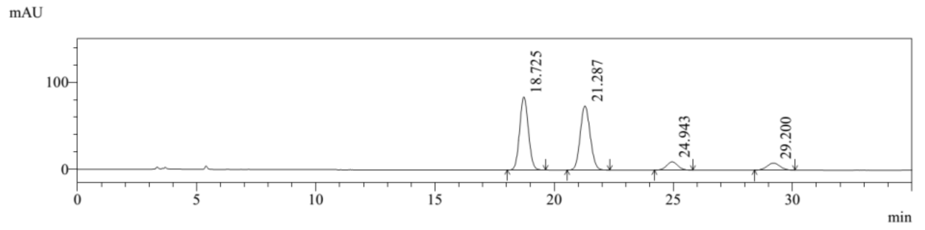

Peak Table

PDA Ch1 254nm

Peak#	Ret. Time	Area	Height	Area%	Height%
1	18.725	2141313	83726	43.832	47.897
2	21.287	2134992	73579	43.702	42.093
3	24.943	305872	9446	6.261	5.404
4	29.200	303116	8052	6.205	4.606
Total		4885292	174803	100.000	100.000

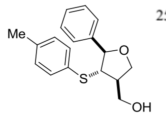

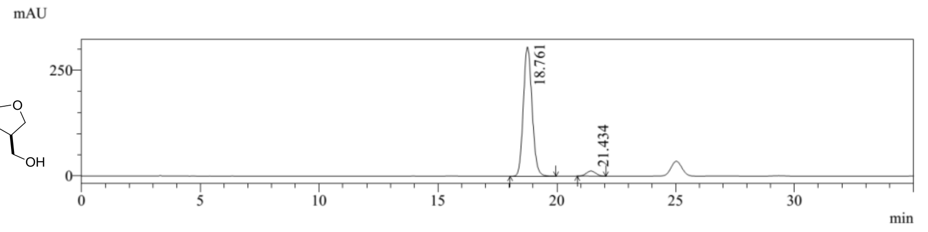

Peak Table

PDA Ch1 254nm

Peak#	Ret. Time	Area	Height	Area%	Height%
1	18.761	7815777	305500	95.767	96.205
2	21.434	345507	12051	4.233	3.795
Total		8161284	317550	100.000	100.000

Supplementary Figure 169. HPLC traces for product **7**

Supplementary Methods

Unless otherwise noted, commercial reagents were purchased from Alfa Aesar, TCI, *J & K* or Adamas and used without further purification. THF, Et_2O and toluene were dried by distillation over sodium prior to use. MeCN, dichloromethane and 1,2-dichloroethane were dried by distillation over CaH_2. Hexanes and isopropanol for HPLC were purchased from Fisher Scientific and were HPLC grade without a note. All reactions were carried out using oven-dried glassware and all catalytic reactions proceeded without special care. Analytical thin layer chromatography was performed on 0.20 mm silica gel HSGF-254 plates (Huanghai, China), and visualized under 254 nm UV light or by staining with potassium permanganate. Column chromatography was performed on 200-300 mesh silica gel (Huanghai, China).

^{1}H, ^{19}F and $^{13}C\{^{1}H\}$ NMR spectra were recorded on an Bruker Ascend 400MHz spectrometer and Bruker Ultrashield 300MHz at ambient temperature. ^{1}H NMR spectra are referred to the TMS signal and ^{13}C NMR spectra are referred to the residual solvent signal. Data for ^{1}H NMR are reported as follows: chemical shifts (δ ppm), multiplicities (s = singlet, d = doublet, t = triplet, q = quartet, m = multiplet, br = broad), coupling constants (Hz), integration. Data for $^{13}C\{^{1}H\}$ NMR and ^{19}F NMR are reported as follows: chemical shift (δ ppm), multiplicity (q = quartet), coupling constant (Hz).

High resolution mass spectra of novel compounds were recorded on LTQ Orbitrap Elite LC/MS (ESI), or MAT 95XP (Thermo, EI) at analytical center of Sun Yat-Sen University, and Thermo Fisher Scientific LTQ FT Ultra at Shanghai Institute of Organnic Chemistry, Chinese Academy of Sciences. Infrared (IR) spectra were recorded on Bruker FT-IR spectrometer (EQUINOX 55) at analytical center of Sun Yat-Sen University and reported in wave numbers (cm^{-1}). Melting points were determined on a YUHUA X-5 micro melting point apparatus. Enantiomeric excesses were determined by HPLC analysis on Shimadzu HPLC units including the following instruments: LC-20AT pump, SPD-M20A detector and Dacicel Chiralpak IA, IB, IC,

OJ-H, OD-H, AD-H columns. Optical rotations were recorded on an Anton Paar MCD-200 polarimeter.

All the allyl chlorides were directly purchased or synthesized through the method in reported literature[1] unless otherwise noted. All the racemic products were obtained by using PhSePh as the catalyst at -60 ^{o}C or -45 ^{o}C without a note. The diastereoselectivities of products were determined by crude ^{19}F NMR.

All the calculations were carried out with the Gaussian 09 package.[2] The geometries were optimized using B3LYP[3] and the 6-31G(d) basis set. Frequnecy analysis was conducted at the same level of theory to verify the stationary points to be real minima or saddle points and to obtain the thermodynamic energy corrections. Single-point energies were calculated using M06-2X[4]-D3[5]/6-311+G(d,p)[6] level using IEFPCM[7] salvation model (solvent = dichloromethane) on the B3LYP-optimized structure. The $ee_{predicted}$ and $dr_{predicted}$ were calculated based on Boltzmann distribution.

Determination of the *dr* ratios of products

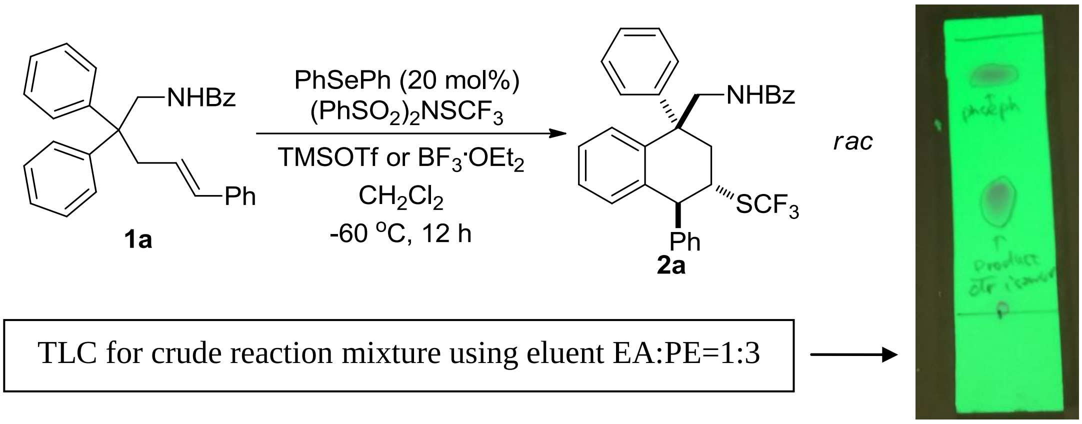

Supplementary Figure 170. PhSePh-catalyzed reaction gave racemic **2a** with 8:1 *dr*.

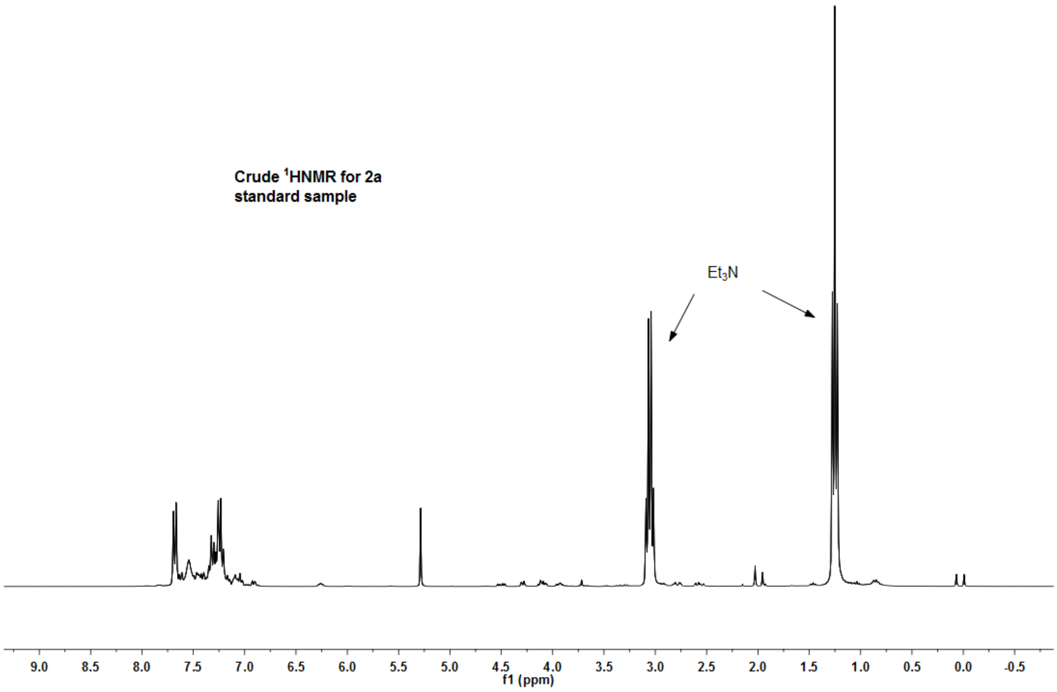

Supplementary Figure 171. Crude ^{1}H NMR for the reaction (Et_3N quench)

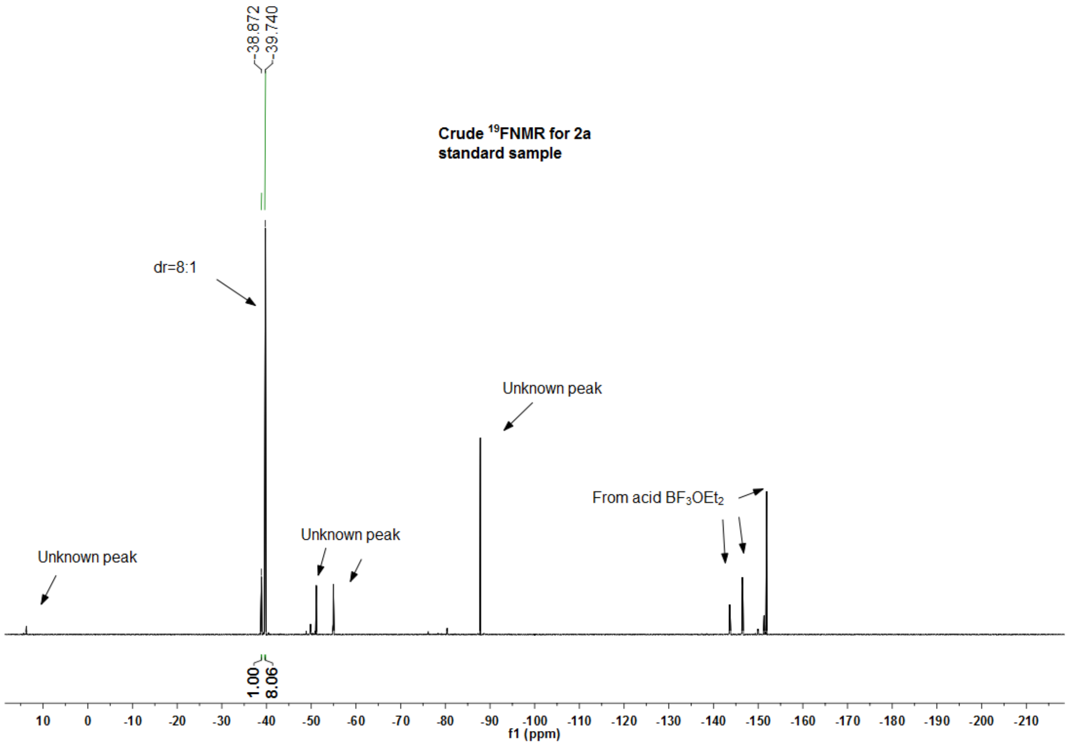

Supplementary Figure 172. Crude ^{19}F NMR for the reaction (Et_3N quench)

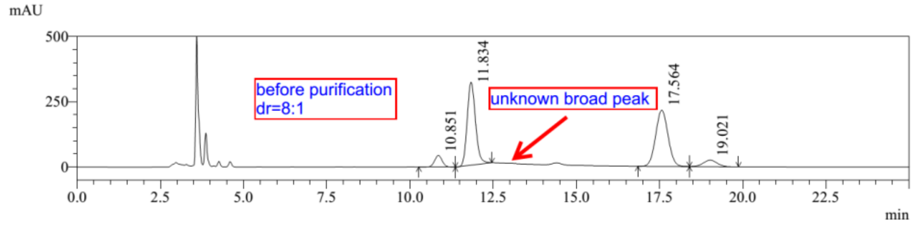

Peak Table

PDA Ch1 222nm

Peak#	Ret. Time	Area	Height	Area%	Height%
1	10.851	707537	44104	5.668	7.337
2	11.834	5505780	317249	44.109	52.780
3	17.564	5565356	215320	44.586	35.822
4	19.021	703617	24405	5.637	4.060
Total		12482289	601078	100.000	100.000

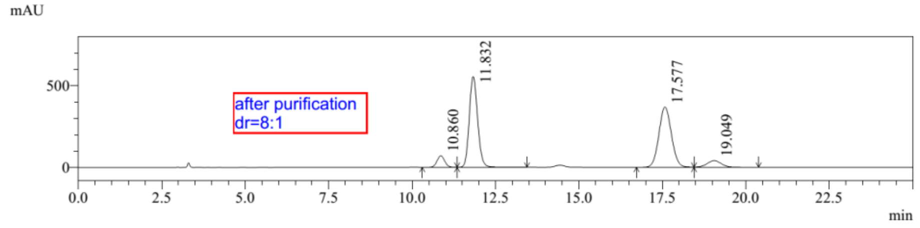

Peak Table

PDA Ch1 222nm

Peak#	Ret. Time	Area	Height	Area%	Height%
1	10.860	1152666	69084	5.323	6.807
2	11.832	9687387	542955	44.735	53.498
3	17.577	9625359	363958	44.448	35.861
4	19.049	1189751	38916	5.494	3.834
Total		21655164	1014913	100.000	100.000

Supplementary Figure 173. HPLC traces for the crude reaction mixture and product after purification by preparative TLC

Note: (1) Crude ^{19}F NMR shows 8:1 *dr* for the product. (2) Purification did not change the *dr* of the product.

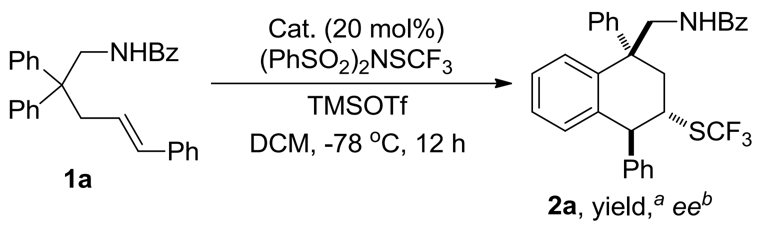

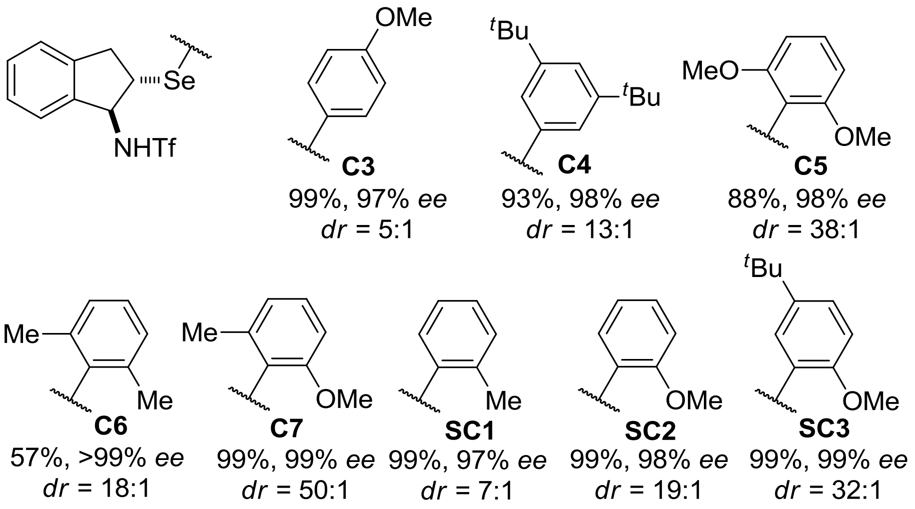

Supplementary Figure 174. Catalyst optimization. Conditions: **1a** (0.05 mmol), $(PhSO_2)_2NSCF_3$ (1.5 equiv), TMSOTf (1.0 equiv), DCM (2.0 mL), -78 °C, 12 h. [a]NMR yield using trifluoromethylbenzene as the internal standard. [b]Determined by HPLC analysis.

Supplementary Table 1. Condition optimization for desymmetrization[a]

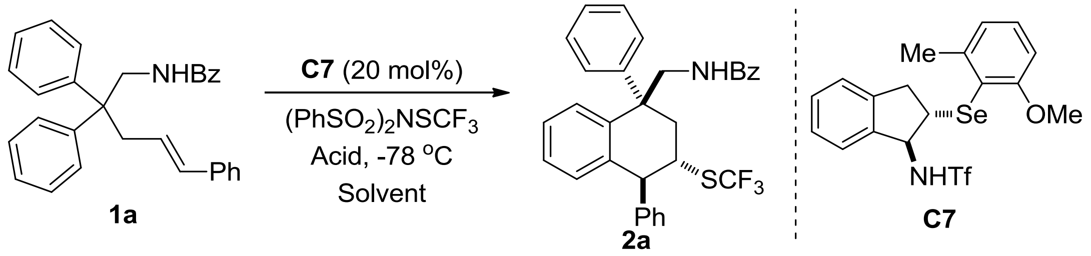

Entry	Solvent	Acid	Yield [%][b], ee [%][c]
1[d]	Toluene (2 mL)	TMSOTf (1.0 equiv)	23, 97
2[e]	DCM (1mL)+DCE (1 mL)	TMSOTf (1.0 equiv)	>99, >99
3[f]	DCM (1 mL)+DCE (1 mL)	$BF_3{\cdot}OEt_2$ (1.0 equiv)	>99, 92
4	DCM (1 mL)+DCE (1 mL)	TfOH (1.0 equiv)	>99, 98
5	DCM (1 mL)+DCE (1 mL)	Tf_2NH (1.0 equiv)	80, 99
6	DCM (1 mL)+DCE (1 mL)	TMSOTf (0.5 equiv)	66, 98
7	DCM (1 mL)+DCE (1 mL)	TMSOTf (2.0 equiv)	>99, >99
8[g]	DCM (1 mL)+DCE (1 mL)	TMSOTf (1.0 equiv)	>99, 98

9[h]	DCM (1 mL)+DCE (1 mL)	TMSOTf (1.0 equiv)	58, 99
10[i]	DCM (1 mL)+DCE (1 mL)	TMSOTf (1.0 equiv)	72, >99
11[j]	DCM (1 mL)+DCE (1 mL)	TMSOTf (1.0 equiv)	>99, >99

[a]Conditions: **1a** (0.05 mmol), $(PhSO_2)_2NSCF_3$ (1.5 equiv), catalyst (20 mol%), TMSOTf (1.0 equiv), DCM (1.0 mL) + DCE (1.0 mL), 12 h, -78 °C. All the products were obtained with 50:1 diastereoselectivities if not noted. [b]Refers to NMR yield using trifluoromethylbenzene as the internal standard. [c]Determined by HPLC analysis on a chiral stationary phase. [d]*dr* = 50:3. [e]Isolated yield in 0.1 mmol scale. [f]*dr* = 100:3. [g]Saccharin-SCF_3 was used. [h]1 mol% catalyst was used. [i]Quenched at 1 h. [j]Quenched at 3 h. Note: *dr* was determined by crude ^{19}F NMR.

Supplementary Table 2. The effect of reaction temperature for desymmetrization

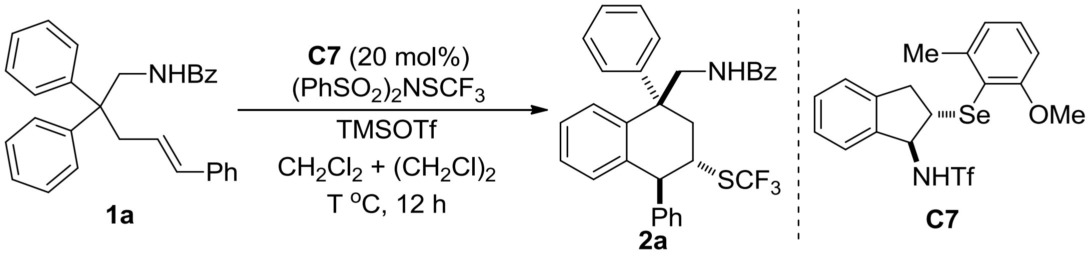

Entry	Temperature	ee [%]	*dr*
1	25 °C	91	5:1
2	0 °C	94	8:1
3	-20 °C	96	11:1
4	-40 °C	97	17:1
5	-60 °C	98	30:1
6	-78 °C	99	50:1

Conditions: **1a** (0.05 mmol), $(PhSO_2)_2NSCF_3$ (1.5 equiv), catalyst (20 mol%), TMSOTf (1.0 equiv), DCM (1.0 mL) + DCE (1.0 mL), 12 h. Note: *dr* was determined by crude ^{19}F NMR. All the reactions were in full coversion.

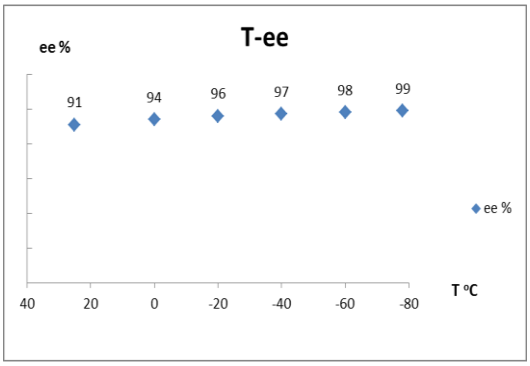

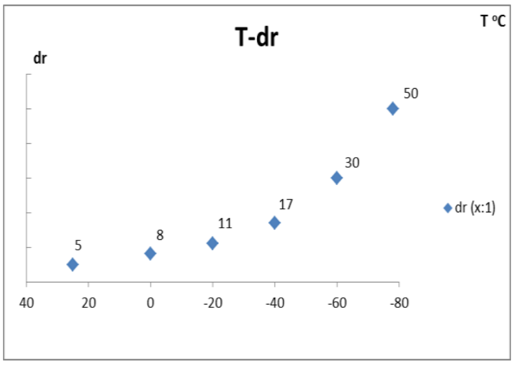

Supplementary Table 3. Additional examination of different amine groups on substrate

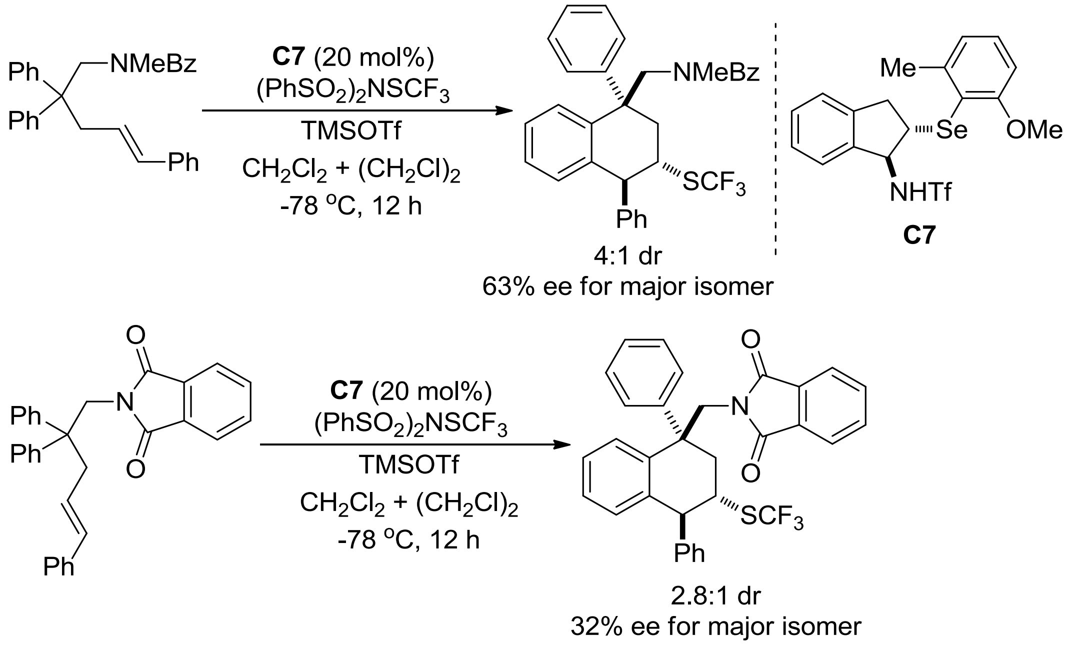

Conditions: Substrate (0.05 mmol), $(PhSO_2)_2NSCF_3$ (1.5 equiv), catalyst (20 mol%), TMSOTf (1.0 equiv), DCM (1.0 mL) + DCE (1.0 mL), 12 h.

Supplementary Note 1

General experimental procedures and analytical data

Catalyst **C1-C7**, **SC1-SC3** were prepared according to the literature method.[8]

Preparation of substrates

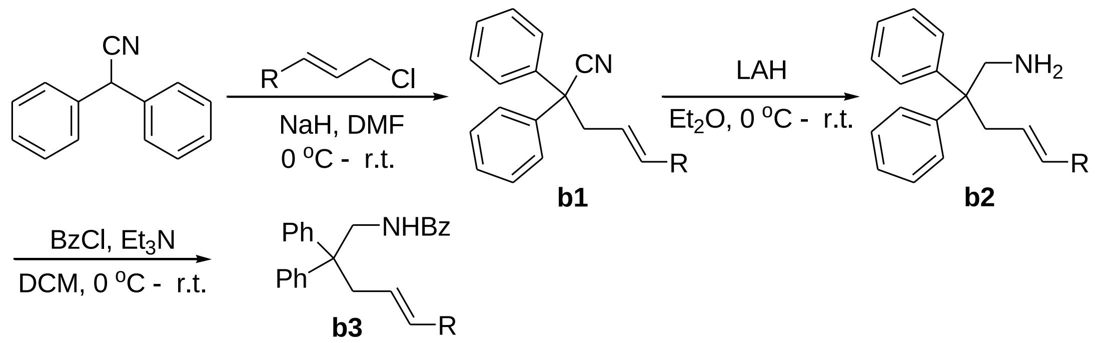

A solution of diphenylacetonitrile (0.87 g, 4.5 mmol) in DMF (5 mL) was added slowly to a suspension of NaH (0.12 g, 5.0 mmol) in DMF (2 mL) at 0 ^{o}C. The resulting mixture was stirred at room temperature for 0.5 h after which it was cooled to 0 ^{o}C, treated with allyl bromide or allyl chloride (5.0 mmol), warmed to room temperature and stirred for 12 h. Then the mixture was poured into ice/water (15 mL) and was extracted with Et_2O (15 mL x 3). The combined organic layers were washed with water (10 mL x 3), dried with Na_2SO_4, and concentrated to give crude product **b1**, which was purified by flash column chromatography (PE/EtOAc = 30/1; v/v) and used in the subsequent step.

To a suspension of $LiAlH_4$ (0.3 g, 8.0 mmol) in Et_2O (10 mL) was added a solution of **b1** (2.0 mmol) in 10 mL Et_2O at 0 ^{o}C. The mixture was warmed to room temperature and stirred for another 2.5 h. After TLC revealed the absence of the starting material, the reaction was quenched with cold water carefully at 0 ^{o}C and then 10% NaOH (10 mL) was added. The aqueous phase was extracted with Et_2O (20 mL x 3) and the combined organic layers were washed with brine and dried over Na_2SO_4. Filtration and evaporation of the solvent left a crude mixture, which was used directly without further purification.

To a solution of **b2** (1.0 mmol) in DCM (5 mL) were subsequently added Et_3N (210

μL, 1.5 mmol) and BzCl (170 μL, 1.5 mmol) at 0 ^{o}C, and then the mixture was stirred at room temperature overnight. The resulting mixture was treated with water (5 mL), and extracted with DCM (5 mL x 3). The combined organic layers were dried over Na_2SO_4 and concentrated. The crude product **b3** was purified by flash column chromatography (PE/EtOAc = 10/1; v/v).

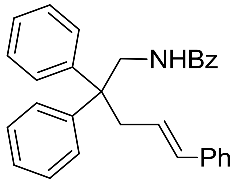

(*E*)-*N*-(2,2,5-Triphenylpent-4-en-1-yl)benzamide (1a): Purified by flash column chromatography using eluent (PE/EtOAc = 30/1; v/v) for the first step and eluent (PE/EtOAc = 10/1; v/v) for the last step. 41% overall yield. White solid. mp: 107.6–109.4 ℃. **IR** (KBr): 3317, 3025, 2923, 1644, 1545, 1483, 1443, 1299, 965, 696 cm^{-1}. **^{1}H NMR** (400 MHz, CDCl3) δ 7.54 – 7.49 (m, 2H), 7.47 – 7.12 (m, 18H), 6.31 (d, *J* = 15.8 Hz, 1H), 5.94 – 5.81 (m, 1H), 5.76 (s, 1H), 4.18 (t, *J* = 9.1 Hz, 2H), 3.07 (d, *J* = 7.2 Hz, 2H). **^{13}C NMR** (101 MHz, CDCl3) δ 167.3, 145.3, 137.5, 134.7, 133.9, 131.5, 128.7, 128.6, 128.5, 128.2, 127.2, 126.9, 126.8, 126.3, 125.6, 51.3, 46.8, 41.7. **HR-ESI-MS** *m/z* calcd. for $C_{30}H_{28}ON$ [M+H]$^{+}$: 418.2165, found: 418.2161.

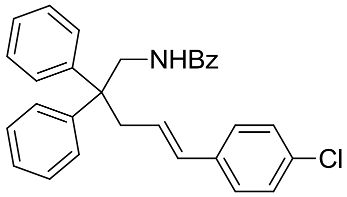

(*E*)-*N*-(5-(4-Chlorophenyl)-2,2-diphenylpent-4-en-1-yl)benzamide (1b): Purified by flash column chromatography using eluent (PE/EtOAc = 30/1; v/v) for the first step and eluent (PE/EtOAc = 10/1; v/v) for the last step. 60% overall yield. White solid. mp: 79.7–82.5 ℃. **IR** (KBr): 3324, 3026, 2929, 1662, 1521, 1486, 1292, 1089, 702, 532 cm^{-1}. **^{1}H NMR** (400 MHz, $CDCl_3$) δ 7.51 (d, *J* = 7.6 Hz, 2H), 7.47 – 7.41 (m, 1H), 7.40 – 7.23 (m, 12H), 7.17 (d, *J* = 7.7 Hz, 2H), 7.08 (d, *J* = 7.8 Hz, 2H), 6.23 (d, *J* = 15.8 Hz, 1H), 5.92 – 5.78 (m, 1H), 5.70 (s, 1H), 4.18 (t, *J* = 7.1 Hz, 2H), 3.04 (d, *J* = 7.2 Hz, 2H). **^{13}C NMR** (101 MHz, $CDCl_3$) δ 167.3, 145.3, 136.1, 134.7, 132.8, 131.6, 128.7, 128.6, 128.2, 127.5, 127.0, 126.8, 126.4, 51.4, 46.6, 41.4. **HR-ESI-MS** *m/z* calcd. for $C_{30}H_{26}ClON$ [M-H]$^{-}$: 450.1630, found: 450.1625.

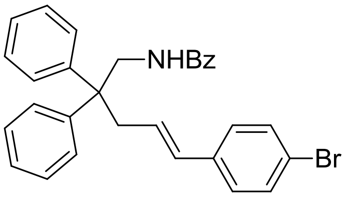

(*E*)-*N*-(5-(4-Bromophenyl)-2,2-diphenylpent-4-en-1-yl)benzamide (1c): Purified by flash column chromatography using eluent (PE/EtOAc = 30/1; v/v)

for the first step and eluent (PE/EtOAc = 10/1; v/v) for the last step. 50% overall yield. White solid. mp: 81.1–83.1 ℃. **IR** (KBr): 3334, 3057, 2925, 1664, 1520, 1485, 1443, 1284, 1007, 700, 521 cm^{-1}. **^{1}H NMR** (400 MHz, $CDCl_3$) δ 7.51 (d, *J* = 7.4 Hz, 2H), 7.43 (t, *J* = 7.3 Hz, 1H), 7.34 (dd, *J* = 16.4, 8.4 Hz, 8H), 7.30 – 7.23 (m, 6H), 7.04 (t, *J* = 12.6 Hz, 2H), 6.21 (d, *J* = 15.8 Hz, 1H), 5.94 – 5.78 (m, 1H), 5.71 (s, 1H), 4.18 (d, *J* = 5.7 Hz, 2H), 3.03 (d, *J* = 7.2 Hz, 2H). **^{13}C NMR** (101 MHz, $CDCl_3$) δ 167.3, 145.3, 136.5, 134.6, 132.8, 131.5, 128.7, 128.6, 128.2, 127.8, 126.9, 126.8, 126.5, 120.9, 51.4, 46.6, 41.4. **HR-ESI-MS** *m/z* calcd. for $C_{30}H_{25}ONBr$ $[M-H]^-$: 494.1125, found: 494.1128.

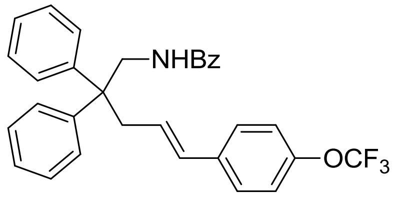

(*E*)-*N*-(2,2-Diphenyl-5-(4-(trifluoromethoxy)phenyl)pent-4-en-1-yl)benzamide (1d): Purified by flash column chromatography using eluent (PE/EtOAc = 30/1; v/v) for the first step and eluent (PE/EtOAc = 10/1; v/v) for the last step. 27% overall yield. White solid. mp: 97.3–99.7 ℃. **IR** (KBr): 3434, 3052, 2934, 1668, 1527, 1491, 1259, 1225, 1197, 1162, 708, 531 cm^{-1}. **^{1}H NMR** (400 MHz, $CDCl_3$) δ 7.52 (d, *J* = 7.5 Hz, 2H), 7.44 (t, *J* = 7.2 Hz, 1H), 7.35 (q, *J* = 7.8 Hz, 6H), 7.31 – 7.25 (m, 6H), 7.17 (d, *J* = 8.6 Hz, 2H), 7.06 (d, *J* = 8.3 Hz, 2H), 6.27 (d, *J* = 15.8 Hz, 1H), 5.92 – 5.78 (m, 1H), 5.71 (s, 1H), 4.19 (d, *J* = 5.8 Hz, 2H), 3.05 (d, *J* = 7.2 Hz, 2H). **^{13}C NMR** (101 MHz, $CDCl_3$) δ 167.3, 148.3, 145.3, 136.4, 134.7, 132.5, 131.6, 128.7, 128.7, 128.2, 127.5, 127.0, 126.8, 124.4 (q, *J* = 256.9 Hz), 121.9, 121.0, 119.3, 116.8, 51.4, 46.6, 41.4. **19F NMR** (377 MHz, $CDCl_3$) δ -57.89. **HR-ESI-MS** *m/z* calcd. for $C_{31}H_{25}O_2NF_3$ $[M-H]^-$: 500.1843, found: 500.1840.

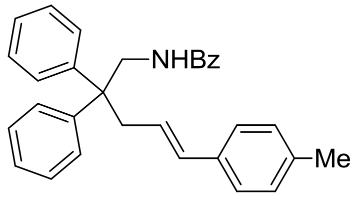

(*E*)-*N*-(2,2-Diphenyl-5-(p-tolyl)pent-4-en-1-yl)benzamide (1e): Purified by flash column chromatography using eluent (PE/EtOAc = 50/1; v/v) for the first step and eluent (PE/EtOAc = 10/1; v/v) for the last step. 59% overall yield. Colourless oil. **IR** (KBr): 3332, 3025, 2920, 1664, 1514, 1486, 1283, 700, 511 cm^{-1}. **^{1}H NMR** (400 MHz, $CDCl_3$) δ 7.51 (d, *J* = 7.2 Hz, 2H), 7.41 (t, *J* = 7.4 Hz, 1H), 7.38 – 7.22 (m, 12H), 7.04 (dd, *J* = 22.1, 8.0 Hz, 4H), 6.27 (d, *J* = 15.8 Hz, 1H), 5.89 – 5.71 (m, 2H), 4.18 (d, *J* = 5.7 Hz, 2H), 3.05 (d, *J* = 7.2 Hz, 2H), 2.27 (s, 3H). **^{13}C NMR** (101 MHz, $CDCl_3$) δ 167.2, 145.3, 136.9, 134.7, 134.7, 133.7,

131.4, 129.1, 128.6, 128.5, 128.2, 126.8, 126.8, 126.2, 124.5, 51.3, 46.8, 41.7, 21.2. **HR-ESI-MS** *m/z* calcd. for $C_{31}H_{28}ON$ [M-H]$^-$: 430.2176, found: 430.2175.

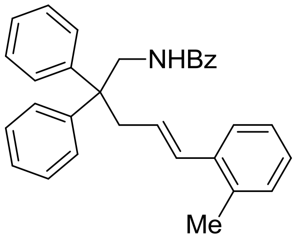

(*E*)-*N*-(2,2-Diphenyl-5-(o-tolyl)pent-4-en-1-yl)benzamide (1f): Purified by flash column chromatography using eluent (PE/EtOAc = 50/1; v/v) for the first step and eluent (PE/EtOAc = 10/1; v/v) for the last step. 67% overall yield. Viscous colourless oil. **IR** (KBr): 3427, 3028, 2925, 1668, 1510, 1484, 1287, 762, 704, 520 cm^{-1}. **^{1}H NMR** (400 MHz, $CDCl_3$) δ 7.52 (d, *J* = 7.7 Hz, 2H), 7.43 (t, *J* = 7.3 Hz, 1H),δ 7.39 – 7.29 (m, 9H), 7.28 (s, 2H), 7.24 (s, 1H), 7.21 – 7.15 (m, 1H), 7.09 – 7.03 (m, 3H), 6.46 (d, *J* = 15.6 Hz, 1H), 5.93 – 5.62 (m, 2H), 4.22 (d, *J* = 5.6 Hz, 2H), 3.09 (d, *J* = 7.2 Hz, 2H), 2.15 (s, 3H). **^{13}C NMR** (101 MHz, $CDCl_3$) δ 167.3, 145.4, 136.8, 135.3, 134.7, 132.1, 131.5, 130.1, 128.7, 128.6, 128.3, 127.1, 126.9, 126.8, 126.0, 125.9, 51.4, 46.8, 41.9, 19.8. **HR-ESI-MS** *m/z* calcd. for $C_{31}H_{28}ON$ [M-H]$^-$: 430.2176, found: 430.2175.

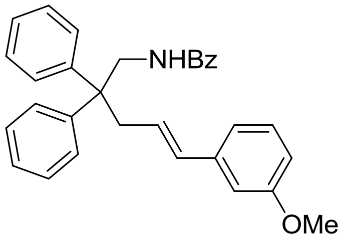

(*E*)-*N*-(5-(3-Methoxyphenyl)-2,2-diphenylpent-4-en-1-yl)benzamide (1g): Purified by flash column chromatography using eluent (PE/EtOAc = 20/1; v/v) for the first step and eluent (PE/EtOAc = 10/1; v/v) for the last step. 69% overall yield. White solid. mp: 97.5–99.4 ℃. **IR** (KBr): 3328, 3056, 2936, 1643, 1551, 1485, 1441, 1307, 1289, 1155, 967, 698 cm^{-1}. **^{1}H NMR** (400 MHz, $CDCl_3$) δ 7.51 (d, *J* = 7.7 Hz, 2H), 7.43 (t, *J* = 7.1 Hz, 1H), 7.39 – 7.24 (m, 12H), 7.13 (t, *J* = 7.7 Hz, 1H), 6.75 (dd, *J* = 27.4, 8.1 Hz, 1H), 6.70 (s, 2H), 6.29 (d, *J* = 15.8 Hz, 1H), 5.93 – 5.81 (m, 1H), 5.75 (s, 1H), 4.18 (t, *J* = 8.9 Hz, 2H), 3.75 (s, 3H), 3.06 (d, *J* = 7.2 Hz, 2H). **^{13}C NMR** (101 MHz, $CDCl_3$) δ 167.3, 159.8, 145.3, 139.0, 134.7, 133.8, 131.5, 129.4, 128.7, 128.6, 128.2, 126.9, 126.8, 126.0, 119.0, 112.8, 111.8, 55.3, 51.4, 46.9 41.7. **HR-ESI-MS** *m/z* calcd. for $C_{31}H_{28}O_2N$ [M-H]$^-$: 446.2126, found: 446.2124.

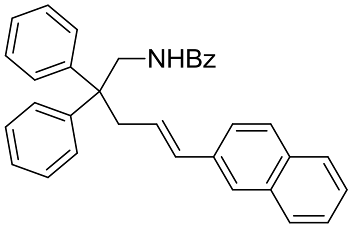

(*E*)-*N*-(5-(Naphthalen-2-yl)-2,2-diphenylpent-4-en-1-yl)benzamide (1h): Purified by flash column chromatography using eluent (PE/EtOAc = 30/1; v/v) for the first step and eluent (PE/EtOAc = 10/1; v/v) for

the last step. 29% overall yield. White solid. mp: 119.3–120.6 ℃. **IR** (KBr): 3444, 3082, 2924, 1667, 1523, 1487, 1444, 1284, 702, 509 cm^{-1}. **^{1}H NMR** (400 MHz, $CDCl_3$) δ 7.74 (m, 2H), 7.67 (d, *J* = 8.5 Hz, 1H), 7.53 (d, *J* = 9.1 Hz, 3H), 7.47 – 7.24 (m, 16H), 6.46 (d, *J* = 15.9 Hz, 1H), 6.15 – 5.93 (m, 1H), 5.77 (s, 1H), 4.23 (d, *J* = 5.6 Hz, 2H), 3.12 (d, *J* = 7.2 Hz, 2H). **^{13}C NMR** (101 MHz, $CDCl_3$) δ 167.3, 145.4, 135.0, 134.8, 134.0, 133.7, 132.9, 131.5, 128.7, 128.6, 128.3, 128.1, 128.0, 127.7, 126.9, 126.8, 126.2, 126.1, 125.9, 125.7, 123.8, 51.5, 46.8, 41.8. **HR-ESI-MS** *m/z* calcd. for $C_{34}H_{28}ON$ $[M-H]^-$: 466.2176, found: 466.2171.

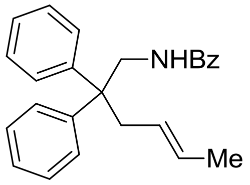

(*E*)-*N*-(2,2-Diphen.ylhex-4-en-1-yl)benzamide (1i): Purified by flash column chromatography using eluent (PE/EtOAc = 50/1; v/v) for the first step and eluent (PE/EtOAc = 12/1; v/v) for the last step. 61% overall yield. White solid, contains 9% Z isomer. mp: 96.9–97.8 ℃. **IR** (KBr): 3335, 3058, 2917, 1666, 1520, 1486, 1444, 1284, 970, 701, 529 cm^{-1}. **^{1}H NMR** (400 MHz, $CDCl_3$) δ 7.51 (d, *J* = 7.2 Hz, 2H), 7.43 (d, *J* = 7.3 Hz, 1H), 7.34 (dd, *J* = 13.1, 7.3 Hz, 6H), 7.25 (t, *J* = 6.0 Hz, 6H), 5.72 (s, 1H), 5.41 (dq, *J* = 12.9, 6.3 Hz, 1H), 5.08 (dt, *J* = 14.1, 6.5 Hz, 1H), 4.12 (d, *J* = 5.7 Hz, 2H), 2.86 (d, *J* = 7.0 Hz, 2H), 1.54 (d, *J* = 6.2 Hz, 3H). **^{13}C NMR** (101 MHz, $CDCl_3$) δ 167.3, 145.6, 134.8, 131.5, 129.6, 128.7, 128.5, 128.2, 126.8, 126.7, 126.0, 50.8, 46.5, 41.4, 18.2. **HR-ESI-MS** *m/z* calcd. for $C_{25}H_{24}ON$ $[M-H]^-$: 354.1863, found: 354.1863.

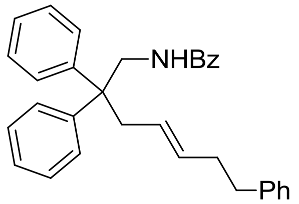

(*E*)-*N*-(2,2,7-Triphenylhept-4-en-1-yl)benzamide (1j): Purified by flash column chromatography using eluent (PE/EtOAc = 50/1; v/v) for the first step and eluent (PE/EtOAc = 12/1; v/v) for the last step. 41% overall yield. Colourless oil. **IR** (KBr): 3339, 3058, 2924, 1667, 1519, 1486, 1444, 1284, 973, 700, 529 cm^{-1}. **^{1}H NMR** (400 MHz, $CDCl_3$) δ 7.51 (d, *J* = 7.1 Hz, 2H), 7.44 (t, *J* = 7.4 Hz, 1H), 7.41 – 7.28 (m, 6H), 7.29 – 7.07 (m, 11H), 5.66 (s, 1H), 5.50 – 5.29 (m, 1H), 5.20 – 5.01 (m, 1H), 4.10 (d, *J* = 5.7 Hz, 2H), 2.83 (d, *J* = 7.0 Hz, 2H), 2.53 (t, *J* = 7.7 Hz, 2H), 2.20 (dd, *J* = 14.7, 7.2 Hz, 2H). **^{13}C NMR** (101 MHz, $CDCl_3$) δ 167.3, 145.5, 142.1, 134.8, 134.2, 131.5, 128.7, 128.6, 128.5, 128.3, 128.2, 126.8, 126.7, 125.8, 125.6, 50.9, 46.4, 41.14, 35.9, 34.6. **HR-ESI-MS** *m/z* calcd. for $C_{32}H_{30}ON$ $[M-H]^-$: 444.2333, found: 444.2328.

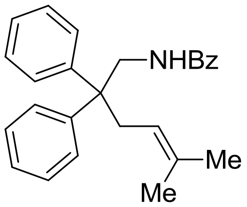

***N*-(5-Methyl-2,2-diphenylhex-4-en-1-yl)benzamide (1k):** Purified by flash column chromatography using eluent (PE/EtOAc = 50/1; v/v) for the first step and eluent (PE/EtOAc = 12/1; v/v) for the last step. 43% overall yield. White solid. mp: 104.7–106.4 ℃. **IR** (KBr): 3424, 3028, 2927, 1668, 1600, 1522, 1487, 1443, 1286, 702, 527 cm^{-1}. **^{1}H NMR** (400 MHz, $CDCl_3$) δ 7.54 – 7.49 (m, 2H), 7.44 (t, *J* = 7.3 Hz, 1H), 7.38 – 7.30 (m, 6H), 7.30 – 7.21 (m, 6H), 5.76 (s, 1H), 4.93 (t, *J* = 7.1 Hz, 1H), 4.12 (d, *J* = 5.5 Hz, 2H), 2.87 (d, *J* = 7.2 Hz, 2H), 1.58 (s, 3H), 1.37 (s, 3H). **^{13}C NMR** (101 MHz, $CDCl_3$) δ 167.3, 145.6, 135.3, 134.8, 131.5, 128.7, 128.4, 128.3, 126.8, 126.7, 119.2, 51.1, 46.9, 36.5, 26.1, 17.9. **HR-ESI-MS** *m/z* calcd. for $C_{26}H_{26}ON$ $[M-H]^-$: 368.2020, found: 368.2020.

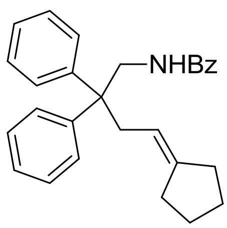

***N*-(4-Cyclopentylidene-2,2-diphenylbutyl)benzamide (1l):** Purified by flash column chromatography using eluent (PE/EtOAc = 50/1; v/v) for the first step and eluent (PE/EtOAc = 12/1; v/v) for the last step. 49% overall yield. White solid. mp: 46.6–47.5 ℃. **IR** (KBr): 3325, 3058, 2932, 1648, 1526, 1488, 1445, 1286, 700, 549 cm^{-1}. **^{1}H NMR** (400 MHz, $CDCl_3$) δ 7.51 (d, *J* = 7.3 Hz, 2H), 7.46 – 7.41 (m, 1H), 7.38 – 7.30 (m, 6H), 7.27 (d, *J* = 8.3 Hz, 6H), 5.80 (s, 1H), 5.08 (s, 1H), 4.14 (d, *J* = 5.5 Hz, 2H), 2.86 (d, *J* = 7.0 Hz, 2H), 2.11 (s, 2H), 1.89 (s, 2H), 1.53 – 1.42 (m, 4H). **^{13}C NMR** (101 MHz, $CDCl_3$) δ 167.3, 147.2, 145.6, 134.8, 131.5, 128.7, 128.4, 128.3, 126.8, 126.7, 114.9, 51.2, 47.1, 38.4, 33.9, 28.9, 26.3, 26.2. **HR-ESI-MS** *m/z* calcd. for $C_{28}H_{28}ON$ $[M-H]^-$: 394.2176, found: 394.2175.

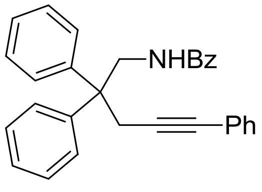

***N*-(2,2,5-Triphenylpent-4-yn-1-yl)benzamide (1m):** Prepared by using propynyl chloride instead of allyl chloride. Purified by flash column chromatography using eluent (PE/EtOAc = 30/1; v/v) for the first step and eluent (PE/EtOAc = 10/1; v/v) for the last step. 12% overall yield. White solid. mp: 39.4–42.8 ℃. **IR** (KBr): 3324, 3057, 2926, 2243, 1658, 1520, 1487, 1283, 694, 526 cm^{-1}. **^{1}H NMR** (400 MHz, $CDCl_3$) δ 7.50 (d, *J* = 7.7 Hz, 2H), 7.44 – 7.32 (m, 9H), 7.28 (d, *J* = 8.8 Hz, 3H), 7.25 (s, 2H), 7.21 (d, *J* = 8.0 Hz, 4H), 6.18 (s, 1H), 4.36 (d, *J* = 5.5 Hz, 2H), 3.29 (s, 2H). **^{13}C NMR** (101 MHz, $CDCl_3$) δ 167.4, 144.5, 134.6,

131.6, 131.5, 128.6, 128.5, 128.2, 128.2, 128.0, 127.1, 126.9, 123.4, 87.1, 84.5, 51.1, 48.2, 30.6. **HR-ESI-MS** *m/z* calcd. for $C_{30}H_{24}ON$ $[M-H]^-$: 414.1863, found: 414.1863.

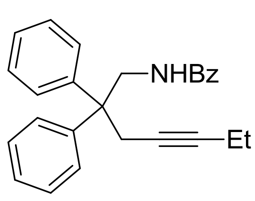

***N*-(2,2-Diphenylhept-4-yn-1-yl)benzamide (1n):** Prepared by using propynyl bromide instead of allyl chloride. Purified by flash column chromatography using eluent (PE/EtOAc = 50/1; v/v) for the first step and eluent (PE/EtOAc = 12/1; v/v) for the last step. 71% overall yield. White solid. mp: 121.9–123.9 ℃. **IR** (KBr): 3416, 3067, 2932, 2236, 1665, 1519, 1487, 1289, 703, 551 cm^{-1}. **^{1}H NMR** (400 MHz, $CDCl_3$) δ 7.52 (d, *J* = 7.2 Hz, 2H), 7.43 (t, *J* = 7.3 Hz, 1H), 7.38 – 7.22 (m, 12H), 6.20 (s, 1H), 4.29 (d, *J* = 5.6 Hz, 2H), 3.03 (s, 2H), 2.02 (q, *J* = 7.4 Hz, 2H), 0.95 (t, *J* = 7.5 Hz, 3H). **^{13}C NMR** (101 MHz, $CDCl_3$) δ 167.4, 144.7, 134.9, 131.5, 128.6, 128.4, 128.1, 126.9, 126.8, 85.9, 76.7, 50.7, 47.8, 30.0, 14.0, 12.5. **HR-ESI-MS** *m/z* calcd. for $C_{26}H_{24}ON$ $[M-H]^-$: 366.1863, found: 366.1857.

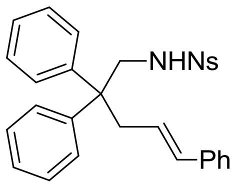

(*E*)-4-Nitro-*N*-(2,2,5-triphenylpent-4-en-1-yl)benzenesulfonamide (1o): Prepared by using NsCl (0.33 g, 1.5 mmol) instead of BzCl. Purified by flash column chromatography using eluent (PE/EtOAc = 30/1; v/v) for the first step and eluent (PE/EtOAc = 6/1; v/v) for the last step. 38% overall yield. White solid. mp: 160.9–162.1 ℃. **IR** (KBr): 3326, 3022, 1810, 1605, 1525, 1348, 1310, 1159, 1095, 758, 504 cm^{-1}. **^{1}H NMR** (400 MHz, $CDCl_3$) δ 8.22 (d, *J* = 8.3 Hz, 2H), 7.84 (d, *J* = 8.3 Hz, 2H), 7.35 – 7.02 (m, 15H), 6.28 (d, *J* = 15.8 Hz, 1H), 5.69 – 5.55 (m, 1H), 4.17 (t, *J* = 5.6 Hz, 1H), 3.63 (d, *J* = 5.9 Hz, 2H), 3.03 (d, *J* = 7.1 Hz, 2H). **^{13}C NMR** (101 MHz, $CDCl_3$) δ 150.4, 145.2, 144.3, 137.2, 134.3, 128.8, 128.6, 128.4, 127.8, 127.5, 127.2, 126.2, 124.5, 124.5, 50.1, 49.6, 40.4. **HR-ESI-MS** *m/z* calcd. for $C_{29}H_{25}O_4N_2S$ $[M-H]^-$: 497.1541, found: 497.1537.

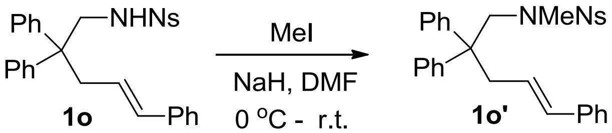

A solution of **1o** (0.50 g, 1.0 mmol) in DMF (5 mL) was added slowly to a suspension of NaH (60.0 mg, 1.5 mmol) in DMF (2 mL) at 0 °C. The resulting mixture was stirred at room temperature for 0.5 h, after which it was cooled to 0 °C,

treated with MeI (95 μL, 1.5 mmol), warmed to room temperature and stirred for 12 h. Then the mixture was poured into ice/water (15 mL) and was extracted with Et_2O (15 mL x 3). The combined organic layers were washed with water (10 mL x 3), dried with Na_2SO_4, and concentrated to give the crude product **1o'**, which was purified by flash column chromatography on silica gel (PE/EtOAc = 10/1; v/v).

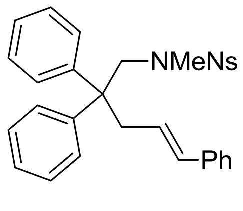

(*E*)-*N*-Methyl-4-nitro-*N*-(2,2,5-triphenylpent-4-en-1-yl)benzenesulfonamide (1o'): 90% yield. White solid. mp: 138.8–139.6 ℃. **IR** (KBr): 3326, 3024, 1807, 1603, 1532, 1350, 1164, 760 cm^{-1}. **^{1}H NMR** (400 MHz, $CDCl_3$) δ 8.43 – 8.30 (m, 2H), 7.98 – 7.92 (m, 2H), 7.32 – 7.26 (m, 4H), 7.26 – 7.18 (m, 8H), 7.17 – 7.12 (m, 3H), 6.29 (d, *J* = 15.9 Hz, 1H), 6.09 – 5.89 (m, 1H), 3.84 (s, 2H), 3.26 (d, *J* = 7.0 Hz, 2H), 1.92 (s, 3H). **^{13}C NMR** (101 MHz, $CDCl_3$) δ 150.26, 145.59, 143.38, 138.01, 133.82, 128.93, 128.62, 128.47, 128.36, 127.01, 126.87, 126.19, 125.94, 124.52, 59.32, 51.29, 40.43, 37.74. **HR-ESI-MS** *m/z* calcd. for $C_{30}H_{29}O_4N_2S$ $[M+H]^+$: 513.1843, found: 513.1834.

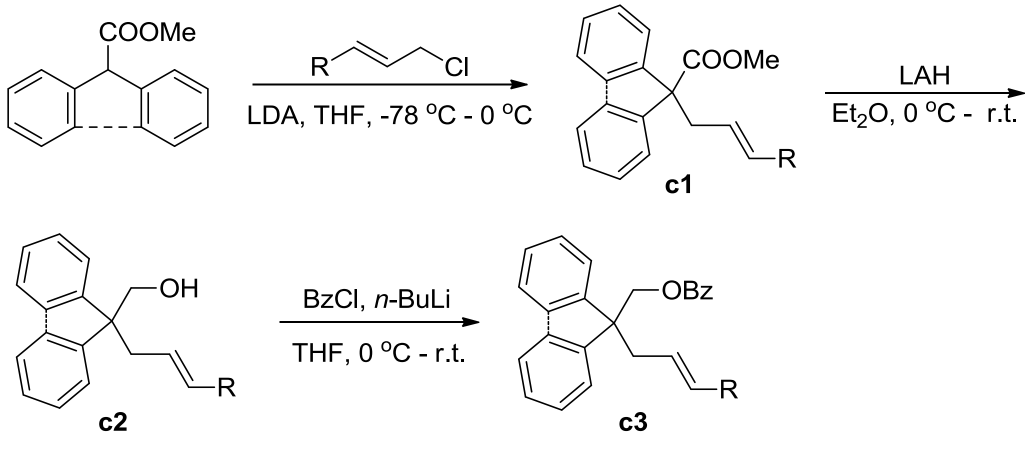

To a stirred solution of LDA (7.0 mmol, 1M in THF) at -78 ℃ was added methyl 2,2-diphenylacetate (1.3 g, 5.8 mmol) in THF (4 mL) dropwise over 15 min. After the solution stirred at -78 ℃ for an additional 30 min, allyl chloride (6.4 mmol) was added dropwise over 10 min. The reaction was allowed to stir at -78 ℃ for 45 min and then warm up to room temperature. After stirred for 12 h, the reaction was quenched by the addition of 1 M HCl (15 mL). The aqueous layer was extracted with

EtOAc (15 mL x 3). The combined organic layers were washed with brine, dried with Na_2SO_4, and concentrated. The crude residue was purified by silica gel chromatography (PE/EtOAc = 60/1; v/v) to provide the ester **c1** as a colorless oil.

To a suspension of $LiAlH_4$ (0.1 g, 2.0 mmol) in THF (3 mL) was added a solution of **c1** (2.0 mmol) in 3 mL THF at 0 ^oC. The mixture was warmed to room temperature and stirred for another 2.5 h. After TLC revealed the consumption of the starting material, the reaction was quenched with cold solution of 10% NaOH carefully at 0 ^oC. The resulting mixture was filtered through Celite. And after evaporation of the solvent the crude alcohol **c2** was obtained and used directly to the next step without further purification.

A solution of **c2** (1.0 mmol) in THF (1 mL) was cooled down to 0 ^oC and *n*-BuLi (1.1 mmol, 2.4 M in hexane) was added dropwise. After 15 min, allyl chloride (1.1 mmol) in THF (1 mL) was added dropwise. The mixture was allowed to stir at 0 ^oC for 1 h and then at room temperature overnight. Afterwards, the mixture was quenched by the addition of an aqueous saturated solution of NH_4Cl. The resulting mixture was extracted with DCM (5 mL x 3) and the combined organic layers were washed with brine, dried over Na_2SO_4. The solvent was evaporated under reduced pressure and the crude product was purified by chromatography (PE/EtOAc = 60/1; v/v) to afford the final product **c3**.

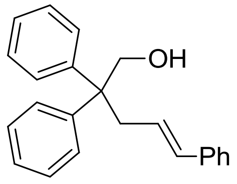

(*E*)-2,2,5-Triphenylpent-4-en-1-ol (1p): Purified by flash column chromatography using eluent (PE/EtOAc = 60/1; v/v) for the first step and eluent (PE/EtOAc = 20/1; v/v) for the second step. 56% overall yield. White solid. mp: 68.3–69.7 ℃. **IR** (KBr): 3315, 3021, 2951, 1650, 1596, 1493, 1444, 1067, 966, 743, 696 cm^{-1}. ^{1}**H NMR** (400 MHz, $CDCl_3$) δ 7.31 (t, *J* = 7.3 Hz, 4H), 7.28 – 7.13 (m, 11H), 6.43 (d, *J* = 15.8 Hz, 1H), 5.87 – 5.67 (m, 1H), 4.18 (d, *J* = 6.5 Hz, 2H), 3.10 (d, *J* = 7.2 Hz, 2H), 1.19 (t, *J* = 6.6 Hz, 1H). ^{13}**C NMR** (101 MHz, $CDCl_3$) δ 145.4, 137.6, 133.3, 128.6, 128.4, 128.3, 127.2, 126.6, 126.4, 126.2, 68.1, 52.3, 40.2. **HR-EI-MS** *m/z* calcd. for $C_{23}H_{32}O$ $[M]^+$: 314.1665, found: 314.1667.

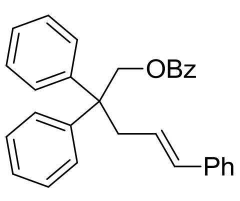

(*E*)-2,2,5-Triphenylpent-4-en-1-yl benzoate (1q): Purified by flash column chromatography using eluent (PE/EtOAc = 60/1; v/v) for the last step. 53% yield. White solid. mp: 38.0–40.1 ℃. **IR** (KBr): 3058, 2953, 1600, 1494, 1446, 1272, 1114, 749, 698 cm^{-1}. **^{1}H NMR** (400 MHz, $CDCl_3$) δ 7.86 (d, *J* = 7.2 Hz, 2H), 7.50 (t, *J* = 7.4 Hz, 1H), 7.42 – 7.07 (m, 17H), 6.36 (d, *J* = 15.8 Hz, 1H), 5.92 – 5.75 (m, 1H), 4.91 (s, 2H), 3.23 (d, *J* = 7.2 Hz, 2H). **^{13}C NMR** (101 MHz, $CDCl_3$) δ 166.4, 145.3, 137.4, 133.6, 133.0, 130.2, 129.6, 128.1, 128.5, 128.3, 128.1, 127.2, 126.6, 126.2, 125.8, 69.6, 50.4, 41.2. **HR-ESI-MS** *m/z* calcd. for $C_{30}H_{26}O_2Na$ $[M+Na]^+$: 441.2815, found: 441.1821.

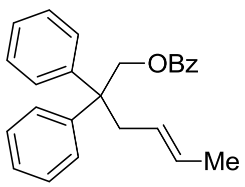

(*E*)-2,2-Diphenylhex-4-en-1-yl benzoate (1r): Purified by flash column chromatography using eluent (PE/EtOAc = 60/1; v/v) for the first step and eluent (PE/EtOAc = 60/1; v/v) for the last step. 47% overall yield. White solid. 63% overall yield.White solid, contains 8% Z isomer. mp: 67.4–69.5 ℃. **IR** (KBr): 3060, 2936, 1601, 1496, 1447, 1272, 1116, 700 cm^{-1}. **^{1}H NMR** (400 MHz, $CDCl_3$) δ 7.86 (d, *J* = 7.2 Hz, 2H), 7.50 (t, *J* = 7.4 Hz, 1H), 7.37 (t, *J* = 7.7 Hz, 2H), 7.33 – 7.17 (m, 10H), 5.55 – 5.32 (m, 1H), 5.19 – 4.99 (m, 1H), 4.85 (s, 2H), 3.01 (d, *J* = 7.0 Hz, 2H), 1.52 (d, *J* = 6.1 Hz, 3H). **^{13}C NMR** (101 MHz, $CDCl_3$) δ 166.4, 145.6 133.0, 130.3, 129.6, 129.3, 128.4, 128.2, 128.1, 126.4, 126.2, 69.5, 49.9, 40.7, 18.1. **HR-ESI-MS** *m/z* calcd. for $C_{25}H_{24}O_2Na$ $[M+Na]^+$: 379.1669, found: 379.1668.

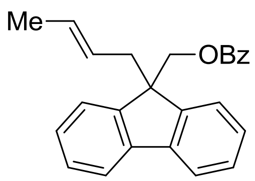

(*E*)-(9-(But-2-en-1-yl)-9H-fluoren-9-yl)methyl benzoate (1z): Purified by flash column chromatography using eluent (PE/EtOAc = 60/1; v/v) for the first step and eluent (PE/EtOAc = 60/1; v/v) for the last step. 68% overall yield. White solid. White solid, contains 5% Z isomer. mp: 74.3–77.6 ℃. **IR** (KBr): 3026, 2933, 1600, 1447, 1275, 1119, 962, 709 cm^{-1}. **1H NMR** (400 MHz, $CDCl_3$) δ 7.98 (d, *J* = 7.4 Hz, 2H), 7.75 (d, *J* = 7.5 Hz, 2H), 7.55 (d, *J* = 7.3 Hz, 3H), 7.44 (t, *J* = 7.6 Hz, 2H), 7.38 (t, *J* = 7.3 Hz, 2H), 7.30 (t, *J* = 7.3 Hz, 2H), 5.36 (dq, *J* = 12.9, 6.3 Hz, 1H), 5.15 – 5.01 (m, 1H), 4.49 (s, 2H), 2.81 (d, *J* = 7.1 Hz, 2H), 1.45 (d, *J* = 6.0 Hz, 3H). **13C NMR** (101 MHz, $CDCl_3$) δ 166.5, 147.5, 140.9, 133.1, 130.4, 129.7, 129.2, 128.6, 127.9, 127.3, 125.4, 124.5, 120.1, 69.7, 54.2, 38.5, 18.0. **HR-ESI-MS** *m/z* calcd. for $C_{25}H_{22}O_2Na$ $[M+Na]^+$: 377.1512, found: 377.1509.

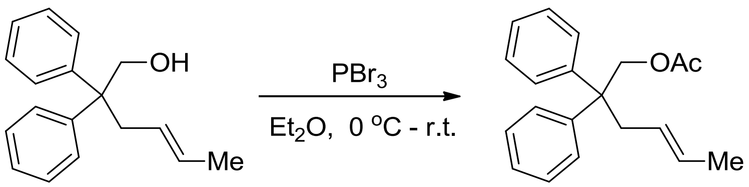

1s

A solution of alcohol (675 mg, 2.7 mmol) in anhydrous ether (18 mL) was treated with phosphorus tribromide (84.0 μL, 0.9 mmol) at 0 ^{o}C under a N_2 atmosphere. The reaction mixture was stirred at room temperature overnight and poured into ice water containing sodium bicarbonate. The resulting mixture was extracted with EtOAc (15 mL x 3). The combined organic layers were washed with brine, dried over Na_2SO_4. Then the solvent was evaporated under reduced pressure and the crude product was purified by chromatography (PE/EtOAc = 60/1; v/v) to afford the final product **1s**.

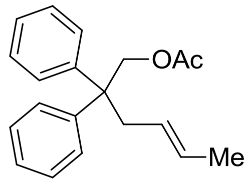

(*E*)-2,2-Diphenylhex-4-en-1-yl acetate (1s): 22% yield. White solid. Colourless oil, contains 9% Z isomer. **IR** (KBr): 3027, 2917, 1600, 1497, 1446, 1380, 1234, 1043, 700 cm^{-1}. **^{1}H NMR** (400 MHz, $CDCl_3$) δ 7.32 – 7.23 (m, 4H), 7.24 – 7.13 (m, 6H), 5.53 – 5.33 (m, 1H), 5.11 – 4.94 (m, 1H), 4.62 (s, 2H), 2.90 (d, *J* = 7.0 Hz, 2H), 1.90 (s, 3H), 1.55 (d, *J* = 6.1 Hz, 3H). **^{13}C NMR** (101 MHz, $CDCl_3$) δ 171.0, 145.5, 129.1, 128.1, 128.1, 126.4, 126.4, 126.3, 68.8, 49.7, 40.5, 21.0, 18.1. **HR-ESI-MS** *m/z* calcd. for $C_{20}H_{22}O_2Na$ [M+Na]$^+$: 317.1512, found: 317.1509.

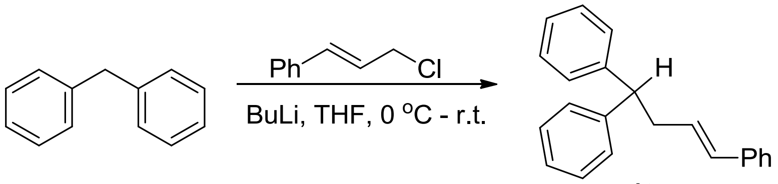

1t

To a solution of diphenylmethane (505 mg, 3.0 mmol) in THF (25 mL) was added *n*-BuLi (1.4 mL, 2.4 M in hexane) at room temperature. After 2 h, cinnamyl chloride (460 μL, 3.3 mmol) was added and the reaction mixture was stirred at room temperature overnight and poured into 1M HCl (20 mL). The resulting mixture was extracted with EtOAc (15 mL x 3). The combined organic layers were washed with brine, dried over Na_2SO_4. Then the solvent was evaporated under reduced pressure

and the crude product was purified by chromatography (PE) to afford the final product **1t**.

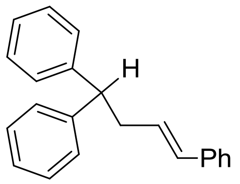

(*E*)-But-3-ene-1,1,4-triyltribenzene (1t): 86% yield. White solid. mp 75.7–77.7 ℃. **IR** (KBr): 3025, 2922, 1596, 1490, 1448, 967, 741, 698 cm^{-1}. **^{1}H NMR** (400 MHz, $CDCl_3$) δ 7.39 – 7.08 (m, 15H), 6.39 (d, *J* = 15.8 Hz, 1H), 6.20 – 6.01 (m, 1H), 4.08 (t, *J* = 7.7 Hz, 1H), 2.96 (t, *J* = 7.3 Hz, 2H). **^{13}C NMR** (101 MHz, $CDCl_3$) δ 144.6, 137.8, 131.7, 128.8, 128.6, 128.6, 128.1, 127.1, 126.4, 126.2, 51.8, 39.4. **HR-EI-MS** *m/z* calcd. for $C_{22}H_{20}$ $[M]^+$: 284.1560, found: 284.1557.

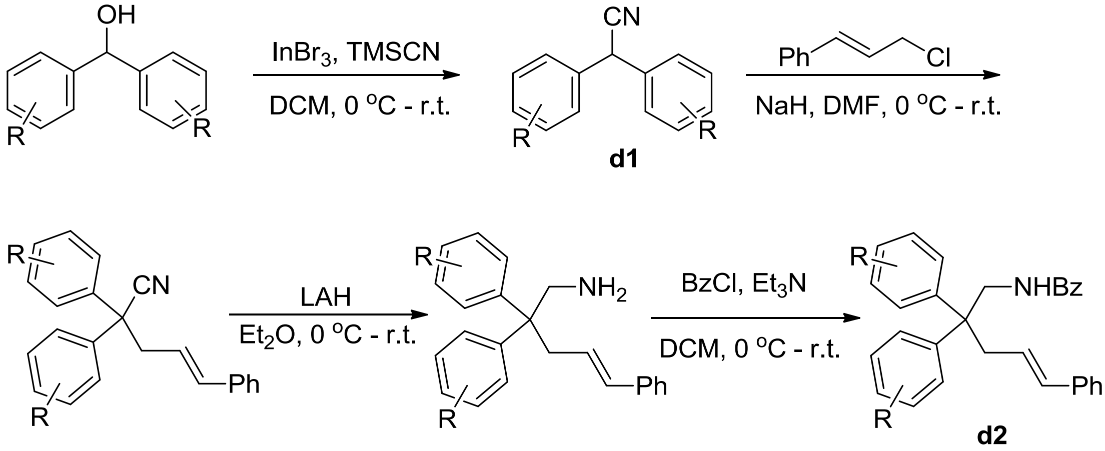

Alcohol (6.0 mmol) was added to a mixture of TMSCN (1.5 mL, 12.0 mmol) and $InBr_3$ (213 mg, 10 mol%) at 0 °C in DCM (15 mL) under an atmosphere of nitrogen, and the reaction mixture was stirred at room temperature overnight. After full conversion, the reaction was quenched with aqueous saturated solution of $NaHCO_3$. The aqueous layer was extracted with DCM (15 mL x 3), and the combined organic layers were dried over Na_2SO_4 and filtrated. The solvent was removed under reduced pressure and the product **d1** was purified by flash column chromatography on silica gel (PE/EtOAc = 30/1; v/v) and used to the further reaction.

The final product **d2** was synthesized from nitrile **d1** using the same method for the synthesis of **b3** described above.

Note: Product **d1** may be hard to dissolve, for this case you can use DCM to elute out the product and use $CHCl_3$/THF to recrystallize to get crude product, which can be directly use to next step.

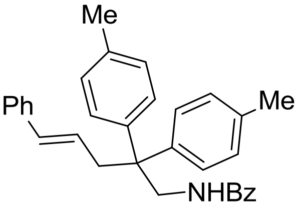

(*E*)-*N*-(5-Phenyl-2,2-di-p-tolylpent-4-en-1-yl)benzamide (1u): Flash column chromatography using eluent (PE/EtOAc = 30/1 to 10/1; v/v) for the first step to quickly get the crude product and eluent (PE/EtOAc = 50/1; v/v) for the second step. The product was finally purified in the last step by flash column chromatography (PE/EtOAc = 10/1; v/v) in 32% overall yield. White solid. mp: 126.8–130.2 ℃. **IR** (KBr): 3312, 3022, 2918, 1646, 1513, 1482, 1287, 716, 696 cm^{-1}. **^{1}H NMR** (400 MHz, $CDCl_3$) δ 7.52 (d, *J* = 7.5 Hz, 2H), 7.43 (t, *J* = 7.2 Hz, 1H), 7.33 (t, *J* = 7.4 Hz, 2H), 7.26 – 7.11 (m, 13H), 6.32 (d, *J* = 15.8 Hz, 1H), 5.96 – 5.83 (m, 1H), 5.79 (s, 1H), 4.14 (d, *J* = 5.5 Hz, 2H), 3.03 (d, *J* = 7.2 Hz, 2H), 2.34 (s, 6H). **^{13}C NMR** (101 MHz, $CDCl_3$) δ 167.3, 142.32 137.6, 136.3, 134.8, 133.7, 131.4, 129.3, 128.6, 128.5, 128.1, 127.1, 126.8, 126.3, 125.9, 50.6, 46.9, 41.8, 21.1. **HR-ESI-MS** *m/z* calcd. for $C_{32}H_{30}ON$ $[M-H]^-$: 444.2333, found: 444.2329.

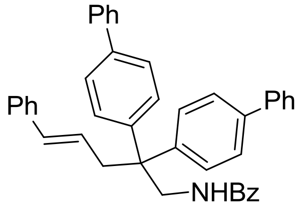

(*E*)-*N*-(2,2-Di([1,1'-biphenyl]-4-yl)-5-phenylpent-4-en-1-yl)benzamide (1v): Flash column chromatography using eluent (DCM) for the first step to quickly get the crude product and eluent (PE/EtOAc = 50/1; v/v) for the second step (maybe still impure). The product was finally purified in the last step by flash column chromatography (PE/EtOAc = 10/1; v/v) in 38% overall yield. White solid. mp: 138.1–141.3 ℃. **IR** (KBr): 3421, 3026, 2923, 1664, 1561, 1485, 1285, 693 cm^{-1}. **^{1}H NMR** (400 MHz, $CDCl_3$) δ 7.62 (d, *J* = 5.4 Hz, 8H), 7.55 (d, *J* = 7.3 Hz, 2H), 7.49 – 7.38 (m, 9H), 7.38 – 7.32 (m, 4H), 7.19 (dd, *J* = 20.4, 6.6 Hz, 5H), 6.38 (d, *J* = 15.8 Hz, 1H), 6.04 – 5.92 (m, 1H), 5.86 (s, 1H), 4.27 (d, *J* = 5.5 Hz, 2H), 3.14 (d, *J* = 7.1 Hz, 2H). **^{13}C NMR** (101 MHz, $CDCl_3$) δ 167.4, 144.3, 140.5, 139.6, 137.5, 134.7, 134.1, 131.5, 129.0, 128.7, 128.5, 127.5, 127.3, 127.2, 127.1, 126.9, 126.4, 125.5, 51.1, 47.0, 42.0. **HR-ESI-MS** *m/z* calcd. for $C_{42}H_{34}ON$ $[M-H]^-$: 568.2646, found: 568.2642.

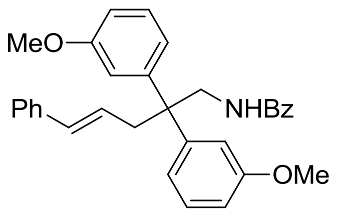

(*E*)-*N*-(2,2-Bis(3-methoxyphenyl)-5-phenylpent-4-en-1-yl)benzamide (1w): Flash column chromatography using eluent (PE/EtOAc = 30/1 to 10/1; v/v) for the first step to quickly get the crude product and eluent (PE/EtOAc = 50/1; v/v) for the second step. The product was finally purified in the last step by flash column chromatography (PE/EtOAc = 10/1; v/v) in 32% overall yield. White solid. mp: 50.2–52.4 ℃. **IR** (KBr): 3339, 3026, 2933, 1664, 1601, 1580, 1521, 1487, 1430, 1290, 1252, 1043, 694 cm^{-1}. **^{1}H NMR** (400 MHz, $CDCl_3$) δ 7.53 (d, *J* = 7.6 Hz, 2H), 7.43 (t, *J* = 7.2 Hz, 1H), 7.33 (t, *J* = 7.6 Hz, 2H), 7.30 – 7.26 (m, 2H), 7.24 – 7.12 (m, 5H), 6.88 (d, *J* = 7.8 Hz, 2H), 6.85 – 6.77 (m, 4H), 6.34 (d, *J* = 15.8 Hz, 1H), 5.96 – 5.85 (m, 1H), 5.81 (s, 1H), 4.16 (d, *J* = 5.7 Hz, 2H), 3.75 (s, 6H), 3.04 (d, *J* = 7.2 Hz, 2H). **^{13}C NMR** (101 MHz, $CDCl_3$) δ 167.3, 159.8, 146.9, 137.6, 134.8, 133.8, 131.5, 129.5, 128.7, 128.5, 127.2, 126.8, 126.3, 125.7, 120.6, 114.5, 112.0, 55.4, 51.4, 46.8, 41.7. **HR-ESI-MS** *m/z* calcd. for $C_{32}H_{30}O_3N$ [M-H]$^-$: 476.2231, found: 476.2228.

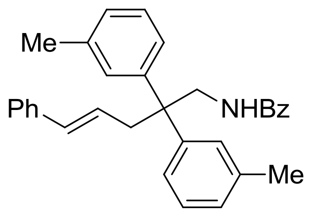

(*E*)-*N*-(5-Phenyl-2,2-di-m-tolylpent-4-en-1-yl)benzamide (1x): Flash column chromatography using eluent (PE/EtOAc = 30/1 to 10/1; v/v) for the first step to quickly get the crude product and eluent (PE/EtOAc = 50/1; v/v) for the second step. The product was finally purified in the last step by flash column chromatography (PE/EtOAc = 10/1; v/v) in 18% overall yield. Colourless oil. **IR** (KBr): 3339, 3026, 2920, 1663, 1520, 1485, 1283, 968, 709, 693 cm^{-1}. **^{1}H NMR** (400 MHz, $CDCl_3$) δ 7.52 (d, *J* = 7.3 Hz, 2H), 7.43 (t, *J* = 7.3 Hz, 1H), 7.33 (t, *J* = 7.5 Hz, 2H), 7.27 – 7.14 (m, 7H), 7.08 (m, 6H), 6.32 (d, *J* = 15.8 Hz, 1H), 5.92 – 5.80 (m, 1H), 5.75 (s, 1H), 4.15 (d, *J* = 5.6 Hz, 2H), 3.04 (d, *J* = 7.2 Hz, 2H), 2.33 (s, 6H). **^{13}C NMR** (101 MHz, $CDCl_3$) δ 167.2, 145.3, 138.1, 137.7, 134.9, 133.8, 131.4, 128.9, 128.7, 128.5, 128.4, 127.6, 127.1, 126.8, 126.3, 125.9, 125.3, 51.1, 46.8, 41.6, 21.8. **HR-ESI-MS** *m/z* calcd. for $C_{32}H_{30}NO$ [M-H]$^-$: 444.2333, found: 444.2332.

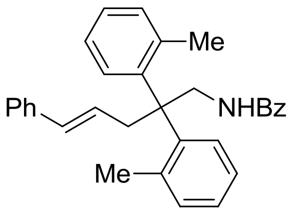

(*E*)-*N*-(5-Phenyl-2,2-di-o-tolylpent-4-en-1-yl)benzamide (1y): Flash column chromatography using eluent (PE/EtOAc = 30/1 to 10/1; v/v) for the first step to quickly get the crude product and eluent (PE/EtOAc = 50/1; v/v) for the second step. The product was finally purified in the last step by flash column chromatography (PE/EtOAc = 10/1; v/v) in 22% overall yield. Viscous colourless oil. **IR** 3263, 3057, 2940, 1638, 1550, 1487, 1447, 1311, 960, 726, 710, 691 (KBr): cm^{-1}. **^{1}H NMR** (400 MHz, $CDCl_3$) δ 7.61 – 7.26 (m, 9H), 7.25 – 7.17 (m, 4H), 7.17 – 7.12 (m, 3H), 7.10 (d, *J* = 7.2 Hz, 2H), 6.33 (d, *J* = 15.8 Hz, 1H), 5.78 – 5.62 (m, 1H), 5.55 (s, 1H), 4.80 (br, 1H), 3.81 (br, 1H), 3.10 (d, *J* = 7.2 Hz, 2H), 1.77 (s, 6H). **^{13}C NMR** (101 MHz, $CDCl_3$) δ 167.3, 142.6 – 142.1 (br), 137.8, 134.8, 133.9, 133.5– 132.8 (br), 131.5, 128.7, 128.4, 127.5 – 127.0 (br), 126.9, 126.4, 126.3, 126.1, 50.7, 43.2, 38.2, 21.8. **HR-ESI-MS** *m/z* calcd. for $C_{32}H_{30}ON$ $[M-H]^-$: 444.2333, found: 444.2328.

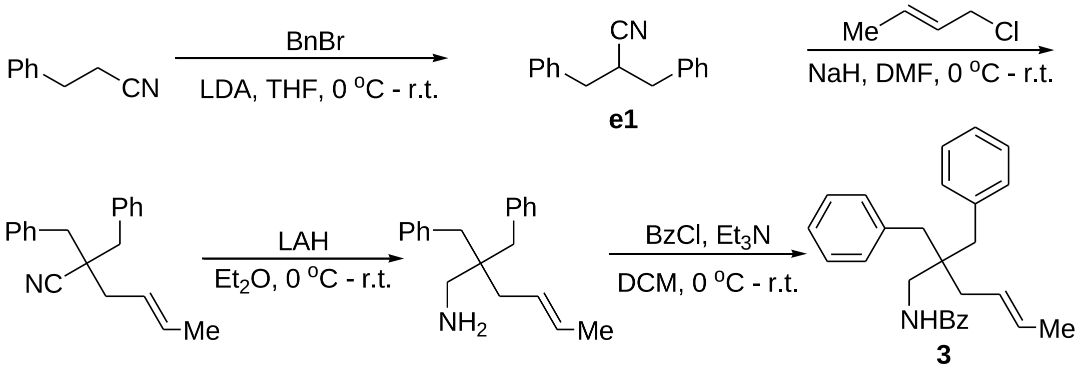

In a 50 mL Schlenk flask, nitrile (10.0 mmol) in dry THF (5.0 mL) was added dropwise to a solution of LDA (10.0 mmol, 1M THF) at −78 °C by means of a syringe. After stirred for 10 min at −78 °C, the solution was stirred at 0 °C for 5 min. Then the solution was cooled to −78 °C again and a solution of BnBr (10.5 mmol) in dry THF (5.0 mL) was added dropwise. The reaction mixture was stirred at −78 °C for 1 h and at room temperature for 1 h. After complete conversion (as monitored by TLC), saturated NH_4Cl solution was added and the solution was extracted with EtOAc (10 mL x 3). The combined organic phases were dried over Na_2SO_4 and the solvent was removed under reduced pressure. The crude product was purified by flash column chromatography (PE/EtOAc = 50/1; v/v) to afford **e1**.

The final product **3** was synthesized from nitrile **e1** using the same method for the synthesis of **b3** described above.

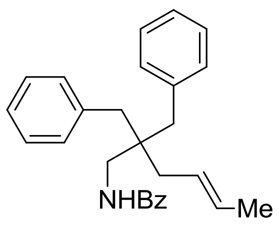

(*E*)-*N*-(2,2-Dibenzylhex-4-en-1-yl)benzamide (3): 76% yield for the first step, 59% for the second step and 71% for the last two steps. White solid. mp: 66.2–67.3 ℃. **IR** (KBr): 3345, 3058, 2933, 1635, 1537, 1448, 1286, 969, 708 cm^{-1}. **^{1}H NMR** (400 MHz, $CDCl_3$) δ 7.41 (t, *J* = 7.2 Hz, 1H), 7.36 – 7.21 (m, 14H), 5.75 – 5.50 (m, 3H), 3.49 (d, *J* = 5.9 Hz, 2H), 2.75 (q, *J* = 13.5 Hz, 4H), 2.18 (d, *J* = 6.9 Hz, 2H), 1.73 (d, *J* = 5.6 Hz, 3H). **^{13}C NMR** (101 MHz, $CDCl_3$) δ 166. 8, 138.3, 134.5, 131.3, 130.8, 129.5, 128.6, 128.4, 126.9, 126.7, 126.7, 46.5, 44.1, 42.1, 38.5, 18.4. **HR-ESI-MS** *m/z* calcd. for $C_{27}H_{28}ON$ $[M-H]^-$: 382.2176, found: 382.2172.

Preparation of sulfenyl reagent 8a

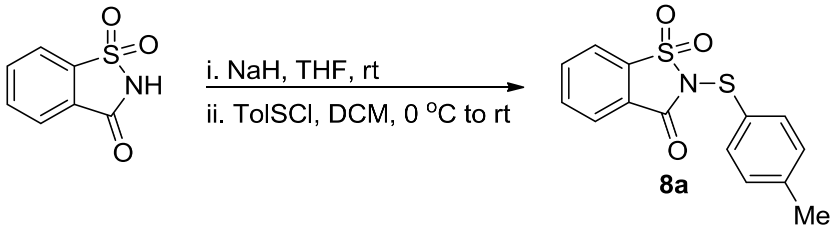

8a

A modified procedure was used according to the literature[9].

To a suspension of NaH (0.22 g, 5.5 mmol, 1.1 equiv, 60%) in THF (15 mL) at rt was carefully added a solution of saccharin (0.92 g, 5.0 mmol) in THF (10 mL) dropwise over 5 min. The resulting white suspension was stirred at rt for 16 h before being concentrated in vacuo. Then anhydrous DCM (15 mL) was added and the reaction solution was cooled to 0 °C. A solution of 4-methylbenzenesulfenyl chloride (6.4 mL, 5.0 mmol, 1.0 equiv) in DCM (5 mL) was added dropwise over 3 min. The resulting solution was stirred for 1 h at 0 ℃ and stirred a further 2 h at rt before being concentrated in vacuo. Acetone (20 mL) was added and the suspension stirred for 1 h. Stirring was then stopped and the fine precipitate allowed to settle for 30 min. The suspension was then filtered through celite to give a pale yellow solution which was concentrated in vacuo. The residue was added 25 mL DCM to dissolve the product

while most sachharin remains undissolved. Solvent was carefully transferred and concentrated in vacuo to afford a yellow solid which was recrystallized by hexane/DCM afford a pale white solid.

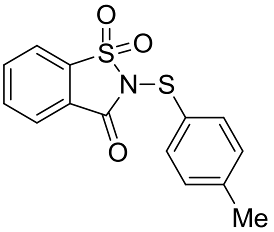

2-(*p*-Tolylthio)benzo[d]isothiazol-3(2H)-one 1,1-dioxide (8a): 34% yield. Pale white solid. mp: 160.2–160.9 ℃. **IR** (KBr): 3090, 2926, 1743, 1721, 1590, 1457, 1347, 1193, 1120, 947, 750 cm^{-1}. **^{1}H NMR** (400 MHz, $CDCl_3$) δ 8.06 (d, *J* = 7.6 Hz, 1H), 7.96 – 7.71 (m, 5H), 7.17 (d, *J* = 7.9 Hz, 2H), 2.33 (s, 3H). **^{13}C NMR** (101 MHz, $CDCl_3$) δ = 159.48, 141.64, 138.13, 135.47, 134.46, 130.12, 129.56, 127.23, 125.84, 121.60, 21.50. **HR-EI-MS** *m/z* calcd. for $C_{14}H_{11}O_3NS_2$ $[M]^+$: 305.0175, found: 305.0179.

Chiral selenide catalyzed enantioselective desymmetrization **Method A**:

To a solution of olefin (0.1 mmol), $(PhSO_2)_2N\text{-}SCF_3$ (59.8 mg, 0.15 mmol) and catalyst **C7** (9.3 mg, 20 mol%) in solvent (DCM 2 mL, DCE 2 mL) at -78 ^{o}C was added TMSOTf (18.0 *μ*L, 0.1 mmol). The resultant mixture was stirred at -78 ^{o}C for 12 h, and then quenched with MeOH (0.2 mL) and Et_3N (0.2 mL), and concentrated *in vacuo*. The residue was purified by flash silica gel column chromatography to yield the corresponding CF_3S-product.

Gram scale experiment: To a solution of **1a** (1.0 g, 2.4 mmol), $(PhSO_2)_2N\text{-}SCF_3$ (1.5 g, 3.6 mmol) and catalyst **C7** (22.2 mg, 2 mol%) in solvent (DCM 20 mL, DCE 20 mL) at -78 ^{o}C was added TMSOTf (1.1 mL, 6.0 mmol). The resultant mixture was stirred at -78 ^{o}C for 3 d, and then quenched with MeOH (5.0 mL) and Et_3N (5.0 mL). The resulting solution was treated with water (30 mL), and was extracted with DCM (30 mL x 3). The combined organic layers were dried over Na_2SO_4 and concentrated. The residue was purified by flash silica gel column chromatography to afford product **2a** (1.23 g, 99%, 99.2% ee, *dr* = 50:1).

Chiral selenide catalyzed enantioselective sulfenocyclization Method B:

To a solution of olefin **5** (17.8 mg, 0.1 mmol), saccharin-STol (36.6 mg, 0.12 mmol) and catalyst **C7** (9.3 mg, 20 mol%) in solvent (DCM 4 mL) at -78 ^{o}C was added TMSOTf (18.0 μL, 0.1 mmol). The resultant mixture was stirred at -78 ^{o}C for 12 h, and then quenched by saturated $NaHCO_3$ (1 mL) and then extracted with dichloromethane (8 mL x 4). The combined organic phases were concentrated concentrated *in vacuo*. The residue was purified by flash silica gel column chromatography to yield the corresponding TolS-product **7** (67%, 92% ee, 9:1 *dr*).

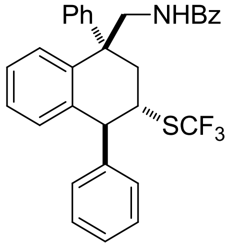

***N*-(((1*R*,3*S*,4*S*)-1,4-Diphenyl-3-((trifluoromethyl)thio)-1,2,3,4-tetrahydronaphthalen-1-yl)methyl)benzamide (2a):** Prepared by method A and purified by flash silica gel column chromatography (eluent: PE/EtOAc = 15/1 to 8/1 with 0.2% Et_3N, v/v) to afford **2a** as a white solid. mp: 116.2–118.5 ℃. 52.2 mg, >99% yield, 99.2% ee, *dr* = 50:1. $[\alpha]^{25}_{D}$ = +36.4 (c = 0.2, $CHCl_3$). **IR** (KBr): 3352, 3025, 2925, 1641, 1540, 1492, 1445, 1261, 1162, 1106, 801, 698 cm^{-1}. **^{1}H NMR** (400 MHz, $CDCl_3$) δ 7.68 – 7.59 (m, 2H), 7.50 (dd, *J* = 10.5, 4.2 Hz, 1H), 7.44 – 7.32 (m, 4H), 7.29 (t, *J* = 6.3 Hz, 3H), 7.24 – 7.15 (m, 3H), 7.14 – 7.01 (m, 5H), 6.93 (d, *J* = 7.6 Hz, 1H), 6.21 (t, *J* = 5.6 Hz, 1H), 4.52 (dd, *J* = 14.3, 7.2 Hz, 1H), 4.30 (d, *J* = 7.6 Hz, 1H), 4.10 (dd, *J* = 14.3, 5.1 Hz, 1H), 3.93 (td, *J* = 9.0, 3.8 Hz, 1H), 2.79 (dd, *J* = 14.4, 3.5 Hz, 1H), 2.58 (dd, *J* = 14.4, 9.1 Hz, 1H). **^{13}C NMR** (101 MHz, $CDCl_3$) δ 167.4, 145.9, 143.6, 139.0, 138.3, 135.5, 134.2, 132.5 (q, *J* = 307.3 Hz), 131.8, 129.4, 129.1, 128.9, 128.8, 128.8, 128.7, 128.0, 127.6, 127.4, 127.2, 127.2, 126.9, 126.4, 52.0, 48.2, 47.8, 46.0, 41.1. **^{19}F NMR** (377 MHz, $CDCl_3$) δ -39.74. **HR-ESI-MS** *m/z* calcd. for $C_{31}H_{25}ONF_3S$ $[M-H]^-$: 516.1614, found: 516.1613. **HPLC** (Daicel Chiralpak IA column, *i*-PrOH/hexane = 10/90, 1 mL/min, 222 nm) t_1 = 12.0 min (major), t_2 = 17.7 min (minor); t_3 = 11.1 min (diastereomer), t_4 = 19.3 min (diastereomer).

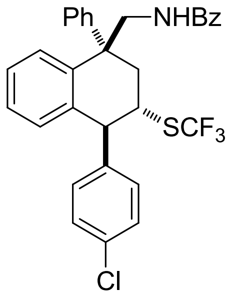

***N*-(((1*R*,3*S*,4*S*)-4-(4-Chlorophenyl)-1-phenyl-3-((trifluoromethyl)thio)-1,2,3,4-tetrahydronaphthalen-1-yl)methyl)benzamide (2b):** Prepared by method A at -60 ^oC, purified by flash silica gel column chromatography (eluent: PE/EtOAc = 15/1 to 8/1 with 0.2% Et_3N, v/v) to afford **2b** as a white solid. mp: 76.4–78.7 ℃. 56.3 mg, >99% yield, 98% ee. $[\alpha]^{26}_D$ = +46.2 (c = 0.2, $CHCl_3$). **IR** (KBr): 3340, 3028, 2925, 1641, 1541, 1490, 1448, 1261, 1154, 1107, 701 cm^{-1}. **^{1}H NMR** (400 MHz, $CDCl_3$) δ 7.62 (d, *J* = 7.6 Hz, 2H), 7.51 (t, *J* = 7.3 Hz, 1H), 7.46 – 7.32 (m, 4H), 7.32 – 7.26 (m, 3H), 7.24 – 7.15 (m, 2H), 7.07 (d, *J* = 8.2 Hz, 3H), 6.99 (d, *J* = 8.1 Hz, 2H), 6.88 (d, *J* = 7.6 Hz, 1H), 6.21 (s, 1H), 4.51 (dd, *J* = 14.3, 7.1 Hz, 1H), 4.26 (d, *J* = 7.9 Hz, 1H), 4.09 (dd, *J* = 14.3, 5.1 Hz, 1H), 3.88 (td, *J* = 9.0, 3.3 Hz, 1H), 2.76 (dd, *J* = 14.3, 2.6 Hz, 1H), 2.58 (dd, *J* = 14.3, 9.5 Hz, 1H). **^{13}C NMR** (101 MHz, $CDCl_3$) δ 167.4, 145.9, 142.1, 139.3, 137.9, 135.5 (q, *J* = 307.9 Hz), 134.2, 133.2, 132.4, 131.9 131.5, 130.5, 129.3, 129.1, 129.0, 128.9, 128.9, 127.9, 127.7, 127.4, 127.4, 126.9, 126.3, 51.5, 48.4, 47.7, 46.0, 41.7. **^{19}F NMR** (377 MHz, $CDCl_3$) δ -39.63. **HR-ESI-MS** *m/z* calcd. for $C_{31}H_{24}ONClF_3S$ $[M-H]^-$: 550.1225, found: 550.1226. **HPLC** (Daicel Chiralpak IA column, *i*-PrOH/hexane = 10/90, 1 mL/min, 222 nm) t_1 = 10.9 min (major), t_2 = 16.2 min (minor).

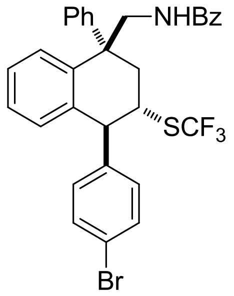

***N*-(((1*R*,3*S*,4*S*)-4-(4-Bromophenyl)-1-phenyl-3-((trifluoromethyl)thio)-1,2,3,4-tetrahydronaphthalen-1-yl)methyl)benzamide (2c):** Prepared by method A at -60 ^oC, purified by flash silica gel column chromatography (eluent: PE/EtOAc = 15/1 to 8/1 with 0.2% Et_3N, v/v) to afford **2c** as a white solid. mp: 121.4–122.8 ℃. 44.4 mg, 74% yield, 98% ee. $[\alpha]^{26}_D$ = +54.0 (c = 0.2, $CHCl_3$). **IR** (KBr): 3341, 3026, 2925, 1641, 1541, 1489, 1535, 1109, 701 cm^{-1}. **^{1}H NMR** (400 MHz, $CDCl_3$) δ 7.62 (d, *J* = 7.4 Hz, 2H), 7.52 (t, *J* = 7.3 Hz, 1H), 7.42 (t, *J* = 7.5 Hz, 2H), 7.39 – 7.33 (m, 2H), 7.33 – 7.24 (m, 4H), 7.24 – 7.21 (m, 2H), 7.20 – 7.15 (m, 1H), 7.07 (d, *J* = 7.4 Hz, 1H), 6.90 (dd, *J* = 17.4, 7.9 Hz, 3H), 6.21 (s, 1H), 4.51 (dd, *J* = 14.3, 7.1 Hz, 1H), 4.24 (d, *J* = 7.9 Hz, 1H), 4.09 (dd, *J* = 14.3, 5.1 Hz, 1H), 3.88 (td, *J* = 9.0, 3.5 Hz, 1H), 2.75 (dd, *J* = 14.3, 2.8 Hz, 1H), 2.57 (dd, *J* = 14.3, 9.5 Hz, 1H). **^{13}C NMR** (101 MHz, $CDCl_3$) δ 167.4, 145.8, 142.6, 139.3, 137.8, 135.4 (q, *J* = 307.4 Hz), 134.2, 132.4, 131.9, 131.9, 131.6, 130.8, 129.3, 129.1, 128.9, 128.9, 127.9, 127.7, 127.4, 127.4, 126.9, 126.3, 121.3, 51.5, 48.4, 47.7, 45.9, 41.6. **^{19}F NMR** (377 MHz,

$CDCl_3$) δ -39.65. **HR-ESI-MS** *m/z* calcd. for $C_{31}H_{24}ONBrF_3S$ [M-H]$^-$: 594.0720, found: 594.0722. **HPLC** (Daicel Chiralpak IA column, *i*-PrOH/hexane = 10/90, 1 mL/min, 240 nm) t_1 = 11.0 min (major), t_2 = 17.8 min (minor).

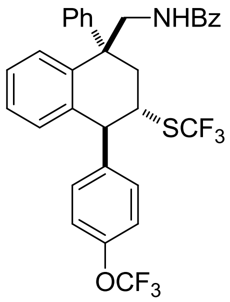

***N*-(((1*R*,3*S*,4*S*)-1-Phenyl-4-(4-(trifluoromethoxy)phenyl)-3-((trifluoromethyl)thio)-1,2,3,4-tetrahydronaphthalen-1-yl)methyl)benzamide (2d):** Prepared by method A at -60 ^{o}C, purified by flash silica gel column chromatography (eluent: PE/EtOAc = 15/1 to 8/1 with 0.2% Et_3N, v/v) to afford **2d** as a white solid. mp: 128.9–130.2 ℃. 59.4 mg, 99% yield, 98% ee. $[\alpha]^{26}_{D}$ = 33.7 (c = 0.2, $CHCl_3$). **IR** (KBr): 3360, 3026, 2924, 1640, 1538, 1444, 1259, 1155, 1109, 1021, 702 cm^{-1}. **^{1}H NMR** (400 MHz, $CDCl_3$) δ 7.66 – 7.59 (m, 2H), 7.51 (mk, 1H), 7.45 – 7.34 (m, 4H), 7.33 – 7.26 (m, 3H), 7.25 – 7.17 (m, 2H), 7.14 – 7.09 (m, 1H), 7.08 – 7.03 (m, 2H), 6.93 – 6.88 (m, 3H), 6.22 (t, *J* = 5.8 Hz, 1H), 4.55 (dd, *J* = 14.3, 7.4 Hz, 1H), 4.33 (d, *J* = 7.4 Hz, 1H), 4.06 (dd, *J* = 14.3, 5.1 Hz, 1H), 3.87 (td, *J* = 8.8, 3.8 Hz, 1H), 2.76 (dd, *J* = 14.4, 3.6 Hz, 1H), 2.57 (dd, *J* = 14.4, 9.0 Hz, 1H). **^{13}C NMR** (101 MHz, $CDCl_3$) δ 167.3, 148.3, 145.8, 142.3, 139.1, 137.8, 135.5 (q, *J* = 307.4 Hz), 134.2, 132.4, 131.9, 131.7, 130.5, 129.4, 129.1, 128.9, 128.9, 128.0, 127.8, 127.5, 127.4, 126.9, 126.3, 124.4 (q, *J* = 257.3 Hz), 121.8, 121.1, 119.3, 116.7, 51.5, 48.4, 47.7, 46.0, 41.0. **^{19}F NMR** (377 MHz, $CDCl_3$) δ -39.92, -57.79. **HR-ESI-MS** *m/z* calcd. for $C_{32}H_{24}O_2NF_6S$ [M-H]$^-$: 600.1437, found: 600.1435. **HPLC** (Daicel Chiralpak IA column, *i*-PrOH/hexane = 10/90, 1 mL/min, 222 nm) t_1 = 9.0 min (major), t_2 = 11.3 min (minor).

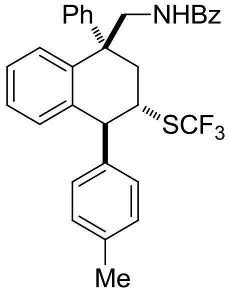

***N*-(((1*R*,3*S*,4*S*)-1-Phenyl-4-(p-tolyl)-3-((trifluoromethyl)thio)-1,2,3,4-tetrahydronaphthalen-1-yl)methyl)benzamide (2e):** Prepared by method A using 10 mol% catalyst in 4 mL DCM and 4 mL DCE at -60 ^{o}C, purified by flash silica gel column chromatography (eluent: PE/EtOAc = 15/1 to 8/1 with 0.2% Et_3N, v/v) to afford **2e** as a white solid. mp: 72.5–74.2 ℃. 49.8 mg, 94% yield, 99% ee, *dr* = 50:1. $[\alpha]^{26}_{D}$ = +46.1 (c = 0.2, $CHCl_3$). **IR** (KBr): 3329, 3023, 2925, 1653, 1514, 1447, 1261, 1103, 1024, 802, 703 cm^{-1}. **^{1}H NMR** (400 MHz, $CDCl_3$) δ 7.63 (d, *J* = 7.6 Hz, 2H), 7.50 (t, *J* = 7.2 Hz, 1H), 7.41 (t, *J* = 7.4 Hz, 2H), 7.38 – 7.32 (m, 2H), 7.32 – 7.26 (m, 3H), 7.23 – 7.14 (m, 2H), 7.09 (d, *J* = 7.6 Hz,

1H), 6.97 – 6.88 (m, 5H), 6.20 (s, 1H), 4.50 (dd, J = 14.2, 7.1 Hz, 1H), 4.26 (d, J = 7.6 Hz, 1H), 4.09 (dd, J = 14.2, 4.8 Hz, 1H), 3.93 (dd, J = 10.8, 5.4 Hz, 1H), 2.79 (d, J = 14.3 Hz, 1H), 2.57 (dd, J = 14.3, 9.2 Hz, 1H), 2.26 (s, 3H). ^{13}**C NMR** (101 MHz, $CDCl_3$) δ 167.4, 146.0, 140.5, 139.0, 138.6, 136.9, 135.6 (q, J = 307.6 Hz), 134.3, 132.5, 131.8, 129.5, 128.9, 128.8, 128.8, 128.0, 127.6, 127.3, 127.1, 126.9, 126.4, 51.6, 48.2, 47.8, 46.1, 41.3, 21.2. ^{19}**F NMR** (377 MHz, $CDCl_3$) δ -39.62. **HR-ESI-MS** *m/z* calcd. for $C_{32}H_{27}ONF_3S$ $[M-H]^-$: 530.1771, found: 530.1770. **HPLC** (Daicel Chiralpak OD column, *i*-PrOH/hexane = 6/94, 1 mL/min, 209 nm) t_1 = 11.0 min (minor), t_2 = 17.7 min (major); t_3 = 12.5 min (diastereomer), t_4 = 16.0 min (diastereomer).

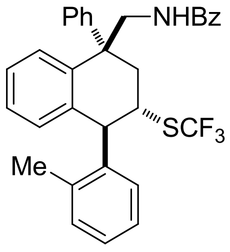

***N*-(((1*R*,3*S*,4*S*)-1-Phenyl-4-(o-tolyl)-3-((trifluoromethyl)thio)-1,2,3,4-tetrahydronaphthalen-1-yl)methyl)benzamide (2f):** Prepared by method A and purified by flash silica gel column chromatography (eluent: PE/EtOAc = 15/1 to 8/1 with 0.2% Et_3N, v/v) to afford **2f** as a white solid. mp: 57.0–58.8 ℃. 50.2 mg, 94% yield, 99.5% ee. $[\alpha]^{26}_D$ = +57.5 (c = 0.2, $CHCl_3$). **IR** (KBr): 3338, 3023, 2925, 1658, 1519, 1486, 1560, 1261, 1104, 1025, 801, 704 cm^{-1}. ^{1}**H NMR** (400 MHz, $CDCl_3$) δ 7.66 (d, J = 7.6 Hz, 2H), 7.49 (t, J = 7.2 Hz, 1H), 7.38 (dt, J = 14.0, 7.1 Hz, 6H), 7.30 (d, J = 6.8 Hz, 1H), 7.22 (d, J = 7.5 Hz, 1H), 7.19 – 7.05 (m, 3H), 6.96 (dd, J = 14.3, 7.3 Hz, 3H), 6.70 (d, J = 7.4 Hz, 1H), 6.26 (s, 1H), 4.49 – 4.36 (m, 2H), 4.31 (dd, J = 14.2, 5.7 Hz, 1H), 4.10 (t, J = 9.0 Hz, 1H), 2.83 (d, J = 14.1 Hz, 1H), 2.75 – 2.63 (m, 1H), 2.39 (s, 3H). ^{13}**C NMR** (101 MHz, $CDCl_3$) δ 167.4, 146.3, 141.6 – 141.0 (br), 140.0, 139.2, 137.0, 135.4 (q, J = 307.1 Hz), 134.4, 132.3, 131.8, 131.0 – 130.2 (br), 129.3, 129.2, 129.0, 128.7, 127.7, 127.6, 127.3, 127.2, 126.9, 126.8, 126.2, 48.8, 47.4, 46.5 – 45.5 (br), 44.6, 20.3. ^{19}**F NMR** (377 MHz, $CDCl_3$) δ -38.75. **HR-ESI-MS** *m/z* calcd. for $C_{32}H_{27}ONF_3S$ $[M-H]^-$: 530.1771, found: 530.1771. **HPLC** (Daicel Chiralpak IA column, *i*-PrOH/hexane = 10/90, 1 mL/min, 240 nm) t_1 = 8.4 min (major), t_2 = 10.8 min (minor).

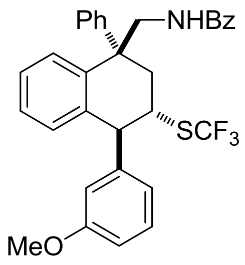

***N*-(((1*R*,3*S*,4*S*)-4-(3-Methoxyphenyl)-1-phenyl-3-((trifluoromethyl)thio)-1,2,3,4-tetrahydronaphthalen-1-yl)methyl)benzamide (2g):** Prepared by method A and purified by flash silica gel column chromatography (eluent: PE/EtOAc = 15/1 to 8/1 with 0.2% Et_3N, v/v) to afford **2g** as a white solid. mp: 71.0–72.2 ℃. 52.4 mg, 96% yield, 99% ee. $[\alpha]^{26}_D$ = +37.4 (c = 0.2, $CHCl_3$). **IR** (KBr): 3329, 3025, 2925, 1656, 1600, 1519, 1487, 1262, 1105, 1043, 703 cm^{-1}. **^{1}H NMR** (400 MHz, $CDCl_3$) δ 7.63 (d, *J* = 7.6 Hz, 2H), 7.48 (t, *J* = 7.3 Hz, 1H), 7.43 – 7.32 (m, 4H), 7.31 – 7.26 (m, 3H), 7.23 – 7.14 (m, 2H), 7.06 (d, *J* = 7.4 Hz, 1H), 7.03 – 6.92 (m, 2H), 6.81 – 6.69 (m, 2H), 6.59 (d, *J* = 7.5 Hz, 1H), 6.22 (s, 1H), 4.48 (dd, *J* = 14.2, 6.8 Hz, 1H), 4.25 (d, *J* = 8.0 Hz, 1H), 4.12 (dt, *J* = 18.0, 9.0 Hz, 1H), 3.98 (dd, *J* = 11.5, 5.5 Hz, 1H), 3.72 (s, 3H), 2.87 – 2.72 (m, 1H), 2.59 (dd, *J* = 14.2, 9.6 Hz, 1H). **^{13}C NMR** (101 MHz, $CDCl_3$) δ 167.5, 160.0, 146.0, 145.0, 139.2, 138.3, 135.6 (q, *J* = 307.3 Hz), 134.3, 132.5, 131.7, 131.6, 129.8, 129.5, 128.9, 128.9, 128.8, 128.0, 127.6, 127.3, 127.2, 127.0, 126.4, 121.4, 115.4, 112.3, 55.3, 52.0, 48.3, 47.7, 46.0, 41.9. **^{19}F NMR** (377 MHz, $CDCl_3$) δ -39.53. **HR-ESI-MS** *m/z* calcd. for $C_{32}H_{27}O_2NF_3S$ $[M-H]^-$: 546.1720, found: 546.1719. **HPLC** (Daicel Chiralpak OD column, *i*-PrOH/hexane = 10/90, 1 mL/min, 206 nm) t_1 = 8.8 min (minor), t_2 = 12.6 min (major).

Note: The standard sample is made by the mixture of equal amount of enantiomers of **C7**.

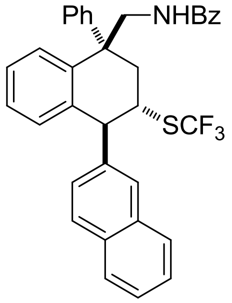

***N*-(((1*S*,2*S*,4*R*)-4-Phenyl-2-((trifluoromethyl)thio)-1,2,3,4-tetrahydro-[1,2'-binaphthalen]-4-yl)methyl)benzamide (2h):** Prepared by method A using 10 mol% catalyst in 4 mL DCM and 4 mL DCE at -60 ^{o}C, purified by flash silica gel column chromatography (eluent: PE/EtOAc = 20/1 to 10/1 with 0.2% Et_3N, v/v) to afford **2h** as a white solid. mp: 102.8–104.1 ℃. 55.1 mg, 97% yield, 99% ee, dr = 50:1. $[\alpha]^{26}_D$ = +92.2 (c = 0.2, $CHCl_3$). **IR** (KBr): 3351, 3010, 2925, 1641, 1539, 1450, 1261, 1105, 1026, 800 cm^{-1}. **^{1}H NMR** (400 MHz, $CDCl_3$) δ 7.77 (d, *J* = 7.9 Hz, 1H), 7.65 (d, *J* = 7.3 Hz, 3H), 7.57 – 7.48 (m, 3H), 7.49 – 7.26 (m, 9H), 7.19 (d, *J* = 8.0 Hz, 2H), 7.16 – 7.05 (m, 2H), 6.90 (d, *J* = 7.8 Hz, 1H), 6.28 (s, 1H), 4.52 (dd, *J* = 14.2, 6.7 Hz, 1H), 4.42 (d, *J* = 8.5 Hz, 1H), 4.21 (dd, *J* = 14.3, 5.1 Hz, 1H), 4.09 (t, *J* = 7.7 Hz, 1H), 2.86 (d, *J* = 14.1 Hz, 1H), 2.65 (dd, *J* =

14.0, 10.2 Hz, 1H). **^{13}C NMR** (101 MHz, $CDCl_3$) δ 167.4, 146.0, 140.6, 139.5, 138.3, 135.5 (q, *J* = 307.2 Hz), 134.4, 133.3, 132.7, 132.4, 131.8, 131.6, 129.4, 129.1, 129.0, 128.9, 128.9, 128.7, 127.9, 127.9, 127.8, 127.6, 127.3, 127.2, 127.0, 126.4, 126.3, 126.1, 52.1, 48.4, 47.7, 45.8, 42.4. **^{19}F NMR** (377 MHz, $CDCl_3$) δ -39.27. **HR-ESI-MS** *m/z* calcd. for $C_{35}H_{27}ONF_3S$ $[M-H]^-$: 566.1771, found: 566.1776. **HPLC** (Daicel Chiralpak IA column, *i*-PrOH/hexane = 10/90, 1 mL/min, 245 nm) t_1 = 13.2 min (major), t_2 = 19.5 min (minor); t_3 = 11.7 min (diastereomer), t_4 = 20.9 min (diastereomer).

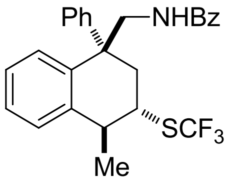

***N*-(((1*R*,3*S*,4*S*)-4-Methyl-1-phenyl-3-((trifluoromethyl)thio)-1,2,3,4-tetrahydronaphthalen-1-yl)methyl)benzamide (2i):** Prepared by method A and purified by flash silica gel column chromatography (eluent: PE/EtOAc = 15/1 to 10/1 with 0.2% Et_3N, v/v) to afford **2i** as a white solid. mp: 96.6–98.6 ℃. 32.4 mg, 71% yield, 97% ee. $[\alpha]^{26}_D$ = -18.7 (c = 0.2, $CHCl_3$). **IR** (KBr): 3330, 3027, 2925, 1658, 1520, 1486, 1459, 1280, 1110, 704 cm^{-1}. **^{1}H NMR** (400 MHz, $CDCl_3$) δ 7.56 (d, *J* = 7.6 Hz, 2H), 7.46 (t, *J* = 7.2 Hz, 1H), 7.40 – 7.29 (m, 6H), 7.27 – 7.16 (m, 4H), 7.00 (d, *J* = 7.8 Hz, 1H), 6.02 (s, 1H), 4.34 (dd, *J* = 14.1, 6.6 Hz, 1H), 3.97 (dd, *J* = 14.1, 4.8 Hz, 1H), 3.55 (dd, *J* = 11.5, 7.8 Hz, 1H), 3.06 (p, *J* = 6.6 Hz, 1H), 2.73 (dd, *J* = 14.3, 3.3 Hz, 1H), 2.57 (dd, *J* = 14.3, 9.1 Hz, 1H), 1.51 (d, *J* = 6.8 Hz, 3H). **^{13}C NMR** (101 MHz, $CDCl_3$) δ 167.5, 145.8, 140.4, 138.5, 135.7 (q, *J* = 307.0 Hz), 134.3, 132.6, 131.7, 129.6, 129.4, 128.9, 128.8, 128.8, 127.9, 127.7, 127.2, 126.8, 126.8, 126.5, 48.0, 47.5, 45.3, 41.4, 39.2, 22.3. **^{19}F NMR** (377 MHz, $CDCl_3$) δ -40.11. **HR-ESI-MS** *m/z* calcd. for $C_{26}H_{23}ONF_3S$ $[M-H]^-$: 454.1458, found: 454.1455. **HPLC** (Daicel Chiralpak ID column, *i*-PrOH/hexane = 8/92, 1 mL/min, 222 nm) t_1 = 12.5 min (major), t_2 = 14.4 min (minor).

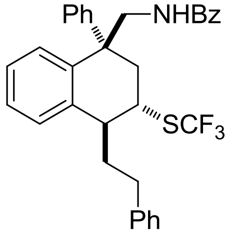

***N*-(((1*R*,3*S*,4*S*)-4-Phenethyl-1-phenyl-3-((trifluoromethyl)thio)-1,2,3,4-tetrahydronaphthalen-1-yl)methyl)benzamide (2j):** Prepared by method A and purified by flash silica gel column chromatography (eluent: PE/EtOAc = 15/1 to 10/1 with 0.2% Et_3N, v/v) to afford **2j** as a white solid. mp: 124.7–127.0 ℃. 43.8 mg, 80% yield, 97% ee, dr = 50:1. $[\alpha]^{25}_D$ = +5.0 (c = 0.2, $CHCl_3$). **IR** (KBr): 3445, 3031, 2926, 1659, 1521, 1443, 1273, 1115, 703 cm^{-1}. **^{1}H NMR** (400 MHz, $CDCl_3$) δ

7.51 (d, J = 7.3 Hz, 2H), 7.42 (t, J = 7.4 Hz, 1H), 7.38 – 7.07 (m, 14H), 7.00 (d, J = 6.9 Hz, 2H), 5.96 (d, J = 2.7 Hz, 1H), 4.55 (dd, J = 14.0, 8.0 Hz, 1H), 3.83 (ddd, J = 18.2, 12.1, 4.5 Hz, 2H), 3.19 (dd, J = 11.4, 4.8 Hz, 1H), 2.87 (dd, J = 14.7, 3.4 Hz, 1H), 2.76 – 2.48 (m, 3H), 2.20 – 1.94 (m, 2H). **^{13}C NMR** (101 MHz, $CDCl_3$) δ 167.6, 146.3, 141.4, 139.6, 137.7, 135.7 (q, J = 307.0 Hz), 134.2, 132.7, 131.7, 130.3, 129.6, 129.2, 128.8, 128.6, 128.5, 128.3, 128.2, 127.8, 127.3, 127.1, 126.8, 126.6, 126.0, 48.2, 47.7, 45.2, 41.9, 39.1, 38.4, 32.7. **^{19}F NMR** (377 MHz, $CDCl_3$) δ -40.51. **HR-ESI-MS** *m/z* calcd. for $C_{33}H_{29}ONF_3S$ $[M-H]^-$: 544.1927, found: 544.1923. **HPLC** (Daicel Chiralpak IA column, *i*-PrOH/hexane = 10/90, 1 mL/min, 222 nm) t_1 = 11.4 min (major), t_2 = 16.5 min (minor); t_3 = 12.6 min (diastereomer), t_4 = 20.6 min (diastereomer).

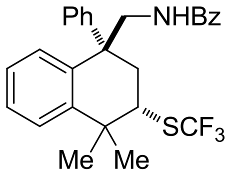

***N*-(((1*R*,3*S*)-4,4-Dimethyl-1-phenyl-3-((trifluoromethyl)thio)-1,2,3,4-tetrahydronaphthalen-1-yl)methyl)benzamide (2k):** Prepared by method A and purified by flash silica gel column chromatography (eluent: PE/EtOAc = 15/1 to 10/1 with 0.2% Et_3N, v/v) to afford **2k** as a white solid. mp: 99.8–101.2 ℃. 46.8 mg, 99% yield, 92% ee. $[\alpha]^{25}_D$ = -47.9 (c = 0.2, $CHCl_3$). **IR** (KBr): 3331, 3030, 2929, 1636, 1546, 1490, 1452, 1293, 1112, 801, 697 cm^{-1}. **^{1}H NMR** (400 MHz, $CDCl_3$) δ 7.54 (d, J = 7.8 Hz, 1H), 7.48 – 7.37 (m, 4H), 7.37 – 7.20 (m, 7H), 7.07 (d, J = 7.4 Hz, 2H), 5.81 (d, J = 8.6 Hz, 1H), 4.77 (dd, J = 13.8, 9.6 Hz, 1H), 3.71 (dd, J = 13.8, 2.2 Hz, 1H), 3.28 – 3.07 (m, 1H), 2.70 (t, J = 13.6 Hz, 1H), 2.55 (dd, J = 13.6, 2.2 Hz, 1H), 1.50 (s, 3H), 1.20 (s, 3H). **^{13}C NMR** (101 MHz, $CDCl_3$) δ 167.7, 147.5, 145.7, 135.9, 135.6 (q, J = 306.2 Hz), 134.4, 132.6, 131.7, 129.5, 128.7, 128.6, 128.0, 127.6, 127.5, 127.2, 127.1, 126.7, 126.5, 50.2, 49.4, 47.9, 40.7, 38.8, 28.7, 27.0. **^{19}F NMR** (377 MHz, $CDCl_3$) δ -40.64. **HR-ESI-MS** *m/z* calcd. for $C_{27}H_{25}ONF_3S$ $[M-H]^-$: 468.1614, found: 468.1612. **HPLC** (Daicel Chiralpak IA column, *i*-PrOH/hexane = 10/90, 1 mL/min, 222 nm) t_1 = 14.2 min (major), t_2 = 17.2 min (minor).

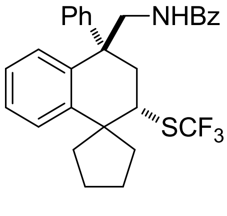

***N*-(((2'*S*,4'*R*)-4'-Phenyl-2'-((trifluoromethyl)thio)-3',4'-dihydro-2'H-spiro[cyclopentane-1,1'-naphthalen]-4'-yl)methyl)benzamide (2l):** Prepared by method A and purified by flash silica gel column chromatography (eluent: PE/EtOAc = 15/1 to 10/1 with 0.2% Et_3N, v/v) to afford **2l** as a white solid. mp: 98.5–100.1 ℃. 33.8 mg, 68% yield,

97% ee. $[\alpha]^{26}_{D}$ = -44.5 (c = 0.2, $CHCl_3$). **IR** (KBr): 3343, 3028, 2925, 1639, 1543, 1489, 1451, 1261, 1109, 1021, 800, 699 cm^{-1}. **^{1}H NMR** (400 MHz, $CDCl_3$) δ 7.49 – 7.27 (m, 10H), 7.25 – 7.18 (m, 2H), 7.10 – 7.05 (m, 2H), 5.79 (d, *J* = 8.0 Hz, 1H), 4.75 (dd, *J* = 13.8, 9.4 Hz, 1H), 3.70 (dd, *J* = 13.8, 2.7 Hz, 1H), 3.29 (dd, *J* = 12.3, 3.0 Hz, 1H), 2.80 – 2.56 (m, 2H), 2.34 – 2.09 (m, 2H), 2.09 – 1.92 (m, 2H), 1.92 – 1.71 (m, 3H), 1.70 – 1.60 (m, 1H). **^{13}C NMR** (101 MHz, $CDCl_3$) δ 167.6, 150.7, 146.2, 135.6, 135.4, 134.5, 132.6 (q, *J* = 306.3 Hz), 131.6, 129.5, 128.7, 128.6, 128.5, 128.2, 127.6, 127.5, 127.1, 126.9, 126.7, 126.5, 77.2, 50.6, 50.3, 50.0, 48.2, 43.0, 41.1, 41.0, 28.5, 27.5. **^{19}F NMR** (377 MHz, $CDCl_3$) δ -40.37. **HR-ESI-MS** *m/z* calcd. for $C_{29}H_{27}ONF_3S$ $[M-H]^-$: 494.1771, found: 494.1769. **HPLC** (Daicel Chiralpak IA column, *i*-PrOH/hexane = 10/90, 1 mL/min, 222 nm) t_1 = 11.1 min (major), t_2 = 15.6 min (minor).

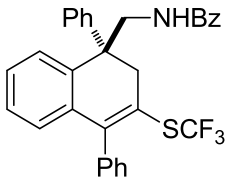

(*R*)-*N*-((1,4-Diphenyl-3-((trifluoromethyl)thio)-1,2-dihydronaphthalen-1-yl)methyl)benzamide (2m): Prepared by method A using 2 equivalents of TMSOTf and purified by flash silica gel column chromatography (eluent: PE/EtOAc = 15/1 to 8/1 with 0.2% Et_3N, v/v) to afford **2m** as a white solid. mp: 57.6–61.0 ℃. 34.9 mg, 68% yield, 94% ee. $[\alpha]^{26}_{D}$ = -136.0 (c = 0.2, $CHCl_3$). **IR** (KBr): 3321, 3026, 2925, 1650, 1600, 1521, 1486, 1445, 1263, 1102, 1027, 799, 699 cm^{-1}. **^{1}H NMR** (400 MHz, $CDCl_3$) δ 7.60 (d, *J* = 7.6 Hz, 2H), 7.50 – 7.26 (m, 13H), 7.24 – 7.13 (m, 2H), 7.05 – 6.76 (m, 2H), 6.07 (s, 1H), 4.34 (dd, *J* = 14.1, 6.2 Hz, 1H), 4.20 (dd, *J* = 14.1, 5.3 Hz, 1H), 3.45 (d, *J* = 17.1 Hz, 1H), 3.17 (d, *J* = 17.0 Hz, 1H). **^{13}C NMR** (101 MHz, $CDCl_3$) δ 167.6, 147.8, 143.3, 138.7, 137.9, 136.3, 134.7, 134.3 (q, *J* = 310.2 Hz), 131.7, 131.2, 129.9 – 129.4 (br), 129.1, 128.9, 128.8, 128.5, 128.1, 128.0, 127.7, 127.4, 126.9, 126.8, 125.1, 121.4, 48.5, 45.1, 42.5. **^{19}F NMR** (377 MHz, $CDCl_3$) δ -38.70. **HR-ESI-MS** *m/z* calcd. for $C_{31}H_{23}ONF_3S$ $[M-H]^-$: 514.1458, found: 514.1460. **HPLC** (Daicel Chiralpak IA column, *i*-PrOH/hexane = 10/90, 1 mL/min, 290 nm) t_1 = 8.4 min (major), t_2 = 12.5 min (minor).

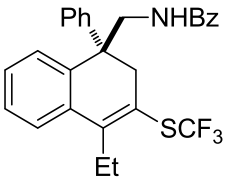

(*R*)-*N*-((4-Ethyl-1-phenyl-3-((trifluoromethyl)thio)-1,2-dihydronaphthalen-1-yl)methyl)benzamide (2n): Prepared by method A using 2 equivalents of TMSOTf and purified by flash silica gel column chromatography (eluent: PE/EtOAc = 15/1 to 8/1 with

0.2% Et_3N, v/v) to afford **2n** as a white solid. mp: 39.8–41.8 ℃. 34.1 mg, 73% yield, 87% ee. $[\alpha]^{26}_{D}$ = -114.8 (c = 0.2, $CHCl_3$). **IR** (KBr): 3332, 3029, 2925, 1647, 1603, 1527, 1447, 1283, 1104, 699 cm^{-1}. **^{1}H NMR** (400 MHz, $CDCl_3$) δ 7.56 (t, *J* = 9.4 Hz, 3H), 7.45 (t, *J* = 7.3 Hz, 1H), 7.42 – 7.27 (m, 6H), 7.27 – 7.20 (m, 4H), 5.98 (s, 1H), 4.13 (d, *J* = 5.8 Hz, 2H), 3.29 (d, *J* = 17.0 Hz, 1H), 3.08 – 2.78 (m, 3H), 0.97 (t, *J* = 7.5 Hz, 3H). **^{13}C NMR** (101 MHz, $CDCl_3$) δ 167.5, 149.6, 143.2, 139.9, 134.6, 134.5 (q, *J* = 310.1 Hz), 134.4, 131.6, 131.4, 129.9 – 129.4 (br), 129.0, 128.7, 128.6, 128.3, 127.9, 127.7, 127.2, 127.1, 126.9, 126., 125.2, 118.2, 48.1, 44.9, 43.3, 23.8, 13.6. **^{19}F NMR** (377 MHz, $CDCl_3$) δ -39.67. **HR-ESI-MS** *m/z* calcd. for $C_{27}H_{23}ONF_3S$ $[M-H]^-$: 466.1458, found: 466.1457. **HPLC** (Daicel Chiralpak IA column, *i*-PrOH/hexane = 10/90, 1 mL/min, 280 nm) t_1 = 8.7 min (major), t_2 = 12.9 min (minor).

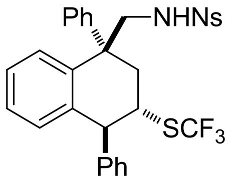

***N*-(((1*R*,3*S*,4*S*)-1,4-Diphenyl-3-((trifluoromethyl)thio)-1,2,3,4-tetrahydronaphthalen-1-yl)methyl)-4-nitrobenzenesulfonamide (2o):** Prepared by method A using DCM (4 mL) + DCE (4mL) as ths solvents, purified by flash silica gel column chromatography (eluent: PE/EtOAc = 15/1 to 8/1 with 0.2% Et_3N, v/v) to afford **2o** as a white solid. mp: 165.2–167.4 ℃. 54.5 mg, 91% yield and 94% ee. $[\alpha]^{25}_{D}$ = +33.4 (c = 0.2, $CHCl_3$). **IR** (KBr): 3281, 3257, 2955, 2924, 2855, 1603, 1531, 1451, 1345, 1261, 1096, 1023, 803 cm^{-1}. **^{1}H NMR** (400 MHz, $CDCl_3$) δ 8.28 (d, *J* = 8.3 Hz, 2H), 7.95 (d, *J* = 8.1 Hz, 2H), 7.41 – 7.22 (m, 6H), 7.18 (dd, *J* = 11.3, 7.5 Hz, 2H), 7.09 (t, *J* = 5.6 Hz, 4H), 6.94 (d, *J* = 4.2 Hz, 2H), 4.68 (s, 1H), 4.29 (d, *J* = 7.2 Hz, 1H), 3.95 – 3.85 (m, 1H), 3.78 (d, *J* = 12.7 Hz, 1H), 3.62 (d, *J* = 12.8 Hz, 1H), 2.90 – 2.79 (m, 1H), 2.46 (dd, *J* = 14.3, 8.7 Hz, 1H). **^{13}C NMR** (101 MHz, $CDCl_3$) δ 150.2, 145.4, 145.0, 143.3, 138.8, 137.3, 135.5 (q, *J* = 307.5 Hz), 132.4, 132.0, 129.4, 129.1, 129.0 128.9, 128.6, 128.4, 127.9, 127. 7, 127.6, 127.4, 126.3, 124.6, 52.3, 51.9, 47.2, 45.7, 41.0. **^{19}F NMR** (377 MHz, $CDCl_3$) δ -39.69. **HR-ESI-MS** *m/z* calcd. for $C_{30}H_{24}O_4N_2F_3S_2$ $[M-H]^-$: 597.1135, found: 597.1131. **HPLC** (Daicel Chiralpak IA column, *i*-PrOH/hexane = 20/80, 1 mL/min, 262 nm) t_1 = 7.3 min (major), t_2 = 20.6 min (minor).

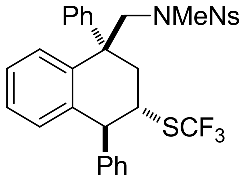

***N*-(((1*R*,3*S*,4*S*)-1,4-Diphenyl-3-((trifluoromethyl)thio)-1,2,3,4-tetrahydronaphthalen-1-yl)methyl)-*N*-methyl-4-nitrobenzene sulfonamide (2o'):** Prepared by method A and purified by flash silica gel column chromatography (eluent: PE/EtOAc = 20/1 to

10/1 with 0.2% Et_3N, v/v) to afford **2o'** as a white solid. mp: 167.4–168.5 ℃. 29.4 mg, 55% yield and 85% ee, *dr* = 8:1. $[\alpha]^{26}_{D}$ = +31.7 (c = 0.2, $CHCl_3$). **IR** (KBr): 3058, 2920, 2852, 1603, 1529, 1352, 1116, 760 cm^{-1}. **^{1}H NMR** (400 MHz, $CDCl_3$) δ8.44 – 8.35 (m, 2H), 8.02 – 7.97 (m, 2H), 7.40 (dd, *J* = 10.0, 4.6 Hz, 2H), 7.36 – 7.26 (m, 4H), 7.24 – 7.13 (m, 6H), 7.10 – 7.05 (m, 1H), 6.94 – 6.89 (m, 1H), 4.56 (d, *J* = 13.9 Hz, 1H), 4.32 – 4.21 (m, 2H), 3.35 (d, *J* = 13.9 Hz, 1H), 3.27 – 3.19 (m, 1H), 2.60 – 2.47 (m, 1H), 2.32 (s, 3H). **^{13}C NMR** (101 MHz, $CDCl_3$) δ 150.35, 146.89, 143.27, 143.06, 139.64, 137.79, 135.54 (q, J = 307.4 Hz), 132.48, 131.63, 130.16, 129.43, 129.34, 128.95, 128.93, 128.67, 127.77, 127.69, 127.46, 127.33, 126.65, 126.37, 124.63, 77.16, 61.34, 52.04, 47.71, 46.13, 42.54, 37.89. **^{19}F NMR** (377 MHz, $CDCl_3$) δ -38.80. **HR-ESI-MS** *m/z* calcd. for $C_{31}H_{28}O_4N_2F_3S_2$ $[M+H]^+$: 613.1437, found: 613.1431. **HPLC** (Daicel Chiralpak IA column, *i*-PrOH/hexane = 10/90, 1 mL/min, 207 nm) t_1 = 10.3 min (minor), t_2 = 18.2 min (major).

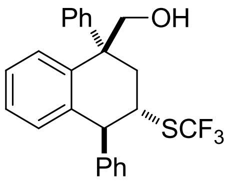

((1*R*,3*S*,4*S*)-1,4-Diphenyl-3-((trifluoromethyl)thio)-1,2,3,4-tetrahydronaphthalen-1-yl)methanol (2p): Prepared by method A using 1 equivalent of TIPSOTf (27 μL, 0.1 mmol) as acid and purified by flash silica gel column chromatography (eluent: PE/EtOAc = 20/1 to 10/1 with 0.2% Et_3N, v/v) to afford **2p** as a white solid. mp: 37.6–39.0 ℃. 34.1 mg, 82% yield, 81% ee. $[\alpha]^{25}_{D}$ = +88.8 (c = 0.2, $CHCl_3$). **IR** (KBr): 3444, 3027, 2925, 1599, 1492, 1448, 1261, 1112, 756, 701 cm^{-1}. **^{1}H NMR** (400 MHz, $CDCl_3$) δ 7.38 – 7.21 (m, 8H), 7.20 – 7.07 (m, 4H), 6.98 (dd, *J* = 7.6, 1.6 Hz, 1H), 6.86 – 6.81 (m, 1H), 4.34 (d, *J* = 11.3 Hz, 1H), 4.27 (d, *J* = 11.3 Hz, 1H), 4.20 (d, *J* = 9.4 Hz, 1H), 4.16 – 4.08 (m, 1H), 2.93 (dd, *J* = 14.1, 3.4 Hz, 1H), 2.47 (dd, *J* = 14.1, 10.5 Hz, 1H), 1.66 (br, 1H). **^{13}C NMR** (101 MHz, $CDCl_3$) δ 146.9, 143.6, 139.6, 139.1, 135.6 (q, *J* = 307.1 Hz), 132.6, 131.0, 129.5, 129.4, 129.3, 128.8, 128.6, 128.0, 127.4, 127.2, 127.0, 126.9, 126.5, 70.4, 52.0, 49.5, 46.5, 43.4. **^{19}F NMR** (377 MHz, $CDCl_3$) δ -39.23. **HR-ESI-MS** *m/z* calcd. for $C_{25}H_{22}O_3F_3S$ $[M+HCOO]^-$: 459.1247, found: 459.1244. **HPLC** (Daicel Chiralpak IA column, *i*-PrOH/hexane = 5/95, 1 mL/min, 207 nm) t_1 = 8.2 min (minor), t_2 = 12.0 min (major).

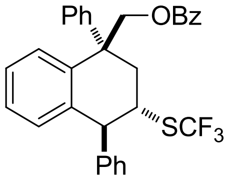

((1*R*,3*S*,4*S*)-1,4-Diphenyl-3-((trifluoromethyl)thio)-1,2,3,4-tetrahydronaphthalen-1-yl)methyl benzoate (2q): Prepared by method A at -60 ^{o}C and purified by flash silica gel column chromatography (eluent: PE/EtOAc = 200/1 to 100/1, v/v) to afford **2q** as a white solid. mp: 48.1–49.7 ℃. 41.0 mg, 79% yield, 94% ee, *dr* = 50:1. $[\alpha]^{25}_{D}$ = +83.6 (c = 0.2, $CHCl_3$). **IR** (KBr): 3028, 2925, 1601, 1450, 1265, 1113, 709 cm^{-1}. **^{1}H NMR** (400 MHz, $CDCl_3$) δ 8.00 (d, *J* = 7.3 Hz, 2H), 7.60 – 7.52 (m, 1H), 7.42 (t, *J* = 7.7 Hz, 2H), 7.39 – 7.25 (m, 8H), 7.17 (dd, *J* = 12.4, 6.8 Hz, 2H), 7.12 – 7.04 (m, 2H), 6.91 (dd, *J* = 5.6, 3.6 Hz, 1H), 6.80 (dd, *J* = 4.9, 3.9 Hz, 1H), 5.05 – 4.94 (m, 2H), 4.21 (d, *J* = 10.2 Hz, 1H), 3.96 (td, *J* = 11.3, 3.1 Hz, 1H), 3.07 (dd, *J* = 14.1, 2.8 Hz, 1H), 2.60 (dd, *J* = 13.8, 11.8 Hz, 1H). **^{13}C NMR** (101 MHz, $CDCl_3$) δ 166.4, 146.6, 143.2, 139.3, 138.6, 135.3 (q, *J* = 307.0 Hz), 133.4, 132.3, 130.8, 129.8, 129.5, 129.2, 128.9, 128.7, 128.6, 128.1, 127.5, 127.4, 127.0, 126.9, 126.2, 69.0, 51.9, 48.2, 46.3. **^{19}F NMR** (377 MHz, $CDCl_3$) δ -39.07. **HR-EI-MS** *m/z* calcd. for $C_{31}H_{25}O_2F_3S$ $[M]^+$: 518.1522, found: 518.1525. **HPLC** (Daicel Chiralpak IA column, *i*-PrOH/hexane = 3/97, 0.5 mL/min, 222 nm) t_1 = 8.4 min (minor), t_2 = 12.0 min (major); t_3 = 9.7 min (diastereomer), t_4 = 10.9 min (diastereomer).

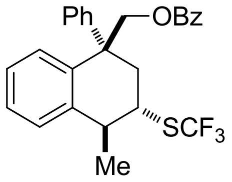

((1*R*,3*S*,4*S*)-4-Methyl-1-phenyl-3-((trifluoromethyl)thio)-1,2,3,4-tetrahydronaphthalen-1-yl)methyl benzoate (2r): Prepared by method A and purified by flash silica gel column chromatography (eluent: PE/EtOAc = 200/1 to 100/1, v/v) to afford **2r** as a white solid. mp: 81.8–84.8 ℃. 43.2 mg, 95% yield, 97% ee. $[\alpha]^{25}_{D}$ = +32.3 (c = 0.2, $CHCl_3$). **IR** (KBr): 3027, 2924, 1602, 1488, 1450 ,1262, 1112, 1024, 710 cm^{-1}. **^{1}H NMR** (400 MHz, $CDCl_3$) δ 8.02 (d, *J* = 7.3 Hz, 2H), 7.66 – 7.54 (m, 1H), 7.37 (dtd, *J* = 27.7, 15.3, 6.9 Hz, 9H), 7.12 (t, *J* = 7.5 Hz, 1H), 6.85 (d, *J* = 7.4 Hz, 1H), 4.85 (q, *J* = 12.1 Hz, 2H), 3.52 (td, *J* = 11.7, 3.8 Hz, 1H), 3.11 – 2.91 (m, 2H), 2.67 – 2.47 (m, 1H), 1.66 (d, *J* = 6.8 Hz, 3H). **^{13}C NMR** (101 MHz, $CDCl_3$) δ 166.4, 146.3, 139.7, 139.2, 135.6 (q, *J* = 306.6 Hz), 133.3, 132.5, 129.9, 129.8, 129.5, 128.6, 128.2, 128.0, 127.5, 126.9, 126.4, 68.5, 48.2, 46.0, 43.0, 38.4, 21.0. **^{19}F NMR** (377 MHz, $CDCl_3$) δ -39.42. **HR-ESI-MS** *m/z* calcd. for $C_{26}H_{23}O_2F_3NaS$ $[M+Na]^+$: 479.1263, found: 479.1264. **HPLC** (Daicel Chiralpak IA column, *i*-PrOH/hexane = 1/99, 0.5 mL/min, 222 nm) t_1 = 14.9 min (minor), t_2 = 18.0 min (major).

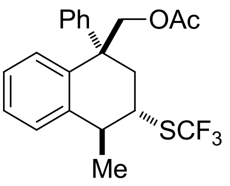

((1*R*,3*S*,4*S*)-4-Methyl-1-phenyl-3-((trifluoromethyl)thio)-1,2,3,4-tetrahydronaphthalen-1-yl)methyl acetate (2s): Prepared by method A and purified by flash silica gel column chromatography (eluent: PE/EtOAc = 200/1 to 100/1, v/v) to afford **2s** as a colourless oil. 30.9 mg, 78% yield, 97% ee. $[\alpha]^{25}_{D}$ = +39.3 (c = 0.2, $CHCl_3$). **IR** (KBr): 3027, 2935, 1601, 1491, 1445, 1381, 1229, 1111, 1035, 702 cm^{-1}. **^{1}H NMR** (400 MHz, $CDCl_3$) δ 7.38 – 7.27 (m, 3H), 7.27 – 7.15 (m, 4H), 7.05 (t, *J* = 7.5 Hz, 1H), 6.72 (d, *J* = 7.9 Hz, 1H), 4.66 – 4.49 (m, 2H), 3.52 – 3.33 (m, 1H), 3.06 – 2.90 (m, 1H), 2.80 (dd, *J* = 13.9, 3.1 Hz, 1H), 2.41 (t, *J* = 13.0 Hz, 1H), 2.06 (s, 3H), 1.61 (d, *J* = 6.8 Hz, 3H). **^{13}C NMR** (101 MHz, $CDCl_3$) δ 170.8, 146.4, 139.8, 139.3, 135.8 (q, *J* = 306.6 Hz), 132.7, 129.8, 129.7, 128.5, 128.2, 127.9, 127.5, 126.8, 126.6, 126.4, 68.3, 47.9, 46.0, 43.2, 38.3, 20.9, 20.8. **^{19}F NMR** (377 MHz, $CDCl_3$) δ -39.56. **HR-ESI-MS** *m/z* calcd. for $C_{21}H_{21}O_2F_3NaS$ $[M+Na]^+$: 417.1107, found: 417.1109. **HPLC** (Daicel Chiralpak IC column, *i*-PrOH/hexane = 3/97, 0.5 mL/min, 222 nm) t_1 = 8.1 min (minor), t_2 = 8.7 min (major).

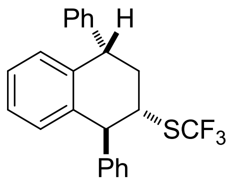

((1*S*,2*S*,4*S*)-1,4-Diphenyl-1,2,3,4-tetrahydronaphthalen-2-yl)(trifluoromethyl)sulfane (2t): Prepared by method A using 1 equivalent TIPSOTf (27 μL, 0.1 mmol) as acid and purified by flash silica gel column chromatography (eluent: PE/EtOAc = PE to 200/1 to 100/1, v/v) to afford **2t** as a white solid. mp 83.2–84.7 ℃. 31.2 mg, 81% yield, 86% ee. $[\alpha]^{26}_{D}$ = +90.7 (c = 0.2, $CHCl_3$). **IR** (KBr): 3029, 2925, 1599, 1492, 1452, 1261, 1148, 111, 1027, 801, 700 cm^{-1}. **^{1}H NMR** (400 MHz, $CDCl_3$) δ 7.31 (ddt, *J* = 20.2, 13.4, 6.7 Hz, 6H), 7.20 (dd, *J* = 14.2, 7.6 Hz, 4H), 7.06 – 6.96 (m, 2H), 6.84 – 6.69 (m, 2H), 4.34 (dd, *J* = 11.8, 4.7 Hz, 1H), 4.17 (d, *J* = 11.2 Hz, 1H), 3.89 – 3.71 (m, 1H), 2.79 (d, *J* = 13.3 Hz, 1H), 2.37 (q, *J* = 12.4 Hz, 1H). **^{13}C NMR** (101 MHz, $CDCl_3$) δ 145.3, 143.5, 139.1, 138.8, 135.5 (q, *J* = 306.9 Hz), 132. 4, 130.3, 129.5, 129.4, 129.2, 129.0, 128.9, 128.8, 127.4, 127.0, 126.6, 126.6, 126.4 52.4, 49.5, 47.0, 42.0. **^{19}F NMR** (377 MHz, $CDCl_3$) δ -38.54. **HR-ESI-MS** *m/z* calcd. for $C_{23}H_{19}F_3S$ $[M]^+$: 384.1154, found: 384.1157. **HPLC** (Daicel Chiralpak OD column, *i*-PrOH/hexane = 0.7/99.3, 1 mL/min, 222 nm) t_1 = 4.1 min (minor), t_2 = 4.6 min (major).

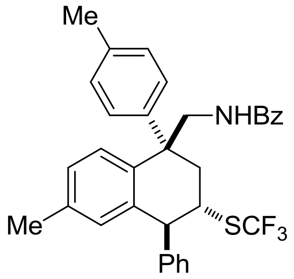

***N*-(((1*R*,3*S*,4*S*)-6-Methyl-4-phenyl-1-(p-tolyl)-3-((trifluoromethyl)thio)-1,2,3,4-tetrahydronaphthalen-1-yl)methyl)benzamide (2u):** Prepared by method A and purified by flash silica gel column chromatography (eluent: PE/EtOAc = 15/1 to 8/1 with 0.2% Et_3N, v/v) to afford **2u** as a white solid. mp: 125.8–128.3 ℃. 56.6 mg, >99% yield, 99.4% ee. $[\alpha]^{25}_D$ = +46.1 (c = 0.2, $CHCl_3$). **IR** (KBr): 3370, 3025, 2925, 1641, 1539, 1449, 1262, 1077, 1030, 808 cm^{-1}. **^{1}H NMR** (400 MHz, $CDCl_3$) δ 7.63 (d, *J* = 7.3 Hz, 2H), 7.50 (t, *J* = 7.3 Hz, 1H), 7.41 (t, *J* = 7.5 Hz, 2H), 7.24 (s, 1H), 7.14 (s, 5H), 7.10 – 6.99 (m, 6H), 6.76 (s, 1H), 6.22 (d, *J* = 5.4 Hz, 1H), 4.49 (dd, *J* = 14.2, 7.4 Hz, 1H), 4.30 (d, *J* = 6.7 Hz, 1H), 4.00 (dd, *J* = 14.2, 4.7 Hz, 1H), 3.88 (td, *J* = 8.1, 3.8 Hz, 1H), 2.74 (dd, *J* = 14.3, 2.9 Hz, 1H), 2.51 (dd, *J* = 14.4, 8.4 Hz, 1H), 2.35 (s, 3H), 2.22 (s, 3H). **^{13}C NMR** (101 MHz, $CDCl_3$) δ 167.344, 143.9, 143.0, 137.9, 137.2, 137.0, 135.9, 135.6 (q, *J* = 307.3 Hz), 134.3, 132.6, 132.3, 131.7, 129.5, 129.4, 129.0, 128.8, 128.7, 128.7, 128.3, 128.0, 127.1, 126.9, 126.5, 52.0, 47.9, 47.4, 46.2, 40.3, 21.2, 21.1. **^{19}F NMR** (377 MHz, $CDCl_3$) δ -39.96. **HR-ESI-MS** *m/z* calcd. for $C_{33}H_{29}ONF_3S$ $[M-H]^-$: 544.1927, found: 544.1929. **HPLC** (Daicel Chiralpak IA column, *i*-PrOH/hexane = 7/93, 1 mL/min, 238 nm) t_1 = 12.9 min (major), t_2 = 17.4 min (minor); t_3 = 10.9 min (diastereomer), t_4 = 18.5 min (diastereomer).

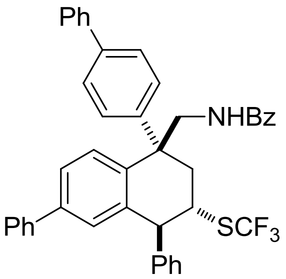

***N*-(((1*R*,3*S*,4*S*)-1-([1,1'-Biphenyl]-4-yl)-4,6-diphenyl-3-((trifluoromethyl)thio)-1,2,3,4-tetrahydronaphthalen-1-yl)methyl)benzamide (2v):** Prepared by method A and purified by flash silica gel column chromatography (eluent: PE/EtOAc = 15/1 to 8/1 with 0.2% Et_3N, v/v) to afford **2v** as a white solid. mp: 101.5–103.2 ℃. 56.9 mg, 86% yield, 99.2% ee, *dr* = 50:1. $[\alpha]^{26}_D$ = +37.4 (c = 0.2, $CHCl_3$). **IR** (KBr): 3333, 3029, 2924, 2855, 1659, 1516, 1485, 1451, 1262, 1109, 762, 697 cm^{-1}. **^{1}H NMR** (400 MHz, $CDCl_3$) δ 7.66 (d, *J* = 7.6 Hz, 2H), 7.61 (t, *J* = 6.3 Hz, 4H), 7.55 – 7.33 (m, 13H), 7.33 – 7.24 (m, 2H), 7.21 – 7.14 (m, 2H), 7.14 – 7.06 (m, 4H), 6.29 (s, 1H), 4.58 (dd, *J* = 14.1, 7.1 Hz, 1H), 4.41 (d, *J* = 7.1 Hz, 1H), 4.14 (dd, *J* = 14.2, 4.6 Hz, 1H), 4.01 (d, *J* = 22.8 Hz, 1H), 2.85 (d, *J* = 13.8 Hz, 1H), 2.63 (dd, *J* = 14.3, 8.9 Hz, 1H). **^{13}C NMR** (101 MHz, $CDCl_3$) δ 167.4, 144.9, 143.5, 140.4, 140.3, 140.2, 140.1, 138.8, 138.0, 135.6 (q, *J* = 307.4 Hz), 134.3, 132.6, 131.8, 130.3, 129.5, 129.1, 129.0, 128.9, 128.8, 128.5, 127.7, 127.6, 127.4, 127.4, 127.1, 127.0, 126.4,

126.0, 52.3, 47.9, 47.9, 46.2, 40.9. **^{19}F NMR** (377 MHz, $CDCl_3$) δ -39.73. **HR-ESI-MS** *m/z* calcd. for $C_{43}H_{33}ONF_3S$ [M-H]$^-$: 668.2240, found: 668.2239. **HPLC** (Daicel Chiralpak IA column, *i*-PrOH/hexane = 10/90, 1 mL/min, 230 nm) t_1 = 18.8 min (major), t_2 = 24.8 min (minor); t_3 = 17.6 min (diastereomer), t_4 = 30.1 min (diastereomer)

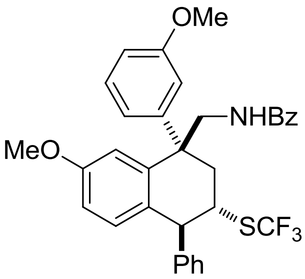

***N*-(((1*R*,3*S*,4*S*)-7-methoxy-1-(3-methoxyphenyl)-4-phenyl-3-((trifluoromethyl)thio)-1,2,3,4-tetrahydronaphthalen-1-yl)methyl)benzamide (2w):** Two regioisomers (about 5:3 from crude NMR) were obtained by method A. The structure of minor isomer was confirmed by the X-ray study. The major isomer **2w** was purified by flash silica gel column chromatography (eluent: PE/EtOAc = 15/1 to 10/1 with 0.2% Et_3N, v/v) as a white solid. mp: 62.4–63.6 ℃. 29.8 mg, 52% yield, 99.5% ee. $[\alpha]^{26}_D$ = +36.8 (c = 0.2, $CHCl_3$). **IR** (KBr): 3333 3027, 2925, 1658, 1605, 1581, 1487, 1453, 1261, 1106, 1033, 800, 703 cm^{-1}. **^{1}H NMR** (400 MHz, $CDCl_3$) δ 7.65 (d, *J* = 7.2 Hz, 2H), 7.50 (t, *J* = 7.4 Hz, 1H), 7.41 (t, *J* = 7.5 Hz, 2H), 7.32 – 7.26 (m, 1H), 7.18 (t, *J* = 7.2 Hz, 1H), 7.12 (t, *J* = 7.3 Hz, 2H), 7.05 (d, *J* = 7.2 Hz, 2H), 6.88 (d, *J* = 8.1 Hz, 1H), 6.82 (dd, *J* = 8.5, 5.4 Hz, 3H), 6.74 (dd, *J* = 8.7, 2.5 Hz, 1H), 6.60 (d, *J* = 2.3 Hz, 1H), 6.25 (s, 1H), 4.48 (dd, *J* = 14.3, 7.0 Hz, 1H), 4.21 (d, *J* = 7.9 Hz, 1H), 4.08 (dd, *J* = 14.3, 5.2 Hz, 1H), 3.89 (tt, *J* = 10.7, 5.3 Hz, 1H), 3.79 (s, 3H), 3.68 (s, 3H), 2.76 (dd, *J* = 14.3, 3.0 Hz, 1H), 2.56 (dd, *J* = 14.3, 9.3 Hz, 1H). **^{13}C NMR** (101 MHz, $CDCl_3$) δ 167.4, 159.9, 158.4, 147.5, 143.7, 140.4, 135.6 (q, *J* = 307.4 Hz), 134.3, 132.7, 132.5, 131.8, 130.6, 129.8, 129.5, 129.1, 128.8, 128.8, 127.2, 127.0, 126.0, 120.3, 114.7, 113.9, 113.6, 112.1, 55.5, 55.4, 51.4, 48.5, 47.8, 46.2, 41.6. **^{19}F NMR** (377 MHz, $CDCl_3$) δ -39.67. **HR-ESI-MS** *m/z* calcd. for $C_{33}H_{29}O_3NF_3S$ [M-H]$^-$: 576.1826, found: 576.1825. **HPLC** (Daicel Chiralpak ID column, *i*-PrOH/hexane = 3/97, 1 mL/min, 220 nm) t_1 = 54.9 min (minor), t_2 = 59.2 min (major).

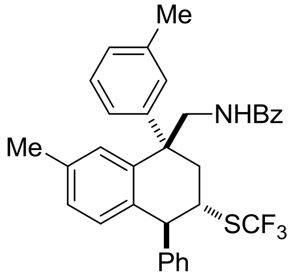

***N*-(((1*R*,3*S*,4*S*)-7-Methyl-4-phenyl-1-(m-tolyl)-3-((trifluoromethyl)thio)-1,2,3,4-tetrahydronaphthalen-1-yl)methyl)benzamide (2x):** Two regioisomers (about 4:1 from crude NMR) were obtained by method A. The major product **2x** was purified by flash silica gel column chromatography

(eluent: PE/EtOAc = 15/1 to 8/1 with 0.2% Et_3N, v/v) as a white solid. mp: 87.7–89.9 ℃. 46.3 mg, 85% yield, 99.6% ee. $[\alpha]^{26}_{D}$ = +33.2 (c = 0.2, $CHCl_3$). **IR** (KBr): 3359, 3028, 2960, 1641, 1542, 1488, 1453, 1261, 1103, 1023, 801, 702 cm^{-1}. **^{1}H NMR** (400 MHz, $CDCl_3$) δ 7.64 (d, *J* = 7.4 Hz, 2H), 7.49 (t, *J* = 7.3 Hz, 1H), 7.41 (t, *J* = 7.5 Hz, 2H), 7.24 – 7.03 (m, 9H), 6.98 (d, *J* = 7.9 Hz, 1H), 6.86 (s, 1H), 6.78 (d, *J* = 8.0 Hz, 1H), 6.22 (s, 1H), 4.50 (dd, *J* = 14.2, 7.1 Hz, 1H), 4.23 (d, *J* = 8.1 Hz, 1H), 4.08 (dd, *J* = 14.2, 4.9 Hz, 1H), 3.92 (td, *J* = 9.3, 3.3 Hz, 1H), 2.75 (dd, *J* = 14.2, 2.7 Hz, 1H), 2.63 – 2.48 (m, 1H), 2.35 (s, 3H), 2.24 (s, 3H). **^{13}C NMR** (101 MHz, $CDCl_3$) δ 167.4, 146.1, 143.7, 139.0, 138.5, 136.6, 135.6, 135.5, 134.4, 132.5 (q, *J* = 307.3 Hz), 131.7, 131.4, 129.5, 129.3, 129.1, 128.8, 128.7, 128.7, 128.6, 128.2, 127.2, 126.9, 126.4, 125.1, 51.8, 48.2, 47.8, 46.3, 42.2, 21.8, 21.3. **^{19}F NMR** (377 MHz, $CDCl_3$) δ -39.58. **HR-ESI-MS** *m/z* calcd. for $C_{33}H_{29}ONF_3S$ $[M-H]^-$: 544.1927, found: 544.1925. **HPLC** (Daicel Chiralpak IA column, *i*-PrOH/hexane = 10/90, 1 mL/min, 222 nm) t_1 = 7.6 min (major), t_2 = 11.5 min (minor).

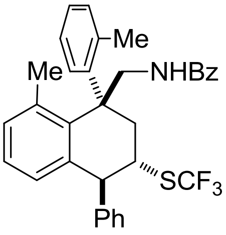

***N*-(((1*R*,3*S*,4*S*)-8-Methyl-4-phenyl-1-(o-tolyl)-3-((trifluoromethyl)thio)-1,2,3,4-tetrahydronaphthalen-1-yl)methyl)benzamide (2y):** Prepared by method A at -60 °C and purified by flash silica gel column chromatography (eluent: PE/EtOAc = 15/1 to 8/1 with 0.2% Et_3N, v/v) to afford **2y** as a white solid. mp: 61.0–62.4 ℃. 38.8 mg, 71% yield, 99.1% ee. $[\alpha]^{26}_{D}$ = +134.1 (c = 0.2, $CHCl_3$). **IR** (KBr): 3436, 2027, 2924, 1669, 1515, 1456, 1262, 1106, 801 cm^{-1}. **^{1}H NMR** (400 MHz, $CDCl_3$) δ 7.64 (d, *J* = 7.6 Hz, 2H), 7.53 (dd, *J* = 14.1, 7.3 Hz, 2H), 7.42 (t, *J* = 7.5 Hz, 2H), 7.31 (t, *J* = 7.5 Hz, 1H), 7.20 (dt, *J* = 14.6, 6.5 Hz, 4H), 7.12 (d, *J* = 7.5 Hz, 1H), 7.01 (dd, *J* = 21.0, 7.4 Hz, 3H), 6.93 (d, *J* = 7.3 Hz, 1H), 6.62 (d, *J* = 7.7 Hz, 1H), 6.14 (s, 1H), 4.83 (dd, *J* = 14.3, 6.9 Hz, 1H), 4.38 (dd, *J* = 14.3, 4.1 Hz, 1H), 4.11 – 3.93 (m, 2H), 2.76 (d, *J* = 14.2 Hz, 1H), 2.68 – 2.57 (m, 1H), 1.78 (s, 3H), 1.73 (s, 3H). **^{13}C NMR** (101 MHz, $CDCl_3$) δ 167.2, 145.4, 143.0, 139.8, 137.7, 136.4, 135.7, 135.3 (q, *J* = 307.4 Hz), 134.4, 133.5, 132.2, 131.8, 131.5, 129.8, 129.5, 129.1, 128.8, 128.8, 127.4, 127.3, 127.0, 127.0, 126.1, 125.4, 52. 4, 48.6, 47.3, 46.1, 46.0, 22.2, 21.3. **^{19}F NMR** (377 MHz, $CDCl_3$) δ -38.00. **HR-ESI-MS** *m/z* calcd. for $C_{33}H_{29}ONF_3S$ $[M-H]^-$: 544.1927, found: 544.1925. **HPLC** (Daicel Chiralpak IA column, *i*-PrOH/hexane = 30/70, 1 mL/min, 222 nm) t_1 = 5.1 min (major), t_2 = 7.5 min (minor).

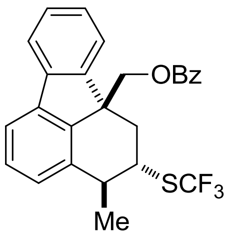

((4*S*,5*S*,6a*R*)-4-Methyl-5-((trifluoromethyl)thio)-4,5,6,6a-tetrahydrofluoranthen-6a-yl)methyl benzoate (2z): Prepared by method A using 2 equivalents of BF_3OEt_2 (25 μL, 0.2 mmol) as acid and purified by flash silica gel column chromatography (eluent: PE/EtOAc = 200/1 to 100/1 with 0.2% Et_3N, v/v) to afford **2z** as a white solid. mp: 27.3–29.1 ℃. 42.1 mg, 93% yield and 94% ee. $[\alpha]^{26}_{D}$ = 136.7 (c = 0.2, $CHCl_3$). **IR** (KBr): 2925, 1454, 1264, 1112, 801, 709 cm^{-1}. **^{1}H NMR** (400 MHz, $CDCl_3$) δ 7.88 (m, 2H), 7.75 (d, *J* = 7.5 Hz, 1H), 7.67 – 7.59 (m, 2H), 7.53 (t, *J* = 7.4 Hz, 1H), 7.47 – 7.34 (m, 4H), 7.28 (dd, *J* = 8.0, 7.3 Hz, 1H), 7.24 (d, *J* = 3.4 Hz, 1H), 4.69 (d, *J* = 11.1 Hz, 1H), 4.27 (d, *J* = 11.1 Hz, 1H), 3.24 – 3.06 (m, 3H), 2.12 (dd, *J* = 14.5, 8.1 Hz, 1H), 1.66 (d, *J* = 5.7 Hz, 3H). **^{13}C NMR** (101 MHz, $CDCl_3$) δ 166.3, 150.1, 144.3, 140.9, 139.5, 137.8, 135.8 (q, *J* = 306.5 Hz), 133.1, 132.8, 130.1, 129.7, 129.6, 129.1, 128.5, 128.2, 127.5, 126.7, 124.4, 123.1, 120.9, 118.8, 67.0, 50.4, 47.8, 36.9, 35.6, 16.7. **^{19}F NMR** (377 MHz, $CDCl_3$) δ -39.49. **HR-ESI-MS** *m/z* calcd. for $C_{17}H_{14}O_4N_2BrF_3S_2$ $[M]^+$: 454.1209, found: 454.1208. **HPLC** (Daicel Chiralpak IA column, *i*-PrOH/hexane = 4/96, 1 mL/min, 268 nm) t_1 = 5.9 min (major), t_2 = 7.9 min (minor); t_3 = 4.1 min (diastereomer), t_4 = 4.6 min (diastereomer)

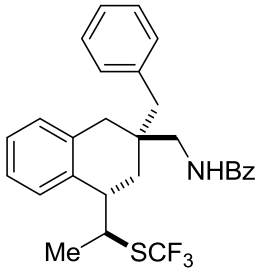

***N*-(((2*R*,4*S*)-2-Benzyl-4-((S)-1-((trifluoromethyl)thio)ethyl)-1,2,3,4-tetrahydronaphthalen-2-yl)methyl)benzamide (4):** Prepared by method A using 2 equivalents of TMSOTf at -60 °C and purified by flash silica gel column chromatography (eluent: PE/EtOAc = 15/1 to 10/1 with 0.2% Et_3N, v/v) to afford **4** as a white solid. mp: 48.7–52.1 ℃. 38.6 mg, 80% yield, 74% ee, *dr* = 14:1. $[\alpha]^{25}_{D}$ = +2.3 (c = 0.2, $CHCl_3$). **IR** (KBr): 3313, 2027, 2925, 1641, 1539, 1453, 1261, 1100, 1027, 801, 703 cm^{-1}. **^{1}H NMR** (400 MHz, $CDCl_3$) δ 7.46 (d, *J* = 7.6 Hz, 3H), 7.42 – 7.19 (m, 8H), 7.13 (d, *J* = 7.1 Hz, 1H), 7.07 (d, *J* = 7.2 Hz, 2H), 5.84 (s, 1H), 4.04 (d, *J* = 4.9 Hz, 1H), 3.63 (dd, *J* = 13.8, 7.3 Hz, 1H), 3.41 (dd, *J* = 13.5, 5.3 Hz, 2H), 2.77 – 2.57 (m, 3H), 2.52 (d, *J* = 13.6 Hz, 1H), 1.99 (dd, *J* = 13.2, 7.0 Hz, 1H), 1.62 – 1.41 (m, 4H). **^{13}C NMR** (101 MHz, $CDCl_3$) δ 167.6, 138.3, 136.0, 135.8, 134.6, 132.9 (q, *J* = 306.1 Hz), 131.6, 130.2, 130.0, 129.9, 128.9, 128.6, 127.2, 127.1, 126.9, 126.8, 48.8, 47.0, 41.1, 40.5, 38.9, 37.0, 34.3, 21.5. **^{19}F NMR** (377 MHz, $CDCl_3$) δ -39.26. H **HR-ESI-MS** *m/z* calcd. for $C_{28}H_{27}ONF_3S$ $[M-H]^-$: 482.1771,

found: 482.1769. **HPLC** (Daicel Chiralpak IA column, *i*-PrOH/hexane = 9/91, 0.5 mL/min, 222 nm) t_1 = 22.0 min (minor), t_2 = 24.3 min (major).

Transformation of the products

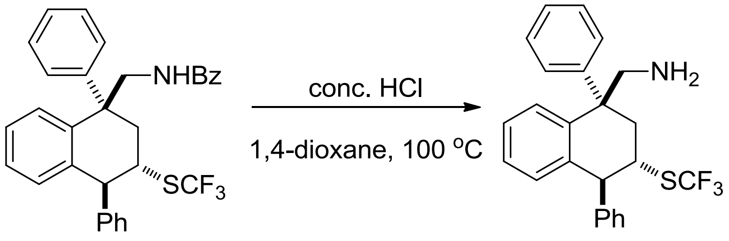

2a **2ab**

To a solution of **2a** (0.1 mmol, 51.8 mg) in 1,4-dioxane (2.0 ml) was added concentrated HCl (0.8 mL, 12M). The solution was refluxed at 100 °C for 4 d. The resulting mixture was quenched with 1M NaOH (adjust pH to >14). The mixture was extracted with DCM (10 mL x 3). The combined organic layers were dried over anhydrous Na_2SO_4 and concentrated. The crude product **2ab** was purified by flash column chromatography (eluent: PE/EtOAc = 5/1 to 3/1 with 0.2% Et_3N, v/v).

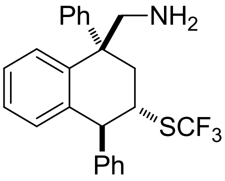

((1*R*,3*S*,4*S*)-1,4-Diphenyl-3-((trifluoromethyl)thio)-1,2,3,4-tetrahydronaphthalen-1-yl)methanamine (2ab): White solid. mp: 97.4–100.0 °C. 39.6 mg, 96% yield, 99% ee. $[\alpha]^{26}_D$ = +86.2 (c = 0.2, $CHCl_3$). **IR** (KBr): 3407, 3337, 3063, 2917, 2856, 1598, 1489, 1443, 1152, 1102, 701 cm^{-1}. **^{1}H NMR** (400 MHz, $CDCl_3$) δ 7.41 – 7.27 (m, 5H), 7.27 – 7.17 (m, 5H), 7.17 – 7.05 (m, 2H), 7.01 – 6.94 (m, 1H), 6.83 (d, *J* = 7.4 Hz, 1H), 4.29 – 4.09 (m, 2H), 3.64 – 3.38 (m, 2H), 2.93 – 2.75 (m, 1H), 2.55 – 2.37 (m, 1H), 1.28 (s, 2H). **^{13}C NMR** (101 MHz, $CDCl_3$) δ 147.6, 143.8, 140.1, 139.1, 135.7 (q, *J* = 307.1 Hz), 132.7, 131.0, 129.6, 129.4, 129.4, 128.8, 128.6, 128.0, 127.3, 126.9, 126.7, 126.5, 52.1, 51.8, 49.2, 46.6, 43.9. **^{19}F NMR** (377 MHz, $CDCl_3$) δ -39.32. **HR-ESI-MS** *m/z* calcd. for $C_{24}H_{23}NF_3S$ $[M+H]^+$: 414.1498, found: 414.1495. **HPLC** (Daicel Chiralpak IA column, *i*-PrOH/hexane = 5/95, 0.5 mL/min, 222 nm) t_1 = 10.5 min (minor), t_2 = 12.1 min (major).

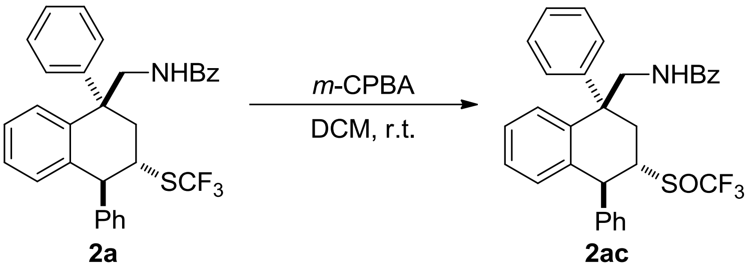

To a solution of **2a** (0.1 mmol, 51.8 mg) in DCM (4 ml) was added *m*-CPBA (0.3 mmol, 52.0 mg). The solution was stirred overnight, after which the solvent was evaporated and the resulting mixture was directly purified by flash column chromatography (eluent: PE/EtOAc = 15/1 to 8/1, v/v) to afford product **2ac**.

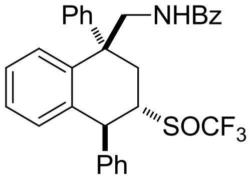

***N*-(((1*R*,3*S*,4*S*)-1,4-Diphenyl-3-((trifluoromethyl)sulfinyl)-1,2,3,4-tetrahydronaphthalen-1-yl)methyl)benzamide (2ac):** White solid. mp: 63.2–65.3 ℃. 33.6 mg, 62% yield, 99% ee. $[\alpha]^{26}_{D}$ = +66.9 (c = 0.2, $CHCl_3$). **IR** (KBr): 3349, 3027, 2927, 1659, 1522, 1487, 1455, 1281, 1175, 1137, 1073, 702 cm^{-1}. **^{1}H NMR** (400 MHz, $CDCl_3$) δ 7.65 (d, *J* = 7.3 Hz, 2H), 7.50 (t, *J* = 7.3 Hz, 1H), 7.36 (tt, *J* = 12.5, 9.5 Hz, 10H), 7.25 – 7.21 (m, 2H), 7.16 – 7.08 (m, 2H), 6.98 – 6.78 (m, 2H), 6.28 (s, 1H), 4.56 (d, *J* = 11.4 Hz, 1H), 4.36 (d, *J* = 6.0 Hz, 2H), 3.92 (td, *J* = 11.6, 3.4 Hz, 1H), 2.92 – 2.77 (m, 1H), 2.58 – 2.43 (m, 1H). **^{13}C NMR** (101 MHz, $CDCl_3$) δ 167.5, 145.9 141.5, 140.1, 138.0, 134.2, 131.9, 130.6, 130.5, 129.7, 129.5, 129.2, 129.0, 128.9, 128.1, 127.6, 127.6, 127.4, 127.3, 127.2, 126.9, 123.9 (q, *J* = 335.6 Hz), 120.6, 60.0, 48.0, 47.7, 46.6, 32.0. **^{19}F NMR** (377 MHz, $CDCl_3$) δ -68.90. **HR-ESI-MS** *m/z* calcd. for $C_{31}H_{25}O_2NF_3S$ $[M-H]^-$: 532.1564, found: 532.1562. **HPLC** (Daicel Chiralpak IA column, *i*-PrOH/hexane = 10/90, 1 mL/min, 222 nm) t_1 = 12.1 min (major), t_2 = 20.1 min (minor).

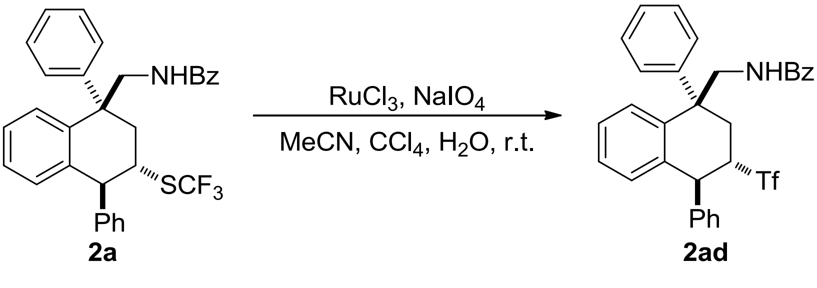

A literature method of oxidation of $-SCF_3$ to -Tf was used.[10] To an intensively

stirred mixture of sulfane **2a** (0.5 mmol, 259 mg) in the mixed solvents of CCl_4 (3 mL), CH_3CN (3 mL) and H_2O (6 ml) were subsequently added $NaIO_4$ (321 mg, 1.5 mmol) and $RuCl_3$ (0.05 mmol, 10.0 mg) at room temperature. After the mixture was stirred for 14 h, it was treated subsequently with DCM (10 mL) and H_2O (10 mL). The mixture was extracted with DCM (10 mL x 3). The combined organic layers were dried over anhydrous Na_2SO_4 and concentrated. The crude product **2ad** was purified by flash column chromatography (eluent: PE/EtOAc = 15/1 to 8/1, v/v).

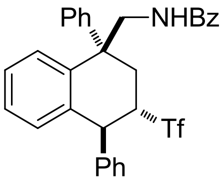

***N*-(((1*R*,3*S*,4*S*)-1,4-Diphenyl-3-((trifluoromethyl)sulfonyl)-1,2,3,4-tetrahydronaphthalen-1-yl)methyl)benzamide (2ad):** White solid. mp: 74.2–76.0 ℃. 280 mg, >99% yield, 99% ee. $[\alpha]^{26}_{D}$ = +88.7 (c = 0.2, $CHCl_3$). **IR** (KBr): 3343, 3028, 2933, 1661, 1520, 1487, 1353, 1204, 1112, 701, 518 cm^{-1}. **^{1}H NMR** (400 MHz, $CDCl_3$) δ 7.66 (d, *J* = 7.3 Hz, 2H), 7.50 (t, *J* = 7.3 Hz, 1H), 7.46 – 7.25 (m, 12H), 7.10 (p, *J* = 6.9 Hz, 2H), 6.93 – 6.87 (m, 1H), 6.87 – 6.79 (m, 1H), 6.36 (s, 1H), 4.83 (d, *J* = 9.6 Hz, 1H), 4.66 (dd, *J* = 17.2, 8.1 Hz, 1H), 4.46 (dd, *J* = 14.7, 7.7 Hz, 1H), 4.27 (dd, *J* = 14.7, 4.8 Hz, 1H), 2.71 (d, *J* = 7.9 Hz, 2H). **^{13}C NMR** (101 MHz, $CDCl_3$) δ 167.8, 145.0, 142.4, 139.2, 137.5, 134.0, 132.0, 131.4, 129.6, 129.2, 129.1, 128.9, 128.9, 127.8, 127.7, 127.6, 127.0, 127.0, 124.6 (q, *J* = 329.9 Hz), 121.3, 118.0, 114.7, 62.3, 48.1, 46.7, 44.8, 33.7. **^{19}F NMR** (377 MHz, $CDCl_3$) δ -75.20. **HR-ESI-MS** *m/z* calcd. for $C_{31}H_{25}O_3NF_3S$ $[M-H]^-$: 548.1513, found: 548.1511. **HPLC** (Daicel Chiralpak IA column, *i*-PrOH/hexane = 10/90, 1 mL/min, 222 nm) t_1 = 11.1 min (major), t_2 = 14.4 min (minor).

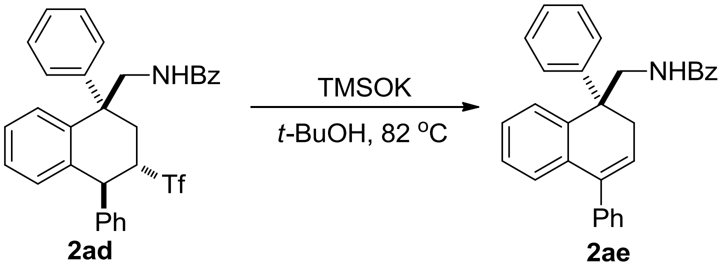

2ad **2ae**

To a stirred solution of TMSOK (0.2 mmol, 25.6 mg) in *t*-BuOH (2.5 mL) was added **2ad** (0.1 mmol, 55.0 mg) at room temperature. After the mixture was stirred at 82 °C for 24 h, it was treated subsequently with AcOH (0.2 mL) and DCM (10 mL).

The mixture was concentrated and the crude product **2ae** was purified by flash column chromatography (eluent: PE/EtOAc = 20/1 to 12/1, v/v).

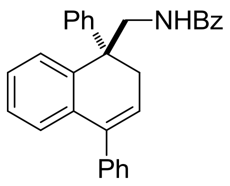

(*R*)-*N*-((1,4-Diphenyl-1,2-dihydronaphthalen-1-yl)methyl)benzamide (2ae): White solid. mp: 70.8–72.6 ℃. 29.1 mg, 70% yield, 99% ee. $[\alpha]^{25}_{D}$ = -98.1 (c = 0.2, $CHCl_3$). **IR** (KBr): 3325, 3027, 2924, 1649, 1524, 1487, 1445, 1282, 1075, 1028, 700 cm^{-1}. **^{1}H NMR** (400 MHz, $CDCl_3$) δ 7.59 (d, *J* = 7.3 Hz, 2H), 7.45 (t, *J* = 7.3 Hz, 1H), 7.39 – 7.25 (m, 12H), 7.25 – 7.20 (m, 3H), 7.14 (d, *J* = 7.5 Hz, 1H), 6.10 (s, 1H), 5.96 (t, *J* = 4.5 Hz, 1H), 4.29 (dd, *J* = 13.9, 6.1 Hz, 1H), 4.16 (dd, *J* = 13.9, 5.3 Hz, 1H), 3.06 (dd, *J* = 17.2, 5.0 Hz, 1H), 2.76 (dd, *J* = 17.2, 4.1 Hz, 1H). **^{13}C NMR** (101 MHz, $CDCl_3$) δ 167.5, 144.5, 140.4, 139.7, 139.3, 135.9, 134.7, 131.6, 128.8, 128.7, 128.4, 128.4, 127.7, 127.7, 127.4, 127.2, 126.9, 126.8, 125.6, 47.3, 45.6, 35.6. **HR-ESI-MS** *m/z* calcd. for $C_{30}H_{24}ON$ $[M-H]^-$: 414.1863, found: 414.1862. **HPLC** (Daicel Chiralpak IA column, *i*-PrOH/hexane = 10/90, 1 mL/min, 240 nm) t_1 = 10.3 min (major), t_2 = 14.9 min (minor).

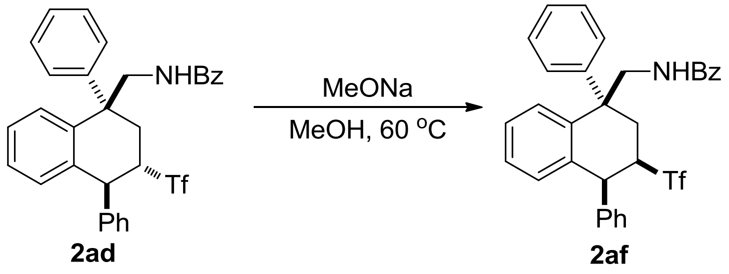

To a stirred solution of MeONa (0.5 mmol, prepared by mixing of Na and MeOH, and stir for 0.5 h) in MeOH (1 mL) was added **2ad** (0.1 mmol, 55.0 mg) at room temperature. After the mixture was stirred at 60 °C for 24 h, it was treated subsequently with AcOH (0.2 mL) and DCM (10 mL). The mixture was concentrated and the crude product **2af** was purified by flash column chromatography (eluent: PE/EtOAc = 12/1 to 6/1, v/v).

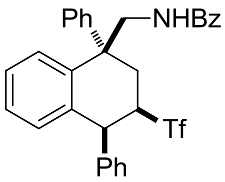

***N*-(((1*R*,3*R*,4*S*)-1,4-Diphenyl-3-((trifluoromethyl)sulfonyl)-1,2,3,4-tetrahydronaphthalen-1-yl)methyl)benzamide (2af):** White solid. mp: 72.7–73.9 ℃. 42.1 mg, 76% yield, 99% ee. $[\alpha]^{25}_{D}$ = +50.8 (c = 0.2, $CHCl_3$). **IR** (KBr): 3415, 3026, 2923, 1625, 1447, 1413, 699 cm^{-1}. **^{1}H NMR** (400 MHz, $CDCl_3$) δ 7.66 (d, *J* = 7.4 Hz, 2H), 7.54 (t, *J* = 7.4 Hz, 1H), 7.43 (t, *J* = 7.5 Hz, 4H), 7.39 – 7.27 (m, 4H), 7.17 – 6.99 (m, 6H), 6.82 (t, *J* = 7.6 Hz, 2H), 6.24 (d, *J* = 7.6 Hz, 1H), 5.03 (dd, *J* = 14.0, 9.6 Hz, 1H), 4.93 (d, *J* = 4.4 Hz, 1H), 3.92 (dd, *J* = 14.1, 2.8 Hz, 1H), 3.77 (dd, *J* = 12.5, 4.3 Hz, 1H), 2.98 (t, *J* = 13.5 Hz, 1H), 2.43 (d, *J* = 14.0 Hz, 1H). **^{13}C NMR** (101 MHz, $CDCl_3$) δ 167.1, 145.0, 139.9, 138.9, 136.5, 133.7, 132.1, 132.0, 130.8, 128.9, 128.6, 128.5, 128.3, 128.0, 127.8, 127.8, 127.5, 127.0, 124.2 (q, *J* = 330.7 Hz), 120.9, 117.6, 114.4, 57.5, 50.3, 47.1, 43.4, 31.1. **^{19}F NMR** (377 MHz, $CDCl_3$) δ -76.13. **HR-ESI-MS** *m/z* calcd. for $C_{31}H_{25}O_3NF_3S$ $[M-H]^-$: 548.1513, found: 548.1509. **HPLC** (Daicel Chiralpak IA column, *i*-PrOH/hexane = 30/70, 1 mL/min, 222 nm) t_1 = 6.0 min (major), t_2 = 16.1 min (minor).

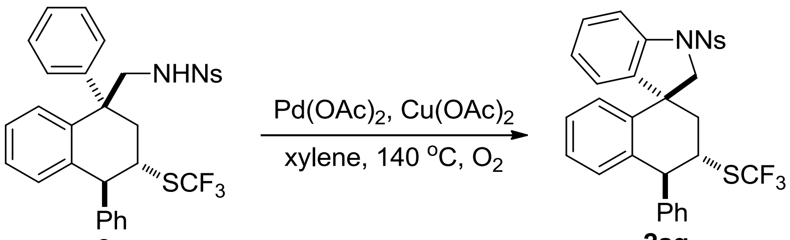

2o **2ag**

A modified literature method was used.[11] An oven-dried Schlenk tube (15 mL in volume) was cooled under vacuum. **2o** (0.09 mmol, 53.8 mg), $Pd(OAc)_2$ (0.02 mmol, 4.5 mg), anhydrous $Cu(OAc)_2$ (36.2 mg, 0.2 mmol) were added under air. The tube was evacuated and refilled with O_2 for three times, after which xylene (3 mL) was added. The mixture was refluxed under O_2 atmosphere at 140 °C for 3 d. After cooling to room temperature, water (5 mL) was added and the mixture was extracted with DCM (8 mL x 3). The combined organic layers were dried over anhydrous Na_2SO_4 and concentrated. The crude product **2ae** was purified by flash column chromatography (eluent: PE/EtOAc = 20/1 to 12/1, v/v).

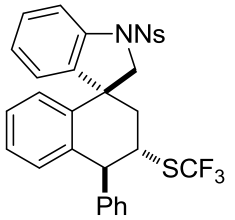

(1'*S*,3'*S*,4'*S*)-1-((4-Nitrophenyl)sulfonyl)-4'-phenyl-3'-((trifluoromethyl)thio)-3',4'-dihydro-2'H-spiro[indoline-3,1'-naphthalene] (2ag): Yellow solid. mp: 92.5–94.0 ℃. 33.8 mg, 62% yield, 94% ee. $[\alpha]^{25}_{D}$ = -29.1 (c = 0.2, $CHCl_3$). **IR** (KBr): 3028, 2929, 1604, 1529, 1461, 1350, 1167, 1101, 704, 622, 463 cm^{-1}. **^{1}H NMR** (400 MHz, $CDCl_3$) δ 7.88 (d, *J* = 8.8 Hz, 2H), 7.60 (dd, *J* = 8.2, 4.9 Hz, 3H), 7.42 – 7.21 (m, 5H), 7.12 (t, *J* = 7.4 Hz, 1H), 7.06 – 6.95 (m, 4H), 6.56 (dd, *J* = 17.0, 7.6 Hz, 3H), 4.50 (d, *J* = 11.5 Hz, 1H), 4.26 (d, *J* = 11.5 Hz, 1H), 3.83 – 3.74 (m, 1H), 3.64 (t, *J* = 5.5 Hz, 1H), 3.01 (dd, *J* = 14.1, 1.8 Hz, 1H), 2.57 (dd, *J* = 14.1, 7.4 Hz, 1H). **^{13}C NMR** (101 MHz, $CDCl_3$) δ 150.2, 143.6, 143.5, 141.7, 139.5, 137.9, 135.4 (q, *J* = 307.3 Hz), 133.7, 132.3, 130.0, 129.3, 129.0, 128.9, 128.8, 127.7, 127.6, 127.6, 127.1, 126.2, 124.5, 124.1, 114.5, 68.5, 50.0, 47.6, 46.6, 42.5. **^{19}F NMR** (377 MHz, $CDCl_3$) δ -41.26. **HR-EI-MS** *m/z* calcd. for $C_{30}H_{23}O_4N_2F_3S$ $[M]^+$: 596.1046, found: 596.1050. **HPLC** (Daicel Chiralpak IA column, *i*-PrOH/hexane = 10/90, 1 mL/min, 222 nm) t_1 = 7.6 min (major), t_2 = 9.1 min (minor).

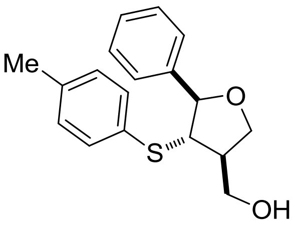

((3*R*,4*S*,5*R*)-5-Phenyl-4-(p-tolylthio)tetrahydrofuran-3-yl)methanol (7): Prepared by method B and purified by flash silica gel column chromatography (eluent: PE/EtOAc = 10/1 to 5/1, v/v) to afford **7** as a colourless oil. 20.0 mg, 67% yield, 92% ee, *dr*=9:1. $[\alpha]^{25}_{D}$ = +10.8 (c = 0.2, $CHCl_3$). **IR** (KBr): 3420, 3031, 2923, 2867, 1492, 1453, 1049, 809, 700 cm^{-1}. **^{1}H NMR** (400 MHz, $CDCl_3$) δ 7.41 – 7.16 (m, 7H), 7.02 (d, *J* = 7.9 Hz, 2H), 4.67 (d, *J* = 8.0 Hz, 1H), 4.02 (qd, *J* = 9.0, 6.2 Hz, 2H), 3.73 – 3.60 (m, 2H), 3.18 (dd, *J* = 8.0, 6.7 Hz, 1H), 2.59 – 2.46 (m, 1H), 2.29 (s, 3H), 1.62 (br, 1H). **^{13}C NMR** (101 MHz, $CDCl_3$) δ = 140.27, 138.15, 133.67, 129.89, 128.50, 128.01, 126.38, 86.32, 77.16, 70.06, 63.53, 56.51, 49.49, 21.22. **HR-EI-MS** *m/z* calcd. for $C_{18}H_{20}O_2S$ $[M]^+$: 300.1179, found: 300.1175. **HPLC** (Daicel Chiralpak IC column, *i*-PrOH/hexane = 5/95, 1 mL/min, 254 nm) t_1 = 18.8 min (major), t_2 = 21.4 min (minor); t_1 = 24.9 min (diastereomer), t_2 = 29.2 min (diastereomer)

Supplementary Note 2

Computational details

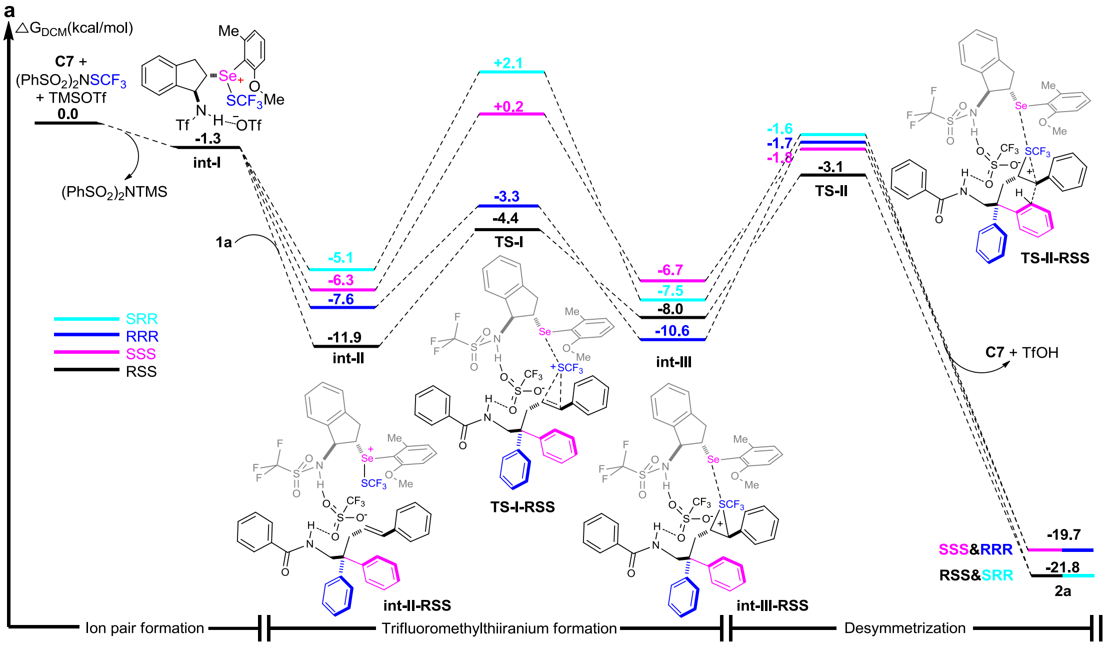

Supplementary Figure 175. DFT calculation for the reaction pathway of **1a** at 195.15 K: 99.9% $ee_{predicted}$, $dr_{predicted}$ = 37:1

Supplementary Table 4. Distortion interaction analysis at 195.15 K in kcal/mol for TS-I of 1a†

Entry	$\Delta E_{dist\text{-}cat}$	$\Delta E_{dist\text{-}sub}$	ΔE_{dist}	ΔE_i	ΔE_{act}	ΔH	ΔG
**TS-I-RSS**	22.3	10.3	32.6	-46.9	-14.3	-13.2	-3.1
**TS-I-SRR**	23.1	8.8	31.8	-39.6	-7.7	-6.5	3.4
**TS-I-SSS**	26.4	9.8	36.2	-46.0	-9.8	-8.7	1.5
**TS-I-RRR**	26.8	14.0	40.8	-62.4	-21.6	-12.9	-2.0

†$\Delta E_{dist} = \Delta E_{dist\text{-}cat} + \Delta E_{dist\text{-}sub}$; $\Delta E_{act} = \Delta E_{dist} + \Delta E_i$; ΔG related to **int-I**.

Supplementary Table 5. Distortion interaction analysis at 195.15 K in kcal/mol for TS-II of 1a†

Entry	$\Delta E_{dist\text{-}cat}$	$\Delta E_{dist\text{-}sub}$	ΔE_{dist}	ΔE_i	ΔE_{act}	ΔH	ΔG
**TS-II-RSS**	52.5	57.7	110.2	-124.2	-14.0	-13.8	-1.8
**TS-II-SRR**	59.8	50.9	110.7	-123.7	-13.0	-12.8	-0.3
**TS-II-SSS**	50.8	54.9	105.7	-117.7	-12.1	-12.6	-0.5
**TS-II-RRR**	58.5	49.8	108.3	-119.5	-11.1	-11.3	-0.4

†$\Delta E_{dist} = \Delta E_{dist\text{-}cat} + \Delta E_{dist\text{-}sub}$; $\Delta E_{act} = \Delta E_{dist} + \Delta E_i$; ΔG related to **int-II**.

The above tables show the difference of distortion and interaction energies in **TS-I** and **TS-II**. The two factors, distortion and interaction in each transition state, result in different ΔG and further determine the enantio- and diastereoselectivity of the reaction. For example, although the energy of distortion, especially the energy of substrate distortion of **TS-II-RSS** is high (ΔE_{dist} = 110.2 kcal/mol, $\Delta E_{dist\text{-}sub}$ = 57.7 kcal/mol), the interaction in this transition state largely offsets the unfavorable distortion (ΔE_i = -124.2 kcal/mol). So the mutual contribution of both interaction and distortion determines the energy of the transition state (ΔG = -1.8 kcal/mol) and the composition of the final product. For **TS-I-RRR**, the interaction mode in this transition state is beneficial for the system, but the distortion energy, especially the distortion of substrate, is high in this case. These two cofactors finally result in the total energy of **TS-I-RRR** (ΔG = -2.0 kcal/mol).

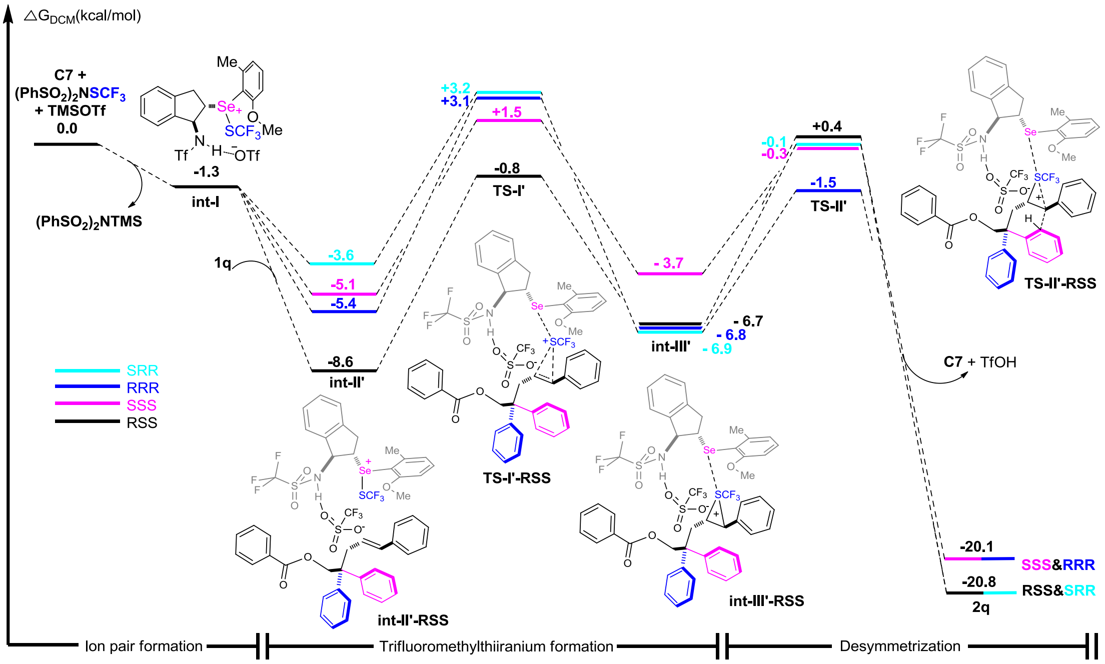

Supplementary Figure 176. DFT calculation for the reaction pathway of **1q** at 195.15 K: 99.6% $ee_{predicted}$, $dr_{predicted}$ = 19:1

Supplementary Table 6. Distortion interaction analysis at 195.15 K in kcal/mol for TS-I' of 1q†

Entry	$\Delta E_{dist\text{-}cat}$	$\Delta E_{dist\text{-}sub}$	ΔE_{dist}	ΔE_i	ΔE_{act}	ΔH	ΔG
**TS-I'-RSS**	23.1	9.2	32.3	-44.1	-11.8	-10.5	0.5
**TS-I'-SRR**	24.0	9.1	33.2	-40.6	-7.4	-6.0	4.5
**TS-I'-SSS**	26.2	10.1	36.3	-45.7	-9.4	-8.1	2.8
**TS-I'-RRR**	24.0	10.3	34.3	-42.8	-8.5	-7.1	4.4

† $\Delta E_{dist} = \Delta E_{dist\text{-}cat} + \Delta E_{dist\text{-}sub}$; $\Delta E_{act} = \Delta E_{dist} + \Delta E_i$; ΔG related to **int-I**.

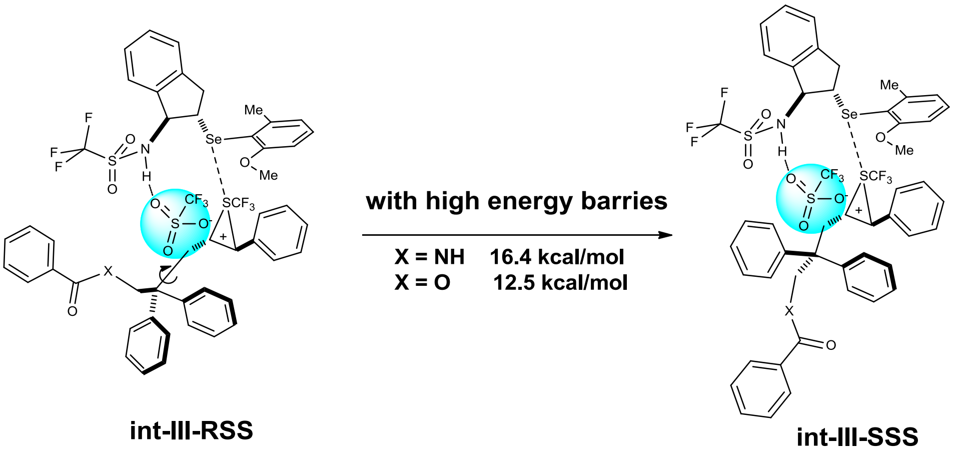

Supplementary Figure 177. DFT calculations of rotation barrier in **int-III-RSS.**

The rotation barrier for quaternary carbon center in **int-III-RSS** and **int-III'-RSS** was calculated. For both **1a** and **1q**, the energy barrier is high so that **int-III-SSS** and **int-III'-SSS** cannot be generated prior to the formation of **TS-II** and **TS-II'** through the rotation of **int-III-RSS** and **int-III'-RSS** in this step, respectively. We propose that the energy barrier comes from the hindrance of the binding anion.

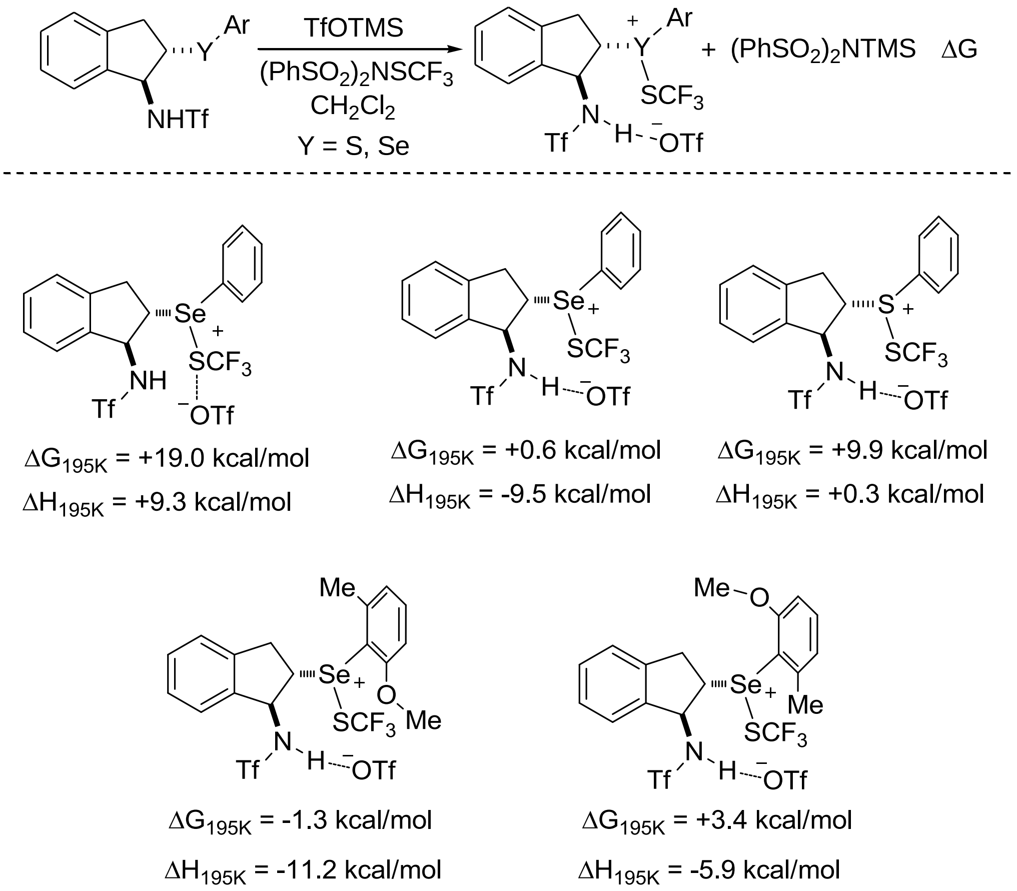

Supplementary Figure 178. Calculation of ΔG & ΔH with the aid of acid

Supplementary Table 7.

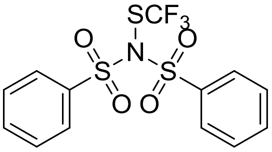

B3LYP /6-31G(d) 195.15 K Thermal correction to Gibbs Free Energy = 0.192884

Thermal correction to Enthalpy = 0.234837

M062x-D3/6-311+G(d,p) (IEFPCM, Dichloromethane) Energy = -2350.88627614

Standard orientation:

Center Number	Atomic Number	Atomic Type	Coordinates (Angstroms) X	Y	Z
1	7	0	0.363669	-0.498787	0.015248
2	16	0	0.557944	0.685437	1.306185
3	8	0	-0.696602	0.628440	2.053240
4	8	0	1.851482	0.479777	1.948063
5	16	0	-0.839555	-1.836002	0.180518
6	8	0	-0.539885	-2.692322	-0.961956
7	8	0	-0.793872	-2.320390	1.552816
8	6	0	0.618415	2.222577	0.386531
9	6	0	1.844734	2.866703	0.219167
10	6	0	-0.574481	2.762095	-0.101172
11	6	0	1.873270	4.082033	-0.465844
12	1	0	2.747733	2.423041	0.623514
13	6	0	-0.528511	3.975371	-0.785111
14	1	0	-1.515119	2.246786	0.062901
15	6	0	0.692185	4.631905	-0.967835
16	1	0	2.818588	4.598194	-0.604646
17	1	0	-1.445789	4.411494	-1.169667
18	1	0	0.720948	5.578651	-1.499916
19	6	0	-2.408516	-1.026585	-0.121234
20	6	0	-3.213209	-0.667568	0.960947
21	6	0	-2.808202	-0.823125	-1.444725
22	6	0	-4.449406	-0.074373	0.701968
23	1	0	-2.869470	-0.844157	1.972708
24	6	0	-4.044807	-0.226027	-1.684644
25	1	0	-2.171577	-1.143109	-2.262582
26	6	0	-4.861578	0.148481	-0.613873
27	1	0	-5.090322	0.210505	1.531047
28	1	0	-4.373908	-0.063144	-2.706755
29	1	0	-5.826490	0.609174	-0.806787
30	16	0	1.529570	-0.577384	-1.212095
31	6	0	2.842292	-1.677209	-0.504303

32	9	0	2.330676	-2.678126	0.210636
33	9	0	3.721152	-1.019388	0.259139
34	9	0	3.507090	-2.171200	-1.564975

Supplementary Table 8.

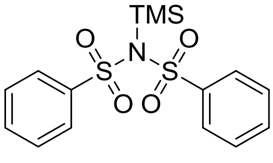

B3LYP /6-31G(d) 195.15 K Thermal correction to Gibbs Free Energy = 0.291312
Thermal correction to Enthalpy = 0.333398
M062x-D3/6-311+G(d,p) (IEFPCM, Dichloromethane) Energy = -2024.35313589
Standard orientation:

Center Number	Atomic Number	Atomic Type	Coordinates (Angstroms) X	Y	Z
1	7	0	-0.178767	-0.938605	-0.204603
2	16	0	-0.906059	0.082407	-1.384836
3	8	0	0.179750	0.709882	-2.139862
4	8	0	-1.962757	-0.653764	-2.082944
5	16	0	1.502984	-1.334712	-0.402555
6	8	0	1.718994	-2.295522	0.687142
7	8	0	1.794154	-1.706657	-1.781843
8	6	0	-1.692352	1.361588	-0.391992
9	6	0	-3.012566	1.703088	-0.687089
10	6	0	-0.954340	2.058603	0.567670
11	6	0	-3.609786	2.756329	0.007585
12	1	0	-3.556114	1.144975	-1.441322
13	6	0	-1.565132	3.103911	1.258674
14	1	0	0.075885	1.787397	0.773273
15	6	0	-2.889813	3.452445	0.979738
16	1	0	-4.637989	3.029369	-0.211476
17	1	0	-1.004676	3.649246	2.012598
18	1	0	-3.359353	4.269325	1.520652
19	6	0	2.419432	0.154663	0.005404
20	6	0	2.950621	0.936683	-1.020619
21	6	0	2.641246	0.455382	1.352431
22	6	0	3.707326	2.059242	-0.682468
23	1	0	2.763308	0.668967	-2.053023
24	6	0	3.395394	1.583529	1.673864
25	1	0	2.247320	-0.193584	2.127209

26	6	0	3.925310	2.384112	0.657934
27	1	0	4.126787	2.678814	-1.469601
28	1	0	3.579878	1.828749	2.715915
29	1	0	4.516619	3.259216	0.913233
30	14	0	-1.176420	-2.098581	0.860902
31	6	0	-2.979169	-1.552341	0.797040
32	1	0	-3.394396	-1.582196	-0.212798
33	1	0	-3.541520	-2.263248	1.417499
34	1	0	-3.142172	-0.554680	1.214914
35	6	0	-0.597234	-1.928418	2.645663
36	1	0	-1.314626	-2.432273	3.306126
37	1	0	0.388252	-2.371405	2.802316
38	1	0	-0.560904	-0.874890	2.947605
39	6	0	-1.037823	-3.842200	0.177154
40	1	0	-0.018171	-4.228007	0.252335
41	1	0	-1.704475	-4.514676	0.732105
42	1	0	-1.341014	-3.865054	-0.875583

Supplementary Table 9.

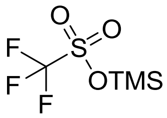

B3LYP /6-31G(d) 195.15 K Thermal correction to Gibbs Free Energy = 0.116775

Thermal correction to Enthalpy = 0.149748

M062x-D3/6-311+G(d,p) (IEFPCM, Dichloromethane) Energy = -1370.68324665

Standard orientation:

Center Number	Atomic Number	Atomic Type	Coordinates (Angstroms) X	Y	Z
1	16	0	-0.802227	0.876900	0.052162
2	8	0	-1.358180	1.844286	0.981622
3	8	0	-0.516189	1.225606	-1.336904
4	6	0	-1.966636	-0.583187	0.019653
5	8	0	0.451653	0.159635	0.718772
6	9	0	-3.108095	-0.203559	-0.552108
7	9	0	-2.205083	-1.007996	1.258169
8	9	0	-1.422814	-1.576139	-0.692272
9	14	0	2.026288	-0.192192	0.019650
10	6	0	2.871470	-1.041611	1.460297
11	1	0	3.895160	-1.329981	1.191527
12	1	0	2.926245	-0.381477	2.332674
13	1	0	2.334751	-1.949312	1.757222

14	6	0	1.787124	−1.325784	−1.454155
15	1	0	2.760181	−1.599873	−1.880579
16	1	0	1.271450	−2.250130	−1.173156
17	1	0	1.201608	−0.833009	−2.236695
18	6	0	2.824615	1.438360	−0.443520
19	1	0	3.840280	1.274838	−0.824882
20	1	0	2.248616	1.947998	−1.222691
21	1	0	2.895523	2.107595	0.421221

Supplementary Table 10.

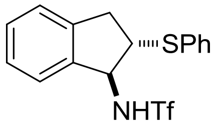

B3LYP /6-31G(d) 195.15 K Thermal correction to Gibbs Free Energy = 0.248669

Thermal correction to Enthalpy= 0.289426

M062x-D3/6-311+G(d,p) (IEFPCM, Dichloromethane) Energy = -1919.05972074

Standard orientation:

Center Number	Atomic Number	Atomic Type	Coordinates (Angstroms) X	Y	Z
1	6	0	3.286154	−3.292109	0.223607
2	6	0	2.136467	−2.568607	−0.086237
3	6	0	2.113891	−1.177373	0.068437
4	6	0	3.231599	−0.491263	0.538391
5	6	0	4.382798	−1.219030	0.853185
6	6	0	4.410584	−2.608150	0.694946
7	1	0	3.307780	−4.373330	0.110796
8	1	0	3.210364	0.585598	0.670067
9	1	0	5.261551	−0.700547	1.226812
10	1	0	5.311159	−3.161448	0.947443
11	6	0	0.758167	−0.628486	−0.353782
12	1	0	0.784148	−0.259536	−1.385009
13	6	0	−0.150932	−1.894148	−0.280899
14	1	0	−0.506336	−1.998753	0.750407
15	6	0	0.805880	−3.067030	−0.603671
16	7	0	0.282971	0.487424	0.466344
17	1	0	0.178746	0.365046	1.467979
18	16	0	−0.165886	1.970910	−0.077181
19	8	0	−0.543278	1.884857	−1.483189
20	8	0	−0.992761	2.586054	0.954233

21	6	0	1.427705	2.949799	-0.079174
22	9	0	2.288483	2.407809	-0.950310
23	9	0	1.183469	4.211342	-0.424655
24	9	0	1.979440	2.922449	1.141807
25	6	0	-2.905740	-1.188183	-0.346959
26	6	0	-3.390528	0.091384	-0.644187
27	6	0	-3.467430	-1.916338	0.711680
28	6	0	-4.419017	0.641517	0.124053
29	6	0	-4.484975	-1.355554	1.483405
30	6	0	-4.963637	-0.076414	1.189374
31	1	0	-4.784515	1.638346	-0.106111
32	1	0	-4.913115	-1.923233	2.305501
33	1	0	-5.760371	0.357092	1.787744
34	16	0	-1.629618	-1.925596	-1.378146
35	1	0	0.848454	-3.236458	-1.689674
36	1	0	0.472638	-4.005307	-0.148940
37	1	0	-3.112713	-2.921861	0.920429
38	1	0	-2.950665	0.655630	-1.459590

Supplementary Table 11.

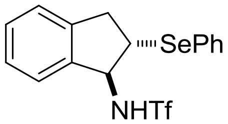

B3LYP /6-31G(d)   195.15 K   Thermal correction to Gibbs Free Energy = 0.247619

Thermal correction to Enthalpy = 0.289072

M062x-D3/6-311+G(d,p) (IEFPCM, Dichloromethane)   Energy = -3922.42239366

Standard orientation:

Center Number	Atomic Number	Atomic Type	Coordinates (Angstroms) X	Y	Z
1	6	0	1.232351	4.443218	-0.334501
2	6	0	0.768358	3.173840	0.004644
3	6	0	1.617736	2.066033	-0.109804
4	6	0	2.928119	2.199214	-0.558268
5	6	0	3.389021	3.473395	-0.901830
6	6	0	2.546965	4.585166	-0.790499
7	1	0	0.582465	5.311393	-0.255337
8	1	0	3.570288	1.326577	-0.643306
9	1	0	4.406513	3.601194	-1.261089
10	1	0	2.916889	5.569386	-1.065361
11	6	0	0.891841	0.812574	0.338752
12	1	0	1.150593	0.573387	1.376793

13	6	0	-0.597339	1.230838	0.238061
14	1	0	-0.939401	1.057066	-0.787387
15	6	0	-0.588881	2.747028	0.530570
16	7	0	1.234564	-0.378760	-0.448814
17	1	0	0.820119	-0.413471	-1.377773
18	16	0	1.239490	-1.895375	0.250305
19	8	0	1.299292	-1.763528	1.700739
20	8	0	0.306635	-2.782536	-0.443196
21	6	0	2.937751	-2.465222	-0.281205
22	9	0	3.870544	-1.647179	0.212781
23	9	0	3.146149	-3.699961	0.173139
24	9	0	3.013818	-2.462264	-1.616418
25	6	0	-3.155028	-0.232529	0.121870
26	6	0	-3.083897	-1.377741	-0.679553
27	6	0	-4.254047	0.626978	0.022172
28	6	0	-4.107894	-1.649787	-1.589434
29	6	0	-5.274754	0.349954	-0.888833
30	6	0	-5.201212	-0.787472	-1.695918
31	1	0	-4.051300	-2.538814	-2.212038
32	1	0	-6.127018	1.020095	-0.965380
33	1	0	-5.997864	-1.004192	-2.402676
34	1	0	-0.668273	2.924476	1.611863
35	1	0	-1.431105	3.265550	0.060192
36	1	0	-4.306123	1.504311	0.659755
37	1	0	-2.232510	-2.045686	-0.588824
38	34	0	-1.752459	0.126655	1.395417

Supplementary Table 12.

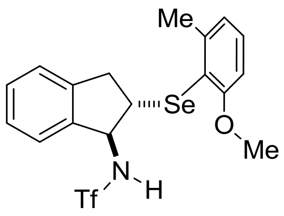

B3LYP /6-31G(d) 195.15 K Thermal correction to Gibbs Free Energy = 0.307538
Thermal correction to Enthalpy = 0.352082
M062x-D3/6-311+G(d,p) (IEFPCM, Dichloromethane) Energy = -4076.24679865
Standard orientation:

Center Number	Atomic Number	Atomic Type	Coordinates (Angstroms) X	Y	Z
1	6	0	1.641329	4.251526	-0.881857

2	6	0	1.050974	3.127639	−0.309221
3	6	0	1.805133	1.967274	−0.070793
4	6	0	3.161786	1.929529	−0.386541
5	6	0	3.749806	3.056843	−0.967897
6	6	0	2.997692	4.208612	−1.215206
7	1	0	1.055245	5.147222	−1.073533
8	1	0	3.757241	1.047995	−0.184894
9	1	0	4.804729	3.035634	−1.227743
10	1	0	3.470006	5.075738	−1.669329
11	6	0	0.921940	0.929360	0.606331
12	1	0	1.039726	1.008356	1.696751
13	6	0	−0.493773	1.404035	0.199047
14	1	0	−0.706590	1.018465	−0.802016
15	6	0	−0.380500	2.937735	0.146574
16	7	0	1.052793	−0.488588	0.191190
17	1	0	0.143913	−0.967232	0.241700
18	16	0	2.193831	−1.474585	0.896377
19	8	0	3.172837	−0.674130	1.627961
20	8	0	1.547041	−2.651808	1.479032
21	6	0	3.064672	−2.140362	−0.618799
22	9	0	3.639780	−1.156742	−1.313783
23	9	0	4.000050	−3.007423	−0.229273
24	9	0	2.177075	−2.767541	−1.398679
25	34	0	−1.904637	0.691730	1.388803
26	6	0	−3.047273	−0.114741	0.062602
27	6	0	−4.212303	0.547672	−0.364691
28	6	0	−2.740978	−1.400751	−0.431565
29	6	0	−5.055313	−0.093384	−1.283019
30	6	0	−3.594373	−2.023203	−1.349094
31	6	0	−4.748125	−1.361673	−1.765609
32	1	0	−5.957135	0.410642	−1.618910
33	1	0	−3.366800	−3.008026	−1.738951
34	1	0	−5.410680	−1.846520	−2.477548
35	8	0	−1.587215	−1.981953	0.019803
36	6	0	−1.320379	−3.344403	−0.318481
37	1	0	−0.415140	−3.606038	0.229298
38	1	0	−2.147023	−3.991292	−0.003727
39	1	0	−1.150076	−3.461567	−1.395771
40	6	0	−4.567981	1.927483	0.138912
41	1	0	−3.781125	2.652861	−0.093399
42	1	0	−4.687754	1.941171	1.227401
43	1	0	−5.502242	2.274236	−0.312500
44	1	0	−0.543892	3.371392	1.143404
45	1	0	−1.123052	3.384976	−0.522793

——

Supplementary Table 13.

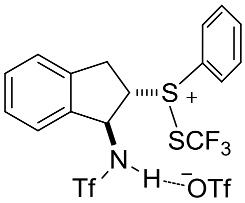

B3LYP /6-31G(d) 195.15 K Thermal correction to Gibbs Free Energy = 0.284688
Thermal correction to Enthalpy = 0.342942
M062x-D3/6-311+G(d,p) (IEFPCM, Dichloromethane) Energy = -3616.27797007
Standard orientation:

Center Number	Atomic Number	Atomic Type	Coordinates (Angstroms) X	Y	Z
1	16	0	1.348564	1.849427	-0.920283
2	6	0	1.715864	3.589782	-0.403239
3	9	0	2.950833	3.708326	0.117432
4	9	0	1.639397	4.318220	-1.517985
5	9	0	0.853029	4.058660	0.497858
6	6	0	-0.910485	-2.946822	3.418697
7	6	0	-0.608609	-1.816488	2.663491
8	6	0	-1.612098	-1.129685	1.976028
9	6	0	-2.934491	-1.560470	2.019957
10	6	0	-3.239482	-2.693997	2.777639
11	6	0	-2.237782	-3.379837	3.472853
12	1	0	-0.130216	-3.491102	3.944310
13	1	0	-3.707377	-1.034236	1.471525
14	1	0	-4.264690	-3.050475	2.820222
15	1	0	-2.491402	-4.264219	4.050522
16	6	0	-1.037522	0.085496	1.255096
17	1	0	-1.136748	0.976407	1.887166
18	6	0	0.453257	-0.354992	1.156458
19	1	0	0.586642	-0.960639	0.257859
20	6	0	0.741445	-1.166439	2.439908
21	7	0	-1.663330	0.393226	-0.029753
22	1	0	-1.270107	-0.075120	-0.861868
23	16	0	-2.261252	1.905189	-0.349535
24	8	0	-1.896991	2.813201	0.740676
25	8	0	-2.042615	2.224588	-1.753537
26	6	0	-4.104416	1.648191	-0.175995
27	9	0	-4.391546	1.294388	1.086100
28	9	0	-4.731368	2.786677	-0.463945

29	9	0	-4.514745	0.686645	-1.000874
30	1	0	1.013549	-0.513951	3.283521
31	1	0	1.556316	-1.880749	2.295476
32	6	0	3.260881	0.217762	0.962237
33	6	0	3.550326	-0.779608	0.025740
34	6	0	4.189580	0.646303	1.919112
35	6	0	4.812668	-1.370250	0.074514
36	6	0	5.444487	0.038861	1.946737
37	6	0	5.752550	-0.966725	1.027495
38	1	0	5.050977	-2.146548	-0.645816
39	1	0	6.178520	0.356219	2.681086
40	1	0	6.732920	-1.433933	1.050047
41	8	0	0.342853	-1.817941	-3.980570
42	16	0	0.693189	-1.888556	-2.562535
43	8	0	2.049705	-2.360628	-2.206339
44	8	0	0.289195	-0.678391	-1.745242
45	6	0	-0.434018	-3.180110	-1.834725
46	9	0	-1.719482	-2.821896	-1.976533
47	9	0	-0.188787	-3.310225	-0.509868
48	9	0	-0.251698	-4.369683	-2.411857
49	1	0	2.841827	-1.102530	-0.735893
50	1	0	3.940288	1.435344	2.622241
51	16	0	1.668374	1.042720	1.015663

Supplementary Table 14.

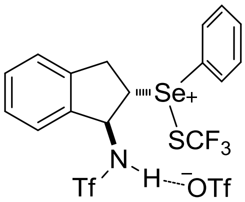

B3LYP /6-31G(d) 195.15 K Thermal correction to Gibbs Free Energy = 0.283966

Thermal correction to Enthalpy= 0.342123

M062x-D3/6-311+G(d,p) (IEFPCM, Dichloromethane) Energy = -5619.65583211

Standard orientation:

Center Number	Atomic Number	Atomic Type	Coordinates (Angstroms) X	Y	Z
1	16	0	1.304675	-1.725424	1.238692
2	6	0	1.702648	-3.480061	0.819326
3	9	0	2.803104	-3.546776	0.033822
4	9	0	1.952086	-4.107048	1.970059
5	9	0	0.725138	-4.112205	0.169662

6	34	0	1.211806	−1.180211	−0.927659
7	6	0	−1.048939	3.322492	−3.009959
8	6	0	−0.829563	2.125307	−2.333516
9	6	0	−1.883882	1.477728	−1.684957
10	6	0	−3.171935	2.001117	−1.693093
11	6	0	−3.392576	3.202335	−2.371808
12	6	0	−2.340966	3.855806	−3.024148
13	1	0	−0.233010	3.841191	−3.506539
14	1	0	−3.977282	1.490214	−1.174174
15	1	0	−4.387785	3.637374	−2.385892
16	1	0	−2.528161	4.793919	−3.539100
17	6	0	−1.397457	0.183454	−1.057218
18	1	0	−1.562135	−0.640718	−1.760497
19	6	0	0.116676	0.482944	−0.936367
20	1	0	0.363644	0.982106	0.001121
21	6	0	0.464420	1.350880	−2.165530
22	7	0	−2.057959	−0.178838	0.189293
23	16	0	−2.404110	−1.759755	0.482922
24	8	0	−1.640021	−2.596364	−0.455090
25	8	0	−2.430210	−2.005716	1.916720
26	6	0	−4.170842	−1.872442	−0.116297
27	9	0	−4.219039	−1.498707	−1.404934
28	9	0	−4.603222	−3.126459	−0.004175
29	9	0	−4.949663	−1.062480	0.598664
30	1	0	0.680089	0.741482	−3.057261
31	1	0	1.335196	1.985505	−1.982168
32	6	0	2.973901	−0.427697	−1.186976
33	6	0	3.494731	0.550821	−0.336144
34	6	0	3.691462	−0.931424	−2.274323
35	6	0	4.774588	1.035942	−0.604276
36	6	0	4.969608	−0.429943	−2.526409
37	6	0	5.507426	0.550885	−1.692153
38	1	0	5.200712	1.795051	0.044801
39	1	0	5.541410	−0.812735	−3.366347
40	1	0	6.504342	0.935990	−1.886238
41	1	0	−1.855445	0.404630	1.026523
42	8	0	−1.194162	1.645035	2.123180
43	16	0	0.211952	1.863866	2.562883
44	8	0	0.447388	1.991141	3.999343
45	8	0	1.182624	0.987086	1.818472
46	6	0	0.612339	3.533205	1.839575
47	9	0	0.441004	3.507992	0.496782
48	9	0	1.890346	3.860464	2.080306
49	9	0	−0.176523	4.484655	2.342832

50	1	0	2.928968	0.918840	0.516319
51	1	0	3.269635	-1.705270	-2.909672

Supplementary Table 15.

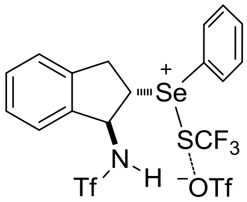

B3LYP /6-31G(d) 195.15 K Thermal correction to Gibbs Free Energy = 0.282741

Thermal correction to Enthalpy = 0.341643

M062x-D3/6-311+G(d,p) (IEFPCM, Dichloromethane) Energy = -5619.62535288

Standard orientation:

Center Number	Atomic Number	Atomic Type	Coordinates (Angstroms) X	Y	Z
1	6	0	-0.231111	3.389467	2.528169
2	6	0	-0.655338	2.224849	1.889507
3	6	0	-0.671323	2.153572	0.495712
4	6	0	-0.256713	3.233650	-0.284048
5	6	0	0.165993	4.399458	0.354725
6	6	0	0.177443	4.475527	1.752381
7	1	0	-0.217409	3.453704	3.613088
8	1	0	-0.251566	3.171885	-1.369030
9	1	0	0.494932	5.249633	-0.235610
10	1	0	0.513020	5.387434	2.238183
11	6	0	-1.137474	0.794081	0.011899
12	1	0	-0.369285	0.318679	-0.606062
13	6	0	-1.384718	-0.008000	1.321667
14	1	0	-2.378878	-0.446315	1.335876
15	6	0	-1.150025	0.945242	2.521861
16	7	0	-2.313747	0.772730	-0.898561
17	1	0	-2.101421	0.993479	-1.867779
18	16	0	-3.808023	1.388020	-0.488184
19	8	0	-3.816166	1.757182	0.923959
20	8	0	-4.282947	2.259152	-1.557041
21	6	0	-4.859431	-0.156524	-0.605852
22	9	0	-4.455556	-1.057540	0.304025
23	9	0	-6.127792	0.164875	-0.364099
24	9	0	-4.754780	-0.689828	-1.822660
25	6	0	-0.665508	-2.491958	-0.149653
26	6	0	0.181356	-2.482091	-1.259994

27	6	0	-1.881435	-3.183627	-0.177038
28	6	0	-0.213127	-3.162307	-2.415499
29	6	0	-2.267105	-3.849617	-1.339402
30	6	0	-1.432533	-3.838611	-2.459541
31	1	0	0.444241	-3.161217	-3.280064
32	1	0	-3.213425	-4.382181	-1.364553
33	1	0	-1.730156	-4.364534	-3.362254
34	1	0	-0.432682	0.536512	3.244660
35	1	0	-2.092405	1.102757	3.058300
36	1	0	-2.523437	-3.203004	0.698924
37	1	0	1.138201	-1.971492	-1.237730
38	16	0	1.905939	-0.408221	0.889840
39	6	0	2.774234	-1.869818	1.658902
40	8	0	3.594428	0.497281	0.434978
41	16	0	4.161309	0.303835	-1.021226
42	8	0	5.568510	0.677177	-1.053643
43	8	0	3.698169	-0.941392	-1.636882
44	6	0	3.242889	1.659750	-1.917172
45	9	0	3.497981	2.849406	-1.376073
46	9	0	3.599911	1.672861	-3.202726
47	9	0	1.913666	1.432027	-1.846956
48	9	0	2.317593	-3.027053	1.128940
49	9	0	2.512843	-1.924659	2.978925
50	9	0	4.084845	-1.821218	1.489309
51	34	0	-0.207115	-1.600468	1.508476

Supplementary Table 16.

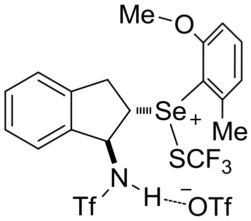

B3LYP /6-31G(d) 195.15 K Thermal correction to Gibbs Free Energy = 0.342147

Thermal correction to Enthalpy = 0.404761

M062x-D3/6-311+G(d,p) (IEFPCM, Dichloromethane) Energy = -5773.47408706

Standard orientation:

Center Number	Atomic Number	Atomic Type	Coordinates (Angstroms) X	Y	Z
1	16	0	-1.169579	0.232975	-1.990562
2	6	0	-2.455480	-0.882892	-2.699928

3	9	0	−3.675950	−0.615177	−2.182844
4	9	0	−2.485716	−0.659134	−4.015630
5	9	0	−2.216352	−2.176429	−2.474416
6	34	0	−1.651318	−0.474543	0.080864
7	6	0	1.308388	0.372289	4.565039
8	6	0	0.879708	0.009749	3.291336
9	6	0	1.710979	−0.735484	2.452265
10	6	0	2.978761	−1.137367	2.856439
11	6	0	3.409735	−0.773647	4.134714
12	6	0	2.582237	−0.026510	4.980207
13	1	0	0.675385	0.965123	5.220198
14	1	0	3.615638	−1.707654	2.187225
15	1	0	4.400406	−1.065329	4.471534
16	1	0	2.937926	0.257002	5.966847
17	6	0	1.000229	−1.033457	1.144562
18	1	0	0.483234	−1.996322	1.231804
19	6	0	−0.057558	0.106738	1.126494
20	1	0	0.328445	1.020600	0.682662
21	6	0	−0.440388	0.312674	2.608326
22	7	0	1.862653	−1.122446	−0.021843
23	1	0	2.409907	−0.284448	−0.317443
24	16	0	1.600936	−2.281640	−1.150687
25	8	0	0.241238	−2.812366	−0.971655
26	8	0	2.118815	−1.853968	−2.440626
27	6	0	2.719848	−3.658169	−0.561904
28	9	0	2.393886	−3.979139	0.701048
29	9	0	2.568964	−4.729063	−1.339537
30	9	0	3.991307	−3.259256	−0.590344
31	1	0	−1.220926	−0.394583	2.930235
32	1	0	−0.813753	1.321843	2.800891
33	6	0	−3.045811	0.734158	0.668742
34	6	0	−2.989069	2.140535	0.662048
35	6	0	−4.200721	0.036606	1.094850
36	6	0	−4.136116	2.822579	1.095318
37	6	0	−5.324143	0.749685	1.516322
38	6	0	−5.277193	2.142647	1.509854
39	1	0	−4.123512	3.907965	1.098706
40	1	0	−6.217436	0.233156	1.845721
41	1	0	−6.148386	2.703365	1.836630
42	8	0	0.925930	2.037940	−1.450593
43	16	0	2.066731	2.389995	−0.560243
44	8	0	1.647336	2.935961	0.751211
45	8	0	3.116719	1.332584	−0.489308
46	6	0	2.910825	3.801462	−1.428289

47	9	0	3.319324	3.428167	-2.646169
48	9	0	3.971671	4.214886	-0.724655
49	9	0	2.058968	4.829191	-1.560497
50	6	0	-1.788713	2.936360	0.223183
51	1	0	-0.928360	2.817952	0.890047
52	1	0	-1.433558	2.666039	-0.773883
53	1	0	-2.033026	4.001497	0.209212
54	8	0	-4.115819	-1.319018	1.073733
55	6	0	-5.274944	-2.092988	1.378571
56	1	0	-6.100287	-1.848644	0.700336
57	1	0	-4.977524	-3.132108	1.235461
58	1	0	-5.589436	-1.940417	2.417606

Supplementary Table 17.

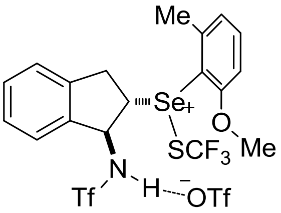

B3LYP /6-31G(d) 195.15 K Thermal correction to Gibbs Free Energy = 0.343032

Thermal correction to Enthalpy = 0.404568

M062x-D3/6-311+G(d,p) (IEFPCM, Dichloromethane) Energy = -5773.48237500

Standard orientation:

Center Number	Atomic Number	Atomic Type	Coordinates (Angstroms) X	Y	Z
1	16	0	1.164547	1.566753	0.849347
2	6	0	1.886291	1.640133	2.542307
3	9	0	3.162263	1.182109	2.546264
4	9	0	1.889161	2.921079	2.915343
5	9	0	1.218532	0.914683	3.440657
6	34	0	1.510424	-0.642624	0.731778
7	6	0	-0.741474	-3.722081	-3.184014
8	6	0	-0.520257	-2.893260	-2.087208
9	6	0	-1.586900	-2.501236	-1.273270
10	6	0	-2.887515	-2.920388	-1.528662
11	6	0	-3.109366	-3.752071	-2.629260
12	6	0	-2.046301	-4.149051	-3.448099
13	1	0	0.078164	-4.023108	-3.831533
14	1	0	-3.705460	-2.595374	-0.892835
15	1	0	-4.117787	-4.085283	-2.857105

16	1	0	−2.238253	−4.787694	−4.305802
17	6	0	−1.082080	−1.633115	−0.135072
18	1	0	−0.868468	−2.267724	0.733455
19	6	0	0.247460	−1.116853	−0.747779
20	1	0	0.097371	−0.202968	−1.332288
21	6	0	0.781565	−2.291161	−1.590389
22	7	0	−2.003020	−0.598593	0.306044
23	1	0	−2.273630	0.154371	−0.369639
24	16	0	−2.154253	−0.253150	1.898747
25	8	0	−0.993448	−0.801686	2.617012
26	8	0	−2.608083	1.118525	2.069239
27	6	0	−3.577149	−1.352889	2.406898
28	9	0	−3.263089	−2.629788	2.132363
29	9	0	−3.801393	−1.227161	3.713685
30	9	0	−4.677032	−1.024821	1.729793
31	1	0	1.341700	−3.016156	−0.979337
32	1	0	1.446714	−1.951871	−2.388149
33	6	0	3.264640	−0.782164	−0.040706
34	6	0	3.558440	−0.142315	−1.262710
35	6	0	4.236373	−1.494052	0.686860
36	6	0	4.858079	−0.222206	−1.777123
37	6	0	5.521980	−1.564400	0.141668
38	6	0	5.821789	−0.935302	−1.068037
39	1	0	5.109110	0.262508	−2.713047
40	1	0	6.293557	−2.111664	0.674394
41	1	0	6.829857	−1.000242	−1.467867
42	8	0	−0.106012	1.748695	−1.803438
43	16	0	−1.496404	2.142130	−2.205638
44	8	0	−1.643307	2.582960	−3.595260
45	8	0	−2.526199	1.176174	−1.718683
46	6	0	−1.796905	3.658256	−1.169655
47	9	0	−1.649152	3.367904	0.135096
48	9	0	−3.032592	4.130639	−1.362116
49	9	0	−0.915917	4.620049	−1.486111
50	8	0	2.531607	0.502052	−1.837903
51	6	0	2.738789	1.309186	−3.006626
52	1	0	3.044066	0.686120	−3.854914
53	1	0	1.769193	1.769342	−3.189688
54	1	0	3.497459	2.074348	−2.810250
55	6	0	3.928627	−2.151132	2.014179
56	1	0	3.618312	−1.411377	2.760434
57	1	0	3.120849	−2.887588	1.931474
58	1	0	4.811693	−2.667399	2.398309

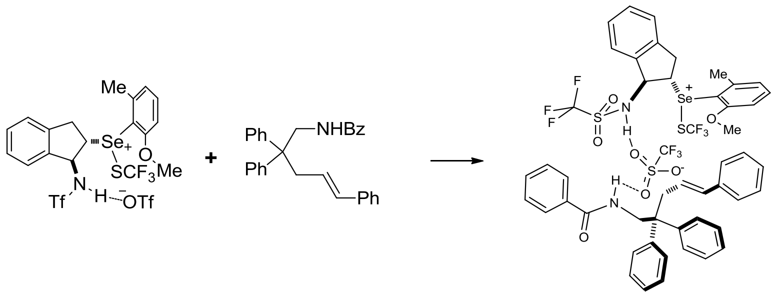

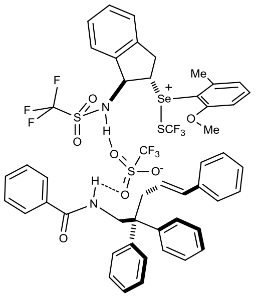

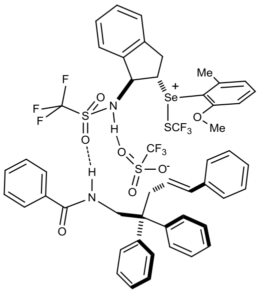

int-II-RSS-a

$\Delta G_{195.15\ K}$ = -10.6 kcal/mol

$\Delta\Delta G_{195.15\ K}$ = 0.0 kcal/mol

int-II-RSS-b

$\Delta G_{195.15\ K}$ = -8.3 kcal/mol

$\Delta\Delta G_{195.15\ K}$ = 2.3 kcal/mol

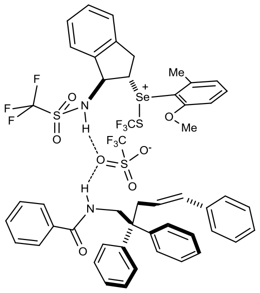

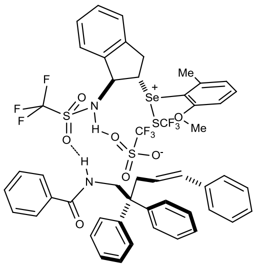

int-II-RRR-a

$\Delta G_{195.15\ K}$ = - 6.3 kcal/mol

$\Delta\Delta G_{195.15\ K}$ = 4.3 kcal/mol

int-II-RRR-b

$\Delta G_{195.15\ K}$ = -6.0 kcal/mol

$\Delta\Delta G_{195.15\ K}$ = 4.6 kcal/mol

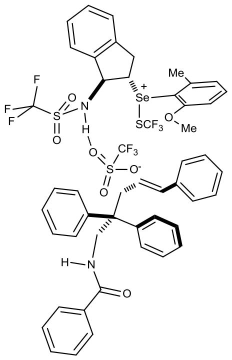

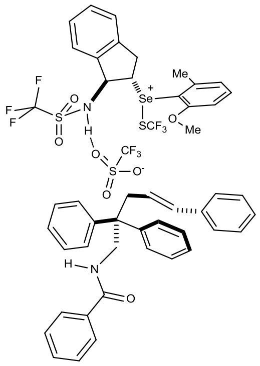

int-II-SSS

$\Delta G_{195.15\ K}$ = - 5.0 kcal/mol

$\Delta\Delta G_{195.15\ K}$ = 5.6 kcal/mol

int-II-SRR

$\Delta G_{195.15\ K}$ = -3.8 kcal/mol

$\Delta\Delta G_{195.15\ K}$ = 6.9 kcal/mol

Supplementary Figure 179. DFT calculations for the int-II of 1a; ΔG related to **int-I**.

Supplementary Table 18.

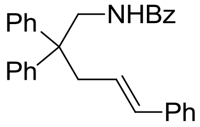

B3LYP /6-31G(d) 195.15 K Thermal correction to Gibbs Free Energy = 0.457629

Thermal correction to Enthalpy = 0.504580

M062x-D3/6-311+G(d, p) (IEFPCM, Dichloromethane) Energy = -1289.24198790

Standard orientation:

Center Number	Atomic Number	Atomic Type	Coordinates (Angstroms) X	Y	Z
1	6	0	3.669349	-0.547170	-1.085413
2	6	0	2.677796	0.357206	-1.098670
3	6	0	1.323883	0.108711	-1.698883
4	6	0	0.081241	0.224672	-0.738335
5	1	0	3.466041	-1.532712	-1.506114
6	1	0	2.839964	1.344903	-0.670376
7	1	0	1.172705	0.803966	-2.538356
8	1	0	1.298140	-0.897661	-2.132137
9	6	0	5.028748	-0.379338	-0.549399
10	6	0	5.892340	-1.488929	-0.535334
11	6	0	5.518477	0.840403	-0.044761
12	6	0	7.188607	-1.392097	-0.031673
13	1	0	5.534467	-2.440437	-0.922213
14	6	0	6.812023	0.938820	0.458799
15	1	0	4.883687	1.721864	-0.050332
16	6	0	7.655141	-0.176607	0.469376
17	1	0	7.833679	-2.266911	-0.031407
18	1	0	7.167210	1.891771	0.842794
19	1	0	8.665221	-0.095809	0.861799
20	6	0	-1.155022	0.123294	-1.692365
21	1	0	-1.064012	0.936990	-2.424171
22	6	0	0.162403	-0.934306	0.271470
23	6	0	-0.551116	-2.130168	0.102245
24	6	0	1.048931	-0.850794	1.359363
25	6	0	-0.387778	-3.195919	0.992007
26	1	0	-1.254469	-2.250616	-0.714474
27	6	0	1.212828	-1.913174	2.246776
28	1	0	1.617687	0.059402	1.518401
29	6	0	0.492846	-3.095287	2.067039
30	1	0	-0.960490	-4.106829	0.837643
31	1	0	1.907543	-1.815210	3.077041

32	1	0	0.616678	-3.925278	2.757812
33	6	0	-0.017274	1.603753	-0.050739
34	6	0	0.436332	2.773483	-0.683256
35	6	0	-0.686088	1.752688	1.177242
36	6	0	0.251015	4.031539	-0.107552
37	1	0	0.942156	2.713998	-1.641719
38	6	0	-0.879859	3.010554	1.752519
39	1	0	-1.045000	0.870997	1.698222
40	6	0	-0.408258	4.157867	1.115130
41	1	0	0.623370	4.914324	-0.620940
42	1	0	-1.397244	3.088027	2.705355
43	1	0	-0.551888	5.136682	1.564504
44	1	0	-1.148524	-0.815051	-2.249253
45	7	0	-2.446800	0.211514	-1.038665
46	1	0	-2.591735	0.976341	-0.394791
47	6	0	-3.431397	-0.722731	-1.198060
48	8	0	-3.272551	-1.746337	-1.864513
49	6	0	-4.731125	-0.444741	-0.493180
50	6	0	-5.152415	0.838281	-0.115983
51	6	0	-5.565228	-1.540492	-0.233679
52	6	0	-6.377279	1.017495	0.527402
53	1	0	-4.548333	1.710840	-0.350815
54	6	0	-6.784058	-1.362193	0.416004
55	1	0	-5.235559	-2.523103	-0.554202
56	6	0	-7.192006	-0.082430	0.800814
57	1	0	-6.698060	2.017353	0.807031
58	1	0	-7.418683	-2.220399	0.619890
59	1	0	-8.144866	0.058311	1.304123

Supplementary Table 19.

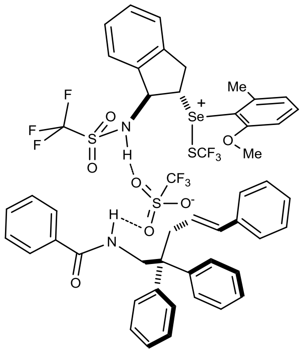

int-II-RSS-a

B3LYP /6-31G(d) 195.15 K Thermal correction to Gibbs Free Energy = 0.816044

Thermal correction to Enthalpy = 0.910983

M062x-D3/6-311+G(d,p) (IEFPCM, Dichloromethane) Energy = -7062.75666549

Standard orientation:

Center Number	Atomic Number	Atomic Type	Coordinates (Angstroms) X	Y	Z
1	6	0	-1.327823	-3.243337	0.423059
2	6	0	-1.697421	-1.941235	0.426604
3	6	0	-2.552110	-1.272105	-0.615641
4	6	0	-4.089944	-1.261555	-0.289201
5	16	0	1.056252	-1.480770	-0.803660
6	6	0	0.835391	-2.278350	-2.457408
7	9	0	1.864367	-3.125369	-2.715098
8	9	0	-0.296966	-2.982075	-2.474455
9	9	0	0.812995	-1.398338	-3.464280
10	1	0	-1.620280	-3.863452	-0.423020
11	1	0	-1.414897	-1.315513	1.269283
12	1	0	-2.216769	-0.235315	-0.709732
13	1	0	-2.413948	-1.751953	-1.589620
14	34	0	3.055377	-0.634421	-1.535495
15	6	0	5.858916	3.213359	1.137215
16	6	0	4.890547	2.508600	0.426333
17	6	0	3.774421	3.171357	-0.090465
18	6	0	3.597761	4.539743	0.083645
19	6	0	4.568681	5.246596	0.797696
20	6	0	5.688391	4.588965	1.319309
21	1	0	6.726104	2.706188	1.552519
22	1	0	2.721702	5.038047	-0.320507
23	1	0	4.449541	6.314679	0.955582
24	1	0	6.429728	5.152099	1.879381
25	6	0	2.890837	2.196901	-0.847515
26	1	0	3.151433	2.222749	-1.912116
27	6	0	3.339757	0.845966	-0.224285
28	1	0	2.751428	0.601539	0.662962
29	6	0	4.839552	1.031267	0.088375
30	7	0	1.466523	2.479084	-0.751590
31	16	0	0.482159	2.232559	-2.037153
32	8	0	1.216150	1.466283	-3.052186
33	8	0	-0.850076	1.855954	-1.578201
34	6	0	0.322226	3.957323	-2.739351
35	9	0	1.549363	4.434746	-2.998728
36	9	0	-0.386302	3.924865	-3.865040
37	9	0	-0.273944	4.752593	-1.852201
38	1	0	5.470837	0.809661	-0.785633
39	1	0	5.168523	0.378915	0.900767

40	6	0	4.295646	−1.993951	−0.979954
41	6	0	4.334580	−2.412349	0.366084
42	6	0	5.096925	−2.588409	−1.971762
43	6	0	5.197284	−3.452206	0.731077
44	6	0	5.956678	−3.617315	−1.573751
45	6	0	5.998699	−4.040416	−0.244847
46	1	0	5.239466	−3.796331	1.757336
47	1	0	6.591320	−4.093180	−2.315063
48	1	0	6.669170	−4.846874	0.038614
49	8	0	3.520235	−1.749165	1.207533
50	6	0	3.388682	−2.180734	2.569274
51	1	0	2.640592	−1.517370	2.998901
52	1	0	3.036114	−3.215496	2.611501
53	1	0	4.343929	−2.079918	3.097817
54	6	0	5.038670	−2.156781	−3.420367
55	1	0	4.038146	−2.306076	−3.840400
56	1	0	5.286480	−1.095750	−3.542179
57	1	0	5.745812	−2.735477	−4.019702
58	6	0	−0.606515	−3.950561	1.490169
59	6	0	−0.239826	−5.293100	1.283407
60	6	0	−0.287371	−3.360294	2.729931
61	6	0	0.422499	−6.023340	2.268705
62	1	0	−0.486851	−5.766896	0.336000
63	6	0	0.369220	−4.092358	3.715684
64	1	0	−0.561517	−2.329397	2.929612
65	6	0	0.729782	−5.425811	3.492284
66	1	0	0.690093	−7.060631	2.084211
67	1	0	0.591637	−3.620984	4.669855
68	1	0	1.233292	−5.994967	4.269510
69	6	0	−4.323565	−0.574872	1.095253
70	1	0	−5.388959	−0.591934	1.335688
71	6	0	−4.581555	−2.725116	−0.270394
72	6	0	−4.616836	−3.444477	−1.478499
73	6	0	−4.968932	−3.399810	0.894893
74	6	0	−5.011475	−4.779258	−1.520946
75	1	0	−4.348602	−2.939960	−2.402860
76	6	0	−5.372590	−4.738799	0.856375
77	1	0	−4.966140	−2.892392	1.852824
78	6	0	−5.392986	−5.436692	−0.348190
79	1	0	−5.030829	−5.304142	−2.472992
80	1	0	−5.670737	−5.231712	1.778318
81	1	0	−5.707325	−6.476755	−0.377245
82	6	0	−4.874763	−0.479843	−1.366893
83	6	0	−6.277568	−0.562685	−1.373179

84	6	0	−4.266367	0.334791	−2.330046
85	6	0	−7.041688	0.132957	−2.305025
86	1	0	−6.778461	−1.183957	−0.636557
87	6	0	−5.031432	1.033073	−3.270127
88	1	0	−3.189264	0.450143	−2.361475
89	6	0	−6.420120	0.935084	−3.264736
90	1	0	−8.125237	0.051358	−2.278416
91	1	0	−4.529933	1.659053	−4.004085
92	1	0	−7.013842	1.480127	−3.994148
93	1	0	−3.786037	−1.128657	1.871764
94	7	0	−3.892087	0.810567	1.154032
95	1	0	−2.952569	0.981780	1.498368
96	6	0	−4.808581	1.830056	1.225627
97	8	0	−6.022126	1.637712	1.171495
98	6	0	−4.246307	3.218408	1.374257
99	6	0	−2.916370	3.550486	1.083161
100	6	0	−5.124088	4.222768	1.804706
101	6	0	−2.470368	4.863370	1.237794
102	1	0	−2.224475	2.801096	0.715843
103	6	0	−4.676709	5.531939	1.962546
104	1	0	−6.154834	3.949799	2.005588
105	6	0	−3.346274	5.855360	1.680819
106	1	0	−1.436061	5.103965	1.008090
107	1	0	−5.364333	6.301882	2.303554
108	1	0	−2.996223	6.877590	1.802794
109	1	0	1.007638	2.468325	0.182311
110	8	0	0.390500	2.407040	1.828486
111	16	0	0.209763	1.047259	2.408626
112	8	0	−1.191327	0.597840	2.550782
113	8	0	1.137229	0.029615	1.837422
114	6	0	0.816500	1.216002	4.159430
115	9	0	2.064534	1.708015	4.171483
116	9	0	0.840788	0.002400	4.742454
117	9	0	0.027768	2.019850	4.872758

Supplementary Table 20.

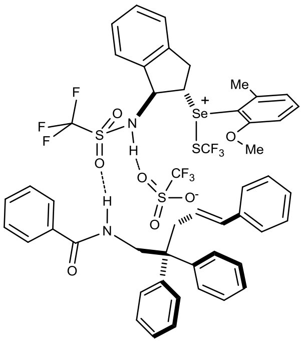

int-II-RSS-b

B3LYP /6-31G(d) 195.15 K Thermal correction to Gibbs Free Energy = 0.817625

Thermal correction to Enthalpy = 0.911001

M062x-D3/6-311+G(d,p) (IEFPCM, Dichloromethane) Energy = -7062.75463737

Standard orientation:

Center Number	Atomic Number	Atomic Type	Coordinates (Angstroms) X	Y	Z
1	6	0	-0.726876	-3.399233	-0.030091
2	6	0	-1.272229	-2.166004	0.121208
3	6	0	-2.331854	-1.602990	-0.787228
4	6	0	-3.784678	-1.633082	-0.186749
5	16	0	1.187868	-1.155785	-1.138938
6	6	0	0.543801	-1.410410	-2.861051
7	9	0	1.543058	-1.392861	-3.780047
8	9	0	-0.039563	-2.612820	-2.934515
9	9	0	-0.351089	-0.490387	-3.227298
10	1	0	-1.003290	-3.974781	-0.913023
11	1	0	-1.006013	-1.573278	0.993580
12	1	0	-2.086778	-0.562142	-1.028639
13	1	0	-2.340131	-2.165280	-1.726359
14	34	0	2.925592	0.186449	-1.850670
15	6	0	5.731738	3.430491	1.512491
16	6	0	4.731364	2.825655	0.755437
17	6	0	3.480484	3.434923	0.627440
18	6	0	3.197230	4.648350	1.245129
19	6	0	4.200553	5.254007	2.005741
20	6	0	5.456185	4.650532	2.137060
21	1	0	6.705282	2.960690	1.627429
22	1	0	2.215823	5.102526	1.145458
23	1	0	4.000902	6.197020	2.506482
24	1	0	6.222666	5.130329	2.739300
25	6	0	2.584115	2.604952	-0.272425

26	1	0	2.648509	2.985738	-1.298810
27	6	0	3.270899	1.213097	-0.177505
28	1	0	2.868747	0.631254	0.656071
29	6	0	4.774879	1.521236	-0.016355
30	7	0	1.176194	2.630074	0.106388
31	16	0	0.012374	2.559645	-1.035559
32	8	0	0.607968	2.160232	-2.317430
33	8	0	-1.175861	1.906998	-0.492411
34	6	0	-0.446199	4.356676	-1.264349
35	9	0	0.647925	5.041719	-1.624783
36	9	0	-1.368652	4.462194	-2.223898
37	9	0	-0.921667	4.856557	-0.127731
38	1	0	5.271289	1.659597	-0.989013
39	1	0	5.299241	0.715655	0.503756
40	6	0	4.352477	-1.096172	-1.948047
41	6	0	4.633394	-1.919469	-0.838098
42	6	0	5.035022	-1.230843	-3.170655
43	6	0	5.611433	-2.913569	-0.956491
44	6	0	6.015960	-2.224827	-3.254087
45	6	0	6.290683	-3.052629	-2.164918
46	1	0	5.837383	-3.563979	-0.120019
47	1	0	6.563113	-2.351366	-4.183315
48	1	0	7.052075	-3.822158	-2.256808
49	8	0	3.921406	-1.659269	0.272642
50	6	0	3.977103	-2.555926	1.393242
51	1	0	3.256858	-2.153887	2.102737
52	1	0	3.681034	-3.564173	1.088940
53	1	0	4.984206	-2.566576	1.826054
54	6	0	4.733448	-0.351786	-4.363743
55	1	0	3.701743	-0.480639	-4.708175
56	1	0	4.864996	0.711126	-4.129854
57	1	0	5.398954	-0.595582	-5.195537
58	6	0	0.163174	-4.096691	0.905540
59	6	0	0.792703	-5.284286	0.487369
60	6	0	0.383951	-3.654399	2.225271
61	6	0	1.621128	-6.003406	1.347735
62	1	0	0.621157	-5.646594	-0.523967
63	6	0	1.211217	-4.374168	3.083539
64	1	0	-0.100016	-2.753020	2.585097
65	6	0	1.833490	-5.550202	2.652166
66	1	0	2.090153	-6.922134	1.004386
67	1	0	1.364900	-4.012725	4.096663
68	1	0	2.468270	-6.114541	3.330713
69	6	0	-3.861788	-0.690706	1.062991

70	1	0	−4.766713	−0.910614	1.630937
71	6	0	−4.100282	−3.091600	0.207858
72	6	0	−4.386029	−4.031457	−0.796827
73	6	0	−4.057371	−3.547879	1.532553
74	6	0	−4.623570	−5.370340	−0.491929
75	1	0	−4.441421	−3.703676	−1.831266
76	6	0	−4.300829	−4.888968	1.842774
77	1	0	−3.830235	−2.864262	2.343085
78	6	0	−4.584437	−5.807064	0.834137
79	1	0	−4.848608	−6.071621	−1.291851
80	1	0	−4.264878	−5.210737	2.880416
81	1	0	−4.775312	−6.849340	1.076103
82	6	0	−4.813283	−1.147768	−1.230790
83	6	0	−6.180983	−1.217763	−0.916347
84	6	0	−4.457554	−0.621184	−2.479541
85	6	0	−7.152825	−0.781796	−1.813063
86	1	0	−6.488867	−1.610886	0.047202
87	6	0	−5.431695	−0.186527	−3.384089
88	1	0	−3.415575	−0.537749	−2.768861
89	6	0	−6.782902	−0.265150	−3.056850
90	1	0	−8.201968	−0.842574	−1.536080
91	1	0	−5.124504	0.216501	−4.346119
92	1	0	−7.540123	0.074765	−3.758629
93	1	0	−3.002892	−0.858351	1.720258
94	7	0	−3.937596	0.726636	0.746221
95	1	0	−3.082522	1.218141	0.520130
96	6	0	−5.053391	1.452052	1.074828
97	8	0	−6.044734	0.944479	1.598661
98	6	0	−5.020893	2.924341	0.765864
99	6	0	−4.228481	3.488142	−0.242284
100	6	0	−5.873379	3.751749	1.509733
101	6	0	−4.278151	4.861261	−0.489182
102	1	0	−3.593522	2.856777	−0.854871
103	6	0	−5.914051	5.122719	1.269907
104	1	0	−6.499393	3.293445	2.268098
105	6	0	−5.114876	5.681244	0.268875
106	1	0	−3.664881	5.287143	−1.277626
107	1	0	−6.572087	5.756255	1.859065
108	1	0	−5.150133	6.750573	0.075812
109	1	0	0.901253	2.306166	1.062100
110	8	0	0.627870	1.732575	2.672175
111	16	0	0.680176	0.252349	2.842334
112	8	0	−0.623567	−0.422928	2.983110
113	8	0	1.636025	−0.410765	1.907919

114	6	0	1.500035	0.026023	4.497460
115	9	0	2.687390	0.649318	4.522892
116	9	0	1.714263	-1.285693	4.719156
117	9	0	0.741035	0.506180	5.484983

Supplementary Table 21.

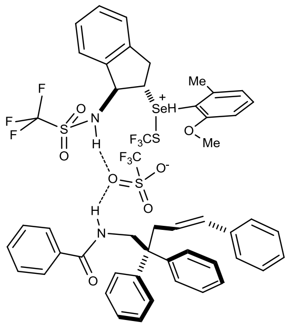

int-II-RRR-a

B3LYP /6-31G(d) 195.15 K Thermal correction to Gibbs Free Energy = 0.81685

Thermal correction to Enthalpy = 0.911096

M062x-D3/6-311+G(d,p) (IEFPCM, Dichloromethane) Energy = -7062.7505982

Standard orientation:

Center Number	Atomic Number	Atomic Type	Coordinates (Angstroms) X	Y	Z
1	6	0	0.015230	3.700765	0.111891
2	6	0	0.869617	2.852550	-0.510242
3	6	0	3.084212	2.060493	0.570671
4	16	0	-1.741773	1.236957	-0.701626
5	6	0	-2.393362	2.033539	-2.240609
6	9	0	-3.650194	2.490636	-2.016931
7	9	0	-1.617566	3.053057	-2.585341
8	9	0	-2.480212	1.185654	-3.270013
9	1	0	-0.231824	3.491427	1.152600
10	34	0	-3.465199	-0.269922	-0.800515
11	6	0	-3.755556	-5.026882	1.810480
12	6	0	-3.349402	-3.958257	1.015889
13	6	0	-2.271847	-4.101697	0.139773
14	6	0	-1.577166	-5.301530	0.033215
15	6	0	-1.983917	-6.373910	0.831099
16	6	0	-3.063734	-6.237391	1.710418
17	1	0	-4.584266	-4.922092	2.505988
18	1	0	-0.733645	-5.393719	-0.644351
19	1	0	-1.451597	-7.319069	0.774274

20	1	0	−3.361186	−7.078215	2.330815
21	6	0	−2.054489	−2.816802	−0.638175
22	1	0	−2.579003	−2.883786	−1.598512
23	6	0	−2.758794	−1.786398	0.280751
24	1	0	−2.078444	−1.403857	1.040840
25	6	0	−3.924552	−2.557755	0.938555
26	7	0	−0.660900	−2.512241	−0.938808
27	16	0	−0.246613	−1.953827	−2.420877
28	8	0	−1.438401	−1.422627	−3.092819
29	8	0	1.006632	−1.212691	−2.329664
30	6	0	0.141847	−3.532749	−3.343931
31	9	0	−0.953503	−4.306841	−3.365860
32	9	0	0.510787	−3.250401	−4.590108
33	9	0	1.121249	−4.197463	−2.724603
34	1	0	−4.834413	−2.539803	0.318708
35	1	0	−4.182603	−2.135679	1.913122
36	6	0	−4.744386	0.547963	0.373863
37	6	0	−4.378005	0.885107	1.694224
38	6	0	−6.020959	0.829147	−0.145541
39	6	0	−5.324302	1.500281	2.522817
40	6	0	−6.942316	1.444897	0.707429
41	6	0	−6.594511	1.770423	2.018736
42	1	0	−5.072332	1.764047	3.542653
43	1	0	−7.937580	1.670791	0.337079
44	1	0	−7.326819	2.247912	2.663654
45	8	0	−3.116864	0.580605	2.054915
46	6	0	−2.642494	0.936911	3.365307
47	1	0	−1.596666	0.634569	3.387363
48	1	0	−2.736225	2.017313	3.519917
49	1	0	−3.204182	0.394535	4.132968
50	6	0	−6.402170	0.495014	−1.570793
51	1	0	−5.747058	1.000976	−2.288194
52	1	0	−6.334989	−0.580935	−1.771286
53	1	0	−7.429287	0.807715	−1.773571
54	6	0	−0.578449	4.930087	−0.429097
55	6	0	−1.638109	5.539712	0.266358
56	6	0	−0.115385	5.552087	−1.604461
57	6	0	−2.230951	6.710610	−0.202483
58	1	0	−1.997526	5.082826	1.185701
59	6	0	−0.706352	6.721129	−2.073518
60	1	0	0.726613	5.126916	−2.142342
61	6	0	−1.769556	7.304998	−1.377904
62	1	0	−3.049666	7.161426	0.352362
63	1	0	−0.329566	7.186641	−2.980460

64	1	0	-2.225074	8.221089	-1.744049
65	6	0	3.629090	0.862949	1.432597
66	1	0	4.601622	1.137237	1.845135
67	6	0	3.066024	3.330831	1.451835
68	6	0	3.392357	4.597754	0.943310
69	6	0	2.624116	3.265328	2.785491
70	6	0	3.312190	5.744390	1.735300
71	1	0	3.719879	4.692861	-0.085958
72	6	0	2.545595	4.410117	3.580528
73	1	0	2.317599	2.318108	3.216639
74	6	0	2.894862	5.657222	3.062647
75	1	0	3.576957	6.708212	1.307450
76	1	0	2.208704	4.321002	4.610540
77	1	0	2.837117	6.548180	3.682697
78	6	0	4.002748	2.199506	-0.657705
79	6	0	5.325689	2.643878	-0.487103
80	6	0	3.615373	1.812918	-1.948222
81	6	0	6.208754	2.731499	-1.560441
82	1	0	5.672437	2.921721	0.503166
83	6	0	4.498142	1.897942	-3.029008
84	1	0	2.624757	1.411171	-2.129879
85	6	0	5.796957	2.364476	-2.842988
86	1	0	7.225266	3.076272	-1.389886
87	1	0	4.163968	1.586427	-4.015545
88	1	0	6.485186	2.429124	-3.681797
89	1	0	2.942222	0.665596	2.257159
90	7	0	3.817004	-0.376390	0.699207
91	1	0	3.012253	-0.990825	0.632452
92	6	0	5.071311	-0.898654	0.520155
93	8	0	6.094347	-0.323148	0.889201
94	6	0	5.145870	-2.238789	-0.160182
95	6	0	4.158043	-2.719443	-1.029733
96	6	0	6.291796	-3.011818	0.070844
97	6	0	4.312458	-3.962721	-1.645807
98	1	0	3.286410	-2.113124	-1.258102
99	6	0	6.437857	-4.257416	-0.534112
100	1	0	7.057414	-2.609830	0.726243
101	6	0	5.446367	-4.736411	-1.394502
102	1	0	3.548211	-4.321020	-2.328446
103	1	0	7.326346	-4.853131	-0.340574
104	1	0	5.561748	-5.705429	-1.873987
105	1	0	-0.010421	-2.263548	-0.165244
106	8	0	0.976711	-1.592765	1.169318
107	16	0	0.441399	-1.389539	2.557516

108	8	0	0.685705	-0.039630	3.111447
109	8	0	-0.938731	-1.900725	2.731568
110	6	0	1.504118	-2.516747	3.587976
111	9	0	2.792993	-2.180737	3.450649
112	9	0	1.346135	-3.788523	3.201335
113	9	0	1.166198	-2.415284	4.879567
114	1	0	1.138684	3.041880	-1.547688
115	6	0	1.589547	1.735181	0.191664
116	1	0	1.570713	0.822813	-0.413908
117	1	0	1.071508	1.496555	1.125183

Supplementary Table 22.

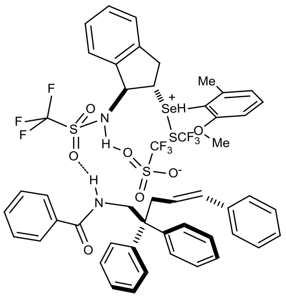

int-II-RRR-b

B3LYP /6-31G(d) 195.15 K Thermal correction to Gibbs Free Energy = 0.817206

Thermal correction to Enthalpy = 0.911309

M062x-D3/6-311+G(d,p) (IEFPCM, Dichloromethane) Energy = -7062.750461

Standard orientation:

Center Number	Atomic Number	Atomic Type	Coordinates (Angstroms) X	Y	Z
1	6	0	0.472815	3.015764	-0.011452
2	6	0	1.539116	2.406373	-0.583330
3	6	0	2.195882	1.177932	-0.016143
4	6	0	3.629422	1.402255	0.580366
5	16	0	-1.082926	0.835715	-1.324324
6	6	0	-0.230441	0.672365	-2.962457
7	9	0	0.266632	1.863133	-3.315409
8	9	0	0.775250	-0.205422	-2.934784
9	9	0	-1.085733	0.283798	-3.938305
10	1	0	0.070662	2.587372	0.904887
11	1	0	2.005068	2.859383	-1.455980
12	1	0	1.557343	0.776658	0.775884
13	1	0	2.260228	0.396582	-0.782361

14	34	0	−2.748132	−0.672671	−1.809356
15	6	0	−5.725367	−3.038866	2.103370
16	6	0	−4.690606	−2.616951	1.273342
17	6	0	−3.414619	−3.171017	1.403090
18	6	0	−3.139271	−4.149190	2.351522
19	6	0	−4.178008	−4.571629	3.185423
20	6	0	−5.458509	−4.021831	3.061126
21	1	0	−6.718706	−2.605240	2.021668
22	1	0	−2.139096	−4.561503	2.445290
23	1	0	−3.987377	−5.327463	3.941980
24	1	0	−6.252653	−4.354564	3.723699
25	6	0	−2.487314	−2.581033	0.357218
26	1	0	−2.495299	−3.228126	−0.528037
27	6	0	−3.212875	−1.248845	0.038333
28	1	0	−2.930324	−0.469274	0.745015
29	6	0	−4.714929	−1.575277	0.170667
30	7	0	−1.091396	−2.444334	0.775026
31	16	0	0.092610	−2.808433	−0.303677
32	8	0	−0.413636	−2.741833	−1.681422
33	8	0	1.322136	−2.133156	0.100968
34	6	0	0.366335	−4.629774	0.019916
35	9	0	−0.790211	−5.285088	−0.162194
36	9	0	1.278459	−5.094324	−0.830357
37	9	0	0.778802	−4.813480	1.271227
38	1	0	−5.128724	−1.993652	−0.759715
39	1	0	−5.296805	−0.685295	0.421449
40	6	0	−4.162825	0.518878	−2.321040
41	6	0	−4.543668	1.572506	−1.462616
42	6	0	−4.739974	0.342372	−3.592228
43	6	0	−5.529241	2.471062	−1.888085
44	6	0	−5.726955	1.252274	−3.983731
45	6	0	−6.108374	2.298401	−3.142993
46	1	0	−5.835404	3.291977	−1.251510
47	1	0	−6.194571	1.139843	−4.957069
48	1	0	−6.873303	2.996617	−3.470981
49	8	0	−3.914960	1.621790	−0.273045
50	6	0	−4.181462	2.709709	0.630982
51	1	0	−3.540461	2.528319	1.491638
52	1	0	−3.920985	3.662838	0.160373
53	1	0	−5.234657	2.702732	0.931952
54	6	0	−4.319739	−0.775381	−4.520426
55	1	0	−3.265949	−0.685549	−4.805919
56	1	0	−4.446146	−1.760909	−4.057306
57	1	0	−4.918207	−0.757552	−5.434578

58	6	0	-0.167220	4.267885	-0.438843
59	6	0	-1.057229	4.893920	0.454729
60	6	0	0.063574	4.877593	-1.686296
61	6	0	-1.678889	6.096829	0.119421
62	1	0	-1.243680	4.427872	1.418390
63	6	0	-0.561591	6.075442	-2.020449
64	1	0	0.725647	4.404119	-2.404989
65	6	0	-1.434129	6.693121	-1.118880
66	1	0	-2.349980	6.572454	0.830516
67	1	0	-0.369496	6.530256	-2.988975
68	1	0	-1.916952	7.630823	-1.381547
69	6	0	4.045308	0.076477	1.314468
70	1	0	5.009782	0.214700	1.805019
71	6	0	4.694296	1.678841	-0.498248
72	6	0	4.431453	1.610430	-1.873775
73	6	0	6.021831	1.935222	-0.110578
74	6	0	5.439890	1.815112	-2.820274
75	1	0	3.435508	1.380216	-2.235145
76	6	0	7.030148	2.140891	-1.048779
77	1	0	6.273399	1.972694	0.944586
78	6	0	6.743554	2.086786	-2.413964
79	1	0	5.198419	1.753851	-3.878618
80	1	0	8.044726	2.332750	-0.709854
81	1	0	7.528942	2.244156	-3.148544
82	6	0	3.518784	2.525787	1.638655
83	6	0	4.093583	3.793540	1.469201
84	6	0	2.739553	2.318667	2.791549
85	6	0	3.925039	4.800140	2.423054
86	1	0	4.680207	4.005524	0.582622
87	6	0	2.565227	3.322429	3.743147
88	1	0	2.226608	1.376040	2.951465
89	6	0	3.165515	4.569702	3.568538
90	1	0	4.387671	5.770525	2.260038
91	1	0	1.942856	3.123936	4.611177
92	1	0	3.031725	5.354204	4.308833
93	1	0	3.303919	-0.155299	2.084598
94	6	0	5.377195	-1.705495	0.245356
95	8	0	6.431043	-1.304412	0.734957
96	6	0	5.339848	-2.949035	-0.606094
97	6	0	4.303660	-3.249612	-1.501260
98	6	0	6.420486	-3.833905	-0.493391
99	6	0	4.339979	-4.424324	-2.253476
100	1	0	3.476847	-2.559897	-1.637944
101	6	0	6.454083	-5.008926	-1.240570

102	1	0	7.224328	-3.574234	0.187507
103	6	0	5.411462	-5.309107	-2.121083
104	1	0	3.532728	-4.643714	-2.947392
105	1	0	7.294438	-5.690783	-1.138955
106	1	0	5.437909	-6.224565	-2.706715
107	1	0	-0.850751	-1.640768	1.396110
108	8	0	-0.589094	-0.003447	2.062164
109	16	0	-1.567041	0.784768	2.882611
110	8	0	-1.452123	2.248296	2.715546
111	8	0	-2.944254	0.242977	2.824679
112	6	0	-0.999401	0.472003	4.627600
113	9	0	-1.035907	-0.839103	4.902023
114	9	0	-1.785219	1.115239	5.498482
115	9	0	0.263318	0.903731	4.792441
116	7	0	4.176203	-1.077214	0.441901
117	1	0	3.327292	-1.570328	0.200717

Supplementary Table 23.

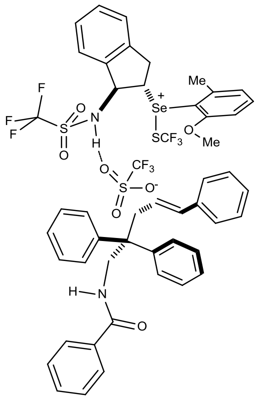

int-II-SSS

B3LYP /6-31G(d) 195.15 K Thermal correction to Gibbs Free Energy = 0.81612

Thermal correction to Enthalpy = 0.911147

M062x-D3/6-311+G(d,p) (IEFPCM, Dichloromethane) Energy = -7062.74772613

Standard orientation:

Center Number	Atomic Number	Atomic Type	Coordinates (Angstroms) X	Y	Z
1	6	0	0.982495	3.090699	0.312111
2	6	0	1.297068	1.783963	0.484638
3	6	0	2.186895	1.011326	-0.448510
4	6	0	3.639835	0.666508	0.064115
5	16	0	-1.366665	1.339561	-1.004605

6	6	0	−0.829703	1.805860	−2.712563
7	9	0	−1.784251	2.564864	−3.306757
8	9	0	0.293796	2.518822	−2.656804
9	9	0	−0.638137	0.746913	−3.500947
10	1	0	1.385662	3.596911	−0.564560
11	1	0	0.932353	1.253533	1.362324
12	1	0	1.690035	0.071648	−0.717529
13	1	0	2.318349	1.575574	−1.377944
14	34	0	−3.262679	0.401106	−1.881044
15	6	0	−6.350098	−3.085843	0.979981
16	6	0	−5.316102	−2.469998	0.280031
17	6	0	−4.136751	−3.168637	0.006405
18	6	0	−3.960042	−4.484500	0.418568
19	6	0	−4.997702	−5.101935	1.122033
20	6	0	−6.181119	−4.408945	1.399177
21	1	0	−7.266726	−2.548420	1.209366
22	1	0	−3.034519	−5.010414	0.204579
23	1	0	−4.881007	−6.126477	1.463651
24	1	0	−6.974322	−4.901025	1.955154
25	6	0	−3.191709	−2.293379	−0.796448
26	1	0	−3.351182	−2.478518	−1.865235
27	6	0	−3.720500	−0.883541	−0.423767
28	1	0	−3.272164	−0.525558	0.507214
29	6	0	−5.245167	−1.060417	−0.277793
30	7	0	−1.775096	−2.501471	−0.538591
31	16	0	−0.705433	−2.545864	−1.773784
32	8	0	−1.341641	−1.980085	−2.969602
33	8	0	0.609344	−2.120363	−1.307127
34	6	0	−0.560140	−4.375634	−2.125515
35	9	0	−1.779232	−4.862988	−2.405234
36	9	0	0.244343	−4.574926	−3.167004
37	9	0	−0.077320	−5.007381	−1.056170
38	1	0	−5.762144	−0.995941	−1.247703
39	1	0	−5.679394	−0.303110	0.378989
40	6	0	−4.529620	1.830691	−1.690726
41	6	0	−4.731015	2.429249	−0.429430
42	6	0	−5.181845	2.286833	−2.850810
43	6	0	−5.604519	3.518242	−0.326313
44	6	0	−6.057960	3.367888	−2.712180
45	6	0	−6.257409	3.972056	−1.470070
46	1	0	−5.767152	4.003856	0.628217
47	1	0	−6.580910	3.740857	−3.587468
48	1	0	−6.935974	4.816640	−1.389538
49	8	0	−4.050186	1.879771	0.591628

50	6	0	−4.132632	2.451489	1.907973
51	1	0	−3.541957	1.781124	2.530147
52	1	0	−3.708349	3.460028	1.911234
53	1	0	−5.173230	2.470961	2.251207
54	6	0	−4.953877	1.653375	−4.205133
55	1	0	−3.908217	1.744957	−4.518390
56	1	0	−5.199063	0.584838	−4.203113
57	1	0	−5.575778	2.136956	−4.962402
58	6	0	0.200708	3.954281	1.206634
59	6	0	0.013811	5.300368	0.840076
60	6	0	−0.357210	3.510777	2.422881
61	6	0	−0.702342	6.178672	1.651108
62	1	0	0.444574	5.658490	−0.092497
63	6	0	−1.069757	4.392574	3.232685
64	1	0	−0.237219	2.479491	2.740594
65	6	0	−1.248145	5.727638	2.854136
66	1	0	−0.827994	7.214743	1.347053
67	1	0	−1.481126	4.034175	4.173109
68	1	0	−1.798995	6.410545	3.495981
69	6	0	4.204165	−0.270013	−1.057219
70	1	0	3.529213	−1.132224	−1.123711
71	6	0	4.410663	1.991693	0.197444
72	6	0	5.235677	2.490291	−0.821975
73	6	0	4.219921	2.795712	1.335288
74	6	0	5.848476	3.741240	−0.702966
75	1	0	5.431167	1.907935	−1.716052
76	6	0	4.828824	4.044403	1.453618
77	1	0	3.592410	2.436059	2.144343
78	6	0	5.648941	4.525366	0.431697
79	1	0	6.490004	4.095146	−1.506218
80	1	0	4.659449	4.640058	2.347121
81	1	0	6.128579	5.496777	0.521947
82	6	0	3.654506	−0.154258	1.370478
83	6	0	2.591593	−0.996335	1.729489
84	6	0	4.801778	−0.169138	2.186598
85	6	0	2.657060	−1.800167	2.869500
86	1	0	1.686257	−1.040029	1.135903
87	6	0	4.876381	−0.984734	3.317048
88	1	0	5.638107	0.480860	1.947406
89	6	0	3.800315	−1.801386	3.667005
90	1	0	1.796666	−2.408632	3.130235
91	1	0	5.774880	−0.969003	3.929315
92	1	0	3.850282	−2.424933	4.555911
93	1	0	4.196434	0.225839	−2.029216

94	7	0	5.560267	-0.738592	-0.839965
95	1	0	5.764057	-1.158550	0.056182
96	6	0	6.568368	-0.570296	-1.743546
97	8	0	6.422635	0.046988	-2.800520
98	6	0	7.898099	-1.171574	-1.372580
99	6	0	8.047052	-2.229871	-0.465143
100	6	0	9.035473	-0.647136	-2.000933
101	6	0	9.313551	-2.739876	-0.177967
102	1	0	7.177311	-2.686892	-0.000442
103	6	0	10.300351	-1.150857	-1.707946
104	1	0	8.899810	0.155418	-2.718212
105	6	0	10.442914	-2.197483	-0.793250
106	1	0	9.415942	-3.565800	0.520787
107	1	0	11.176214	-0.729925	-2.194598
108	1	0	11.429235	-2.593954	-0.566803
109	1	0	-1.385346	-2.317409	0.412166
110	8	0	-0.808188	-1.933928	1.996550
111	16	0	-1.324151	-0.828831	2.860244
112	8	0	-0.344091	0.226835	3.173012
113	8	0	-2.671560	-0.343291	2.453764
114	6	0	-1.620707	-1.686372	4.483862
115	9	0	-2.534392	-2.656622	4.342350
116	9	0	-2.066407	-0.812883	5.397819
117	9	0	-0.484284	-2.234937	4.935500

Supplementary Table 24.

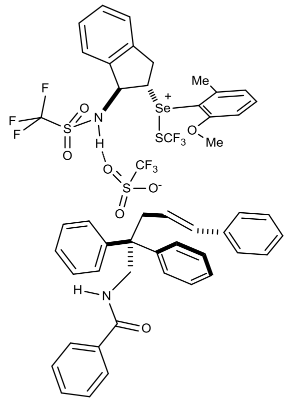

int-II-SRR

B3LYP /6-31G(d) 195.15 K Thermal correction to Gibbs Free Energy = 0.815313

Thermal correction to Enthalpy = 0.911012

M062x-D3/6-311+G(d,p) (IEFPCM, Dichloromethane) Energy = -7062.74497132

Standard orientation:

Center Number	Atomic Number	Atomic Type	Coordinates (Angstroms) X	Y	Z
1	6	0	0.816445	2.898099	0.719409
2	6	0	1.571467	1.974335	0.084736
3	6	0	1.865265	0.613790	0.652891
4	6	0	3.392144	0.247979	0.716112
5	16	0	-1.444190	1.260234	-0.803089
6	6	0	-1.205871	2.026330	-2.469844
7	9	0	-2.241398	2.848672	-2.759929
8	9	0	-0.081779	2.734926	-2.480963
9	9	0	-1.160287	1.115696	-3.448962
10	1	0	0.366735	2.609468	1.669279
11	1	0	2.019768	2.219926	-0.876597
12	1	0	1.439368	0.547609	1.655549
13	1	0	1.355951	-0.146525	0.048978
14	34	0	-3.392743	0.332590	-1.522453
15	6	0	-6.052693	-3.503592	1.312799
16	6	0	-5.113539	-2.784845	0.578353
17	6	0	-3.949647	-3.408497	0.121243
18	6	0	-3.694285	-4.750337	0.380920
19	6	0	-4.636830	-5.471181	1.118358
20	6	0	-5.804828	-4.853319	1.578525
21	1	0	-6.953859	-3.024837	1.686989
22	1	0	-2.779951	-5.216564	0.026926
23	1	0	-4.456308	-6.517983	1.345276
24	1	0	-6.521872	-5.425786	2.160301
25	6	0	-3.117671	-2.427373	-0.683652
26	1	0	-3.377580	-2.523002	-1.744622
27	6	0	-3.648531	-1.077626	-0.131206
28	1	0	-3.117187	-0.774033	0.771053
29	6	0	-5.141076	-1.325455	0.164462
30	7	0	-1.672002	-2.610479	-0.577914
31	16	0	-0.738206	-2.438815	-1.922208
32	8	0	-1.503943	-1.767134	-2.980892
33	8	0	0.600046	-2.007515	-1.532860
34	6	0	-0.579042	-4.211206	-2.492960
35	9	0	-1.806520	-4.723568	-2.669195
36	9	0	0.085363	-4.249913	-3.645947
37	9	0	0.065360	-4.931135	-1.577475
38	1	0	-5.769518	-1.179954	-0.727563
39	1	0	-5.508808	-0.656089	0.945254
40	6	0	-4.696008	1.679546	-1.110005
41	6	0	-4.815782	2.185894	0.200472

42	6	0	−5.473362	2.168677	−2.176750
43	6	0	−5.744993	3.202653	0.452518
44	6	0	−6.397551	3.177801	−1.890662
45	6	0	−6.524819	3.683621	−0.596471
46	1	0	−5.854669	3.612824	1.448902
47	1	0	−7.016158	3.571791	−2.691103
48	1	0	−7.245343	4.472349	−0.399043
49	8	0	−4.006826	1.631216	1.121532
50	6	0	−4.039606	2.109099	2.475272
51	1	0	−3.336138	1.476965	3.011447
52	1	0	−3.738613	3.161889	2.512049
53	1	0	−5.042503	1.989553	2.899425
54	6	0	−5.326992	1.642235	−3.587412
55	1	0	−4.307903	1.783543	−3.962669
56	1	0	−5.549852	0.570525	−3.649686
57	1	0	−6.010183	2.164162	−4.261796
58	6	0	0.547484	4.281216	0.299461
59	6	0	−0.453545	5.009805	0.966777
60	6	0	1.262663	4.929839	−0.724892
61	6	0	−0.748523	6.325171	0.612998
62	1	0	−1.002838	4.530653	1.774355
63	6	0	0.968700	6.243226	−1.079509
64	1	0	2.069814	4.409209	−1.231219
65	6	0	−0.040661	6.947447	−0.416174
66	1	0	−1.527002	6.866335	1.144883
67	1	0	1.537827	6.724880	−1.870362
68	1	0	−0.263100	7.974740	−0.691987
69	6	0	3.816113	−0.078861	−0.751995
70	1	0	3.115039	−0.835132	−1.125929
71	6	0	3.604524	−1.028499	1.562271
72	6	0	4.877275	−1.315007	2.090872
73	6	0	2.578617	−1.957456	1.791761
74	6	0	5.116339	−2.486749	2.810105
75	1	0	5.682336	−0.597397	1.962096
76	6	0	2.812965	−3.122404	2.526758
77	1	0	1.575106	−1.777671	1.425573
78	6	0	4.082099	−3.397139	3.033781
79	1	0	6.109665	−2.676808	3.209425
80	1	0	1.986490	−3.803148	2.711861
81	1	0	4.261354	−4.302609	3.607709
82	6	0	4.143137	1.437925	1.340309
83	6	0	4.983177	2.289128	0.610681
84	6	0	3.923774	1.731061	2.698261
85	6	0	5.588447	3.392612	1.222642

86	1	0	5.184907	2.107591	−0.439988
87	6	0	4.522362	2.831958	3.305510
88	1	0	3.273840	1.087282	3.285234
89	6	0	5.363308	3.670062	2.568647
90	1	0	6.240692	4.033369	0.634358
91	1	0	4.332936	3.034760	4.356737
92	1	0	5.835297	4.527815	3.040975
93	1	0	3.729646	0.797099	−1.397063
94	7	0	5.173499	−0.571049	−0.916350
95	1	0	5.450396	−1.354266	−0.341821
96	6	0	6.076144	−0.022346	−1.778072
97	8	0	5.844053	1.002828	−2.423330
98	6	0	7.402770	−0.726185	−1.888496
99	6	0	7.599718	−2.073291	−1.553053
100	6	0	8.482507	0.017350	−2.383498
101	6	0	8.858816	−2.658150	−1.691028
102	1	0	6.769744	−2.687123	−1.213160
103	6	0	9.740996	−0.564649	−2.514482
104	1	0	8.307158	1.051256	−2.661466
105	6	0	9.933095	−1.903926	−2.165728
106	1	0	8.997398	−3.705037	−1.434738
107	1	0	10.572806	0.024578	−2.891309
108	1	0	10.913961	−2.360140	−2.270783
109	1	0	−1.226909	−2.257315	0.294105
110	8	0	−0.894791	−1.154119	1.651434
111	16	0	−1.499967	−1.307246	3.021871
112	8	0	−2.899564	−0.817368	3.075871
113	8	0	−1.209636	−2.583383	3.683888
114	6	0	−0.563520	−0.028628	4.004159
115	9	0	0.759197	−0.247655	3.961736
116	9	0	−0.956385	−0.031471	5.281841
117	9	0	−0.792652	1.210011	3.502895

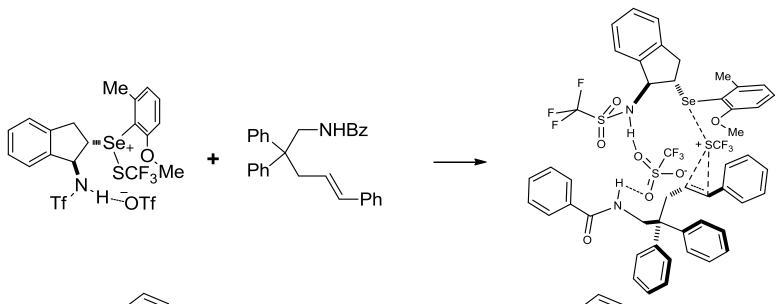

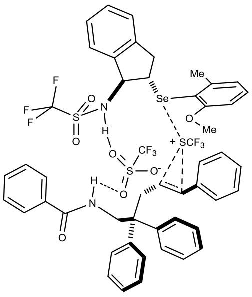

TS-I-RSS-a

$\Delta G_{195.15\ K}$ = -3.1 kcal/mol

$\Delta\Delta G_{195.15\ K}$ = 0.0 kcal/mol

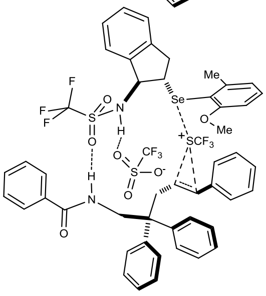

TS-I-RSS-b

$\Delta G_{195.15\ K}$ = -1.9 kcal/mol

$\Delta\Delta G_{195.15\ K}$ = 1.2 kcal/mol

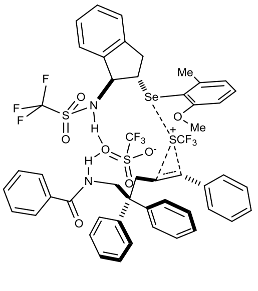

TS-I-RRR-a

$\Delta G_{195.15\ K}$ = - 2.0 kcal/mol

$\Delta\Delta G_{195.15\ K}$ = 1.1 kcal/mol

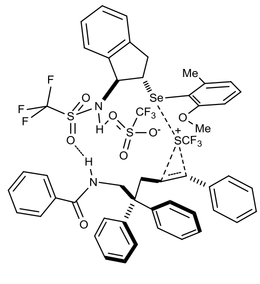

TS-I-RRR-b

$\Delta G_{195.15\ K}$ = +1.8 kcal/mol

$\Delta\Delta G_{195.15\ K}$ = 4.9 kcal/mol

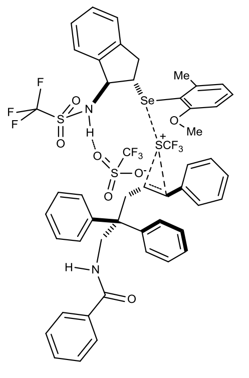

TS-I-SSS

$\Delta G_{195.15\ K}$ = +1.5 kcal/mol

$\Delta\Delta G_{195.15\ K}$ = 4.6 kcal/mol

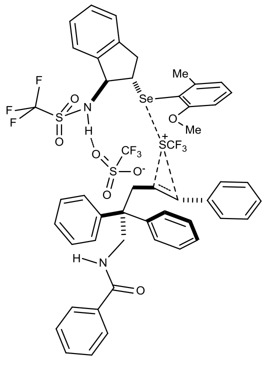

TS-I-SRR

$\Delta G_{195.15\ K}$ = +3.4 kcal/mol

$\Delta\Delta G_{195.15\ K}$ = 6.2 kcal/mol

Supplementary Figure 180. DFT calculations for **TS-I** of **1a**; ΔG related to **int-I**.

Supplementary Table 25.

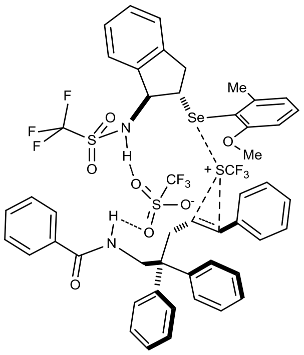

TS-I-RSS-a

B3LYP /6-31G(d) 195.15 K Thermal correction to Gibbs Free Energy = 0.818508

Thermal correction to Energy = 0.910978

M062x-D3/6-311+G(d,p) (IEFPCM, Dichloromethane) Energy = -7062.74721054

Standard orientation:

Center Number	Atomic Number	Atomic Type	Coordinates (Angstroms) X	Y	Z
1	6	0	-1.155870	-3.076975	0.425083
2	6	0	-1.315676	-1.706034	0.142300
3	6	0	-2.283951	-1.143870	-0.880915
4	6	0	-3.791411	-1.282443	-0.460767
5	16	0	0.746595	-1.590234	-0.598322
6	6	0	0.538099	-2.287343	-2.303433
7	9	0	1.646680	-2.974606	-2.664325
8	9	0	-0.493165	-3.148485	-2.400494
9	9	0	0.353961	-1.327548	-3.215489
10	1	0	-1.402910	-3.787415	-0.357506
11	1	0	-1.131253	-1.024646	0.970827
12	1	0	-2.040655	-0.086250	-0.996202
13	1	0	-2.160021	-1.621445	-1.853513
14	34	0	3.067701	-0.626122	-1.627270
15	6	0	6.039360	3.094247	1.075576
16	6	0	5.039727	2.416003	0.381853
17	6	0	3.919152	3.107282	-0.086979
18	6	0	3.770748	4.474828	0.119199
19	6	0	4.773067	5.153740	0.817324
20	6	0	5.897291	4.468641	1.290755
21	1	0	6.911501	2.566062	1.453232
22	1	0	2.890681	4.995548	-0.247049
23	1	0	4.675159	6.220279	0.999703
24	1	0	6.664824	5.008847	1.838466
25	6	0	2.998678	2.158935	-0.832255

26	1	0	3.207619	2.214846	−1.907040
27	6	0	3.446506	0.777799	−0.279091
28	1	0	2.887919	0.532164	0.624991
29	6	0	4.953218	0.948338	0.012370
30	7	0	1.578178	2.450607	−0.664271
31	16	0	0.526338	2.320224	−1.904425
32	8	0	1.156458	1.595691	−3.010356
33	8	0	−0.796635	1.968483	−1.390806
34	6	0	0.380533	4.087823	−2.493544
35	9	0	1.596461	4.534049	−2.840944
36	9	0	−0.432575	4.155385	−3.545316
37	9	0	−0.094461	4.857027	−1.509748
38	1	0	5.559097	0.734949	−0.879981
39	1	0	5.293236	0.274788	0.803564
40	6	0	4.246290	−2.060830	−1.123660
41	6	0	4.313640	−2.509019	0.211176
42	6	0	4.990986	−2.690996	−2.136863
43	6	0	5.139564	−3.588985	0.540995
44	6	0	5.809324	−3.771201	−1.782678
45	6	0	5.881165	−4.210136	−0.462746
46	1	0	5.202069	−3.941648	1.563635
47	1	0	6.393043	−4.267381	−2.552616
48	1	0	6.523076	−5.048739	−0.206992
49	8	0	3.551251	−1.830354	1.099881
50	6	0	3.461412	−2.285029	2.449267
51	1	0	2.752238	−1.609053	2.924328
52	1	0	3.083168	−3.313222	2.489440
53	1	0	4.436487	−2.229571	2.949283
54	6	0	4.911429	−2.241467	−3.578011
55	1	0	3.903465	−2.384232	−3.984332
56	1	0	5.145360	−1.176011	−3.681448
57	1	0	5.610094	−2.809229	−4.198530
58	6	0	−0.700568	−3.646740	1.672166
59	6	0	−0.487817	−5.042655	1.732751
60	6	0	−0.492280	−2.876148	2.840083
61	6	0	−0.071660	−5.648433	2.912092
62	1	0	−0.657011	−5.642645	0.842397
63	6	0	−0.073604	−3.488405	4.015180
64	1	0	−0.668250	−1.807129	2.833439
65	6	0	0.139350	−4.871094	4.055984
66	1	0	0.085992	−6.722615	2.944752
67	1	0	0.082455	−2.881330	4.901969
68	1	0	0.461362	−5.343474	4.980192
69	6	0	−4.034132	−0.571073	0.910137

70	1	0	-5.080970	-0.694966	1.197529
71	6	0	-4.124140	-2.786843	-0.369767
72	6	0	-4.117360	-3.558425	-1.547392
73	6	0	-4.403221	-3.447971	0.835412
74	6	0	-4.362797	-4.928409	-1.520887
75	1	0	-3.935074	-3.069716	-2.500414
76	6	0	-4.657809	-4.824140	0.865183
77	1	0	-4.434034	-2.899458	1.769906
78	6	0	-4.634027	-5.571750	-0.308812
79	1	0	-4.353491	-5.494022	-2.449164
80	1	0	-4.876697	-5.304507	1.815301
81	1	0	-4.833933	-6.639734	-0.286210
82	6	0	-4.699520	-0.626381	-1.526363
83	6	0	-6.082592	-0.867479	-1.473688
84	6	0	-4.222614	0.225953	-2.530225
85	6	0	-6.954322	-0.289486	-2.391636
86	1	0	-6.483412	-1.516921	-0.700992
87	6	0	-5.096095	0.805537	-3.455868
88	1	0	-3.168321	0.465651	-2.604962
89	6	0	-6.463179	0.549770	-3.393868
90	1	0	-8.019716	-0.491768	-2.320108
91	1	0	-4.696737	1.463849	-4.223098
92	1	0	-7.140965	1.003069	-4.112445
93	1	0	-3.410164	-1.031818	1.683077
94	7	0	-3.752565	0.851803	0.901413
95	1	0	-2.837538	1.126487	1.245393
96	6	0	-4.777560	1.764478	0.994501
97	8	0	-5.961466	1.433186	0.986529
98	6	0	-4.367893	3.206650	1.105894
99	6	0	-3.102616	3.679271	0.730766
100	6	0	-5.324312	4.111604	1.586446
101	6	0	-2.797966	5.035603	0.850802
102	1	0	-2.359702	3.006087	0.316289
103	6	0	-5.015038	5.463270	1.713574
104	1	0	-6.304917	3.729755	1.851007
105	6	0	-3.748897	5.928387	1.346991
106	1	0	-1.817357	5.390129	0.546563
107	1	0	-5.760834	6.156084	2.095051
108	1	0	-3.507705	6.984263	1.442046
109	1	0	1.160843	2.458123	0.284708
110	8	0	0.503910	2.420697	1.962651
111	16	0	0.235138	1.060006	2.492095
112	8	0	-1.182164	0.618611	2.414772
113	8	0	1.210044	0.025941	2.055056

114	6	0	0.544462	1.197004	4.321772
115	9	0	1.801913	1.592225	4.556410
116	9	0	0.370318	-0.011728	4.894954
117	9	0	-0.295791	2.062440	4.890979

Supplementary Table 26.

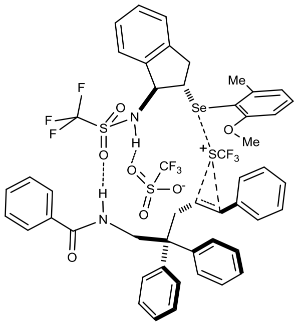

TS-I-RSS-b

B3LYP /6-31G(d) 195.15 K Thermal correction to Gibbs Free Energy = 0.819643

Thermal correction to Energy = 0.910983

M062x-D3/6-311+G(d,p) (IEFPCM, Dichloromethane) Energy = -7062.74629073

Standard orientation:

Center Number	Atomic Number	Atomic Type	Coordinates (Angstroms) X	Y	Z
1	6	0	-0.622248	-3.253603	-0.094487
2	6	0	-1.027053	-1.905525	-0.084090
3	6	0	-2.167469	-1.400881	-0.944272
4	6	0	-3.581281	-1.595205	-0.283170
5	16	0	0.934392	-1.327419	-0.936710
6	6	0	0.628463	-1.666828	-2.733490
7	9	0	1.776544	-2.041822	-3.343850
8	9	0	-0.250315	-2.667838	-2.941264
9	9	0	0.173422	-0.585794	-3.376299
10	1	0	-0.847119	-3.824703	-0.990732
11	1	0	-0.867222	-1.351606	0.839570
12	1	0	-2.015194	-0.336132	-1.140510
13	1	0	-2.166477	-1.923529	-1.902122
14	34	0	2.979648	0.178456	-1.867738
15	6	0	5.748376	3.604274	1.369483
16	6	0	4.759027	2.948607	0.640141
17	6	0	3.502338	3.537246	0.476677
18	6	0	3.204703	4.778701	1.029448
19	6	0	4.197074	5.434474	1.762801
20	6	0	5.457997	4.851738	1.930315

21	1	0	6.727185	3.152460	1.510872
22	1	0	2.219697	5.218221	0.900729
23	1	0	3.985197	6.400540	2.212377
24	1	0	6.217162	5.370261	2.509697
25	6	0	2.617992	2.649424	-0.378716
26	1	0	2.655127	2.989776	-1.420195
27	6	0	3.321097	1.269053	-0.246043
28	1	0	2.922871	0.723424	0.610051
29	6	0	4.817167	1.610342	-0.067974
30	7	0	1.211845	2.666939	0.023514
31	16	0	0.018395	2.587916	-1.081260
32	8	0	0.566583	2.216964	-2.387578
33	8	0	-1.142666	1.907381	-0.504400
34	6	0	-0.500050	4.373746	-1.261540
35	9	0	0.548785	5.098666	-1.670468
36	9	0	-1.479075	4.464933	-2.167966
37	9	0	-0.931326	4.847017	-0.094178
38	1	0	5.322409	1.705621	-1.039850
39	1	0	5.345536	0.836947	0.496079
40	6	0	4.383855	-1.136957	-1.819982
41	6	0	4.655045	-1.866952	-0.644248
42	6	0	5.088862	-1.394002	-3.009537
43	6	0	5.643087	-2.857724	-0.654078
44	6	0	6.072068	-2.391610	-2.994830
45	6	0	6.342940	-3.110201	-1.832835
46	1	0	5.861795	-3.424924	0.242857
47	1	0	6.626981	-2.600817	-3.904781
48	1	0	7.110384	-3.879413	-1.840368
49	8	0	3.917344	-1.534809	0.440068
50	6	0	4.036383	-2.297414	1.640373
51	1	0	3.305605	-1.864852	2.321741
52	1	0	3.797743	-3.351429	1.456551
53	1	0	5.046484	-2.214274	2.060627
54	6	0	4.799498	-0.636363	-4.285406
55	1	0	3.778918	-0.823765	-4.638596
56	1	0	4.889990	0.446035	-4.140528
57	1	0	5.491539	-0.936158	-5.077080
58	6	0	0.042661	-3.985228	0.956932
59	6	0	0.496082	-5.293861	0.674030
60	6	0	0.224710	-3.467313	2.259731
61	6	0	1.123260	-6.056016	1.651721
62	1	0	0.349546	-5.701508	-0.322978
63	6	0	0.853943	-4.235565	3.232368
64	1	0	-0.139772	-2.478816	2.514314

65	6	0	1.305444	−5.525871	2.933676
66	1	0	1.466154	−7.060642	1.421476
67	1	0	0.989621	−3.822861	4.227250
68	1	0	1.791666	−6.121783	3.701619
69	6	0	−3.723874	−0.663317	0.967198
70	1	0	−4.621565	−0.942347	1.521356
71	6	0	−3.724207	−3.078912	0.116646
72	6	0	−3.875340	−4.051554	−0.889085
73	6	0	−3.669902	−3.521147	1.446671
74	6	0	−3.966194	−5.407072	−0.580018
75	1	0	−3.946101	−3.735610	−1.926451
76	6	0	−3.767671	−4.880360	1.760015
77	1	0	−3.550289	−2.811623	2.257698
78	6	0	−3.913691	−5.829450	0.751005
79	1	0	−4.090922	−6.133335	−1.379376
80	1	0	−3.728246	−5.190897	2.800764
81	1	0	−3.992681	−6.885256	0.996068
82	6	0	−4.691657	−1.228436	−1.293555
83	6	0	−6.027289	−1.504993	−0.957726
84	6	0	−4.443354	−0.602573	−2.521848
85	6	0	−7.071704	−1.176849	−1.818002
86	1	0	−6.253806	−1.977944	−0.007422
87	6	0	−5.489784	−0.274384	−3.389318
88	1	0	−3.432084	−0.352777	−2.823560
89	6	0	−6.807803	−0.561371	−3.043419
90	1	0	−8.094733	−1.398331	−1.526032
91	1	0	−5.265346	0.211152	−4.335679
92	1	0	−7.621712	−0.304379	−3.716192
93	1	0	−2.864546	−0.775451	1.634979
94	7	0	−3.880362	0.745579	0.648060
95	1	0	−3.044925	1.293490	0.486312
96	6	0	−5.053757	1.394314	0.942421
97	8	0	−6.032789	0.809274	1.404223
98	6	0	−5.096650	2.874479	0.677476
99	6	0	−4.243271	3.531140	−0.218899
100	6	0	−6.076476	3.613509	1.354835
101	6	0	−4.360194	4.907339	−0.420278
102	1	0	−3.502522	2.973144	−0.781568
103	6	0	−6.186784	4.987622	1.159313
104	1	0	−6.742655	3.084709	2.028253
105	6	0	−5.326802	5.638675	0.271051
106	1	0	−3.695829	5.404120	−1.120800
107	1	0	−6.944932	5.551660	1.696440
108	1	0	−5.413928	6.710875	0.114126

109	1	0	0.944834	2.378635	0.986599
110	8	0	0.487318	1.849321	2.626819
111	16	0	0.504478	0.372516	2.793229
112	8	0	-0.819515	-0.283881	2.883514
113	8	0	1.465080	-0.323594	1.892881
114	6	0	1.249883	0.116182	4.479660
115	9	0	2.449411	0.707265	4.558079
116	9	0	1.424167	-1.205435	4.698778
117	9	0	0.463496	0.603622	5.441309

Supplementary Table 27.

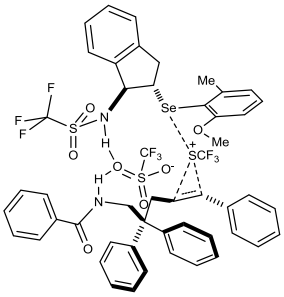

TS-I-RRR-a

B3LYP /6-31G(d) 195.15 K Thermal correction to Gibbs Free Energy = 0.820024

Thermal correction to Energy = 0.911175

M062x-D3/6-311+G(d,p) (IEFPCM, Dichloromethane) Energy = -7062.74687138644

Standard orientation:

Center Number	Atomic Number	Atomic Type	Coordinates (Angstroms) X	Y	Z
1	6	0	0.875743	3.267606	0.463535
2	6	0	1.330567	2.177741	-0.326952
3	6	0	3.492846	1.098385	0.588870
4	16	0	-0.721649	1.697065	-0.609102
5	6	0	-0.944606	2.506528	-2.264089
6	9	0	-2.126426	3.153608	-2.308691
7	9	0	0.013178	3.409354	-2.541157
8	9	0	-0.943057	1.603382	-3.249405
9	1	0	0.531234	3.000468	1.460499
10	34	0	-3.142128	0.462333	-1.363181
11	6	0	-5.075014	-3.734885	1.594718
12	6	0	-4.315374	-2.890748	0.788664
13	6	0	-3.159265	-3.370434	0.165275
14	6	0	-2.741180	-4.687062	0.324152

15	6	0	−3.503553	−5.532527	1.135251
16	6	0	−4.659903	−5.059672	1.764405
17	1	0	−5.970318	−3.371680	2.093544
18	1	0	−1.838060	−5.041852	−0.164107
19	1	0	−3.192002	−6.562786	1.283581
20	1	0	−5.237885	−5.726685	2.398467
21	6	0	−2.537209	−2.271544	−0.676197
22	1	0	−2.897676	−2.357530	−1.708078
23	6	0	−3.134533	−0.996475	−0.024497
24	1	0	−2.518339	−0.679905	0.817644
25	6	0	−4.540230	−1.431947	0.438736
26	7	0	−1.078367	−2.298268	−0.740379
27	16	0	−0.287296	−2.055680	−2.148095
28	8	0	−1.181521	−1.412286	−3.113588
29	8	0	1.054525	−1.550698	−1.866269
30	6	0	−0.056945	−3.794288	−2.793832
31	9	0	−1.264094	−4.356886	−2.961391
32	9	0	0.581293	−3.765327	−3.962324
33	9	0	0.638667	−4.520662	−1.919066
34	1	0	−5.273265	−1.332961	−0.374892
35	1	0	−4.901093	−0.829830	1.276990
36	6	0	−4.405594	1.731549	−0.654025
37	6	0	−4.385475	2.116236	0.702210
38	6	0	−5.326823	2.305403	−1.549228
39	6	0	−5.307366	3.057739	1.173660
40	6	0	−6.233369	3.253248	−1.057181
41	6	0	−6.225071	3.618115	0.286480
42	1	0	−5.307279	3.354832	2.215699
43	1	0	−6.952087	3.701007	−1.737535
44	1	0	−6.939727	4.350189	0.652761
45	8	0	−3.441078	1.524025	1.472978
46	6	0	−3.375358	1.829615	2.864162
47	1	0	−2.564314	1.213916	3.249948
48	1	0	−3.151109	2.892263	3.022718
49	1	0	−4.317988	1.579630	3.366934
50	6	0	−5.345889	1.930559	−3.013529
51	1	0	−4.406755	2.206089	−3.507524
52	1	0	−5.467177	0.850083	−3.149612
53	1	0	−6.163544	2.438777	−3.532195
54	6	0	0.846925	4.674805	0.153681
55	6	0	0.138186	5.534122	1.023303
56	6	0	1.549083	5.241188	−0.934106
57	6	0	0.104357	6.904361	0.797904
58	1	0	−0.390995	5.107618	1.871167

59	6	0	1.518263	6.611804	-1.151355
60	1	0	2.134374	4.606402	-1.589465
61	6	0	0.792391	7.445195	-0.292293
62	1	0	-0.450543	7.552228	1.469801
63	1	0	2.066235	7.037681	-1.986536
64	1	0	0.772665	8.517180	-0.467835
65	6	0	3.903257	-0.109878	1.517034
66	1	0	4.979325	-0.070495	1.691662
67	6	0	3.741821	2.413395	1.367593
68	6	0	4.406132	3.517564	0.809565
69	6	0	3.228844	2.560878	2.673303
70	6	0	4.573539	4.705466	1.524547
71	1	0	4.808337	3.449484	-0.194559
72	6	0	3.398855	3.746774	3.389254
73	1	0	2.681267	1.749818	3.144318
74	6	0	4.075976	4.826419	2.821174
75	1	0	5.098823	5.536865	1.061139
76	1	0	3.000449	3.820405	4.398015
77	1	0	4.212782	5.747813	3.381028
78	6	0	4.309158	0.996869	-0.713266
79	6	0	5.707193	1.144357	-0.664771
80	6	0	3.737822	0.684790	-1.954623
81	6	0	6.492658	1.026728	-1.807899
82	1	0	6.192008	1.349245	0.284772
83	6	0	4.523503	0.562555	-3.105415
84	1	0	2.676788	0.484529	-2.046232
85	6	0	5.902249	0.742027	-3.041014
86	1	0	7.570443	1.142945	-1.730814
87	1	0	4.046387	0.311246	-4.049103
88	1	0	6.513532	0.644264	-3.934316
89	1	0	3.387319	-0.021697	2.474378
90	7	0	3.605328	-1.420607	0.975088
91	1	0	2.658476	-1.763580	1.090579
92	6	0	4.599370	-2.279987	0.590598
93	8	0	5.778537	-1.934205	0.522421
94	6	0	4.188739	-3.688443	0.244631
95	6	0	2.974279	-4.274020	0.630624
96	6	0	5.118320	-4.454944	-0.472137
97	6	0	2.698837	-5.600777	0.294988
98	1	0	2.240320	-3.720896	1.209174
99	6	0	4.836767	-5.774919	-0.814082
100	1	0	6.059925	-3.989978	-0.744022
101	6	0	3.624097	-6.352754	-0.429948
102	1	0	1.758402	-6.047209	0.607524

103	1	0	5.564079	-6.355672	-1.375730
104	1	0	3.404176	-7.385251	-0.690007
105	1	0	-0.512170	-2.208317	0.121882
106	8	0	0.470304	-2.041562	1.675882
107	16	0	0.077031	-1.010163	2.691627
108	8	0	1.187361	-0.512439	3.522383
109	8	0	-0.827343	0.035194	2.140720
110	6	0	-1.022056	-1.944238	3.866404
111	9	0	-0.346687	-2.910420	4.493552
112	9	0	-2.047994	-2.497523	3.199567
113	9	0	-1.524175	-1.102529	4.785785
114	1	0	1.714352	2.408067	-1.316299
115	6	0	1.949362	0.971744	0.352385
116	1	0	1.741173	0.070127	-0.225408
117	1	0	1.475105	0.849853	1.326315

Supplementary Table 28.

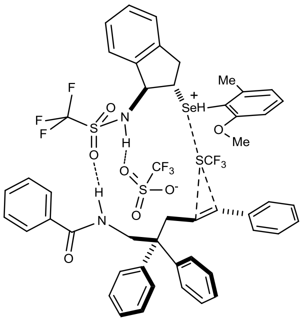

TS-I-RRR-b

B3LYP /6-31G(d) 195.15 K Thermal correction to Gibbs Free Energy = 0.819286

Thermal correction to Energy = 0.911017

M062x-D3/6-311+G(d,p) (IEFPCM, Dichloromethane) Energy = -7062.74008254

Standard orientation:

Center Number	Atomic Number	Atomic Type	Coordinates (Angstroms) X	Y	Z
1	6	0	0.403143	2.878213	0.283902
2	6	0	1.143482	2.043800	-0.571303
3	6	0	1.972370	0.907599	-0.012066
4	6	0	3.387885	1.333918	0.514075
5	16	0	-0.778085	0.990391	-1.182585
6	6	0	-0.489241	1.535135	-2.923733
7	9	0	-0.397841	2.876849	-3.006851
8	9	0	0.653715	1.029731	-3.425782

9	9	0	−1.482007	1.159310	−3.752768
10	1	0	0.068986	2.437924	1.221862
11	1	0	1.498271	2.480110	−1.500479
12	1	0	1.419885	0.455433	0.814980
13	1	0	2.089567	0.129034	−0.768046
14	34	0	−2.757526	−0.688142	−1.936456
15	6	0	−5.662610	−3.338700	1.868709
16	6	0	−4.647976	−2.843546	1.053552
17	6	0	−3.363251	−3.388670	1.124428
18	6	0	−3.061695	−4.428168	1.997458
19	6	0	−4.080427	−4.922963	2.816350
20	6	0	−5.369227	−4.383013	2.750957
21	1	0	−6.663006	−2.914714	1.832073
22	1	0	−2.055415	−4.833575	2.048388
23	1	0	−3.867733	−5.728010	3.514284
24	1	0	−6.148781	−4.772609	3.400107
25	6	0	−2.460008	−2.708986	0.111705
26	1	0	−2.447589	−3.298558	−0.812817
27	6	0	−3.206342	−1.372818	−0.133386
28	1	0	−2.919704	−0.637765	0.616768
29	6	0	−4.699882	−1.732989	0.023002
30	7	0	−1.064783	−2.566118	0.543815
31	16	0	0.149213	−2.919049	−0.495759
32	8	0	−0.280593	−2.803312	−1.891229
33	8	0	1.377220	−2.289165	−0.006848
34	6	0	0.390319	−4.752131	−0.218039
35	9	0	−0.744731	−5.401471	−0.510005
36	9	0	1.367928	−5.191417	−1.013275
37	9	0	0.710873	−4.986996	1.053643
38	1	0	−5.124543	−2.094645	−0.924639
39	1	0	−5.290033	−0.869479	0.340279
40	6	0	−4.162019	0.571694	−2.305465
41	6	0	−4.551236	1.538729	−1.354884
42	6	0	−4.757891	0.529092	−3.580129
43	6	0	−5.546314	2.468149	−1.681251
44	6	0	−5.748041	1.471690	−3.881842
45	6	0	−6.134679	2.425479	−2.943377
46	1	0	−5.855289	3.216778	−0.962096
47	1	0	−6.218511	1.450176	−4.860546
48	1	0	−6.906204	3.148316	−3.194161
49	8	0	−3.918554	1.492075	−0.159749
50	6	0	−4.224761	2.475529	0.832437
51	1	0	−3.598884	2.229364	1.687694
52	1	0	−3.984777	3.480250	0.464303

53	1	0	−5.282361	2.426160	1.117255
54	6	0	−4.352267	−0.494114	−4.616756
55	1	0	−3.304296	−0.373360	−4.914617
56	1	0	−4.456813	−1.516238	−4.235726
57	1	0	−4.970135	−0.397678	−5.513664
58	6	0	−0.012710	4.239892	0.072611
59	6	0	−0.891080	4.801912	1.029410
60	6	0	0.438856	5.045293	−0.998287
61	6	0	−1.317894	6.119819	0.904521
62	1	0	−1.223196	4.182233	1.857517
63	6	0	0.008175	6.358689	−1.114889
64	1	0	1.134204	4.642601	−1.726450
65	6	0	−0.872364	6.897901	−0.167087
66	1	0	−1.991376	6.543773	1.643757
67	1	0	0.361449	6.972870	−1.938065
68	1	0	−1.201745	7.929089	−0.262835
69	6	0	3.972987	0.091490	1.284184
70	1	0	4.930101	0.359448	1.733279
71	6	0	4.371390	1.686659	−0.617970
72	6	0	4.056038	1.567164	−1.978714
73	6	0	5.679761	2.088151	−0.293432
74	6	0	4.994990	1.861154	−2.972543
75	1	0	3.078946	1.221298	−2.295913
76	6	0	6.618006	2.383350	−1.279058
77	1	0	5.973371	2.166297	0.748350
78	6	0	6.278497	2.276440	−2.629132
79	1	0	4.715263	1.754015	−4.017523
80	1	0	7.620775	2.685303	−0.989074
81	1	0	7.009954	2.502358	−3.400459
82	6	0	3.192521	2.482279	1.531814
83	6	0	3.629001	3.796418	1.297121
84	6	0	2.482040	2.241649	2.723977
85	6	0	3.391199	4.815604	2.221688
86	1	0	4.163883	4.030604	0.383910
87	6	0	2.237757	3.258833	3.645910
88	1	0	2.080749	1.257607	2.939070
89	6	0	2.698689	4.552959	3.403279
90	1	0	3.748055	5.820403	2.009665
91	1	0	1.671193	3.029559	4.543541
92	1	0	2.512555	5.347465	4.121026
93	1	0	3.286533	−0.185610	2.089604
94	6	0	5.473619	−1.555061	0.236339
95	8	0	6.476312	−0.970503	0.643671
96	6	0	5.590238	−2.848509	−0.529382

97	6	0	4.522061	-3.484695	-1.177830
98	6	0	6.862734	-3.434136	-0.582907
99	6	0	4.723652	-4.688324	-1.853937
100	1	0	3.527948	-3.050622	-1.177328
101	6	0	7.063591	-4.635178	-1.258443
102	1	0	7.678918	-2.922686	-0.084110
103	6	0	5.992758	-5.267617	-1.894897
104	1	0	3.884998	-5.170600	-2.348617
105	1	0	8.055237	-5.079094	-1.289064
106	1	0	6.146938	-6.205596	-2.422194
107	1	0	-0.835692	-1.807954	1.214917
108	8	0	-0.565585	-0.168731	2.003013
109	16	0	-1.502038	0.546672	2.932802
110	8	0	-1.364426	2.021680	2.881691
111	8	0	-2.882218	0.027736	2.908026
112	6	0	-0.847679	0.092027	4.616040
113	9	0	-0.835834	-1.237647	4.768551
114	9	0	-1.607067	0.630175	5.576427
115	9	0	0.412116	0.545965	4.769132
116	7	0	4.207539	-1.077589	0.457779
117	1	0	3.410853	-1.666389	0.256152

Supplementary Table 29.

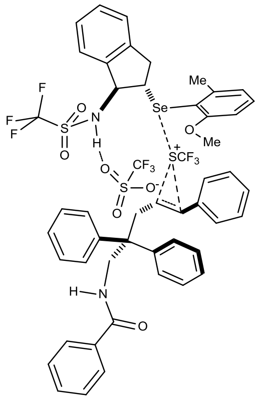

TS-I-SSS

B3LYP /6-31G(d) 195.15 K Thermal correction to Gibbs Free Energy = 0.818659

Thermal correction to Energy = 0.910916

M062x-D3/6-311+G(d,p) (IEFPCM, Dichloromethane) Energy = -7062.73997958

Standard orientation:

Center Number	Atomic Number	Atomic Type	Coordinates (Angstroms) X	Y	Z
1	6	0	1.174448	2.495420	0.218519

2	6	0	1.163129	1.075947	0.170551
3	6	0	2.166907	0.266286	-0.629493
4	6	0	3.618842	0.144289	-0.021283
5	16	0	-0.723398	1.173584	-0.815512
6	6	0	-0.198522	1.492257	-2.570048
7	9	0	-1.127594	2.247129	-3.195585
8	9	0	0.965960	2.166961	-2.672892
9	9	0	-0.070787	0.356881	-3.263125
10	1	0	1.603711	3.012424	-0.634002
11	1	0	0.801723	0.574172	1.067282
12	1	0	1.748326	-0.733418	-0.772631
13	1	0	2.300612	0.706041	-1.618452
14	34	0	-3.080266	0.329544	-2.089703
15	6	0	-7.092846	-2.253349	0.580827
16	6	0	-5.873997	-1.881112	0.017941
17	6	0	-4.817955	-2.794896	-0.033534
18	6	0	-4.950028	-4.085852	0.467385
19	6	0	-6.172315	-4.457421	1.033724
20	6	0	-7.234219	-3.548163	1.089301
21	1	0	-7.919259	-1.548689	0.634241
22	1	0	-4.116069	-4.780646	0.426008
23	1	0	-6.296608	-5.457437	1.439975
24	1	0	-8.176659	-3.848884	1.539268
25	6	0	-3.618073	-2.167870	-0.718605
26	1	0	-3.609635	-2.455206	-1.776655
27	6	0	-3.924796	-0.649364	-0.587511
28	1	0	-3.507166	-0.258120	0.340498
29	6	0	-5.466840	-0.561363	-0.605703
30	7	0	-2.336061	-2.590035	-0.155409
31	16	0	-1.037682	-2.848543	-1.107072
32	8	0	-1.268599	-2.282056	-2.438419
33	8	0	0.186457	-2.585119	-0.350946
34	6	0	-1.077948	-4.699486	-1.356430
35	9	0	-2.248802	-5.041566	-1.913281
36	9	0	-0.082365	-5.070337	-2.161775
37	9	0	-0.958461	-5.322951	-0.183411
38	1	0	-5.848002	-0.484274	-1.633992
39	1	0	-5.830933	0.313962	-0.060234
40	6	0	-4.043996	1.996234	-2.127347
41	6	0	-4.237175	2.753145	-0.953616
42	6	0	-4.492844	2.471966	-3.372345
43	6	0	-4.898198	3.984823	-1.020343
44	6	0	-5.147250	3.710004	-3.415854
45	6	0	-5.348172	4.451554	-2.254513

46	1	0	−5.056680	4.573685	−0.124598
47	1	0	−5.501126	4.088414	−4.370599
48	1	0	−5.860989	5.408256	−2.305938
49	8	0	−3.752243	2.208529	0.187684
50	6	0	−3.813030	2.952547	1.402376
51	1	0	−3.317928	2.328254	2.143854
52	1	0	−3.280879	3.906239	1.302404
53	1	0	−4.853070	3.141023	1.697355
54	6	0	−4.266717	1.690871	−4.646638
55	1	0	−3.197488	1.594815	−4.869728
56	1	0	−4.664018	0.672745	−4.569578
57	1	0	−4.746260	2.187059	−5.495008
58	6	0	0.667855	3.337351	1.274565
59	6	0	0.646591	4.734765	1.057326
60	6	0	0.221326	2.831532	2.517142
61	6	0	0.183464	5.598683	2.040819
62	1	0	0.999133	5.128749	0.107544
63	6	0	−0.245725	3.703678	3.494166
64	1	0	0.243097	1.767625	2.728378
65	6	0	−0.267789	5.082775	3.261498
66	1	0	0.172869	6.670070	1.862457
67	1	0	−0.596964	3.298874	4.438208
68	1	0	−0.630668	5.757268	4.032580
69	6	0	4.317930	−0.853985	−1.011475
70	1	0	3.759056	−1.796132	−0.957154
71	6	0	4.249755	1.548477	−0.033317
72	6	0	4.944656	2.041751	−1.149225
73	6	0	4.050702	2.423734	1.050475
74	6	0	5.419982	3.354997	−1.176802
75	1	0	5.154351	1.402197	−1.999815
76	6	0	4.523820	3.737475	1.021936
77	1	0	3.539768	2.068662	1.939380
78	6	0	5.210157	4.211445	−0.095490
79	1	0	5.964513	3.702543	−2.050877
80	1	0	4.355051	4.384884	1.878527
81	1	0	5.585127	5.231287	−0.119975
82	6	0	3.671428	−0.517198	1.370328
83	6	0	2.668933	−1.389367	1.816756
84	6	0	4.809638	−0.357592	2.184764
85	6	0	2.782004	−2.052793	3.040865
86	1	0	1.780845	−1.571511	1.223338
87	6	0	4.931613	−1.033500	3.399159
88	1	0	5.600828	0.318270	1.874552
89	6	0	3.913138	−1.882206	3.835938

90	1	0	1.967118	-2.690114	3.368934
91	1	0	5.820650	-0.885008	4.007109
92	1	0	3.998092	-2.397847	4.788701
93	1	0	4.261334	-0.499071	-2.041604
94	7	0	5.717728	-1.109947	-0.740182
95	1	0	5.958696	-1.440805	0.183333
96	6	0	6.710451	-0.886597	-1.652788
97	8	0	6.498018	-0.379341	-2.755178
98	6	0	8.099445	-1.280769	-1.230389
99	6	0	8.368809	-2.239179	-0.242989
100	6	0	9.169336	-0.663570	-1.892373
101	6	0	9.685664	-2.558554	0.088959
102	1	0	7.558439	-2.769862	0.249982
103	6	0	10.483677	-0.976665	-1.554873
104	1	0	8.943654	0.057464	-2.670896
105	6	0	10.745093	-1.923360	-0.561042
106	1	0	9.882760	-3.309548	0.849112
107	1	0	11.305662	-0.485602	-2.068776
108	1	0	11.770478	-2.171479	-0.300030
109	1	0	-2.125689	-2.397527	0.843415
110	8	0	-1.853503	-2.028652	2.580762
111	16	0	-1.481222	-0.613420	2.844140
112	8	0	-0.105679	-0.387261	3.334940
113	8	0	-1.909153	0.330860	1.774507
114	6	0	-2.565882	-0.125761	4.276243
115	9	0	-3.859343	-0.304175	3.971836
116	9	0	-2.386836	1.184949	4.555295
117	9	0	-2.280081	-0.831585	5.373628

Supplementary Table 30.

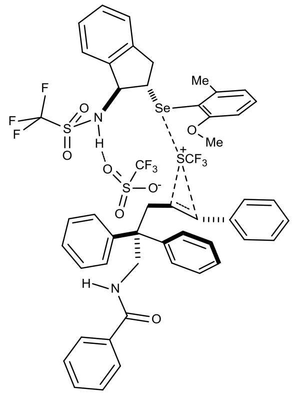

TS-I-SRR

B3LYP /6-31G(d) 195.15 K Thermal correction to Gibbs Free Energy = 0.818389

Thermal correction to Energy = 0.911083

M062x-D3/6-311+G(d,p) (IEFPCM, Dichloromethane) Energy = -7062.73668321065

Standard orientation:

Center Number	Atomic Number	Atomic Type	Coordinates (Angstroms) X	Y	Z
1	6	0	1.181285	1.797275	0.786498
2	6	0	1.445208	1.137359	-0.426364
3	6	0	1.783540	-0.339404	-0.443961
4	6	0	3.294255	-0.618430	-0.084435
5	16	0	-0.769816	1.210855	-0.946392
6	6	0	-0.347732	2.202237	-2.448535
7	9	0	0.307934	3.334241	-2.120794
8	9	0	0.444652	1.516750	-3.291255
9	9	0	-1.439275	2.574491	-3.144631
10	1	0	0.753150	1.190027	1.583166
11	1	0	1.885128	1.727515	-1.225976
12	1	0	1.135790	-0.871617	0.256304
13	1	0	1.564385	-0.728032	-1.441104
14	34	0	-3.341440	0.935402	-1.742939
15	6	0	-6.876392	-1.572550	1.610651
16	6	0	-5.816688	-1.251759	0.766518
17	6	0	-4.934768	-2.248236	0.338061
18	6	0	-5.086478	-3.572085	0.734611
19	6	0	-6.149266	-3.892310	1.584186
20	6	0	-7.036266	-2.901058	2.016790
21	1	0	-7.561423	-0.804299	1.960693
22	1	0	-4.387614	-4.332640	0.399141
23	1	0	-6.283168	-4.917997	1.916830
24	1	0	-7.852870	-3.164260	2.683791
25	6	0	-3.908068	-1.649023	-0.605045
26	1	0	-4.260476	-1.764175	-1.637057
27	6	0	-3.953005	-0.152441	-0.207933
28	1	0	-3.301734	0.031351	0.644625
29	6	0	-5.424510	0.093617	0.185531
30	7	0	-2.576280	-2.256653	-0.543346
31	16	0	-1.773648	-2.609709	-1.930451
32	8	0	-2.316598	-1.850502	-3.060008
33	8	0	-0.341790	-2.672153	-1.652070
34	6	0	-2.307585	-4.373776	-2.238149
35	9	0	-3.641625	-4.420573	-2.363145
36	9	0	-1.743738	-4.820921	-3.360469
37	9	0	-1.941947	-5.151112	-1.217507
38	1	0	-6.037124	0.337622	-0.694582

39	1	0	−5. 520688	0. 919483	0. 894871
40	6	0	−4. 003851	2. 697391	−1. 354571
41	6	0	−3. 852887	3. 278791	−0. 077705
42	6	0	−4. 608902	3. 411300	−2. 406662
43	6	0	−4. 312077	4. 582085	0. 148017
44	6	0	−5. 056901	4. 713535	−2. 154693
45	6	0	−4. 909150	5. 287521	−0. 894425
46	1	0	−4. 201001	5. 041969	1. 122366
47	1	0	−5. 528294	5. 275159	−2. 955985
48	1	0	−5. 263596	6. 299164	−0. 716416
49	8	0	−3. 262908	2. 509326	0. 865936
50	6	0	−3. 076112	3. 035372	2. 182294
51	1	0	−2. 622729	2. 228287	2. 754446
52	1	0	−2. 409574	3. 905944	2. 158586
53	1	0	−4. 037136	3. 315082	2. 630075
54	6	0	−4. 779566	2. 812310	−3. 784652
55	1	0	−3. 812934	2. 618087	−4. 263838
56	1	0	−5. 310022	1. 854436	−3. 744517
57	1	0	−5. 344052	3. 490649	−4. 430323
58	6	0	1. 354268	3. 188108	1. 112712
59	6	0	0. 849219	3. 624232	2. 360852
60	6	0	2. 036096	4. 113031	0. 288347
61	6	0	1. 003018	4. 948352	2. 758174
62	1	0	0. 341879	2. 904014	2. 996438
63	6	0	2. 185591	5. 431275	0. 693077
64	1	0	2. 458515	3. 792505	−0. 657431
65	6	0	1. 667356	5. 852523	1. 925306
66	1	0	0. 613824	5. 275225	3. 718128
67	1	0	2. 715016	6. 135084	0. 057650
68	1	0	1. 793169	6. 885882	2. 237469
69	6	0	4. 138488	−0. 029143	−1. 260264
70	1	0	3. 715820	−0. 432672	−2. 190161
71	6	0	3. 569676	−2. 139974	−0. 033268
72	6	0	4. 676297	−2. 622692	0. 690791
73	6	0	2. 803724	−3. 070172	−0. 750824
74	6	0	5. 008127	−3. 977979	0. 690288
75	1	0	5. 273223	−1. 930876	1. 277129
76	6	0	3. 130674	−4. 428659	−0. 743182
77	1	0	1. 927683	−2. 760462	−1. 305757
78	6	0	4. 234813	−4. 890351	−0. 028976
79	1	0	5. 866591	−4. 318922	1. 263485
80	1	0	2. 507309	−5. 124994	−1. 297797
81	1	0	4. 485133	−5. 947890	−0. 024013
82	6	0	3. 589617	0. 021705	1. 283341

83	6	0	4.446387	1.118303	1.461378
84	6	0	2.925287	-0.488717	2.414792
85	6	0	4.634125	1.676765	2.730041
86	1	0	4.975166	1.556529	0.621551
87	6	0	3.100893	0.077201	3.674602
88	1	0	2.250963	-1.332682	2.309276
89	6	0	3.964624	1.163005	3.839132
90	1	0	5.307275	2.522930	2.841948
91	1	0	2.549346	-0.327110	4.517888
92	1	0	4.109124	1.604924	4.821573
93	1	0	4.056609	1.057932	-1.306316
94	6	0	6.520858	0.646696	-1.313829
95	8	0	6.249425	1.849489	-1.342260
96	6	0	7.945948	0.167562	-1.352248
97	6	0	8.321431	-1.120710	-1.758913
98	6	0	8.940494	1.083775	-0.984105
99	6	0	9.666386	-1.490613	-1.776746
100	1	0	7.575191	-1.834958	-2.096663
101	6	0	10.282365	0.711510	-0.994489
102	1	0	8.635606	2.084164	-0.695668
103	6	0	10.648609	-0.578015	-1.388453
104	1	0	9.946175	-2.488990	-2.101680
105	1	0	11.044416	1.427434	-0.698494
106	1	0	11.695879	-0.868036	-1.400558
107	1	0	-1.961920	-1.995619	0.248755
108	8	0	-0.958827	-1.053099	1.500960
109	16	0	-1.380504	-0.481308	2.821553
110	8	0	-0.562032	0.680275	3.245668
111	8	0	-2.839550	-0.326020	2.973540
112	6	0	-0.914889	-1.819239	4.030566
113	9	0	-1.529662	-2.967048	3.720045
114	9	0	-1.262551	-1.466845	5.273861
115	9	0	0.415070	-2.033924	4.009151
116	7	0	5.560296	-0.319156	-1.222311
117	1	0	5.835450	-1.282613	-1.093928

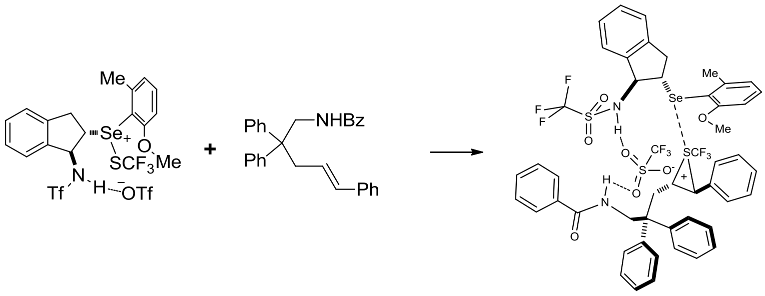

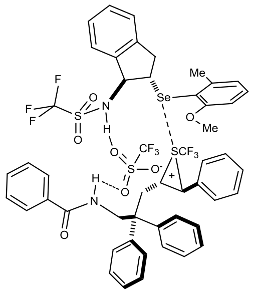

int-III-RSS-a

$\Delta G_{195.15\ K}$ = -6.7 kcal/mol

$\Delta\Delta G_{195.15\ K}$ = 0.0 kcal/mol

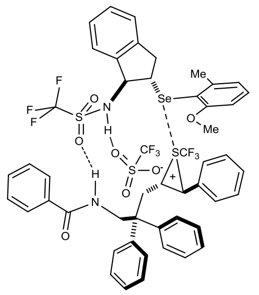

int-III-RSS-b

$\Delta G_{195.15\ K}$ = -4.9 kcal/mol

$\Delta\Delta G_{195.15\ K}$ = 1.8 kcal/mol

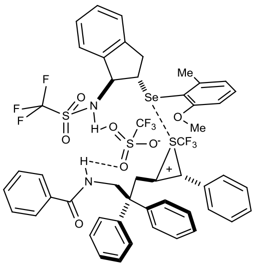

int-III-RRR-a

$\Delta G_{195.15\ K}$ = - 9.3 kcal/mol

$\Delta\Delta G_{195.15\ K}$ = -2.6 kcal/mol

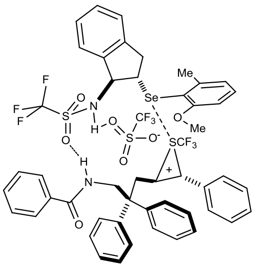

int-III-RRR-b

$\Delta G_{195.15\ K}$ = -8.2 kcal/mol

$\Delta\Delta G_{195.15\ K}$ = -1.4 kcal/mol

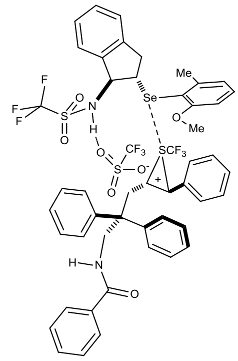

int-III-SSS

$\Delta G_{195.15\ K}$ = -5.4 kcal/mol

$\Delta\Delta G_{195.15\ K}$ = 1.2 kcal/mol

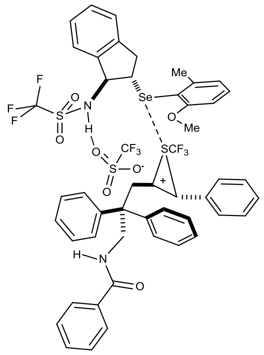

int-III-SRR

$\Delta G_{195.15\ K}$ = -6.2 kcal/mol

$\Delta\Delta G_{195.15\ K}$ = 0.5 kcal/mol

Supplementary Figure 181. DFT calculations for **int-III** of **1a**; ΔG related to **int-I**.

Supplementary Table 31.

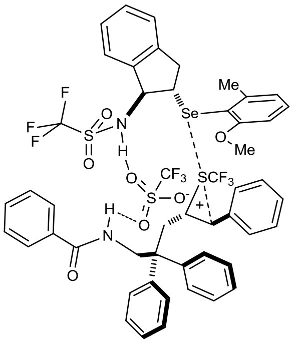

int-III-RSS-a

B3LYP /6-31G(d) 195.15 K Thermal correction to Gibbs Free Energy = 0.81852

Thermal correction to Energy = 0.911989

M062x-D3/6-311+G(d,p) (IEFPCM, Dichloromethane) Energy = -7062.75289964

Standard orientation:

Center Number	Atomic Number	Atomic Type	Coordinates (Angstroms) X	Y	Z
1	6	0	-1.197943	-3.026349	0.371110
2	6	0	-1.284844	-1.621007	0.046238
3	6	0	-2.271347	-1.053139	-0.969691
4	6	0	-3.770857	-1.192563	-0.540817
5	16	0	0.593836	-1.698913	-0.531078
6	6	0	0.430400	-2.292692	-2.284410
7	9	0	1.558077	-2.935619	-2.634377
8	9	0	-0.586986	-3.161927	-2.466567
9	9	0	0.249953	-1.276402	-3.129346
10	1	0	-1.389632	-3.730938	-0.430920
11	1	0	-1.174810	-0.956545	0.904100
12	1	0	-2.019317	0.003335	-1.079255
13	1	0	-2.151450	-1.523962	-1.945342
14	34	0	3.204322	-0.624359	-1.651308
15	6	0	6.212327	3.000506	1.151876
16	6	0	5.208235	2.340516	0.446731
17	6	0	4.093096	3.048873	-0.010885
18	6	0	3.956044	4.414103	0.218277
19	6	0	4.963050	5.074077	0.927893
20	6	0	6.081469	4.372295	1.390173
21	1	0	7.080573	2.459447	1.520444
22	1	0	3.080549	4.948398	-0.139587
23	1	0	4.873469	6.138363	1.127455
24	1	0	6.853466	4.897272	1.946558
25	6	0	3.165015	2.119388	-0.770326

26	1	0	3.360779	2.198767	−1.846184
27	6	0	3.606728	0.721439	−0.257919
28	1	0	3.044448	0.458762	0.638817
29	6	0	5.110901	0.880733	0.049259
30	7	0	1.744530	2.407408	−0.580127
31	16	0	0.673231	2.342258	−1.805174
32	8	0	1.277399	1.689013	−2.966799
33	8	0	−0.638758	1.951663	−1.286110
34	6	0	0.497015	4.138642	−2.288706
35	9	0	1.694830	4.613874	−2.656835
36	9	0	−0.357948	4.260859	−3.302112
37	9	0	0.057066	4.848836	−1.244357
38	1	0	5.717375	0.678501	−0.844678
39	1	0	5.444620	0.190499	0.829588
40	6	0	4.301498	−2.126518	−1.150137
41	6	0	4.318194	−2.610043	0.173929
42	6	0	5.041018	−2.775948	−2.154704
43	6	0	5.092079	−3.727853	0.503004
44	6	0	5.802382	−3.900063	−1.806267
45	6	0	5.830235	−4.364412	−0.494471
46	1	0	5.117224	−4.100923	1.520183
47	1	0	6.379044	−4.407323	−2.574680
48	1	0	6.431211	−5.233214	−0.239616
49	8	0	3.552027	−1.926847	1.062821
50	6	0	3.451133	−2.390567	2.405646
51	1	0	2.755048	−1.705475	2.887650
52	1	0	3.053738	−3.412863	2.439424
53	1	0	4.425746	−2.361357	2.909459
54	6	0	5.011639	−2.298138	−3.588052
55	1	0	4.013071	−2.415676	−4.026646
56	1	0	5.258738	−1.233257	−3.658613
57	1	0	5.718660	−2.864131	−4.201261
58	6	0	−0.903329	−3.594985	1.658241
59	6	0	−0.770239	−5.001442	1.748488
60	6	0	−0.775085	−2.818295	2.834861
61	6	0	−0.514654	−5.611185	2.968720
62	1	0	−0.876149	−5.602190	0.849250
63	6	0	−0.513824	−3.436865	4.050734
64	1	0	−0.886649	−1.741513	2.801188
65	6	0	−0.382160	−4.828290	4.121738
66	1	0	−0.417958	−6.691210	3.027610
67	1	0	−0.411890	−2.828123	4.943572
68	1	0	−0.180553	−5.304492	5.077465
69	6	0	−4.015768	−0.460442	0.818888

70	1	0	−5.062339	−0.584286	1.107026
71	6	0	−4.100979	−2.696702	−0.427003
72	6	0	−4.067179	−3.492483	−1.588994
73	6	0	−4.418437	−3.331547	0.783228
74	6	0	−4.320056	−4.860324	−1.541041
75	1	0	−3.860199	−3.024164	−2.547151
76	6	0	−4.680940	−4.705550	0.833907
77	1	0	−4.472887	−2.763205	1.704633
78	6	0	−4.627706	−5.477179	−0.323523
79	1	0	−4.289658	−5.444889	−2.456937
80	1	0	−4.930234	−5.165051	1.786749
81	1	0	−4.834784	−6.543288	−0.284944
82	6	0	−4.684104	−0.556679	−1.614999
83	6	0	−6.064912	−0.808965	−1.562178
84	6	0	−4.211901	0.289981	−2.625931
85	6	0	−6.939379	−0.247090	−2.487592
86	1	0	−6.462362	−1.453252	−0.783446
87	6	0	−5.088055	0.853281	−3.558856
88	1	0	−3.159337	0.537652	−2.700780
89	6	0	−6.453080	0.586402	−3.496872
90	1	0	−8.003183	−0.457338	−2.416204
91	1	0	−4.692769	1.507554	−4.331509
92	1	0	−7.133123	1.027024	−4.221067
93	1	0	−3.391204	−0.904226	1.601265
94	7	0	−3.743967	0.963445	0.784947
95	1	0	−2.831341	1.249659	1.125867
96	6	0	−4.779272	1.866374	0.873745
97	8	0	−5.958736	1.519785	0.874924
98	6	0	−4.387331	3.313828	0.968104
99	6	0	−3.131119	3.799550	0.579037
100	6	0	−5.352530	4.210075	1.447695
101	6	0	−2.844583	5.161135	0.684517
102	1	0	−2.381970	3.131921	0.165390
103	6	0	−5.060835	5.566781	1.560482
104	1	0	−6.325803	3.817495	1.723265
105	6	0	−3.803930	6.045264	1.180042
106	1	0	−1.872005	5.527037	0.369581
107	1	0	−5.813067	6.252846	1.941376
108	1	0	−3.576112	7.105022	1.263598
109	1	0	1.336526	2.390876	0.371471
110	8	0	0.640928	2.327109	2.042178
111	16	0	0.227606	0.980830	2.506314
112	8	0	−1.215774	0.660643	2.330768
113	8	0	1.131681	−0.125154	2.096494

114	6	0	0.425912	1.050616	4.355635
115	9	0	1.707014	1.259100	4.682422
116	9	0	0.042229	-0.126294	4.894228
117	9	0	-0.321041	2.020791	4.884548

Supplementary Table 32.

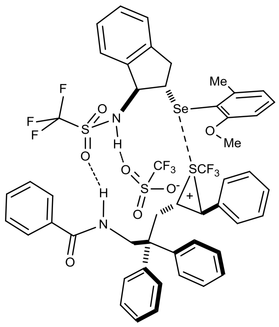

int-III-RSS-b

B3LYP /6-31G(d) 195.15 K Thermal correction to Gibbs Free Energy = 0.819634

Thermal correction to Energy = 0.911867

M062x-D3/6-311+G(d,p) (IEFPCM, Dichloromethane) Energy = -7062.75114784

Standard orientation:

Center Number	Atomic Number	Atomic Type	Coordinates (Angstroms) X	Y	Z
1	6	0	-0.727268	-3.221860	-0.265982
2	6	0	-1.032320	-1.807216	-0.229675
3	6	0	-2.221511	-1.266923	-1.018713
4	6	0	-3.594694	-1.444089	-0.283433
5	16	0	0.741334	-1.422238	-0.986121
6	6	0	0.390651	-1.567952	-2.803133
7	9	0	1.546173	-1.757877	-3.460878
8	9	0	-0.406418	-2.617248	-3.113378
9	9	0	-0.193359	-0.468434	-3.287834
10	1	0	-0.922209	-3.723481	-1.208186
11	1	0	-0.918642	-1.342237	0.749675
12	1	0	-2.050926	-0.204762	-1.210483
13	1	0	-2.275173	-1.773423	-1.983917
14	34	0	3.115243	0.278938	-1.902326
15	6	0	5.852134	3.443107	1.633427
16	6	0	4.879547	2.839603	0.839077
17	6	0	3.629876	3.446167	0.681306
18	6	0	3.326005	4.653996	1.301260
19	6	0	4.301832	5.256453	2.099892

20	6	0	5.554334	4.655249	2.263867
21	1	0	6.825100	2.977245	1.770334
22	1	0	2.348447	5.109953	1.172522
23	1	0	4.083623	6.194922	2.602067
24	1	0	6.301230	5.131582	2.893456
25	6	0	2.763964	2.616696	-0.248212
26	1	0	2.816777	3.025022	-1.264464
27	6	0	3.460003	1.228349	-0.200683
28	1	0	3.040709	0.631829	0.609438
29	6	0	4.949700	1.550632	0.044904
30	7	0	1.347441	2.602106	0.127166
31	16	0	0.166772	2.633799	-0.989915
32	8	0	0.709136	2.363419	-2.321552
33	8	0	-1.011347	1.927424	-0.475612
34	6	0	-0.324769	4.436132	-1.030978
35	9	0	0.753740	5.181989	-1.302452
36	9	0	-1.241940	4.626740	-1.986838
37	9	0	-0.827664	4.805602	0.145123
38	1	0	5.478431	1.706783	-0.905771
39	1	0	5.461626	0.738854	0.570128
40	6	0	4.417427	-1.140353	-1.912503
41	6	0	4.631911	-1.953925	-0.780698
42	6	0	5.111866	-1.393072	-3.109779
43	6	0	5.554313	-3.004210	-0.835082
44	6	0	6.023867	-2.456846	-3.146384
45	6	0	6.244664	-3.246862	-2.022214
46	1	0	5.729845	-3.628469	0.033098
47	1	0	6.566003	-2.657146	-4.066362
48	1	0	6.960483	-4.063386	-2.064976
49	8	0	3.899117	-1.644050	0.319804
50	6	0	4.011399	-2.451232	1.487063
51	1	0	3.308925	-2.020845	2.199054
52	1	0	3.734059	-3.491410	1.274079
53	1	0	5.030661	-2.421928	1.893229
54	6	0	4.882703	-0.555714	-4.346956
55	1	0	3.863830	-0.681330	-4.733602
56	1	0	5.005009	0.511782	-4.133418
57	1	0	5.580936	-0.837030	-5.140507
58	6	0	-0.195851	-4.044024	0.778540
59	6	0	0.143042	-5.382197	0.457935
60	6	0	-0.024833	-3.592229	2.109917
61	6	0	0.646836	-6.236194	1.427694
62	1	0	0.005603	-5.734002	-0.561095
63	6	0	0.485428	-4.453818	3.072304

64	1	0	−0.304213	−2.582994	2.389382
65	6	0	0.822896	−5.769917	2.736325
66	1	0	0.903425	−7.260080	1.172778
67	1	0	0.620453	−4.094277	4.087310
68	1	0	1.219453	−6.437009	3.497140
69	6	0	−3.681663	−0.467462	0.937245
70	1	0	−4.568564	−0.711917	1.524276
71	6	0	−3.713171	−2.912072	0.178497
72	6	0	−3.844283	−3.927505	−0.789171
73	6	0	−3.675672	−3.298294	1.526854
74	6	0	−3.927119	−5.270390	−0.426237
75	1	0	−3.912832	−3.654224	−1.838896
76	6	0	−3.764963	−4.644503	1.893010
77	1	0	−3.574262	−2.554950	2.309453
78	6	0	−3.887245	−5.636213	0.921554
79	1	0	−4.038322	−6.029768	−1.196192
80	1	0	−3.738978	−4.912372	2.945881
81	1	0	−3.960860	−6.681450	1.209479
82	6	0	−4.755724	−1.119897	−1.251136
83	6	0	−6.068746	−1.429352	−0.860648
84	6	0	−4.572472	−0.494065	−2.490868
85	6	0	−7.154468	−1.136890	−1.681640
86	1	0	−6.245081	−1.900124	0.101427
87	6	0	−5.660586	−0.200236	−3.318165
88	1	0	−3.581070	−0.216220	−2.831671
89	6	0	−6.955490	−0.522792	−2.919847
90	1	0	−8.158873	−1.384176	−1.348687
91	1	0	−5.487090	0.286892	−4.274259
92	1	0	−7.801779	−0.293092	−3.561738
93	1	0	−2.805358	−0.574478	1.583442
94	7	0	−3.820832	0.932290	0.574132
95	1	0	−2.974919	1.463675	0.410771
96	6	0	−4.982742	1.606117	0.862976
97	8	0	−5.969087	1.042551	1.334994
98	6	0	−5.001660	3.083259	0.579770
99	6	0	−4.104975	3.725387	−0.284443
100	6	0	−6.000270	3.837058	1.212089
101	6	0	−4.198721	5.101334	−0.498567
102	1	0	−3.345759	3.157651	−0.812087
103	6	0	−6.089018	5.210588	1.002559
104	1	0	−6.697031	3.319370	1.862676
105	6	0	−5.186210	5.846744	0.146666
106	1	0	−3.499345	5.587142	−1.171871
107	1	0	−6.862798	5.785848	1.504271

108	1	0	-5.255260	6.918693	-0.020392
109	1	0	1.062659	2.253191	1.062304
110	8	0	0.561014	1.636506	2.682459
111	16	0	0.507435	0.155961	2.774197
112	8	0	-0.846085	-0.437286	2.897105
113	8	0	1.380499	-0.548811	1.796374
114	6	0	1.310400	-0.228204	4.409037
115	9	0	2.552998	0.269750	4.450907
116	9	0	1.396961	-1.568281	4.564399
117	9	0	0.608705	0.270083	5.428731

Supplementary Table 33.

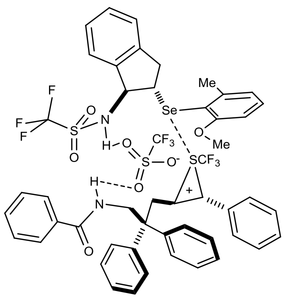

int-III-RRR-a

B3LYP /6-31G(d) 195.15 K Thermal correction to Gibbs Free Energy = 0.82033

Thermal correction to Energy = 0.912368

M062x-D3/6-311+G(d,p) (IEFPCM, Dichloromethane) Energy = -7062.75882962

Standard orientation:

Center Number	Atomic Number	Atomic Type	Coordinates (Angstroms) X	Y	Z
1	6	0	0.226794	2.808686	0.579550
2	6	0	1.262520	2.141964	-0.216239
3	6	0	3.714194	1.560514	0.358855
4	16	0	-0.507138	1.429966	-0.591653
5	6	0	-0.939267	2.402179	-2.139263
6	9	0	-2.116021	3.010136	-1.971180
7	9	0	-0.029195	3.328715	-2.462390
8	9	0	-1.033363	1.532813	-3.140938
9	1	0	0.044911	2.345147	1.550156
10	34	0	-3.366018	0.152653	-1.382959
11	6	0	-5.053395	-4.271130	1.405532
12	6	0	-4.347804	-3.347513	0.637672
13	6	0	-3.184745	-3.738275	-0.034504

14	6	0	−2.709358	−5.043447	0.038128
15	6	0	−3.417623	−5.968148	0.810841
16	6	0	−4.579059	−5.584106	1.489219
17	1	0	−5.953809	−3.977318	1.939756
18	1	0	−1.804752	−5.330894	−0.490148
19	1	0	−3.059889	−6.991063	0.890198
20	1	0	−5.115176	−6.312028	2.092418
21	6	0	−2.621290	−2.564872	−0.814794
22	1	0	−2.961519	−2.610741	−1.855906
23	6	0	−3.281258	−1.353022	−0.104162
24	1	0	−2.668929	−1.049871	0.746005
25	6	0	−4.648606	−1.884869	0.370493
26	7	0	−1.160126	−2.508016	−0.850915
27	16	0	−0.336861	−2.094467	−2.195991
28	8	0	−1.272745	−1.655197	−3.232429
29	8	0	0.841311	−1.301280	−1.834459
30	6	0	0.341470	−3.732847	−2.785565
31	9	0	−0.674770	−4.586697	−2.972566
32	9	0	0.992366	−3.564526	−3.935750
33	9	0	1.169981	−4.236338	−1.870159
34	1	0	−5.401834	−1.779653	−0.422533
35	1	0	−5.022883	−1.345170	1.246076
36	6	0	−4.585107	1.377294	−0.521454
37	6	0	−4.418841	1.762241	0.824075
38	6	0	−5.635595	1.920354	−1.283553
39	6	0	−5.320086	2.650855	1.420575
40	6	0	−6.516008	2.826252	−0.674786
41	6	0	−6.365713	3.177694	0.662384
42	1	0	−5.209679	2.934647	2.460710
43	1	0	−7.331210	3.244876	−1.258414
44	1	0	−7.064971	3.868794	1.125348
45	8	0	−3.343140	1.231894	1.477129
46	6	0	−3.244650	1.401324	2.890132
47	1	0	−2.372497	0.824640	3.192345
48	1	0	−3.099272	2.456218	3.158493
49	1	0	−4.142536	1.023985	3.394566
50	6	0	−5.824074	1.550782	−2.736241
51	1	0	−4.990626	1.910373	−3.352553
52	1	0	−5.855719	0.463802	−2.866081
53	1	0	−6.747537	1.983145	−3.132229
54	6	0	−0.221915	4.216531	0.434713
55	6	0	−1.512843	4.571976	0.857807
56	6	0	0.643227	5.204574	−0.053885
57	6	0	−1.935516	5.895153	0.780880

58	1	0	−2.188699	3.801883	1.218420
59	6	0	0.218935	6.532056	−0.115088
60	1	0	1.654397	4.946906	−0.351229
61	6	0	−1.068523	6.878842	0.295377
62	1	0	−2.940989	6.157943	1.096169
63	1	0	0.899490	7.294434	−0.482751
64	1	0	−1.397131	7.912880	0.239536
65	6	0	4.451139	0.614633	1.385300
66	1	0	5.527480	0.783643	1.329401
67	6	0	3.823120	3.015534	0.882853
68	6	0	4.334410	4.073767	0.115268
69	6	0	3.358252	3.324285	2.176408
70	6	0	4.403875	5.374555	0.624248
71	1	0	4.696701	3.883925	−0.888198
72	6	0	3.423911	4.620929	2.685161
73	1	0	2.935713	2.545279	2.804449
74	6	0	3.953649	5.655788	1.912690
75	1	0	4.817911	6.166284	0.004743
76	1	0	3.061538	4.819677	3.690364
77	1	0	4.012486	6.665178	2.310558
78	6	0	4.346481	1.377225	−1.034623
79	6	0	5.721272	1.621677	−1.207285
80	6	0	3.625946	0.937981	−2.154179
81	6	0	6.341047	1.458295	−2.442777
82	1	0	6.322600	1.939818	−0.361418
83	6	0	4.245425	0.770289	−3.397107
84	1	0	2.576028	0.678787	−2.081247
85	6	0	5.602963	1.034920	−3.550103
86	1	0	7.406866	1.648525	−2.535495
87	1	0	3.656259	0.417915	−4.239525
88	1	0	6.085920	0.899723	−4.514108
89	1	0	4.117679	0.865871	2.395216
90	7	0	4.210403	−0.797296	1.172563
91	1	0	3.417786	−1.190709	1.670888
92	6	0	5.199256	−1.633362	0.714452
93	8	0	6.310557	−1.221343	0.386645
94	6	0	4.848911	−3.094147	0.635965
95	6	0	3.534405	−3.581494	0.668445
96	6	0	5.911915	−3.998339	0.503045
97	6	0	3.294021	−4.953393	0.584301
98	1	0	2.684893	−2.910168	0.739989
99	6	0	5.669526	−5.367273	0.424942
100	1	0	6.920438	−3.600428	0.461453
101	6	0	4.358154	−5.848645	0.467283

102	1	0	2.270148	-5.315637	0.607210
103	1	0	6.501830	-6.059908	0.329268
104	1	0	4.167402	-6.917146	0.404770
105	1	0	-0.633540	-2.424481	0.036983
106	8	0	0.241233	-2.394183	1.588677
107	16	0	0.368937	-1.197307	2.463500
108	8	0	1.740446	-0.932462	2.949647
109	8	0	-0.363395	0.002640	1.963397
110	6	0	-0.599312	-1.656301	3.984263
111	9	0	-0.067116	-2.714053	4.597401
112	9	0	-1.867718	-1.942137	3.644211
113	9	0	-0.623263	-0.622652	4.843323
114	1	0	1.612197	2.673914	-1.096026
115	6	0	2.215117	1.126791	0.389822
116	1	0	2.120269	0.166559	-0.121547
117	1	0	1.917596	0.949906	1.422408

Supplementary Table 34.

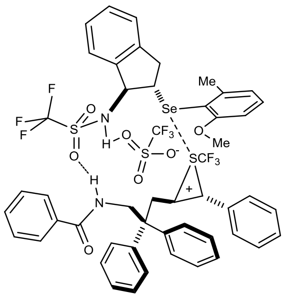

int-III-RRR-b

B3LYP /6-31G(d) 195.15 K Thermal correction to Gibbs Free Energy = 0.819007

Thermal correction to Energy = 0.912292

M062x-D3/6-311+G(d,p) (IEFPCM, Dichloromethane) Energy = -7062.7558044

Standard orientation:

Center Number	Atomic Number	Atomic Type	Coordinates (Angstroms) X	Y	Z
1	6	0	0.621157	2.819437	0.267681
2	6	0	1.204178	1.937977	-0.733216
3	6	0	2.046281	0.771822	-0.203903
4	6	0	3.479051	1.160926	0.276439
5	16	0	-0.493137	1.168236	-1.240943
6	6	0	-1.046252	2.406926	-2.490209
7	9	0	-1.774562	3.396751	-1.946720
8	9	0	-0.006024	2.987780	-3.127930
9	9	0	-1.797981	1.798587	-3.411577

10	1	0	0.230942	2.332929	1.159992
11	1	0	1.640593	2.431288	-1.597642
12	1	0	1.506301	0.318396	0.631808
13	1	0	2.111915	0.002515	-0.975340
14	34	0	-2.945810	-0.787903	-2.006491
15	6	0	-5.637349	-3.097479	2.190497
16	6	0	-4.678688	-2.686746	1.267730
17	6	0	-3.399713	-3.251188	1.288850
18	6	0	-3.052576	-4.226611	2.217477
19	6	0	-4.015373	-4.635675	3.144623
20	6	0	-5.296980	-4.076058	3.129678
21	1	0	-6.631589	-2.657296	2.191632
22	1	0	-2.052213	-4.649315	2.229860
23	1	0	-3.763703	-5.388632	3.886587
24	1	0	-6.033163	-4.398077	3.861526
25	6	0	-2.565480	-2.664933	0.164214
26	1	0	-2.615986	-3.320675	-0.713191
27	6	0	-3.308529	-1.341278	-0.141747
28	1	0	-2.958197	-0.561865	0.533570
29	6	0	-4.788442	-1.662022	0.156370
30	7	0	-1.141326	-2.499679	0.484496
31	16	0	0.018747	-3.007540	-0.538250
32	8	0	-0.471986	-3.093934	-1.912599
33	8	0	1.267755	-2.312825	-0.205058
34	6	0	0.302719	-4.781268	-0.021325
35	9	0	-0.830210	-5.478135	-0.174821
36	9	0	1.258343	-5.317752	-0.784530
37	9	0	0.675400	-4.835378	1.258550
38	1	0	-5.280544	-2.091805	-0.727236
39	1	0	-5.350651	-0.768400	0.441850
40	6	0	-4.394995	0.439476	-2.329438
41	6	0	-4.611085	1.530937	-1.462620
42	6	0	-5.199118	0.269225	-3.469695
43	6	0	-5.644618	2.437874	-1.718239
44	6	0	-6.223090	1.195476	-3.714754
45	6	0	-6.444135	2.260633	-2.847312
46	1	0	-5.822223	3.274933	-1.053423
47	1	0	-6.852680	1.069227	-4.591201
48	1	0	-7.246654	2.965684	-3.047271
49	8	0	-3.767244	1.622321	-0.399940
50	6	0	-3.942577	2.677824	0.541400
51	1	0	-3.188675	2.511814	1.310627
52	1	0	-3.790571	3.654856	0.065378
53	1	0	-4.940525	2.640454	0.994444

54	6	0	-4.975222	-0.875414	-4.430331
55	1	0	-4.029308	-0.763197	-4.975153
56	1	0	-4.913800	-1.831719	-3.900634
57	1	0	-5.782105	-0.928905	-5.167284
58	6	0	0.407511	4.225521	0.188312
59	6	0	-0.320047	4.827189	1.248971
60	6	0	0.913395	5.043349	-0.854958
61	6	0	-0.542561	6.198277	1.253393
62	1	0	-0.700140	4.191031	2.042765
63	6	0	0.685911	6.409060	-0.838171
64	1	0	1.481762	4.603640	-1.666368
65	6	0	-0.043707	6.986546	0.212307
66	1	0	-1.101884	6.655195	2.064030
67	1	0	1.073973	7.034270	-1.636463
68	1	0	-0.218261	8.059177	0.216418
69	6	0	4.030308	-0.085436	1.068920
70	1	0	5.014047	0.147341	1.477512
71	6	0	4.446425	1.449057	-0.887975
72	6	0	4.076933	1.359183	-2.237341
73	6	0	5.789130	1.762114	-0.608727
74	6	0	4.996532	1.596323	-3.263706
75	1	0	3.069402	1.074202	-2.519834
76	6	0	6.708981	2.000455	-1.626662
77	1	0	6.126560	1.812302	0.421373
78	6	0	6.315840	1.924220	-2.964186
79	1	0	4.673875	1.512119	-4.298199
80	1	0	7.739418	2.232124	-1.371537
81	1	0	7.033239	2.104766	-3.760075
82	6	0	3.354563	2.340188	1.270086
83	6	0	3.901680	3.612069	1.028709
84	6	0	2.625060	2.165505	2.465248
85	6	0	3.741670	4.653239	1.943871
86	1	0	4.461319	3.792926	0.118473
87	6	0	2.464611	3.205412	3.381259
88	1	0	2.154846	1.214932	2.690824
89	6	0	3.026244	4.456046	3.126786
90	1	0	4.180527	5.623997	1.727829
91	1	0	1.885722	3.025583	4.281839
92	1	0	2.907175	5.268205	3.838844
93	1	0	3.357058	-0.298516	1.904529
94	7	0	4.178090	-1.286977	0.271868
95	1	0	3.347685	-1.842373	0.111368
96	6	0	5.414011	-1.832368	0.027823
97	8	0	6.453631	-1.285515	0.392757

98	6	0	5.444045	-3.147285	-0.705590
99	6	0	4.332562	-3.724584	-1.336227
100	6	0	6.675263	-3.816306	-0.749055
101	6	0	4.450808	-4.954084	-1.984937
102	1	0	3.370600	-3.223205	-1.344573
103	6	0	6.792196	-5.043452	-1.396655
104	1	0	7.526633	-3.349409	-0.265577
105	6	0	5.678462	-5.617283	-2.015029
106	1	0	3.579966	-5.390564	-2.466074
107	1	0	7.751597	-5.553657	-1.419691
108	1	0	5.767598	-6.575613	-2.520213
109	1	0	-0.848416	-1.758296	1.146137
110	8	0	-0.347435	-0.190606	2.021017
111	16	0	-1.274487	0.594194	2.900805
112	8	0	-1.081502	2.063600	2.781231
113	8	0	-2.668705	0.125859	2.892217
114	6	0	-0.640246	0.223534	4.612196
115	9	0	-0.671618	-1.090747	4.855661
116	9	0	-1.378253	0.852414	5.533490
117	9	0	0.636707	0.644853	4.736336

Supplementary Table 35.

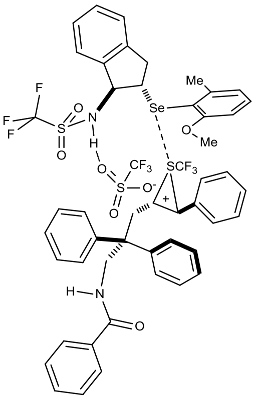

int-III-SSS

B3LYP /6-31G(d)   195.15 K   Thermal correction to Gibbs Free Energy = 0.818498

Thermal correction to Energy = 0.91183

M062x-D3/6-311+G(d,p)   (IEFPCM, Dichloromethane)   Energy = -7062.74405026

Standard orientation:

Center Number	Atomic Number	Atomic Type	Coordinates (Angstroms) X	Y	Z
1	6	0	1.119373	2.644148	0.230729

2	6	0	1.081329	1.206602	0.065260
3	6	0	2.119930	0.495191	-0.797635
4	6	0	3.555604	0.303451	-0.176514
5	16	0	-0.698783	1.343017	-0.791575
6	6	0	-0.253167	1.814340	-2.529365
7	9	0	-1.329231	2.369788	-3.108836
8	9	0	0.743572	2.727618	-2.601909
9	9	0	0.125224	0.762649	-3.260193
10	1	0	1.536135	3.204556	-0.598133
11	1	0	0.809610	0.649312	0.965093
12	1	0	1.706583	-0.476611	-1.078310
13	1	0	2.266868	1.058904	-1.722871
14	34	0	-3.359187	0.352728	-2.069812
15	6	0	-6.739973	-2.617450	1.087908
16	6	0	-5.667922	-2.129959	0.343815
17	6	0	-4.544310	-2.934322	0.127309
18	6	0	-4.464801	-4.225008	0.639537
19	6	0	-5.540585	-4.711060	1.387921
20	6	0	-6.668175	-3.913583	1.609059
21	1	0	-7.615800	-1.999613	1.271540
22	1	0	-3.580342	-4.832793	0.471720
23	1	0	-5.497190	-5.712556	1.807146
24	1	0	-7.494142	-4.301838	2.199217
25	6	0	-3.535763	-2.188456	-0.726476
26	1	0	-3.671096	-2.460233	-1.780245
27	6	0	-3.960484	-0.711589	-0.514013
28	1	0	-3.473039	-0.312213	0.375679
29	6	0	-5.490567	-0.781478	-0.329017
30	7	0	-2.139508	-2.468562	-0.394844
31	16	0	-1.017881	-2.757506	-1.534552
32	8	0	-1.477125	-2.259306	-2.830977
33	8	0	0.306837	-2.435979	-1.001552
34	6	0	-1.036710	-4.619944	-1.693090
35	9	0	-2.254535	-5.015921	-2.089947
36	9	0	-0.131339	-5.014738	-2.588220
37	9	0	-0.767067	-5.181010	-0.510891
38	1	0	-6.004259	-0.759050	-1.300631
39	1	0	-5.871906	0.057596	0.260213
40	6	0	-4.387482	1.977137	-1.939876
41	6	0	-4.505433	2.673538	-0.719359
42	6	0	-4.975717	2.491742	-3.109639
43	6	0	-5.223852	3.872559	-0.660604
44	6	0	-5.682801	3.699725	-3.031675
45	6	0	-5.807785	4.376930	-1.822297

46	1	0	−5.323463	4.409511	0.275446
47	1	0	−6.141436	4.102681	−3.930370
48	1	0	−6.365053	5.308836	−1.777268
49	8	0	−3.888164	2.105010	0.347600
50	6	0	−3.909592	2.768669	1.606976
51	1	0	−3.324440	2.132759	2.269777
52	1	0	−3.447482	3.761314	1.536446
53	1	0	−4.935630	2.870215	1.983799
54	6	0	−4.847547	1.778983	−4.435956
55	1	0	−3.806720	1.761449	−4.782540
56	1	0	−5.166535	0.733877	−4.358095
57	1	0	−5.449693	2.274068	−5.203248
58	6	0	0.570572	3.436169	1.296629
59	6	0	0.452531	4.830599	1.081777
60	6	0	0.120890	2.885028	2.518207
61	6	0	−0.130432	5.644684	2.041685
62	1	0	0.807823	5.256261	0.147024
63	6	0	−0.457652	3.709983	3.475140
64	1	0	0.227022	1.823875	2.719324
65	6	0	−0.593592	5.082327	3.238269
66	1	0	−0.227671	6.711849	1.865460
67	1	0	−0.809898	3.273284	4.404786
68	1	0	−1.053088	5.718356	3.990280
69	6	0	4.327460	−0.442763	−1.321769
70	1	0	3.750532	−1.347165	−1.550915
71	6	0	4.103929	1.707000	0.130060
72	6	0	4.889943	2.433850	−0.775941
73	6	0	3.708892	2.350629	1.318549
74	6	0	5.248809	3.758134	−0.508131
75	1	0	5.258678	1.973755	−1.686915
76	6	0	4.066666	3.674280	1.585003
77	1	0	3.146639	1.795638	2.064813
78	6	0	4.832493	4.388787	0.666070
79	1	0	5.862207	4.295942	−1.229358
80	1	0	3.744848	4.139483	2.512932
81	1	0	5.115848	5.418582	0.867050
82	6	0	3.601341	−0.613508	1.062203
83	6	0	2.643584	−1.608720	1.294115
84	6	0	4.697840	−0.540018	1.943665
85	6	0	2.756150	−2.476100	2.383499
86	1	0	1.785474	−1.724114	0.643143
87	6	0	4.817424	−1.413903	3.023809
88	1	0	5.459469	0.219072	1.792580
89	6	0	3.840530	−2.384762	3.252405

90	1	0	1.972258	-3.208006	2.552629
91	1	0	5.671772	-1.327119	3.690506
92	1	0	3.921910	-3.056272	4.102939
93	1	0	4.369472	0.158153	-2.231423
94	7	0	5.690401	-0.824079	-1.009310
95	1	0	5.830763	-1.418054	-0.204086
96	6	0	6.779608	-0.400706	-1.718480
97	8	0	6.705403	0.434770	-2.620825
98	6	0	8.096398	-1.014140	-1.327051
99	6	0	8.210095	-2.271160	-0.715762
100	6	0	9.260086	-0.295307	-1.631677
101	6	0	9.465593	-2.789836	-0.397837
102	1	0	7.325598	-2.870455	-0.516700
103	6	0	10.512674	-0.809242	-1.304944
104	1	0	9.157248	0.663613	-2.128784
105	6	0	10.618328	-2.057214	-0.685141
106	1	0	9.542196	-3.769326	0.066194
107	1	0	11.408269	-0.239381	-1.537427
108	1	0	11.595843	-2.460895	-0.435057
109	1	0	-1.791600	-2.341819	0.573277
110	8	0	-1.231240	-2.128220	2.294971
111	16	0	-1.077895	-0.719316	2.744122
112	8	0	0.311634	-0.288650	3.026736
113	8	0	-1.858011	0.262805	1.943247
114	6	0	-1.893664	-0.676566	4.415711
115	9	0	-3.178038	-1.044391	4.329085
116	9	0	-1.848826	0.578966	4.907815
117	9	0	-1.269605	-1.488339	5.274893

Supplementary Table 36.

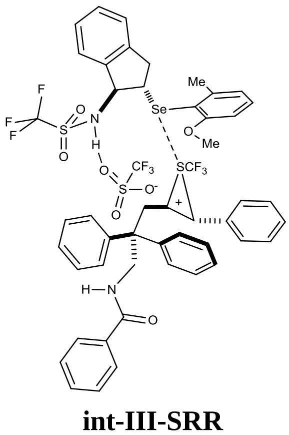

int-III-SRR

B3LYP /6-31G(d)   195.15 K   Thermal correction to Gibbs Free Energy = 0.819569

Thermal correction to Energy = 0.912509

M062x-D3/6-311+G(d,p) (IEFPCM, Dichloromethane) Energy = -7062.75315922

Standard orientation:

Center Number	Atomic Number	Atomic Type	Coordinates (Angstroms) X	Y	Z
1	6	0	1.124771	1.802614	0.827085
2	6	0	1.344631	1.255624	-0.504320
3	6	0	1.757163	-0.216252	-0.592555
4	6	0	3.259090	-0.479703	-0.235353
5	16	0	-0.533806	1.344688	-0.932424
6	6	0	-0.620241	3.066138	-1.598029
7	9	0	-0.955619	3.961354	-0.655059
8	9	0	0.551096	3.473947	-2.133720
9	9	0	-1.542807	3.117151	-2.563675
10	1	0	0.635448	1.137169	1.536580
11	1	0	1.882052	1.897562	-1.197826
12	1	0	1.114136	-0.802497	0.068023
13	1	0	1.551379	-0.550822	-1.610872
14	34	0	-3.581193	0.810129	-1.950630
15	6	0	-6.897632	-2.070686	1.361288
16	6	0	-5.870592	-1.637765	0.526709
17	6	0	-4.868362	-2.528456	0.127391
18	6	0	-4.870044	-3.854467	0.545229
19	6	0	-5.900747	-4.286975	1.385034
20	6	0	-6.905817	-3.402367	1.788692
21	1	0	-7.675373	-1.384091	1.687112
22	1	0	-4.082604	-4.533675	0.232167
23	1	0	-5.917197	-5.316752	1.731917
24	1	0	-7.696710	-3.750850	2.447825
25	6	0	-3.903615	-1.819646	-0.806718
26	1	0	-4.203749	-1.994937	-1.846329
27	6	0	-4.133524	-0.333371	-0.436313
28	1	0	-3.534997	-0.075113	0.436792
29	6	0	-5.631500	-0.267118	-0.077460
30	7	0	-2.499955	-2.235843	-0.704502
31	16	0	-1.641428	-2.707444	-2.014156
32	8	0	-2.525768	-2.843230	-3.168493
33	8	0	-0.371839	-1.981028	-2.105128
34	6	0	-1.120061	-4.438555	-1.540303
35	9	0	-2.185380	-5.239855	-1.440803
36	9	0	-0.296210	-4.923394	-2.477687
37	9	0	-0.477451	-4.410745	-0.370102
38	1	0	-6.242645	-0.122684	-0.979517

39	1	0	−5.855140	0.557310	0.605818
40	6	0	−4.451474	2.475660	−1.532493
41	6	0	−4.231638	3.096450	−0.285037
42	6	0	−5.262407	3.092194	−2.500831
43	6	0	−4.841276	4.321628	0.005040
44	6	0	−5.855027	4.325646	−2.194428
45	6	0	−5.650268	4.926823	−0.956505
46	1	0	−4.684452	4.803444	0.962778
47	1	0	−6.486887	4.807886	−2.935155
48	1	0	−6.122864	5.879186	−0.731069
49	8	0	−3.414556	2.432815	0.575962
50	6	0	−3.174537	2.984103	1.868284
51	1	0	−2.544818	2.260393	2.384489
52	1	0	−2.658414	3.949661	1.790580
53	1	0	−4.112206	3.112329	2.422404
54	6	0	−5.495112	2.461194	−3.853610
55	1	0	−4.580218	2.462410	−4.459528
56	1	0	−5.797719	1.413616	−3.754863
57	1	0	−6.266059	3.003597	−4.408925
58	6	0	1.400160	3.118362	1.302567
59	6	0	0.960398	3.430431	2.615562
60	6	0	2.122036	4.091297	0.564803
61	6	0	1.224380	4.680120	3.162019
62	1	0	0.411996	2.675920	3.171809
63	6	0	2.379733	5.332866	1.121295
64	1	0	2.480680	3.867928	−0.433255
65	6	0	1.929004	5.629509	2.416521
66	1	0	0.884478	4.916629	4.165674
67	1	0	2.935188	6.075311	0.556622
68	1	0	2.135767	6.606860	2.844399
69	6	0	4.102062	0.114773	−1.409757
70	1	0	3.677561	−0.286319	−2.339523
71	6	0	3.520069	−2.005494	−0.191903
72	6	0	4.631846	−2.504940	0.510837
73	6	0	2.717028	−2.924132	−0.884834
74	6	0	4.932762	−3.867644	0.513552
75	1	0	5.256146	−1.822146	1.079535
76	6	0	3.011519	−4.289740	−0.871844
77	1	0	1.839188	−2.598325	−1.429493
78	6	0	4.121684	−4.768538	−0.178420
79	1	0	5.795926	−4.223834	1.069995
80	1	0	2.354959	−4.973793	−1.402350
81	1	0	4.347888	−5.831329	−0.167816
82	6	0	3.556014	0.151916	1.136534

83	6	0	4.442670	1.223901	1.324999
84	6	0	2.874836	-0.350847	2.263896
85	6	0	4.639435	1.767786	2.597665
86	1	0	4.985217	1.653918	0.489776
87	6	0	3.062817	0.200752	3.529073
88	1	0	2.183364	-1.179984	2.152855
89	6	0	3.952655	1.263233	3.702257
90	1	0	5.335044	2.594187	2.718757
91	1	0	2.501746	-0.199609	4.367746
92	1	0	4.108839	1.692488	4.688425
93	1	0	4.020076	1.202608	-1.452444
94	7	0	5.523541	-0.174849	-1.378043
95	1	0	5.799091	-1.142939	-1.292642
96	6	0	6.484040	0.793186	-1.457620
97	8	0	6.209664	1.995889	-1.443958
98	6	0	7.907554	0.317174	-1.532843
99	6	0	8.276695	-0.959350	-1.980715
100	6	0	8.907577	1.224311	-1.156750
101	6	0	9.621295	-1.327040	-2.031373
102	1	0	7.525919	-1.665153	-2.326154
103	6	0	10.249243	0.853942	-1.200215
104	1	0	8.607461	2.216327	-0.836228
105	6	0	10.609296	-0.424062	-1.635211
106	1	0	9.896268	-2.315712	-2.388246
107	1	0	11.015831	1.562429	-0.898352
108	1	0	11.656323	-0.712457	-1.673249
109	1	0	-1.930056	-1.866348	0.076321
110	8	0	-0.832404	-1.102892	1.367115
111	16	0	-1.360107	-0.572506	2.667002
112	8	0	-0.630010	0.633447	3.137992
113	8	0	-2.827455	-0.510165	2.751953
114	6	0	-0.859091	-1.882276	3.892671
115	9	0	-1.383302	-3.066254	3.559005
116	9	0	-1.274991	-1.556202	5.121719
117	9	0	0.484335	-2.009013	3.922873

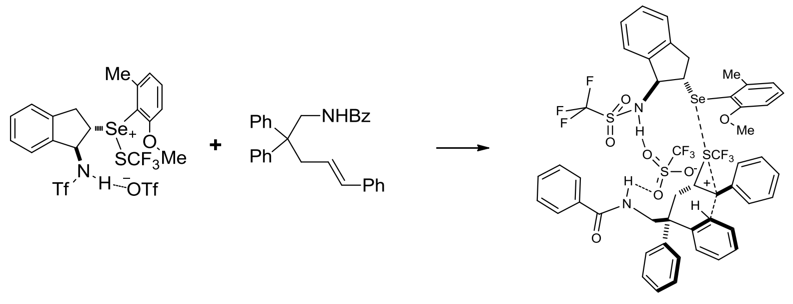

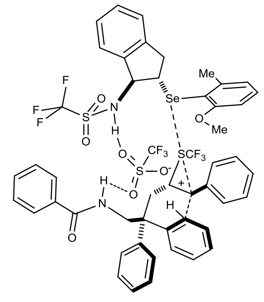	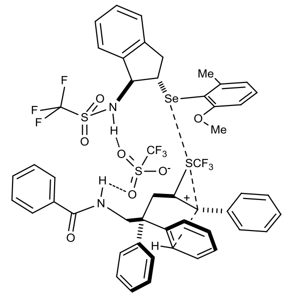
**TS-II-RSS**	**TS-II-RRR**
$\Delta G_{195.15\ K}$ = -1.8 kcal/mol	$\Delta G_{195.15\ K}$ = -0.4kcal/mol
**$\Delta\Delta G_{195.15\ K}$ = 0.0 kcal/mol**	**$\Delta\Delta G_{195.15\ K}$ = 1.4 kcal/mol**

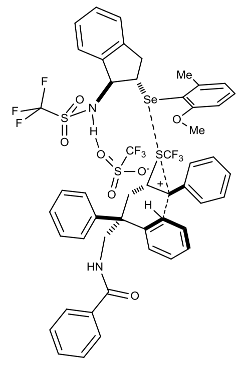	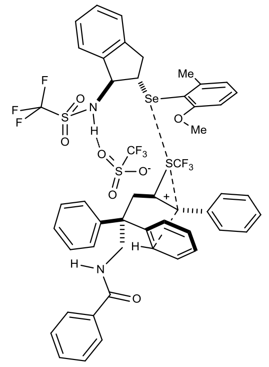
**int-III-SSS**	**int-III-SRR**
$\Delta G_{195.15\ K}$ = -0.5 kcal/mol	$\Delta G_{195.15\ K}$ = -0.3 kcal/mol
**$\Delta\Delta G_{195.15\ K}$ = 1.3 kcal/mol**	**$\Delta\Delta G_{195.15\ K}$ = 1.5 kcal/mol**

Supplementary Figure 182. DFT calculations for **TS-II** of **1a**; ΔG related to **int-I**.

Supplementary Table 37.

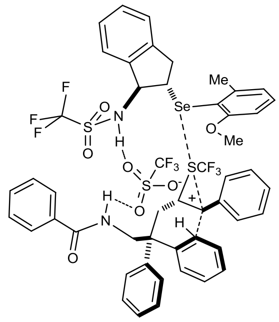

TS-II-RSS

B3LYP /6-31G(d) 195.15 K Thermal correction to Gibbs Free Energy = 0.820148

Thermal correction to Energy = 0.911742

M062x-D3/6-311+G(d,p) (IEFPCM, Dichloromethane) Energy = -7062.74421107

Standard orientation:

Center Number	Atomic Number	Atomic Type	Coordinates (Angstroms) X	Y	Z
1	6	0	-0.826498	-3.445532	0.221838
2	6	0	-0.831338	-1.968583	-0.095068
3	6	0	-1.991044	-1.522200	-0.988571
4	6	0	-3.421755	-1.747675	-0.388263
5	16	0	0.843053	-1.479929	-0.808584
6	6	0	0.708636	-2.091153	-2.527699
7	9	0	0.154049	-3.331288	-2.601024
8	9	0	-0.032909	-1.298639	-3.323741
9	9	0	1.934506	-2.173055	-3.068881
10	1	0	-1.015716	-4.064862	-0.652692
11	1	0	-0.789235	-1.380062	0.826677
12	1	0	-1.868810	-0.460993	-1.214956
13	1	0	-1.936010	-2.072026	-1.930593
14	34	0	3.232502	0.416395	-1.952456
15	6	0	5.300717	4.304282	1.324140
16	6	0	4.468263	3.485938	0.563698
17	6	0	3.124216	3.828526	0.382593
18	6	0	2.586137	4.977871	0.952604
19	6	0	3.422410	5.796034	1.717469
20	6	0	4.768641	5.462635	1.899393
21	1	0	6.345084	4.043662	1.479066
22	1	0	1.536404	5.221767	0.819476
23	1	0	3.021309	6.693161	2.181113
24	1	0	5.405113	6.104982	2.502446
25	6	0	2.441944	2.797892	-0.501785

26	1	0	2.434997	3.148804	-1.540998
27	6	0	3.383850	1.575801	-0.361445
28	1	0	3.087692	0.997488	0.512500
29	6	0	4.783958	2.191460	-0.158438
30	7	0	1.047586	2.528864	-0.127031
31	16	0	-0.128675	2.447180	-1.251741
32	8	0	0.395233	2.169809	-2.588485
33	8	0	-1.259078	1.694476	-0.693175
34	6	0	-0.725388	4.216271	-1.336982
35	9	0	0.279286	5.008399	-1.732902
36	9	0	-1.726790	4.301846	-2.222563
37	9	0	-1.159374	4.623884	-0.145522
38	1	0	5.262351	2.387015	-1.128077
39	1	0	5.447863	1.525788	0.401872
40	6	0	4.674299	-0.836016	-1.704122
41	6	0	4.904926	-1.462242	-0.461340
42	6	0	5.462219	-1.175315	-2.818828
43	6	0	5.932778	-2.400435	-0.324017
44	6	0	6.479479	-2.127735	-2.664825
45	6	0	6.715310	-2.726683	-1.431695
46	1	0	6.119188	-2.877913	0.630865
47	1	0	7.091273	-2.391363	-3.523305
48	1	0	7.513192	-3.456820	-1.325001
49	8	0	4.077050	-1.096086	0.555754
50	6	0	4.213677	-1.720719	1.825362
51	1	0	3.430772	-1.290296	2.449941
52	1	0	4.066209	-2.804686	1.750926
53	1	0	5.197141	-1.514283	2.267869
54	6	0	5.217201	-0.547425	-4.170929
55	1	0	4.242267	-0.843992	-4.578118
56	1	0	5.205079	0.545997	-4.102959
57	1	0	5.987389	-0.849462	-4.886859
58	6	0	0.185322	-4.049638	1.119216
59	6	0	0.746516	-5.286861	0.752716
60	6	0	0.583362	-3.445908	2.324916
61	6	0	1.695359	-5.902773	1.563932
62	1	0	0.445202	-5.756269	-0.180509
63	6	0	1.523099	-4.073842	3.140110
64	1	0	0.152245	-2.497499	2.632297
65	6	0	2.080601	-5.299410	2.764696
66	1	0	2.131350	-6.851195	1.263088
67	1	0	1.817734	-3.596268	4.069487
68	1	0	2.811571	-5.784764	3.405958
69	6	0	-3.642694	-0.823259	0.875636

70	1	0	−4. 514314	−1. 172746	1. 432089
71	6	0	−3. 436975	−3. 205091	0. 042513
72	6	0	−4. 133049	−4. 200488	−0. 650860
73	6	0	−2. 563632	−3. 619251	1. 119257
74	6	0	−4. 079079	−5. 532854	−0. 243425
75	1	0	−4. 741805	−3. 929321	−1. 504325
76	6	0	−2. 607040	−4. 985554	1. 566074
77	1	0	−2. 314372	−2. 882746	1. 880884
78	6	0	−3. 329967	−5. 932702	0. 880897
79	1	0	−4. 637186	−6. 277650	−0. 805144
80	1	0	−2. 011686	−5. 260713	2. 430659
81	1	0	−3. 332490	−6. 970413	1. 199293
82	6	0	−4. 511822	−1. 442988	−1. 434086
83	6	0	−5. 864558	−1. 568281	−1. 071146
84	6	0	−4. 221894	−1. 030613	−2. 742023
85	6	0	−6. 884775	−1. 303461	−1. 982358
86	1	0	−6. 130575	−1. 846796	−0. 057026
87	6	0	−5. 244623	−0. 761312	−3. 656089
88	1	0	−3. 197548	−0. 897544	−3. 070013
89	6	0	−6. 579436	−0. 900337	−3. 283486
90	1	0	−7. 919983	−1. 399199	−1. 666707
91	1	0	−4. 987615	−0. 436570	−4. 660800
92	1	0	−7. 373836	−0. 687666	−3. 993722
93	1	0	−2. 778006	−0. 870133	1. 542052
94	7	0	−3. 898590	0. 561797	0. 549562
95	1	0	−3. 101319	1. 157652	0. 361399
96	6	0	−5. 109461	1. 137019	0. 851667
97	8	0	−6. 043547	0. 485877	1. 320263
98	6	0	−5. 246248	2. 608098	0. 582725
99	6	0	−4. 417173	3. 318400	−0. 296039
100	6	0	−6. 285140	3. 281890	1. 239985
101	6	0	−4. 617177	4. 684120	−0. 499887
102	1	0	−3. 629265	2. 813134	−0. 844212
103	6	0	−6. 479009	4. 646035	1. 040460
104	1	0	−6. 927808	2. 712766	1. 903197
105	6	0	−5. 643512	5. 351070	0. 170181
106	1	0	−3. 968441	5. 222327	−1. 184083
107	1	0	−7. 281647	5. 160574	1. 562225
108	1	0	−5. 794654	6. 415860	0. 012085
109	1	0	0. 870859	1. 925459	0. 698926
110	8	0	0. 870366	0. 522292	1. 853835
111	16	0	−0. 117328	0. 501929	2. 980175
112	8	0	−0. 784476	1. 777916	3. 254086
113	8	0	−0. 990646	−0. 707407	2. 953398

114	6	0	0.970585	0.187131	4.459783
115	9	0	1.661067	-0.970761	4.297355
116	9	0	0.244451	0.076876	5.576223
117	9	0	1.860004	1.172631	4.617250

Supplementary Table 38.

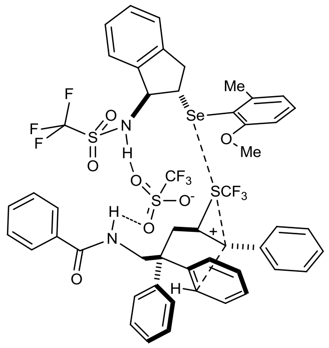

TS-II-RRR

B3LYP /6-31G(d) 195.15 K Thermal correction to Gibbs Free Energy = 0.817854

Thermal correction to Energy = 0.911241

M062x-D3/6-311+G(d,p) (IEFPCM, Dichloromethane) Energy = -7062.74211881

Standard orientation:

Center Number	Atomic Number	Atomic Type	Coordinates (Angstroms) X	Y	Z
1	6	0	1.765244	3.181935	0.490271
2	6	0	1.626986	1.971432	-0.396555
3	6	0	1.950458	0.628854	0.264039
4	6	0	3.417720	0.529482	0.801468
5	16	0	-0.223958	1.868413	-0.798820
6	6	0	-0.327790	2.959733	-2.277277
7	9	0	-0.565155	4.247658	-1.969170
8	9	0	0.801399	2.930272	-3.015908
9	9	0	-1.337196	2.551462	-3.059721
10	1	0	1.183492	3.096373	1.406885
11	1	0	2.161301	2.090769	-1.337467
12	1	0	1.280013	0.459629	1.114084
13	1	0	1.759897	-0.182362	-0.441090
14	34	0	-3.346522	1.009302	-1.331771
15	6	0	-6.536603	-2.727008	1.104253
16	6	0	-5.508852	-2.054825	0.446205
17	6	0	-4.462475	-2.775397	-0.139257
18	6	0	-4.420133	-4.164695	-0.082679

19	6	0	−5.450742	−4.837122	0.580039
20	6	0	−6.500236	−4.123476	1.168242
21	1	0	−7.350593	−2.176805	1.570517
22	1	0	−3.597953	−4.709862	−0.536853
23	1	0	−5.432878	−5.921754	0.644659
24	1	0	−7.291430	−4.659194	1.686397
25	6	0	−3.490065	−1.823999	−0.814349
26	1	0	−3.713266	−1.765419	−1.886392
27	6	0	−3.814830	−0.477151	−0.120313
28	1	0	−3.215268	−0.390819	0.784165
29	6	0	−5.315825	−0.567218	0.225496
30	7	0	−2.082291	−2.216634	−0.693982
31	16	0	−1.055049	−2.113318	−1.959442
32	8	0	−1.687088	−1.428356	−3.087149
33	8	0	0.286882	−1.765546	−1.487751
34	6	0	−0.920935	−3.898026	−2.494820
35	9	0	−2.134953	−4.366254	−2.815230
36	9	0	−0.119388	−3.993480	−3.560711
37	9	0	−0.417541	−4.639735	−1.504627
38	1	0	−5.929543	−0.213699	−0.614687
39	1	0	−5.577282	0.043344	1.096035
40	6	0	−3.922435	2.544258	−0.319274
41	6	0	−3.535461	2.713530	1.028117
42	6	0	−4.680778	3.536796	−0.964858
43	6	0	−3.951235	3.842728	1.742054
44	6	0	−5.070979	4.670700	−0.238000
45	6	0	−4.721123	4.813785	1.100962
46	1	0	−3.679824	3.966386	2.784080
47	1	0	−5.661310	5.438293	−0.731101
48	1	0	−5.045021	5.689980	1.656692
49	8	0	−2.750376	1.734405	1.552464
50	6	0	−2.273949	1.856245	2.888014
51	1	0	−1.613595	1.003175	3.044934
52	1	0	−1.711891	2.790839	3.021547
53	1	0	−3.101120	1.830326	3.608997
54	6	0	−5.055201	3.408304	−2.423083
55	1	0	−4.165651	3.438919	−3.064761
56	1	0	−5.548096	2.450764	−2.623418
57	1	0	−5.721253	4.220555	−2.728977
58	6	0	1.757025	4.543903	−0.087318
59	6	0	0.997769	5.546511	0.541105
60	6	0	2.487849	4.869000	−1.244189
61	6	0	0.945946	6.832862	0.011970
62	1	0	0.427835	5.303732	1.434002

63	6	0	2.445336	6.159182	−1.764106
64	1	0	3.093008	4.114484	−1.738596
65	6	0	1.671713	7.142382	−1.140934
66	1	0	0.338404	7.592133	0.495572
67	1	0	3.012798	6.398079	−2.658694
68	1	0	1.636030	8.147004	−1.552625
69	6	0	3.587495	−0.868305	1.496527
70	1	0	4.581407	−0.916616	1.950378
71	6	0	4.484342	0.678135	−0.294714
72	6	0	4.223981	0.375303	−1.637875
73	6	0	5.795253	1.039320	0.056094
74	6	0	5.234234	0.454988	−2.600204
75	1	0	3.237223	0.047090	−1.946878
76	6	0	6.803679	1.122721	−0.900992
77	1	0	6.035463	1.243038	1.097260
78	6	0	6.525129	0.834892	−2.238365
79	1	0	5.006070	0.208195	−3.633524
80	1	0	7.810313	1.398830	−0.598812
81	1	0	7.310399	0.893510	−2.986966
82	6	0	3.479264	1.659226	1.815212
83	6	0	3.586217	3.005724	1.315116
84	6	0	3.229040	1.487420	3.181025
85	6	0	3.642703	4.102535	2.238143
86	1	0	4.101095	3.135856	0.368251
87	6	0	3.211731	2.583408	4.043438
88	1	0	3.031391	0.504782	3.587364
89	6	0	3.433622	3.896537	3.582022
90	1	0	3.808660	5.101971	1.848586
91	1	0	3.024073	2.413756	5.100353
92	1	0	3.444884	4.726343	4.281880
93	1	0	2.819255	−0.979326	2.262365
94	7	0	3.445583	−1.986304	0.591247
95	1	0	2.541044	−2.447252	0.638575
96	6	0	4.546877	−2.708941	0.195912
97	8	0	5.693474	−2.363807	0.476931
98	6	0	4.274964	−3.946942	−0.611173
99	6	0	5.316587	−4.877545	−0.728416
100	6	0	3.056471	−4.194017	−1.259224
101	6	0	5.138826	−6.046300	−1.463973
102	1	0	6.257466	−4.659301	−0.234071
103	6	0	2.883508	−5.362209	−2.002781
104	1	0	2.247013	−3.472268	−1.212536
105	6	0	3.920173	−6.291242	−2.102863
106	1	0	5.949936	−6.765672	−1.542336

107	1	0	1.938020	-5.538227	-2.506252
108	1	0	3.781074	-7.201420	-2.680852
109	1	0	-1.645027	-2.106582	0.238397
110	8	0	0.871168	-2.601215	1.862286
111	16	0	-0.075053	-1.752051	2.618695
112	8	0	0.492254	-0.477484	3.135561
113	8	0	-1.413235	-1.601227	1.980449
114	6	0	-0.436298	-2.729605	4.160578
115	9	0	-0.978969	-3.913286	3.853620
116	9	0	-1.290452	-2.058393	4.948660
117	9	0	0.695288	-2.943466	4.848951

Supplementary Table 39.

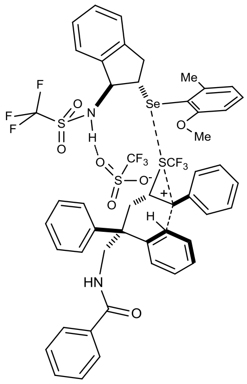

TS-II-SSS

B3LYP /6-31G(d) 195.15 K Thermal correction to Gibbs Free Energy = 0.819096

Thermal correction to Energy = 0.911464

M062x-D3/6-311+G(d,p) (IEFPCM, Dichloromethane) Energy = -7062.74356726

Standard orientation:

Center Number	Atomic Number	Atomic Type	Coordinates (Angstroms) X	Y	Z
1	6	0	1.560098	2.655099	0.382587
2	6	0	1.113965	1.250453	0.034419
3	6	0	2.084483	0.452036	-0.834193
4	6	0	3.523633	0.260507	-0.229070
5	16	0	-0.612339	1.367223	-0.732937
6	6	0	-0.215555	1.873227	-2.448994
7	9	0	-1.302210	2.424184	-3.013492
8	9	0	0.776682	2.804209	-2.513173
9	9	0	0.175370	0.851756	-3.230000
10	1	0	1.822916	3.238848	-0.496829
11	1	0	0.871835	0.693005	0.944623

12	1	0	1.643269	-0.522729	-1.052611
13	1	0	2.220971	0.958524	-1.793719
14	34	0	-3.397281	0.355066	-2.089916
15	6	0	-6.732917	-2.646929	1.075708
16	6	0	-5.664122	-2.147116	0.335105
17	6	0	-4.538248	-2.944926	0.105632
18	6	0	-4.454310	-4.241031	0.603685
19	6	0	-5.526662	-4.739306	1.349261
20	6	0	-6.656604	-3.948557	1.581871
21	1	0	-7.609938	-2.033358	1.268210
22	1	0	-3.568393	-4.844428	0.427465
23	1	0	-5.478663	-5.745425	1.757109
24	1	0	-7.480090	-4.346358	2.169372
25	6	0	-3.535257	-2.186219	-0.744813
26	1	0	-3.668304	-2.454297	-1.799849
27	6	0	-3.967274	-0.711716	-0.527306
28	1	0	-3.471375	-0.311643	0.357227
29	6	0	-5.494309	-0.791422	-0.321944
30	7	0	-2.136098	-2.464559	-0.413152
31	16	0	-1.012762	-2.748227	-1.550171
32	8	0	-1.458909	-2.248522	-2.849412
33	8	0	0.314840	-2.443212	-1.009987
34	6	0	-1.030317	-4.609217	-1.722124
35	9	0	-2.245457	-5.007262	-2.124682
36	9	0	-0.121808	-4.996097	-2.619887
37	9	0	-0.759519	-5.182644	-0.545240
38	1	0	-6.018573	-0.757269	-1.287280
39	1	0	-5.870149	0.039663	0.282128
40	6	0	-4.418309	1.979270	-1.935775
41	6	0	-4.527471	2.668373	-0.709561
42	6	0	-4.999849	2.515437	-3.099011
43	6	0	-5.234643	3.873357	-0.638743
44	6	0	-5.694836	3.729755	-3.010356
45	6	0	-5.815123	4.395662	-1.794496
46	1	0	-5.327047	4.402416	0.302616
47	1	0	-6.147683	4.146531	-3.905991
48	1	0	-6.363842	5.332291	-1.739596
49	8	0	-3.912315	2.087847	0.352739
50	6	0	-3.901137	2.756578	1.608421
51	1	0	-3.310530	2.117036	2.262887
52	1	0	-3.427266	3.742563	1.525112
53	1	0	-4.918506	2.873015	2.005310
54	6	0	-4.871627	1.816698	-4.432425
55	1	0	-3.830783	1.805659	-4.780006

56	1	0	−5.184758	0.769265	−4.361600
57	1	0	−5.476178	2.317031	−5.194921
58	6	0	0.775795	3.447796	1.368220
59	6	0	0.534502	4.807947	1.104779
60	6	0	0.278274	2.882129	2.554307
61	6	0	−0.199945	5.583811	1.997578
62	1	0	0.912733	5.251547	0.186675
63	6	0	−0.454880	3.663177	3.446463
64	1	0	0.442260	1.833234	2.786393
65	6	0	−0.694450	5.012004	3.174282
66	1	0	−0.389539	6.630345	1.775613
67	1	0	−0.846408	3.202540	4.348762
68	1	0	−1.265035	5.617003	3.873989
69	6	0	4.324236	−0.456761	−1.363993
70	1	0	3.757688	−1.360871	−1.615891
71	6	0	3.988485	1.672563	0.074644
72	6	0	4.919513	2.372159	−0.690416
73	6	0	3.286633	2.378544	1.131997
74	6	0	5.251737	3.692399	−0.366690
75	1	0	5.433957	1.895545	−1.520307
76	6	0	3.732313	3.702064	1.487210
77	1	0	2.927047	1.764788	1.956457
78	6	0	4.672158	4.360097	0.728482
79	1	0	5.992607	4.206351	−0.973902
80	1	0	3.263779	4.190876	2.335097
81	1	0	4.976208	5.372743	0.974606
82	6	0	3.581082	−0.627693	1.029205
83	6	0	2.634403	−1.627662	1.280064
84	6	0	4.672930	−0.508840	1.910641
85	6	0	2.763631	−2.470275	2.386427
86	1	0	1.775496	−1.763077	0.634331
87	6	0	4.807057	−1.357781	3.008949
88	1	0	5.426318	0.256260	1.737703
89	6	0	3.847907	−2.343299	3.251474
90	1	0	1.990690	−3.209075	2.573122
91	1	0	5.657352	−1.242604	3.676288
92	1	0	3.941022	−2.996999	4.114338
93	1	0	4.375803	0.159843	−2.263030
94	7	0	5.686104	−0.833226	−1.030932
95	1	0	5.811806	−1.485217	−0.269770
96	6	0	6.784741	−0.376752	−1.702845
97	8	0	6.715341	0.513269	−2.555184
98	6	0	8.099990	−0.998287	−1.328251
99	6	0	8.217550	−2.261295	−0.729845

100	6	0	9.261491	-0.272400	-1.625961
101	6	0	9.474536	-2.777666	-0.415830
102	1	0	7.337089	-2.868521	-0.537011
103	6	0	10.515746	-0.785606	-1.305504
104	1	0	9.155580	0.691096	-2.113216
105	6	0	10.624855	-2.038362	-0.696398
106	1	0	9.554529	-3.760960	0.039116
107	1	0	11.409524	-0.211608	-1.534184
108	1	0	11.603419	-2.441399	-0.449925
109	1	0	-1.786624	-2.328536	0.553288
110	8	0	-1.208140	-2.141900	2.279328
111	16	0	-1.058812	-0.737169	2.747505
112	8	0	0.333570	-0.320194	3.044777
113	8	0	-1.838301	0.253735	1.964531
114	6	0	-1.882918	-0.717831	4.415817
115	9	0	-3.166054	-1.088508	4.319116
116	9	0	-1.845279	0.531128	4.924905
117	9	0	-1.262691	-1.538315	5.272092

Supplementary Table 40.

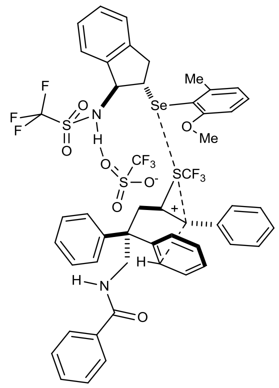

TS-II-SRR

B3LYP /6-31G(d) 195.15 K Thermal correction to Gibbs Free Energy = 0.820981

Thermal correction to Energy = 0.911800

M062x-D3/6-311+G(d,p) (IEFPCM, Dichloromethane) Energy = -7062.74510560

Standard orientation:

Center Number	Atomic Number	Atomic Type	Coordinates (Angstroms) X	Y	Z
1	6	0	1.749787	1.612309	0.735885
2	6	0	1.501693	1.148703	-0.681772
3	6	0	1.747577	-0.344542	-0.950462
4	6	0	3.195206	-0.824496	-0.595972

5	16	0	−0.354042	1.367227	−0.972657
6	6	0	−0.420421	3.076341	−1.635469
7	9	0	−0.664269	4.000153	−0.689064
8	9	0	0.723969	3.441558	−2.258998
9	9	0	−1.410814	3.156974	−2.536802
10	1	0	1.191388	1.032462	1.472100
11	1	0	2.008126	1.777961	−1.412578
12	1	0	1.032513	−0.936718	−0.373166
13	1	0	1.542908	−0.533522	−2.007015
14	34	0	−3.633054	1.053109	−1.715698
15	6	0	−6.795436	−1.768083	1.795711
16	6	0	−5.829656	−1.359252	0.879524
17	6	0	−4.936580	−2.291441	0.339372
18	6	0	−4.989286	−3.634231	0.696495
19	6	0	−5.958676	−4.042506	1.617753
20	6	0	−6.854293	−3.116741	2.161909
21	1	0	−7.486970	−1.050275	2.230697
22	1	0	−4.286585	−4.346351	0.274308
23	1	0	−6.012130	−5.085787	1.917724
24	1	0	−7.598222	−3.446633	2.882607
25	6	0	−4.021359	−1.598259	−0.655708
26	1	0	−4.432941	−1.699108	−1.666759
27	6	0	−4.109321	−0.120199	−0.199491
28	1	0	−3.408881	0.053635	0.617798
29	6	0	−5.555162	0.021783	0.316463
30	7	0	−2.646048	−2.110602	−0.716232
31	16	0	−1.961440	−2.605239	−2.114097
32	8	0	−2.953957	−2.613440	−3.185126
33	8	0	−0.638308	−2.003552	−2.298086
34	6	0	−1.566593	−4.397104	−1.756681
35	9	0	−2.687954	−5.107085	−1.594523
36	9	0	−0.873437	−4.910840	−2.782752
37	9	0	−0.825476	−4.486555	−0.648301
38	1	0	−6.240925	0.252767	−0.510709
39	1	0	−5.650629	0.822827	1.055356
40	6	0	−4.307124	2.767183	−1.154077
41	6	0	−3.938904	3.302091	0.097744
42	6	0	−5.112883	3.512936	−2.031422
43	6	0	−4.386290	4.570742	0.480864
44	6	0	−5.544894	4.786762	−1.633206
45	6	0	−5.188626	5.305067	−0.392467
46	1	0	−4.108066	4.987145	1.442014
47	1	0	−6.170381	5.367545	−2.305865
48	1	0	−5.535640	6.291433	−0.095647

49	8	0	−3.151009	2.509826	0.870707
50	6	0	−2.733610	2.976656	2.147780
51	1	0	−2.174000	2.151146	2.586104
52	1	0	−2.096898	3.865052	2.048847
53	1	0	−3.594959	3.210528	2.786397
54	6	0	−5.506521	2.976298	−3.387612
55	1	0	−4.641996	2.912708	−4.060583
56	1	0	−5.909482	1.961149	−3.308230
57	1	0	−6.253258	3.621660	−3.860197
58	6	0	1.785719	3.061139	1.042515
59	6	0	1.098755	3.511003	2.185228
60	6	0	2.482994	3.990135	0.250858
61	6	0	1.082761	4.866346	2.505924
62	1	0	0.573672	2.786700	2.801938
63	6	0	2.472874	5.342224	0.582187
64	1	0	3.050573	3.658696	−0.613892
65	6	0	1.768395	5.783982	1.705439
66	1	0	0.538622	5.206998	3.382449
67	1	0	3.015828	6.051992	−0.035096
68	1	0	1.759642	6.840489	1.959176
69	6	0	4.161515	−0.090091	−1.585639
70	1	0	3.935395	−0.479592	−2.584979
71	6	0	3.362227	−2.341709	−0.803934
72	6	0	4.521654	−2.987419	−0.332372
73	6	0	2.418265	−3.111883	−1.496280
74	6	0	4.735430	−4.346770	−0.559736
75	1	0	5.250315	−2.429799	0.250130
76	6	0	2.628721	−4.476174	−1.716205
77	1	0	1.497503	−2.671979	−1.858930
78	6	0	3.788092	−5.098360	−1.257801
79	1	0	5.637218	−4.819498	−0.179150
80	1	0	1.866231	−5.045656	−2.239897
81	1	0	3.948284	−6.159188	−1.429674
82	6	0	3.362182	−0.424133	0.862847
83	6	0	3.572385	0.961386	1.184503
84	6	0	3.063663	−1.303173	1.904894
85	6	0	3.713040	1.340685	2.562741
86	1	0	4.113013	1.576080	0.466690
87	6	0	3.097924	−0.873904	3.231504
88	1	0	2.778195	−2.325157	1.688986
89	6	0	3.462363	0.441984	3.571400
90	1	0	3.964487	2.372298	2.787789
91	1	0	2.820407	−1.572602	4.013835
92	1	0	3.516414	0.740786	4.613196

93	1	0	3.969855	0.982041	-1.603416
94	7	0	5.583910	-0.225812	-1.332055
95	1	0	6.004435	-1.132754	-1.470917
96	6	0	6.374810	0.834640	-0.985301
97	8	0	5.906414	1.949416	-0.734974
98	6	0	7.849458	0.570267	-0.896235
99	6	0	8.490406	-0.480188	-1.569366
100	6	0	8.612727	1.446989	-0.112903
101	6	0	9.867523	-0.660874	-1.443471
102	1	0	7.932538	-1.142318	-2.226665
103	6	0	9.986474	1.260851	0.018585
104	1	0	8.106964	2.269202	0.382015
105	6	0	10.616508	0.204492	-0.644140
106	1	0	10.355691	-1.471521	-1.977045
107	1	0	10.567705	1.941237	0.634714
108	1	0	11.689055	0.061298	-0.545418
109	1	0	-1.972365	-1.822986	0.014421
110	8	0	-0.649271	-1.310789	1.219623
111	16	0	-0.951227	-0.758423	2.577870
112	8	0	0.065529	0.213481	3.058279
113	8	0	-2.357442	-0.376072	2.791579
114	6	0	-0.694932	-2.232580	3.688509
115	9	0	-1.545799	-3.215236	3.376004
116	9	0	-0.875571	-1.896707	4.971971
117	9	0	0.563185	-2.712983	3.558126

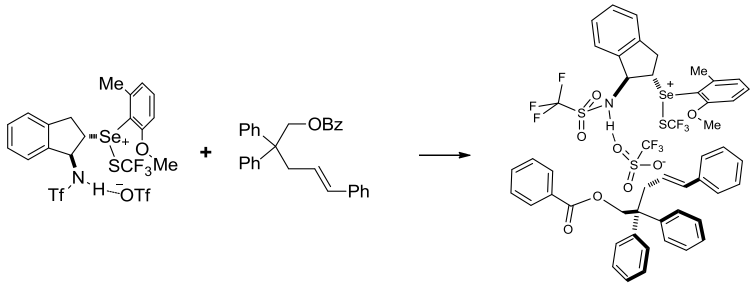

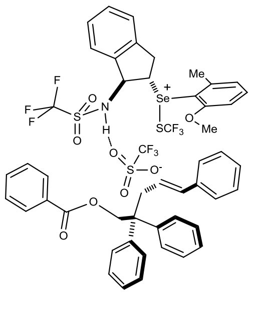

int-II'-RSS

$\Delta G_{195.15\ K}$ = -7.3 kcal/mol

$\Delta\Delta G_{195.15\ K}$ = 0.0 kcal/mol

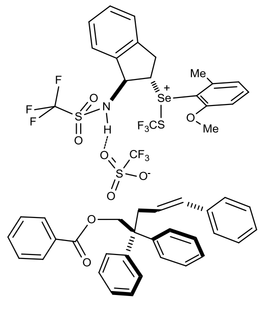

int-II'-RRR

$\Delta G_{195.15\ K}$ = -4.1 kcal/mol

$\Delta\Delta G_{195.15\ K}$ = 3.2 kcal/mol

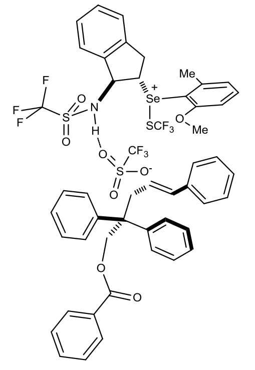

int-II'-SSS

$\Delta G_{195.15\ K}$ = - 3.8 kcal/mol

$\Delta\Delta G_{195.15\ K}$ = 3.5 kcal/mol

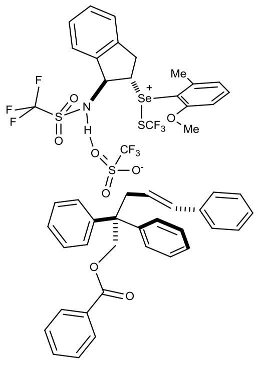

int-II'-SRR

$\Delta G_{195.15\ K}$ = -2.3 kcal/mol

$\Delta\Delta G_{195.15\ K}$ = 5.0 kcal/mol

Supplementary Figure 183. DFT calculations for **int-II'** of **1q**; ΔG related to **int-I**.

Supplementary Table 41.

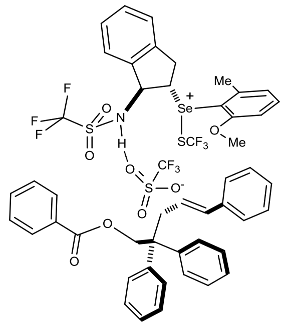

int-II'-RSS

B3LYP /6-31G(d) 195.15 K Thermal correction to Gibbs Free Energy = 0.803717

Thermal correction to Energy = 0.898261

M062x-D3/6-311+G(d,p) (IEFPCM, Dichloromethane) Energy = -7082.61724225

Standard orientation:

Center Number	Atomic Number	Atomic Type	Coordinates (Angstroms) X	Y	Z
1	6	0	-1.515349	-3.008700	0.641268
2	6	0	-1.916700	-1.717110	0.623883
3	6	0	-2.759218	-1.086337	-0.452647
4	6	0	-4.302696	-1.063490	-0.155929
5	16	0	0.902759	-1.487545	-0.833572
6	6	0	0.477140	-2.205480	-2.482230
7	9	0	1.429798	-3.086042	-2.881132
8	9	0	-0.682482	-2.861271	-2.400741
9	9	0	0.387729	-1.284339	-3.447531
10	1	0	-1.800542	-3.648315	-0.193392
11	1	0	-1.646621	-1.062778	1.450477
12	1	0	-2.419655	-0.054962	-0.572758
13	1	0	-2.610254	-1.600465	-1.406687
14	34	0	2.862752	-0.724183	-1.720673
15	6	0	6.096158	2.868633	0.821032
16	6	0	5.033216	2.238338	0.179044
17	6	0	3.896328	2.966923	-0.181892
18	6	0	3.792323	4.328090	0.082309
19	6	0	4.858671	4.960132	0.727186
20	6	0	5.999376	4.236739	1.092695
21	1	0	6.980790	2.309984	1.116145
22	1	0	2.899517	4.877407	-0.201074
23	1	0	4.798431	6.020867	0.953469
24	1	0	6.815898	4.741852	1.601210
25	6	0	2.906473	2.066213	-0.897617
26	1	0	3.073976	2.136527	-1.978754

27	6	0	3.358944	0.672211	-0.383680
28	1	0	2.858837	0.409299	0.551619
29	6	0	4.889269	0.784172	-0.229574
30	7	0	1.506486	2.386656	-0.664739
31	16	0	0.415134	2.257140	-1.880184
32	8	0	1.043798	1.545359	-3.001168
33	8	0	-0.889358	1.883267	-1.348613
34	6	0	0.257652	4.029597	-2.450896
35	9	0	1.473357	4.483994	-2.794306
36	9	0	-0.551438	4.095434	-3.506227
37	9	0	-0.224470	4.782849	-1.464441
38	1	0	5.412835	0.591434	-1.178619
39	1	0	5.277256	0.075927	0.506648
40	6	0	4.073302	-2.176063	-1.371743
41	6	0	4.219368	-2.678820	-0.062602
42	6	0	4.734850	-2.750232	-2.472532
43	6	0	5.043249	-3.790102	0.151914
44	6	0	5.562950	-3.849451	-2.223805
45	6	0	5.705986	-4.359728	-0.932771
46	1	0	5.161619	-4.201389	1.147080
47	1	0	6.091286	-4.312081	-3.051833
48	1	0	6.347417	-5.220642	-0.766333
49	8	0	3.538727	-2.021799	0.892521
50	6	0	3.478107	-2.549002	2.226333
51	1	0	2.829618	-1.863099	2.767070
52	1	0	3.038988	-3.551115	2.221515
53	1	0	4.477780	-2.570963	2.675743
54	6	0	4.561221	-2.225186	-3.880423
55	1	0	3.518235	-2.298724	-4.207078
56	1	0	4.853437	-1.171900	-3.964289
57	1	0	5.174651	-2.798402	-4.579887
58	6	0	-0.765133	-3.686594	1.709418
59	6	0	-0.411446	-5.036800	1.533286
60	6	0	-0.394964	-3.055735	2.914279
61	6	0	0.286637	-5.737135	2.516438
62	1	0	-0.696788	-5.541960	0.612950
63	6	0	0.301283	-3.756516	3.895557
64	1	0	-0.649301	-2.015370	3.087853
65	6	0	0.646829	-5.098955	3.704916
66	1	0	0.540997	-6.782165	2.357425
67	1	0	0.573029	-3.247929	4.817195
68	1	0	1.181591	-5.643169	4.479388
69	6	0	-4.549693	-0.365831	1.203978
70	1	0	-5.615488	-0.288219	1.424235

71	6	0	−4.814284	−2.518484	−0.122926
72	6	0	−4.840166	−3.252988	−1.321495
73	6	0	−5.231345	−3.169024	1.045644
74	6	0	−5.254893	−4.581911	−1.351190
75	1	0	−4.546757	−2.766279	−2.247717
76	6	0	−5.655036	−4.501934	1.019332
77	1	0	−5.235114	−2.649960	1.997733
78	6	0	−5.666191	−5.216082	−0.175763
79	1	0	−5.265357	−5.120699	−2.295450
80	1	0	−5.974896	−4.977218	1.943087
81	1	0	−5.995198	−6.251726	−0.195271
82	6	0	−5.068424	−0.275891	−1.245172
83	6	0	−6.471771	−0.350058	−1.271422
84	6	0	−4.440801	0.545059	−2.190650
85	6	0	−7.218850	0.360461	−2.206565
86	1	0	−6.986595	−0.979729	−0.551174
87	6	0	−5.188843	1.257018	−3.134161
88	1	0	−3.362717	0.653699	−2.204169
89	6	0	−6.578293	1.168336	−3.148930
90	1	0	−8.303039	0.283416	−2.197990
91	1	0	−4.673356	1.886583	−3.855032
92	1	0	−7.157982	1.723791	−3.881637
93	1	0	−4.045179	−0.900831	2.012725
94	6	0	−4.797064	2.014871	1.367825
95	8	0	−6.003553	1.927419	1.482943
96	6	0	−4.035436	3.294047	1.405819
97	6	0	−2.639617	3.325963	1.529414
98	6	0	−4.761084	4.491924	1.340752
99	6	0	−1.977597	4.552041	1.582189
100	1	0	−2.077113	2.403950	1.606398
101	6	0	−4.094122	5.713253	1.382688
102	1	0	−5.842011	4.442750	1.257495
103	6	0	−2.701183	5.743869	1.503882
104	1	0	−0.897883	4.561911	1.697205
105	1	0	−4.657647	6.640945	1.325708
106	1	0	−2.182433	6.698835	1.544213
107	1	0	1.127846	2.340510	0.304575
108	8	0	0.697797	2.263241	2.009254
109	16	0	0.612301	0.895952	2.601968
110	8	0	−0.720451	0.483015	3.070857
111	8	0	1.355383	−0.128592	1.810921
112	6	0	1.637101	1.024342	4.150889
113	9	0	2.871455	1.467204	3.861679
114	9	0	1.755561	−0.193173	4.717625

115	9	0	1.080325	1.853017	5.036517
116	8	0	-3.973608	0.947670	1.194476

Supplementary Table 42.

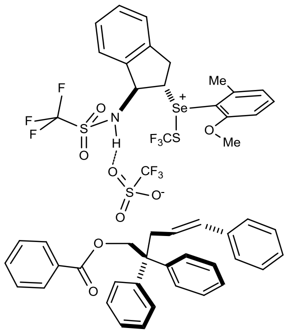

int-II'-RRR

B3LYP /6-31G(d) 195.15 K Thermal correction to Gibbs Free Energy = 0.803501

Thermal correction to Energy = 0.898502

M062x-D3/6-311+G(d,p) (IEFPCM, Dichloromethane) Energy = -7082.61184572

Standard orientation:

Center Number	Atomic Number	Atomic Type	Coordinates (Angstroms) X	Y	Z
1	6	0	1.075999	3.458847	-0.101931
2	6	0	1.780077	2.441076	-0.645553
3	6	0	3.660897	1.194257	0.639966
4	16	0	-1.273226	1.428864	-0.854017
5	6	0	-1.640201	2.253318	-2.468306
6	9	0	-2.681957	3.107195	-2.326743
7	9	0	-0.572267	2.933993	-2.868275
8	9	0	-1.990601	1.388169	-3.425833
9	1	0	0.727861	3.337942	0.923819
10	34	0	-3.361673	0.532238	-0.844848
11	6	0	-5.025801	-3.499075	2.418792
12	6	0	-4.352553	-2.732694	1.471189
13	6	0	-3.450454	-3.333710	0.591232
14	6	0	-3.198104	-4.700744	0.631406
15	6	0	-3.873637	-5.469985	1.582013
16	6	0	-4.779290	-4.873953	2.466717
17	1	0	-5.718303	-3.038753	3.118687
18	1	0	-2.485180	-5.150578	-0.052929
19	1	0	-3.687253	-6.538562	1.640818
20	1	0	-5.288311	-5.484859	3.207054
21	6	0	-2.881024	-2.300948	-0.363523

22	1	0	−3.465735	−2.304978	−1.290709
23	6	0	−3.140689	−0.990890	0.426733
24	1	0	−2.318932	−0.762426	1.102601
25	6	0	−4.440387	−1.240254	1.220543
26	7	0	−1.484466	−2.528549	−0.733393
27	16	0	−1.011115	−2.229105	−2.280946
28	8	0	−2.026853	−1.419028	−2.967124
29	8	0	0.407553	−1.893526	−2.304190
30	6	0	−1.172614	−3.925223	−3.049324
31	9	0	−2.428201	−4.365692	−2.880209
32	9	0	−0.899831	−3.854656	−4.350140
33	9	0	−0.333713	−4.781343	−2.462138
34	1	0	−5.339712	−0.990525	0.637176
35	1	0	−4.461351	−0.646429	2.137269
36	6	0	−4.342409	1.835843	0.168060
37	6	0	−3.904428	2.223141	1.452311
38	6	0	−5.472029	2.409219	−0.444416
39	6	0	−4.634088	3.194251	2.149885
40	6	0	−6.177838	3.375119	0.279069
41	6	0	−5.761535	3.755421	1.555161
42	1	0	−4.326405	3.506129	3.140294
43	1	0	−7.056764	3.832399	−0.164754
44	1	0	−6.324919	4.508055	2.099585
45	8	0	−2.794861	1.614879	1.912115
46	6	0	−2.254803	2.002015	3.188716
47	1	0	−1.342959	1.420572	3.311160
48	1	0	−2.032346	3.074540	3.193678
49	1	0	−2.960646	1.757643	3.989178
50	6	0	−5.917609	2.018534	−1.836436
51	1	0	−5.131290	2.211327	−2.574236
52	1	0	−6.171822	0.954002	−1.902978
53	1	0	−6.801786	2.590447	−2.128316
54	6	0	0.753613	4.755257	−0.714842
55	6	0	−0.178742	5.594455	−0.079054
56	6	0	1.350034	5.216359	−1.904125
57	6	0	−0.519465	6.835327	−0.614588
58	1	0	−0.639401	5.262400	0.848694
59	6	0	1.010823	6.454772	−2.440377
60	1	0	2.098223	4.607221	−2.402602
61	6	0	0.072132	7.270491	−1.801090
62	1	0	−1.243526	7.464174	−0.102864
63	1	0	1.487878	6.791143	−3.357256
64	1	0	−0.186380	8.239273	−2.219936
65	6	0	3.844094	−0.105035	1.478913

66	1	0	4.814309	−0.112260	1.977034
67	6	0	3.834706	2.410406	1.577272
68	6	0	4.457280	3.593277	1.149765
69	6	0	3.283478	2.397301	2.870820
70	6	0	4.559121	4.705172	1.987091
71	1	0	4.874241	3.649034	0.150154
72	6	0	3.389181	3.507272	3.711228
73	1	0	2.739548	1.530639	3.231112
74	6	0	4.033241	4.665979	3.277945
75	1	0	5.050969	5.604049	1.623598
76	1	0	2.960730	3.460173	4.709368
77	1	0	4.117539	5.528656	3.933950
78	6	0	4.715872	1.147411	−0.482187
79	6	0	6.076550	1.304563	−0.164740
80	6	0	4.395337	0.845480	−1.812504
81	6	0	7.068069	1.197800	−1.137696
82	1	0	6.364774	1.515460	0.860614
83	6	0	5.385784	0.737979	−2.792849
84	1	0	3.366030	0.663999	−2.100235
85	6	0	6.726728	0.919461	−2.462640
86	1	0	8.110231	1.325042	−0.856403
87	1	0	5.100993	0.500690	−3.814755
88	1	0	7.498084	0.834256	−3.223542
89	1	0	3.049491	−0.214392	2.216626
90	6	0	4.774978	−2.142084	0.631227
91	8	0	5.776941	−2.018546	1.308928
92	6	0	4.530871	−3.289570	−0.290356
93	6	0	3.335086	−3.436650	−1.006099
94	6	0	5.546592	−4.246097	−0.424132
95	6	0	3.163318	−4.533597	−1.849555
96	1	0	2.546963	−2.700546	−0.899352
97	6	0	5.371427	−5.338447	−1.269532
98	1	0	6.462154	−4.112480	0.142621
99	6	0	4.178375	−5.483531	−1.983169
100	1	0	2.232452	−4.645358	−2.396835
101	1	0	6.162042	−6.077135	−1.371945
102	1	0	4.040484	−6.337290	−2.642206
103	1	0	−0.774542	−2.258315	−0.022429
104	8	0	0.085019	−1.264234	1.177165
105	16	0	−0.087156	−1.114803	2.658260
106	8	0	0.645250	0.038129	3.228006
107	8	0	−1.492647	−1.257187	3.103082
108	6	0	0.783467	−2.612171	3.339868
109	9	0	2.086788	−2.578074	3.028858

110	9	0	0.249680	-3.727987	2.820509
111	9	0	0.660538	-2.658710	4.671651
112	1	0	2.144414	2.537200	-1.666838
113	6	0	2.176968	1.205865	0.113291
114	1	0	2.012682	0.308374	-0.492022
115	1	0	1.535639	1.097120	0.991629
116	8	0	3.747051	-1.261079	0.626022

Supplementary Table 43.

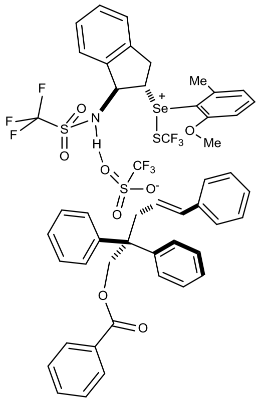

int-II’-SSS

B3LYP /6-31G(d) 195.15 K Thermal correction to Gibbs Free Energy = 0.803356

Thermal correction to Energy = 0.898462

M062x-D3/6-311+G(d,p) (IEFPCM, Dichloromethane) Energy = -7082.61139649

Standard orientation:

Center Number	Atomic Number	Atomic Type	Coordinates (Angstroms) X	Y	Z
1	6	0	1.048715	3.044059	0.315842
2	6	0	1.332596	1.730074	0.484323
3	6	0	2.203029	0.941859	-0.455137
4	6	0	3.660344	0.594580	0.047748
5	16	0	-1.348508	1.339132	-0.997863
6	6	0	-0.816284	1.811057	-2.705217
7	9	0	-1.760641	2.592871	-3.285431
8	9	0	0.320891	2.502688	-2.651124
9	9	0	-0.649554	0.756138	-3.504791
10	1	0	1.460306	3.542684	-0.561344
11	1	0	0.959296	1.205936	1.361780
12	1	0	1.696616	0.003492	-0.710899
13	1	0	2.325302	1.500632	-1.389130
14	34	0	-3.265255	0.438422	-1.865477
15	6	0	-6.375936	-3.028086	0.995209

16	6	0	−5.339448	−2.420638	0.291661
17	6	0	−4.170231	−3.131604	0.006475
18	6	0	−4.006154	−4.451572	0.410515
19	6	0	−5.046298	−5.060583	1.117642
20	6	0	−6.219676	−4.355319	1.406247
21	1	0	−7.284604	−2.481191	1.233690
22	1	0	−3.088257	−4.987029	0.187568
23	1	0	−4.939271	−6.088164	1.453225
24	1	0	−7.014859	−4.841039	1.964980
25	6	0	−3.220409	−2.263270	−0.798206
26	1	0	−3.390085	−2.440074	−1.866850
27	6	0	−3.729807	−0.849669	−0.413201
28	1	0	−3.272331	−0.503125	0.517640
29	6	0	−5.255513	−1.008759	−0.258793
30	7	0	−1.804404	−2.489054	−0.552314
31	16	0	−0.744993	−2.537960	−1.796411
32	8	0	−1.387960	−1.965641	−2.985718
33	8	0	0.576402	−2.120874	−1.341220
34	6	0	−0.613841	−4.367918	−2.152454
35	9	0	−1.838188	−4.847076	−2.423248
36	9	0	0.180637	−4.569481	−3.201315
37	9	0	−0.126019	−5.004463	−1.088718
38	1	0	−5.777687	−0.932462	−1.225098
39	1	0	−5.676349	−0.250144	0.405209
40	6	0	−4.507095	1.886964	−1.655479
41	6	0	−4.688078	2.480532	−0.388702
42	6	0	−5.160388	2.362072	−2.807343
43	6	0	−5.541272	3.584180	−0.271669
44	6	0	−6.016414	3.457163	−2.654713
45	6	0	−6.195202	4.056707	−1.407169
46	1	0	−5.687186	4.066587	0.687174
47	1	0	−6.539885	3.844797	−3.523290
48	1	0	−6.858054	4.912592	−1.315813
49	8	0	−4.008835	1.912752	0.623122
50	6	0	−4.073130	2.475431	1.944541
51	1	0	−3.494441	1.787274	2.558509
52	1	0	−3.627484	3.474684	1.953363
53	1	0	−5.111500	2.514095	2.292719
54	6	0	−4.954285	1.733566	−4.167459
55	1	0	−3.909466	1.807785	−4.488071
56	1	0	−5.219323	0.669784	−4.170582
57	1	0	−5.572686	2.233621	−4.916852
58	6	0	0.289549	3.923863	1.214044
59	6	0	0.133331	5.274652	0.850520

60	6	0	−0.276109	3.490899	2.430439
61	6	0	−0.560444	6.167759	1.664823
62	1	0	0.570417	5.624645	−0.082215
63	6	0	−0.966499	4.387358	3.243350
64	1	0	−0.179621	2.456479	2.745797
65	6	0	−1.114352	5.727025	2.867926
66	1	0	−0.662450	7.207129	1.363173
67	1	0	−1.384227	4.036589	4.183835
68	1	0	−1.647976	6.421224	3.512199
69	6	0	4.212748	−0.308197	−1.086914
70	1	0	3.610523	−1.220232	−1.131170
71	6	0	4.430133	1.921166	0.182833
72	6	0	5.201700	2.453520	−0.861180
73	6	0	4.290681	2.693787	1.348526
74	6	0	5.816876	3.702591	−0.739596
75	1	0	5.346664	1.901570	−1.783594
76	6	0	4.900844	3.941552	1.470648
77	1	0	3.703853	2.308938	2.175897
78	6	0	5.670498	4.453727	0.425124
79	1	0	6.416738	4.081694	−1.563241
80	1	0	4.772072	4.512331	2.386781
81	1	0	6.151573	5.423944	0.519192
82	6	0	3.685749	−0.236907	1.346974
83	6	0	2.602155	−1.032404	1.742086
84	6	0	4.857627	−0.295068	2.121660
85	6	0	2.672214	−1.836085	2.883122
86	1	0	1.677319	−1.044108	1.177866
87	6	0	4.934347	−1.103536	3.254755
88	1	0	5.716601	0.303164	1.836813
89	6	0	3.837899	−1.875865	3.644406
90	1	0	1.798669	−2.412290	3.171854
91	1	0	5.852392	−1.122994	3.837459
92	1	0	3.892368	−2.496759	4.535073
93	1	0	4.175371	0.180899	−2.061265
94	6	0	6.476500	−0.630742	−1.815820
95	8	0	6.236763	−0.184895	−2.923591
96	6	0	7.811762	−1.145062	−1.395964
97	6	0	8.037339	−1.695828	−0.126378
98	6	0	8.861532	−1.071857	−2.321693
99	6	0	9.306579	−2.164341	0.209635
100	1	0	7.221156	−1.754121	0.585136
101	6	0	10.127631	−1.540457	−1.980754
102	1	0	8.662833	−0.645110	−3.299307
103	6	0	10.351511	−2.087228	−0.714241

104	1	0	9.480344	-2.591226	1.193642
105	1	0	10.939678	-1.480853	-2.700332
106	1	0	11.339680	-2.453578	-0.447973
107	1	0	-1.405995	-2.311353	0.396137
108	8	0	-0.822190	-1.931843	1.978813
109	16	0	-1.334560	-0.839689	2.860387
110	8	0	-0.356866	0.217859	3.174394
111	8	0	-2.688615	-0.356001	2.473633
112	6	0	-1.610984	-1.716236	4.477474
113	9	0	-2.520199	-2.690315	4.333555
114	9	0	-2.053983	-0.854875	5.404267
115	9	0	-0.467724	-2.262586	4.913845
116	8	0	5.567452	-0.715479	-0.819582

Supplementary Table 44.

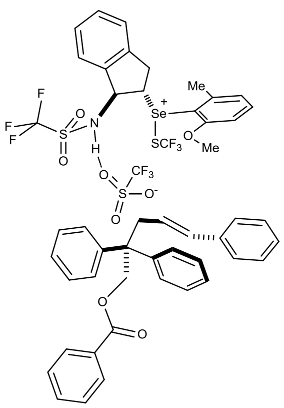

int-II-SRR

B3LYP /6-31G(d) 195.15 K Thermal correction to Gibbs Free Energy =0.803529

Thermal correction to Energy = 0.898409

M062x-D3/6-311+G(d,p) (IEFPCM, Dichloromethane) Energy = -7082.60900956

Standard orientation:

Center Number	Atomic Number	Atomic Type	Coordinates (Angstroms) X	Y	Z
1	6	0	0.825530	2.900576	0.757341
2	6	0	1.578559	1.990491	0.102146
3	6	0	1.882474	0.622943	0.649455
4	6	0	3.409173	0.246720	0.690416
5	16	0	-1.463277	1.267292	-0.792251
6	6	0	-1.227128	2.042753	-2.454107
7	9	0	-2.279544	2.839640	-2.754865
8	9	0	-0.121194	2.780018	-2.448670
9	9	0	-1.146330	1.136237	-3.434384

10	1	0	0.388493	2.597186	1.708486
11	1	0	2.011693	2.251225	-0.862153
12	1	0	1.476140	0.548713	1.659549
13	1	0	1.360026	-0.132323	0.050061
14	34	0	-3.389093	0.307975	-1.521259
15	6	0	-5.967471	-3.586606	1.312996
16	6	0	-5.045277	-2.847263	0.577690
17	6	0	-3.864380	-3.442853	0.126201
18	6	0	-3.574922	-4.776275	0.392983
19	6	0	-4.500600	-5.517793	1.131371
20	6	0	-5.685473	-4.928287	1.585491
21	1	0	-6.881626	-3.129597	1.682847
22	1	0	-2.647702	-5.220233	0.043982
23	1	0	-4.293512	-6.558411	1.363907
24	1	0	-6.389019	-5.516427	2.168109
25	6	0	-3.057316	-2.443502	-0.681666
26	1	0	-3.316994	-2.547401	-1.741908
27	6	0	-3.621841	-1.107227	-0.131142
28	1	0	-3.100972	-0.790718	0.772611
29	6	0	-5.108935	-1.390440	0.157690
30	7	0	-1.607134	-2.587677	-0.580368
31	16	0	-0.683651	-2.406052	-1.930493
32	8	0	-1.470987	-1.762088	-2.990678
33	8	0	0.644876	-1.936972	-1.552060
34	6	0	-0.484219	-4.179005	-2.486913
35	9	0	-1.700691	-4.719830	-2.655688
36	9	0	0.177441	-4.211357	-3.641535
37	9	0	0.178348	-4.876027	-1.567506
38	1	0	-5.735847	-1.264633	-0.738452
39	1	0	-5.497970	-0.727872	0.933953
40	6	0	-4.721804	1.627967	-1.117122
41	6	0	-4.859751	2.132857	0.192139
42	6	0	-5.503672	2.098583	-2.188932
43	6	0	-5.812033	3.129862	0.437413
44	6	0	-6.451045	3.087742	-1.909420
45	6	0	-6.596194	3.592389	-0.616584
46	1	0	-5.936264	3.538908	1.432556
47	1	0	-7.073837	3.467006	-2.713712
48	1	0	-7.334705	4.365582	-0.424218
49	8	0	-4.044620	1.596510	1.118272
50	6	0	-4.093766	2.076984	2.470786
51	1	0	-3.380348	1.460635	3.012129
52	1	0	-3.814509	3.135799	2.506051
53	1	0	-5.096084	1.937779	2.890208

54	6	0	−5.338491	1.572460	−3.597607
55	1	0	−4.319521	1.730820	−3.966309
56	1	0	−5.542054	0.496872	−3.659114
57	1	0	−6.026240	2.080941	−4.277587
58	6	0	0.539812	4.286395	0.357125
59	6	0	−0.456696	4.999400	1.047645
60	6	0	1.234050	4.952449	−0.670380
61	6	0	−0.767253	6.316328	0.713303
62	1	0	−0.989723	4.506823	1.858037
63	6	0	0.924557	6.267361	−1.005604
64	1	0	2.037065	4.443998	−1.195428
65	6	0	−0.080056	6.956016	−0.319199
66	1	0	−1.541738	6.845136	1.263142
67	1	0	1.477678	6.762426	−1.799576
68	1	0	−0.314733	7.984527	−0.579895
69	6	0	3.798860	−0.051881	−0.777131
70	1	0	3.162906	−0.860886	−1.147511
71	6	0	3.628191	−1.036589	1.524837
72	6	0	4.920505	−1.363388	1.971364
73	6	0	2.584610	−1.918509	1.836183
74	6	0	5.159329	−2.527444	2.699616
75	1	0	5.744343	−0.691243	1.755792
76	6	0	2.820542	−3.080708	2.576903
77	1	0	1.567471	−1.709707	1.527823
78	6	0	4.107285	−3.393117	3.008956
79	1	0	6.168637	−2.751026	3.037209
80	1	0	1.981763	−3.725691	2.824612
81	1	0	4.289668	−4.294309	3.588916
82	6	0	4.176001	1.430251	1.308539
83	6	0	4.969865	2.310037	0.561397
84	6	0	4.017406	1.690946	2.680685
85	6	0	5.591067	3.407470	1.166952
86	1	0	5.119028	2.158296	−0.502236
87	6	0	4.630794	2.786251	3.283562
88	1	0	3.404344	1.024921	3.281686
89	6	0	5.426386	3.651383	2.528154
90	1	0	6.207186	4.069477	0.563536
91	1	0	4.488786	2.962996	4.346829
92	1	0	5.910419	4.503976	2.997390
93	1	0	3.675124	0.815754	−1.426675
94	6	0	5.936525	−0.016736	−1.873108
95	8	0	5.589044	0.869531	−2.633708
96	6	0	7.274899	−0.673121	−1.915191
97	6	0	7.617299	−1.731254	−1.061087

98	6	0	8.203583	−0.206898	−2.855710
99	6	0	8.881724	−2.311535	−1.149865
100	1	0	6.894219	−2.091421	−0.337716
101	6	0	9.465564	−0.789318	−2.939566
102	1	0	7.915647	0.610118	−3.509105
103	6	0	9.806076	−1.842502	−2.086369
104	1	0	9.145735	−3.131871	−0.488030
105	1	0	10.183592	−0.424527	−3.669017
106	1	0	10.790835	−2.297994	−2.152172
107	1	0	−1.168151	−2.222189	0.288936
108	8	0	−0.894267	−1.099349	1.654020
109	16	0	−1.492667	−1.281796	3.024156
110	8	0	−2.902579	−0.824060	3.086075
111	8	0	−1.172299	−2.559100	3.669974
112	6	0	−0.582037	0.005383	4.019167
113	9	0	0.744765	−0.184100	3.970928
114	9	0	−0.971571	−0.021220	5.297479
115	9	0	−0.839566	1.244645	3.532896
116	8	0	5.160408	−0.515784	−0.887330

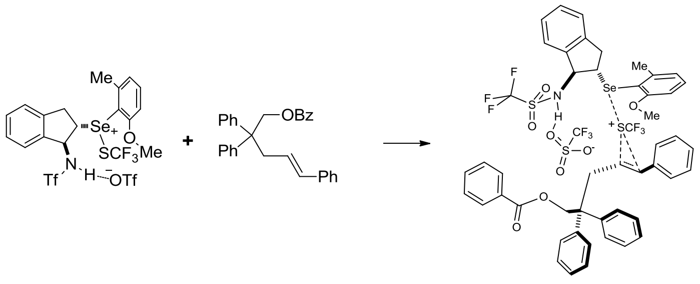

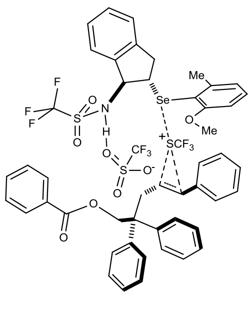

TS-I’-RSS

$\Delta G_{195.15\ K}$ = 0.5 kcal/mol

$\Delta\Delta G_{195.15\ K}$ = 0.0 kcal/mol

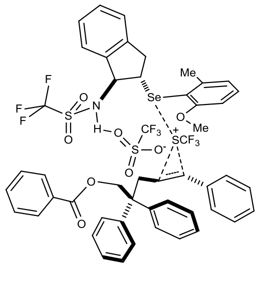

TS-I’-RRR

$\Delta G_{195.15\ K}$ = 4.4 kcal/mol

$\Delta\Delta G_{195.15\ K}$ = 3.9 kcal/mol

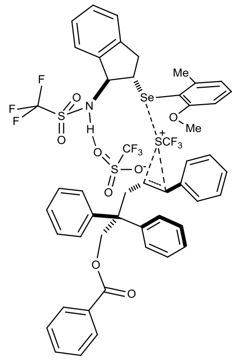

TS-I’-SSS

$\Delta G_{195.15\ K}$ = 2.8 kcal/mol

$\Delta\Delta G_{195.15\ K}$ =2.3 kcal/mol

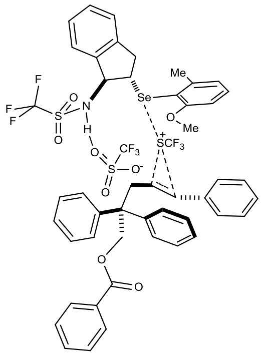

TS-I’-SRR

$\Delta G_{195.15\ K}$ = 4.5 kcal/mol

$\Delta\Delta G_{195.15\ K}$ = 4.0 kcal/mol

Supplementary Figure 184. DFT calculations for **TS-I’** of **1q**; ΔG related to **int-I**.

Supplementary Table 45.

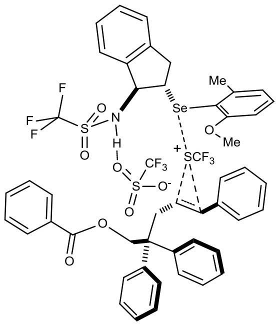

TS-I’-RSS

B3LYP /6-31G(d) 195.15 K Thermal correction to Gibbs Free Energy = 0.806225

Thermal correction to Energy = 0.898224

M062x-D3/6-311+G(d,p) (IEFPCM, Dichloromethane) Energy = -7082.60735761

Standard orientation:

Center Number	Atomic Number	Atomic Type	Coordinates (Angstroms) X	Y	Z
1	6	0	-1.315699	-2.983958	0.407075
2	6	0	-1.434108	-1.598671	0.158957
3	6	0	-2.412229	-0.986784	-0.827798
4	6	0	-3.912190	-1.078041	-0.368457
5	16	0	0.589915	-1.581123	-0.638354
6	6	0	0.290930	-2.213299	-2.357225
7	9	0	1.351335	-2.940788	-2.776290
8	9	0	-0.784221	-3.022063	-2.450291
9	9	0	0.124122	-1.215281	-3.231117
10	1	0	-1.609275	-3.661855	-0.388161
11	1	0	-1.206341	-0.940723	0.996785
12	1	0	-2.133272	0.062112	-0.933595
13	1	0	-2.336471	-1.457395	-1.808275
14	34	0	2.937734	-0.692265	-1.739942
15	6	0	6.136464	2.816033	0.995292
16	6	0	5.092826	2.198762	0.309718
17	6	0	3.976369	2.942192	-0.084632
18	6	0	3.876881	4.302403	0.187839
19	6	0	4.923716	4.920046	0.877715
20	6	0	6.043283	4.182557	1.277531
21	1	0	7.005769	2.246803	1.315645
22	1	0	3.000762	4.864150	-0.122979
23	1	0	4.864583	5.979539	1.111070
24	1	0	6.845955	4.675141	1.819832
25	6	0	3.004859	2.054034	-0.839657
26	1	0	3.188080	2.148045	-1.916449

27	6	0	3.427846	0.639944	−0.357496
28	1	0	2.897833	0.378352	0.559218
29	6	0	4.949059	0.752619	−0.123533
30	7	0	1.596780	2.375182	−0.625482
31	16	0	0.522961	2.351906	−1.855820
32	8	0	1.119447	1.683235	−3.015055
33	8	0	−0.802090	2.005124	−1.347928
34	6	0	0.422396	4.154992	−2.335656
35	9	0	1.647042	4.587068	−2.673252
36	9	0	−0.398969	4.305982	−3.373664
37	9	0	−0.020426	4.879297	−1.305957
38	1	0	5.510015	0.565253	−1.050595
39	1	0	5.302097	0.033400	0.620568
40	6	0	4.063364	−2.200843	−1.340438
41	6	0	4.166455	−2.704002	−0.027603
42	6	0	4.730456	−2.827582	−2.408211
43	6	0	4.950194	−3.835295	0.224661
44	6	0	5.507138	−3.960145	−2.131981
45	6	0	5.614530	−4.453109	−0.833755
46	1	0	5.038827	−4.229785	1.229974
47	1	0	6.030258	−4.454160	−2.945699
48	1	0	6.223532	−5.331599	−0.638393
49	8	0	3.477530	−2.025174	0.918552
50	6	0	3.420763	−2.527481	2.252522
51	1	0	2.762917	−1.840718	2.782987
52	1	0	2.997818	−3.538877	2.270165
53	1	0	4.416818	−2.535748	2.712799
54	6	0	4.610792	−2.318927	−3.826626
55	1	0	3.579198	−2.392810	−4.189833
56	1	0	4.896083	−1.263644	−3.901329
57	1	0	5.249355	−2.897579	−4.499717
58	6	0	−0.851239	−3.608151	1.623556
59	6	0	−0.683600	−5.011945	1.631706
60	6	0	−0.587646	−2.883180	2.809243
61	6	0	−0.255862	−5.669908	2.778070
62	1	0	−0.895059	−5.575752	0.726763
63	6	0	−0.156125	−3.548751	3.950737
64	1	0	−0.730321	−1.808784	2.843340
65	6	0	0.012627	−4.937800	3.940062
66	1	0	−0.132539	−6.749075	2.771375
67	1	0	0.047433	−2.976635	4.851083
68	1	0	0.345865	−5.450966	4.838259
69	6	0	−4.090149	−0.370340	0.998363
70	1	0	−5.127974	−0.427065	1.331080

71	6	0	−4.301856	−2.566246	−0.266337
72	6	0	−4.366725	−3.334252	−1.443604
73	6	0	−4.565677	−3.214760	0.948724
74	6	0	−4.668876	−4.692782	−1.407601
75	1	0	−4.194492	−2.852354	−2.401957
76	6	0	−4.876090	−4.578812	0.987961
77	1	0	−4.538407	−2.667735	1.884722
78	6	0	−4.925047	−5.324848	−0.186494
79	1	0	−4.714661	−5.257671	−2.335124
80	1	0	−5.080028	−5.051092	1.945267
81	1	0	−5.168169	−6.383560	−0.156437
82	6	0	−4.830043	−0.373804	−1.395832
83	6	0	−6.217954	−0.574376	−1.309339
84	6	0	−4.353562	0.493951	−2.386897
85	6	0	−7.096613	0.057099	−2.185419
86	1	0	−6.617018	−1.237886	−0.547414
87	6	0	−5.234152	1.125432	−3.270704
88	1	0	−3.294830	0.705119	−2.481475
89	6	0	−6.606690	0.909598	−3.177037
90	1	0	−8.165310	−0.116487	−2.091159
91	1	0	−4.835899	1.793337	−4.029973
92	1	0	−7.289252	1.403123	−3.863790
93	1	0	−3.438810	−0.806071	1.761436
94	6	0	−4.634936	1.950008	1.194098
95	8	0	−5.799434	1.697386	1.432254
96	6	0	−4.047908	3.316372	1.196748
97	6	0	−2.662689	3.528016	1.222908
98	6	0	−4.926682	4.408925	1.212723
99	6	0	−2.165046	4.829970	1.259080
100	1	0	−1.983895	2.684787	1.237153
101	6	0	−4.423024	5.706332	1.236698
102	1	0	−5.995323	4.219902	1.206558
103	6	0	−3.040730	5.917585	1.260339
104	1	0	−1.091068	4.985667	1.293585
105	1	0	−5.104678	6.552837	1.241643
106	1	0	−2.648469	6.931397	1.285951
107	1	0	1.195579	2.334023	0.329575
108	8	0	0.584761	2.278962	2.042174
109	16	0	0.397679	0.906071	2.584437
110	8	0	−1.006214	0.473586	2.765243
111	8	0	1.270595	−0.116804	1.944264
112	6	0	1.066388	0.998898	4.319218
113	9	0	2.335941	1.426249	4.314818
114	9	0	1.041851	−0.232260	4.872991

115	9	0	0.336999	1.819258	5.078974
116	8	0	-3.702680	1.002549	0.898808

Supplementary Table 46.

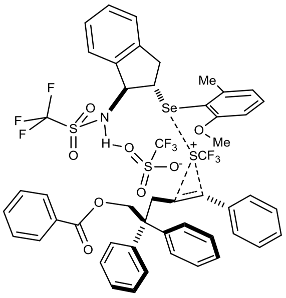

TS-I'-RRR

B3LYP /6-31G(d) 195.15 K Thermal correction to Gibbs Free Energy = 0.807286

Thermal correction to Energy = 0.898424

M062x-D3/6-311+G(d,p) (IEFPCM, Dichloromethane) Energy = -7082.6021407

Standard orientation:

Center Number	Atomic Number	Atomic Type	Coordinates (Angstroms) X	Y	Z
1	6	0	-0.591858	-2.750326	0.275591
2	6	0	-1.297779	-1.862691	-0.558204
3	6	0	-2.074230	-0.706696	0.038275
4	6	0	-3.490753	-1.117954	0.580446
5	16	0	0.668351	-0.940783	-1.190705
6	6	0	0.316509	-1.451187	-2.931260
7	9	0	0.139231	-2.784359	-3.025822
8	9	0	-0.801888	-0.870908	-3.407377
9	9	0	1.315701	-1.130857	-3.774560
10	1	0	-0.219351	-2.339691	1.212890
11	1	0	-1.690755	-2.269049	-1.485657
12	1	0	-1.495773	-0.290472	0.866253
13	1	0	-2.184720	0.099120	-0.688456
14	34	0	2.811646	0.548772	-1.963048
15	6	0	5.942777	2.890149	1.872309
16	6	0	4.891357	2.498346	1.047864
17	6	0	3.659749	3.154701	1.122820
18	6	0	3.448655	4.204893	2.009189
19	6	0	4.504092	4.595693	2.837961
20	6	0	5.740023	3.944243	2.768549
21	1	0	6.901783	2.379513	1.832342
22	1	0	2.482309	4.698011	2.060744

23	1	0	4.361188	5.407063	3.546362
24	1	0	6.548653	4.253974	3.425174
25	6	0	2.705596	2.573323	0.096190
26	1	0	2.758396	3.171800	-0.820996
27	6	0	3.328811	1.177341	-0.158699
28	1	0	2.975704	0.465983	0.585813
29	6	0	4.847890	1.400952	0.002067
30	7	0	1.299412	2.554044	0.508144
31	16	0	0.130892	2.991489	-0.559118
32	8	0	0.616568	2.903381	-1.939623
33	8	0	-1.137504	2.403406	-0.137615
34	6	0	-0.026283	4.823680	-0.228574
35	9	0	1.149268	5.424065	-0.461493
36	9	0	-0.952473	5.341349	-1.040657
37	9	0	-0.378806	5.037737	1.038595
38	1	0	5.303973	1.736150	-0.940785
39	1	0	5.360059	0.485567	0.309244
40	6	0	4.076703	-0.851313	-2.334329
41	6	0	4.378274	-1.848042	-1.382749
42	6	0	4.663398	-0.874253	-3.614017
43	6	0	5.274785	-2.871923	-1.712453
44	6	0	5.552663	-1.911424	-3.920025
45	6	0	5.852415	-2.894193	-2.980025
46	1	0	5.516166	-3.643783	-0.991829
47	1	0	6.014045	-1.940204	-4.902891
48	1	0	6.546709	-3.690742	-3.233355
49	8	0	3.763563	-1.736573	-0.182198
50	6	0	4.008074	-2.725904	0.820589
51	1	0	3.417614	-2.418947	1.681359
52	1	0	3.685488	-3.714800	0.472851
53	1	0	5.071027	-2.755309	1.088158
54	6	0	4.354922	0.181238	-4.652001
55	1	0	3.298770	0.163984	-4.944751
56	1	0	4.560972	1.188960	-4.274093
57	1	0	4.955957	0.022317	-5.551527
58	6	0	-0.257907	-4.131304	0.046187
59	6	0	0.591570	-4.753684	0.991873
60	6	0	-0.765959	-4.898939	-1.027289
61	6	0	0.935794	-6.094352	0.853597
62	1	0	0.965525	-4.162536	1.823034
63	6	0	-0.416951	-6.235027	-1.157460
64	1	0	-1.439770	-4.448359	-1.747467
65	6	0	0.436077	-6.834687	-0.220679
66	1	0	1.587107	-6.564892	1.584461

67	1	0	−0.812521	−6.819685	−1.982892
68	1	0	0.701417	−7.883145	−0.327294
69	6	0	−4.058240	0.108108	1.357961
70	1	0	−5.010601	−0.138444	1.828185
71	6	0	−4.498975	−1.447752	−0.538896
72	6	0	−4.214841	−1.279073	−1.901212
73	6	0	−5.799951	−1.859822	−0.197212
74	6	0	−5.177243	−1.538959	−2.881823
75	1	0	−3.245070	−0.921013	−2.226658
76	6	0	−6.761767	−2.120019	−1.170456
77	1	0	−6.067844	−1.977435	0.848194
78	6	0	−6.453121	−1.965802	−2.523362
79	1	0	−4.922185	−1.395047	−3.928548
80	1	0	−7.757167	−2.434294	−0.868214
81	1	0	−7.202124	−2.165093	−3.285040
82	6	0	−3.304081	−2.267938	1.598504
83	6	0	−3.754079	−3.577779	1.366374
84	6	0	−2.587555	−2.030510	2.787102
85	6	0	−3.522679	−4.597255	2.291982
86	1	0	−4.291612	−3.809342	0.454053
87	6	0	−2.350122	−3.048227	3.710069
88	1	0	−2.172478	−1.050977	2.996350
89	6	0	−2.824655	−4.338089	3.471133
90	1	0	−3.887815	−5.599484	2.082317
91	1	0	−1.776018	−2.823447	4.603721
92	1	0	−2.643015	−5.133030	4.189463
93	1	0	−3.352389	0.441354	2.121460
94	6	0	−5.491632	1.751882	0.367104
95	8	0	−6.470724	1.293805	0.922646
96	6	0	−5.510598	2.960395	−0.503988
97	6	0	−4.335185	3.550236	−0.989869
98	6	0	−6.756234	3.524735	−0.812216
99	6	0	−4.412615	4.697460	−1.778610
100	1	0	−3.372809	3.118449	−0.740728
101	6	0	−6.827561	4.665246	−1.607605
102	1	0	−7.651976	3.055843	−0.418230
103	6	0	−5.655151	5.253562	−2.091007
104	1	0	−3.498619	5.158301	−2.142553
105	1	0	−7.794605	5.098573	−1.848379
106	1	0	−5.711001	6.147076	−2.707753
107	1	0	0.997759	1.821069	1.175348
108	8	0	0.579594	0.194833	1.995737
109	16	0	1.460084	−0.582788	2.928176
110	8	0	1.205941	−2.043582	2.893180

111	8	0	2.877649	-0.177561	2.896307
112	6	0	0.852560	-0.064498	4.610983
113	9	0	0.962028	1.260649	4.760739
114	9	0	1.560109	-0.667647	5.572708
115	9	0	-0.444232	-0.400775	4.768497
116	8	0	-4.242594	1.228570	0.479497

Supplementary Table 47.

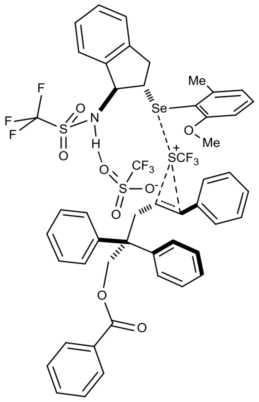

TS-I'-SSS

B3LYP /6-31G(d)   195.15 K   Thermal correction to Gibbs Free Energy = 0.805997

Thermal correction to Energy = 0.898204

M062x-D3/6-311+G(d,p)   (IEFPCM, Dichloromethane)   Energy = -7082.60354363

Standard orientation:

Center Number	Atomic Number	Atomic Type	Coordinates (Angstroms) X	Y	Z
1	6	0	1.259422	2.410196	0.244596
2	6	0	1.192595	0.992553	0.189862
3	6	0	2.171501	0.148347	-0.607652
4	6	0	3.632508	0.031746	-0.019157
5	16	0	-0.684278	1.146438	-0.804684
6	6	0	-0.141201	1.460450	-2.553832
7	9	0	-1.047035	2.241450	-3.181102
8	9	0	1.041120	2.105933	-2.646177
9	9	0	-0.036624	0.326289	-3.253513
10	1	0	1.710684	2.915394	-0.603483
11	1	0	0.809598	0.503629	1.084088
12	1	0	1.738706	-0.849799	-0.716448
13	1	0	2.289515	0.561924	-1.609644
14	34	0	-3.048398	0.362299	-2.094822
15	6	0	-7.152857	-2.109332	0.538308

16	6	0	−5.918004	−1.769752	−0.010257
17	6	0	−4.885706	−2.710680	−0.047978
18	6	0	−5.057273	−3.997098	0.452863
19	6	0	−6.295520	−4.335999	1.004709
20	6	0	−7.334028	−3.399327	1.046336
21	1	0	−7.961236	−1.383345	0.581016
22	1	0	−4.240976	−4.713149	0.422856
23	1	0	−6.450594	−5.331792	1.410732
24	1	0	−8.289324	−3.674768	1.485243
25	6	0	−3.660848	−2.116775	−0.718122
26	1	0	−3.643761	−2.408724	−1.774791
27	6	0	−3.930237	−0.590192	−0.596557
28	1	0	−3.511311	−0.205469	0.333589
29	6	0	−5.469397	−0.461988	−0.630443
30	7	0	−2.399782	−2.570335	−0.132863
31	16	0	−1.090604	−2.858149	−1.061205
32	8	0	−1.283806	−2.283737	−2.395549
33	8	0	0.125727	−2.625356	−0.282916
34	6	0	−1.173265	−4.707080	−1.315823
35	9	0	−2.342718	−5.017490	−1.894393
36	9	0	−0.173594	−5.101462	−2.104820
37	9	0	−1.090772	−5.336099	−0.142895
38	1	0	−5.838369	−0.375799	−1.662413
39	1	0	−5.815812	0.423188	−0.089343
40	6	0	−3.975065	2.049327	−2.146590
41	6	0	−4.156835	2.816070	−0.977391
42	6	0	−4.408923	2.528026	−3.395736
43	6	0	−4.792993	4.060285	−1.052570
44	6	0	−5.038713	3.778419	−3.447710
45	6	0	−5.229429	4.529414	−2.290675
46	1	0	−4.942771	4.656747	−0.160353
47	1	0	−5.381526	4.159075	−4.405592
48	1	0	−5.723196	5.495712	−2.348723
49	8	0	−3.686512	2.267569	0.167934
50	6	0	−3.737816	3.017264	1.379577
51	1	0	−3.260165	2.385262	2.125979
52	1	0	−3.185087	3.959107	1.278706
53	1	0	−4.775108	3.228817	1.668427
54	6	0	−4.192762	1.736700	−4.665456
55	1	0	−3.124615	1.617660	−4.882769
56	1	0	−4.611459	0.727345	−4.586297
57	1	0	−4.657691	2.238953	−5.518389
58	6	0	0.780612	3.264425	1.302594
59	6	0	0.811473	4.662927	1.091404

60	6	0	0.311955	2.770033	2.541916
61	6	0	0.377218	5.539050	2.076966
62	1	0	1.181506	5.047429	0.144413
63	6	0	-0.125648	3.654795	3.521117
64	1	0	0.295249	1.705286	2.749383
65	6	0	-0.096477	5.034712	3.294029
66	1	0	0.406242	6.610868	1.903284
67	1	0	-0.494128	3.259530	4.462547
68	1	0	-0.436858	5.718796	4.066928
69	6	0	4.312542	-0.951933	-1.015668
70	1	0	3.840475	-1.932580	-0.907460
71	6	0	4.263380	1.437352	-0.060046
72	6	0	4.851485	1.947588	-1.229208
73	6	0	4.169738	2.296831	1.048990
74	6	0	5.332988	3.257055	-1.282883
75	1	0	4.960784	1.325427	-2.110833
76	6	0	4.648927	3.607754	0.995813
77	1	0	3.734094	1.932567	1.973029
78	6	0	5.233546	4.095807	-0.171906
79	1	0	5.793071	3.616742	-2.199509
80	1	0	4.563485	4.242813	1.873649
81	1	0	5.613287	5.113172	-0.214883
82	6	0	3.706387	-0.614749	1.378295
83	6	0	2.671220	-1.406147	1.891265
84	6	0	4.887576	-0.507339	2.135314
85	6	0	2.794002	-2.039579	3.131450
86	1	0	1.749316	-1.553546	1.341607
87	6	0	5.016553	-1.148045	3.365803
88	1	0	5.712568	0.087059	1.757662
89	6	0	3.964935	-1.914519	3.873760
90	1	0	1.955052	-2.613505	3.511524
91	1	0	5.939608	-1.042010	3.930635
92	1	0	4.058460	-2.404191	4.839505
93	1	0	4.212707	-0.628660	-2.052619
94	6	0	6.604302	-0.985688	-1.720723
95	8	0	6.307032	-0.669535	-2.857835
96	6	0	7.995644	-1.262806	-1.266678
97	6	0	8.289594	-1.682269	0.038802
98	6	0	9.030907	-1.099669	-2.197598
99	6	0	9.611227	-1.931917	0.404744
100	1	0	7.485220	-1.812708	0.754090
101	6	0	10.349409	-1.349171	-1.826434
102	1	0	8.780123	-0.778442	-3.203154
103	6	0	10.640886	-1.765439	-0.524554

104	1	0	9.838165	-2.258571	1.415844
105	1	0	11.149677	-1.220934	-2.550042
106	1	0	11.669979	-1.961533	-0.234929
107	1	0	-2.202723	-2.381082	0.869358
108	8	0	-1.959570	-2.005115	2.607282
109	16	0	-1.524840	-0.605928	2.859255
110	8	0	-0.143851	-0.436218	3.357244
111	8	0	-1.903473	0.346074	1.777634
112	6	0	-2.595260	-0.055858	4.279485
113	9	0	-3.893705	-0.184720	3.970892
114	9	0	-2.363982	1.250250	4.542653
115	9	0	-2.343834	-0.758208	5.387246
116	8	0	5.706122	-1.126387	-0.715223

Supplementary Table 48.

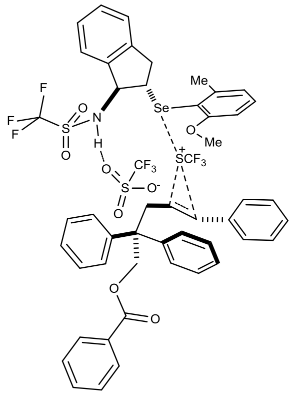

TS-I'-SRR

B3LYP /6-31G(d) 195.15 K Thermal correction to Gibbs Free Energy = 0.805722

Thermal correction to Energy = 0.898379

M062x-D3/6-311+G(d,p) (IEFPCM, Dichloromethane) Energy = -7082.60044423

Standard orientation:

Center Number	Atomic Number	Atomic Type	Coordinates (Angstroms) X	Y	Z
1	6	0	1.175894	1.812568	0.784079
2	6	0	1.437344	1.178516	-0.445166
3	6	0	1.802742	-0.292540	-0.486986
4	6	0	3.316674	-0.564355	-0.126208
5	16	0	-0.771868	1.229683	-0.936446
6	6	0	-0.390920	2.272400	-2.414614
7	9	0	0.252104	3.405277	-2.064055
8	9	0	0.399010	1.625635	-3.289532
9	9	0	-1.499443	2.646050	-3.081178

10	1	0	0.762725	1.183172	1.571369
11	1	0	1.861297	1.790499	−1.237253
12	1	0	1.164617	−0.845482	0.206056
13	1	0	1.589228	−0.672064	−1.489052
14	34	0	−3.367416	0.936502	−1.705403
15	6	0	−6.806092	−1.696106	1.657310
16	6	0	−5.768360	−1.340173	0.800107
17	6	0	−4.876800	−2.312368	0.336710
18	6	0	−4.997219	−3.645916	0.710623
19	6	0	−6.037951	−4.001333	1.573411
20	6	0	−6.934319	−3.034739	2.041055
21	1	0	−7.498204	−0.947240	2.034389
22	1	0	−4.291273	−4.387154	0.347858
23	1	0	−6.147164	−5.035330	1.888916
24	1	0	−7.733415	−3.325290	2.717838
25	6	0	−3.879063	−1.676292	−0.613090
26	1	0	−4.249770	−1.775541	−1.640345
27	6	0	−3.941724	−0.189508	−0.183505
28	1	0	−3.280695	−0.013370	0.663221
29	6	0	−5.410643	0.022931	0.237670
30	7	0	−2.535692	−2.259926	−0.591453
31	16	0	−1.755076	−2.572113	−2.000928
32	8	0	−2.345343	−1.813043	−3.106335
33	8	0	−0.316205	−2.595967	−1.755653
34	6	0	−2.245523	−4.346354	−2.321634
35	9	0	−3.579777	−4.427218	−2.424526
36	9	0	−1.689235	−4.764625	−3.458441
37	9	0	−1.841698	−5.124958	−1.316487
38	1	0	−6.041584	0.271271	−0.628147
39	1	0	−5.510389	0.833896	0.963537
40	6	0	−4.046943	2.682085	−1.274394
41	6	0	−3.887587	3.241506	0.011133
42	6	0	−4.674764	3.407828	−2.304807
43	6	0	−4.362373	4.533414	0.268033
44	6	0	−5.137420	4.698665	−2.022301
45	6	0	−4.982310	5.250323	−0.753025
46	1	0	−4.245339	4.975982	1.249647
47	1	0	−5.626568	5.268847	−2.806752
48	1	0	−5.348717	6.253173	−0.551075
49	8	0	−3.273150	2.463250	0.932042
50	6	0	−3.086313	2.963422	2.258206
51	1	0	−2.618167	2.150941	2.810459
52	1	0	−2.432902	3.844275	2.249981
53	1	0	−4.048601	3.219237	2.717462

54	6	0	−4.854646	2.832862	−3.691751
55	1	0	−3.891446	2.657686	−4.185000
56	1	0	−5.374333	1.868676	−3.663364
57	1	0	−5.433425	3.516944	−4.318435
58	6	0	1.332594	3.197795	1.138939
59	6	0	0.837103	3.597058	2.403341
60	6	0	1.990053	4.152154	0.328172
61	6	0	0.976913	4.913556	2.830073
62	1	0	0.347921	2.854780	3.027687
63	6	0	2.125071	5.462590	0.761994
64	1	0	2.402799	3.860729	−0.631198
65	6	0	1.616885	5.846861	2.010566
66	1	0	0.595123	5.211901	3.802173
67	1	0	2.634396	6.189612	0.136313
68	1	0	1.731106	6.874497	2.345216
69	6	0	4.141796	0.041655	−1.286956
70	1	0	3.793377	−0.397113	−2.227626
71	6	0	3.600678	−2.084448	−0.073376
72	6	0	4.733088	−2.552444	0.615744
73	6	0	2.801617	−3.023959	−0.737149
74	6	0	5.055081	−3.908075	0.638522
75	1	0	5.364475	−1.847065	1.145372
76	6	0	3.122532	−4.384586	−0.711498
77	1	0	1.907548	−2.722542	−1.267300
78	6	0	4.249153	−4.834183	−0.027509
79	1	0	5.933741	−4.240990	1.185562
80	1	0	2.475681	−5.089900	−1.226513
81	1	0	4.494472	−5.892814	−0.005331
82	6	0	3.610831	0.077466	1.241166
83	6	0	4.449712	1.187745	1.418357
84	6	0	2.966871	−0.449869	2.375932
85	6	0	4.641792	1.743816	2.686923
86	1	0	4.959071	1.642378	0.575762
87	6	0	3.145503	0.112578	3.636805
88	1	0	2.308155	−1.305858	2.271152
89	6	0	3.992296	1.211875	3.799393
90	1	0	5.301700	2.600474	2.797090
91	1	0	2.609917	−0.306275	4.483231
92	1	0	4.139955	1.650117	4.782898
93	1	0	4.041459	1.126186	−1.349198
94	6	0	6.444735	0.693400	−1.430760
95	8	0	6.145178	1.849056	−1.673868
96	6	0	7.843996	0.187371	−1.367766
97	6	0	8.142276	−1.158800	−1.111534

98	6	0	8.881971	1.105028	-1.581807
99	6	0	9.471297	-1.576780	-1.068216
100	1	0	7.336068	-1.865234	-0.948030
101	6	0	10.207620	0.681889	-1.537180
102	1	0	8.628040	2.140875	-1.781419
103	6	0	10.503508	-0.659644	-1.280191
104	1	0	9.702004	-2.619782	-0.869821
105	1	0	11.010212	1.395206	-1.703052
106	1	0	11.538390	-0.990112	-1.246042
107	1	0	-1.911770	-2.007102	0.195058
108	8	0	-0.912087	-1.067988	1.462584
109	16	0	-1.321089	-0.534951	2.803263
110	8	0	-0.507897	0.624102	3.245125
111	8	0	-2.779085	-0.398631	2.979941
112	6	0	-0.824707	-1.896620	3.972837
113	9	0	-1.427583	-3.044315	3.640339
114	9	0	-1.163479	-1.579354	5.228006
115	9	0	0.507663	-2.092113	3.932219
116	8	0	5.539974	-0.280726	-1.175688

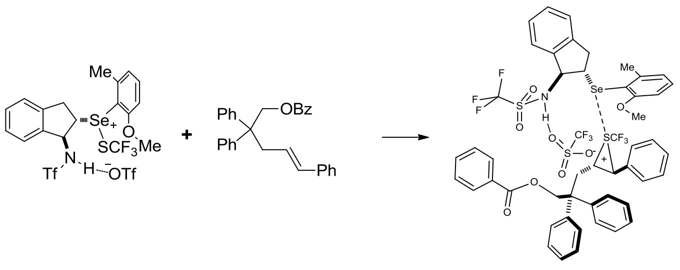

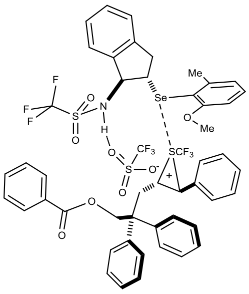

int-III’-RSS

$\Delta G_{195.15\ K}$ = -5.4 kcal/mol

$\Delta\Delta G_{195.15\ K}$ = 0.0 kcal/mol

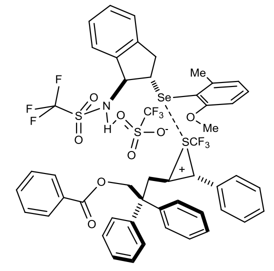

int-III’-RRR

$\Delta G_{195.15\ K}$ = -5.5 kcal/mol

$\Delta\Delta G_{195.15\ K}$ = -0.1 kcal/mol

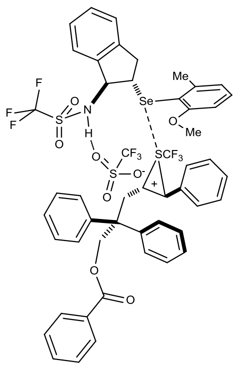

int-III’-SSS

$\Delta G_{195.15\ K}$ = -2.4 kcal/mol

$\Delta\Delta G_{195.15\ K}$ = 3.0 kcal/mol

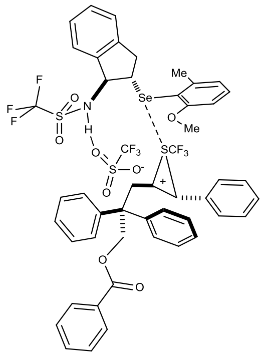

int-III’-SRR

$\Delta G_{195.15\ K}$ = -5.6 kcal/mol

$\Delta\Delta G_{195.15\ K}$ = -0.2 kcal/mol

Supplementary Figure 185. DFT calculations for **int-III’** of **1q**; ΔG related to **int-I**.

Supplementary Table 49.

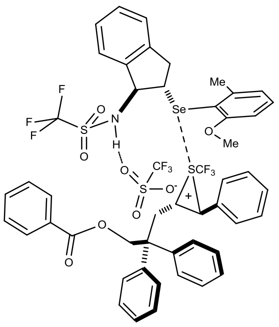

int-III'-RSS

B3LYP /6-31G(d) 195.15 K Thermal correction to Gibbs Free Energy = 0.804913

Thermal correction to Energy = 0.898989

M062x-D3/6-311+G(d,p) (IEFPCM, Dichloromethane) Energy = -7082.6153137

Standard orientation:

Center Number	Atomic Number	Atomic Type	Coordinates (Angstroms) X	Y	Z
1	6	0	-0.796210	-3.095835	-0.258058
2	6	0	-1.104280	-1.683259	-0.182558
3	6	0	-2.297059	-1.100939	-0.933625
4	6	0	-3.673322	-1.352238	-0.220319
5	16	0	0.670043	-1.362825	-0.968777
6	6	0	0.286538	-1.472177	-2.784513
7	9	0	1.419994	-1.725609	-3.457680
8	9	0	-0.575969	-2.468359	-3.095369
9	9	0	-0.236229	-0.334597	-3.246500
10	1	0	-1.018978	-3.582208	-1.201671
11	1	0	-0.958232	-1.235168	0.800664
12	1	0	-2.137579	-0.024367	-1.033597
13	1	0	-2.349763	-1.532849	-1.934076
14	34	0	3.138439	0.204219	-1.914189
15	6	0	6.027557	3.155354	1.694434
16	6	0	5.036038	2.626186	0.871316
17	6	0	3.825943	3.307820	0.706223
18	6	0	3.582067	4.518321	1.346499
19	6	0	4.577034	5.046122	2.174208
20	6	0	5.789230	4.369613	2.346171
21	1	0	6.969383	2.630881	1.837304
22	1	0	2.635504	5.033586	1.210275
23	1	0	4.404785	5.985199	2.692947
24	1	0	6.551116	4.788347	2.998411
25	6	0	2.932687	2.547537	-0.256149
26	1	0	3.042878	2.962067	-1.265313

27	6	0	3.540668	1.119359	-0.208337
28	1	0	3.080609	0.547721	0.597630
29	6	0	5.043707	1.350765	0.051107
30	7	0	1.507614	2.613347	0.070475
31	16	0	0.367513	2.730967	-1.088837
32	8	0	0.966433	2.525866	-2.408889
33	8	0	-0.844711	2.029229	-0.666586
34	6	0	-0.075400	4.545068	-1.038556
35	9	0	1.026987	5.280654	-1.231291
36	9	0	-0.960441	4.821512	-2.003145
37	9	0	-0.606113	4.856473	0.144942
38	1	0	5.585690	1.496774	-0.893890
39	1	0	5.506834	0.501872	0.562889
40	6	0	4.337088	-1.305016	-1.928618
41	6	0	4.503171	-2.131488	-0.798221
42	6	0	5.006492	-1.604071	-3.129489
43	6	0	5.350047	-3.243393	-0.858747
44	6	0	5.842098	-2.728716	-3.172408
45	6	0	6.014326	-3.533169	-2.050150
46	1	0	5.488181	-3.878129	0.008672
47	1	0	6.363740	-2.964962	-4.095744
48	1	0	6.671715	-4.397233	-2.097416
49	8	0	3.802416	-1.771953	0.308176
50	6	0	3.861958	-2.590513	1.471384
51	1	0	3.191032	-2.117463	2.186791
52	1	0	3.514419	-3.608252	1.253357
53	1	0	4.881820	-2.632453	1.875106
54	6	0	4.831058	-0.750260	-4.364254
55	1	0	3.805099	-0.805848	-4.748692
56	1	0	5.024898	0.306001	-4.147810
57	1	0	5.507069	-1.075742	-5.160215
58	6	0	-0.260571	-3.944121	0.769180
59	6	0	0.068735	-5.275918	0.419249
60	6	0	-0.082662	-3.522178	2.108069
61	6	0	0.568474	-6.154767	1.369694
62	1	0	-0.073100	-5.605458	-0.606653
63	6	0	0.422640	-4.408233	3.051459
64	1	0	-0.349888	-2.516102	2.409940
65	6	0	0.749779	-5.719294	2.687826
66	1	0	0.817259	-7.174517	1.091279
67	1	0	0.562906	-4.070308	4.073321
68	1	0	1.142671	-6.405467	3.433406
69	6	0	-3.791940	-0.463391	1.048967
70	1	0	-4.674740	-0.741306	1.625420

71	6	0	−3.781052	−2.844688	0.153420
72	6	0	−3.910931	−3.801446	−0.871440
73	6	0	−3.738176	−3.307717	1.476683
74	6	0	−3.990066	−5.162994	−0.585904
75	1	0	−3.978453	−3.468144	−1.903597
76	6	0	−3.824533	−4.672864	1.765611
77	1	0	−3.633105	−2.611865	2.301646
78	6	0	−3.948047	−5.606280	0.738618
79	1	0	−4.099672	−5.876990	−1.398245
80	1	0	−3.793799	−5.000749	2.801101
81	1	0	−4.019377	−6.666576	0.965254
82	6	0	−4.836893	−0.975737	−1.168492
83	6	0	−6.143398	−1.363670	−0.829263
84	6	0	−4.659709	−0.214375	−2.330902
85	6	0	−7.230280	−1.015774	−1.627948
86	1	0	−6.313618	−1.945230	0.071936
87	6	0	−5.748250	0.132376	−3.136255
88	1	0	−3.674347	0.131845	−2.621940
89	6	0	−7.037028	−0.267760	−2.790928
90	1	0	−8.229814	−1.328423	−1.337884
91	1	0	−5.580755	0.724732	−4.031894
92	1	0	−7.883181	0.004353	−3.416101
93	1	0	−2.905485	−0.527355	1.685146
94	6	0	−5.043361	1.566309	1.074771
95	8	0	−5.939221	1.035259	1.702188
96	6	0	−5.055244	2.995227	0.656934
97	6	0	−3.965067	3.604454	0.021089
98	6	0	−6.211997	3.739360	0.930042
99	6	0	−4.040586	4.949339	−0.340840
100	1	0	−3.068093	3.029675	−0.178647
101	6	0	−6.282401	5.080371	0.564039
102	1	0	−7.040595	3.247373	1.428514
103	6	0	−5.195934	5.686454	−0.072968
104	1	0	−3.194509	5.419834	−0.831194
105	1	0	−7.180989	5.653800	0.775405
106	1	0	−5.249331	6.734248	−0.357966
107	1	0	1.171988	2.267852	0.988000
108	8	0	0.612935	1.686350	2.630848
109	16	0	0.515527	0.211900	2.754482
110	8	0	−0.853919	−0.344495	2.884128
111	8	0	1.372574	−0.539948	1.796666
112	6	0	1.298028	−0.165928	4.400952
113	9	0	2.549173	0.308669	4.448751
114	9	0	1.356604	−1.505596	4.574266

115	9	0	0.596744	0.358574	5.408270
116	8	0	-3.914547	0.918815	0.677611

Supplementary Table 50.

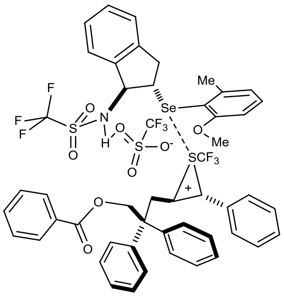

int-III'-RRR

B3LYP /6-31G(d) 195.15 K Thermal correction to Gibbs Free Energy = 0.805975

Thermal correction to Energy = 0.899468

M062x-D3/6-311+G(d,p) (IEFPCM, Dichloromethane) Energy = -7082.61653594

Standard orientation:

Center Number	Atomic Number	Atomic Type	Coordinates (Angstroms) X	Y	Z
1	6	0	0.620965	2.818197	0.267459
2	6	0	1.203435	1.936547	-0.733592
3	6	0	2.045136	0.770004	-0.204495
4	6	0	3.478156	1.158470	0.275617
5	16	0	-0.494296	1.167558	-1.241065
6	6	0	-1.047104	2.406557	-2.490161
7	9	0	-1.774897	3.396659	-1.946486
8	9	0	-0.006741	2.987006	-3.128032
9	9	0	-1.799254	1.798591	-3.411433
10	1	0	0.230701	2.331803	1.159810
11	1	0	1.639908	2.429724	-1.598066
12	1	0	1.505110	0.316759	0.631284
13	1	0	2.110307	0.000714	-0.975989
14	34	0	-2.947935	-0.787494	-2.006297
15	6	0	-5.639717	-3.096171	2.191030
16	6	0	-4.681043	-2.685792	1.268118
17	6	0	-3.402306	-3.250778	1.288981
18	6	0	-3.055420	-4.226403	2.217489
19	6	0	-4.018227	-4.635113	3.144781
20	6	0	-5.299599	-4.074950	3.130094
21	1	0	-6.633769	-2.655566	2.192365
22	1	0	-2.055234	-4.649533	2.229672

23	1	0	−3.766747	−5.388220	3.886656
24	1	0	−6.035789	−4.396698	3.862053
25	6	0	−2.568021	−2.664812	0.164232
26	1	0	−2.618960	−3.320481	−0.713202
27	6	0	−3.310561	−1.340824	−0.141521
28	1	0	−2.959779	−0.561599	0.533780
29	6	0	−4.790558	−1.660956	0.156838
30	7	0	−1.143740	−2.500183	0.484273
31	16	0	0.015936	−3.008476	−0.538707
32	8	0	−0.475075	−3.094582	−1.912975
33	8	0	1.265298	−2.314311	−0.205694
34	6	0	0.299246	−4.782355	−0.021936
35	9	0	−0.834006	−5.478732	−0.175273
36	9	0	1.254507	−5.319201	−0.785340
37	9	0	0.672129	−4.836698	1.257871
38	1	0	−5.282997	−2.090478	−0.726707
39	1	0	−5.352337	−0.767111	0.442469
40	6	0	−4.396654	0.440521	−2.328916
41	6	0	−4.612128	1.532022	−1.461996
42	6	0	−5.201051	0.270678	−3.469042
43	6	0	−5.645320	2.439413	−1.717381
44	6	0	−6.224672	1.197379	−3.713867
45	6	0	−6.445112	2.262579	−2.846323
46	1	0	−5.822453	3.276509	−1.052485
47	1	0	−6.854469	1.071448	−4.590210
48	1	0	−7.247366	2.967982	−3.046099
49	8	0	−3.768062	1.622985	−0.399460
50	6	0	−3.942780	2.678508	0.541972
51	1	0	−3.188813	2.512132	1.311058
52	1	0	−3.790442	3.655503	0.065982
53	1	0	−4.940665	2.641536	0.995190
54	6	0	−4.977810	−0.873999	−4.429784
55	1	0	−4.031944	−0.762153	−4.974766
56	1	0	−4.916701	−1.830362	−3.900154
57	1	0	−5.784845	−0.927104	−5.166598
58	6	0	0.407903	4.224376	0.188210
59	6	0	−0.319213	4.826291	1.249032
60	6	0	0.913951	5.042050	−0.855101
61	6	0	−0.541143	6.197474	1.253574
62	1	0	−0.699436	4.190248	2.042855
63	6	0	0.687051	6.407857	−0.838194
64	1	0	1.481988	4.602147	−1.666637
65	6	0	−0.042137	6.985591	0.212446
66	1	0	−1.100129	6.654582	2.064336

67	1	0	1.075238	7.032948	-1.636517
68	1	0	-0.216235	8.058296	0.216650
69	6	0	4.029022	-0.088172	1.067928
70	1	0	5.012931	0.144163	1.476361
71	6	0	4.445447	1.446258	-0.888950
72	6	0	4.075679	1.356621	-2.238256
73	6	0	5.788334	1.758729	-0.609920
74	6	0	4.995198	1.593430	-3.264769
75	1	0	3.067978	1.072085	-2.520588
76	6	0	6.708108	1.996739	-1.628003
77	1	0	6.125967	1.808713	0.420124
78	6	0	6.314698	1.920749	-2.965462
79	1	0	4.672324	1.509424	-4.299210
80	1	0	7.738688	2.227954	-1.373045
81	1	0	7.032034	2.101036	-3.761467
82	6	0	3.354343	2.337727	1.269356
83	6	0	3.901958	3.609390	1.027956
84	6	0	2.624977	2.163284	2.464635
85	6	0	3.742553	4.650574	1.943208
86	1	0	4.461514	3.790063	0.117633
87	6	0	2.465131	3.203206	3.380736
88	1	0	2.154399	1.212898	2.690238
89	6	0	3.027251	4.453616	3.126237
90	1	0	4.181784	5.621158	1.727145
91	1	0	1.886324	3.023570	4.281407
92	1	0	2.908652	5.265784	3.838363
93	1	0	3.355829	-0.301015	1.903643
94	6	0	5.411799	-1.835631	0.026486
95	8	0	6.451716	-1.289242	0.391268
96	6	0	5.441145	-3.150518	-0.707009
97	6	0	4.329306	-3.727307	-1.337484
98	6	0	6.672071	-3.820060	-0.750730
99	6	0	4.446915	-4.956820	-1.986287
100	1	0	3.367556	-3.225519	-1.345632
101	6	0	6.788368	-5.047217	-1.398422
102	1	0	7.523725	-3.353553	-0.267375
103	6	0	5.674282	-5.620539	-2.016634
104	1	0	3.575803	-5.392902	-2.467296
105	1	0	7.747549	-5.557829	-1.421657
106	1	0	5.762922	-6.578876	-2.521890
107	1	0	-0.850399	-1.758963	1.145906
108	8	0	-0.348598	-0.191537	2.020789
109	16	0	-1.275161	0.593606	2.900787
110	8	0	-1.081572	2.062937	2.781264

111	8	0	-2.669580	0.125863	2.892417
112	6	0	-0.640777	0.222576	4.612044
113	9	0	-0.672665	-1.091706	4.855438
114	9	0	-1.378354	0.851715	5.533504
115	9	0	0.636377	0.643344	4.735984
116	8	0	4.176154	-1.289729	0.270780

Supplementary Table 51.

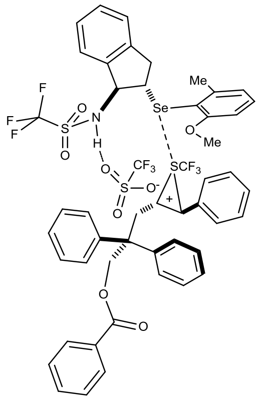

int-III'-SSS

B3LYP /6-31G(d)   195.15 K   Thermal correction to Gibbs Free Energy = 0.80606

Thermal correction to Energy = 0.899186

M062x-D3/6-311+G(d,p) (IEFPCM, Dichloromethane)   Energy = -7082.60767803

Standard orientation:

Center Number	Atomic Number	Atomic Type	Coordinates (Angstroms) X	Y	Z
1	6	0	1.275484	2.371174	0.263679
2	6	0	1.165736	0.935129	0.172031
3	6	0	2.151725	0.084202	-0.621048
4	6	0	3.612446	-0.024404	-0.040534
5	16	0	-0.585227	1.218642	-0.730830
6	6	0	-0.067040	1.491032	-2.497465
7	9	0	-0.991102	2.254140	-3.106912
8	9	0	1.113590	2.135159	-2.632509
9	9	0	0.025423	0.337668	-3.163837
10	1	0	1.705895	2.880726	-0.591802
11	1	0	0.818350	0.449008	1.083279
12	1	0	1.712812	-0.912453	-0.717031
13	1	0	2.264276	0.486209	-1.627990
14	34	0	-3.100856	0.348316	-2.137365
15	6	0	-7.230349	-2.091182	0.497735
16	6	0	-5.991537	-1.752794	-0.042998

17	6	0	−4.958430	−2.693600	−0.067895
18	6	0	−5.134262	−3.978136	0.436693
19	6	0	−6.376495	−4.315517	0.980465
20	6	0	−7.415338	−3.378820	1.010388
21	1	0	−8.039534	−1.365489	0.530358
22	1	0	−4.318273	−4.694897	0.414673
23	1	0	−6.534539	−5.309926	1.388891
24	1	0	−8.374076	−3.652626	1.442928
25	6	0	−3.727588	−2.101357	−0.728528
26	1	0	−3.689787	−2.407301	−1.780638
27	6	0	−4.000455	−0.574100	−0.632628
28	1	0	−3.587375	−0.178698	0.295517
29	6	0	−5.539385	−0.449142	−0.669232
30	7	0	−2.472207	−2.536926	−0.114667
31	16	0	−1.144350	−2.844127	−1.006465
32	8	0	−1.321554	−2.345727	−2.372179
33	8	0	0.055294	−2.543456	−0.222771
34	6	0	−1.176140	−4.705478	−1.160092
35	9	0	−2.330515	−5.079486	−1.730372
36	9	0	−0.158087	−5.115393	−1.917899
37	9	0	−1.087670	−5.266507	0.046366
38	1	0	−5.902938	−0.369174	−1.703192
39	1	0	−5.889529	0.439378	−0.135361
40	6	0	−4.008901	2.046993	−2.202802
41	6	0	−4.172246	2.829356	−1.041095
42	6	0	−4.451063	2.522303	−3.450045
43	6	0	−4.803278	4.075659	−1.118635
44	6	0	−5.071838	3.777818	−3.507704
45	6	0	−5.249566	4.538876	−2.356007
46	1	0	−4.941092	4.680148	−0.229770
47	1	0	−5.420179	4.152548	−4.466115
48	1	0	−5.739162	5.507209	−2.416576
49	8	0	−3.685013	2.292934	0.106339
50	6	0	−3.759504	3.040592	1.316200
51	1	0	−3.280250	2.415890	2.068018
52	1	0	−3.221350	3.992314	1.223137
53	1	0	−4.802365	3.237448	1.595868
54	6	0	−4.251950	1.720115	−4.715303
55	1	0	−3.187145	1.620327	−4.959749
56	1	0	−4.643163	0.702599	−4.608863
57	1	0	−4.748973	2.200875	−5.562780
58	6	0	0.888688	3.207672	1.368588
59	6	0	0.966463	4.610440	1.192750
60	6	0	0.467427	2.694425	2.617587

61	6	0	0.629001	5.472704	2.225834
62	1	0	1.297879	5.007311	0.236716
63	6	0	0.125896	3.567402	3.644310
64	1	0	0.410838	1.625594	2.796162
65	6	0	0.203802	4.950636	3.453900
66	1	0	0.693728	6.547163	2.081313
67	1	0	-0.208479	3.160714	4.593265
68	1	0	-0.063411	5.624640	4.263452
69	6	0	4.286871	-1.021045	-1.029887
70	1	0	3.824778	-2.003292	-0.896605
71	6	0	4.244653	1.380867	-0.107893
72	6	0	4.789454	1.882895	-1.301963
73	6	0	4.199120	2.245975	0.999499
74	6	0	5.275950	3.188777	-1.380929
75	1	0	4.862163	1.255459	-2.183599
76	6	0	4.684299	3.553803	0.921179
77	1	0	3.797402	1.889703	1.941689
78	6	0	5.225641	4.033065	-0.270481
79	1	0	5.701838	3.541444	-2.316550
80	1	0	4.637224	4.193144	1.798768
81	1	0	5.610129	5.047596	-0.332925
82	6	0	3.697926	-0.653965	1.363836
83	6	0	2.661816	-1.429100	1.899644
84	6	0	4.890002	-0.549221	2.104304
85	6	0	2.793728	-2.046851	3.146934
86	1	0	1.732350	-1.577684	1.363213
87	6	0	5.027930	-1.175110	3.341361
88	1	0	5.716765	0.030689	1.708576
89	6	0	3.974898	-1.923471	3.872972
90	1	0	1.953660	-2.606957	3.544627
91	1	0	5.959394	-1.071945	3.892698
92	1	0	4.075778	-2.401156	4.843933
93	1	0	4.168774	-0.717482	-2.070876
94	6	0	6.567870	-1.031111	-1.768232
95	8	0	6.249440	-0.723020	-2.901635
96	6	0	7.968666	-1.290561	-1.334252
97	6	0	8.286817	-1.705853	-0.033112
98	6	0	8.988107	-1.115150	-2.280339
99	6	0	9.616587	-1.939236	0.313484
100	1	0	7.494657	-1.846283	0.693802
101	6	0	10.314845	-1.348171	-1.928387
102	1	0	8.718783	-0.797794	-3.282311
103	6	0	10.630395	-1.760371	-0.630839
104	1	0	9.862318	-2.262960	1.321110

105	1	0	11.102783	-1.210425	-2.663655
106	1	0	11.665925	-1.943770	-0.356309
107	1	0	-2.292599	-2.324798	0.885556
108	8	0	-2.052381	-1.924695	2.620397
109	16	0	-1.534905	-0.552894	2.861400
110	8	0	-0.146176	-0.464193	3.362614
111	8	0	-1.849948	0.414228	1.773114
112	6	0	-2.571302	0.070940	4.276313
113	9	0	-3.874236	0.031272	3.961677
114	9	0	-2.252192	1.358138	4.539498
115	9	0	-2.373575	-0.646172	5.385547
116	8	0	5.685809	-1.178225	-0.748340

Supplementary Table 52.

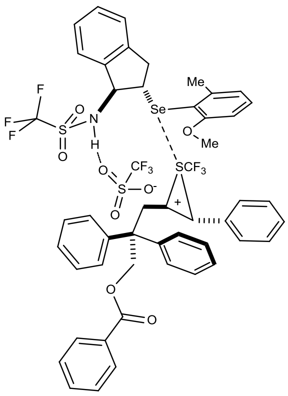

int-III'-SRR

B3LYP /6-31G(d) 195.15 K Thermal correction to Gibbs Free Energy = 0.805619

Thermal correction to Energy = 0.899384

M062x-D3/6-311+G(d,p) (IEFPCM, Dichloromethane) Energy = -7082.61635961

Standard orientation:

Center Number	Atomic Number	Atomic Type	Coordinates (Angstroms) X	Y	Z
1	6	0	1.176392	1.854336	0.870659
2	6	0	1.349985	1.359883	-0.499016
3	6	0	1.790923	-0.106594	-0.629247
4	6	0	3.298311	-0.376127	-0.295050
5	16	0	-0.494519	1.402148	-0.980054
6	6	0	-0.648845	3.158348	-1.508037
7	9	0	-0.917491	3.991680	-0.487464
8	9	0	0.471751	3.613195	-2.103752
9	9	0	-1.652122	3.251161	-2.388048
10	1	0	0.684610	1.165482	1.555316

11	1	0	1.889865	2.024885	−1.169061
12	1	0	1.164148	−0.719254	0.023870
13	1	0	1.579144	−0.418239	−1.654036
14	34	0	−3.678846	0.814578	−1.934795
15	6	0	−6.851234	−2.158292	1.444031
16	6	0	−5.857730	−1.699914	0.581430
17	6	0	−4.850392	−2.569392	0.146578
18	6	0	−4.814753	−3.898796	0.554519
19	6	0	−5.811748	−4.357053	1.422430
20	6	0	−6.820988	−3.493515	1.862602
21	1	0	−7.632895	−1.489753	1.796367
22	1	0	−4.025647	−4.562170	0.212813
23	1	0	−5.798929	−5.389404	1.761325
24	1	0	−7.585506	−3.861378	2.541798
25	6	0	−3.925771	−1.832952	−0.807111
26	1	0	−4.249718	−2.003987	−1.839674
27	6	0	−4.173434	−0.353986	−0.421247
28	1	0	−3.556161	−0.094395	0.439451
29	6	0	−5.662564	−0.321853	−0.021627
30	7	0	−2.511459	−2.222771	−0.743699
31	16	0	−1.667697	−2.671108	−2.067208
32	8	0	−2.567966	−2.799902	−3.213862
33	8	0	−0.409942	−1.926623	−2.176945
34	6	0	−1.122003	−4.405159	−1.630004
35	9	0	−2.180545	−5.213957	−1.529352
36	9	0	−0.308430	−4.863827	−2.586242
37	9	0	−0.465854	−4.385434	−0.467654
38	1	0	−6.298568	−0.190156	−0.907647
39	1	0	−5.890088	0.495058	0.669167
40	6	0	−4.586707	2.449121	−1.473845
41	6	0	−4.312060	3.079584	−0.241631
42	6	0	−5.475274	3.036221	−2.391481
43	6	0	−4.938091	4.287901	0.083410
44	6	0	−6.085257	4.253096	−2.050662
45	6	0	−5.821965	4.865626	−0.828768
46	1	0	−4.740075	4.777151	1.029555
47	1	0	−6.775940	4.713764	−2.751433
48	1	0	−6.307778	5.804296	−0.576394
49	8	0	−3.430818	2.440150	0.574906
50	6	0	−3.096205	3.032214	1.829594
51	1	0	−2.388663	2.346169	2.295535
52	1	0	−2.626883	4.012568	1.688776
53	1	0	−3.981760	3.135807	2.467172
54	6	0	−5.773605	2.396037	−3.727088

55	1	0	-4.902435	2.432515	-4.393325
56	1	0	-6.029805	1.338178	-3.609337
57	1	0	-6.599934	2.908830	-4.227832
58	6	0	1.461987	3.140352	1.383641
59	6	0	1.074193	3.399198	2.729308
60	6	0	2.133697	4.157053	0.647999
61	6	0	1.341710	4.629682	3.310366
62	1	0	0.559323	2.618901	3.280742
63	6	0	2.393565	5.379539	1.239167
64	1	0	2.441750	3.981575	-0.376033
65	6	0	1.997442	5.617103	2.566457
66	1	0	1.043211	4.826414	4.334718
67	1	0	2.904611	6.156359	0.680160
68	1	0	2.205921	6.581579	3.020212
69	6	0	4.114474	0.233044	-1.460650
70	1	0	3.750665	-0.186845	-2.403196
71	6	0	3.555358	-1.903989	-0.260101
72	6	0	4.680052	-2.408155	0.414927
73	6	0	2.723105	-2.818157	-0.921717
74	6	0	4.961662	-3.774013	0.428519
75	1	0	5.340047	-1.725641	0.939897
76	6	0	3.000678	-4.188569	-0.903943
77	1	0	1.838916	-2.485778	-1.451964
78	6	0	4.120565	-4.673791	-0.231287
79	1	0	5.836905	-4.134867	0.962409
80	1	0	2.325766	-4.870174	-1.414250
81	1	0	4.334671	-5.738971	-0.215010
82	6	0	3.623604	0.250856	1.072311
83	6	0	4.510894	1.324682	1.245507
84	6	0	2.979991	-0.264395	2.216029
85	6	0	4.742079	1.863888	2.513456
86	1	0	5.023956	1.765829	0.398720
87	6	0	3.206415	0.276220	3.480108
88	1	0	2.295892	-1.100984	2.116346
89	6	0	4.092490	1.344796	3.635085
90	1	0	5.433908	2.694705	2.620524
91	1	0	2.682909	-0.138438	4.336266
92	1	0	4.276513	1.767022	4.618891
93	1	0	4.026330	1.319713	-1.508664
94	6	0	6.428886	0.819655	-1.688627
95	8	0	6.144351	1.967385	-1.989428
96	6	0	7.817616	0.288817	-1.629257
97	6	0	8.094294	-1.048531	-1.306618
98	6	0	8.870813	1.170174	-1.915425

99	6	0	9.414869	−1.493578	−1.269230
100	1	0	7.279540	−1.729625	−1.088240
101	6	0	10.188296	0.720551	−1.876751
102	1	0	8.640097	2.200331	−2.165624
103	6	0	10.461555	−0.611991	−1.553380
104	1	0	9.627806	−2.529026	−1.019683
105	1	0	11.001239	1.405822	−2.098611
106	1	0	11.489344	−0.963120	−1.523936
107	1	0	−1.933854	−1.865040	0.035272
108	8	0	−0.821016	−1.189956	1.374801
109	16	0	−1.292744	−0.711005	2.710508
110	8	0	−0.609203	0.523328	3.173229
111	8	0	−2.756019	−0.736606	2.892519
112	6	0	−0.657153	−2.015254	3.878024
113	9	0	−1.147834	−3.218169	3.554324
114	9	0	−1.019255	−1.729559	5.136405
115	9	0	0.687243	−2.080617	3.831804
116	8	0	5.508115	−0.114815	−1.357807

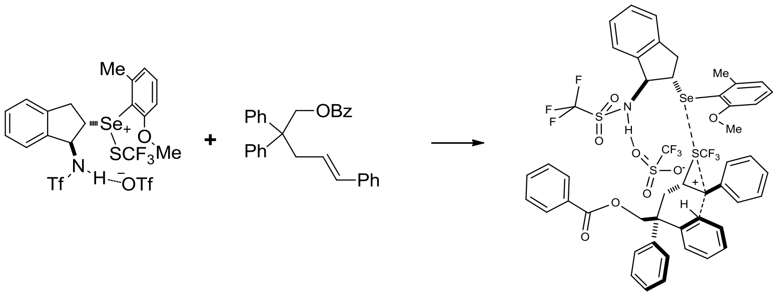

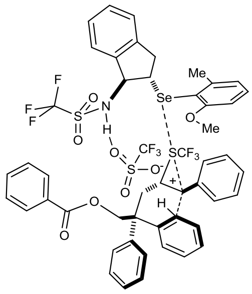

TS-II’-RSS

$\Delta G_{195.15\ K}$ = 1.7 kcal/mol

$\Delta\Delta G_{195.15\ K}$ = 0.0 kcal/mol

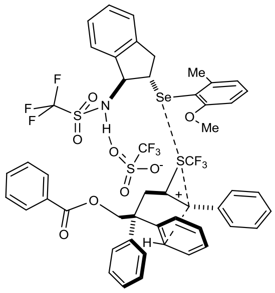

TS-II’-RRR

$\Delta G_{195.15\ K}$ = -0.2 kcal/mol

$\Delta\Delta G_{195.15\ K}$ = -1.9 kcal/mol

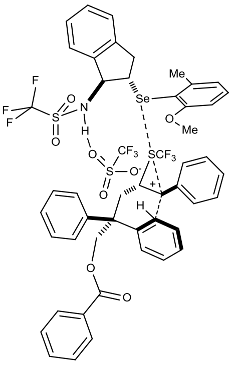

TS-II’-SSS

$\Delta G_{195.15\ K}$ = 1.0 kcal/mol

$\Delta\Delta G_{195.15\ K}$ = -0.7 kcal/mol

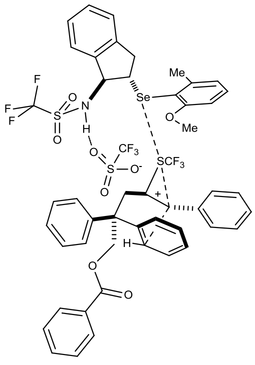

TS-II’-SRR

$\Delta G_{195.15\ K}$ = 1.2 kcal/mol

$\Delta\Delta G_{195.15\ K}$ = -0.5 kcal/mol

Supplementary Figure 186. DFT calculations for **TS-II’** of **1q**; ΔG related to **int-I**.

Supplementary Table 53.

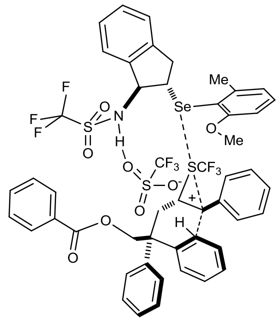

TS-II'-RSS

B3LYP /6-31G(d)   195.15 K   Thermal correction to Gibbs Free Energy = 0.808934

Thermal correction to Energy = 0.899225

M062x-D3/6-311+G(d,p)   (IEFPCM, Dichloromethane)   Energy = -7082.6080077

Standard orientation:

Center Number	Atomic Number	Atomic Type	Coordinates (Angstroms) X	Y	Z
1	6	0	-1.011080	-3.388820	0.294413
2	6	0	-0.961968	-1.906689	-0.036647
3	6	0	-2.107758	-1.383548	-0.906201
4	6	0	-3.543277	-1.639387	-0.320380
5	16	0	0.733880	-1.522145	-0.766451
6	6	0	0.514460	-2.048119	-2.505599
7	9	0	1.718716	-2.228968	-3.070657
8	9	0	-0.158790	-3.226612	-2.621772
9	9	0	-0.156245	-1.156136	-3.258179
10	1	0	-1.091649	-4.006300	-0.599065
11	1	0	-0.890265	-1.323611	0.885323
12	1	0	-1.975255	-0.307504	-1.045942
13	1	0	-2.068300	-1.861069	-1.886529
14	34	0	3.209775	0.253020	-1.940188
15	6	0	5.483398	3.803100	1.582002
16	6	0	4.623329	3.096567	0.744353
17	6	0	3.346325	3.597770	0.469710
18	6	0	2.904969	4.798081	1.016748
19	6	0	3.768535	5.503465	1.859805
20	6	0	5.047022	5.009748	2.138761
21	1	0	6.475278	3.420025	1.810284
22	1	0	1.906985	5.169406	0.802128
23	1	0	3.441207	6.437955	2.307464
24	1	0	5.705296	5.565192	2.801834
25	6	0	2.622794	2.664557	-0.484264

26	1	0	2.747069	3.020073	-1.514171
27	6	0	3.402517	1.335966	-0.298548
28	1	0	2.974676	0.775423	0.532921
29	6	0	4.844146	1.785120	0.016175
30	7	0	1.183023	2.557198	-0.247138
31	16	0	0.096087	2.592592	-1.453400
32	8	0	0.756500	2.366192	-2.738124
33	8	0	-1.109739	1.867000	-1.052392
34	6	0	-0.430376	4.385323	-1.492624
35	9	0	0.627000	5.160600	-1.766397
36	9	0	-1.366881	4.573352	-2.428802
37	9	0	-0.929064	4.742399	-0.303353
38	1	0	5.411896	1.944044	-0.911374
39	1	0	5.385899	1.040032	0.605999
40	6	0	4.591302	-1.076939	-1.781134
41	6	0	4.819171	-1.766712	-0.571946
42	6	0	5.334878	-1.405305	-2.929266
43	6	0	5.801651	-2.759724	-0.500250
44	6	0	6.306911	-2.411588	-2.841255
45	6	0	6.540807	-3.074782	-1.640407
46	1	0	5.985197	-3.285725	0.429457
47	1	0	6.885386	-2.666715	-3.725097
48	1	0	7.303865	-3.846809	-1.585461
49	8	0	4.034756	-1.400582	0.474349
50	6	0	4.140335	-2.096011	1.710470
51	1	0	3.376966	-1.653119	2.349077
52	1	0	3.938165	-3.165852	1.579079
53	1	0	5.134826	-1.963684	2.157363
54	6	0	5.090125	-0.706041	-4.245909
55	1	0	4.089025	-0.928385	-4.636496
56	1	0	5.142511	0.382357	-4.131086
57	1	0	5.822455	-1.018848	-4.996221
58	6	0	-0.021630	-3.949601	1.264323
59	6	0	0.642631	-5.144722	0.941688
60	6	0	0.239033	-3.335359	2.500076
61	6	0	1.556795	-5.709881	1.828236
62	1	0	0.451540	-5.622881	-0.016102
63	6	0	1.149938	-3.906496	3.387566
64	1	0	-0.254710	-2.407874	2.773409
65	6	0	1.808127	-5.093487	3.057666
66	1	0	2.071773	-6.628406	1.560630
67	1	0	1.347674	-3.408063	4.331788
68	1	0	2.515128	-5.537037	3.753757
69	6	0	-3.753966	-0.818204	0.997190

70	1	0	−4.618663	−1.190227	1.550256
71	6	0	−3.565622	−3.123432	−0.007140
72	6	0	−4.233764	−4.061249	−0.794648
73	6	0	−2.689581	−3.613349	1.045788
74	6	0	−4.179876	−5.422746	−0.490822
75	1	0	−4.824064	−3.727806	−1.639123
76	6	0	−2.761047	−5.016324	1.396600
77	1	0	−2.529753	−2.944503	1.890512
78	6	0	−3.463951	−5.906211	0.623268
79	1	0	−4.716630	−6.124025	−1.124611
80	1	0	−2.187546	−5.356872	2.252267
81	1	0	−3.476234	−6.964789	0.862816
82	6	0	−4.640279	−1.244339	−1.327360
83	6	0	−5.990143	−1.428534	−0.979112
84	6	0	−4.355894	−0.681892	−2.578448
85	6	0	−7.016067	−1.076715	−1.854049
86	1	0	−6.248816	−1.833686	−0.005245
87	6	0	−5.384279	−0.326569	−3.455721
88	1	0	−3.333119	−0.495451	−2.883976
89	6	0	−6.716655	−0.525614	−3.101367
90	1	0	−8.049786	−1.224672	−1.553794
91	1	0	−5.133839	0.113824	−4.416882
92	1	0	−7.514596	−0.245878	−3.783675
93	1	0	−2.872546	−0.839456	1.640449
94	6	0	−5.134146	1.124968	1.085551
95	8	0	−5.996007	0.517409	1.692046
96	6	0	−5.227345	2.557436	0.701100
97	6	0	−4.187940	3.227232	0.040260
98	6	0	−6.410450	3.239159	1.021636
99	6	0	−4.341861	4.570612	−0.300719
100	1	0	−3.270545	2.701900	−0.199199
101	6	0	−6.557182	4.579767	0.678453
102	1	0	−7.198470	2.700650	1.537640
103	6	0	−5.522579	5.246015	0.015079
104	1	0	−3.535806	5.086862	−0.811966
105	1	0	−7.474638	5.106231	0.927350
106	1	0	−5.636597	6.293336	−0.253186
107	1	0	0.820164	2.289350	0.685547
108	8	0	0.183627	1.910544	2.360804
109	16	0	0.276636	0.482388	2.752923
110	8	0	−1.019180	−0.246509	2.821039
111	8	0	1.356386	−0.273456	2.070283
112	6	0	0.825705	0.523802	4.530751
113	9	0	2.014232	1.128630	4.645861

114	9	0	0.950925	-0.737477	4.996584
115	9	0	-0.061493	1.166599	5.296389
116	8	0	-3.965471	0.557014	0.675600

Supplementary Table 54.

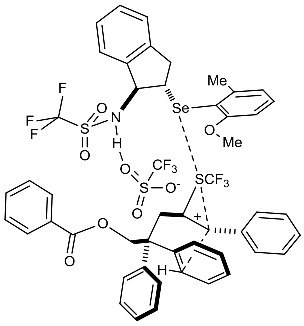

TS-II'-RRR

B3LYP /6-31G(d)   195.15 K   Thermal correction to Gibbs Free Energy = 0.805462

Thermal correction to Energy = 0.898756

M062x-D3/6-311+G(d,p)   (IEFPCM, Dichloromethane)   Energy = -7082.60772607

Standard orientation:

Center Number	Atomic Number	Atomic Type	Coordinates (Angstroms) X	Y	Z
1	6	0	-1.194362	-2.896896	0.445393
2	6	0	-1.467271	-1.821869	-0.576907
3	6	0	-2.122573	-0.554450	-0.024394
4	6	0	-3.506506	-0.775151	0.669895
5	16	0	0.251261	-1.255519	-1.138227
6	6	0	0.534223	-2.350318	-2.584566
7	9	0	1.193987	-3.477973	-2.269798
8	9	0	-0.618012	-2.720950	-3.189422
9	9	0	1.272671	-1.697678	-3.496664
10	1	0	-0.640291	-2.515846	1.304110
11	1	0	-1.994576	-2.201108	-1.451077
12	1	0	-1.467058	-0.101393	0.723066
13	1	0	-2.225196	0.185280	-0.818553
14	34	0	3.093766	0.269524	-1.950727
15	6	0	6.292685	3.055854	1.532825
16	6	0	5.236145	2.579679	0.759378
17	6	0	4.048943	3.313660	0.668741
18	6	0	3.893125	4.524797	1.334982
19	6	0	4.953433	4.999877	2.111995
20	6	0	6.143015	4.270482	2.209733

21	1	0	7.217468	2.489994	1.618423
22	1	0	2.963603	5.081174	1.256418
23	1	0	4.850674	5.939110	2.648928
24	1	0	6.956926	4.648269	2.823245
25	6	0	3.070181	2.603446	−0.249428
26	1	0	3.125402	3.035052	−1.255559
27	6	0	3.619706	1.154192	−0.263704
28	1	0	3.186883	0.600598	0.568755
29	6	0	5.142361	1.317502	−0.074852
30	7	0	1.669648	2.699731	0.174802
31	16	0	0.471587	2.997789	−0.894274
32	8	0	0.984424	2.952573	−2.263513
33	8	0	−0.742042	2.289257	−0.486186
34	6	0	0.090225	4.799704	−0.586052
35	9	0	1.184437	5.540058	−0.809846
36	9	0	−0.881712	5.198650	−1.416870
37	9	0	−0.306506	4.981824	0.673226
38	1	0	5.639298	1.452116	−1.045532
39	1	0	5.595053	0.441984	0.401154
40	6	0	4.185063	−1.319612	−1.921740
41	6	0	4.237689	−2.146624	−0.779684
42	6	0	4.896242	−1.675593	−3.081546
43	6	0	5.028504	−3.300610	−0.781292
44	6	0	5.667307	−2.846899	−3.070575
45	6	0	5.739315	−3.642316	−1.931822
46	1	0	5.089323	−3.929323	0.099500
47	1	0	6.220982	−3.124226	−3.963567
48	1	0	6.352490	−4.539774	−1.933124
49	8	0	3.487629	−1.745936	0.282261
50	6	0	3.461714	−2.543521	1.458881
51	1	0	2.734929	−2.068636	2.118897
52	1	0	3.139279	−3.568367	1.231735
53	1	0	4.448729	−2.576630	1.939020
54	6	0	4.829223	−0.832135	−4.333093
55	1	0	3.830840	−0.872542	−4.786735
56	1	0	5.025568	0.222100	−4.110645
57	1	0	5.551413	−1.180037	−5.077838
58	6	0	−0.789377	−4.252394	0.010744
59	6	0	0.239371	−4.901838	0.716197
60	6	0	−1.395071	−4.915512	−1.070895
61	6	0	0.671667	−6.168179	0.331340
62	1	0	0.703255	−4.395251	1.557892
63	6	0	−0.969448	−6.186629	−1.446462
64	1	0	−2.200825	−4.440468	−1.623233

65	6	0	0.067131	−6.813962	−0.750163
66	1	0	1.478715	−6.652119	0.873956
67	1	0	−1.443400	−6.687736	−2.285467
68	1	0	0.400668	−7.803825	−1.048698
69	6	0	−3.922704	0.590828	1.285935
70	1	0	−4.855707	0.500344	1.846359
71	6	0	−4.618370	−1.254363	−0.280656
72	6	0	−4.554700	−1.070579	−1.668022
73	6	0	−5.782084	−1.830209	0.254903
74	6	0	−5.610429	−1.466306	−2.493921
75	1	0	−3.688060	−0.600614	−2.120405
76	6	0	−6.836104	−2.226572	−0.565493
77	1	0	−5.869099	−1.962784	1.330825
78	6	0	−6.752491	−2.049189	−1.947910
79	1	0	−5.536052	−1.309519	−3.566590
80	1	0	−7.726030	−2.665850	−0.123002
81	1	0	−7.573417	−2.354888	−2.590607
82	6	0	−3.224888	−1.803073	1.754400
83	6	0	−2.965005	−3.156483	1.334829
84	6	0	−3.025752	−1.480082	3.095652
85	6	0	−2.696348	−4.158672	2.324100
86	1	0	−3.447513	−3.489408	0.420103
87	6	0	−2.661645	−2.462932	4.018058
88	1	0	−3.105564	−0.459040	3.439605
89	6	0	−2.515175	−3.810798	3.643422
90	1	0	−2.583884	−5.189139	2.002330
91	1	0	−2.477615	−2.168366	5.046951
92	1	0	−2.262617	−4.559770	4.387315
93	1	0	−3.121678	0.977097	1.919624
94	6	0	−5.325380	2.094460	0.049768
95	8	0	−6.315183	1.747466	0.662286
96	6	0	−5.298767	3.157727	−0.991663
97	6	0	−6.524580	3.684068	−1.422500
98	6	0	−4.097663	3.652062	−1.521907
99	6	0	−6.551302	4.689107	−2.385588
100	1	0	−7.440270	3.293298	−0.990715
101	6	0	−4.131155	4.665076	−2.479255
102	1	0	−3.149206	3.253916	−1.178463
103	6	0	−5.354196	5.180600	−2.914498
104	1	0	−7.502538	5.092759	−2.721757
105	1	0	−3.198958	5.053117	−2.879152
106	1	0	−5.375283	5.968836	−3.662756
107	1	0	1.389749	2.175477	1.020121
108	8	0	−0.962512	0.792634	3.028219

109	16	0	0.383945	0.179574	3.045495
110	8	0	0.427210	-1.242657	2.609140
111	8	0	1.470641	1.028198	2.490372
112	6	0	0.794791	0.064200	4.856597
113	9	0	0.824397	1.273926	5.424106
114	9	0	1.984542	-0.526153	5.039921
115	9	0	-0.139472	-0.675694	5.490986
116	8	0	-4.090408	1.557123	0.242729

Supplementary Table 55.

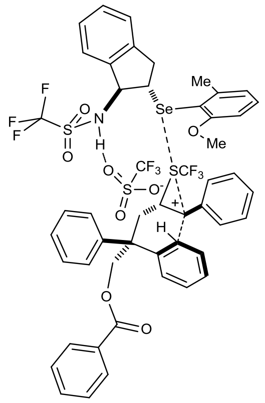

TS-II'-SSS

B3LYP /6-31G(d) 195.15 K Thermal correction to Gibbs Free Energy = 0.806161

Thermal correction to Energy = 0.898707

M062x-D3/6-311+G(d,p) (IEFPCM, Dichloromethane) Energy = -7082.60647533

Standard orientation:

Center Number	Atomic Number	Atomic Type	Coordinates (Angstroms) X	Y	Z
1	6	0	1.606507	2.611519	0.398903
2	6	0	1.142167	1.212304	0.036717
3	6	0	2.099852	0.412007	-0.845421
4	6	0	3.550496	0.227779	-0.258337
5	16	0	-0.586249	1.350592	-0.719102
6	6	0	-0.194708	1.872473	-2.431724
7	9	0	-1.277162	2.445169	-2.982413
8	9	0	0.809880	2.791155	-2.493251
9	9	0	0.177317	0.855683	-3.228293
10	1	0	1.840111	3.206385	-0.481645
11	1	0	0.902361	0.647709	0.943074
12	1	0	1.658221	-0.565572	-1.050478
13	1	0	2.219162	0.915170	-1.808769
14	34	0	-3.385144	0.388920	-2.074871

15	6	0	−6.761662	−2.614555	1.041246
16	6	0	−5.683214	−2.116371	0.313554
17	6	0	−4.567058	−2.924824	0.075008
18	6	0	−4.502252	−4.230318	0.551040
19	6	0	−5.584202	−4.727104	1.283505
20	6	0	−6.704639	−3.925510	1.525216
21	1	0	−7.631367	−1.992834	1.240667
22	1	0	−3.623451	−4.841996	0.367815
23	1	0	−5.551203	−5.740625	1.674127
24	1	0	−7.535758	−4.322395	2.102497
25	6	0	−3.549751	−2.166326	−0.758330
26	1	0	−3.677939	−2.419038	−1.817736
27	6	0	−3.965792	−0.689370	−0.523725
28	1	0	−3.464551	−0.303999	0.364367
29	6	0	−5.494083	−0.752410	−0.320331
30	7	0	−2.157259	−2.467140	−0.418767
31	16	0	−1.024017	−2.730836	−1.551122
32	8	0	−1.455259	−2.200876	−2.843785
33	8	0	0.299646	−2.443319	−0.992290
34	6	0	−1.050444	−4.587601	−1.762315
35	9	0	−2.265124	−4.969587	−2.182097
36	9	0	−0.137965	−4.960089	−2.662050
37	9	0	−0.791542	−5.187186	−0.596401
38	1	0	−6.017462	−0.694651	−1.284984
39	1	0	−5.859042	0.073395	0.297556
40	6	0	−4.388429	2.021885	−1.902979
41	6	0	−4.488282	2.699918	−0.669624
42	6	0	−4.965609	2.575953	−3.060113
43	6	0	−5.182399	3.911712	−0.585797
44	6	0	−5.647497	3.796525	−2.958184
45	6	0	−5.758947	4.451465	−1.735410
46	1	0	−5.267727	4.432276	0.360917
47	1	0	−6.097320	4.227079	−3.848808
48	1	0	−6.297659	5.393243	−1.670425
49	8	0	−3.877711	2.102318	0.385241
50	6	0	−3.855886	2.758330	1.647729
51	1	0	−3.270724	2.105688	2.293987
52	1	0	−3.370923	3.739530	1.572820
53	1	0	−4.870983	2.882181	2.048092
54	6	0	−4.846818	1.889333	−4.400712
55	1	0	−3.806498	1.869564	−4.749465
56	1	0	−5.172398	0.845039	−4.340484
57	1	0	−5.446135	2.404597	−5.157362
58	6	0	0.823587	3.392782	1.400852

59	6	0	0.565164	4.751329	1.148556
60	6	0	0.348147	2.815780	2.589858
61	6	0	-0.162977	5.515722	2.056684
62	1	0	0.924820	5.203629	0.227156
63	6	0	-0.380237	3.584677	3.496906
64	1	0	0.521433	1.766194	2.812450
65	6	0	-0.635304	4.932863	3.236912
66	1	0	-0.365240	6.561711	1.843270
67	1	0	-0.756904	3.114439	4.400456
68	1	0	-1.201422	5.528411	3.948267
69	6	0	4.327718	-0.470528	-1.403075
70	1	0	3.818796	-1.412644	-1.623551
71	6	0	4.007550	1.643653	0.035314
72	6	0	4.915863	2.354802	-0.744920
73	6	0	3.311876	2.343476	1.104694
74	6	0	5.250024	3.674475	-0.420934
75	1	0	5.404984	1.891040	-1.594639
76	6	0	3.767514	3.666754	1.462198
77	1	0	2.991899	1.720822	1.939361
78	6	0	4.692740	4.331268	0.692701
79	1	0	5.973932	4.196719	-1.041278
80	1	0	3.312097	4.149372	2.320622
81	1	0	5.001028	5.341968	0.941100
82	6	0	3.631874	-0.654855	1.002903
83	6	0	2.680167	-1.639538	1.285651
84	6	0	4.742260	-0.532815	1.857165
85	6	0	2.824834	-2.469066	2.400555
86	1	0	1.807420	-1.775820	0.658909
87	6	0	4.889601	-1.364332	2.965271
88	1	0	5.505154	0.212805	1.648131
89	6	0	3.926367	-2.337842	3.241738
90	1	0	2.051165	-3.200228	2.612160
91	1	0	5.754753	-1.248504	3.613288
92	1	0	4.031499	-2.980000	4.111962
93	1	0	4.361318	0.134097	-2.310942
94	6	0	6.702248	-0.420093	-1.794195
95	8	0	6.578662	0.326046	-2.750423
96	6	0	7.998739	-0.985714	-1.334814
97	6	0	8.092308	-1.837277	-0.223827
98	6	0	9.152455	-0.646172	-2.056142
99	6	0	9.334671	-2.339561	0.158949
100	1	0	7.197812	-2.097170	0.331117
101	6	0	10.390210	-1.152644	-1.669781
102	1	0	9.056675	0.010170	-2.914800

103	6	0	10.482246	-1.999600	-0.561805
104	1	0	9.407306	-2.999474	1.018826
105	1	0	11.282389	-0.890495	-2.231462
106	1	0	11.448445	-2.396021	-0.261095
107	1	0	-1.814192	-2.341485	0.551736
108	8	0	-1.257999	-2.167801	2.280150
109	16	0	-1.066182	-0.767791	2.746017
110	8	0	0.337057	-0.392081	3.045130
111	8	0	-1.815005	0.244911	1.960415
112	6	0	-1.893521	-0.721777	4.412409
113	9	0	-3.187721	-1.051612	4.312790
114	9	0	-1.818905	0.525999	4.921519
115	9	0	-1.301841	-1.560343	5.271033
116	8	0	5.668322	-0.818353	-1.014246

Supplementary Table 56.

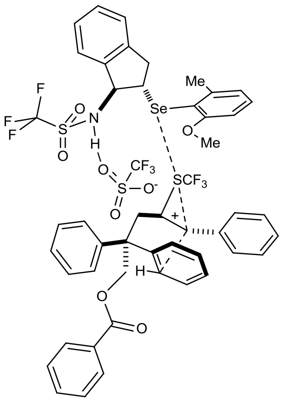

TS-II'-SRR

B3LYP /6-31G(d) 195.15 K Thermal correction to Gibbs Free Energy = 0.808086
Thermal correction to Energy = 0.899135
M062x-D3/6-311+G(d,p) (IEFPCM, Dichloromethane) Energy = -7082.60813053
Standard orientation:

Center Number	Atomic Number	Atomic Type	Coordinates (Angstroms) X	Y	Z
1	6	0	1.724355	1.617404	0.722439
2	6	0	1.493870	1.182850	-0.707693
3	6	0	1.780443	-0.296430	-1.009035
4	6	0	3.238334	-0.758829	-0.660281
5	16	0	-0.367391	1.365512	-0.995659
6	6	0	-0.464631	3.077424	-1.649491
7	9	0	-0.726909	3.990789	-0.698510
8	9	0	0.674181	3.467700	-2.268967

9	9	0	−1.454069	3.144882	−2.552965
10	1	0	1.177949	1.006326	1.441878
11	1	0	1.985300	1.842615	−1.421572
12	1	0	1.080915	−0.916064	−0.442146
13	1	0	1.579806	−0.474457	−2.068651
14	34	0	−3.642724	1.035834	−1.686823
15	6	0	−6.760827	−1.821079	1.834916
16	6	0	−5.804246	−1.401816	0.913823
17	6	0	−4.906451	−2.324886	0.365894
18	6	0	−4.945369	−3.668928	0.720031
19	6	0	−5.905475	−4.087697	1.646308
20	6	0	−6.805719	−3.171061	2.198207
21	1	0	−7.455980	−1.110385	2.275802
22	1	0	−4.239470	−4.374027	0.291508
23	1	0	−5.948073	−5.132112	1.944017
24	1	0	−7.542344	−3.509114	2.922606
25	6	0	−4.003832	−1.621275	−0.633519
26	1	0	−4.422624	−1.722328	−1.641627
27	6	0	−4.100779	−0.145743	−0.171426
28	1	0	−3.396554	0.029963	0.642165
29	6	0	−5.544906	−0.017073	0.352649
30	7	0	−2.625074	−2.121514	−0.706756
31	16	0	−1.947360	−2.602534	−2.113496
32	8	0	−2.946965	−2.607940	−3.177944
33	8	0	−0.629219	−1.991119	−2.300216
34	6	0	−1.542668	−4.394929	−1.770279
35	9	0	−2.660596	−5.109220	−1.602367
36	9	0	−0.856332	−4.900563	−2.804069
37	9	0	−0.792508	−4.487169	−0.668062
38	1	0	−6.236891	0.209966	−0.470459
39	1	0	−5.643628	0.781459	1.093855
40	6	0	−4.314257	2.747169	−1.113976
41	6	0	−3.954452	3.272126	0.144384
42	6	0	−5.112832	3.500674	−1.991427
43	6	0	−4.405440	4.537272	0.534798
44	6	0	−5.547509	4.771254	−1.586202
45	6	0	−5.201191	5.278986	−0.338282
46	1	0	−4.134064	4.945915	1.501229
47	1	0	−6.167577	5.357699	−2.258944
48	1	0	−5.550491	6.262810	−0.035816
49	8	0	−3.169887	2.474908	0.915842
50	6	0	−2.786560	2.919277	2.211563
51	1	0	−2.236686	2.086781	2.649516
52	1	0	−2.149368	3.810423	2.146047

53	1	0	−3.664946	3.140740	2.831183
54	6	0	−5.496486	2.974574	−3.354558
55	1	0	−4.625301	2.906851	−4.018441
56	1	0	−5.909415	1.962623	−3.284930
57	1	0	−6.232579	3.628701	−3.831690
58	6	0	1.717273	3.060361	1.063390
59	6	0	1.030110	3.461921	2.223846
60	6	0	2.377738	4.028971	0.287924
61	6	0	0.979538	4.808013	2.578682
62	1	0	0.534593	2.707550	2.828868
63	6	0	2.331864	5.371914	0.652184
64	1	0	2.940545	3.737236	−0.594034
65	6	0	1.628816	5.764994	1.794179
66	1	0	0.436463	5.110791	3.469653
67	1	0	2.844979	6.112499	0.045632
68	1	0	1.592373	6.814211	2.074286
69	6	0	4.182671	0.016418	−1.618803
70	1	0	4.043221	−0.387263	−2.625090
71	6	0	3.426562	−2.271591	−0.892778
72	6	0	4.634968	−2.886141	−0.516165
73	6	0	2.441566	−3.065304	−1.494431
74	6	0	4.850007	−4.244729	−0.740128
75	1	0	5.411203	−2.298103	−0.037939
76	6	0	2.657746	−4.428714	−1.716511
77	1	0	1.488413	−2.646019	−1.791075
78	6	0	3.860617	−5.023860	−1.344516
79	1	0	5.791309	−4.695844	−0.436618
80	1	0	1.867545	−5.016421	−2.174807
81	1	0	4.026107	−6.084100	−1.515838
82	6	0	3.398629	−0.379665	0.805423
83	6	0	3.549824	1.008729	1.155663
84	6	0	3.155945	−1.293960	1.830091
85	6	0	3.691871	1.363219	2.541370
86	1	0	4.056992	1.660477	0.448662
87	6	0	3.184446	−0.891534	3.165591
88	1	0	2.923212	−2.324377	1.592702
89	6	0	3.491124	0.432281	3.531266
90	1	0	3.900270	2.399450	2.787684
91	1	0	2.950893	−1.619499	3.935623
92	1	0	3.542320	0.710368	4.578824
93	1	0	3.973574	1.084881	−1.636730
94	6	0	6.313950	0.947767	−1.022429
95	8	0	5.838685	2.065748	−0.914042
96	6	0	7.756363	0.633000	−0.851872

97	6	0	8.274646	-0.650693	-1.079338
98	6	0	8.611789	1.673076	-0.460334
99	6	0	9.637376	-0.887276	-0.910062
100	1	0	7.612137	-1.449863	-1.392288
101	6	0	9.972010	1.430384	-0.291026
102	1	0	8.192532	2.660160	-0.296121
103	6	0	10.485814	0.150128	-0.515286
104	1	0	10.038638	-1.880791	-1.088727
105	1	0	10.632415	2.237280	0.013539
106	1	0	11.547995	-0.038479	-0.384647
107	1	0	-1.945482	-1.826770	0.015281
108	8	0	-0.592886	-1.301956	1.188404
109	16	0	-0.887967	-0.794373	2.565478
110	8	0	0.123011	0.174303	3.064733
111	8	0	-2.296335	-0.433022	2.802137
112	6	0	-0.604885	-2.296578	3.630859
113	9	0	-1.442622	-3.281929	3.292386
114	9	0	-0.785189	-2.001148	4.924218
115	9	0	0.659488	-2.752650	3.480953
116	8	0	5.577586	-0.156646	-1.304119

Supplementary References:

1. Brozek, L. A., Ardolino & Morken, M. J. J. P. Diastereocontrol in asymmetric allyl–allyl cross-coupling: Stereocontrolled reaction of prochiral allylboronates with prochiral allyl chlorides. *J. Am. Chem. Soc.* **133,** 16778-16781 (2011).
2. Frisch, M. J. *et al. Gaussian 09, Revision D.01* (Gaussian, Inc.: Wallingford, CT, 2009).
3. Becke, A. D. Density-functional thermochemistry. III. The role of exact exchange. *J. Chem. Phys.* **98**, 5648-5652 (1993).
4. Zhao, Y. & Truhlar, D. G. The M06 suite of density functionals for main group thermochemistry, thermochemical kinetics, noncovalent interactions, excited states, and transition elements: two new functionals and systematic testing of four M06-class functionals and 12 other functionals. *Theor. Chem. Acc.* **120**, 215-241 (2008).
5. Grimme, S., Ehrlich, S. & Goerigk, L. Effect of the damping function in dispersion corrected density functional theory. *J. Comput. Chem.* **32**, 1456-1465 (2011).

6. Krishnan, R. *et al.* Self-consistent molecular orbital methods. XX. A basis set for correlated wave functions. *J. Chem. Phys.* **72**, 650-654 (1980).

7. Tomasi, J., Mennucci, B. & Cancès, R. Quantum mechanical continuum solvation models. *Chem. Rev.* **105**, 2999-3094 (2005).

8. Luo, J., Liu, Y. & Zhao, X. Chiral selenide-catalyzed enantioselective construction of saturated trifluoromethylthiolated azaheterocycles. *Org. Lett.* **19**, 3434-3437 (2017).

9. Denmark, S. E., Hartmann, E., Kornfilt, D. J. P. & Wang, H. Mechanistic, crystallographic, and computational studies on the catalytic, enantioselective sulfenofunctionalization of alkenes. *Nat. Chem.* **6,** 1056-1064 (2014).

10. Hellmann, G. *et al.* Chiral fluorinateda-sulfonyl carbanions: Enantioselective synthesis and electrophilic capture, racemization dynamics, and structure. *Chem. Eur. J.* **19,** 3869-3897 (2013).

11. Tsang, W. C. P. *et al.* Palladium-catalyzed method for the synthesis of carbazoles via tandem C-H functionalization and C-N bond formation. *J. Org. Chem.* **73,** 7603-7610 (2008).
